# Distinctive expression patterns of *185/333 *genes in the purple sea urchin, *Strongylocentrotus purpuratus*: an unexpectedly diverse family of transcripts in response to LPS, β-1,3-glucan, and dsRNA

**DOI:** 10.1186/1471-2199-8-16

**Published:** 2007-03-01

**Authors:** David P Terwilliger, Katherine M Buckley, Virginia Brockton, Nicole J Ritter, L Courtney Smith

**Affiliations:** 1 Department of Biological Sciences, 340 Lisner Hall, George Washington University, 2023 G St NW, Washington DC, USA; 2 DPT, College of Osteopathic Medicine, Touro University, Henderson NV, USA; NJR, Butler University, Indianapolis IN, USA

## Abstract

**Background:**

A diverse set of transcripts called *185/333 *is strongly expressed in sea urchins responding to immune challenge. Optimal alignments of full-length *185/333 *cDNAs requires the insertion of large gaps that define 25 blocks of sequence called *elements*. The presence or absence of individual elements also defines a specific *element pattern *for each message. Individual sea urchins were challenged with pathogen associated molecular patterns (PAMPs) (lipopolysaccharide, β-1,3-glucan, or double stranded RNA), and changes in the *185/333 *message repertoire were followed over time.

**Results:**

Each animal expressed a diverse set of *185/333 *messages prior to challenge and a 0.96 kb message was the predominant size after challenge. Sequence analysis of the cloned messages indicated that the major element pattern expressed in immunoquiescent sea urchins was either *C1 *or *E2.1*. In contrast, most animals responding to lipopolysaccharide, β-1,3-glucan or injury, predominantly expressed messages of the *E2 *pattern. In addition to the major patterns, extensive element pattern diversity was observed among the different animals before and after challenge. Nucleotide sequence diversity of the transcripts increased in response to β-1,3-glucan, double stranded RNA and injury, whereas diversity decreased in response to LPS.

**Conclusion:**

These results illustrate that sea urchins appear to be able to differentiate among different PAMPs by inducing the transcription of different sets of *185/333 *genes. Furthermore, animals may share a suite of *185/333 *genes that are expressed in response to common pathogens, while also maintaining a large number of unique genes within the population.

## Background

Recent advances in invertebrate immunology have led to a paradigm shift in our understanding of the ways in which animals respond to immunological challenges. Previously, it was assumed that invertebrate immune response proteins were germ-line encoded and were selected over evolutionary time scales for broad recognition of conserved pathogen-associated molecular patterns (PAMPs). This was originally based on the assumption that immune diversification only occurred in jawed vertebrates through somatic recombination of the immunoglobulin (Ig) gene family that employed recombination activating gene (RAG)-mediated rearrangements of gene segments. However, recent studies on invertebrates, jawless vertebrates and higher plants have suggested that diversification of immunological responses may occur in all organisms through a variety of mechanisms. Lampreys and hagfish monoallelically express somatically diversified variable lymphocyte receptors (VLRs) that contain different numbers and sequences of leucine rich repeats (LRRs) [1-3]. In shrimp, three classes of penaeidins show significant population diversity and have varying antimicrobial activities against fungi and Gram-positive bacteria based on substitutions and deletions within the proline-rich and cysteine-rich regions [4-6]. The Down syndrome cell adhesion molecule (DSCAM) gene in *Drosophila *has 95 exons which undergo extensive mutually exclusive alternative mRNA splicing [7]. A DSCAM homologue has also been identified in the mosquito with 101 exons [8]. The DSCAM gene potentially produces ~18,000 or ~16,000 different transcripts in *Drosophila *or *Anopheles *hemocytes, respectively, that encode a diverse set of proteins putatively involved in phagocytosis [8,9]. In the tunicate, *Ciona intestinalis*, and in amphioxus, *Branchiostoma floridae*, IgV-region containing chitin-binding proteins (VCBP) are encoded by polymorphic gene families and may have gut associated antimicrobial function [10-15]. The freshwater snail, *Biomphalaria glabrata*, has 13 families of fibrinogen-related protein (FREP) genes that are expressed in response to infection with trematode parasites, and diversify through gene conversion and alternative splicing [16-18]. Finally, plant disease resistance (R) genes encoding proteins with LRR domains, which function in pathogen detection, generate diversity through a variety of mechanisms including meiotic mispairing, gene duplication and gene conversion [19-21]. The immune response of the purple sea urchin, *Strongylocentrotus purpuratus*, is likely mediated, in part, by a number of large gene families [22]. These include Toll-like receptors (TLRs), NACHT-domain containing NOD-like receptors (NLRs) [23-25] and scavenger receptor cysteine-rich repeat-containing proteins (SRCRs) [26] that have undergone expansion and diversification in the genome of this species [22]. Thus, a wide variety of organisms are able to generate a diverse immune response using a variety of molecular mechanisms [27,28].

In addition to the large gene families in the *S. purpuratus *genome mentioned above, a highly variable family of transcripts has been identified called *185/333*, which shows striking increases in response to bacteria and LPS [29,30]. Optimal alignment of the *185/333 *sequences requires the insertion of large gaps that define 25 blocks of sequence called elements [31]. Different subsets of elements are present in the mRNAs that are encoded by a single exon and do not result from alternative splicing. Elements range in size from 15 to 357 nucleotides and show sequence diversity based on single nucleotide polymorphisms (SNPs) and small insertions/deletions (indels). cDNAs that share the same set of elements (i.e., have identical element patterns) have been used to categorize sets of cDNAs. Based on the nucleotide diversity in each of the elements, sets of cDNAs are likely to be composed of members with unique nucleotide sequences. Analysis of 81 full-length cDNAs identified significant variability based on the element pattern and sequence diversity, in addition to the position and number of several different tandem and interspersed repeats [31]. Message variations were used to define 22 different element patterns, and the nucleotide diversity resulted in 67 unique mRNAs that encoded 64 different proteins. One of the 81 cDNAs had a frame shift that resulted in missense sequence and an early stop codon. The unexpected diversity of the *185/333 *mRNAs, which are expressed in response to immune challenge, has made them an intriguing gene family for understanding innate immune diversity in an invertebrate.

The *185/333 *messages were originally identified from a cDNA library constructed from coelomocyte mRNA pooled from five sea urchins responding to a large dose of a mixture of whole marine bacteria [26,29,32,33]. In the current study, we have simplified this approach by analyzing coelomocyte mRNA from individual animals challenged with single PAMPs – either lipopolysaccharide (LPS), β-1,3-glucan (Laminarin; Lam), or double stranded RNA (dsRNA) – to identify differences in the *185/333 *mRNA repertoire in response to different immunological challenges. We report that animals injected with LPS and Lam express messages with different element patterns before vs. after immune challenge, whereas a similar result was not identified for the dsRNA challenge. The sequence diversity of the transcripts decreased in response to LPS, suggesting that a specific subset of genes may be expressed in response to this molecular pattern. On the other hand, sequence diversity increased in response to dsRNA even though the major element pattern did not change, possibly suggesting that a greater number of genes with the same element pattern are expressed. The diversity of the response suggested that the innate immune system of the purple sea urchin may have evolved to detect and respond efficiently to microbial challenge with an array of 185/333 proteins.

## Results

### *185/333 *Expression

The outbred sea urchins used in this study were injected with three different PAMPs and coelomocyte mRNA was analyzed for *185/333 *expression before challenge and at various times after challenge. Messages were amplified by RT-PCR using primers that annealed to the 5' and 3' UTRs (see Methods). Sea urchins that did not respond to the initial injection, as measured by expression of *Sp056*, a C-type lectin called SpEchinoidin [34-36], received a second injection. In total, nine animals were used in this study and eight were challenged with a single PAMP. To compensate for the large variations in the genetic make-up of individual sea urchins [37,38], which may be reflected in variations in the immune response, animal 2 was injected with LPS, Lam and dsRNA, in separate experiments. For clarity, this animal has been identified as 2-LPS, 2-Lam or 2-dsRNA.

#### 185/333 transcript sizes match cDNA insert sizes

**Figure 1:**
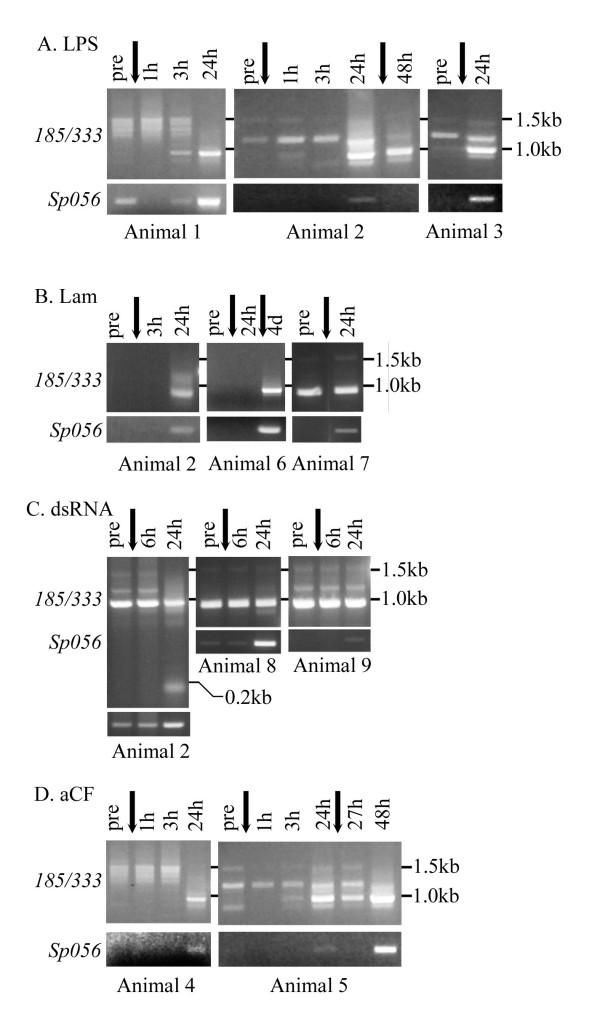
**Expression of 185/333 in response to various antigens**. Nine animals were injected with either (A) LPS (n = 3), (B) Lam (n = 3), (C) dsRNA (n = 3), or (D) aCF (n = 2). Whole coelomic fluid (wCF) samples were collected prior to injection (pre) and at various times after challenge. The expression of both *185/333 *(upper gels) and *Sp056 *(lower gels) were analyzed by RT-PCR in separate reactions and the agarose gels are shown. The vertical arrows indicate the time points at which the animals received injection(s). All animals received at least one injection at 0 h. Based on immune activation responses, three animals received second injections; animal 2-LPS and animal 5 at 24 h, animal 6 at 72 h.

**Figure 2:**
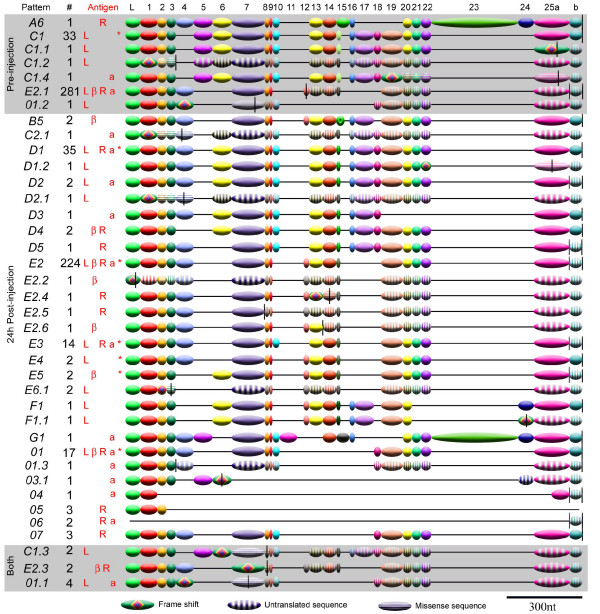
**Element patterns identified from sequences of the *185/333 *amplicons**. The element patterns (defined in [31]) for all of the clones isolated from this study are represented in graphical form. Different elements are shown as differently colored circles and ovals. Gaps are shown as horizontal lines. Short vertical black lines indicate the position of a stop codon and cDNAs with an early stop codon are designated by a decimal point followed by an integer. Elements with a diamond indicate the locations of indels that lead to a frame shift and missense amino acid sequence (elements with horizontal stripes). The sequences located 3' of the stop codon are shown to indicate that the remainder of the transcript sequence is present even though it may not be translated (vertical stripes). The frequency with which each pattern was identified (#) and the antigen that induced the pattern (Antigen) are indicated; L: LPS; β: Lam; R: dsRNA; a: aCF; *: whole bacterial challenge as identified in [31].

The range of expressed sizes of the *185/333 *messages varied among animals and was influenced by immune challenge (Figure 1). The amplified messages ranged in size from 0.16 kb to 1.5 kb, which was generally consistent with previous results [31]. Amplicons were cloned from nine animals for both pre and post challenge and 608 cDNAs were fully sequenced. From this set of cDNAs, 23 different element patterns were identified (Figure 2) of which nine had been reported previously [31]. The major band size observed for all animals on RT-PCR gels was approximately 0.96 kb (Figure 1), which corresponded in size to the major element patterns, *E2 *and *E2.1 *through *E2.6 *(511 clones; Figure 2), which were 960 nucleotides (nt) in length (Tables 1, 2). A second major band observed on gels was 1.2 kb (Figure 1), which corresponded in size with four types of element patterns: *C1 *and *C1.1 *through *C1.4 *(38 clones), *C2.1 *(1 clone), *D1 *and *D1.2 *(36 clones), and *D2 *(2 clones) (Figure 2; Table 1). The largest amplified fragment observed prior to challenge was approximately 1.5 kb (Figure 1) and corresponded in size with patterns *A6 *and *G1 *(1 clone each). The 0.85 kb amplicon corresponded with two element patterns,*01 *(17 clones) and *E6.1 *(2 clones). Thus, the observed major band sizes on gels corresponded to the major element patterns determined by sequencing the clones.

Patterns *05 *and *06*, isolated from animal 2-dsRNA, were much shorter than the other clones from both this study and from previous work [31]. Pattern *06 *(79 nt, 2 clones) only had a 5' UTR and element 25b with no other elements (Figure 2 and Additional File 1, see clone *2–2423*). Pattern *05 *(161 nt, 3 clones) had a 5'UTR followed by the leader and elements 1 and 2. The remainder of the elements were missing and no stop codon was identified (Figure 2). Pattern *05 *was similar in size to the 0.2 kb fragment amplified by RT-PCR from animal 2-dsRNA (Figure 1C). Pattern *04 *had a 5'UTR and leader followed by element 1 and part of element 25 (Figure 2). It was cloned from animal 4, although a fragment of 0.2 kb was not observed on the gel (Figure 1D).

#### LPS challenge

All animals injected with LPS (1, 2, and 3) showed similar expression patterns and changes in expression over time (Figure 1A). A C-type lectin called *Sp056 *was used to assess levels of immune activation [34-36]. It was expressed by animal 1 prior to challenge and increased expression by 24 hours (h) after challenge, whereas animal 2-LPS and animal 3 expressed *Sp056 *only after challenge. This indicated that these animals were either immunoquiescent (no expression of *Sp056*) or down-regulated (very low expression of *Sp056*) at the start of the experiment and that immune challenge with LPS activated their immune response. Prior to challenge, animal 1 expressed *185/333 *transcripts ranging in size from 0.96 kb to 1.5 kb, and after challenge began to express a predominant 0.96 kb fragment at 3 h that became the only discernable band by 24 h. Sequence analysis showed that the most common element pattern identified prior to challenge for animal 1 was *E2.1 *(11 of 15 clones; Table 1, Figure 2). After injection with LPS, *185/333 *expression changed such that *E2.1 *expression diminished and the predominant element pattern was *E2 *(28 of 31 clones). For a description of mRNAs with early stop codons (i.e., element pattern *E2.1*), see *early stop codons *below.

Animal 2-LPS expressed three message sizes prior to challenge but by 24 h after the first challenge, only two predominant message sizes were observed (Figure 1A). The 27 h sample from animal 2-LPS did not show any *185/333 *fragments, although *Sp056 *expression was detected (data not shown). By 48 h, 24 h after the second challenge, the 0.96 kb fragment was present. The only pre-challenge element pattern identified from animal 2-LPS was *C1 *(10 clones), which was not found among the post-challenge clones (Table 1, Figure 2). After injection, an assortment of element patterns was identified from 12 clones.

Although animal 3 expressed four *185/333 *sizes both before and after challenge with LPS, the major band size changed from 1.5 kb to 0.96 kb (Figure 1A). The set of clones collected prior to challenge was composed of six distinct element patterns, with the major pattern being *C1 *(12 of 18 clones). Seven different element patterns were isolated from animal 3 after injection (Table 1; Figure 2). Similar to animal 1, *E2 *(8 of 18 clones) was the predominant pattern expressed post-challenge (Table 1; Figure 2).

Analysis of 104 clones isolated from three animals challenged with LPS revealed that patterns *C1 *(22 clones) and *E2.1 *(11 clones) represented the majority of transcript types isolated prior to challenge (33 of 104 clones). In contrast, of the 61 clones analyzed from the 24 h sample, the predominant patterns were *E2 *(41 clones) or *E3 *(4 clones; Table 1; Figure 2). However, among the more rare element patterns (those isolated fewer than four times), there were differences in the types and numbers of patterns present among the three animals that had received LPS.

#### β-1,3-glucan challenge

The three animals (2, 6, and 7) injected with β-1,3-glucan (laminarin; Lam) expressed similarly sized *185/333 *messages prior to and after challenge (Figure 1B). Animal 2-Lam did not show expression of either *Sp056 *or *185/333 *prior to or at 3 h after challenge. However, by 24 h, *Sp056 *transcription was detected in addition to two sizes of *185/333 *messages, the most prominent of which was 0.96 kb. Prior to injection, the major element patterns were *E2 *(6 of 15 clones) and *E2.1 *(8 of 15 clones). After injection, however, the *E2 *pattern (33 of 36 clones) was the principal pattern identified (Table 1; Figure 2).

Neither *185/333 *nor *Sp056 *were expressed by animal 6 either prior to challenge or at 24 h. Consequently, a second injection of Lam was given 3 days (d) after the initial injection, and gene expression was measured on d 4. A single *185/333 *fragment (0.96 kb) was observed in addition to *Sp056 *expression on d 4 (Figure 1B). Because animal 6 did not express detectable *185/333 *transcripts prior to injection, the only sample used for sequencing was after the second injection (4 d). Of the 42 clones characterized, seven different patterns were identified (Table 1; Figure 2) of which the principal element pattern was *E2 *(33 clones).

Animal 7 expressed three sizes of *185/333 *messages, the predominant being 0.96 kb, which was present both before and after challenge with Lam (Figure 1B). Lack of *Sp056 *expression indicated that the immune response of animal 7 was immunoquiescent prior to injection and at 3 h, but was activated by 24 h. Of the 91 clones that were sequenced from animal 7, *E2.1 *was the most common pattern both before (40 of 47 clones) and after challenge (37 of 44) (Table 2; Figure 2)

In general, animals challenged with Lam expressed a 0.96 kb fragment 24 h after injection, which was similar to the response observed for animals challenged with LPS. Two of the three sea urchins challenged with Lam expressed a diverse set of *185/333 *transcripts including messages with rarely observed pattern types (Table 1). The predominant post challenge pattern was *E2 *for animal 2-Lam and animal 6, which was consistent with the response to LPS, and consistent with responses to injury (injections of artificial coelomic fluid [aCF], discussed below). This was in contrast to animal 7, which did not alter *185/333 *expression in response to Lam, but continued to express *E2.1 *despite changes in *Sp056 *expression that indicated that the immune response had been activated.

#### dsRNA challenge

All three animals (2, 8, and 9) challenged with dsRNA expressed similarly sized *185/333 *transcripts (Figure 1C). Prior to injection, all expressed the major 0.96 kb band, even though the absence of *Sp056 *expression suggested that all animals were immunoquiescent. Expression of *Sp056 *in these animals increased in response to challenge, indicating immune activation. After challenge, all three animals continued to express the 0.96 kb fragment, while animal 2-dsRNA and animal 9 expressed additional larger (>0.96 kb) transcripts. At 24 h, animal 2-dsRNA expressed an additional ~0.2 kb band, the only time a message of this size was observed on gels.

Most of the clones isolated and sequenced from animal 2-dsRNA both before (21 of 32 clones) and after (19 of 44 clones) challenge with dsRNA were the *E2.1 *pattern, although the *E2 *pattern was also isolated after challenge (12 of 44 clones) (Table 1). Similarly, animal 8 expressed the *E2.1 *pattern both before (44 of 46 clones) and after (28 of 41 clones) challenge. Only four *185/333 *message patterns were obtained from animal 9, of which all were identified in animal 8 and/or animal 2-dsRNA. The major pattern expressed by animal 9 was *E2.1 *both before (30 of 43 clones) and after (29 of 40 clones) challenge. Among the three animals analyzed, the sizes (Figure 1C) and element patterns (Table 2) of the *185/333 *transcripts appeared to be unaffected by dsRNA challenge, which was the only PAMP that did not induce obvious changes in the element patterns for the *185/333 *messages.

#### Response to Injury

Because each PAMP was suspended in aCF, injections of aCF into two animals (animals 4 and 5) served as injury controls (Figure 1D). Animal 4 expressed a multitude of transcripts ranging in size from 0.96 kb to 1.5 kb before injury and at 3 h after injection. However, by 24 h, only the 0.96 kb fragment was observed. Expression of *Sp056 *was only observed at 24 h, indicating that animal 4 was immunoquiescent prior to injury. Sequences of clones from animal 4 before injury yielded three different element patterns with *E2.1 *as the major pattern (5 of 7 clones; Table 1). After injury, 14 element patterns were identified (Table 1), the majority of which were *E2 *(19 of 43 clones).

Animal 5 expressed three message sizes prior to injury and at 1 h after injection of aCF (Figure 1D). At 24 h post injury, *Sp056 *expression remained undetectable, suggesting that the immune response had not been activated. Therefore, a second injection of aCF was given at 24 h, and by 48 h (24 h after a second injection of aCF), expression of *Sp056 *indicated that the animal had activated its immune system. The 0.96 kb *185/333 *transcript was first observed at 3 h and remained until 48 h post injury, becoming the predominant transcript at 24 h. The major element pattern expressed in animal 5 prior to injury was *C1 *(9 of 10 clones). After injection of aCF, six different element patterns were identified from 14 clones with *D1 *(5 clones) and *E2 *(5 clones) as the most common patterns. Overall, the animals responding to injury predominantly expressed element patterns *C1 *and/or *E2.1 *prior to injury and *E2 *after injury, although there were differences in the minor patterns expressed in each animal.

#### Animal 2

Analysis of *185/333 *expression in a single sea urchin challenged with different PAMPs over time was valuable due to the 4.5% difference among genomes of individual sea urchins [37,38]. We have assumed that this level of genetic variation among individuals would create differences in immune responses and make comparisons difficult. Therefore, we followed a single individual through the experimental protocol to eliminate the possibility that the observed differences in *185/333 *expression were the result of individual variations rather than differential responses to the PAMPs. Animal 2 received separate injections of LPS, Lam, and dsRNA in that order during the study. The animal was rested for 17 months after the LPS challenge and 15 months after challenge with Lam and the entire experiment required over 4 years to complete, including 17 months that the animal was housed in our aquaria without manipulation before the initial injection of LPS. Because the life span estimate for *S. purpuratus *is about 50 years [38], an increase in age by four years for animal 2 was not expected to have an impact on immune responsiveness. The level of immune activation for animal 2 was monitored by the expression of *Sp056 *before and after each challenge. *Sp056 *was not expressed prior to challenge with LPS and Lam, indicating immunoquiescence, but was expressed at low levels prior to dsRNA challenge, suggesting only down-regulation of the immune response (Figure 1). Animal 2 expressed a set of *185/333 *transcripts with a variety of element patterns in response to LPS, Lam and dsRNA. Prior to challenge with LPS, *C1 *was the only pattern identified (10 of 10 clones), whereas after challenge, *E2 *was the most common pattern (5 of 12 clones; Table 1). Upon return to immunoquiescence and prior to Lam challenge, animal 2 expressed both *E2 *and *E2.1 *(6 and 8 respectively of 15 clones). Following challenge with Lam, the animal again predominantly expressed *E2 *(33 of 36 clones). However, both before and after challenge with dsRNA, animal 2 primarily expressed element pattern *E2.1 *(21 of 32 clones pre-injection; 19 of 44 clones post-injection). Although the lack of *Sp056 *expression or its low expression prior to each of the three challenges suggested that animal 2 had returned to immunoquiescence, the animal expressed different *185/333 *patterns before each challenge, which may suggest that it had retained a low response level to the previous challenge. Given that animal 2 did not appear to have responded to dsRNA by changing the expressed *185/333 *element patterns, it is noteworthy that it switched from expressing pattern *E2.1 *to pattern *E2 *in response to LPS and Lam. Not only did animal 2 exhibit variation in the *185/333 *responses to challenges, but also showed variations in the expressed element patterns between challenges.

### Sequence Diversity

In addition to the variety of element patterns present in the *185/333 *mRNAs, significant nucleotide sequence diversity was observed that resulted in different deduced amino acid sequences in the encoded proteins (see Additional file 1). The mRNA diversity was analyzed in two ways: 1) ratios of nonsynonymous vs. synonymous (dn/ds) nucleotide substitutions, and 2) entropy-based sequence diversity scores [31]. Dn/ds ratios of greater than 1.0 indicate that genes are under selective pressure for diversification [39]. Diversity scores are based on the frequency of different nucleotides present at each position within a given alignment and are influenced by any nucleotide change, regardless of whether or not that change alters the protein sequence [40].

#### Nonsynonymous/synonymous ratios

Dn/ds values were obtained from individual sea urchins for sets of cDNAs (transcripts with unique nucleotide sequences but with the same element pattern) that had three or more members (Table 2). The *C1 *element pattern from animal 2-LPS prior to challenge had a dn/ds ratio greater than 1, as did the *E2 *pattern from animal 2 after Lam challenge. For the two animals that served as the injury controls, element pattern *C1 *in animal 5 prior to challenge and pattern *E2 *in animal 4 after challenge also had elevated dn/ds ratios.

The dn/ds scores for *185/333 *sequences had been calculated previously from cDNAs obtained from a library derived from coelomocytes pooled from five sea urchins [31]. In comparing the dn/ds scores of *185/333 *sequences from individual animals, we found that they were lower than those from pooled mRNA. This difference may be due either to the genetic variation among animals [37] or to different complexities of immunogens that were used to challenge the animals. To test this, the dn/ds ratios were calculated for all *E2 *sequences isolated in this study (205 clones encoding 109 unique sequences obtained from nine animals) and were compared to the dn/ds ratio reported previously for *E2 *(24 clones with 15 unique sequences) from five animals used to construct the cDNA library [31]. Notably, the dn/ds ratio for the nine animals in this study was 0.7205, whereas that for the previous dataset was 1.267. The difference between these results was unlikely due to differences in methods used to generate the clones (clones from a conventional library vs. cloned RT-PCR amplicons, see methods). It was more likely that the set of *185/333 *messages expressed by five animals responding to a complex mixture of marine bacteria [31] was more diverse than the messages expressed by nine animals responding to specific PAMPs.

#### Element diversity

In an effort to characterize the diversity among the elements and to identify possible hypervariable regions, diversity scores were calculated for specific elements before and after challenge. The diversity score of element 1 increased more than three fold after challenge with LPS and Lam, but did not change in response to dsRNA or injury (data not shown). Sequence diversity in the remaining elements expressed in response to LPS, Lam, dsRNA, or injury did not show discernable changes compared to pre-challenge diversity. Similar results were obtained from an analysis of elements in messages from animal 2 responding to all three PAMPs (data not shown). Therefore, despite changes in expressed element patterns in response to challenge, the sequence diversity of individual elements did not change (with the exception of element 1). Furthermore, the *185/333 *sequences from individual sea urchins did not reveal elements or regions that were hypervariable, which was in agreement with the previous report [31].

#### Diversity of cDNA sets

Diversity scores were also calculated for sets of cDNAs (Table 2). For all animals in which *E2 *and *E2.1 *were expressed, diversity scores for *E2 *consistently increased after challenge, whereas changes in *E2.1 *diversity scores were variable (Table 2). When cDNA sets from individuals were combined for both pre- and post-challenge, diversity scored tended to be higher than when pre- and post-challenge sets were calculated independently (Table 2; columns labeled total). Although there were some exceptions to this result, it suggested that, although the same patterns were present both before and after challenge, different sequence variants of messages with the same element patterns were expressed at different times during an immune challenge.

Diversity scores were also calculated for sequences collected from all animals responding to the same PAMP (Table 2; last 3 columns). Comparisons showed that scores increased in response to Lam and dsRNA. On the other hand, scores decreased for animals injected with LPS, and scores for the injury controls remained about the same. This indicated that a set of *185/333 *messages with increased diversity was expressed in response to Lam and dsRNA, whereas LPS and injury did not induce a similar response.

Because the *E2 *pattern was the only message type expressed by all animals, the diversity of this pattern type was calculated and compared to the *E2 *diversity scores from individuals. When all *E2 *sequences were analyzed, the diversity score was 0.0154, which was equal to or greater than the diversity scores obtained from each of the *E2 *sets from individual animals (Table 1). This suggested that although all animals expressed *E2*, the nucleotide diversity of this pattern was higher among the population than within individuals. However, because the dn/ds score for *E2 *was low, the variability in the nucleotide sequence inferred from the diversity scores indicated that the nucleotide changes did not have a greatly alter the amino acid sequence. Overall, diversity scores of cDNA sets from individual animals were lower than scores for sets of sequences from all nine animals. The implication was that sea urchins share *185/333 *genes that have common element patterns, but that the common patterns do not share identical sequence.

#### Animal 2 injected with each PAMP

Diversity scores and dn/ds ratios of cDNAs collected from animal 2 were compared among the three challenges (Table 3). Element patterns *E2 *and *E2.1 *were expressed in animal 2 both pre- and post-challenge and were employed in an analysis of sequence variability. The dn/ds ratios for *E2 *and *E2.1 *increased in response to challenge while diversity scores remained about the same. This indicated that although the nucleotide diversity did not change in response to immune challenge, the sequence variation present after challenge (dn/ds ratios) likely led to changes in the amino acid sequence.

#### Early stop codons

In addition to introducing changes in the deduced amino acid sequence, sequence diversity also introduced frame shifts and altered the positions of stop codons. Previous analysis had established the locations of three possible stop codons associated with element 25 [31]. Sequence analysis showed that in addition to the stop codons in element 25, 50% of the cDNAs had an early stop in a different element. Of the eight general pattern types (A to 0, Table 1; Figure 2) that were identified in this study, five (C to F and 0; Table 1) had members with a stop codon positioned 5' of element 25 (i.e., *E2.1, E2.2*, etc; see Figure 2). Four of the patterns with an early stop codon (*E2.1*, *E2.6*, *E2.5*, and *01.3*; Figure 2 and Additional file 2) had a SNP that changed a translated codon to a stop codon. The remaining patterns had small indels which introduced frame shifts (Figure 2, designated by a diamond in the element) that led to missense sequence and an early stop codon. If translated, these variations in the mRNAs would result in truncated proteins.

## Discussion

Previous reports analyzed cDNAs from coelomocytes pooled from five animals challenged with a mixture of marine bacteria [30,32,33]. This led to the identification of the *185/333 *transcripts, a diverse family of messages expressed in response to immune challenge [29,31]. The current study presents a simplified approach for investigating the expression patterns of *185/333 *genes by challenging individual animals with small amounts of single PAMPs. The substantial diversity evident in the *185/333 *sequences is illustrated by the identification of 23 different element patterns in this study (excluding variants with early stop codons) plus 21 element patterns described previously [31]. From these two sources of cDNAs, nine element patterns are shared for a total of 35 different patterns identified from 14 sea urchins. When the nucleotide sequences were compared for these two data sets, 62% of the messages were unique and the *C1 *element patterns (13 of 42 clones) were shared between the sets [this study; [31]].

### Changes in Element Patterns

The sea urchins employed in this study expressed different *185/333 *genes in response to various PAMPs. Changes in the expression of messages with different element patterns were identified by sequencing cDNA clones isolated from nine animals. The general trend in *185/333 *expression in response to each PAMP indicated a shift from primarily *C1 *and *E2.1 *expression in immunoquiescent coelomocytes to *E2 *as the predominant element pattern after challenge (Table 4). The shift from *C1 *to *E2 *was a significant change in the message and the encoded protein, implying a change in gene expression after immune challenge. The shift from *E2.1 *to *E2 *was significant as well, because the presence of a SNP that changed the position of the first stop codon caused a major change in the putative translated protein. This trend was not observed for animals responding to dsRNA, which maintained expression of the *E2.1 *pattern after challenge. It has been noted previously that the *185/333 *response to injury progresses more slowly than responses to bacteria [30]. Unlike the response to LPS and Lam, the animals responding to injury tended to express patterns *E2 *and *D1 *patterns (Table 4). Although there were differences in element patterns expressed in different animals, animal 2 demonstrated that individual sea urchins most likely expressed different patterns over time and in response to different challenges.

In addition to the expression of major element patterns, each animal expressed an array of mRNAs with rarely observed element patterns that were generally not shared among individuals. In response to immune challenge, the number of different rare element patterns expressed in individual sea urchins increased in seven of 10 animals (animal 2 was used three times and animal 6 did not have pre-injection data; see bottom of Table 1, Figure 2). The number of rare element patterns increased in all groups of animals responding to all PAMPs (Table 4) and 27 of 35 rare patterns were present only after challenge (including rare patterns with altered stop codon positions, and excluding all major patterns; *E2*, *E2.1*, *C1*, *D1*; Table 1). The rare patterns constituted an interesting aspect of the diversity in *185/333 *expression after challenge. These results suggested that there may be a suite of genes found in all sea urchins that encode the most common or major element patterns and that there may be a much larger number of genes encoding rare or minor element patterns that are present in low frequencies within the population.

### *185/333 *Sequence Diversity

Extensive nucleotide diversity was present in the *185/333 *cDNA sequences such that 60% of sequences were unique (using leader through element 25, excluding flanking sequence) and 50% of the cDNAs had a premature stop codon. Many of the clones with early stop codons were found only once, but others (*E2.1*, *E2.3, E6.1*, and *01.1*; Figure 2) were identified multiple times with identical element patterns and early stop codons in identical positions. One obvious source of the observed message diversity would be sequence diversity within the *185/333 *gene family. However, preliminary sequence analysis of more than 180 unique *185/333 *genes shows that, except for one pseudogene with an unusual deletion, there were no early stop codons or frame shifts within the two exons (Buckley and Smith, unpublished). This implies that post-transcriptional processing of an unknown nature may be altering mRNA sequences.

The deduced proteins have an N-terminal glycine-rich region and a C-terminal histidine-rich region [29,31]. Messages with early stop codons would putatively encode truncated 185/333 proteins that include only portions of these two regions. The most common *185/333 *patterns, *C1 *and *E2*, had stop codons at expected positions in element 25 and encoded proteins with 382 and 292 amino acids, respectively. However, *E2.1 *had an early stop in element 12 such that the E2.1 protein would only have 142 amino acids consisting of the glycine-rich region and missing most of the C-terminal histidine-rich region. Although the functionality of the proteins encoded by these abbreviated transcripts is unknown, it is an intriguing possibility that the glycine-rich region of the 185/333 proteins may have functions that can be altered by the absence of the histidine-rich region. Functional truncated proteins are unusual, but some have been demonstrated in other systems [41,42]. Preliminary analysis of 185/333 protein sizes indicates that not only do these proteins appear to multimerize, but they reveal intermediate sizes not predicted by a single full-length cDNA (Brockton et al, unpublished data). Consequently, the transcripts with early stop codons may be translated and perhaps some of the truncated proteins have a role in 185/333 activity.

### Gene Expression

Message diversity appeared to be influenced by both the type of challenge and the individual responding to the challenge. Previous work indicates that large doses of marine bacteria induce the *185/333 *response within 6 h [33], while small doses of LPS and other PAMPs induce response more slowly. Animals responding to LPS showed decreases in sequence diversity after challenge in addition to changes in element patterns. This may indicate that the LPS-induced response selectively alters transcription to a smaller set of *185/333 *genes that would putatively encode proteins that may have some association with LPS. On the other hand, animals injected with dsRNA did not show a change in the element patterns of the *185/333 *messages even though nucleotide sequence diversity of those messages increased after challenge. Consequently, it was difficult to determine whether sea urchins respond to viral molecular signatures. Because nothing is known about sea urchin-specific viruses, it is not clear whether polyGC is recognized as a threat, and it is also not clear whether the antiviral response involves 185/333 proteins. Recent studies in shrimp show that dsRNA of low complexity may not induce responses to the same degree as highly complex dsRNA [43]. Alternatively, the rate of the sea urchin response to dsRNA may be slower than that observed for LPS and Lam, and the lack of obvious changes in element patterns may be the result of terminating the experiment too early. The beginnings of responses to dsRNA may have been present because both animal 2-dsRNA and animal 8 showed post-challenge increases in messages of the *E2 *pattern, and all of the animals showed elevated sequence diversity.

The response to Lam was not striking. Two of the three animals responding to Lam showed the switch to *E2 *expression after challenge, but there was only a small increase in sequence diversity. β-1,3-glucan is isolated from giant kelp, *Laminaria*, and because kelp is a favored food of the purple sea urchin, this molecular pattern may not be recognized as a strong immunogen. On the other hand, the 185/333 proteins may not interact with fungal immunogens. Injury did not induce a change in sequence diversity of the messages, which was unlike responses to PAMPs.

Changes in message prevalence are typically attributed to changes in transcriptional activity of the genes or to decreases in the rate of message degradation. Yet it is feasible that changes in the messages could also be the result of changes in subpopulations of coelomocytes responding to immune challenge. Preliminary results indicate that the 185/333 proteins are expressed in two subpopulations of phagocytes, of which one increases in numbers in response to LPS (Brockton et al., unpublished; Majeske and Smith, unpublished). However, for a change in coelomocyte populations to alter the types and prevalence of specific *185/333 *mRNA, there must be a restricted expression of *185/333 *genes in specific coelomocytes. Restricted gene expression has been noted for VLR genes in lamprey lymphocytes [1,44], has been proposed as a theory for sea urchin TLRs [22] (see below), and is theoretically feasible for 185/333-positive coelomocytes. However, verification of this notion will require analysis of *185/333 *expression in single coelomocytes.

Because the sequence diversity and element patterns changed during the *185/333 *response to different PAMPs in individuals, this implies that sea urchins may have mechanisms to differentiate between the foreign molecular patterns that may dictate the regulation of *185/333 *gene expression. The purple sea urchin has a large family of Toll-like receptors (TLRs) that are expressed on coelomocytes [22,38]. Speculation on function of TLRs suggests that they may be restricted in expression to a few per coelomocyte and that possible combinatorial interactions on coelomocyte cell surfaces may result in diverse yet highly specific pathogen recognition capabilities [28]. Although the analysis of the *185/333 *gene promoters is underway, it is not currently known whether the TLR detection system is involved in the regulation of this gene family. However, the estimated size of the *185/333 *gene family and the diversity of the *185/333 *response appears to fit with the complexity of the TLR genes.

### Large Gene Families

Immune systems typically have large gene families, as exemplified by the Ig family, which includes the novel immune-type receptor (NITR) genes in fish [45] and the FREP genes in snails [16,18]. Often, immune genes are closely linked, which is a locus structure that appears to promote gene duplication and sequence diversification, and is an organization that has been noted for FREP genes [18]. Large families of linked genes in the sea urchin are also putatively associated with immune function including TLRs and NLRs [22,28,38]. Many of the sea urchin TLR genes are closely linked and show sequence diversity in the LRR domains, a result that is reminiscent of LRR domain diversity in the plant R genes, which are also closely linked [19-21,46,47]. Similarly, the *185/333 *genes are present in the genome as a large family of very closely linked genes ([31], Buckley and Smith, unpublished) and this may promote sequence diversification for this family as well. Although RAG homologues have been identified in the sea urchin genome [22,48], it is not known whether the echinoderm recombinases are involved in immune diversification. Consequently, for organisms lacking higher vertebrate mechanisms for somatic diversification of immune genes, a number of alternative mechanisms are expected to exist to maintain an edge in the arms race with the pathogens.

## Conclusion

There are several conclusions that can be drawn from the data presented here. Changes in element patterns and sequence diversity of the *185/333 *mRNAs occurred in response to immune challenge and the encoded proteins may be directed towards specific PAMPs. However, changes in element patterns did not always correlate with changes in sequence diversity of mRNAs (Table 4). The predominantly expressed element patterns changed in response to LPS, Lam and injury, but not in response to dsRNA. Sequence diversity increased for Lam and dsRNA, decreased for LPS, and did not change in response to injury. Although challenge with either Lam or dsRNA caused an increase in nucleotide diversity, decreased sequence diversity was observed after LPS challenge, possibly indicating transcription of a smaller set of genes. Due to the minimal changes in element patterns following challenge, it was possible that sea urchins may not have recognized polyGC as pathogenic. Differential responses to the PAMPs indicated that sea urchins could differentiate among the molecular patterns and mount different responses to those challenges.

The striking diversity of the sequences among the small sample of animals reported here and previously [31] implies that the variety of the *185/333 *sequences within the population is large. There may be a core set of genes that are shared among all purple sea urchins that are augmented by a large number of genes that are more rare in the population. Preliminary work on the structure of the *185/333 *gene locus suggests that the number and orientation of *185/333 *genes may promote diversification as has been shown for R genes in plants [46]. Future work in the area of innate immune diversification is expected to reveal a multitude of mechanisms to accomplish this end in a variety of organisms.

## Methods

### Animals

This study followed previously published protocols for generating and using immunoquiescent sea urchins [36,49-51]. Sea urchins were maintained in marine aquaria without manipulation for at least 17 months prior to the current study.

### Animal Challenge and Tissue Collection

Sea urchins were challenged with LPS in a manner similar to that described previously and was based on the estimated volume of the coelomic fluid (CF) from each animal [36,49,51]. Sea urchins were injected with 2 μg of LPS per 1 ml CF (n = 3 animals), or 4 μg of either laminarin (Lam; n = 3 animals; Sigma Aldrich, St. Louis, MO) or polyGC (dsRNA; n = 3 animals; Sigma Aldrich) per 1 ml CF to simulate an infection with gram-negative bacteria, fungi, or virus, respectively. Each antigen was suspended in aCF (10 mM CaCl_2_, 14 mM KCl, 50 mM MgCl_2_, 398 mM NaCl, 1.7 mM Na_2_HCO_3_, 25 mM Na_2_SO_4_) and injected through the peristomial membrane directly into the coelomic cavity of the sea urchin. Samples of whole coelomic fluid (wCF; fluid plus cells) were taken 15 min before challenge and 24 h after challenge. Two animals were used as injury controls and received injections of aCF using the same timing and volumes as the PAMP challenges. Animal 2-LPS and animal 5 were injected a second time at 24 h and samples were taken at 48 h. Animal 6 received a second injection of Lam at 3 days. Expression of *Sp056 *(GenBank Acc. # AY336600) was were used to assess immune activation [34,36]. Amplification of *SpL8 *(a homologue of ribosomal L8) and actin were used as controls for gel loading and cDNA integrity (data not shown). These controls indicated that the absence of amplicons for *185/333 *and *Sp056 *was due to expression that was below the detection sensitivity of the gel imaging system rather than a protocol failure.

Samples of wCF were collected into anti-coagulant buffer (aCF with 50 mM imidazole pH 7.4, 30 mM EDTA), centrifuged at 10,000 × *g *for 5 min at 4°C and the pelleted coelomocytes were lysed and stored in RNA *later *(Ambion Diagnostics, Austin, TX). Total RNA was isolated using the RNeasy Mini Kit (Qiagen, Valencia, CA) per the manufacturer. Total RNA samples were treated with DNase using the DNA-*free *kit per the manufacturer (Ambion, Austin, TX).

### RT-PCR

Reverse transcription (RT) was performed on 1 μg of total RNA as previously described [36]. A pair of primers (5'UTR forward: TAG CAT CGG AGA GAC CT; 3'UTR reverse: AAA TTC TAC ACC TCG GCG AC) specific for *185/333 *were designed to anneal in the untranslated regions (UTRs) of the known *185/333 *transcripts and amplified the entire coding region [29,31]. Expression of *Sp056 *(GenBank Acc. # AY336600, [35]) was analyzed with Sp056F (GCA CAG CCA GCA ACC AGC ACT ACA AT) and Sp056R (ACG CCG ATG GGT TCT ACA GTG AAG GT) primers, which amplified a 640 bp fragment of the message. Amplification of different transcripts was always done in separate reactions. Each PCR reaction of 20 μl had 1 μM forward primer, 1 μM reverse primer, 0.5 μM of each deoxynucleotide, 2 mM MgCl_2_, 1× company supplied buffer, 0.5 U of *ExTaq *polymerase (Takara Bio, Otsu, Shiga, Japan), plus 1 μl of the RT reaction, which served as the cDNA template. Reactions were performed with a Mastercycler Gradient thermocycler (Eppendorf, Hamburg, Germany) using the following program: 94°C for 5 min, followed by 25 cycles of 94°C for 30 sec, 55°C (*185/*333) or 68°C (*Sp056*) for 30 s, 72°C for 2 min with a final hold at 4°C. Images of the agarose gels were obtained with a DC120 digital camera (Eastman Kodak Co. New Haven, CT) with Digital Science1D software ver. 3.0.0 (Eastman Kodak Co.).

### Cloning and Sequencing

Cloning into pCR4-TOPO vector was performed with 1 μl of the PCR reaction using the TOPO-TA Cloning kit for Sequencing (Invitrogen, Carlsbad, CA) per the manufacturer. The cDNA clones were grown overnight and plasmids were isolated using Wizard Plus SV Minipreps DNA Isolation Kit (Invitrogen).

Plasmids were sequenced directly using universal T7 and T3 primers with 2× to 9× coverage. Sequencing was completed using either the DTCS Quick Start Kit for Dye Terminator Cycle Sequencing (Beckman Coulter, Fullerton, CA) and analyzed on a CEQ8000 DNA sequencer (Beckman Coulter) or plasmids were sent to SeqWright DNA Technology Services (Houston, TX). Sequences have been deposited in GenBank with accession numbers EF065719 through EF066327.

The fidelity of the *Taq *polymerase used in this study was tested to ensure that sequence diversity observed was not a byproduct of *Taq*-induced errors. One clone (*Sp0228*, GenBank Acc. # DQ183174; see Table S1 in [31]) with an insert of 1519 nt was amplified by PCR and re-cloned as described above. Subclones (n = 24) were sequenced and alignments were used to identify and quantify the number of nucleotide errors. An error rate of 1/18,228 nt was calculated (2 errors in 36,456 bases {24 clones multiplied by 1,519 nt}), which equates to one *Taq*-induced error in about 18 clones (608 cDNA clones yielded 602,635 nt).

### Bioinformatics

Sequence alignments were performed with BioEdit [52]. WinClada ver. 1.00.08 [53] was used to identify identical sequences from the nucleotide alignments. Neighbor-joining phylogenetic trees were created in MEGA3 [54] and employed in Phylogenetic Analysis by Maximum Likelihood (PAML) [55] using three dn/ds models (M1, M2 and M20) to identify statistically significant non-synonymous and synonymous (dn/ds) ratios. Diversity scores were acquired using an in-house Perl script implementing an entropy equation [40] as previously described [31].

## Authors' contributions

DPT was responsible for the experimental design, immune challenges, RT-PCR, electrophoresis, cloning, sequencing, sequence analysis, and bioinformatic analysis. KMB completed the bioinformatic analysis on all of the sequences, and determined diversities of nucleotide and amino acid sequences for elements and cDNA sets. VB and NJR provided significant contributions to the acquisition and analysis of the data. LCS directed the research, assisted in data analysis and produced the final version of the manuscript. DPT produced the final draft of the manuscript and KMB provided significant editorial improvements. All authors approved the final manuscript.

## Supplementary Material

Additional File 1 Nucleotide alignment for *185/333 *cDNAs isolated from individual sea urchins. Alignment of the *185/333 *cDNAs showing the sequence diversity, part of the 5' untranslated region, the start codon, indels, gaps, elements, and stop codons. Click here for file

Additional File 2 Alignment of amino acid sequences deduced from the *185/333 *cDNAs. Alignment of the translated cDNA sequences showing the 185/333 proteins and their diversity, gaps, elements, repeats, glycosylation sites, motifs and stop codons. Click here for file

## Figures and Tables

**Table 1:** Element patterns of *185/333 *cDNAs expressed before and after challenge

**Challenge:**		**LPS**						**Laminarin**					**dsRNA**						**aCF**				**Total**		
						
**Animal**		**1**		**2**		**3**		**2**		**6**	**7**		**2**		**8**		**9**		**4**		**5**				
													
**Pattern**	**Size (kb)**	-	+	-	+	-	+	-	+	+	-	+	-	+	-	+	-	+	-	+	-	+	-	+	**Total**
*A6*	1.50	**·**	**·**	**·**	**·**	**·**	**·**	**·**	**·**	**·**	**·**	**·**	**·**	**·**	**·**	**·**	1	0	**·**	**·**	**·**	**·**	1	0	1
*B3*	1.04	**·**	**·**	**·**	**·**	**·**	**·**	**·**	**·**	**·**	**·**	**·**	**·**	**·**	**·**	**·**	**·**	**·**	0	2	0	1	0	3	3
*B5*	1.07	**·**	**·**	**·**	**·**	**·**	**·**	**·**	**·**	2	**·**	**·**	**·**	**·**	**·**	**·**	**·**	**·**	**·**	**·**	**·**	**·**	0	2	2
*C1*	1.18	**·**	**·**	10	0	12	3	**·**	**·**	**·**	**·**	**·**	1	0	**·**	**·**	**·**	**·**	**·**	**·**	9	0	32	3	35
*C1.1*	1.18	**·**	**·**	**·**	**·**	1	0	**·**	**·**	**·**	**·**	**·**	**·**	**·**	**·**	**·**	**·**	**·**	**·**	**·**	**·**	**·**	1	0	1
*C1.2*	1.18	**·**	**·**	**·**	**·**	1	1	**·**	**·**	**·**	**·**	**·**	**·**	**·**	**·**	**·**	**·**	**·**	**·**	**·**	**·**	**·**	1	1	2
*C1.3*	1.18	**·**	**·**	**·**	**·**	1	1	**·**	**·**	**·**	**·**	**·**	**·**	**·**	**·**	**·**	**·**	**·**	**·**	**·**	**·**	**·**	1	1	2
*C1.4*	1.18	**·**	**·**	**·**	**·**	**·**	**·**	**·**	**·**	**·**	**·**	**·**	**·**	**·**	**·**	**·**	**·**	**·**	**·**	**·**	1	0	1	0	1
*C2.1*	1.15	**·**	**·**	**·**	**·**	**·**	**·**	**·**	**·**	**·**	**·**	**·**	**·**	**·**	**·**	**·**	**·**	**·**	**·**	**·**	0	1	0	1	1
*D1*	1.16	**·**	**·**	**·**	**·**	0	1	**·**	**·**	**·**	**·**	**·**	1	2	0	2	3	2	1	5	0	5	5	17	22
*D1.2*	1.16	**·**	**·**	**·**	**·**	0	1	**·**	**·**	**·**	**·**	**·**	**·**	**·**	**·**	**·**	**·**	**·**	**·**	**·**	**·**	**·**	0	1	1
*D2*	1.16	**·**	**·**	0	1	**·**	**·**	**·**	**·**	**·**	**·**	**·**	**·**	**·**	**·**	**·**	**·**	**·**	0	1	**·**	**·**	0	2	2
*D2.1*	1.16	**·**	**·**	0	1	**·**	**·**	**·**	**·**	**·**	**·**	**·**	**·**	**·**	**·**	**·**	**·**	**·**	**·**	**·**	**·**	**·**	0	1	1
*D3*	0.99	**·**	**·**	**·**	**·**	**·**	**·**	**·**	**·**	**·**	**·**	**·**	**·**	**·**	**·**	**·**	**·**	**·**	0	1	**·**	**·**	0	1	1
*D4*	1.09	**·**	**·**	**·**	**·**	**·**	**·**	**·**	**·**	1	**·**	**·**	0	1	**·**	**·**	**·**	**·**	**·**	**·**	**·**	**·**	0	2	2
*D5*	1.09	**·**	**·**	**·**	**·**	**·**	**·**	**·**	**·**	**·**	**·**	**·**	0	1	**·**	**·**	**·**	**·**	**·**	**·**	**·**	**·**	0	1	1
*E2*	0.96	4	28	0	5	0	8	6	33	33	7	7	9	12	2	8	9	9	1	19	0	5	38	167	205
*E2.1*	0.96	11	0	**·**	**·**	**·**	**·**	8	2	1	40	37	21	19	44	28	30	29	5	5	**·**	**·**	159	121	280
*E2.2*	0.96	**·**	**·**	**·**	**·**	**·**	**·**	**·**	**·**	1	**·**	**·**	**·**	**·**	**·**	**·**	**·**	**·**	**·**	**·**	**·**	**·**	0	1	1
*E2.3*	0.96	**·**	**·**	**·**	**·**	**·**	**·**	1	0	**·**	**·**	**·**	0	1	**·**	**·**	**·**	**·**	**·**	**·**	**·**	**·**	1	1	2
*E2.4*	0.96	**·**	**·**	**·**	**·**	**·**	**·**	**·**	**·**	**·**	**·**	**·**	**·**	**·**	0	1	**·**	**·**	**·**	**·**	**·**	**·**	0	1	1
*E2.5*	0.96	**·**	**·**	**·**	**·**	**·**	**·**	**·**	**·**	**·**	**·**	**·**	0	1	**·**	**·**	**·**	**·**	**·**	**·**	**·**	**·**	0	1	1
*E2.6*	0.96	**·**	**·**	**·**	**·**	**·**	**·**	**·**	**·**	1	**·**	**·**	**·**	**·**	**·**	**·**	**·**	**·**	**·**	**·**	**·**	**·**	0	1	1
*E3*	0.93	**·**	**·**	0	2	0	2	**·**	**·**	**·**	**·**	**·**	**·**	**·**	0	1	**·**	**·**	0	1	**·**	**·**	0	6	6
*E4*	0.95	0	1	**·**	**·**	**·**	**·**	**·**	**·**	**·**	**·**	**·**	**·**	**·**	**·**	**·**	**·**	**·**	**·**	**·**	**·**	**·**	0	1	1
*E5*	0.96	**·**	**·**	**·**	**·**	**·**	**·**	0	1	**·**	**·**	**·**	**·**	**·**	**·**	**·**	**·**	**·**	**·**	**·**	**·**	**·**	0	1	1
*E6.1*	0.87	0	2	**·**	**·**	**·**	**·**	**·**	**·**	**·**	**·**	**·**	**·**	**·**	**·**	**·**	**·**	**·**	**·**	**·**	**·**	**·**	0	2	2
*F1*	1.06	**·**	**·**	0	1	**·**	**·**	**·**	**·**	**·**	**·**	**·**	**·**	**·**	**·**	**·**	**·**	**·**	**·**	**·**	**·**	**·**	0	1	1
*F1.1*	1.06	**·**	**·**	0	1	**·**	**·**	**·**	**·**	**·**	**·**	**·**	**·**	**·**	**·**	**·**	**·**	**·**	**·**	**·**	**·**	**·**	0	1	1
*G1*	1.44	**·**	**·**	**·**	**·**	**·**	**·**	**·**	**·**	**·**	**·**	**·**	**·**	**·**	**·**	**·**	**·**	**·**	0	1	**·**	**·**	0	1	1
*01*	0.86	**·**	**·**	0	1	0	1	**·**	**·**	3	**·**	**·**	**·**	**·**	0	1	**·**	**·**	0	3	0	1	0	10	10
*01.1*	0.86	**·**	**·**	**·**	**·**	2	0	**·**	**·**	**·**	**·**	**·**	**·**	**·**	**·**	**·**	**·**	**·**	0	1	0	1	2	2	4
*01.2*	0.86	**·**	**·**	**·**	**·**	1	0	**·**	**·**	**·**	**·**	**·**	**·**	**·**	**·**	**·**	**·**	**·**	**·**	**·**	**·**	**·**	1	0	1
*01.3*	0.86	**·**	**·**	**·**	**·**	**·**	**·**	**·**	**·**	**·**	**·**	**·**	**·**	**·**	**·**	**·**	**·**	**·**	0	1	**·**	**·**	0	1	1
*03.1*	0.56	**·**	**·**	**·**	**·**	**·**	**·**	**·**	**·**	**·**	**·**	**·**	**·**	**·**	**·**	**·**	**·**	**·**	0	1	**·**	**·**	0	1	1
*04*	0.2	**·**	**·**	**·**	**·**	**·**	**·**	**·**	**·**	**·**	**·**	**·**	**·**	**·**	**·**	**·**	**·**	**·**	0	1	**·**	**·**	0	1	1
*05*	0.16	**·**	**·**	**·**	**·**	**·**	**·**	**·**	**·**	**·**	**·**	**·**	0	3	**·**	**·**	**·**	**·**	**·**	**·**	**·**	**·**	0	3	3
*06*	0.08	**·**	**·**	**·**	**·**	**·**	**·**	**·**	**·**	**·**	**·**	**·**	0	1	**·**	**·**	**·**	**·**	0	1	**·**	**·**	0	2	2
*07*	0.86	**·**	**·**	**·**	**·**	**·**	**·**	**·**	**·**	**·**	**·**	**·**	0	3	**·**	**·**	**·**	**·**	**·**	**·**	**·**	**·**	0	3	3
Total clones		15	31	10	12	18	18	15	36	42	42	44	32	44	46	41	43	40	7	43	10	14	243	365	608
Different patterns		2	3	1	7	6	8	3	3	7	7	2	4	10	2	6	4	3	3	14	2	6	12	35	39

-, pre challenge; +, post challenge

**Table 2:** Sequence diversity in cDNA sets^#^

			Pre-injection				Post-injection				Total				Diversity Score		
								
Challenge	Animal	cDNA Pattern	# of clones	unique clones	dn/ds	Div. score	# of clones	unique clones	dn/ds	Div. score	# of clones	unique clones	dn/ds	Div. score	Pre	Post	Total
**LPS**	**1**	*E2*	4	3	0.93	0.002	28	12	0.88	0.009	32	14	0.50	0.010			
		
	**2**	*C1*	10	9	**1.87**	0.012	·	·	·	·	·	·	·	·			
		
	**3**	*E3*	·	·	·	·	6	5	0.22	0.003	·	·	·	·	0.365	0.216	0.325
		*C1*	12	8	0.36	0.005	·	·	·	+	15	10	0.63	0.007			
		*E2*	·	·	·	·	8	7	0.78	0.012	·	·	·	·			

**β-1,3-glucan (Lam)**	**2**	*E2*	6	6	**1.00**	0.007	32	15	**1.16**	0.009	38	20	**1.47**	0.010			
		*E2.1*	8	3	0.54	0.005	·	·	·	+	10	4	0.72	0.007			
		
	**6**	*1*	·	·	·	·	3	3	0.46	0.003	·	·	·	·			
		*E2*	·	·	·	·	33	20	0.77	0.009	·	·	·	·	0.287	0.320	0.323
		
	**7**	*E2*	7	4	0.72	0.007	7	7	0.49	0.008	14	9	0.55	0.009			
		*E2.1*	39	12	0.42	0.007	37	12	0.50	0.009	76	19	0.47	0.006			

**dsRNA**	**2**	*E2*	9	8	0.73	0.011	12	8	0.91	0.014	21	14	0.88	0.017			
		*E2.1*	21	7	0.96	0.006	19	4	0.24	0.002	40	10	0.62	0.004			
		
	**8**	*E2*	2	2	·	+	8	6	0.97	0.008	10	7	0.82	0.008			
		*E2.1*	44	19	0.72	0.01	28	10	**1.00**	0.008	72	28	0.93	0.007	0.198	0.336	0.231
		
	**9**	*D1*	3	3	0.15	0.002	·	·	·	+	5	5	0.49	0.003			
		*E2*	9	9	**1.00**	0.005	9	7	0.87	0.007	18	14	**1.14**	0.008			
		*E2.1*	30	8	0.79	0.007	29	8	**1.00**	0.007	59	15	**1.03**	0.009			

**aCF**	**4**	*D1*	·	·	·	·	5	5	0.71	0.023	·	·	·	·			
		*E2*	·	·	·	+	19	16	**1.16**	0.014	20	17	**1.00**	0.015			
		*E2.1*	·	·	·	+	5	4	**1.00**	0.002	10	4	**1.00**	0.002			
															0.328	0.337	0.370
	**5**	*C1*	9	8	**1.70**	0.006	·	·	·	·	·	·	·	·			
		*D1*	·	·	·	·	5	5	0.99	0.015	·	·	·	·			
		*E2*	·	·	·	·	5	4	**1.00**	0.004	·	·	·	·			

^# ^Modeled alignments have been used to understand diversity scores. A diversity score of 0.0954 represents an alignment in which 10% of the sequences have variations at 30% of nucleotide positions. A score of 0.0197 indicates that 1% of the sequences have a single change in 30% of the nucleotide positions [29].

+ Diversity scores were not determined because fewer than three unique sequences were identified. However, sequences were included for calculating total diversity scores.

**Table 3:** Sequence diversity of cDNA sets from animal 2

	Pre-injection				Post-injection				Total			
			
cDNA Pattern	# of clones	unique clones	dn/ds	Diversity score	# of clones	unique clones	dn/ds	Diversity score	# of clones	unique clones	dn/ds	Diversity Score
*E2.1*	29	9	0.61	0.003	21	5	0.99	0.002	50	13	0.68	0.003
*E2*	15	14	**1.02**	0.011	45	22	**1.22**	0.010	60	33	**1.27**	0.012

**Table 4:** Summary of responses to PAMPs

PAMP	Major Pattern		# of Different Minor Patterns^§^		Diversity Change
	Pre	Post	Pre	Post	
LPS	*E2.1, C1*	*E2*	6	13	decrease
Lam*	*E2.1*	*E2*	3	8	increase
dsRNA	*E2.1*	*E2.1*	3	12	increase
injury	*E2.1, C1*	*E2, D1*	3	13	no change

*No pre-injection data were collected for animal 6.

^§^Number of different minor patterns combined from the three animals responding to a single PAMP.

## References

[B1] Adler NM, Rogozin IB, Iyer LM, Glazko GV, Cooper MD, Pancer Z (2005). Diversity and function of adaptive immune receptors in a jawless vertebrate. Science.

[B2] Pancer Z, Amemiya CT, Ehrhardt GR, Ceitlin J, Gartland GL, Cooper MD (2004). Somatic diversification of variable lymphocyte receptors in the agnathan sea lamprey. Nature.

[B3] Pancer Z, Saha NR, Kasamatsu J, Suzuki T, Amemiya CT, Kasahara M, Cooper MD (2005). Variable lymphocyte receptors in hagfish. Proc Natl Acad Sci U S A.

[B4] Cuthbertson BJ, Bullesbach EE, Fievet J, Bachere E, Gross PS (2004). A new class (penaeidin class 4) of antimicrobial peptides from the Atlantic white shrimp (*Litopenaeus setiferus*) exhibits target specificity and an independent proline-rich-domain function. Biochem J.

[B5] Cuthbertson BJ, Shepard EF, Chapman RW, Gross PS (2002). Diversity of the penaeidin antimicrobial peptides in two shrimp species. Immunogenetics.

[B6] Cuthbertson BJ, Yang Y, Bachere E, Bullesbach EE, Gross PS, Aumelas A (2005). Solution structure of synthetic penaeidin-4 with structural and functional comparisons with penaeidin-3. J Biol Chem.

[B7] Schmucker D, Clemens JC, Shu H, Worby CA, Xiao J, Muda M, Dixon JE, Zipursky SL (2000). *Drosophila* Dscam is an axon guidance receptor exhibiting extraordinary molecular diversity. Cell.

[B8] Dong Y, Taylor HE, Dimopoulos G (2006). AgDscam, a hypervariable immunoglobulin domain-containing receptor of the *Anopheles gambiae* innate immune system. PLoS Biol.

[B9] Watson FL, Puttmann-Holgado R, Thomas F, Lamar DL, Hughes M, Kondo M, Rebel VI, Schmucker D (2005). Extensive diversity of Ig-superfamily proteins in the immune system of insects. Science.

[B10] Azumi K, De Santis R, De Tomaso A, Rigoutsos I, Yoshizaki F, Pinto MR, Marino R, Shida K, Ikeda M, Ikeda M, Arai M, Inoue Y, Shimizu T, Satoh N, Rokhsar DS, Du Pasquier L, Kasahara M, Satake M, Nonaka M (2003). Genomic analysis of immunity in a Urochordate and the emergence of the vertebrate immune system: "waiting for Godot". Immunogenetics.

[B11] Cannon JP, Haire RN, Litman GW (2002). Identification of diversified genes that contain immunoglobulin-like variable regions in a protochordate. Nat Immunol.

[B12] Cannon JP, Haire RN, Rast JP, Litman GW (2004). The phylogenetic origins of the antigen-binding receptors and somatic diversification mechanisms. Immunol Rev.

[B13] Cannon JP, Haire RN, Schnitker N, Mueller MG, Litman GW (2004). Individual protochordates have unique immune-type receptor repertoires. Curr Biol.

[B14] Litman GW, Cannon JP, Dishaw LJ (2005). Reconstructing immune phylogeny: new perspectives. Nat Rev Immunol.

[B15] Litman GW, Cannon JP, Rast JP (2005). New insights into alternative mechanisms of immune receptor diversification. Adv Immunol.

[B16] Loker ES, Adema CM, Zhang SM, Kepler TB (2004). Invertebrate immune systems--not homogeneous, not simple, not well understood. Immunol Rev.

[B17] Nowak TS, Loker ES (2005). *Echinostoma paraensei*: differential gene transcription in the sporocyst stage. Exp Parasitol.

[B18] Zhang SM, Adema CM, Kepler TB, Loker ES (2004). Diversification of Ig superfamily genes in an invertebrate. Science.

[B19] Hulbert SH, Webb CA, Smith SM, Sun Q (2001). Resistance gene complexes: evolution and utilization. Annu Rev Phytopathol.

[B20] Parniske M, Hammond-Kosack KE, Golstein C, Thomas CM, Jones DA, Harrison K, Wulff BB, Jones JD (1997). Novel disease resistance specificities result from sequence exchange between tandemly repeated genes at the Cf-4/9 locus of tomato. Cell.

[B21] Parniske M, Jones JD (1999). Recombination between diverged clusters of the tomato Cf-9 plant disease resistance gene family. Proc Natl Acad Sci U S A.

[B22] Hibino T, Loza-Coll M, Messier C, Majeske AJ, Cohen AH, Terwilliger DP, Buckley KM, Brockton V, Nair SV, Berney K, Fugmann SD, Anderson MK, Pancer Z, Cameron RA, Smith LC, Rast JP (2006). The immune gene repertoire encoded in the purple sea urchin genome. Dev Biol.

[B23] Inohara N, Chamaillard M, McDonald C, Nunez G (2005). NOD-LRR proteins: role in host-microbial interactions and inflammatory disease. Annu Rev Biochem.

[B24] Kufer TA, Fritz JH, Philpott DJ (2005). NACHT-LRR proteins (NLRs) in bacterial infection and immunity. Trends Microbiol.

[B25] Ting JP, Kastner DL, Hoffman HM (2006). CATERPILLERs, pyrin and hereditary immunological disorders. Nat Rev Immunol.

[B26] Pancer Z (2000). Dynamic expression of multiple scavenger receptor cysteine-rich genes in coelomocytes of the purple sea urchin. Proc Natl Acad Sci U S A.

[B27] Flajnik MF, Du Pasquier L (2004). Evolution of innate and adaptive immunity: can we draw a line?. Trends Immunol.

[B28] Rast JP, Smith LC, Loza-Coll M, Hibino T, Litman GW (2006). Genomic insights into the immune system of the sea urchin. Science.

[B29] Nair SV, Del Valle H, Gross PS, Terwilliger DP, Smith LC (2005). Macroarray analysis of coelomocyte gene expression in response to LPS in the sea urchin. Identification of unexpected immune diversity in an invertebrate. Physiol Genomics.

[B30] Rast JP, Pancer Z, Davidson EH (2000). New approaches towards an understanding of deuterostome immunity. Curr Top Microbiol Immunol.

[B31] Terwilliger DP, Buckley KM, Mehta D, Moorjani PG, Smith LC (2006). Unexpected diversity displayed in cDNAs expressed by the immune cells of the purple sea urchin, *Strongylocentrotus purpuratus*. Physiological Genomics.

[B32] Cameron RA, Mahairas G, Rast JP, Martinez P, Biondi TR, Swartzell S, Wallace JC, Poustka AJ, Livingston BT, Wray GA, Ettensohn CA, Lehrach H, Britten RJ, Davidson EH, Hood L (2000). A sea urchin genome project: sequence scan, virtual map, and additional resources. Proc Natl Acad Sci U S A.

[B33] Pancer Z, Rast JP, Davidson EH (1999). Origins of immunity: transcription factors and homologues of effector genes of the vertebrate immune system expressed in sea urchin coelomocytes. Immunogenetics.

[B34] Multerer KA, Smith LC (2004). Two cDNAs from the purple sea urchin, *Strongylocentrotus purpuratus*, encoding mosaic proteins with domains found in factor H, factor I, and complement components C6 and C7. Immunogenetics.

[B35] Smith LC, Chang L, Britten RJ, Davidson EH (1996). Sea urchin genes expressed in activated coelomocytes are identified by expressed sequence tags. Complement homologues and other putative immune response genes suggest immune system homology within the deuterostomes. J Immunol.

[B36] Terwilliger DP, Clow LA, Gross PS, Smith LC (2004). Constitutive expression and alternative splicing of the exons encoding SCRs in Sp152, the sea urchin homologue of complement factor B. Implications on the evolution of the Bf/C2 gene family. Immunogenetics.

[B37] Britten RJ, Cetta A, Davidson EH (1978). The single-copy DNA sequence polymorphism of the sea urchin *Strongylocentrotus purpuratus*. Cell.

[B38] Sodergren E, Weinstock GM, Davidson EH, Cameron RA, Gibbs RA, Angerer RC, Angerer LM, Arnone MI, Burgess DR, Burke RD, Coffman JA, Dean M, Elphick MR, Ettensohn CA, Foltz KR, Hamdoun A, Hynes RO, Klein WH, Marzluff W, McClay DR, Morris RL, Mushegian A, Rast JP, Smith LC, Thorndyke MC, Vacquier VD, Wessel GM, Wray G, Zhang L, Elsik CG, Ermolaeva O, Hlavina W, Hofmann G, Kitts P, Landrum MJ, Mackey AJ, Maglott D, Panopoulou G, Poustka AJ, Pruitt K, Sapojnikov V, Song X, Souvorov A, Solovyev V, Wei Z, Whittaker CA, Worley K, Durbin KJ, Shen Y, Fedrigo O, Garfield D, Haygood R, Primus A, Satija R, Severson T, Gonzalez-Garay ML, Jackson AR, Milosavljevic A, Tong M, Killian CE, Livingston BT, Wilt FH, Adams N, Belle R, Carbonneau S, Cheung R, Cormier P, Cosson B, Croce J, Fernandez-Guerra A, Geneviere AM, Goel M, Kelkar H, Morales J, Mulner-Lorillon O, Robertson AJ, Goldstone JV, Cole B, Epel D, Gold B, Hahn ME, Howard-Ashby M, Scally M, Stegeman JJ, Allgood EL, Cool J, Judkins KM, McCafferty SS, Musante AM, Obar RA, Rawson AP, Rossetti BJ, Gibbons IR, Hoffman MP, Leone A, Istrail S, Materna SC, Samanta MP, Stolc V, Tongprasit W, Tu Q, Bergeron KF, Brandhorst BP, Whittle J, Berney K, Bottjer DJ, Calestani C, Peterson K, Chow E, Yuan QA, Elhaik E, Graur D, Reese JT, Bosdet I, Heesun S, Marra MA, Schein J, Anderson MK, Brockton V, Buckley KM, Cohen AH, Fugmann SD, Hibino T, Loza-Coll M, Majeske AJ, Messier C, Nair SV, Pancer Z, Terwilliger DP, Agca C, Arboleda E, Chen N, Churcher AM, Hallbook F, Humphrey GW, Idris MM, Kiyama T, Liang S, Mellott D, Mu X, Murray G, Olinski RP, Raible F, Rowe M, Taylor JS, Tessmar-Raible K, Wang D, Wilson KH, Yaguchi S, Gaasterland T, Galindo BE, Gunaratne HJ, Juliano C, Kinukawa M, Moy GW, Neill AT, Nomura M, Raisch M, Reade A, Roux MM, Song JL, Su YH, Townley IK, Voronina E, Wong JL, Amore G, Branno M, Brown ER, Cavalieri V, Duboc V, Duloquin L, Flytzanis C, Gache C, Lapraz F, Lepage T, Locascio A, Martinez P, Matassi G, Matranga V, Range R, Rizzo F, Rottinger E, Beane W, Bradham C, Byrum C, Glenn T, Hussain S, Manning G, Miranda E, Thomason R, Walton K, Wikramanayke A, Wu SY, Xu R, Brown CT, Chen L, Gray RF, Lee PY, Nam J, Oliveri P, Smith J, Muzny D, Bell S, Chacko J, Cree A, Curry S, Davis C, Dinh H, Dugan-Rocha S, Fowler J, Gill R, Hamilton C, Hernandez J, Hines S, Hume J, Jackson L, Jolivet A, Kovar C, Lee S, Lewis L, Miner G, Morgan M, Nazareth LV, Okwuonu G, Parker D, Pu LL, Thorn R, Wright R (2006). The genome of the sea urchin *Strongylocentrotus purpuratus*. Science.

[B39] Nei M, Gojobori T (1986). Simple methods for estimating the numbers of synonymous and nonsynonymous nucleotide substitutions. Mol Biol Evol.

[B40] Durbin R, Eddy S, Krogh A, Mitchison G (1998). Biological sequence analysis, probability models of proteins and nucleic acids.

[B41] Park IH, Yeon SI, Youn JH, Choi JE, Sasaki N, Choi IH, Shin JS (2004). Expression of a novel secreted splice variant of the receptor for advanced glycation end products (RAGE) in human brain astrocytes and peripheral blood mononuclear cells. Mol Immunol.

[B42] Zhao FQ, Zheng Y, Dong B, Oka T (2004). Cloning, genomic organization, expression, and effect on beta-casein promoter activity of a novel isoform of the mouse Oct-1 transcription factor. Gene.

[B43] Robalino J, Payne C, Parnell P, Shepard E, Grimes AC, Metz A, Prior S, Witteveldt J, Vlak JM, Gross PS, Warr G, Browdy CL (2006). Inactivation of White Spot Syndrome Virus (WSSV) by normal rabbit serum: implications for the role of the envelope protein VP28 in WSSV infection of shrimp. Virus Res.

[B44] Nagawa F, Kishishita N, Shimizu K, Hirose S, Miyoshi M, Nezu J, Nishimura T, Nishizumi H, Takahashi Y, Hashimoto S, Takeuchi M, Miyajima A, Takemori T, Otsuka AJ, Sakano H (2007). Antigen-receptor genes of the agnathan lamprey are assembled by a process involving copy choice. Nat Immunol.

[B45] Yoder JA, Litman RT, Mueller MG, Desai S, Dobrinski KP, Montgomery JS, Buzzeo MP, Ota T, Amemiya CT, Trede NS, Wei S, Djeu JY, Humphray S, Jekosch K, Hernandez Prada JA, Ostrov DA, Litman GW (2004). Resolution of the novel immune-type receptor gene cluster in zebrafish. Proc Natl Acad Sci U S A.

[B46] McDowell JM, Woffenden BJ (2003). Plant disease resistance genes: recent insights and potential applications. Trends Biotechnol.

[B47] Meyers BC, Kaushik S, Nandety RS (2005). Evolving disease resistance genes. Curr Opin Plant Biol.

[B48] Fugmann SD, Messier C, Novack LA, Cameron RA, Rast JP (2006). An ancient evolutionary origin of the Rag1/2 gene locus. Proc Natl Acad Sci U S A.

[B49] Clow LA, Gross PS, Shih CS, Smith LC (2000). Expression of SpC3, the sea urchin complement component, in response to lipopolysaccharide. Immunogenetics.

[B50] Shah M, Brown KM, Smith LC (2003). The gene encoding the sea urchin complement protein, SpC3, is expressed in embryos and can be upregulated by bacteria. Dev Comp Immunol.

[B51] Smith LC, Britten RJ, Davidson EH (1995). Lipopolysaccharide activates the sea urchin immune system. Dev Comp Immunol.

[B52] Hall TA (1999). BioEdit: a user friendly biological sequence alignment editor and analysis program for Windows 95/98/NT.

[B53] Nixon KC (1999). WinClada ver. 1.00.08.

[B54] Kumar S, Tamura K, Nei M (2004). MEGA3: Integrated software for Molecular Evolutionary Genetics Analysis and sequence alignment. Brief Bioinform.

[B55] Yang Z (1997). PAML: a program package for phylogenetic analysis by maximum likelihood. Comput Appl Biosci.

