# Supplementary material for: Distinctive expression patterns of 185/333 genes in the purple sea urchin, Strongylocentrotus purpuratus: an unexpectedly diverse family of transcripts in response to LPS, β-1,3-glucan, and dsRNA
Source: BMC Mol Biol. 2007 Mar 1;8:16. doi: 10.1186/1471-2199-8-16 (PMC1831783; doi:10.1186/1471-2199-8-16)
Supplement: Additional File 1 — Nucleotide alignment for 185/333 cDNAs isolated from individual sea urchins. Alignment of the 185/333 cDNAs showing the sequence diversity, part of the 5' untranslated region, the start codon, indels, gaps, elements, and stop codons. [file 1471-2199-8-16-S1.rtf]

Additional file 1.  Nucleotide alignment for 185/333 cDNAs isolated from individual sea urchins.  An optimal alignment of the cDNAs shows the leader, gaps and 25 elements, which are numbered along the top.  The start codon is outlined in a shaded box.  Element 25 is subdivided into three sub-elements based on nucleotide diversity which alters the location of the stop codon.  The reverse primer used to amplify the cDNAs prior to cloning is located just 3' of the stop codon in 25b.  Therefore, the sequence located 3' of the 25b stop codon is not shown.  Clone numbering is defined as follows: animal number ¯ time of isolation, clone number.  For example, 1-2415 was LPS challenged animal 1 isolated 24h after challenge and clone number 15.  


                   10        20        30        40        50        60        70        80        90       100       110       120       130       140       150       160       170       180       190                           
          ....|....|....|....|....|....|....|....|....|....|....|....|....|....|....|....|....|....|....|....|....|....|....|....|....|....|....|....|....|....|....|....|....|....|....|....|....|....|
1-1515    ----TAGCATCGGAGAGACCTATTACTAACATGGAGGTGAAAGTGACACTGATCGTTGCCATTGTGGCTGCTCTTGCTATCTCGGCTCACGCACAAAGAGATTTCAATGAACGACGAGGAAAGGAGAATGACACAGAGAGAGGACAAGGTGGCTTTGGAGGAAGGCCTGGTGGAATGCAGATGGGTGGTC 186  
1-1523    ----TAGCATCGGAGAGACCTATTACTAACATGGAGGTGAAAGTGACACTGATCGTTGCCATTGTGGCTGCTCTTGCTATCTCGGCTCACGCACAAAGAGATTTCAATGAACGACGAGGAAAGGAGAATGACACAGAGAGAGGACAAGGTGGCTTTGGAGGAAGGCCTGGTGGAATGCAGATGGGTGGTC 186  
1-1504    ----TAGCATCGGAGAGACCTATTACTAACATGGAGGTGAAAGTGACACTGATCGTTGCCATTGTGGCTGCTCTTGCTATCTCGGCTCACGCACAAAGAGATTTCAATGAACGACGAGGAAAGGAGAATGACACAGAGAGAGGACAAGGTGGCTTTGGAGGAAGGCCTGGTGGAATGCAGATGGGTGGTC 186  
1-1533    -----------------------------CATGGAGGTGAAAGTGACACTGATCGTTGCCATTGTGGCTGCTCTTGCTATCTCGGCTCACGCACAAAGAGATTTCAATGAACGACGAGGAAAGGAGAATGACACAGAGAGAGGACAAGGTGGCTTTGGAGGAAGGCCTGGTGGAATGCAGATGGGTGGTC 161  
1-1547    ----TAGCATCGGAGAGACCTATTACTAACATGGAGGTGAAAGTGACACTGATCGTTGCCATTGTGGCTGCTCTTGCTATCTCGGCTCACGCACAAAGAGATTTCAATGAACGACGAGGAAAGGAGAATGACACAGAGAGAGGACAAGGTGGCTTTGGAGGAAGGCCTGGTGGAATGCAGATGGGTGGTC 186  
1-1549    --TATAGCATCGGAGAGACCTATTACTAACATGGAGGTGAAAGTGACACTGATCGTTGCCATTGTGGCTGCTCTTGCTATCTCGGCTCACGCACAAAGAGATTTCAATGAACGACGAGGAAAGGAGAATGACACAGAGAGAGGACAAGGTGGCTTTGGAGGAAGGCCTGGTGGAATGCAGATGGGTGGTC 188  
1-1505    ----TAGCATCGGAGAGACCTATTACTAACATGGAGGTGAAAGTGACACTGATCGTTGCCATTGTGGCTGCTCTTGCTATCTCGGCTCACGCACAAAGAGATTTCAATGAACGACGAGGAAAGGAGAATGACACAGAGAGAGGACAAGGTGGCTTTGGAGGAAGGCCTGGTGGAATGCAGATGGGTGGTC 186  
1-1512    ----TAGCATCGGAGAGACCTATTACTAACATGGAGGTGAAAGTGACACTGATCGTTGCCATTGTGGCTGCTCTTGCTATCTCGGCTCACGCACAAAGAGATTTCAATGAACGACGAGGAAAGGAGAATGACACAGAGAGAGGACAAGGTGGCTTTGGAGGAAGGCCTGGTGGAATGCAGATGGGTGGTC 186  
1-1514    ----TAGCATCGGAGAGACCTATTACTAACATGGAGGTGAAAGTGACACTGATCGTTGCCATTGTGGCTGCTCTTGCTATCTCGGCTCACGCACAAAGAGATTTCAATGAACGACGAGGAAAGGAGAATGACACAGAGAGAGGACAAGGTGGCTTTGGAGGAAGGCCTGGTGGAATGCAGATGGGTGGTC 186  
1-1528    ----TAGCATCGGAGAGACCTATTACTAACATGGAGGTGAAAGTGACACTGATCGTTGCCATTGTGGCTGCTCTTGCTATCTCGGCTCACGCACAAAGAGATTTCAATGAACGACGAGGAAAGGAGAATGACACAGAGAGAGGACAAGGTGGCTTTGGAGGAAGGCCTGGTGGAATGCAGATGGGTGGTC 186  
1-1532    ----TAGCATCGGAGAGACCTATTACTAACATGGAGGTGAAAGTGACACTGATCGTTGCCATTGTGGCTGCTCTTGCTATCTCGGCTCACGCACAAAGAGATTTCAATGAACGACGAGGAAAGGAGAATGACACAGAGAGAGGACAAGGTGGCTTTGGAGGAAGGCCTGGTGGAATGCAGATGGGTGGTC 186  
1-1539    ----TAGCATCGGAGAGACCTATTACTAACATGGAGGTGAAAGTGACACTGATCGTTGCCATTGTGGCTGCTCTTGCTATCTCGGCTCACGCACAAAGAGATTTCAATGAACGACGAGGAAAGGAGAATGACACAGAGAGAGGACAAGGTGGCTTTGGAGGAAGGCCTGGTGGAATGCAGATGGGTGGTC 186  
1-1536    -----AGCATCGGAGAGACCTATTACTAACATGGAGGTGAAAGTGACACTGATCGTTGCCATTGTGGCTGCTCTTGCTATCTCGGCTCACGCACAAAGAGATTTCAATGAACGACGAGGAAAGGAGAATGACACAGAGAGAGGACAAGGTGGCTTTGGAGGAAGGCCTGGTGGAATGCAGATGGGTGGTC 185  
1-1535    ----TAGCATCGGAGAGACCTATTACTAACATGGAGGTGAAAGTGACACTGATCGTTGCCATTGTGGCTGCTCTTGCTATCTCGGCTCACGCACAAAGAGATTTCAATGAACGACGAGGAAAGGAGAATGACACAGAGAGAGGACAAGGTGGCTTTGGAGGAAGGCCTGGTGGAATGCAGATGGGTGGTC 186  
1-1534    ----TAGCATCGGAGAGACCTATTACTAACATGGAGGTGAAAGTGACACTGATCGTTGCCATTGTGGCTGCTCTTGCTATCTCGGCTCACGCACAAAGAGATTTCAATGAACGACGAGGAAAGGAGAATGACACAGAGAGAGGACAAGGTGGCTTTGGAGGAAGGCCTGGTGGAATGCAGATGGGTGGTC 186  
1-2402    ----TAGCATCGGAGAGACCTATCACTAACATGGAGGTGAAAGTGACACTGATCGTTGCCATTGTGGCTGCTCTTGCTATCTCGGCTCACGCACAAAGAGATTTCAATGAACGACGAGGAAAGGAGAATGACACAGAGAGAGGACAAGGTGGCTTTGGAGGAAGGCCTGGTGGAATACAGATGGGTGGTC 186  
1-2404    ------TAGCATCGGAGAGACCTTACTAACATGGAGGTGAAAGTGACACTGATCGTTGCCATTGTGGCTGCTCTTGCTATCTCGGCTCACGCACAAAGAGATTTCAATGAACGACGAGGAAAGGAGAATGACACAGAGAGAGGACAAGGTCGCTTTGGAGGAAGGCCTGGTGGAATGCAGATGGGTGGAC 184  
1-2405    ----TAGCATCGGAGAGACCTATTACTAACATGGAGGTGAAAGTGACACTGATCGTTGCCATTGTGGCTGCTCTTGCTATCTCGGCTCACGCACAAAGAGATTTCAATGAACGACGAGGAAAGGAGAATGACACAGAGAGAGGACAAGGTGGCTTTGGAGGAAGGCCTGGTGGAATGCAGATGGGTGGTC 186  
1-2406    ----TAGCATCGGAGAGACCTATTACTAACATGGAGGTGAAAGTGACACTGATCGTTGCCATTGTGGCTGCTCTTGCTATCTCGGCTCACGCACAAAGAGATTTCAATGAACGACGAGGAAAGGAGAATGACACAGAGAGAGGACAAGGTGGCTTTGGAGGAAGGCCTGGTGGAATGCAGATGGGTGGTC 186  
1-2407    ------TAGCATCGGAGAGACCTTACTAACATGGAGGTGAAAGTGACACTGATCGTTGCCATTGTGGCTGCTCTTGCTATCTCGGCTCACGCACAAAGAGATTTCAATGAACGACGAGGAAAGGAGAATGACACAGAGAGAGGACAAGGTCGCTTTGGAGGAAGGCCTGGTGGAATGCAGATGGGTGGAC 184  
1-2412    ----TAGCATCGGAGAGACCTATTACTAACATGGAGGTGAAAGTGACACTGATCGTTGCCATTGTGGCTGCTCTTGCTATCTCGGCTCACGCACAAAGAGATTTCAATGAACGACGAGGAAAGGAGAATGACACAGAGAGAGGACAAGGTGGCTTTGGAGGAAGGCCTGGTGGAATGCAGATGGGTGGTC 186  
1-2413    ----TAGCATCGGAGAGACCTATTACTAACATGGAGGTGAAAGTGACACTGATCGTTGCCATTGTGGCTGCTCTTGCTATCTCGGCTCACGCACAAAGAGATTTCAATGAACGACGAGGAAAGGAGAATGACACAGAGAGAGGACAAGGTGGCTTTGGAGGAAGGCCTGGTGGAATGCAGATGGGTGGTC 186  
1-2416    ------TAGCATCGGAGAGACCTTACTAACATGGAGGTGAAAGTGACACTGATCGTTGCCATTGTGGCTGCTCTTGCTATCTCGGCTCACGCACAAAGAGATTTCAATGAACGACGAGGAAAGGAGAATGACACAGAGAGAGGACAAGGTCGCTTTGGAGGAAGGCCTGGTGGAATGCAGATGGGTGGAC 184  
1-2417    ------TAGCATCGGAGAGACCTTACTAACATGGAGGTGAAAGTGACACTGATCGTTGCCATTGTGGCTGCTCTTGCTATCTCGGCTCACGCACAAAGAGATTTCAATGAACGACGAGGAAAGGAGAATGACACAGAGAGAGGACAAGGTCGCTTTGGAGGAAGGCCTGGTGGAATGCAGATGGGTGGAC 184  
1-2418    ----TAGCATCGGAGAGACCTATTACTAACATGGAGGTGAAAGTGACACTGATCGTTGCCATTGTGGCTGCTCTTGCTATCTCGGCTCACGCACAAAGAGATTTCAATGAACGACGAGGAAAGGAGAATGACACAGAGAGAGGACAAGGTGGCTTTGGAGGAAGGCCTGGTGGAATGCAGATGGGTGGTC 186  
1-2420    ------TAGCATCGGAGAGACCTTACTAACATGGAGGTGAAAGTGACACTGATCGTTGCCATTGTGGCTGCTCTTGCTATCTCGGCTCACGCACAAAGAGATTTCAATGAACGACGAGGAAAGGAGAATGACACAGAGAGAGGACAAGGTCGCTTTGGAGGAAGGCCTGGTGGAATGCAGATGGGTGGAC 184  
1-2421    ------TAGCATCGGAGAGACCTTACTAACATGGAGGTGAAAGTGACACTGATCGTTGCCATTGTGGCTGCTCTTGCTATCTCGGCTCACGCACAAAGAGATTTCAATGAACGACGAGGAAAGGAGAATGACACAGAGAGAGGACAAGGTCGCTTTGGAGGAAGGCCTGGTGGAATGCAGATGGGTGGAC 184  
1-2422    ----TAGCATCGGAGAGACCTATTACTAACATGGAGGTGAAAGTGACACTGATCGTTGCCATTGTGGCTGCTCTTGCTATCTCGGCTCACGCACAAAGAGATTTCAATGAACGACGAGGAAAGGAGAATGACACAGAGAGAGGACAAGGTGGCTTTGGAGGAAGGCCTGGTGGAATGCAGATGGGTGGTC 186  
1-2425    ----TAGCATCGGAGAGACCTATTACTAACATGGAGGTGAAAGTGACACTGATCGTTGCCATTGTGGCTGCTCTTGCTATCTCGGCTCACGCACAAAGAGATTTCAATGAACGACGAGGAAAGGAGAATGACACAGAGAGAGGACAAGGTGGCTTTGGAGGAAGGCCTGGTGGAATGCAGATGGGTGGTC 186  
1-2426    ----TAGCATCGGAGAGACCTATTACTAACATGGAGGTGAAAGTGACACTGATCGTTGCCATTGTGGCTGCTCTTGCTATCTCGGCTCACGCACAAAGAGATTTCAATGAACGACGAGGAAAGGAGAATGACACAGAGAGAGGACAAGGTGGCTTTGGAGGAAGGCCTGGTGGAATGCAGATGGGTGGTC 186  
1-2427    ----TAGCATCGGAGAGACCTATTACTAACATGGAGGTGAAAGTGACACTGATCGTTGCCATTGTGGCTGCTCTTGCTATCTCGGCTCACGCACAAAGAGATTTCAATGAACGACGAGGAAAGGAGAATGACACAGAGAGAGGACAAGGTGGCTTTGGAGGAAGGCCTGGTGGAATGCAGATGGGTGGTC 186  
1-2428    ----TAGCATCGGAGAGACCTATTACTAACATGGAGGTGAAAGTGACACTGATCGTTGCCATTGTGGCTGCTCTTGCTATCTCGGCTCACGCACAAAGAGATTTCAATGAACGACGAGGAAAGGAGAATGACACAGAGAGAGGACAAGGTGGCTTTGGAGGAAGGCCTGGTGGAATGCAGATGGGTGGTC 186  
1-2430    ----TAGCATCGGAGAGACCTATTACTAACATGGAGGTGAAAGTGACACTGATCGTTGCCATTGTGGCTGCTCTTGCTATCTCGGCTCACGCACAAAGAGATTTCAATGAACGACGAGGAAAGGAGAATGACACAGAGAGAGGACAAGGTGGCTTTGGAGGAAGGCCTGGTGGAATGCAGATGGGTGGTC 186  
1-2431    ------TAGCATCGGAGAGACCTTACTAACATGGAGGTGAAAGTGACACTGATCGTTGCCATTGTGGCTGCTCTTGCTATCTCGGCTCACGCACAAAGAGATTTCAATGAACGACGAGGAAAGGAGAATGACACAGAGAGAGGACAAGGTCGCTTTGGAGGAAGGCCTGGTGGAATGCAGATGGGTGGAC 184  
1-2432    ----TAGCATCGGAGAGACCTATTACTAACATGGAGGTGAAAGTGACACTGATCGTTGCCATTGTGGCTGCTCTTGCTATCTCGGCTCACGCACAAAGAGATTTCAATGAACGACGAGGAAAGGAGAATGACACAGAGAGAGGACAAGGTGGCTTTGGAGGAAGGCCTGGTGGAATGCAGATGGGTGGTC 186  
1-2433    ----TAGCATCGGAGAGACCTATTACTAACATGGAGGTGAAAGTGACACTGATCGTTGCCATTGTGGCTGCTCTTGCTATCTCGGCTCACGCACAAAGAGATTTCAATGAACGACGAGGAAAGGAGAATGACACAGAGAGAGGACAAGGTGGCTTTGGAGGAAGGCCTGGTGGAATGCAGATGGGTGGTC 186  
1-2434    ------TAGCATCGGAGAGACCTTACTAACATGGAGGTGAAAGTGACACTGATCGTTGCCATTGTGGCTGCTCTTGCTATCTCGGCTCACGCACAAAGAGATTTCAATGAACGACGAGGAAAGGAGAATGACACAGAGAGAGGACAAGGTCGCTTTGGAGGAAGGCCTGGTGGAATGCAGATGGGTGGAC 184  
1-2435    ------TAGCATCGGAGAGACCTTACTAACATGGAGGTGAAAGTGACACTGATCGTTGCCATTGTGGCTGCTCTTGCTATCTCGGCTCACGCACAAAGAGATTTCAATGAACGACGAGGAAAGGAGAATGACACAGAGAGAGGACAAGGTCGCTTTGGAGGAAGGCCTGGTGGAATGCAGATGGGTGGAC 184  
1-2436    ------TAGCATCGGAGAGACCTTACTAACATGGAGGTGAAAGTGACACTGATCGTTGCCATTGTGGCTGCTCTTGCTATCTCGGCTCACGCACAAAGAGATCTCAATGAACGACGAGGAAAGGAGAATGACACAGAGAGAGGACAAGGTGGCTTTGGAGGAAGGCCTGGTGGAATGCAGATGGGTGGTC 184  
1-2437    ----TAGCATCGGAGAGACCTATTACTAACATGGAGGTGAAAGTGACACTGATCGTTGCCATTGTGGCTGCTCTTGCTATCTCGGCTCACGCACAAAGAGATTTCAATGAACGACGAGGAAAGGAGAATGACACAGAGAGAGGACAAGGTGGCTTTGGAGGAAGGCCTGGTGGAATGCAGATGGGTGGTC 186  
1-2439    ------TAGCATCGGAGAGACCTTACTAACATGGAGGTGAAAGTGACACTGATCGTTGCCATTGTGGCTGCTCTTGCTATCTCGGCTCACGCACAAAGAGATTTCAATGAACGACGAGGAAAGGAGAATGACACAGAGAGAGGACAAGGTCGCTTTGGAGGAAGGCCTGGTGGAATGCAGATGGGTGGAC 184  
1-2440    ------TAGCATCGGAGAGACCTTACTAACATGGAGGTGAAAGTGACACTGATCGTTGCCATTGTGGCTGCTCTTGCTATCTCGGCTCACGCACAAAGAGATTTCAATGAACGACGAGGAAAGGAGAATGACACAGAGAGAGGACAAGGTCGCTTTGGAGGAAGGCCTGGTGGAATGCAGATGGGTGGAC 184  
1-2441    ----TAGCATCGGAGAGACCTATCACTAACATGGAGGTGAAAGTGACACTGATCGTTGCCATTGTGGCTGCTCTTGCTATCTCGGCTCACGCACAAAGAGATTTCAATGAACGACGAGGAAAGGAGAATGACACAGAGAGAGGACAAGGTGGCTTTGGAGGAAGGCCTGGTGGAATGCAGATGGGTGGTC 186  
1-2442    ------TAGCATCGGAGAGACCTTACTAACATGGAGGTGAAAGTGACACTGATCGTTGCCATTGTGGCTGCTCTTGCTATCTCGGCTCACGCACAAAGAGATTTCAATGAACGACGAGGAAAGGAGAATGACACAGAGAGAGGACAAGGTCGCTTTGGAGGAAGGCCTGGTGGAATGCAGATGGGTGGAC 184  
1-2414    ------TAGCATCGGAGAGACCTTACAAACATG---GTGAAAGTGACACTGATCATTGCCATTGTGGCTGCTCTTGCTATCTCAGCTCACGCACAAAGAGATTACAATGAACTACGAGGAAATAAGAATGGCAGAGAGAGAGGACAAGGTCGCTTTGGAGGAAGGCCGGGTGGAATGCAGATGGGTGGAT 181  
1-2424    -----TAGCATCTGGATAGACCTTACTAACATGGAGGTGAAAGTGACACTGATCGTTGCCATTGTGGCTGCTCTTGCTATCTCGGCTCACGCACAAAGAGATTTCAATGAACGACGAGGAAAGGAGAATGACACAGAGAGAGGACAAGGTCGCTTTGGAGGAAGGCCTGGTGGAATGCAGACAAAATGGC 185  
1-2429    ------TAGCATCGGAGAGACCTTACTAACATGGAGGTGAAAGTGACACTGATCGTTGCCATTGTGGCTGCTCTTGCTATCTCGGCTCACGCACAAAGAGATTTCAATGAACGACGAGGAAAGGAGAATGACACAGAGAGAGGACAAGGTCGCTTTGGAGGAAGGCCTGGTGGAATGCAGACAAAATGGC 184  
2-1501    ------TAGACTCGGAGAGACCTTACTAACATGGAGGTGAAAGTGACACTGATCGTTGCCATTGTGGCTGCTCTTGCTATCTCGGCTCACACACAAAGAGATTACAATGAACGACGAGGAAATGAGAATGGCAGAGAGAGAGGACAAGGTCGCTTTGGAGGAAGGCCTGGTGGAATGCAGATGGGTGGAC 184  
2-1502    ------TAGCATCGGAGAGACCTTACTAACATGGAGGTGAAAGTGACACTGATCGTTGCCATTGTGGCTGCTCTTGCTATCTCGGCTCACACACAAAGAGATTACAATGAACGACGAGGAAATGAGAATGGCAGAGAGAGAGGACAAGGTCGCTTTGGAGGAAGGCCTGGTGGAATGCAGATGGGTGGAC 184  
2-1505    ------TAGCATCGGAGAGACCTTACTAACATGGAGGTGAAAGTGACACTGATCGTTGCCATTGTGGCTGCTCTTGCTATCTCGGCTCACACACAAAGAGATTACAATGAACGACGAGGAAATGAGAATGGCAGAGAGAGAGGACAAGGTCGCTTTGGAGGAAGGCCTGGTGGAATGCAGATGGGTGGAC 184  
2-1506    ------TAGCATCGGAGAGACCTTACTAACATGGAGGTGAAAGTGACACTGATCGTTGCCATTGTGGCTGCTCTTGCTATCTCGGCTCACACACAAAGAGATTACAATGAACGACGAGGAAATGAGAATGGCAGAGAGAGAGGACAAGGTCGCTTTGGAGGAAGGCCTGGTGGAATGCAGATGGGTGGAC 184  
2-1507    -----TAGACATCGGAGAGACCTTACTAACATGGAGGTGAAAGTGACACTGATCGTTGCCATTGTGGCTGCTCTTGCTATCTCGGCTCACACACAAAGAGATTACAATGAACGACGAGGAAATGAGAATGGCAGAGAGAGAGGACAAGGTCGCTTTGGAGGAAGGCCTGGTGGAATGCAGATGGGTGGAC 185  
2-1508    ------TAGCATCGGAGAGACCTTACTAACATGGAGGTGAAAGTGACACTGATCGTTGCCATTGTGGCTGCTCTTGCTATCTCGGCTCACACACAAAGAGATTACAATGAACGACGAGGAAATGAGAATGGCAGAGAGAGAGGACAAGGTCGCTTTGGAGGAAGGCCTGGTGGAATGCAGATGGGTGGAC 184  
2-1509    ------TAGCATCGGAGAGACCTTACTAACATGGAGGTGAAAGTGACACTGATCGTTGCCATTGTGGCTGCTCTTGCTATCTCGGCTCACACACAAAGAGATTACAATGAACGACGAGGAAATGAGAATGGCAGAGAGAGAGGACAAGGTCGCTTTGGAGGAAGGCCTGGTGGAATGCAGATGGGTGGAC 184  
2-1510    --------------GAGAGATCCATCAAGCATGGAGGTGAAAGTGACACTGATCGTTGCCATTGTGGCTGCTCTTGCTATCTCGGCTCACACACAAAGAGATTACAATGAACGACGAGGAAATGAGAATGGCAGAGAGAGAGGACAAGGTCGCTTTGGAGGAAGGCCTGGTGGAATGCAGATGGGTGGAC 176  
2-1514    ------TAGCATCGGAGAGACCTTACTAACATGGAGGTGAAAGTGACACTGATCGTTGCCATTGTGGCTGCTCTTGCTATCTCGGCTCACACACAAAGAGATTACAATGAACGACGAGGAAATGAGAATGGCAGAGAGAGAGGACAAGGTCGCTTTGGAGGAAGGCCTGGTGGAATGCAGATGGGTGGAC 184  
2-1511    ------TAGACTCGGAGAGACCTTACTAGCATGGGGGTGAAAGTGACACTGATCGTTGCCATTGTGGCTGCTCTTGCTATCTCGGCTCACACACAAAGAGATTACAATGAACGACGAGGAAATGAGAATGGCAGAGAGAGAGGACAAGGTCGCTTTGGAGGAAGGCCTGGTGGAATGCAGATGGGTGGAC 184  
2-2401    ----TAGCATCGGAGAGACCTATTACTATCATG---GTGAAAGTGACACTGATCGTTGCCATTGTGGCTGCTCTTGCTATCTCAGCTCACGCACAAAGAGATTTCAATGAACGACGAGGAAAGGAGAATGACACAGAAAGAGGACAAGGTGGCTTTGGAGGAAGGCCTGGTGGAATGCAGATGGGTGGTC 183  
2-2404    ----TAGCATCGGAGAGACCTATTACTATCATG---GTGAAAGTGACACTGATCGTTGCCATTGTGGCTGCTCTTGCTATCTCAGCTCACGCACAAAGAGATTTCAATGAACGACGAGGAAAGGAGAATGACACAGAGAGAGGACAAGGTGGCTTTGGAGGAAGGCCTGGTGGAATGCAGATGGGTGGTC 183  
2-2406    ----TAGCATCGGAGAGACCTATTACTATCATG---GTGAAAGTGACACTGATCGTTGCCATTGTGGCTGCTCTTGCTATCTCGGCTCACGCAGAAAGAGATTTCAATGAACGACGAGGAAAGGAGAATGGCAGAGAGAGAGGACAAGGTGGCTTTGGAGGAAGGCCTGGTGGAATGCAGACGGGTAGTC 183  
2-2407    ----TAGCATCGGAGAGACCTATTACTATCATG---GTGAAAGTGACACTGATCGTTGCCTTTGTGGCTGCTCTTGCTATCTCAGCTCACGCACAAAGAGATTTCAATGAACGACGAGGAAAGGAGAATGACACAGAGAGAGGACAAGGTGGCTTTGGAGGAAGGCCTGGTGGAATGCAGATGGGTGGTC 183  
2-2408    ----TAGCATCGGAGAGACCTATTACTATCATG---GTGAAAGTGACACTGATCGTTGCCATTGTGGCTGCTCTTGCTATCTCAGCTCACGCACAAAGAGATTTCAATGAACGACGAGGAAAGGAGAATGACACAGAGAGAGGACAAGGTGGCTTTGGAGGAAGGCCTGGTGGAATGCAGATGGGTGGTC 183  
2-2409    ----TAGCATCGGAGAGACCTATTACTATCATG---GTGAAAGTGACACTGATCGTTGCCATTGTGGCTGCTCTTGCTATCTCAGCTCACGCACAAAGAGATTTCAATGAACGACGAGGAAAGGAGAATGACACAGAGAGAGGACAAGGTGGCTTTGGAGGAAGGCCTGGTGGAATGCAGATGGGTGGTC 183  
2-2411    ---TAGACTCGGATGAGACCTATTACTATCATG---GTGAAAGTGACACTGATCGTTGCCATTGTGGCTGCTCTTGCTATCTCAGCTCACGCACAAAGAGATTTCAATGAACGACGAGGAAAGGAGAATGACACAGAGAGAGGACAAGGTGGCTTTGGAGGAAGGCCTGGTGGAATGCAGATGGGTGGTC 184  
2-2405    ------TAGCATCGGAGAGACCTTACAAACATGGAGGTGAAAGCAACATTGATCGTTGCCATTGTGGCTGCTCTTGCTATCTCGGCTCACGCACGAAGAGATTTCAATGAACGGCGAGGAAATGAGAATGGCAGAGAGAGAGGACAAGGTCGCTTTGGAGCAAGGCCTGGTGGAATGCAGATGGGTGGAT 184  
2-2413    ------TAGACTGCGGAGAGACCTTACAACATGGAGGTGAAAGCAACATTGATCGTTGCCATTGTGGCTGCTCTTGCTATCTCGGCTCACGCACGAAGAGATTTCAATGAACGGCGAGGAAATGAGAATGGCAGAGAGAGAGGACAAGGTCGCTTTGGAGCAAGGCCTGGTGGAATGCAGATGGGTGGAT 184  
2-2403    ----TAGCATCGGAGAGACCTATTACTAACATGGAGGTGAAAGTGACACTGATCGTTGCCATTGTGGCTGCTCTTGCTATCTCGGCTCACGCACGAAGAGATTTCAATGAACGACGAGGAAAGGAGAATGGCAGAGAGAGAGGACAAGGTGGCTTTGGAGGAAGGCCTGGTGGAATGCAGACGGGTAGTC 186  
2-2415    ----TAGCATCGGAGAGACCTATTACTAACATG---GTGAAAGTGACACTGATCGTTGCCATTGTGGCTGCTCTTGCTATCTCAGCTCACGCACAAAGAGATTACAATGAACTACGAGGAAATAAGAATGGCAGAGAGAGAGGACAAGGTCGCTTTGGAGGAAGGCCGGGTGGAATGCAGATGGGTGGAT 183  
2-2414    ---TAGACATCGGAGAGACCTATTACTAACATG---GTGAAAGTGACACTGATCGTTGCCATTGTGGCTGCTCTTGCTATCTCAGCTCACGCACAAAGAGATTACAATGAACTACGAGGAAATAAGAATGGCAGAGAG--AGGACAAGGTCGCTTTGGAGGAAGGCCGGGTGGAATGCAGATGGGTGGAT 182  
3-15-1006 -----TAGCATCTTGAGAGACCTTACTAACATGGAGGTGAAAGTGACACTGATCGTTGCCATTGTGGCTGCTCTTGCTATCTCGGCTCACACACAAAGAGATTACAATGAACGACGAGGAAATGAGAATGGCAGAGAGAGAGGACAAGGTCGCTTTGGAGGAAGGCCTGGTGGAATGCAGATGGGTGGAC 185  
3-15-4003 ------AGCATCGTGAGAGACCTTACTAACATGGAGGTGAAAGTGACACTGATCGTTGCCATTGTGGCTGCTCTTGCTATCTCGGCTCACACACAAAGAGATTACAATGAACGACGAGGAAATGAGAATGGCAGAGAGAGAGGACAAGGTCGCTTTGGAGGAAGGCCTGGTGGAATGCAGATGGGTGGAC 184  
3-15-4004 ------AGCATCGTGAGAGACCTTACTAACATGGAGGTGAAAGTGACACTGATCGTTGCCATTGTGGCTGCTCTTGCTATCTCGGCTCACACACAAAGAGATTACAATGAACGACGAGGAAATGAGAATGGCAGAGAGAGAGGACAAGGTCGCTTTGGAGGAAGGCCTGGTGGAATGCAGATGGGTGGAC 184  
3-15-4005 -----AGCATCGTGAGAGACCTCTACTAACATGGAGGTGAAAGTGACACTGATCGTTGCCATCGTGGCTGCTCTTGCTATCTCGGCTCACACACAAAGAGATTACAATGAACGACGAGGAAATGAGAATGGCAGAGAGAGAGGACAAGGTCGCTTTGGAGGAAGGCCTGGTGGAATGCAGATGGGTGGAC 185  
3-15-4007 -------AGCTCGGAGAGACCTTCACTAACATGGAGGTGAAAGTGACACTGATCGTTGCCATTGTGGCTGCTCTTGCTATCTCGGCTCACACACAAAGAGATTACAATGAACGACGAGGAAATGAGAATGGCAGAGAGAGAGGGCAAGGTCGCTTTGGAGGAAGGCCTGGTGGAATGCAGATGGGTGGAC 183  
3-15-4011 ------AGCATCGTGAGAGACCTTACTAACATGGAGGTGAAAGTGACACTGATCGTTGCCATTGTGGCTGCTCTTGCTATCTCGGCTCACACACAAAGAGATTACAATGAACGACGAGGAAATGAGAATGGCAGAGAGAGAGGACAAGGTCGCTTTGGAGGAAGGCCTGGTGGAATGCAGATGGGTGGAC 184  
3-15-4013 -----TAGCATCTTGAGAGACCTTACTAACATGGAGGTGAAAGTGACACTGATCGTTGCCATCGTGGCTGCTCTTGCTATCTCGGCTCACACACAAAGAGATTACAATGAACGACGAGGAAATGAGAATGGCAGAGAGAGAGGACAAGGTCGCTTTGGAGGAAGGCCTGGTGGAATGCAGATGGGTGGAC 185  
3-15-4015 ------TAGACTCGGAGAGACCTTACTAACATGGAGGTGAAAGTGACACTGATCGTTGCCATTGTGGCTGCTCTTGCTATCTCGGCTCACACACAAAGAGATTACAATGAACGACGAGGAAATGAGAACGGCAGAGAGAGAGGACAAGGTCGCTTTGGAGGAAGGCCTGGTGGAATGCAGATGGGTGGAC 184  
3-15-4018 ------TAGCTCGGAGAGACCTTCACTAACATGGAGGTGAAAGTGACACTGATCGTTGCCATTGTGGCTGCTCTTGCTATCTCGGCTCACACACAGAGAGATTACAATGAACGACGAGGAAATGAGAATGGCAGAGAGAGAGGACAAGGTCGCTTTGGAGGAAGGCCTGGTGGAATGCAGATGGGTGGAC 184  
3-15-4019 ------TAGCATCGGAGAGACCTTACTAACATGGAGGTGAAAGTGACACTGATCGTTGCCATTGTGGCTGCTCTTGCTATCTCGGCTCACACACAAAGAGATTACAATGAACGACGAGGAAATGAGAATGGCAGAGAGAGAGGACAAGGTCGCTTTGGAGGAAGGCCTGGTGGAATGCAGATGGGTGGAC 184  
3-15-4022 -----TAGACATCGGAGAGACCTTACTAACATGGAGGTGAAAGTGACACTGATCGTTGCCATTGTGGCTGCTCTTGCTATCTCGGCTCACACACAAAGAGATTACAATGAACGACGAGGAAATGAGAATGGCAGAGAGAGAGGACAAGGTCGCTTTGGAGGAAGGCCTGGTGGAATGCAGATGGGTGGAC 185  
3-15-4024 ------TAGACTCGGAGAGACCTTACTAACATGGAGGTGAAAGTGACACTGATCGTTGCCATTGTGGCTGCTCTTGCTATCTCGGCTCACACACAAAGAGATTACAATGAACGACGAGGAAATGAGAATGGCAGAGAGAGAGGACAAGGTCGCTTTGGAGGAAGGCCTGGTGGAATGCAGATGGGTGGAC 184  
3-15-4017 -----TAGACATCGGAGAGACCTTACTAACATGGAGGTGAAAGTGACACTGATCGTTGCCATTGTGGCTGCTCTTGCTATCTCGGCTCACACACAAAGAGATTACAATGAACGACGAGGAAATGAGAATGGCAGAGAGAGAGGACAAGGTCGCTTTGGAGGAAGGCCTGGTGGAATGCAGATGGGTGGAC 185  
3-15-1003 ------TAGCATCGGAGAGACCTTACTAACATGGAGGTGAAAGTGACACTGATCGTTGCCATTGTGGCTGCTCTTGCTATCTCGGCTCACACACAAAGAGATTACAATGAACGACGAGGAAATGAGAATGGCAGAGAGAGAGGACAAGGTCGCTTTGGAGGAAGGCCTGGTGGAATGCAGATGGGTGGAC 184  
3-15-4021 ----TAGCATCGGAGAGACCTATTACTATCATG---GTGAAAGTGACACTGATCGTTGCCATTGTGGCTGCTCTTGCTATCTCGGCTCACGCAGAAAGAGATTTCAATGAACTACGAGGAAAGGAGAATGGCAGAGAGAGAGGACAAGGTGGCTTTGGAGGAAGGCCTGGTGGAATGCAGACGGGTAGTC 183  
3-15-1001 ----TAGCATCGGAGAGACCTATTACTATCATG---GTGAAAGTGACACTGATCGTTGCCATTGTGGCTGCTCTTGCTATCTCGGCTCACGCAGAAAGAGATTTCAATGAACGACGAGGAAAGGAGAATGGCAGAGAGAGAGGACAAGGTGGCTTTGGAGGAAGGCCTGGTGGAATGCAGACGGGTAGTC 183  
3-15-1002 ----TAGCATCGGAGAGACCTATTACTATCATG---GTGAAAGTGACACTGATCGTTGCCATTGTGGCTGCTCTTGCTATCTCGGCTCACGCAGAAAGAGATTTCAATGAACGACGAGGAAAGGAGAATGGCAGAGAGAGAGGACAAGGTGGCTTTGGAGGAAGGCCTGGTGGAATGCAGACGGGTAGTC 183  
3-15-4008 ------TAGCATCGGAGAGACCTTACTAACATGGAGGTGAAAGTGACACTGATCGTTGCCATTGTGGCTGCTCTTGCTATCTCGGCTCACACACAAAGAGATTACAATGAACGACGAGGAAATGAGAATGGCAGAGAGAGGACAAGGTCGCTTTGGAGGAAGGCCTGGTGGAATGCAGATGGGTGGACCA 184  
3-24-4003 ------TAGCATCGGAGAGACCTTACAAACATGGAGGTGAAAGCAACACTGATCGTTGCCATTCTGGCTGTTCTTGCTATCTCGGCTCACGCACAAAGAGATTTCAATGAACGACGAGGAAAGGAGAATGACACAGAGAGAGGACAAGGTGGCTTTGGAGGAAGGCCTGGTGGAATGCAGATGGGTGGTC 184  
3-24-4004 ------TAGCATCGGAGAGACCTTACTAACATGGAGGTGAAAGTGACACTGATCGTTGCCATTGTGGCTGCTCTTGCTATCTCGGCTCACGCACAAAGAGATTTCAATGAACGACGAGGAAAGGAGAATGACACAGAGAGAGGACAAGGTGGCTTTGGAGGAAGGCCTGGTGGAATGCAGATGGGTGGTC 184  
3-24-1006 ------TAGCATCGGAGAGACCTTACAAACATGGAGGTGAAAGCAACACTGATCGTTGCCATTCTGGCTGTTCTTGCTATCTCGGCTCACGCACAAAGAGATTTCAATGAACGACGAGGAAAGGAGAATGACACAGAGAGAGGACAAGGTGGCTTTGGAGGAAGGCCTGGTGGAATGCAGATGGGTGGTC 184  
3-24-4006 ------TAGCATCGGAGAGACCTTACTAACATGGAGGTGAAAGTGACACTGATCGTTGCCATTGTGGCTGCTCTTGCTATCTCGGCTCACGCACAAAGAGATTTCAATGAACGACGAGGAAAGGAGAATGACACAGAGAGAGGACAAGGTGGCTTTGGAGGAAGGCCTGGTGGAATGCAGATGGGTGGTC 184  
3-24-4015 ------TAGCATCGGAGAGACCTTACAAACATGGAGGTGAAAGCAACACTGATCGTTGCCATTCTGGCTGTTCTTGCTATCTCGGCTCACGCACAAAGAGATTTCAATGAACGACGAGGAAAGGAGAATGACACAGAGAGAGGACAAGGTGGCTTTGGAGGAAGGCCTGGTGGAATGCAGATGGGTGGTC 184  
3-24-4021 ------TAGCATCGGAGAGACCTTACTAACATGGAGGTGAAAGTGACACTGATCGTTGCCATTGTGGCTGCTCTTGCTATCTCGGCTCACGCACAAAGGGATTTCAATGAACGACGAGGAAAGGAGAATGACACAGAGAGAGGACAAGGTGGCTTTGGAGGAAGGCCTGGTGGAATGCAGATGGGTGGTC 184  
3-24-4024 -------TAGCTCGGAGAGACCTTACTAACATGGAGGTGAAAGTGACACTGATCGTTGCCATTGTGGCTGCTCTTGCTATCTCGGCTCACGCACAAAGAGATTTCAATGAACGACGAGGAAAGGAGAATGACACAGAGAGAGGACAAGGTGGCTTTGGAGGAAGGCCTGGTGGAATGCAGATGGGTGGTC 183  
3-24-4023 ------TAGCATCGGAGAGACCTTACAAACATGGAGGTGAAAGCAACACTGATCGTTGCCATTCTGGCTGTTCTTGCTATCTCGGCTCACGCACAAAGAGATTTCAATGAACGACGAGGAAAGGAGAATGACACAGAGAGAGGACAAGGTGGCTTTGGAGGAAGGCCTGGTGGAATGCAGATGGGTGGTC 184  
3-24-4001 ----TAGCATCGGAGAGACCTATTACTATCATG---GTGAAAGTGACACTGATCGTTGCCATTGTGGCTGCTCTTGCTATCTCGGCTCACGCACGAAGAGATTTCAATGAACGACGAGGAAAGGAGAATGGCACAGAGAGAGGACAAGGTGGCTTTGGAGGAAGGCCTGGTGGAATGCAGACGGGTAGTC 183  
3-24-4019 ----TAGCATCGGAGAGACCTATTACTATCATG---GTGAAAGTGACACTGATCGTTGCCATTGTGGCTGCTCTTGCTATCTCGGCTCACGCACGAAGAGATTTCAATGAACGACGAGGAAAGGAGAATGGCACAGAGAGAGGACAAGGTGGCTTTGGAGGAAGGCCTGGTGGAATGCAGACGGGTAGTC 183  
3-24-1003 ----TAGCATCGGAGAGACCTATTACTATCATG---GTGAAAGTGACACTGATCGTTGCCATTGTGGCTGCTCTTGCTATCTCGGCTCACGCACGAAGAGATTTCAATGAACGACGAGGAAAGGAGAATGGCAGAGAGAGAGGACAAGGTGGCTTTGGAGGAAGGCCTGGTGGAATGCAGACGGGTAGTC 183  
3-24-4017 ----TAGCATCGGAGAGACCTATTACTAACATG---GTGAAAGTGACACTGATCGTTGCCATTGTGGCTGCTCTTGCTATCTCAGCTCACGCACAAAGAGATTACAATGAACTACGAGGAAATAAGAATGGCAGAGAGAGAGGACAAGGTCGCTTTGGAGGAAGGCCGGGTGGAATGCAGATGGGTGGAT 183  
3-24-4016 ----TAGCATCGGAGAGACCTATTACTAACATG---GTGAAAGTGACACTGATCGTTGCCATTGTGGCTGCTCTTGCTATCTCAGCTCACGCACAAAGAGATTACAATGAACTACGAGGAAATAAGAATGGCAGAGAGAGAGGACAAGGTCGCTTTGGAAGAAGGCCGGGTGGAATGCAGATGGGTGGAT 183  
3-24-4011 ------TAGCATCGGAGAGACCTTACTAACATGGAGGTGAAAGTGACACTGATCGTTGCCATTGTGGCTGCTCTTGCTATCTCGGCTCACACACAAAGAGACTACAATGAACGACGAGGAAATGAGAATGGCAGAGAGAGAGGACAAGGTCGCTTTGGAGGAAGGCCTGGTGGAATGCAGATGGGTGGAC 184  
3-24-4005 -----TAGCATCGTGAGAGACCTTACTAACATGGAGGTGAAAGTGACACTGATCGTTGCCATTGTGGCTGCTCTTGCTATCTCGGCTCACACACAAAGAGATTACAATGAACGACGAGGAAATGAGAATGGCAGAGAGAGAGGACAAGGTCGCTTTGGAGGAAGGCCTGGTGGAATGCAGATGGGTGGAC 185  
3-24-4018 -----TAGCATCGGTAGAGACCTTACTAACATGGAGGTGAAAGTGACACTGATCGTTGCCATTGTGGCTGCTCTTGCTATCTCGGCTCACACACAAAGAGATTACAATGAACGACGAGGAAATGAGAATGGCAGAGAGAGAGGACAAGGTCGCTTTGGAGGAAGGCCTGGTGGAATGCAGATGGGTGGAC 185  
3-24-4022 ------TAGCATCGGAGAGACCTTACTAACATGGAGGTGAAAGTGACACTGATCGTTGCCATTGTGGCTGCTCTTGCTATCTCGGCTCACACACAAAGAGATTACAATGAACGACGAGGAAATGAGAATGGCAGAGAGAGAGGACAAGGTCGCTTTGGAGGAAGGCCTGGTGGAATGCAGATGGGTGGAC 184  
6-2415    ----TAGCATCGGAGAGACCTATTACTATCATG---GTGAAAGTGACACTGATCGTTGCCATTGTGGCTGCTCTTGCTATCTCGGCTCACGCACGAAGAGATTTCAATGAACGACGAGGAAAGGAGAATGGCAGAGAGAGAGGACAAGGTGGCTTTGGAGGAAGGCCTGGTGGAATGCAGACGGGTAGTC 183  
6-2426    ----TAGCATCGGAGAGACCTATTACTATCATG---GTGAAAGTGACACTGATCGTTGCCATTGTGGCCGCTCTTGCTATCTCGGCTCACGCACGAAGAGATTTCAATGAACGACGAGGAAAGGAGAATGGCAGAGAGAGAGGACAAGGTGGCTTTGGAGGAAGGCCTGGTGGAATGCAGACGGGTAGTC 183  
6-2446    ----TAGCATCGGAGAGACCTATTACTATCATG---GTGAAAGTGACACTGATCGTTGCCATTGTGGCTGCTCTTGCTATCTCGGCTCACGCACGAAGAGATTTCAATGAACGACGAGGAAAGGAGAATGGCAGAGAGAGAGGACAAGGTGGCTTTGGAGGAAGGCCTGGTGGAATGCAGACGGGTAGTC 183  
6-2401    ------TAGCATCGGAGAGACCTTACAAACATGGAGGTGAAAGTGACACTGATCGTTGCCATTGTGGCTGCTCTTGCTATCTCGGCTCACGCACAAAGAGATTTCAATGAACGACGAGGAAAGGAGAATGACACAGAGAGAGGACAAGGTGGCTTTGGAGGAAGGCCGGGTGGAATGCAGATGGGTGGTC 184  
6-2402    ----TAGCATCGGAGAGACCTATTACTAACATGGAGGTGAAAGTGACACTGATCGTTGCCATTGTGGCTGCTCTTGCTATCTCGGCTCACGCACAAAGAGATTTCAATGAACGACGAGGAAAGGAGAATGACACAGAGAGAGGACAAGGTGGCTTTGGAGGAAGGCCTGGTGGAATGCAGATGGGTGGTC 186  
6-2404    ----TAGCATCGGAGAGACCTATTACTAACATGGAGGTGAAAGTGACACTGATCGTTGCCATTGTGGCTGCTCTTGCTATCTCGGCTCACGCACAAAGAGATTTCAATGAACGACGAGGAAAGGAGAATGACACAGAGAGAGGACAAGGTGGCTTTGGAGGAAGGCCTGGTGGAATGCAGATGGGTGGTC 186  
6-2407    ------TAGCATCGGAGAGACCTTACAAACATGGAGGTGAAAGTGACACTGATCGTTGCCATTGTGGCTGCTCTTGCTATCTCGGCTCACGCACAAAGAGATTTCAATGAACGACGAGGAAAGGAGAATGACACAGAGAGAGGACAAGGTGGCTTTGGAGGAAGGCCGGGTGGAATGCAGATGGGTGGTC 184  
6-2408    ----TAGCATCGGAGAGACCTATTACTAACATGGAGGTGAAAGTGACACTGATCGTTGCCATTGTGGCTGCTCTTGCTATCTCGGCTCACGCACAAAGAGATTTCAATGAACGACGAGGAAAGGAGAATGACACAGAGAGAGGACAAGGTGGCTTTGGAGGAAGGCCTGGTGGAATGCAGATGGGTGGTC 186  
6-2409    ------TAGCATCGGAGAGACCTTACAAACATGGAGGTGAAAGTGACACTGATCGTTGCCATTGTGGCTGCTCTTGCTATCTCGGCTCACGCACAAAGAGATTTCAATGAACGACGAGGAAAGGAGAATGACACAGAGAGAGGACAAGGTGGCTTTGGAGGAAGGCCGGGTGGAATGCAGATGGGTGGTC 184  
6-2410    ----TAGCATCGGAGAGACCTATTACTAACATGGAGGTGAAAGTGACACTGATCGTTGCCATTGTGGCTGCTCTTGCTATCTCGGCTCACGCACAAAGAGATTTCAATGAACGACGAGGAAAGGAGAATGACACAGAGAGAGGACAAGGTGGCTTTGGAGGAAGGCCTGGTGGAATGCAGATGGGTGGTC 186  
6-2411    ----TAGCATCGGAGAGACCTATTACTAACATGGAGGTGAAAGTGACACTGATCGTTGCCATTGTGGCTGCTCTTGCTATCTCGGCTCACGCACAAAGAGATTTCAACGAACGACGAGGAAAGGAGAATGACACAGAGAGAGGACAAGGTGGCTTTGGAGGAAGGCCGGGTGGAATGCAGATGGGTGGTC 186  
6-2412    ----TAGCATCGGAGAGACCTATTACTAACATGGAGGTGAAAGTGACACTGATCGTTGCCATTGTGGCTGCTCTTGCTATCTCGGCTCACGCACAAAGAGATTTCAATGAACGACGAGGAAAGGAGAATGACACAGAGAGAGGACAAGGTGGCTTTGGAGGAAGGCCTGGTGGAATGCAGATGGGTGGTC 186  
6-2413    ------TAGCATCGGAGAGACCTTACAAACATGGAGGTGAAAGTGACACTGATCGTTGCCATTGTGGCTGCTCTTGCTATCTCGGCTCACGCACAAAGAGATTTCAATGAACGACGAGGAAAGGAGAATGACACAGAGAGAGGACAAGGTGGCTTTGGAGGAAGGCCGGGTGGAATGCAGATGGGTGGTC 184  
6-2414    ----TAGCATCGGAGAGACCTATTACTAACATGGAGGTGAAAGTGACACTGATCGTTGCCATTGTGGCTGCTCTTGCTATCTCGGCTCACGCACAAAGAGATTTCAATGAACGACGAGGAAAGGAGAATGACACAGAGAGAGGACAAGGTGGCTTTGGAGGAAGGCCTGGTGGAATGCAGATGGGTGGTC 186  
6-2416    ------TAGCATCGGAGAGACCTTACAAACATGGAGGTGAAAGTGACACTGATCGTTGCCATTGTGGCTGCTCTTGCTATCTCGGCTCACGCACAAAGAGATTTCAATGAACGACGAGGAAAGGAGAATGACACAGAGAGAGGACAAGGTGGCTTTGGAGGAAGGCCGGGTGGAATGCAGATGGGTGGTC 184  
6-2417    ----TAGCATCGGAGAGACCTATTACTAACATGGAGGTGAAAGTGACACTGATCGTTGCCATTGTGGCTGCTCTTGCTATCTCGGCTCACGCACAAAGAGATTTCAATGAACGACGAGGAAAGGAGAATGACACAGAGAGAGGACAAGGTGGCTTTGGAGGAAGGCCTGGTGGAATGCAGATGGGTGGTC 186  
6-2420    ------TAGCATCGGAGAGACCTTACAAACATGGAGGTGAAAGTGACACTGATCGTTGCCATTGTGGCTGCTCTTGCTATCTCGGCTCACGCACAAAGAGATTTCAATGAACGACGAGGAAAGGAGAATGACACAGAGAGAGGACAAGGTGGCTTTGGAGGAAGGCCGGGTGGAATGCAGATGGGTGGTC 184  
6-2421    ------TAGCATCGGAGAGACCTTACAAACATGGAGGTGAAAGTGACACTGATCGTTGCCATTGTGGCTGCTCTTGCTATCTCGGCTCACGCACAAAGAGATTTCAATGAACGACGAGGAAAGGAGAATGACACAGAGAGAGGACAAGGTGGCTTTGGAGGAAGGCCGGGTGGAATGCAGATGGGTGGTC 184  
6-2422    ----TAGCATCGGAGAGACCTATTACTAACATGGAGGTGAAAGTGACACTGATCGTTGCCATTGTGGCTGCTCTTGCTATCTCGGCTCACGCACAAAGAGATTTCAATGAACGACGAGGAAAGGAGAATGACACAGAGAGAGGACAAGGTGGCTTTGGAGGAAGGCCTGGTGGAATGCAGATGGGTGGTC 186  
6-2424    ------TAGCATCGGAGAGACCTTACAAACATGGAGGTGAAAGTGACACTGATCGTTGCCATTGTGGCTGCTCTTGCTATCTCGGCTCACGCACAAAGAGATTTCAATGAACGACGAGGAAAGGAGAATGACACAGAGAGAGGACAAGGTGGCTTTGGAGGAAGGCCGGGTGGAATGCAGATGGGTGGTC 184  
6-2425    ----TAGCATCGGAGAGACCTATTACTAACATGGAGGTGAAAGTGACACTGATCGTTGCCATTGTGGCTGCTCTTGCTATCTCGGCTCACGCACAAAGAGATTTCAATGAACGACGAGGAAAGGAGAATGACACAGAGAGAGGACAAGGTGGCTTTGGAGGAAGGCCTGGTGGAATGCAGATGGGTGGTC 186  
6-2427    ------TAGCATCGGAGAGACCTTACAAACATGGAGGTGAAAGTGACACTGATCGTTGCCATTGTGGCTGCTCTTGCTATCTCGGCTCACGCACAAAGAGATTTCAATGAACGACGAGGAAAGGAGAATGACACAGAGAGAGGACAAGGTGGCTTTGGAGGAAGGCCGGGTGGAATGCAGATGGGTGGTC 184  
6-2428    ------TAGACTCGGAGAGACCTTACTAACATGGAGGTGAAAGTGACACTGATCGTTGCCATTGTGGCTGCTCTTGCTATCTCGGCTCACGCACAAAGAGATTTCAATGAACGACGAGGAAAGGAGAATGACACAGAGAGAGGACAAGGTGGCTTTGGAGGAAGGCCTGGTGGAATGCAGATGGGTGGTC 184  
6-2430    ----TAGCATCGGAGAGACCTATTACTAACATGGAGGTGAAAGTGACACTGATCGTTGCCATTGTGGCTGCTCTTGCTATCTCGGCTCACGCACAAAGAGATTTCAATGAACGACGAGGAAAGGAGAATGACACAGAGAGAGGACAAGGTGGCTTTGGAGGAAGGCCTGGTGGAATGCAGATGGGTGGTC 186  
6-2431    ------TAGCATCGGAGAGACCTTACAAACATGGAGGTGAAAGTGACACTGATCGTTGCCATTGTGGCTGCTCTTGCTATCTCGGCCCACGCACAAAGAGATTTCAATGAACGACGAGGAAAGGAGAATGACACAGAGAGAGGACAAGGTGGCTTTGGAGGAAGGCCGGGTGGAATGCAGATGGGTGGTC 184  
6-2432    ------TAGCATCGGAGAGACCTTACAAACATGGAGGTGAAAGTGACACTGATCGTTGCCATTGTGGCTGCTCTTGCTATCTCGGCTCACGCACAAAGAGATTTCAATGAACGACGAGGAAAGGAGAATGACACAGAGAGAGGACAAGGTGGCTTTGGAGGAAGGCCGGGTGGAATGCAGATGGGTGGTC 184  
6-2433    ------TAGCATCGGAGAGACCTTACAAACATGGAGGTGAAAGTGACACTGATCGTTGCCATTGTGGCTGCTCTTGCTATCTCGGCTCACGCACAAAGAGATTTCAATGAACGACGAGGAAAGGAGAATGACACAGAGAGAGGACAAGGTGGCTTTGGAGGAAGGCCGGGTGGAATGCAGATGGGTGGTC 184  
6-2434    ------TAGCATCGGAGAGACCTTACAAACATGGAGGTGAAAGTGACACTGATCGTTGCCATTGTGGCTGCTCTTGCTATCTCGGCTCACGCACAAAGAGATTTCAATGAACGACGAGGAAAGGAGAATGACACAGAGAGAGGACAAGGTGGCTTTGGAGGAAGGCCGGGTGGAATGCAGATGGGTGGTC 184  
6-2439    ------TAGCATCGGAGAGACCTTACAAACATGGAGGTGAAAGTGACACTGATCGTTGCCATTGTGGCTGCTCTTGCTATCTCGGCTCACGCACAAAGAGATTTCAATGAACGACGAGGAAAGGAGAATGACACAGAGAGAGGACAAGGTGGCTTTGGAGGAAGGCCGGGTGGAATGCAGATGGGTGGTC 184  
6-2440    ----TAGCATCGGAGAGACCTATTACTAACATGGAGGTGAAAGTGACACTGATCGTTGCCATTGTGGCTGCTCTTGCTATCTCGGCTCACGCACAAAGAGATTTCAATGAACGACGAGGAAAGGAGAATGACACAGAGAGAGGACAAGGTGGCTTTGGAGGAAGGCCTGGTGGAATGCAGATGGGTGGTC 186  
6-2441    ----TAGCATCGGAGAGACCTATTACTAACATGGAGGTGAAAGTGACACTGATCGTTGCCATTGTGGCTGCTCTTGCTATCTCGGCTCACGCACAAAGAGATTTCAATGAACGACGAGGAAAGGAGAATGACACAGAGAGAGGACAAGGTGGCTTTGGAGGAAGGCCTGGTGGAATGCAGATGGGTGGTC 186  
6-2435    -------AGCATCGGAGAGACCTTACAAACATGGAGGTGAAAGTGACACTGATCGTTGCCATTGTGGCTGCTCTTGCTATCTCGGCTCACGCACAAAGAGATTTCAATGAACGACGAGGAAAGGAGAATGACACAGAGAGAGGACAAGGTGGCTTTGGAGGAAGGCCGGGTGGAATGCAGATGGGTGGTC 183  
6-2447    ------TAGCATCGGAGAGACCTTACAAACATGGAGGTGAAAGTGACACTGATCGTTGCCATTGTGGCTGCTCTTGCTATCTCGGCTCACGCACAAAGAGATTTCAATGAACGACGAGGAAAGGAGAATGACACAGAGAGAGGACAAGGTGGCTTTGGAGGAAGGCCGGGTGGAATGCAGATGGGTGGTC 184  
6-2448    ----TAGCATCGGAGAGACCTATTACTAACATGGAGGTGAAAGTGACACTGATCGTTGCCATTGTGGCTGCTCTTGCCATCTCGGCTCACGCACAAAGAGATTTCAATGAACGACGAGGAAAGGAGAATGACACAGAGAGAGGACAAGGTGGCTTTGGAGGAAGGCCTGGTGGAATGCAGATGGGTGGTC 186  
6-2436    -----AGCATCGGAGAGACCTATTACTAACATGGAGGTGAAAGTGACACTGATCGTTGCCATTGTGGCTGCTCTTGCTATCTCGGCTCACGCACAAAGAGATTTCAATGAACGACGAGGAAAGGAGAATGACACAGAGAGAGGACAAGGTGGCTTTGGAGGAAGGCCTGGTGGAATGCAGATGGGTGGTC 185  
6-2450    ------TAGCATCGGAGAGACCTTACAAACATGGAGGTGAAAGTGACACTGATCGTTGCCATTGTGGCTGCTCTTGCTATCTCGGCTCACGCACAAAGAGATTTCAATGAACGACGAGGAAAGGAGAATGACACAGAGAGAGGACAAGGTGGCTTTGGAGGAAGGCCGGGTGGAATGCAGATGGGTGGTC 184  
6-2429    ------TAGCATCGGAGAGACCTTACAAACATGGAGGTGAAAGTGACACTGATCGTTGCCATTGTGGCTGCTCTTGCTATCTCGGCTCACGCACAAAGAGATTTCAATGAACGACGAGGAAAGGAGAATGACACAGAGAGAGGACAAGGTGGCTTTGGAGGAAGGCCGGGTGGAATGCAGATGGGTGGTC 184  
6-2438    ------TAGCATCGGAGAGACCTTACAAACATGGAGGTGAAAGCAACATTGATCGTTGCCATTGTGGCTGCTCTTGCTATCTCGGCTCACGCACGAAGAGATTTCAATGAACGGCGAGGAAATGAGAATGGCAGAGAGAGAGGACAAGGTCGCTTTGGAGGAAGGCCTGGTGGAATGCAGATGGGTGGAT 184  
6-2423    ------TAGCATCGGAGAGACCTTACAAACATGGAGGTGAAAGCAACATTGATCGTTGCCATTGTGGCTGCTCTTGCTATCTCGGCTCACGCACGAAGAGATTTCAATGAACGGCGAGGAAATGAGAATGGCAGAGAGAGAGGACAAGGTCGCTTTGGAGGAAGGCCTGGTGGAATGCAGATGGGTGGAT 184  
6-2403    ----TAGCATCGGAGAGACCTATTACTAACATGGAGGTGAAAGTGACACTGATCGTTGCCATTGTGGCTGCTCTTGCTATCTCGGCTCACGCACAAAGAGATTTCAATGAACGACGAGGAAAGGAGAATGACACAGAGAGAGGACAAGGTGGCTTTGGAGGAAGGCCTGGTGGAATGCAGATGGGTGGTC 186  
6-2444    ----TAGCATCGGAGAGACCTATTACTAACATGGAGGTGAAAGTGACACTGATCGTTGCCATTGTGGCTGCTCTTGCTATCTCGGCTCACGCACAAAGAGATTTCAATGAACGACGAGGAAAGGAGAATGACACAGAGAGAGGACAAGGTGGCTTTGGAGGAAGGCCTGGTGGAATGCAGATGGGTGGTC 186  
6-2449    --TAGATAGCATCGGAGAGACCTTACAAACATGGAGGTGTAAGTGACACTGATCGTTGCCATTGTGGCTGCTCTTGCTATCTCGGCTCACGCACAAAGAGATTTCAATGAACGACGAGGAAAGGAGAATGACACAGAGAGAGGACAAGGTGGCTTTGGAGGAAGGCCGGGTGGAATGCAGATGGGTGGTC 188  
2-1503    -TTGTAGCATCGGAGAGACCTATTACTAACATGGAGGTGAAAGCGACACTGATCGTTGCCATTGTGGCTGCTCTTGCTATCTCGGCTCACGCACAAAGAGATTTCAATGAACGACGAGGAAAGGAGAATGACACAGAGAGAGGACAAGGTGGCTTTGGAGGAAGGCCTGGTGGAATGCAGATGGGTGGTC 189  
2-1509    -TTATAGCATCGGAGAGACCTATTACTAACATGGAGGTGAAAGTGACACTGATCGTTGCCATTGTGGCTGCTCTTGCTATCTCGGCTCACGCACAAAGAGATTTCAATGAACGACGAGGAAAGGAGAATGACACAGAGAGAGGACAAGGTGGCTTTGGAGGAAGGCCTGGTGGAATGCAGATGGGTGGTC 189  
2-1513    -TTGTAGCATCGGAGAGACCTATTACTAACATGGAGGTGAAAGTGACACTGATCGTTGCCATTGTGGCTGCTCTTGCTATCTCGGCTCACGCACAAAGAGATTTCAATGAACGACGAGGAAAGGAGAATGACACAGAGAGAGGACAAGGTGGCTTTGGAGGAAGGCCTGGTGGAATGCAGATGGGTGGTC 189  
2-1523    -CTGTAGCATCGGAGAGACCTATTACTAACATGGAGGTGAAAGTGACACTGATCGTTGCCATTGTGGCTGCTCTTGCTATCTCGGCTCACGCACAAAGAGATTTCAATGAACGACGAGGAAAGGAGAATGACACAGAGAGAGGACAAGGTGGCTTTGGAGGAAGGCCTGGTGGAATGCAGATGGGTGGTC 189  
2-1524    -CTTTAGCATCGGAGAGACCTATTACTAACATGGAGGTGAAAGTGACACTGATCGTTGCCATTGTGGCTGCTCTTGCTATCTCGGCTCACGCACAAAGAGATTTCAATGAACGACGAGGAAAGGAGAATGACACAGAGAGAGGACAAGGTGGCTTTGGAGGAAGGCCTGGTGGAATGCAGATGGGTGGTC 189  
2-1531    -CTGTAGCATCGGAGAGACCTATTACTAACATGGAGGTGAAAGTGACACTGATCGTTGCCATTGTGGCTGCTCTTGCTATCTCGGCTCACGCACAAAGAGATTTCAATGAACGACGAGGAAAGGAGAATGACACAGAGAGAGGACAAGGTGGCTTTGGAGGAAGGCCTGGTGGAATGCAGATGGGTGGTC 189  
2-1533    -CTGTAGCATCGGAGAGACCTATTACTAACATGGAGGTGAAAGTGACACTGATCGTTGCCATTGTGGCTGCTCTTGCTATCTCGGCTCACGCACAAAGAGATTTCAATGAACGACGAGGAAAGGAGAATGACACAGAGAGAGGACAAGGTGGCTTTGGAGGAAGGCCTGGTGGAATGCAGATGGGTGGTC 189  
2-1536    --TGTAGCATCGGAGAGACCTATTACTAACATGGAGGTGAAAGTGACACTGATCGTTGCCATTGTGGCTGCTCTTGCTATCTCGGCTCACGCACAAAGAGATTTCAATGAACGACGAGGAAAGGAGAATGACACAGAGAGAGGACAAGGTGGCTTTGGAGGAAGGCCTGGTGGAATGCAGATGGGTGGTC 188  
2-1502    -CTGTAGCATCGGAGAGACCTATTACTAACATGGAGGTGAAAGTGACACTGATCGTTGCCATTGTGGCTGCTCTTGCTATCTCGGCTCACGCACAAAGAGATTTCAATGAACGACGAGGAAAGGAGAATGACACAGAGAGAGGACAAGGTGGCTTTGGAGGAAGGCCTGGTGGAATGCAGATGGGTGGTC 189  
2-1518    ---TTGTAGCATCGGAGAGACCTTACAAACATGGAGGTGAAAGTGACACTGATCGTTGCCATTGTGGCTGCTCTTGCTATCTCGGCTCACGCACAAAGAGATTTCAATGAACGACGAGGAAAGGAGAATGACACAGAGAGAGGACAAGGTGGCTTTGGAGGAAGGCCGGGTGGAATGCAGATGGGTGGTC 187  
2-1519    ---CTATAGCATCGGAGAGACCTTACAAACATGGAGGTGAAAGTGACACTGATCGTTGCCATTGTGGCTGCTCTTGCTATCTCGGCTCACGCACAAAGAGATTTCAATGAACGACGAGGAAAGGAGAATGACACAGAGAGAGGACAAGGTGGCTTTGGAGGAAGGCCGGGTGGAATGCAGATGGGTGGTC 187  
2-1511    ---CTGTAGCATCGGAGAGACCTTACAAACATGGAGGTGAAAGTGACACTGATCGTTGCCATTGTGGCTGCTCTTGCTATCTCGGCTCACGCACAAAGAGATTTCAATGAACGACGAGGAAAGGAGAATGACACAGAGAGAGGACAAGGTGGCTTTGGAGGAAGGCCGGGTGGAATGCAGATGGGTGGTC 187  
2-1546    ---CTGTAGCATCGGAGAGACCTTACAAACATGGAGGTGAAAGTGACACTGATCGTTGCCATTGTGGCTGCTCTTGCTATCTCGGCTCACGCACAAAGAGATTTCAATGAACGACGAGGAAAGGAGAATGACACAGAGAGAGGACAAGGTGGCTTTGGAGGAAGGCCGGGTGGAATGCAGATGGGTGGTC 187  
2-1548    ---CTGTAGCATCGGAGAGACCTTACAAACATGGAGGTGAAAGTGACACTGATCGTTGCCATTGTGGCTGCTCTTGCTATCTCGGCTCACGCACAAAGAGATTTCAATGAACGACGAGGAAAGGAGAATGACACAGAGAGAGGACAAGGTGGCTTTGGAGGAAGGCCGGGTGGAATGCAGATGGGTGGTC 187  
2-1540    -CTTTAGCATCGGAGAGACCTATTACTAACATGGAGGTGAAAGTGACACTGATCGTTGCCATTGTGGCTGCTCTTGCTATCTCGGCTCACGCACAAAGAGATTTCAATGAACGACGAGGAAAGGAGAATGACACAGAGAGAGGACAAGGTGGCTTTGGAGGAAGGCCTGGTGGAATGCAGATGGGTGGTC 189  
2-2423    ----TAGCATCGGAGAGACCTATTACTAACATGGAGGTGAAAGTGACACTGATCGTTGCCATTGTGGCTGCTCTTGCTATCTCGGCTCACGCACAAAGAGATTTCAATGAACGACGAGGAAAGGAGAATGACACAGAGAGAGGACAAGGTGGCTTTGGAGGAAGGCCTGGTGGAATGCAGATGGGTGGTC 186  
2-2436    ----TAGCATCGGAGAGACCTATTACTAACATGGAGGTGAAAGTGACACTGATCGTTGCCATTGTGGCTGCTCTTGCTATCTCGGCTCACGCACAAAGAGATTTCAATGAACGACGAGGAAAGGAGAATGACACAGAGAGAGGACAAGGTGGCTTTGGAGGAAGGCCTGGTGGAATGCAGATGGGTGGTC 186  
2-2403    ---TAGGCATCGGAGAGATCTATTACTAACATGGAGGTGAAAGTGACACTGATCGTTGCCATTGTGGCTGCTCTTGCTATCTCGGCTCACGCACAAAGAGATTTCAATGAACGACGAGGAAAGGAGAATGACACAGAGAGAGGACAAGGTGGCTTTGGAGGAAGGCCTGGTGGAATGCAGATGGGTGGTC 187  
2-2448    ----TAGCATCGGAGAGACCTATTACTAACATGGAGGTGAAAGTGACACTGATCGTTGCCATTGTGGCTGCTCTTGCTATCTCGGCTCACGCACAAAGAGATTTCAATGAACGACGAGGAAAGGAGAATGACACAGAGAGAGGACAAGGTGGCTTTGGAGGAAGGCCTGGTGGAATGCAGATGGGTGGTC 186  
2-2404    ---TAGTCATCGGAGAGACCTATTACTAACATGGAGGTGAAAGTGACACTGATCGTTGCCATTGTGGCTGCTCTTGCTATCTCGGCTCACGCACAAAGAGATTTCAATGAACGACGAGGAAAGGAGAATGACACAGAGAGAGGACAAGGTGGCTTTGGAGGAAGGCCTGGTGGAATGCAGATGGGTGGTC 187  
2-2405    GCACCCATGACCTGCGAGAGACCTAGCAACATGGAGGTGAAAGTGACACTGATCGTTGCCATTGTGGCTGCTCTTGCTATCTCGGCTCACGCACAAAGAGATTTCAATGAACGACGAGGAAAGGAGAATGACACAGAGAGAGGACAAGGTGGCTTTGGAGGAAGGCCGGGTGGAATGCAGATGGGTGGTC 190  
2-2406    ------TAGCTCGGAGAGACCATTACTAACATGGAGGTGAAAGTGACACTGATCGTTGCCATTGTGGCTGCTCTTGCTATCTCGGCTCACGCACAAAGAGATTTCAATGAACGACGAGGAAAGGAGAATGACACAGAGAGAGGACAAGGTGGCTTTGGAGGAAGGCCTGGTGGAATGCAGATGGGTGGTC 184  
2-2409    ----TAGTCTCGGAGAGACCTATTACTAACATGGAGGTGAAAGTGACACTGATCGTTGCCATTGTGGCTGCTCTTGCTATCTCGGCTCACGCACAAAGAGATTTCAATGAACGACGAGGAAAGGAGAATGACACAGAGAGAGGACAAGGTGGCTTTGGAGGAAGGCCTGGTGGAATGCAGATGGGTGGTC 186  
2-2410    ---TAGCATCGGAGAGATCTATTACTACACATGGAGGTGAAAGTGACACTGATCGTTGCCATTGTGGCTGCTCTTGCTATCTCGGCTCACGCACAAAGAGATTTCAATGAACGACGAGGAAAGGAGAATGACACAGAGAGAGGACAAGGTGGCTTTGGAGGAAGGCCTGGTGGAATGCAGATGGGTGGTC 187  
2-2411    ---TTAGCATCGGAGAGACCTATTACTAACATGGAGGTGAAAGTGACACTGATCGTTGCCATTGTGGCTGCTCTTGCTATCTCGGCTCACGCACAAAGAGATTTCAATGAACGACGAGGAAAGGAGAATGACACAGAGAGAGGACAAGGTGGCTTTGGAGGAAGGCCTGGTGGAATGCAGATGGGTGGTC 187  
2-2412    ----TAGCATCGGAGAGACCTATTACTAACATGGAGGTGAAAGTGACACTGATCGTTGCCATTGTGGCTGCTCTTGCTATCTCGGCTCACGCACAAAGAGATTTCAATGAACGACGAGGAAAGGAGAATGACACAGAGAGAGGACAAGGTGGCTTTGGAGGAAGGCCTGGTGGAATGCAGATGGGTGGTC 186  
2-2413    ----TAGCATCGGAGAGACCTATTACTAACATGGAGGTGAAAGTGACACTGATCGTTGCCATTGTGGCTGCTCTTGCTATCTCGGCTCACGCACAAAGAGATTCCAATGAACGACGAGGAAAGGAGAATGACACAGAGAGAGGACAAGGTGGCTTTGGAGGAAGGCCTGGTGGAATGCAGATGGGTGGTC 186  
2-2415    ---TAGACATCGGAGAGACCTATTACTAACATGGAGGTGAAAGTGACACTGATCGTTGCCATTGTGGCTGCTCTTGCTATCTCGGCTCACGCACAAAGAGATTTCAATGAACGACGAGGAAAGGAGAATGACACAGAGAGAGGACAAGGTGGCTTTGGAGGAAGGCCTGGTGGAATGCAGATGGGTGGTC 187  
2-2416    ----TAGCATCGGAGAGACCTATTACTAACATGGAGGTGAAAGTGACACTGATCGTTGCCATTGTGGCTGCTCTTGCTATCTCGGCTCACGCACAAAGAGATTTCAATGAACGACGAGGAAAGGAGAATGACACAGAGAGAGGACAAGGTGGCTTTGGAGGAAGGCCTGGTGGAATGCAGATGGGTGGTC 186  
2-2417    ----TATCATCGGAGAGACCTATTACTAACATGGAGGTGAAAGTGACACTGATCGTTGCCATTGTGGCTGCTCTTGCTATCTCGGCTCACGCACAAAGAGATTTCAATGAACGACGAGGAAAGGAGAATGACACAGAGAGAGGACAAGGTGGCTTTGGAGGAAGGCCTGGTGGAATGCAGATGGGTGGTC 186  
2-2418    ----TAGCATCGGAGAGACCTATTACTAACATGGAGGTGAAAGTGACACTGATCGTTGCCATTGTGGCTGCTCTTGCTATCTCGGCTCACGCACAAAGAGATTTCAATGAACGACGAGGAAAGGAGAATGACACAGAGAGAGGACAAGGTGGCTTTGGAGGAAGGCCTGGTGGAATGCAGATGGGTGGTC 186  
2-2419    ----TAGCATCGGAGAGACCTATTACTAACATGGAGGTGAAAGTGACACTGATCGTTGCCATTGTGGCTGCTCTTGCTATCTCGGCTCACGCACAAAGAGATTTCAATGAACGACGAGGAAAGGAGAATGACACAGAGAGAGGACAAGGTGGCTTTGGAGGAAGGCCTGGTGGAATGCAGATGGGTGGTC 186  
2-2420    ---AGACATCCGAGAGATCCTTCATCAAGCATGGAGGTGAAAGTGACACTGATCGTTGCCATTGTGGCTGCTCTTGCTATCTCGGCTCACGCACAAAGAGATTTCAATGAACGACGAGGAAAGGAGAATGACACAGAGAGAGGACAAGGTGGCTTTGGAGGAAGGCCTGGTGGAATGCAGATGGGTGGTC 187  
2-2421    ----TAGCATCGGAGAGACCTATTACTAACATGGAGGTGAAAGTGACACTGATCGTTGCCATTGTGGCTGCTCTTGCTATCTCGGCTCACGCACAAAGAGATTTCAATGAACGACGAGGAAAGGAGAATGACACAGAGAGAGGACAAGGTGGCTTTGGAGGAAGGCCTGGTGGAATGCAGATGGGTGGTC 186  
2-2422    ----TAGCATCGGAGAGACCTATTACTAACATGGAGGTGAAAGTGACACTGATCGTTGCCATTGTGGCTGCTCTTGCTATCTCGGCTCACGCACAAAGAGATTTCAATGAACGACGAGGAAAGGAGAATGACACAGAGAGAGGACAAGGTGGCTTTGGAGGAAGGCCTGGTGGAATGCAGATGGGTGGTC 186  
2-2424    --TAGACATACGGAGAGACCTATCACTAACATGGAGGTGAAAGTGACACTGATCGTTGCCATTGTGGCTGCTCTGACTATCTCGGCTCACGCACAAAGAGATTTCAATGAACGACGAGGAAAGGAGAATGACACAGAGAGAGGACAAGGTGGCTTTGGAGGAAGGCCTGGTGGAATGCAGATGGGTGGTC 188  
2-2425    ----TAGCATCGGAGAGACCTATTACTAACATGGAGGTGAAAGTGACACTGATCGTTGCCATTGTGGCTGCTCTTGCTATCTCGGCTCACGCACAAAGAGATTTCAATGAACGACGAGGAAAGGAGAATGACACAGAGAGAGGACAAGGTGGCTTTGGAGGAAGGCCTGGTGGAATGCAGATGGGTGGTC 186  
2-2426    ---TTAGCATCGGAGAGACCTATTACTAACATGGAGGTGAAAGTGACACTGATCGTTGCCATTGTGGCTGCTCTTGCTATCTCGGCTCACGCACAAAGAGATTTCAATGAACGACGAGGAAAGGAGAATGACACAGAGAGAGGACAAGGTGGCTTTGGAGGAAGGCCTGGTGGAATGCAGATGGGTGGTC 187  
2-2427    ---GAGTCGGCGGAGAGACCTATTACTAACATGGAGGTGAAAGTGACACTGATCGTTGCCATTGTGGCTGCTCTTGCTATCTCGGCTCACGCACAAAGAGATTTCAATGAACGACGAGGAAAGGAGAATGACACAGAGAGAGGACAAGGTGGCTTTGGAGGAAGGCCTGGTGGAATGCAGATGGGTGGTC 187  
2-2430    ----TAGACATCGGAGAGACCATTACTAACATGGAGGTGAAAGTGACACTGATCGTTGCCATTGTGGCTGCTCTTGCTATCTCGGCTCACGCACAAAGAGATTTCAATGAACGACGAGGAAAGGAGAATGACACAGAGAGAGGACAAGGTGGCTTTGGAGGAAGGCCTGGTGGAATGCAGATGGGTGGTC 186  
2-2431    -----------CGGAGAGACCATTACTAACATGGAGGTGAAAGTGACACTGATCGTTGCCATTGTGGCTGCTCTTGCTATCTCGGCTCACGCACAAAGAGATTTCAATGAACGACGAGGAAAGGAGAATGACACAGAGAGAGGACAAGGTGGCTTTGGAGGAAGGCCTGGTGGAATGCAGATGGGTGGTC 179  
2-2432    ----TAGCATCGGAGAGACCTATTACTAACATGGAGGTGAAAGTGACACTGATCGTTGCCATTGTGGCTGCTCTTGCTATCTCGGCTCACGCACAAAGAGATTTCAATGAACGACGAGGAAAGGAGAATGACACAGAGAGAGGACAAGGTGGCTTTGGAGGAAGGCCTGGTGGAATGCAGATGGGTGGTC 186  
2-2434    ----TAGACTACGGAGAGACCATTACTACCATGGAGGTGAAAGTGACACTGATCGTTGCCATTGTGGCTGCTCTTGCTATCTCGGCTCACGCACAAAGAGATTTCAATGAACGACGAGGAAAGGAGAATGACACAGAGAGAGGACAAGGTGGCTTTGGAGGAAGGCCTGGTGGAATGCAGATGGGTGGTC 186  
2-2437    ---TTAGCATCGGAGAGACCTATTACTAACATGGAGGTGAAAGTGACACTGATCGTTGCCATTGTGGCTGCTCTTGCTATCTCGGCTCACGCACAAAGAGATTTCAATGAACGACGAGGAAAGGAGAATGACACAGAGAGAGGACAAGGTGGCTTTGGAGGAAGGCCTGGTGGAATGCAGATGGGTGGTC 187  
2-2438    ----TAGCATCGGAGAGACCTATTACTAACATGGAGGTGAAAGTGACACTGATCGTTGCCATTGTGGCTGCTCTTGCTATCTCGGCTCACGCACAAAGAGATTTCAATGAACGACGAGGAAAGGAGAATGACACAGAGAGAGGACAAGGTGGCTTTGGAGGAAGGCCTGGTGGAATGCAGATGGGTGGTC 186  
2-2439    ----TAGACATCGGAGAGACCATTACTAACATGGAGGTGAAAGTGACACTGATCGTTGCCATTGTGGCTGCTCTTGCTATCTCGGCTCACGCACAAAGAGATTTCAATGAACGACGAGGAAAGGAGAATGACACAGAGAGAGGACAAGGTGGCTTTGGAGGAAGGCCTGGTGGAGTGCAGATGGGTGGTC 186  
2-2440    ------CTGCGGAGAGACCTATTGACTAACATGGAGGTGAAAGTGACACTGATCGTTGCCATTGTGGCTGCTCTTGCTATCTCGGCTCACGCACAAAGAGATTTCAATGAACGACGAGGAAAGGAGAATGACACAGAGAGAGGACAAGGTGGCTTTGGAGGAAGGCCTGGTGGAATGCAGATGGGTGGTC 184  
2-2442    -----AGCATCGGAGAGACCTATTACTAACATGGAGGTGAAAGTGACACTGATCGTTGCCATTGTGGCTGCTCTTGCTATCTCGGCTCACGCACAAAGAGATTTCAATGAACGACGAGGAAAGGAGAATGACACAGAGAGAGGACAAGGTGGCTTTGGAGGAAGGCCTGGTGGAATGCAGATGGGTGGTC 185  
2-2445    -------CATCGGAGAGACCTATTACTAACATGGAGGTGAAAGTGACACTGATCGTTGCCATTGTGGCTGCTCTTGCTATCTCGGCTCACGCACAAAGAGATTTCAATGAACGACGAGGAAAGGAGAATGACACAGAGAGAGGACAAGGTGGCTTTGGAGGAAGGCCGGGTGGAATGCAGATGGGTGGTC 183  
2-2446    ----TAGGCTGCGGAGAGACCATTACTAACATGGAGGTGAAAGTGACACTGATCGTTGCCATTGTGGCTGCTCTTGCTATCTCGGCTCACGCACAAAGAGATTTCAATGAACGACGAGGAAAGGAGAATGACACAGAGAGAGGACAAGGTGGCTTTGGAGGAAGGCCTGGTGGAATGCAGATGGGTGGTC 186  
7-1501    ----TAGCATCGGAGAGACCTATTACTAACATGGAGGTGAAAGTGACACTGATCGTTGCCATTGTGGCTGCTCTTGCTATCTCGGCTCACGCACAAAGAGATTTCAATGAACGACGAGGAAAGGAGAATGACACAGAGAGAGGACAAGGTGGCTTTGGAGGAAGGCCTGGTGGAATGCAGATGGGTGGTC 186  
7-1502    ----TAGCATCGGAGAGACCTATTACTAACATGGAGGTGAAAGTGACACTGATCGTTGCCATTGTGGCTGCTCTTGCTATCTCGGCTCACGCACAAAGAGATTTCAATGAACGACGAGGAAAGGAGAGTGACACAGAGAGAGGACAAGGTGGCTTTGGAGGAAGGCCTGGTGGAATGCAGATGGGTGGTC 186  
7-1503    ----TAGCATCGGAGAGACCTATTACTAACATGGAGGTGAAAGTGACACTGATCGTTGCCATTGTGGCTGCTCTTGCTATCTCGGCTCACGCACAAAGAGATTTCAATGAACGACGAGGAAAGGAGAATGACACAGAGAGAGGACAAGGTGGCTTTGGAGGAAGGCCTGGTGGAATGCAGATGGGTGGTC 186  
7-1504    ----TAGCATCGGAGAGACCTATTACTAACATGGAGGTGAAAGTGACACTGATCGTTGCCATTGTGGCTGCTCTTGCTATCTCGGCTCACGCACAAAGAGATTTCAATGAACGACGAGGAAAGGAGAATGACACAGAGAGAGGACAAGGTGGCTTTGGAGGAAGGCCTGGTGGAATGCAGATGGGTGGTC 186  
7-1505    ----TAGCATCGGAGAGACCTATTACTAACATGGAGGTGAAAGTGACACTGATCGTTGCCATTGTGGCTGCTCTTGCTATCTCGGCTCACGCACAAAGAGATTTCAATGAACGACGAGGAAAGGAGAATGACACAGAGAGAGGACAAGGTGGCTTTGGAGGAAGGCCTGGTGGAATGCAGATGGGTGGTC 186  
7-1506    ----TAGCATCGGAGAGACCTATTACTAACATGGAGGTGAAAGTGACACTGATCGTTGCCATTGTGGCTGCTCTTGCTATCTCGGCTCACGCACAAAGAGATTTCAATGAACGACGAGGAAAGGAGAATGACACAGAGAGAGGACAAGGTGGCTTTGGAGGAAGGCCTGGTGGAATGCAGATGGGTGGTC 186  
7-1508    ----TAGCATCGGAGAGACCTATTACTAACATGGAGGTGAAAGTGACACTGATCGTTGCCATTGTGGCTGCTCTTGCTATCTCGGCTCACGCACAAAGAGATTTCAATGAACGACGAGGAAAGGAGAATGACACAGAGAGAGGACAAGGTGGCTTTGGAGGAAGGCCTGGTGGAATGCAGATGGGTGGTC 186  
7-1509    ----TAGCATCGGAGAGACCTATTACTAACATGGAGGTGAAAGTGACACTGATCGTTGCCATTGTGGCTGCTCTCGCTATCTCGGCTCACGCACAAAGAGATTTCAATGAACGACGAGGAAAGGAGAATGACACAGAGAGAGGACAAGGTGGCTTTGGAGGAAGGCCTGGTGGAATGCAGATGGGTGGTC 186  
7-1510    ----TAGCATCGGAGAGACCTATTACTAACATGGAGGTGAAAGTGACACTGATCGTTGCCATTGTGGCTGCTCTTGCTATCTCGGCTCACGCACAAAGAGATTTCAATGAACGACGAGGAAAGGAGAATGACACAGAGAGAGGACAAGGTGGCTTTGGAGGAAGGCCTGGTGGAATGCAGATGGGTGGTC 186  
7-1511    ----TAGCATCGGAGAGACCTATTACTAACATGGAGGTGAAAGTGACACTGATCGTTGCCATTGTGGCTGCTCTTGCTATCTCGGCTCACGCACAAAGAGATTTCAATGAACGACGAGGAAAGGAGAATGACACAGAGAGAGGACAAGGTGGCTTTGGAGGAAGGCCTGGTGGAATGCAGATGGGTGGTC 186  
7-1512    ----TAGCATCGGAGAGACCTATTACTAACATGGAGGTGAAAGTGACACTGATCGTTGCCATTGTGGCTGCTCTTGCTATCTCGGCTCACGCACAAAGAGATTTCAATGAACGACGAGGAAAGGAGAATGACACAGAGAGAGGACAAGGTGGCTTTGGAGGAAGGCCTGGTGGAATGCAGATGGGTGGTC 186  
7-1513    ----TAGCATCGGAGAGACCTATTACTAACATGGAGGTGAAAGTGACACTGATCGTTGCCATTGTGGCTGCTCTTGCTATCTCGGCTCACGCACAAAGAGATTTCAATGAACGACGAGGAAAGGAGAATGACACAGAGAGAGGACAAGGTGGCTTTGGAGGAAGGCCTGGTGGAATGCAGATGGGTGGTC 186  
7-1515    ----TAGCATCGGAGAGACCTATTACTAACATGGAGGTGAAAGTGACACTGATCGTTGCCATTGTGGCTGCTCTTGCTATCTCGGCTCACGCACAAAGAGATTTCAATGAACGACGAGGAAAGGAGAATGACACAGAGAGAGGACAAGGTGGCTTTGGAGGAAGGCCTGGTGGAATGCAGATGGGTGGTC 186  
7-1516    ----TAGCATCGGAGAGACCTATTACTAACATGGAGGTGAAAGTGACACTGATCGTTGCCATTGTGGCTGCTCTTGCTATCTCGGCTCACGCACAAAGAGATTTCAATGAACGACGAGGAATGGAGAATGACACAGAGAGAGGACAAGGTGGCTTTGGAGGAAGGCCTGGTGGAATGCAGATGGGTGGTC 186  
7-1517    ------GCATCGGAGAGACCTATTACTAACATGGAGGTGAAAGTGACACTGATCGTTGCCATTGTGGCTGCTCTTGCTATCTCGGCTCACGCACAAAGAGATTTCAATGAACGACGAGGAAAGGAGAATGACACAGAGAGAGGACAAGGTGGCTTTGGAGGAAGGCCTGGTGGAATGCAGATGGGTGGTC 184  
7-1519    ----TAGCATCGGAGAGACCTATTACTAACATGGAGGTGAAAGTGACACTGATCGTTGCCATTGTGGCTGCTCTTGCTATCTCGGCTCACGCACAAAGAGATTTCAATGAACGACGAGGAAAGGAGAATGACACAGAGAGAGGACAAGGTGGCTTTGGAGGAAGGCCTGGTGGAATGCAGATGGGTGGTC 186  
7-1520    ----TAGCATCGGAGAGACCTATTACTAACATGGAGGTGAAAGTGACACTGATCGTTGCCATTGTGGCTGCTCTTGCTATCTCGGCTCACGCACAAAGAGATTTCAATGAACGACGAGGAAAGGAGAATGACACAGAGAGAGGACAAGGTGGCTTTGGAGGAAGGCCTGGTGGAATGCAGATGGGTGGTC 186  
7-1521    ----TAGCATCGGAGAGACCTATTACTAACATGGAGGTGAAAGTGACACTGATCGTTGCCATTGTGGCTGCTCTTGCTATCTCGGCTCACGCACAAAGAGATTTCAATGAACGACGAGGAAAGGAGAATGACACAGAGAGAGGACAAGGTGGCTTTGGAGGAAGGCCTGGTGGAATGCAGATGGGTGGTC 186  
7-1523    ----TAGCATCGGAGAGACCTATTACTAACATGGAGGTGAAAGTGACACTGATCGTTGCCATTGTGGCTGCTCTTGCTATCTCGGCTCACGCACAAAGAGATTTCAATGAACGACGAGGAAAGGAGAATGACACAGAGAGAGGACAAGGTGGCTTTGGAGGAAGGCCTGGTGGAATGCAGATGGGTGGTC 186  
7-1524    ----TAGCATCGGAGAGACCTATTACTAACATGGAGGTGAAAGTGACACTGATCGTTGCCATTGTGGCTGCTCTTGCTATCTCGGCTCACGCACAAAGAGATTTCAATGAACGACGAGGAAAGGAGAATGACACAGAGAGAGGACAAGGTGGCTTTGGAGGAAGGCCTGGTGGAATGCAGATGGGTGGTC 186  
7-1525    ----TAGCATCGGAGAGACCTATTACTAACATGGAGGTGAAAGTGACACTGATCGTTGCCATTGTGGCTGCTCTTGCTATCTCGGCTCACGCACAAAGAGATTTCAATGAACGACGAGGAAAGGAGAATGACACAGAGAGAGGACAAGGTGGCTTTGGAGGAAGGCCTGGTGGAATGCAGATGGGTGGTC 186  
7-1526    ----TAGCATCGGAGAGACCTATTACTAACATGGAGGTGAAAGTGACACTGATCGTTGCCATTGTGGCTGCTCTTGCTATCTCGGCTCACGCACAAAGAGATTTCAATGAACGACGAGGAAAGGAGAATGACACAGAGAGAGGACAAGGTGGCTTTGGAGGAAGGCCTGGTGGAATGCAGATGGGTGGTC 186  
7-1527    -----AGCATCGGAGAGACCTATTACTAACATGGAGGTGAAAGTGACACTGATCGTTGCCATTGTGGCTGCTCTTGCTATCTCGGCTCACGCACAAAGAGATTTCAATGAACGACGAGGAAAGGAGAATGACACAGAGAGAGGACAAGGTGGCTTTGGAGGAAGGCCTGGTGGAATGCAGATGGGTGGTC 185  
7-1528    ----TAGCATCGGAGAGACCTATTACTAACATGGAGGTGAAAGTGACACTGATCGTTGCCATTGTGGCTGCTCTTGCTATCTCGGCTCACGCACAAAGAGATTTCAATGAACGACGAGGAAAGGAGAATGACACAGAGAGAGGACAAGGTGGCTTTGGAGGAAGGCCTGGTGGAATGCAGATGGGTGGTC 186  
7-1529    ----TAGCATCGGAGAGACCTATTACTAACATGGAGGTGAAAGTGACACTGATCGTTGCCATTGTGGCTGCTCTTGCTATCTCGGCTCACGCACAAAGAGATTTCAATGAACGACGAGGAAAGGAGAATGACACAGAGAGAGGACAAGGTGGCTTTGGAGGAAGGCCTGGTGGAATGCAGATGGGTGGTC 186  
7-1530    ----TAGCATCGGAGAGACCTATTACTAACATGGAGGTGAAAGTGACACTGATCGTTGCCATTGTGGCTGCTCTTGCTATCTCGGCTCACGCACAAAGAGATTTCAATGAACGACGAGGAAAGGAGAATGACACAGAGAGAGGACAAGGTGGCTTTGGAGGAAGGCCTGGTGGAATGCAGATGGGTGGTC 186  
7-1533    ----TAGCATCGGAGAGACCTATTACTAACATGGAGGTGAAAGTGACACTGATCGTTGCCATTGTGGCTGCTCTTGCTATCTCGGCTCACGCACAAAGAGATTTCAATGAACGACGAGGAAAGGAGAATGACACAGAGAGAGGACAAGGTGGCTTTGGAGGAAGGCCTGGTGGAATGCAGATGGGTGGTC 186  
7-1534    ----TAGCATCGGAGAGACCTATTACTAACATGGAGGTGAAAGTGACACTGATCGTTGCCATTGTGGCTGCTCTTGCTATCTCGGCTCACGCACAAAGAGATTTCAATGAACGACGAGGAAAGGAGAATGACACAGAGAGAGGACAAGGTGGCTTTGGAGGAAGGCCTGGTGGAATGCAGATGGGTGGTC 186  
7-1536    ----TAGCATCGGAGAGACCTATTACTAACATGGAGGTGAAAGTGACACTGATCGTTGCCATTGTGGCTGCTCTTGCTATCTCGGCTCACGCACAAAGAGATTTCAATGAACGACGAGGAAAGGAGAATGACACAGAGAGAGGACAAGGTGGCTTTGGAGGAAGGCCTGGTGGAATGCAGATGGGTGGTC 186  
7-1537    ----TAGCATCGGAGAGACCTATTACTAACATGGAGGTGAAAGTGACACTGATCGTTGCCATTGTGGCTGCTCTTGCTATCTCGGCTCACGCACAAAGAGATTTCAATGAACGACGAGGAAAGGAGAATGACACAGAGAGAGGACAAGGTGGCTTTGGAGGAAGGCCTGGTGGAATGCAGATGGGTGGTC 186  
7-1538    ----TAGCATCGGAGAGACCTATTACTAACATGGAGGTGAAAGTGACACTGATCGTTGCCATTGTGGCTGCTCTTGCTATCTCGGCTCACGCACAAAGAGATTTCAATGAACGACGAGGAAAGGAGAATGACACAGAGAGAGGACAAGGTGGCTTTGGAGGAAGGCCTGGTGGAATGCAGATGGGTGGTC 186  
7-1542    ----TAGCATCGGAGAGACCTATTACTAACATGGAGGTGAAAGTGACACTGATCGTTGCCATTGTGGCTGCTCTTGCTATCTCGGCTCACGCACAAAGAGATTTCAATGAACGACGAGGAAAGGAGAATGACACAGAGAGAGGACAAGGTGGCTTTGGAGGAAGGCCTGGTGGAATGCAGATGGGTGGTC 186  
7-1544    ----TAGCATCGGAGAGACCTATTACTAACATGGAGGTGAAAGTGACACTGATCGTTGCCATTGTGGCTGCTCTTGCTATCTCGGCTCACGCACAAAGAGATTTCAATGAACGACGAGGAAAGGAGAATGACACAGAGAGAGGACAAGGTGGCTTTGGAGGAAGGCCTGGTGGAATGCAGATGGGTGGTC 186  
7-1545    ----TAGCATCGGAGAGACCTATTACTAACATGGAGGTGAAAGTGACACTGATCGTTGCCATTGTGGCTGCTCTTGCTATCTCGGCTCACGCACAAAGAGATTTCAATGAACGACGAGGAAAGGAGAATGACACAGAGAGAGGACAAGGTGGCTTTGGAGGAAGGCCTGGTGGAATGCAGATGGGTGGTC 186  
7-1546    ----TAGCATCGGAGAGACCTATTACTAACATGGAGGTGAAAGTGACACTGATCGTTGCCATTGTGGCTGCTCTTGCTATCTCGGCTCACGCACAAAGAGATTTCAATGAACGACGAGGAAAGGAGAATGACACAGAGAGAGGACAAGGTGGCTTTGGAGGAAGGCCTGGTGGAATGCAGATGGGTGGTC 186  
7-1548    ----TAGCATCGGAGAGACCTATTACTAACATGGAGGTGAAAGTGACACTGATCGTTGCCATTGTGGCTGCTCTTGCTATCTCGGCTCACGCACAAAGAGATTTCAATGAACGACGAGGAAAGGAGAATGACACAGAGAGAGGACAAGGTGGCTTTGGAGGAAGGCCTGGTGGAATGCAGATGGGTGGTC 186  
7-1549    ----TAGCATCGGAGAGACCTATTACTAACATGGAGGTGAAAGTGACACTGATCGTTGCCATTGTGGCTGCTCTTGCTATCTCGGCTCACGCACAAAGAGATTTCAATGAACGACGAGGAAAGGAGAATGACACAGAGAGAGGACAAGGTGGCTTTGGAGGAAGGCCTGGTGGAATGCAGATGGGTGGTC 186  
7-1550    ----TAGCATCGGAGAGACCTATTACTAACATGGAGGTGAAAGTGACACTGATCGTTGCCATTGTGGCTGCTCTTGCTATCTCGGCTCACGCACAAAGAGATTTCAATGAGCGACGAGGAAAGGAGAATGACACAGAGAGAGGACAAGGTGGCTTTGGAGGAAGGCCTGGTGGAATGCAGATGGGTGGTC 186  
7-1539    ----TAGCATCGGAGAGACCTATTACTAACATGGAGGTGAAAGTGACACTGATCGTTGCCATTGTGGCTGCTCTTGCTATCTCGGCTCACGCACAAAGAGATTTCAATGAACGACGAGGAAAGGAGAATGACACAGAGAGAGGACAAGGTGGCTTTGGAGGAAGGCCTGGTGGAATGCAGATGGGTGGTC 186  
7-1540    ----TAGCATCGGAGAGACCTATTACTAACATGGAGGTGAAAGTGACACTGATCGTTGCCATTGTGGCTGCTCTTGCTATCTCGGCTCACGCACAAAGAGATTTCAATGAACGACGAGGAAAGGAGAATGACACAGAGAGAGGACAAGGTGGCTTTGGAGGAAGGCCTGGTGGAATGCAGATGGGTGGTC 186  
7-1514    ------TAGCATCGGAGAGACCTTACAAACATGGAGGTGAAAGTGACACTGATCGTTGCCATTGTGGCTGCTCTTGCTATCTCGGCTCACGCACAAAGAGATTTCAATGAACGACGAGGAAAGGAGAATGACACAGAGAGAGGACAAGGTGGCTTTGGAGGAAGGCCGGGTGGAATGCAGATGGGTGGTC 184  
7-1507    ----TAGCATCGGAGAGACCTATTACTAACATGGAGGTGAAAGTGACACTGATCGTTGCCATTGTGGCTGCTCTTGCTATCTCGGCTCACGCACAAAGAGATTTCAATGAACGACGAGGAAAGGAGAATGACACAGAGAGAGGACAAGGTGGCTTTGGAGGAAGGCCTGGTGGAATGCAGATGGGTGGTC 186  
7-1518    ------TAGCATCGGAGAGACCTTACAAACATGGAGGTGAAAGTGACACTGATCGTTGCCATTGTGGCTGCTCTTGCTATCTCGGCTCACGCACAAAGAGATTTCAATGAACGACGAGGAAAGGAGAATGACACAGAGAGAGGACAAGGTGGCTTTGGAGGAAGGCCGGGTGGAATGCAGATGGGTGGTC 184  
7-1522    ------TAGCATCGGAGAGACCTTACAAACATGGAGGTGAAAGTGACACTGATCGTTGCCATTGTGGCTGCTCTTGCTATCTCGGCTCACGCACAAAGAGATTTCAATGAACGACGAGGAAAGGAGAATGACACAGAGAGAGGACAAGGTGGCTTTGGAGGAAGGCCGGGTGGAATGCAGATGGGTGGTC 184  
7-1532    ----TAGCATCGGAGAGACCTATTACTAACATGGAGGTGAAAGTGACACTGATCGTTGCCATTGTGGCTGCTCTTGCTATCTCGGCTCACGCACAAAGAGATTTCAATGAACGACGAGGAAAGGAGAATGACACAGAGAGAGGACAAGGTGGCTTTGGAGGAAGGCCTGGTGGAATGCAGATGGGTGGTC 186  
7-1543    ------TAGCATCGGAGAGACCTTACAAACATGGAGGTGAAAGTGACACTGATCGTTGCCATTGTGGCTGCTCTTGCTATCTCGGCTCACGCACAAAGAGATTTCAATGAACGACGAGGAAAGGAGAATGACACAGAGAGAGGACAAGGTGGCTTTGGAGGAAGGCCGGGTGGAATGCAGATGGGTGGTC 184  
7-1547    ----TAGCATCGGAGAGACCTATTACTAACATGGAGGTGAAAGTGACACTGATCGTTGCCATTGTGGCTGCTCTTGCTATCTCGGCTCACGCACAAAGAGATTTCAATGAACGACGAGGAAAGGAGAATGACACAGAGAGAGGACAAGGTGGCTTTGGAGGAAGGCCTGGTGGAATGCAGATGGGTGGTC 186  
7-2401    ----TAGCATCGGAGAGACCTATTACTAACATGGAGGTGAAAGTGACACTGATCGTTGCCATTGTGGCTGCTCTTGCTATCTCGGCTCACGCACAAAGAGATTTCAATGAACGACGAGGAAAGGAGAATGACACAGAGAGAGGACAAGGTGGCTTTGGAGGAAGGCCTGGTGGAATGCAGATGGGTGGTC 186  
7-2402    ----TAGCATCGGAGAGACCTATTACTAACATGGAGGTGAAAGTGACACTGATCGTTGCCATTGTGGCTGCTCTTGCTATCTCGGCTCACGCACAAAGAGATTTCAATGAACGACGAGGAAAGGAGAATGACACAGAGAGAGGACAAGGTGGCTTTGGAGGAAGGCCTGGTGGAATGCAGATGGGTGGTC 186  
7-2403    ------GCATCGGAGAGACCTATTACTAACATGGAGGTGAAAGTGACACTGATCGTTGCCATTGTGGCTGCTCTTGCTATCTCGGCTCACGCACAAAGAGATTTCAATGAACGACGAGGAAAGGAGAATGACACAGAGAGAGGACAAGGTGGCTTTGGAGGAAGGCCTGGTGGAATGCAGATGGGTGGTC 184  
7-2404    ----TAGCATCGGAGAGACCTATTACTAACATGGAGGTGAAAGTGACACTGATCGTTGCCATTGTGGCTGCTCTTGCTATCTCGGCTCACGCACAAAGAGATTTCAATGAACGACGAGGAAAGGAGAATGACACAGAGAGAGGACAAGGTGGCTTTGGAGGAAGGCCTGGTGGAATGCAGATGGGTGGTC 186  
7-2405    ----TAGCATCGGAGAGACCTATTACTAACATGGAGGTGAAAGTGACACTGATCGTTGCCATTGTGGCTGCTCTTGCTATCTCGGCTCACGCACAAAGAGATTTCAATGAACGACGAGGAAAGGAGAATGACACAGAGAGAGGACAAGGTGGCTTTGGAGGAAGGCCTGGTGGAATGCAGATGGGTGGTC 186  
7-2406    ----TAGCATCGGAGAGACCTATTACTAACATGGAGGTGAAAGTGACACTGATCGTTGCCATTGTGGCTGCTCTTGCTATCTCGGCTCACGCACAAAGAGATTTCAATGAACGACGAGGAAAGGAGAATGACACAGAGAGAGGACAAGGTGGCTTTGGAGGAAGGCCTGGTGGAATGCAGATGGGTGGTC 186  
7-2407    ----TAGCATCGGAGAGACCTATTACTAACATGGAGGTGAAAGTGACACTGATCGTTGCCATTGTGGCTGCTCTTGCTATCTCGGCTCACGCACAAAGAGATTTCAATGAACGACGAGGAAAGGAGAATGACACAGAGAGAGGACAAGGTGGCTTTGGAGGAAGGCCTGGTGGAATGCAGATGGGTGGTC 186  
7-2408    ----TAGTCTCGGAGAGACCTATTACTAACATGGAGGTGAAAGTGACACTGATCGTTGCCATTGTGGCTGCTCTTGCTATCTCGGCTCACGCACAAAGAGATTTCAATGAACGACGAGGAAAGGAGAATGACACAGAGAGAGGACAAGGTGGCTTTGGAGGAAGGCCTGGTGGAATGCAGATGGGTGGTC 186  
7-2409    ----TAGCATCGGAGAGACCTATTACTAACATGGAGGTGAAAGTGACACTGATCGTTGCCATTGTGGCTGCTCTTGCTATCTCGGCTCACGCACAAAGAGATTTCAATGAACGACGAGGAAAGGAGAATGACACAGAGAGAGGACAAGGTGGCTTTGGAGGAAGGCCTGGTGGAATGCAGATGGGTGGTC 186  
7-2410    ----TAGCATCGGAGAGACCTATTACTAACATGGAGGTGAAAGTGACACTGATCGTTGCCATTGTGGCTGCTCTTGCTATCTCGGCTCACGCACAAAGAGATTTCAATGAACGACGAGGAAAGGAGAATGACACAGAGAGAGGACAAGGTGGCTTTGGAGGAAGGCCTGGTGGAATGCAGATGGGTGGTC 186  
7-2411    ----TAGCATCGGAGAGACCTATTACTAACATGGAGGTGAAAGTGACACTGATCGTTGCCATTGTGGCTGCTCTTGCTATCTCAGCTCACGCACAAAGAGATTTCAATGAACGACGAGGAAAGGAGAATGACACAGAGAGAGGACAAGGTGGCTTTGGAGGAAGGCCTGGTGGAATGCAGATGGGTGGTC 186  
7-2412    ----TAGCATCGGAGAGACCTATTACTAACATGGAGGTGAAAGTGACACTGATCGTTGCCATTGTGGCTGCTCTTGCTATCTCGGCTCACGCACAAAGAGATTTCAATGAACGACGAGGAAAGGAGAATGACACAGAGAGAGGACAAGGTGGCTTTGGAGGAAGGCCTGGTGGAATGCAGATGGGTGGTC 186  
7-2413    ----TAGCATCGGAGAGACCTATTACTAACATGGAGGTGAAAGTGACACTGATCGTTGCCATTGTGGCTGCTCTTGCTATCTCGGCTCACGCACAAAGAGATTTCAATGAACGACGAGGAAAGGAGAATGACACAGAGAGAGGACAAGGTGGCTTTGGAGGAAGGCCTGGTGGAATGCAGATGGGTGGTC 186  
7-2414    ----TAGCATCGGAGAGACCTATTACTAACATGGAGGTGAAAGTGACACTGATCGTTGCCATTGTGGCTGCTCTTGCTATCTCGGCTCACGCACAAAGAGATTTCAATGAACGACGAGGAAAGGAGAATGACACAGAGAGAGGACAAGGTGGCTTTGGAGGAAGGCCTGGTGGAATGCAGATGGGTGGTC 186  
7-2416    ----TAGCATCGGAGAGACCTATTACTAACATGGAGGTGAAAGTGACACTGATCGTTGCCATTGTGGCTGCTCTTGCTATCTCGGCTCACGCACAAAGAGATTTCAATGAACGACGAGGAAAGGAGAATGACACAGAGAGAGGACAAGGTGGCTTTGGAGGAAGGCCTGGTGGAATGCAGATGGGTGGTC 186  
7-2417    ----TAGCATCGGAGAGACCTATTACTAACATGGAGGTGAAAGTGACACTGATCGTTGCCATTGTGGCTGCTCTTGCTATCTCGGCTCACGCACAAAGAGATTTCAATGAACGACGAGGAAAGGAGAATGACACAGAGAGAGGACAAGGTGGCTTTGGAGGAAGGCCTGGTGGAATGCAGATGGGTGGTC 186  
7-2418    ----TAGCATCGGAGAGACCTATTACTAACATGGAGGTGAAAGTGACACTGATCGTTGCCATTGTGGCTGCTCTTGCTATCTCGGCTCACGCACAAAGAGATTTCAATGAACGACGAGGAAAGGAGAATGACACAGAGAGAGGACAAGGTGGCTTTGGAGGAAGGCCTGGTGGAATGCAGATGGGTGGTC 186  
7-2420    ----TAGCATCGGAGAGACCTATTACTAACATGGAGGTGAAAGTGACACTGATCGTTGCCATTGTGGCTGCTCTTGCTATCTCGGCTCACGCACAAAGAGATTTCAATGAACGACGAGGAAAGGAGAATGACACAGAGAGAGGACAAGGTGGCTTTGGAGGAAGGCCTGGTGGAATGCAGATGGGTGGTC 186  
7-2421    ----TAGCATCGGAGAGACCTATTACTAACATGGAGGTGAAAGTGACACTGATCGTTGCCATTGTGGCTGCTCTTGCTATCTCGGCTCACGCACAAAGAGATTTCAATGAACGACGAGGAAAGGAGAATGACACAGAGAGAGGACAAGGTGGCTTTGGAGGAAGGCCTGGTGGAATGCAGATGGGTGGTC 186  
7-2423    ----TAGCATCGGAGAGACCTATTACTAACATGGAGGTGAAAGTGACACTGATCGTTGCCATTGTGGCTGCTCTTGCTATCTCGGCTCACGCACAAAGAGATTTCAATGAACGACGAGGAAAGGAGAATGACACAGAGAGAGGACAAGGTGGCTTTGGAGGAAGGCCTGGTGGAATGCAGATGGGTGGTC 186  
7-2424    ----TAGCATCGGAGAGACCTATTACTAACATGGAGGTGAAAGTGACACTGATCGTTGCCATTGTGGCTGCTCTTGCTATCTCGGCTCACGCACAAAGAGATTTCAATGAACGACGAGGAAAGGAGAATGACACAGAGAGAGGACAAGGTGGCTTTGGAGGAAGGCCTGGTGGAATGCAGATGGGTGGTC 186  
7-2425    ----TAGCATCGGAGAGACCTATTACTAACATGGAGGTGAAAGTGACACTGATCGTTGCCATTGTGGCTGCTCTTGCTATCTCGGCTCACGCACAAAGAGATTTCAATGAACGACGAGGAAAGGAGAATGACACAGAGAGAGGACAAGGTGGCTTTGGAGGAAGGCCTGGTGGAATGCAGATGGGTGGTC 186  
7-2426    ----TAGCATCGGAGAGACCTATTACTAACATGGAGGTGAAAGTGACACTGATCGTTGCCATTGTGGCTGCTCTTGCTATCTCGGCTCACGCACAAAGAGATTTCAATGAACGACGAGGAAAGGAGAATGACACAGAGAGAGGACAAGGTGGCTTTGGAGGAAGGCCTGGTGGAATGCAGATGGGTGGTC 186  
7-2430    ----TAGCATCGGAGAGACCTATTACTAACATGGAGGTGATAGTGACACTGATCGTTGCCATTGTGGCTGCTCTTGCTATCTCGGCTCACGCACAAAGAGATTTCAATGAACGACGAGGAAAGGAGAATGACACAGAGAGAGGACAAGGTGGCTTTGGAGGAAGGCCTGGTGGAATGCAGATGGGTGGTC 186  
7-2431    ----TAGCATCGGAGAGACCTATTACTAACATGGAGGTGAAAGTGACACTGATCGTTGCCATTGTGGCTGCTCTTGCTATCTCGGCTCACGCACAAAGAGATTTCAATGAACGACGAGGAAAGGAGAATGACACAGAGAGAGGACAAGGTGGCTTTGGAGGAAGGCCTGGTGGAATGCAGATGGGTGGTC 186  
7-2432    ----TAGCATCGGAGAGACCTATTACTAACATGGAGGTGAAAGTGACACTGATCGTTGCCATTGTGGCTGCTCTTGCTATCTCGGCTCACGCACAAAGAGATTTCAATGAACGACGAGGAAAGGAGAATGACACAGAGAGAGGACAAGGTGGCTTTGGAGGAAGGCCTGGTGGAATGCAGATGGGTGGTC 186  
7-2436    ----TAGCATCGGAGAGACCTATTACTAACATGGAGGTGAAAGTGACACTGATCGTTGCCATTGTGGCTGCTCTTGCTATCTCGGCTCACGCACAAAGAGATTTCAATGAACGACGAGGAAAGGAGAATGACACAGAGAGAGGACAAGGTGGCTTTGGAGGAAGGCCTGGTGGAATGCAGATGGGTGGTC 186  
7-2437    ----TAGCATCGGAGAGACCTATTACTAACATGGAGGTGAAAGTGACACTGATCGTTGCCATTGTGGCTGCTCTTGCTATCTCGGCTCACGCACAAAGAGATTTCAATGAACGACGAGGAAAGGAGAATGACACAGAGAGAGGACAAGGTGGCTTTGGAGGAAGGCCTGGTGGAATGCAGATGGGTGGTC 186  
7-2439    ----TAGCATCGGAGAGACCTATTACTAACATGGAGGTGAAAGTGACACTGATCGTTGCCATTGTGGCTGCTCTTGCTATCTCGGCTCACGCACAAAGAGATTTCAATGAACGACGAGGAAAGGAGAATGACACAGAGAGAGGACAAGGTGGCTTTGGAGGAAGGCCTGGTGGAATGCAGATGGGTGGTC 186  
7-2442    ----TAGCATCGGAGAGACCTATTACTAACATGGAGGTGAAAGTGACACTGATCGTTGCCATTGTGGCTGCTCTTGCTATCTCGGCTCACGCACAAAGAGATTTCAATGAACGACGAGGAAAGGAGAATGACACAGAGAGAGGACAAGGTGGCTTTGGAGGAAGGCCTGGTGGAATGCAGATGGGTGGTC 186  
7-2443    ----TAGCATCGGAGAGACCTATTACTAACATGGAGGTGAAAGTGACACTGATCGTTGCCATTGTGGCTGCTCTTGCTATCTCGGCTCACGCACAAAGAGATTTCAATGAACGACGAGGAAAGGAGAATGACACAGAGAGAGGACAAGGTGGCTTTGGAGGAAGGCCTGGTGGAATGCAGATGGGTGGTC 186  
7-2444    ----TAGCATCGGAGAGACCTATTACTAACATGGAGGTGAAAGTGACACTGATCGTTGCCATTGTGGCTGCTCTTGCTATCTCGGCTCACGCACAAAGAGATTTCAATGAACGACGAGGAAAGGAGAATGACACAGAGAGAGGACAAGGTGGCTTTGGAGGAAGGCCTGGTGGAATGCAGATGGGTGGTC 186  
7-2445    ----TAGCATCGGAGAGACCTATTACTAACATGGAGGTGAAAGTGACACTGATCGTTGCCATTGTGGCTGCTCTTGCTATCTCGGCTCACGCACAAAGAGATTTCAATGAACGACGAGGAAAGGAGAATGACACAGAGAGAGGACAAGGTGGCTTTGGAGGAAGGCCTGGTGGAATGCAGATGGGTGGTC 186  
7-2446    ----TAGCATCGGAGAGACCTATTACTAACATGGAGGTGAAAGTGACACTGATCGTTGCCATTGTGGCTGCTCTTGCTATCTCGGCTCACGCACAAAGAGATTTCAATGAGCGACGAGGAAAGGAGAATGACACAGAGAGAGGACAAGGTGGCTTTGGAGGAAGGCCTGGTGGAATGCAGATGGGTGGTC 186  
7-2447    ----TAGCATCGGAGAGACCTATTACTAACATGGAGGTGAAAGTGACACTGATCGTTGCCATTGTGGCTGCTCTTGCTATCTCGGCTCACGCACAAAGAGATTTCAATGAACGACGAGGAAAGGAGAATGACACAGAGAGAGGACAAGGTGGCTTTGGAGGAAGGCCTGGTGGAATGCAGATGGGTGGTC 186  
7-2448    ----TAGCATCGGAGAGACCTATTACTAACATGGAGGTGAAAGTGACACTGATCGTTGCCATTGTGGCTGCTCTTGCTATCTCGGCTCACGCACAAAGAGATTTCAATGAACGACGAGGAAAGGAGAATGACACAGAGAGAGGACAAGGTGGCTTTGGAGGAAGGCCTGGTGGAATGCAGATGGGTGGTC 186  
7-2450    ----TAGCATCGGAGAGACCTATTACTAACATGGAGGTGAAAGTGACACTGATCGTTGCCATTGTGGCTGCTCTTGCTATCTCGGCTCACGCACAAAGAGATTTCAATGAACGACGAGGAAAGGAGAATGACACAGAGAGAGGACAAGGTGGCTTTGGAGGAAGGCCTGGTGGAATGCAGATGGGTGGTC 186  
7-2415    ----TAGCATCGGAGAGACCTATTACTAACATGGAGGTGAAAGTGACACTGATCGTTGCCATTGTGGCTGCTCTTGCTATCTCGGCTCACGCACAAAGAGATTTCAATGAACGACGAGGAAAGGAGAATGACACAGAGAGAGGACAAGGTGGCTTTGGAGGAAGGCCTGGTGGAATGCAGATGGGTGGTC 186  
7-2427    ----TAGCATCGGAGAGACCTATTACTAACATGGAGGTGAAAGTGACACTGATCGTTGCCATTGTGGCTGCTCTTGCTATCTCGGCTCACGCACAAAGAGATTTCAATGAACGACGAGGAAAGGAGAATGACACAGAGAGAGGACAAGGTGGCTTTGGAGGAAGGCCTGGTGGAATGCAGATGGGTGGTC 186  
7-2428    ------TAGCATCGGAGAGACCTTACTAACATGGAGGTGAAAGTGACACTGATCGTTGCCATTGTGGCTGCTCTTGCTATCTCGGCTCACGCACAAAGAGATTTCAATGAACGACGAGGAAAGGAGAATGACACAGAGAGAGGACAAGGTGGCTTTGGAGGAAGGCCTGGTGGAATGCAGATGGGTGGTC 184  
7-2435    ------TAGCATCGGAGAGACCTTACAAACATGGAGGTGAAAGTGACACTGATCGTTGCCATTGTGGCTGCTCTTGCTATCTCGGCCCACGCACAAAGAGATTTCAATGAACGACGGGGAAAGGAGAATGACACAGAGAGAGGACAAGGTGGCTTTGGAGGAAGGCCGGGTGGAATGCAGATGGGTGGTC 184  
7-2440    ----TAGCATCGGAGAGACCTATTACTAACATGGAGGTGAAAGTGACACTGATCGTTGCCATTGTGGCTGCTCTTGCTATCTCGGCTCACGCACAAAGAGATTTCAATGAACGACGAGGAAAGGAGAATGACACAGAGAGAGGACAAGGTGGCTTTGGAGGAAGGCCTGGTGGAATGCAGATGGGTGGTC 186  
7-2441    ------TAGCATCGGAGAGACCTTACAAACATGGAGGTGAAAGTGACACTGATCGTTGCCATTGTGGCTGCTCTTGCTATCTCGGCTCACGCACAAAGAGATTTCAATGAACGACGAGGAAAGGAGAATGACACAGAGAGAGGACAAGGTGGCTTTGGAGGAAGGCCGGGTGGAATGCAGATGGGTGGTC 184  
7-2449    ------TAGCATCGGAGAGACCTTACAAACATGGAGGTGAAAGTGACACTGATCGTTGCCATTGTGGCTGCTCTTGCTATCTCGGCTCACGCACAAAGAGATTTCAATGAACGACGAGGAAAGGAGAATGACACAGAGAGAGGACAAGGTGGCTTTGGAGGAAGGCCGGGTGGAATGCAGATGGGTGGTC 184  
8-1501    ----TAGCATCGGAGAGACCTATTACTAACATGGAGGTGAAAGTGACACTGATCGTTGCCATTGTGGCTGCTCTTGCTATCTCGGCTCACGCACAAAGAGATTTCAATGAACGACGAGGAAAGGAGAATGACACAGAGAGAGGACAAGGTGGCTTTGGAGGAAGGCCTGGTGGAATGCAGATGGGTGGTC 186  
8-1502    ----TAGCATCGGAGAGACCTATTACTAACATGGAGGTGAAAGTGACACTGATCGTTGCCATTGTGGCTGCTCTTGCTATCTCGGCTCACGCACAAAGAGATTTCAATGAACGACGAGGAAAGGAGAATGACACAGAGAGAGGACAAGGTGGCTTTGGAGGAAGGCCTGGTGGAATGCAGATGGGTGGTC 186  
8-1503    -----AGCATCGGAGAGACCTATTACTAACATGGAGGTGAAAGTGACACTGATCGTTGCCATTGTGGCTGCTCTTGCTATCTCGGCTCACGCACAAAGAGATTTCAATGAACGACGAGGAAAGGAGAATGACACAGAGAGAGGACAAGGTGGCTTTGGAGGAAGGCCTGGTGGAATGCAGATGGGTGGTC 185  
8-1504    ----TAGCATCGGAGAGACCTATTACTAACATGGAGGTGAAAGTGACACTGATCGTTGCCATTGTGGCTGCTCTTGCTATCTCGGCTCACGCACAAAGAGATTTCAATGAACGACGAGGAAAGGAGAATGACACAGAGAGAGGACAAGGTGGCTTTGGAGGAAGGCCTGGTGGAATGCAGATGGGTGGTC 186  
8-1505    ----TAGCATCGGAGAGACCTATTACTAACATGGAGGTGAAAGTGACACTGATCGTTGCCATTGTGGCTGCTCTTGCTATCTCGGCTCACGCACAAAGAGATTTCAATGAACGACGAGGAAAGGAGAATGACACAGAGAGAGGACAAGGTGGCTTTGGAGGAAGGCCTGGTGGAATGCAGATGGGTGGTC 186  
8-1506    ----TAGCATCGGAGAGACCTATTACTAACATGGAGGTGAAAGTGACACTGATCGTTGCCATTGTGGCTGCTCTTGCTATCTCGGCTCACGCACAAAGAGATTTCAATGAACGACGAGGAAAGGAGAATGACACAGAGAGAGGACAAGGTGGCTTTGGAGGAAGGCCTGGTGGAATGCAGATGGGTGGTC 186  
8-1507    ----TAGCATCGGAGAGACCTATTACTAACATGGAGGTGAAAGTGACGCTGATCGTTGCCATTGTGGCTGCTCTTGCTATCTCGGCTCACGCACAAAGAGATTTCAATGAACGACGAGGAAAGGAGAATGACACAGAGAGAGGACAAGGTGGCTTTGGAGGAAGGCCTGGTGGAGTGCAGATGGGTGGTC 186  
8-1508    ----TAGCATCGGAGAGACCTATTACTAACATGGAGGTGAAAGTGACACTGATCGTTGCCATTGTGGCTGCTCTTGCTATCTCGGCTCACGCACAAAGAGATTTCAATGAACGACGAGGAAAGGAGAATGACACAGAGAGAGGACAAGGTGGCTTTGGAGGAAGGCCTGGTGGAATGCAGACGGGTGGTC 186  
8-1509    ----TAGCATCGGAGAGACCTATTACTAACATGGAGGTGAAAGTGACACTGATCGTTGCCATTGTGGCTGCTCTTGCTATCTCGGCTCACGCACAAAGAGATTTCAATGAACGACGAGGAAAGGAGAATGACACAGAGAGAGGACAAGGTGGCTTTGGAGGAAGGCCTGGTGGAATGCAGATGGGTGGTC 186  
8-1510    ----TAGCATCGGAGAGACCTATTACTAACATGGAGGTGAAAGTGACACTGATCGTTGCCATTGTGGCTGCTCTTGCTATCTCGGCTCACGCACAAAGAGATTTCAATGAACGACGAGGAAAGGAGAATGACACAGAGAGAGGACAAGGTGGCTTTGGAGGAAGGCCTGGTGGAATGCAGATGGGTGGTC 186  
8-1511    ----TAGCATCGGAGAGACCTATTACTAACATGGAGGTGAAAGTGACACTGATCGTTGCCATTGTGGCTGCTCTTGCTATCTCGGCTCACGCACAAAGAGATTTCAATGAACGACGAGGAAAGGAGAATGACACAGAGAGAGGACAAGGTGGCTTTGGAGGAAGGCCTGGTGGAATGCAGATGGGTGGTC 186  
8-1512    ----TAGCATCGGAGAGACCTATTACTAACATGGAGGTGAAAGTGACACTGATCGTTGCCATTGTGGCTGCTCTTGCTATCTCGGCTCACGCACAAAGAGATTTCAATGAACGACGAGGAAAGGAGAATGACACAGAGAGAGGACAAGGTGGCTTTGGAGGAAGGCCTGGTGGAATGCAGATGGGTGGTC 186  
8-1513    ----TAGCATCGGAGAGACCTATTACTAACATGGAGGTGAAAGTGACACTGATCGTTGCCATTGTGGCTGCTCTTGCTATCTCGGCTCACGCACAAAGAGATTTCAATGAACGACGAGGAAAGGAGAATGACACAGAGAGAGGACAAGGTGGCTTTGGAGGAAGGCCTGGTGGAATGCAGATGGGTGGTC 186  
8-1514    ----TAGCATCGGAGAGACCTATTACTAACATGGAGGTGAAAGTGACACTGATCGTTGCCATTGTGGCTGCTCTTGCTATCTCGGCTCACGCACAAAGAGATTTCAATGAACGACGAGGAAAGGAGAATGACACAGAGAGAGGACAAGGTGGCTTTGGAGGAAGGCCTGGTGGAATGCAGATGGGTGGTC 186  
8-1515    ----TAGCATCGGAGAGACCTATTACTAACATGGAGGTGAAAGTGACACTGATCGTTGCCATTGTGGCTGCTCTTGCTATCTCGGCTCACGCACAAAGAGATTTCAATGAACGACGAGGAAAGGAGAATGACACAGAGAGAGGACAAGGTGGCTTTGGAGGAAGGCCTGGTGGAATGCAGATGGGTGGTC 186  
8-1516    ----TAGCATCGGAGAGACCTATTACTAACATGGAGGTGAAAGTGACACTGATCGTTGCCATTGTGGCTGCTCTTGCTATCTCGGCTCACGCACAAAGAGATTTCAATGAACGACGAGAAAAGGAGAATGACACAGAGAGAGGACAAGGTGGCTTTGGAGGAAGGCCTGGTGGAATGCAGATGGGTGGTC 186  
8-1517    ----TAGCATCGGAGAGACCTATTACTAACATGGAGGTGAAAGTGACACTGATCGTTGCCATTGTGGCTGCTCTTGCTATCTCGGCTCACGCACAAAGAGATTTCAATGAACGACGAGGAAAGGAGAATGACACAGAGAGAGGACAAGGTGGCTTTGGAGGAAGGCCTGGTGGAATGCAGATGGGTGGTC 186  
8-1518    ----TAGCATCGGAGAGACCTATTACTAACATGGAGGTGAAAGTGACACTGATCGTTGCCATTGTGGCTGCTCTTGCTATCTCGGCTCACGCACAAAGAGATTTCAATGAACGACGAGGAAAGGAGAATGACACAGAGAGAGGACAAGGTGGCTTTGGAGGAAGGCCTGGTGGAATGCAGATGGGTGGTC 186  
8-1519    ----TAGCATCGGAGAGACCTATTACTAACATGGAGGTGAAAGTGACACTGATCGTTGCCATTGTGGCTGCTCTTGCTATCTCGGCTCACGCACAAAGAGATTTCAATGAACGACGAGGAAAGGAGAACGACACAGAGAGAGGACAAGGTGGCTTTGGAGGAAGGCCTGGTGGAATGCAGATGGGTGGTC 186  
8-1520    ----TAGCATCGGAGAGACCTATTACTAACATGGAGGTGAAAGTGACACTGATCGTTGCCATTGTGGCTGCTCTTGCTATCTCGGCTCACGCACAAAGAGATTTCAATGAACGACGAGGAAAGGAGAATGACACAGAGAGAGGACAAGGTGGCTTTGGAGGAAGGCCTGGTGGAATGCAGATGGGTGGTC 186  
8-1521    ----TAGCATCGGAGAGACCTATTACTAACATGGAGGTGAAAGTGACACTGATCGTTGCCATTGTGGCTGCTCTTGCTATCTCGGCTCACGCACAAAGAGATTTCAATGAACGACGAGGAAAGGAGAATGACACAGAGAGAGGACAAGGTGGCTTTGGAGGAAGGCCTGGTGGAATGCAGATGGGTGGTC 186  
8-1522    ----TAGCATCGGAGAGACCTATTACTAACATGGAGGTGAAAGTGACACTGATCGTTGCCATTGTGGCTGCTCTTGCTATCTCGGCTCACGCACAAAGAGATTTCAATGAACGACGAGGAAAGGAGAATGACACAGAGAGAGGACAAGGTGGCTTTGGAGGAAGGCCTGGTGGAATGCAGATGGGTGGTC 186  
8-1524    ----TAGCATCGGAGAGACCTATTACTAACATGGAGGTGAAAGTGACACTGATCGTTGCCATTGTGGCTGCTCTTGCTATCTCGGCTCACGCACAAAGAGATTTCAATGAACGACGAGGAAAGGAGAATGACACAGAGAGAGGACAAGGTGGCTTTGGAGGAAGGCCTGGTGGAATGCAGATGGGTGGTC 186  
8-1525    ----TAGCATCGGAGAGACCTATTACTAACATGGAGGTGAAAGTGACACTGATCGTTGCCATTGTGGCTGCTCTTGCTATCTCGGCTCACGCACAAAGAGATTTCAATGAACGACGAGGAAAGGAGAATGACACAGAGAGAGGACAAGGTGGCTTTGGAGGAAGGCCTGGTGGAATGCAGATGGGTGGTC 186  
8-1526    ----TAGCATCGGAGAGACCTATTACTAACATGGAGGTGAAAGTGACACTGATCGTTGCCATTGTGGCTGCTCTTGCTATCTCGGCTCACGCACAAAGAGATTTCAATGAACGACGAGGAAAGGAGAATGACACAGAGAGAGGACAAGGTGGCTTTGGAGGAAGGCCTGGTGGAATGCAGATGGGTGGTC 186  
8-1527    ----TAGCATCGGAGAGACCTATTACTAACATGGAGGTGAAAGTGACACTGATCGTTGCCATTGTGGCTGCTCTTGCTATCTCGGCTCACGCACAAAGAGATTTCAATGAACGACGAGGAAAGGAGAATGACACAGAGAGAGGACAAGGTGGCTTTGGAGGAAGGCCTGGTGGAATGCAGATGGGTGGTC 186  
8-1528    ----TAGCATCGGAGAGACCTATTACTAACATGGAGGTGAAAGTGACACTGATCGTTGCCATTGTGGCTGCTCTTGCTATCTCGGCTCACGCACAAAGAGATTTCAATGAACGACGAGGAAAGGAGAATGACACAGAGAGAGGACAAGGTGGCTTTGGAGGGAGGCCTGGTGGAATGCAGATGGGTGGTC 186  
8-1529    ----TAGCATCGGAGAGACCTATTACTAACATGGAGGTGAAAGTGACACTGATCGTTGCCATTGTGGCTGCTCTTGCTATCTCGGCTCACGCACAAAGAGATTTCAATGAACGACGAGGAAAGGAGAATGACACAGAGAGAGGACAAGGTGGCTTTGGAGGAAGGCCTGGTGGAATGCAGATGGGTGGTC 186  
8-1530    ----TAGCATCGGAGAGACCTATTACTAACATGGAGGTGAAGGTGACACTGATCGTTGCCATTGTGGCTGCTCTTGCTATCTCGGCTCACGCACAAAGAGATTTCAATGAACGACGAGGAAAGGAGAATGACACAGAGAGAGGACAAGGTGGCTTTGGAGGAAGGCCTGGTGGAATGCAGATGGGTGGTC 186  
8-1531    ----TAGCATCGGAGAGACCTATTACTAACATGGAGGTGAAAGTGACACTGATCGTTGCCATTGTGGCTGCTCTTGCTATCTCGGCTCACGCACAAAGAGGTTTCAATGAACGACGAGGAAAGGAGAATGACACAGAGAGAGGACAAGGTGGCTTTGGAGGAAGGCCTGGTGGAATGCAGATGGGTGGTC 186  
8-1532    ----TAGCATCGGAGAGACCTATTACTAACATGGAGGTGAAAGTGACACTGATCGTTGCCATTGTGGCTGCTCTTGCTATCTCGGCTCACGCACAAAGAGATTTCAATGAACGACGAGGAAAGGAGAATGACACAGAGAGAGGACAAGGTGGCTTTGGAGGAAGGCCTGGTGGAATGCAGATGGGTGGTC 186  
8-1533    ----TAGCATCGGAGAGACCTATTACTAACATGGAGGTGAAAGTGACACTGATCGTTGCCATTGTGGCTGCTCTTGCTATCTCGGCTCACGCACAAAGAGATTTCAATGAACGACGAGGAAAGGAGAATGACACAGAGAGAGGACGAGGTGGCTTTGGAGGAAGGCCTGGTGGAATGCAGATGGGTGGTC 186  
8-1534    ----TAGCATCGGAGAGACCTATTACTAACATGGAGGTGAAAGTGACACTGATCGTTGCCATTGTGGCTGCTCTTGCTATCTCGGCTCACGCACAAAGAGATTTCAATGAACGACGAGGAAAGGAGAATGACACAGAGAGAGGACAAGGTGGCTTTGGAGGAAGGCCTGGTGGAATGCAGATGGGTGGTC 186  
8-1535    ----TAGCATCGGAGAGACCTATTACTAACATGGAGGTGAAAGTGACACTGATCGTTGCCATTGTGGCTGCTCTTGCTATCTCGGCTCACGCACAAAGAGATTTCAATGAACGACGAGGAAAGGAGAATGACACAGAGAGAGGACAAGGTGGCTTTGGAGGAAGGCCTGGTGGAATGCAGATGGGTGGTC 186  
8-1536    ----TAGCATCGGAGAGACCTATTACTAACATGGAGGTGAAAGTGACACTGATCGTTGCCATTGTGGCTGCTCTTGCTATCTCGGCTCACGCACAAAGAGATTTCAATGAACGACGAGGAAAGGAGAATGACACAGAGAGAGGACAAGGTGGCTTTGGAGGAAGGCCTGGTGGAATGCAGATGGGTGGTC 186  
8-1537    ----TAGCATCGGAGAGACCTATTACTAACATGGAGGTGAAAGTGACACTGATCGTTGCCATTGTGGCTGCTCTTGCTATCTCGGCTCACGCACAAAGAGATTTCAATGAACGACGAGGAAAGGAGAATGACACAGAGAGAGGACAAGGTGGCTTTGGAGGAAGGCCTGGTGGAATGCAGATGGGTGGTC 186  
8-1538    ----TAGCATCGGAGAGACCTATTACTAACATGGAGGTGAAAGTGACACTGATCGTTGCCATTGTGGCTGCTCTTGCTATCTCGGCTCACGCACAAAGAGATTTCAATGAACGACGAGGAAAGGAGAATGACACAGAGAGAGGACAAGGTGGCTTTGGAGGAAGGCCTGGTGGAATGCAGATGGGTGGTC 186  
8-1539    ----TAGCATCGGAGAGACCTATTACTAACATGGAGGTGAAAGTGACACTGATCGTTGCCATTGTGGCTGCTCTTGCTATCTCGGCTCACGCACAAAGAGATTTCAATGAACGACGAGGAAAGGAGAATGACACAGAGAGAGGACAAGGTGGCTTTGGAGGAAGGCCTGGTGGAATGCAGATGGGTGGTC 186  
8-1540    ----TAGCATCGGAGAGACCTATTACTAACATGGAGGTGAAAGTGACACTGATCGTTGCCATTGTGGCTGCTCTTGCTATCTCGGCTCACGCACAAAGAGATTTCAATGAACGACGAGGAAAGGAGAATGACACAGAGAGAGGACAAGGTGGCTTTGGAGGAAGGCCTGGTGGAATGCAGATGGGTGGTC 186  
8-1542    ----TAGCATCGGAGAGACCTATTACTAACATGGAGGTGAAAGTGACACTGATCGTTGCCATTGTGGCTGCTCTTGCTATCTCGGCTCACGCACAAAGAGATTTCAATGAACGACGAGGAAAGGAGAATGACACAGAGAGAGGACAAGGTGGCTTTGGAGGAAGGCCTGGTGGAATGCAGATGGGTGGTC 186  
8-1546    ----TAGCATCGGAGAGACCTATTACTAACATGGAGGTGAAAGTGACACTGATCGTTGCCATTGTGGCTGCTCTTGCTATCTCGGCTCACGCACAAAGAGATTTCAATGAACGACGAGGAAAGGAGAATGACACAGAGAGAGGACAAGGTGGCTTTGGAGGAAGGCCTGGTGGAATGCAGATGGGTGGTC 186  
8-1548    ----TAGCATCGGAGAGACCTATTACTAACATGGAGGTGAAAGTGACACTGATCGTTGCCATTGTGGCTGCTCTTGCTATCTCGGCTCACGCACAAAGAGATTTCAATGAACGACGAGGAAAGGAGAATGACACAGAGAGAGGACAAGGTGGCTTTGGAGGAAGGCCTGGTGGAATGCAGATGGGTGGTC 186  
8-1549    ----TAGCATCGGAGAGACCTATTACTAACATGGAGGTGAAAGTGACACTGATCGTTGCCATTGTGGCTGCTCTTGCTATCTCGGCTCACGCACAAAGAGATTTCAATGAACGACGAGGAAAGGAGAATGACACAGAGAGAGGACAAGGTGGCTTTGGAGGAAGGCCTGGTGGAATGCAGATGGGTGGTC 186  
8-1550    ------GCATCGGAGAGACCTATTACTAACATGGAGGTGAAAGTGACACTGATCGTTGCCATTGTGGCTGCTCTTGCTATCTCGGCTCACGCACAAAGAGATTTCAATGAACGACGAGGAAAGGAGAATGACACAGAGAGAGGACAAGGTGGCTTTGGAGGAAGGCCTGGTGGAATGCAGATGGGTGGTC 184  
8-1541    ----TAGCATCGGAGAGACCTATTACTAACATGGAGGTGAAAGTGACACTGATCGTTGCCATTGTGGCTGCTCTTGCTATCTCGGCTCACGCACAAAGAGATTTCAATGAACGACGAGGAAAGGAGAATGACACAGAGAGAGGACAAGGTGGCTTTGGGGGAAGGCCTGGTGGAATGCAGATGGGTGGTC 186  
8-1543    ----TAGCATCGGAGAGACCTATTACTAACATGGAGGTGAAAGTGACACTGATCGTTGCCATTGTGGCTGCTCTTGCTATCTCGGCTCACGCACAAAGAGATTTCAATGAACGACGAGGAAAGGAGAATGACACAGAGAGAGGACAAGGTGGCTTTGGAGGAAGGCCTGGTGGAATGCAGATGGGTGGTC 186  
8-2401    ----TAGCATCGGAGAGACCTATTACTAACATGGAGGTGAAAGTGACACTGATCGTTGCCATTGTGGCTGCTCTTGCTATCTCGGCTCACGCACAAAGAGATTTCAATGAACGACGAGGAAAGGAGAATGACACAGAGAGAGGACAAGGTGGCTTTGGAGGAAGGCCTGGTGGAATGCAGATGGGTGGTC 186  
8-2406    ----TAGCATCGGAGAGACCTATTACTAACATGGAGGTGAAAGTGACACTGATCGTTGCCATTGTGGCTGCTCTTGCTATCTCGGCTCACGCACAAAGAGATTTCAATGAACGACGAGGAAAGGAGAATGACACAGAGAGAGGACAAGGTGGCTTTGGAGGAAGGCCTGGTGGAATGCAGATGGGTGGTC 186  
8-2407    ----TAGCATCGGAGAGACCTATTACTAACATGGAGGTGAAAGTGACACTGATCGTTGCCATTGTGGCTGCTCTTGCTATCTCGGCTCACGCACAAAGAGATTTCAATGAACGACGAGGAAAGGAGAATGACACAGAGAGAGGACAAGGTGGCTTTGGAGGAAGGCCTGGTGGAATGCAGATGGGTGGTC 186  
8-2409    ----TAGCATCGGAGAGACCTATTACTAACATGGAGGTGAAAGTGACACTGATCGTTGCCATTGTGGCTGCTCTTGCTATCTCGGCTCACGCACAAAGAGATTTCAATGAACGACGAGGAAAGGAGAATGACACAGAGAGAGGACAAGGTGGCTTTGGAGGAAGGCCTGGTGGAATGCAGATGGGTGGTC 186  
8-2410    ----TAGCATCGGAGAGACCTATTACTAACATGGAGGTGAAAGTGACACTGATCGTTGCCATTGTGGCTGCTCTTGCTATCTCGGCTCACGCACAAAGAGATTTCAATGAACGACGAGGAAAGGAGAATGACACAGAGAGAGGACAAGGTGGCTTTGGAGGAAGGCCTGGTGGAATGCAGATGGGTGGTC 186  
8-2411    ----TAGCATCGGAGAGACCTATTACTAACATGGAGGTGAAAGTGACACTGATCGTTGCCATTGTGGCTGCTCTTGCTATCTCGGCTCACGCACAAAGAGATTTCAATGAACGACGAGGAAAGGAGAATGACACAGAGAGAGGACAAGGTGGCTTTGGAGGAAGGCCTGGTGGAATGCAGATGGGTGGTC 186  
8-2414    ----TAGCATCGGAGAGACCTATTACTAACATGGAGGTGAAAGTGACACTGATCGTTGCCATTGTGGCTGCTCTTGCTATCTCGGCTCACGCACAAAGAGATTTCAATGAACGACGAGGAAAGGAGAATGACACAGAGAGAGGACAAGGTGGCTTTGGAGGAAGGCCTGGTGGAATGCAGATGGGTGGTC 186  
8-2417    ----TAGCATCGGAGAGACCTATTACTAACATGGAGGTGAAAGTGACACTGATCGTTGCCATTGTGGCTGCTCTTGCTATCTCGGCTCACGCACAAAGAGATTTCAATGAACGACGAGGAAAGGAGAATGACACAGAGAGAGGACAAGGTGGCTTTGGAGGAAGGCCTGGTGGAATGCAGATGGGTGGTC 186  
8-2420    ----TAGCATCGGAGAGACCTATTACTAACATGGAGGTGAAAGTGACACTGATCGTTGCCATTGTGGCTGCTCTTGCTATCTCGGCTCACGCACAAAGAGATTTCAATGAACGACGAGGAAAGGAGAATGACACAGAGAGAGGACAAGGTGGCTTTGGAGGAAGGCCTGGTGGAATGCAGATGGGTGGTC 186  
8-2421    ----TAGCATCGGAGAGACCTATTACTAACATGGAGGTGAAAGTGACACTGATCGTTGCCATTGTGGCTGCTCTTGCTATCTCGGCTCACGCACAAAGAGATTTCAATGAACGACGAGGAAAGGAGAATGACACAGAGAGAGGACAAGGTGGCTTTGGAGGAAGGCCTGGTGGAATGCAGATGGGTGGTC 186  
8-2422    ----TAGCATCGGAGAGACCTATTACTAACATGGAGGTGAAAGTGACACTGATCGTTGCCATTGTGGCTGCTCTTGCTATCTCGGCTCACGCACAAAGAGATTTCAATGAACGACGAGGAAAGGAGAATGACACAGAGAGAGGACAAGGTGGCTTTGGAGGAAGGCCTGGTGGAATGCAGATGGGTGGTC 186  
8-2424    ----TAGCATCGGAGAGACCTATTACTAACATGGAGGTGAAAGTGACACTGATCGTTGCCATTGTGGCTGCTCTTGCTATCTCGGCTCACGCACAAAGAGATTTCAATGAACGACGAGGAAAGGAGAATGACACAGAGAGAGGACAAGGTGGCTTTGGAGGAAGGCCTGGTGGAATGCAGATGGGTGGTC 186  
8-2425    ----TAGCATCGGAGAGACCTATTACTAACATGGAGGTGAAAGTGACACTGATCGTTGCCATTGTGGCTGCTCTTGCTATCTCGGCTCACGCACAAAGAGATTTCAATGAACGACGAGGAAAGGAGAATGACACAGAGAGAGGACAAGGTGGCTTTGGAGGAAGGCCTGGTGGAATGCAGATGGGTGGTC 186  
8-2427    ----TAGCATCGGAGAGACCTATTACTAACATGGAGGTGAAAGTGACACTGATCGTTGCCATTGTGGCTGCTCTTGCTATCTCGGCTCACGCACAAAGAGATTTCAATGAACGACGAGGAAAGGAGAATGACACAGAGAGAGGACAAGGTGGCTTTGGAGGAAGGCCTGGTGGAATGCAGATGGGTGGTC 186  
8-2433    ----TAGCATCGGAGAGACCTATTACTAACATGGAGGTGAAAGTGACACTGATCGTTGCCATTGTGGCTGCTCTTGCTATCTCGGCTCACGCACAAAGAGATTTCAATGAACGACGAGGAAAGGAGAATGACACAGAGAGAGGACAAGGTGGCTTTGGAGGAAGGCCTGGTGGAATGCAGATGGGTGGTC 186  
8-2434    ----TAGCATCGGAGAGACCTATTACTAACATGGAGGTGAAAGTGACACTGATCGTTGCCATTGTGGCTGCTCTTGCTATCTCGGCTCACGCACAAAGAGATTTCAATGAACGACGAGGAAAGGAGAATGACACAGAGAGAGGACAAGGTGGCTTTGGAGGAAGGCCTGGTGGAATGCAGATGGGTGGTC 186  
8-2435    ----TAGCATCGGAGAGACCTATTACTAACATGGAGGTGAAAGTGACACTGATCGTTGCCATTGTGGCTGCTCTTGCTATCTCGGCTCACGCACAAAGAGATTTCAATGAACGACGAGGAAAGGAGAATGGCACAGAGAGAGGACAAGGTGGCTTTGGAGGAAGGCCTGGTGGAATGCAGATGGGTGGTC 186  
8-2436    ----TAGCATCGGAGAGACCTATTACTAACATGGAGGTGAAAGTGACACTGATCGTTGCCATTGTGGCTGCTCTTGCTATCTCGGCTCACGCACAAAGAGATTTCAATGAACGACGAGGAAAGGAGAATGACACAGAGAGAGGACAAGGTGGCTTTGGAGGAAGGCCTGGTGGAATGCAGATGGGTGGTC 186  
8-2437    ----TAGCATCGGAGAGACCTATTACTAACATGGAGGTGAAAGTGACACTGATCGTTGCCATTGTGGCTGCTCTTGCTATCTCGGCTCACGCACAAAGAGATTTCAATGAACGACGAGGAAAGGAGAATGACACAGAGAGAGGACAAGGTGGCTTTGGAGGAAGGCCTGGTGGAATGCAGATGGGTGGTC 186  
8-2438    ----TAGCATCGGAGAGACCTATTACTAACATGGAGGTGAAAGTGACACTGATCGTTGCCATTGTGGCTGCTCTTGCTATCTCGGCTCACGCACAAAGAGATTTCAATGAACGACGAGGAAAGGAGAATGACACAGAGAGAGGACAAGGTGGCTTTGGAGGAAGGCCTGGTGGAATGCAGATGGGTGGTC 186  
8-2439    ----TAGCATCGGAGAGACCTATTACTAACATGGAGGTGAGAGTGACACTGATCGTTGCCATTGTGGCTGCTCTTGCTATCTCGGCTCACGCACAAAGAGATTTCAATGAACGACGAGGAAAGGAGAATGACACAGAGAGAGGACAAGGTGGCTTTGGAGGAAGGCCTGGTGGAATGCAGATGGGTGGTC 186  
8-2440    ----TAGCATCGGAGAGACCTATTACTAACATGGAGGTGAAAGTGACACTGATCGTTGCCATTGTGGCTGCTCTTGCTATCTCGGCTCACGCACAAAGAGATTTCAATGAACGACGAGGAAAGGAGAATGACACAGAGAGAGGACAAGGTGGCTTTGGAGGAAGGCCTGGTGGAATGCAGATGGGTGGTC 186  
8-2442    ----TAGCATCGGAGAGACCTATTACTAACACGGAGGTGAAAGTGACACTGATCGTTGCCATTGTGGCTGCTCTTGCTATCTCGGCTCACGCACAAAGAGATTTCAATGAACGACGAGGAAAGGAGAATGACACAGAGAGAGGACAAGGTGGCTTTGGAGGAAGGCCTGGTGGAATGCAGATGGGTGGTC 186  
8-2444    ----TAGCATCGGAGAGACCTATTACTAACATGGAGGTGAAAGTGACACTGATCGTTGCCATTGTGGCTGCTCTTGCTATCTCGGCTCACGCACAAAGAGATTTCAATGAACGACGAGGAAAGGAGAATGACACAGAGAGAGGACAAGGTGGCTTTGGAGGAAGGCCTGGTGGAATGCAGATGGGTGGTC 186  
8-2445    ----TAGCATCGGAGAGACCTATTACTAACATGGAGGTGAAAGTGACACTGATCGTTGCCATTGTGGCTGCTCTTGCTATCTCGGCTCACGCACAAAGAGATTTCAGTGAACGACGAGGAAAGGAGAATGACACAGAGAGAGGACAAGGTGGCTTTGGAGGAAGGCCTGGTGGAATGCAGATGGGTGGTC 186  
8-2448    ----TAGCATCGGAGAGACCTATTACTAACATGGAGGTGAAAGTGACACTGATCGTTGCCATTGTGGCTGCTCTTGCTATCTCGGCTCACGCACAAAGAGATTTCAATGAACGACGAGGAAGGGAGAATGACACAGAGAGAGGACAAGGTGGCTTTGGAGGAAGGCCTGGTGGAATGCAGATGGGTGGTC 186  
8-2449    ----TAGCATCGGAGAGACCTATTACTAACATGGAGGTGAAAGTGACACTGATCGTTGCCATTGTGGCTGCTCTTGCTATCTCGGCTCACGCACAAAGAGATTTCAATGAACGACGAGGAAAGGAGAATGACACAGAGAGAGGACAAGGTGGCTTTGGAGGAAGGCCTGGTGGAATGCAGATGGGTGGTC 186  
8-2450    ----TAGCATCGGAGAGACCTATTACTAACATGGAGGTGAAAGTGACACTGATCGTTGCCATTGTGGCTGCTCTTGCTATCTCGGCTCACGCACAAAGAGATTTCAATGAACGACGAGGAAAGGAGAATGACACAGAGAGAGGACAAGGTGGCTTTGGAGGAAGGCCTGGTGGAATGCAGATGGGTGGTC 186  
8-2432    ----TAGCATCGGAGAGACCTATTACTAACATG---GTGAAAGTGACACTGATCGTTGCCATTGTGGCTGCTCTTGCTATCTCAGCTCACGCACAAAGGGATTACAATGAACTACGAGGAAATAAGAATGGCAGAGAGAGAGGACAAGGTCGCTTTGGAGGAAGGCCGGGTGGAATGCAGATGGGTGGAT 183  
8-2405    ----TAGCATCGGAGAGACCTATTACTAACATG---GTGAAAGTGACACTGATCGTTGCCATTGTGGCTGCTCTTGCTATCTCAGCTCACGCACAAAGAGATTACAATGAACTACGAGGAAATAAGAATGGCAGAGAGAGAGGACAAGGTCGCTTTGGAGGAAGGCCGGGTGGAATGCAGATGGGTGGAT 183  
8-2443    ----TAGCATCGGAGAGACCTATTACTAACATGGAGGTGAAAGTGACACTGAACGTTGCCATTGTGGCTGCTCTTGCTATCTCGGCTCACGCACAAAGAGATTTCAATGAACGACGAGGAAAGGAGAATGACACAGAGAGAGGACAAGGTGGCTTTGGAGGAAGGCCTGGTGGAATGCAGATGTGTGGTC 186  
8-2413    ----TAGCATCGGAGAGACCTATTACTAACATGGAGGTGAAAGTGACACTGATCGTTGCCATTGTGGCTGCTCTTGCTATCTCGGCTCACGCACAAAGAGATTTCAATGAACGACGAGGAAAGGAGAATGACACAGAGAGAGGACAAGGTGGCTTTGGAGGAAGGCCTGGTGGAATGCAGATGGGTGGTC 186  
8-2418    ----TAGCATCGGAGAGACCTATTACTAACATGGAGGTGAAAGTGACACTGATCGTTGCCATTGTGGCTGCTCTTGCTATCTCGGCTCACGCACAAAGAGATTTCAATGAACGACGAGGAAAGGAGAATGACACAGAGAGAGGACAAGGTGGCTTTGGAGGAAGGCCTGGTGGAATGCAGATGGGTGGTC 186  
8-2419    ----TAGCATCGGAGAGACCTATTACTAACATGGAGGTGAAAGTGACACTGATCGTTGCCATTGTGGCTGCTCTTGCTATCTCGGCTCACGCACAAAGAGATTTCAATGAACGACGAGGAAAGGAGAATGACACAGAGAGAGGACAAGGTGGCTTTGGAGGAAGGCCTGGTGGAATGCAGATGGGTGGTC 186  
8-2423    ----TAGCATCGGAGAGACCTATTACTAACATGGAGGTGAAAGTGACACTGATCGTTGCCATTGTGGCTGCTCTTGCTATCTCGGCTCACGCACAAAGAGATTTCAATGAACGACGAGGAAAGGAGAATGACACAGAGAGAGGACAAGGTGGCTTTGGAGGAAGGCCTGGTGGAATGCAGATGGGTGGTC 186  
8-2430    ------TAGCATCGGAGAGACCTTACAAACATGGAGGTGAAAGCAACATTGATCGTTGCCATCGTGGCTGCTCTTGCTATCTCGGCTCACGCACAAAGAGATTTCAATGAACGACGAGGAAAGGAGAATGACACAGAGAGAGGACAAGGTGGCTTTGGAGGAAGGCCTGGTGGAATGCAGATGGGTGGTC 184  
8-2446    ----TAGCATCGGAGAGACCTATTACTAACATGGAGGTGAAAGTGACACTGATCGTTGCCATTGTGGCTGCTCTTGCTATCTCGGCTCACGCACAAAGAGATTTCAATGAACGACGAGGAAAGGAGAATGACACAGAGAGAGGACAAGGTGGCTTTGGAGGAAGGCCTGGTGGAATGCAGATGGGTGGTC 186  
8-2447    ----TAGCATCGGAGAGACCTATTACTAACATGGAGGTGAAAGTGACACTGATCGTTGCCATTGTGGCTGCTCTTGCTATCTCGGCTCACGCACAAAGAGATTTCAATGAACGACGAGGAAAGGAGAATGACACAGAGAGAGGACAAGGTGGCTTTGGAGGAAGGCCTGGTGGAATGCAGATGGGTGGTC 186  
8-2441    ----TAGCATCGGAGAGACCTATTACTATCATG---GTGAAAGTGACACTGATCGTTGCCATTGTGGCTGCTCTTGCTATCTCGGCTCACGCACGAAGAGATTTCAATGAACGACGAGGAAAGGAGAATGGCAGAGAGAGAGGACAAGGTGGCTTTGGAGGAAGGCCTGGTGGAATGCAGACGGGTAGTC 183  
8-2404    ------TAGCATCGGAGAGACCTTACTAACATGGAGGTGAAAGTGACACTGATCGTTGCCATTGTGGCTGCTCTTGCTATCTCGACTCACGCACAAAGAGATTTCAATGAACGACGAGGAAAGGAGAATGACACAGAGAGAGGACAAGGTGGCTTTGGAGGAAGGCCTGGTGGAATGCAGATGGGTGGTC 184  
8-2415    ----TAGCATCGGAGAGACCTATTACTAACATGGAGGTGAAAGTGACACTGATCGTTGCCATTGTGGCTGCTCTTGCTATCTCGGCTCACGCACAAAGAGATTTCAATGAACGACGAGGAAAGGAGAATGACACAGAGAGAGGACAAGGTGGCTTTGGAGGAAGGCCTGGTGGAATGCAGATGGGTGGTC 186  
2-1517    ----TAGCATCGGAGAGACCTATTACTAACATGGAGGTGAAAGTGACACTGATCGTTGCCATTGTGGCTGCTCTTGCTATCTCGGCTCACGCACAAAGAGATTTCAATGAACGACGAGGAAAGGAGAATGACACAGAGAGAGGACAAGGTGGCTTTGGAGGAAGGCCTGGTGGAATGCAGATGGGTGGTC 186  
2-1501    ----TAGCATCGGAGAGACCTATTACTAACATGGAGGTGAAAGTGACACTGATCGTTGCCATTGTGGCTGCTCTTGCTATCTCGGCTCACGCACAAAGAGATTTCAATGAACGACGAGGAAAGGAGAATGACACAGAGAGAGGACAAGGTGGCTTTGGAGGAAGGCCTGGTGGAATGCAGATGGGTGGTC 186  
2-1503    ----TAGCATCGGAGAGACCTATTACTAACATGGAGGTGAAAGTGACACTGATCGTTGCCATTGTGGCTGCTCTTGCTATCTCGGCTCACGCACAAAGAGATTTCAATGAACGACGAGGAAAGGAGAATGACACAGAGAGAGGACAAGGTGGCTTTGGAGGAAGGCCTGGTGGAATGCAGATGGGTGGTC 186  
2-1505    ----TAGCATCGGAGAGACCTATTACTAACATGGAGGTGAAAGTGACACTGATCGTTGCCATTGTGGCTGCTCTTGCTATCTCGGCTCACGCACAAAGAGATTTCAATGAACGACGAGGAAAGGAGAATGACACAGAGAGAGGACAAGGTGGCTTTGGAGGAAGGCCTGGTGGAATGCAGATGGGTGGTC 186  
2-1508    ----TAGCATCGGAGAGACCTATTACTAACATGGAGGTGAAAGTGACACTGATCGTTGCCATTGTGGCTGCTCTTGCTATCTCGGCTCACGCACAAAGAGATTTCAATGAACGACGAGGAAAGGAGAATGACACAGAGAGAGGACAAGGTGGCTTTGGAGGAAGGCCTGGTGGAATGCAGATGGGTGGTC 186  
2-1509    ----TAGCATCGGAGAGACCTATTACTAACATGGAGGTGAAAGTGACACTGATCGTTGCCATTGTGGCTGCTCTTGCTATCTCGGCTCACGCACAAAGAGATTTCAATGAACGACGAGGAAAGGAGAATGACACAGAGAGAGGACAAGGTGGCTTTGGAGGAGGGCCTGGTGGAATGCAGATGGGTGGTC 186  
2-1510    ----TAGCATCGGAGAGACCTATTACTAACATGGAGGTGAAAGTGACACTGATCGTTGCCATTGTGGCTGCTCTTGCTATCTCGACTCACGCACAAAGAGATTTCAATGAACGACGAGGAAAGGAGAATGACACAGAGAGAGGACAAGGTGGCTTTGGAGGAAGGCCTGGTGGAATGCAGATGGGTGGTC 186  
2-1519    ----TAGCATCGGAGAGACCTATTACTAACATGGAGGTGAAAGTGACACTGATCGTTGCCATTGTGGCTGCTCTTGCTATCTCGGCTCACGCACAAAGAGATTTCAATGAACGACGAGGAAAGGAGAATGACACAGAGAGAGGACAAGGTGGCTTTGGAGGAAGGCCTGGTGGAATGCAGATGGGTGGTC 186  
2-1521    ----TAGCATCGGAGAGACCTATTACTAACATGGAGGTGAAAGTGACACTGATCGTTGCCATTGTGGCTGCTCTTGCTATCTCGGCTCACGCACAAAGAGATTTCAATGAACGACGAGGAAAGGAGAATGACACAGAGAGAGGACAAGGTGGCTTTGGAGGAAGGCCTGGTGGAATGCAGATGGGTGGTC 186  
2-1522    ----TAGCATCGGAGAGACCTATTACTAACATGGAGGTGAAAGTGACACTGATCGTTGCCATTGTGGCTGCTCTTGCTATCTCGGCTCACGCACAAAGAGATTTCAATGAACGACGAGGAAAGGAGAATGACACAGAGAGAGGACAAGGTGGCTTTGGAGGAAGGCCTGGTGGAATGCAGATGGGTGGTC 186  
2-1523    ----TAGCATCGGAGAGACCTATTACTAACATGGAGGTGAAAGTGACACTGATCGTTGCCATTGTGGCTGCTCTTGCTATCTCGGCTCACGCACAAAGAGATTTCAATGAACGACGAGGAAAGGAGAATGACACAGAGAGAGGACAAGGTGGCTTTGGAGGAAGGCCTGGTGGAATGCAGATGGGTGGTC 186  
2-1527    ----TAGCATCGGAGAGACCTATTACTAACATGGAGGTGAAAGTGACACTGATCGTTGCCATTGTGGCTGCTCTTGCTATCTCGGCTCACGCACAAAGAGATTTCAATGAACGACGAGGAAAGGAGAATGACACAGAGAGAGGACAAGGTGGCTTTGGAGGAAGGCCTGGTGGAATGCAGATGGGTGGTC 186  
2-1533    ----TAGCATCGGAGAGACCTATTACTAACATGGAGGTGAAAGTGACACTGATCGTTGCCATTGTGGCTGCTCTTGCTATCTCGGCTCACGCACAAAGAGATTTCAATGAACGACGAGGAAAGGAGAATGACACAGAGAGAGGACAAGGTGGCTTTGGAGGAAGGCCTGGTGGAATGCAGATGGGTGGTC 186  
2-1535    ----TAGCATCGGAGAGACCTATTACTAACATGGAGGTGAAAGTGACACTGATCGTTGCCATTGTGGCTGCTCTTGCTATCTCGGCTCACGCACAAAGAGATTTCAATGAACGACGAGGAAAGGAGAATGACACAGAGAGAGGACAAGGTGGCTTTGGAGGAAGGCCTGGTGGAATGCAGATGGGTGGTC 186  
2-1536    ----TAGCATCGGAGAGACCTATTACTAACATGGAGGTGAAAGTGACACTGATCGTTGCCATTGTGGCTGCTCTTGCTATCTCGGCTCACGCACAAAGAGATTTCAATGAACGACGAGGAAAGGAGAATGACACAGAGAGAGGACAAGGTGGCTTTGGAGGAAGGCCTGGTGGAATGCAGATGGGTGGTC 186  
2-1537    -----AGCATCGGAGAGACCTATTACTAACATGGAGGTGAAAGTGACACTGATCGTTGCCATTGTGGCTGCTCTTGCTATCTCGGCTCACGCACAAAGAGATTTCAATGAACGACGAGGAAAGGAGAATGACACAGAGAGAGGACAAGGTGGCTTTGGAGGAAGGCCTGGTGGAATGCAGATGGGTGGTC 185  
2-1538    ----TAGCATCGGAGAGACCTATTACTAACATGGAGGTGAAAGTGACACTGATCGTTGCCATTGTGGCTGCTCTTGCTATCTCGGCTCACGCACAAAGAGATTTCAATGAACGACGAGGAAAGGAGAATGACACAGAGAGAGGACAAGGTGGCTTTGGAGGAAGGCCTGGTGGAATGCAGATGGGTGGTC 186  
2-1539    ----TAGCATCGGAGAGACCTATTACTAACATGGAGGTGAAAGTGACACTGATCGTTGCCATTGTGGCTGCTCTTGCTATCTCGGCTCACGCACAAAGAGATTTCAATGAACGACGAGGAAAGGAGAATGACACAGAGAGAGGACAAGGTGGCTTTGGAGGAAGGCCTGGTGGAATGCAGATGGGTGGTC 186  
2-1543    ----TAGCATCGGAGAGACCTATTACTAACATGGAGGTGAAAGTGACACTGATCGTTGCCATTGTGGCTGCTCTTGCTATCTCGGCTCACGCACAAAGAGATTTCAATGAACGACGAGGAAAGGAGAGTGACACAGAGAGAGGACAAGGTGGCTTTGGAGGAAGGCCTGGTGGAATGCAGATGGGTGGTC 186  
2-1546    ----TAGCATCGGAGAGACCTATTACTAACATGGAGGTGAAAGTGACACTGATCGTTGCCATTGTGGCTGCTCTTGCTATCTCGGCTCACGCACAAAGAGATTTCAATGAACGACGAGGAAAGGAGAATGACACAGAGAGAGGACAAGGTGGCTTTGGAGGAAGGCCTGGTGGAATGCAGATGGGTGGTC 186  
2-1547    ----TAGCATCGGAGAGACCTATTACTAACATGGAGGTGAAAGTGACACTGATCGTTGCCATTGTGGCTGCTCTTGCTATCTCGGCTCACGCACAAAGAGATTTCAATGAACGACGAGGAAAGGAGAATGACACAGAGAGAGGACAAGGTGGCTTTGGAGGAAGGCCTGGTGGAATGCAGATGGGTGGTC 186  
2-1529    ------TAGCATCGGAGAGACCTTACTAACATGGAGGTGAAAGTGACACTGATCGTTGCCATTGTGGCTGCTCTTGCTATCTCGGCTCACACACAAAGAGATTACAATGAACGACGAGGAAATGAGAATGGCAGAGAGAGAGGACAAGGTCGCTTTGGAGGAAGGCCTGGTGGAATGCAGATGGGTGGAC 184  
2-1540    ----TAGCATCGGAGAGACCTATTACTAACATG---GTGAAAGTGACACTGATCGTTGCCATTGTGGCTGCTCTTGCTATCTCAGCTCACGCACAAAGAGATTACAATGAACTACGAGGAAATAAGAATGGCAGAGAGAGAGGACAAGGTCGCTTTGGAGGAAGGCCGGGTGGAATGCAGATGGGTGGAT 183  
2-1506    ----TAGCATCGGAGAGACCTATTACTAACATGGAGGTGAAAGTGACACTGATCGTTGCCATTGTGGCTGCTCTTGCTATCTCGGCTCACGCACAAAGAGATTTCAATGAACGACGAGGAAAGGAGAATGACACAGAGAGAGGACAAGGTGGCTTTGGAGGAAGGCCTGGTGGAATGCAGATGGGTGGTC 186  
2-1507    ------TAGCATCGGAGAGACCTTACAAACATGGAGGTGAAAGTGACACTGATCGTTGCCATTGTGGCTGCTCTTGCTATCTCGGCTCACGCACAAAGAGATTTCAATGAACGACGAGGAAAGGAGAATGACACAGAGAGAGGACAAGGTGGCTTTGGAGGAAGGCCGGGTGGAATGCAGATGGGTGGTC 184  
2-1511    ------TAGCATCGGAGAGACCTTACAAACATGGAGGTGAAAGTGACACTGATCGTTGCCATTGTGGCTGCTCTTGCTATCTCGGCTCACGCACAAAGAGATTTCAATGAACGACGAGGAAAGGAGAATGACACAGAGAGAGGACAAGGTGGCTTTGGAGGAAGGCCGGGTGGAATGCAGATGGGTGGTC 184  
2-1514    ----TAGCATCGGAGAGACCTATTACTAACATGGAGGTGAAAGTGACACTGATCGTTGCCATTGTGGCTGCTCTTGCTATCTCGGCTCACGCACAAAGAGATTTCAATGAACGACGAGGAAAGGAGAATGACACAGAGAGAGGACAAGGTGGCTTTGGAGGAAGGCCTGGTGGAATGCAGATGGGTGGTC 186  
2-1516    ----TAGCATCGGAGAGACCTATTACTAACATGGAGGTGAAAGTGACACTGATCGTTGCCATTGTGGCTGCTCTTGCTATCTCGGCTCACGCACAAAGAGATTTCAATGAACGACGAGGAAAGGAGAATGACACAGAGAGAGGACAAGGTGGCTTTGGAGGAAGGCCTGGTGGAATGCAGATGGGTGGTC 186  
2-1528    ----TAGCATCGGAGAGACCTATTACTAACATGGAGGTGAAAGTGACACTGATCGTTGCCATTGTGGCTGCTCTTGCTATCTCGGCTCACGCACAAAGAGATTTCAATGAACGACGAGGAAAGGAGAATGACACAGAGAGAGGACAAGGTGGCTTTGGAGGAAGGCCTGGTGGAATGCAGATGGGTGGTC 186  
2-1532    ----TAGCATCGGAGAGACCTATTACTAACATGGAGGTGAAAGTGACACTGATCGTTGCCATTGTGGCTGCTCTTGCTATCTCGGCTCACGCACAAAGAGATTTCAATGAACGACGAGGAAAGGAGAATGACACAGAGAGAGGACAAGGTGGCTTTGGAGGAAGGCCTGGTGGAATGCAGATGGGTGGTC 186  
2-1541    ------TAGCATCGGAGAGACCTTACAAACATGGAGGTGAAAGTGACACTGATCGTTGCCATTGTGGCTGCTCTTGCTATCTCGGCTCACGCACAAAGAGATTTCAATGAACGACGAGGAAAGGAGAATGACACAGAGAGAGGACAAGGTGGCTTTGGAGGAAGGCCGGGTGGGATGCAGATGGGTGGTC 184  
2-1542    ----TAGCATCGGAGAGACCTATTACTAACATGGAGGTGAAAGTGACACTGATCGTTGCCATTGTGGCTGCTCTTGCTATCTCGGCTCACGCACAAAGAGATTTCAATGAACGACGAGGAAAGGAGAATGACACAGAGAGAGGACAAGGTGGCTTTGGAGGAAGGCCTGGTGGAATGCAGATGGGTGGTC 186  
2-2448    ----TAGCATCGGAGAGACCTATTACTAACATGGAGGTGAAAGTGACACTGATCGTTGCCATTGTGGCTGCTCTTGCTATCTCGGCTCACGCACAAAGAGATTTCAATGAACGACGAGGAAAGGAGAATGACACAGAGAGAGGACAAGGTGGCTTTGGAGGAAGGCCTGGTGGAATGCAGATGGGTGGTC 186  
2-2405    ----TAGCATCGGAGAGACCTATTACTAACATGGAGGTGAAAGTGACACTGATCGTTGCCATTGTGGCTGCTCTTGCTATCTCGGCTCACGCACAAAGAGATTTCAATGAACGACGAGGAAAGGAGAATGACACAGAGAGAGGACAAGGTGGCTTTGGAGGAAGGCCTGGTGGAATGCAGATGGGTGGTC 186  
2-2406    ----TAGCATCGGAGAGACCTATTACTAACATGGAGGTGAAAGTGACACTGATCGTTGCCATTGTGGCTGCTCTTGCTATCTCGGCTCACGCACAAAGAGATTTCAATGAACGACGAGGAAAGGAGAATGACACAGAGAGAGGACAAGGTGGCTTTGGAGGAAGGCCTGGTGGAATGCAGATGGGTGGTC 186  
2-2407    ----TAGCATCGGAGAGACCTATTACTAACATGGAGGTGAAAGTGACACTGATCGTTGCCATTGTGGCTGCTCTTGCTATCTCGGCTCACGCACAAAGAGATTTCAATGAACGACGAGGAAAGGAGAATGACACAGAGAGAGGACAAGGTGGCTTTGGAGGAAGGCCTGGTGGAATGCAGATGGGTGGTC 186  
2-2408    ----TAGCATCGGAGAGACCTATTACTAACATGGAGGTGAAAGTGACACTGATCGTTGCCATTGTGGCTGCTCTTGCTATCTCGGCTCACGCACAAAGAGATTTCAATGAACGACGAGGAAAGGAGAATGACACAGAGAGAGGACAAGGTGGCTTTGGAGGAAGGCCTGGTGGAATGCAGATGGGTGGTC 186  
2-2410    ----TAGCATCGGAGAGACCTATTACTAACATGGAGGTGAAAGTGACACTGATCGTTGCCATTGTGGCTGCTCTTGCTATCTCGGCTCACGCACAAAGAGATTTCAATGAACGACGAGGAAAGGAGAATGACACAGAGAGAGGACAAGGTGGCTTTGGAGGAAGGCCTGGTGGAATGCAGATGGGTGGTC 186  
2-2412    ----TAGCATCGGAGAGACCTATTACTAACATGGAGGTGAAAGTGACACTGATCGTTGCCATTGTGGCTGCTCTTGCTATCTCGGCTCACGCACAAAGAGATTTCAATGAACGACGAGGAAAGGAGAATGACACAGAGAGAGGACAAGGTGGCTTTGGAGGAAGGCCTGGTGGAATGCAGATGGGTGGTC 186  
2-2413    ----TAGCATCGGAGAGACCTATTACTAACATGGAGGTGAAAGTGACACTGATCGTTGCCATTGTGGCTGCTCTTGCTATCTCGGCTCACGCACAAAGAGATTTCAATGAACGACGAGGAAAGGAGAATGACACAGAGAGAGGACAAGGTGGCTTTGGAGGAAGGCCTGGTGGAATGCAGATGGGTGGTC 186  
2-2419    ----TAGCATCGGAGAGACCTATTACTAACATGGAGGTGAAAGTGACACTGATCGTTGCCATTGTGGCTGCTCTTGCTATCTCGGCTCACACACAAAGAGATTTCAATGAACGACGAGGAAAGGAGAATGACACAGAGAGAGGACAAGGTGGCTTTGGAGGAAGGCCTGGTGGAATGCAGATGGGTGGTC 186  
2-2420    ----TAGCATCGGAGAGACCTATTACTAACATGGAGGTGAAAGTGACACTGATCGTTGCCATTGTGGCTGCTCTTGCTATCTCGGCTCACGCACAAAGAGATTTCAATGAACGACGAGGAAAGGAGAATGACACAGAGAGAGGACAAGGTGGCTTTGGAGGAAGGCCTGGTGGAATGCAGATGGGTGGCC 186  
2-2422    ----TAGCATCGGAGAGACCTATTACTAACATGGAGGTGAAAGTGACACTGATCGTTGCCATTGTGGCTGCTCTTGCTATCTCGGCTCACGCACAAAGAGATTTCAATGAACGACGAGGAAAGGAGAATGACACAGAGAGAGGACAAGGTGGCTTTGGAGGAAGGCCTGGTGGAATGCAGATGGGTGGTC 186  
2-2424    ----TAGCATCGGAGAGACCTATTACTAACATGGAGGTGAAAGTGACACTGATCGTTGCCATTGTGGCTGCTCTTGCTATCTCGGCTCACGCACAAAGAGATTTCAATGAACGACGAGGAAAGGAGAATGACACAGAGAGAGGACAAGGTGGCTTTGGAGGAAGGCCTGGTGGAATGCAGATGGGTGGTC 186  
2-2427    ----TAGCATCGGAGAGACCTATTACTAACATGGAGGTGAAAGTGACACTGATCGTTGCCATTGTGGCTGCTCTTGCTATCTCGGCTCACGCACAAAGAGATTTCAATGAACGACGAGGAAAGGAGAATGACACAGAGAGAGGACAAGGTGGCTTTGGAGGAAGGCCTGGTGGAATGCAGATGGGTGGTC 186  
2-2428    ----TAGCATCGGAGAGACCTATTACTAACATGGAGGTGAAAGTGACACTGATCGTTGCCATTGTGGCTGCTCTTGCTATCTCGGCTCACGCACAAAGAGATTTCAACGAACGACGAGGAAAGGAGAATGACACAGAGAGAGGACAAGGTGGCTTTGGAGGAAGGCCTGGTGGAATGCAGATGGGTGGTC 186  
2-2429    ----TAGCATCGGAGAGACCTATTACTAACATGGAGGTGAAAGTGACACTGATCGTTGCCATTGTGGCTGCTCTTGCTATCTCGGCTCACGCACAAAGAGATTTCAATGAACGACGAGGAAAGGAGAATGACACAGAGAGAGGACAAGGTGGCTTTGGAGGAAGGCCTGGTGGAATGCAGATGGGTGGTC 186  
2-2431    ----TAGCATCGGAGAGACCTATTACTAACATGGAGGTGAAAGTGACACTGATCGTTGCCATTGTGGCTGCTCTTGCTATCTCGGCTCACGCACAAAGAGATTTCAATGAACGACGAGGAAAGGAGAATGACACAGAGAGAGGACAAGGTGGCTTTGGAGGAAGGCCTGGTGGAATGCAGATGGGTGGTC 186  
2-2436    ----TAGCATCGGAGAGACCTATTACTAACATGGAGGTGAAAGTGACACTGATCGTTGCCATTGTGGCTGCTCTTGCTATCTCGGCTCACGCACAAAGAGATTTCAATGAACGACGAGGAAAGGAGAATGACACAGAGAGAGGACAAGGTGGCTTTGGAGGAAGGCCTGGTGGAATGCAGATGGGTGGTC 186  
2-2441    -----AGCATCGGAGAGACCTATTACTAACATGGAGGTGAAAGTGACACTGATCGTTGCCATTGTGGCTGCTCTTGCTATCTCGGCTCACGCACAAAGAGATTTCAATGAACGACGAGGAAAGGAGAATGACACAGAGAGAGGACAAGGTGGCTTTGGAGGAAGGCCTGGTGGAATGCAGATGGGTGGTC 185  
2-2446    ----TAGCATCGGAGAGACCTATTACTAACATGGAGGTGAAAGTGACACTGATCGTTGCCATTGTGGCTGCTCTTGCTATCTCGGCTCACGCACAAAGAGATTTCAATGAACGACGAGGAAAGGAGAATGACACAGAGAGAGGACAAGGTGGCTTTGGAGGAAGGCCTGGTGGAATGCAGATGGGTGGTC 186  
2-2416    -----AGCATCGGAGAGACCTATTACTAACATGGAGGTGAAAGTGACACTGATCGTTGCCATTGTGGCTGCTCTTGCTATCTCGGCTCACGCACAAAGAGATTTCAATGAACGACGAGGAAAGGAGAATGACACAGAGAGAGGACAAGGTGGCTTTGGAGGAAGGCCTGGTGGAATGCAGATGGGTGGTC 185  
2-2411    ----TAGCATCGGAGAGACCTATTACTAACATG---GTGAAAGTGACACTGATCGTTGCCATTGTGGCTGCTCTTGCTATCTCAGCTCACGCACAAAGAGATTACAATGAACTACGAGGAAATAAGAATGGCAGAGAGAGAGGACAAGGTCGCCGAGGTGTAGAATWA---------------------- 161  
2-2415    ----TAGCATCGGAGAGACCTATTACTAACATG---GTGAAAGTGACACTGATCGTTGCCATTGTGGCTGCTCTTGCTATCTCAGCTCACGCACAAAGAGATTACAATGAACTACGAGGAAATAAGAATGGCAGAGAGAGAGGACAAGGTCGCCGAGGTGTAGAATTT---------------------- 161  
2-2440    ----TAGCATCGGAGAGACCTATTACTAACATG---GTGAAAGTGACACTGATCGTTGCCATTGTGGCTGCTCTTGCTATCTCAGCTCACGCACAAAGAGATTACAATGAACTACGAGGAAATAAGAATGGCAGAGAGAGAGGACAAGGTCGCCGAGGTGTAGAATTT---------------------- 161  
2-2423    ----TAGCATCGGAGAGACCT------------------------------------------------------------------------------------------------------------------------------------------------------------------------- 17   
2-2418    ----TAGCATCGGAGAGACCTATTACTATCATG---GTGAAAGTGACACTGATCGTTGCCATTGTGGCTGCTCTTGCTATCTCGGCTCACGCAGAAAGAGATTTCAATGAACGACGAGGAAAGGAGAATGGCAGAGAGAGAGGACAAGGTGGCTTTGGAGGAAGGCCTGGTGGAATGCAGACGGGTAGTC 183  
2-2421    ----TAGCATCGGAGAGACCTATTACTAACATGGAGGTGAAAGTGACACTGATCGTTGCCATTGTGGCTGCTCTTGCTATCTCGGCTCACGCACGAAGAGATTTCAATGAACGACGAGGAAAGGAGAATGGCAGAGAGAGAGGACAAGGTGGCTTTGGAGGAAGGCCTGGTGGAATGCAGACGGGTAGTC 186  
2-2438    ----TAGCATCGGAGAGACCTATTACTAACATGGAGGTGAAAGTGACACTGATCGTTGCCATTGTGGCTGCTCTTGCTATCTCGGCTCACGCACAAAGAGATTTCAATGAACGACGAGGAAAGGAGAATGACACAGAGAGAGGACAAGGTGGCTTTGGAGGAAGGCCTGGTGGAATGCAGATGGGTGGTC 186  
2-2409    -----AGCATCGGAGAGACCTATTACTAACATG---GTGAAAGTGACACTGATCGTTGCCATTGTGGCTGCTCTTGCTATCTCAGCTCACGCACAAAGAGATTACAATGAACTACGAGGAAATAAGAATGGCAGAGAGAGAGGACAAGGTCGCTTTGGAGGAAGGCCGGGTGGAATGCAGATGGGTGGAT 182  
2-2444    ----TAGCATCGGAGAGACCTATTACTAACATG---GTGAAAGTGACACTGATCGTTGCCATTGTGGCTGCTCTTGCTATCTCAGCTCACGCACAAAGAGATTACAATGAACTACGAGGAAATAAGAATGGCAGAGAGAGAGGACAAGGTCGCTTTGGAGGAAGGCCGGGTGGAATGCAGATGGGTGGAT 183  
2-2414    ----TAGCATCGGAGAGACCTATTACTAACATGGAGGTGAAAGTGACACTGATCGTTGCCATTGTGGCTGCTCTTGCTATCTCGGCTCACGCACAAAGAGATTTCAATGAACGACGAGGAAAGGAGAATGACACAGAGAGAGGACAAGGTGGCTTTGGAGGAAGGCCTGGTGGAATGCAGATGGGTGGTC 186  
2-2403    ----TAGCATCGGAGAGACCTATTACTAACATGGAGGTGAAAGTGACACTGATCGTTGCCATTGTGGCTGCTCTTGCTATCTCGGCTCACGCACAAAGAGATTTCAATGAACGACGAGGAAAGGAGAATGACACAGAGAGAGGACAAGGTGGCTTTGGAGGAAGGCCTGGTGGAATGCAGATGGGTGGTC 186  
2-2404    ----TAGCATCGGAGAGACCTATTACTATCATG---GTGAAAGTGACACTGATCGTTGCCATTGTGGCTGCTCTTGCTATCTCGGCTCACGCAGAAAGAGATTTCAATGAACGACGAGGAAAGGAGAATGGCAGAGAGAGAGGACAAGGTGGCTTTGGAGGAAGGCCTGGTGGAATGCAGACGGGTAGTC 183  
2-2425    ----TAGCATCGGAGAGACCTATTACTATCATG---GTGAAAGTGACACTGATCGTTGCCATTGTGGCTGCTCTTGCTATCTCGGCTCACGCAGAAAGAGATTTCAATGAACGACGAGGAAAGGAGAATGGCAGAGAGAGAGGACAAGGTGGCTTTGGAGGAAGGCCTGGTGGAATGCAGACGGGTAGTC 183  
2-2426    ----TAGCATCGGAGAGACCTATTACTAACATGGAGGTGAAAGTGACACTGATCGTTGCCATTGTGGCTGCTCTTGCTATCTCGGCTCACGCACAAAGAGATTTCAATGAACGACGAGGAAAGGAGAATGACACAGAGAGAGGACAAGGTGGCTTTGGAGGAAGGCCTGGTGGAATGCAGATGGGTGGTC 186  
2-2430    ----TAGCATCGGAGAGACCTATTACTAACATGGAGGTGAAAGTGACACTGATCGTTGCCATTGTGGCTGCTCTTGCTATCTCGGCTCACGCACAAAGAGATTTCAATGAACGACGAGGAAAGGAGAATGACACAGAGAGAGGACAAGGTGGCTTTGGAGGAAGGCCTGGTGGAATGCAGATGGGTGGTC 186  
2-2432    ----TAGCATCGGAGAGACCTATTACTATCATG---GTGAAAGTGACACTGATCGTTGCCATTGTGGCTGCTCTTGCTATCTCGGCTCACGCACAAAGAGATTTCAATGAACGACGAGGAAAGGAGAATGACACAGAGAGAGGACAAGGTGGCTTTGGAGGAAGGCCTGGTGGAATGCAGATGGGTGGTC 183  
2-2437    ----TAGCATCGGAGAGACCTATTACTATCATG---GTGAAAGTGACACTGATCGTTGCCATTGTGGCTGCTCTTGCTATCTCAGCTCACGCACAAAGAGATTTCAATGAACGACGAGGAAAGGAGAATGGCAGAGAGAGAGGACAAGGTGGCTTTGGAGGAAGGCCTGGTGGAATGCAGACGGGTAGTC 183  
2-2439    ----TAGCATCGGAGAGACCTATTACTAACATGGAGGTGAAAGTGACACTGATCGTTGCCATTGTGGCTGCTCTTGCTATCTCGGCTCACGCACAAAGAGATTTCAATGAACGACGAGGAAAGGAGAATGACACAGAGAGAGGACAAGGTGGCTTTGGAGGAAGGCCTGGTGGAATGCAGATGGGTGGTC 186  
2-2442    ----TAGCATCGGAGAGACCTATTACTAACATGGAGGTGAAAGTGACACTGATCGTTGCCATTGTGGCTGCTCTTGCTATCTCGGCTCACGCACAAAGAGATTTCAATGAACGACGAGGAAAGGAGAATGACACAGAGAGAGGACAAGGTGGCTTTGGAGGAAGGCCTGGTGGAATGCAGATGGGTGGTC 186  
2-2443    ----TAGCATCGGAGAGACCTATTACTAACATGGAGGTGAAAGTGACACTGATCGTTGCCATTGTGGCTGCTCTTGCTATCTCGGCTCACGCACAAAGAGATTTCAATGAACGACGAGGAAAGGAGAATGACACAGAGAGAGGACAAGGTGGCTTTGGAGGAAGGCCTGGTGGAATGCAGATGGGTGGTC 186  
2-2445    ----TAGCATCGGAGAGACCTATTACTAACATGGAGGTGAAAGTGACACTGATCGTTGCCATTGTGGCTGCTCTTGCTATCTCGGCTCACGCACAAAGAGATTTCAATGAACGACGAGGAAAGGAGAATGACACAGAGAGAGGACAAGGTGGCTTTGGAGGAAGGCCTGGTGGAATGCAGATGGGTGGTC 186  
2-2435    ----TAGCATCGGAGAGACCTATTACTAACATGGAGGTGAAAGTGACACTGATCGTTGCCATTGTGGCTGCTCTTGCTATCTCGGCTCACGCACGAAGAGATTTCAATGAACGACGAGGAAAGGAGAATGGCAGAGAGAGAGGACAAGGTGGCTTTGGAGGAAGGCCTGGTGGAATGCAGACGGGTAGTC 186  
2-2447    ----TAGCATCGGAGAGACCTATTACTAACATGGAGGTGAAAGTGACACTGATCGTTGCCATTGTGGCTGCTCTTGCTATCTCGGCTCACGCACGAAGAGATTTCAATGAACGACGAGGAAAGGAGAATGGCAGAGAGAGAGGACAAGGTGGCTTTGGAGGAAGGCCTGGTGGAATGCAGACGGGTAGTC 186  
2-2401    ----TAGCATCGGAGAGACCTATTACTAACATGGAGGTGAAAGTGACACTGATCGTTGCCATTGTGGCTGCTCTTGCTATCTCGGCTCACGCACGAAGAGATTTCAATGAACGACGAGGAAAGGAGAATGGCAGAGAGAGAGGACAAGGTGGCTTTGGAGGAAGGCCTGGTGGAATGCAGACGGGTAGTC 186  
9-1504    ----TAGCATCGGAGAGACCTATTACTAACATGGAGGTGAAAGTGACACTGATCGTTGCCATTGTGGCTGCTCTTGCTATCTCGGCTCACGCACAAAGAGATTTCAATGAACGACGAGGAAAGGAGAATGACACAGAGAGAGGACAAGGTGGCTTTGGAGGAAGGCCTGGTGGAATGCAGATGGGTGGTC 186  
9-1505    ----TAGCATCGGAGAGACCTATTACTAACATGGAGGTGAAAGTGACACTGATCGTTGCCATTGTGGCTGCTCTTGCTATCTCGGCTCACGCACAAAGAGATTTCAATGAACGACGAGGAAAGGAGAATGACACAGAGAGAGGACAAGGTGGCTTTGGAGGAAGGCCTGGTGGAATGCAGATGGGTGGTC 186  
9-1506    ----TAGCATCGGAGAGACCTATTACTAACATGGAGGTGAAAGTGACACTGATCGTTGCCATTGTGGCTGCTCTTGCTATCTCGGCTCACGCACAAAGAGATTTCAATGAACGACGAGGAAAGGAGAATGACACAGAGAGAGGACAAGGTGGCTTTGGAGGAAGGCCTGGTGGAATGCAGATGGGTGGTC 186  
9-1507    ----TAGCATCGGAGAGACCTATTACTAACATGGAGGTGAAAGTGACACTGATCGTTGCCATTGTGGCTGCTCTTGCTATCTCGGCTCACGCACAAAGAGATTTCAATGAACGACGAGGAAAGGAGAATGACACAGAGAGAGGACAAGGTGGCTTTGGAGGAAGGCCTGGTGGAATGCAGATGGGTGGTC 186  
9-1509    ----TAGCATCGGAGAGACCTATTACTAACATGGAGGTGAAAGTGACACTGATCGTTGCCATTGTGGCTGCTCTTGCTATCTCGGCTCACGCACAAAGAGATTTCAATGAACGACGAGGAAAGGAGAATGACACAGAGAGAGGACAAGGTGGCTTTGGAGGAAGGCCTGGTGGAATGCAGATGGGTGGTC 186  
9-1512    ----TAGCATCGGAGAGACCTATTACTAACATGGAGGTGAAAGTGACACTGATCGTTGCCATTGTGGCTGCTCTTGCTATCTCGGCTCACGCACAAAGAGATTTCAATGAACGACGAGGAAAGGAGAATGACACAGAGAGAGGACAAGGTGGCTTTGGAGGAAGGCCTGGTGGAATGCAGATGGGTGGTC 186  
9-1514    ----TAGCATCGGAGAGACCTATTACTAACATGGAGGTGAAAGTGACACTGATCGTTGCCATTGTGGCTGCTCTTGCTATCTCGGCTCGCGCACAAAGAGATTTCAATGAACGACGAGGAAAGGAGAATGACACAGAGAGAGGACAAGGTGGCTTTGGAGGAAGGCCTGGTGGAATGCAGATGGGTGGTC 186  
9-1515    ----TAGCATCGGAGAGACCTATTACTAACATGGAGGTGAAAGTGACACTGATCGTTGCCATTGTGGCTGCTCTTGCTATCTCGGCTCACGCACAAAGAGATTTCAATGAACGACGAGGAAAGGAGAATGACACAGAGAGAGGACAAGGTGGCTTTGGAGGAAGGCCTGGTGGAATGCAGATGGGTGGTC 186  
9-1516    ----TAGCATCGGAGAGACCTATTACTAACATGGAGGTGAAAGTGACACTGATCGTTGCCATTGTGGCTGCTCTTGCTATCTCGGCTCACGCACAAAGAGATTTCAATGAACGACGAGGAAAGGAGAATGACACAGAGAGAGGACAAGGTGGCTTTGGAGGAAGGCCTGGTGGAATGCAGATGGGTGGTC 186  
9-1518    ----TAGCATCGGAGAGACCTATTACTAACATGGAGGTGAAAGTGACACTGATCGTTGCCATTGTGGCTGCTCTTGCTATCTCGGCTCACGCACAAAGAGATTTCAATGAACGACGAGGAAAGGAGAATGACACAGAGAGAGGACAAGGTGGCTTTGGAGGAAGGCCTGGTGGAATGCAGATGGGTGGTC 186  
9-1519    ----TAGCATCGGAGAGACCTATTACTAACATGGAGGTGAAAGTGACACTGATCGTTGCCATTGTGGCTGCTCTTGCTATCTCGGCTCACGCACAAAGAGATTTCAATGAACGACGAGGAAAGGAGAATGACACAGAGAGAGGACAAGGTGGCTTTGGAGGAAGGCCTGGTGGAATGCAGATGGGTGGTC 186  
9-1520    ----TAGCATCGGAGAGACCTATTACTAACATGGAGGTGAAAGTGACACTGATCGTTGCCATTGTGGCTGCTCTTGCTATCTCGGCTCACGCACAAAGAGATTTCAATGAACGACGAGGAAAGGAGAATGACACAGAGAGAGGACAAGGTGGCTTTGGAGGAAGGCCTGGTGGAATGCAGATGGGTGGTC 186  
9-1521    ----TAGCATCGGAGAGACCTATTACTAACATGGAGGTGAAAGTGACACTGATCGTTGCCATTGTGGCTGCTCTTGCTATCTCGGCTCACGCACAAAGAGATTTCAATGAACGACGAGGAAAGGAGAATGACACAGAGAGAGGACAAGGTGGCTTTGGAGGAAGGCCTGGTGGAATGCAGATGGGTGGTC 186  
9-1523    ----TAGCATCGGAGAGACCTATTACTAACATGGAGGTGAAAGTGACACTGATCGTTGCCATTGTGGCTGCTCTTGCTATCTCGGCTCACGCACAAAGAGATTTCAATGAACGACGAGGAAAGGAGAATGACACAGAGAGAGGACAAGGTGGCTTTGGAGGAAGGCCTGGTGGAATGCAGATGGGTGGTC 186  
9-1524    ------GCATCGGAGAGACCTATTACTAACATGGAGGTGAAAGTGACACTGATCGTTGCCATTGTGGCTGCTCTTGCTATCTCGGCTCACGCACAAAGAGATTTCAATGAACGACGAGGAAAGGAGAATGACACAGAGAGAGGACAAGGTGGCTTTGGAGGAAGGCCTGGTGGAATGCAGATGGGTGGTC 184  
9-1526    ----TAGCATCGGAGAGACCTATTACTAACATGGAGGTGAAAGTGACACTGATCGTTGCCATTGTGGCTGCTCTTGCTATCTCGGCTCACGCACAAAGAGATTTCAATGAACGACGAGGAAAGGAGAATGACACAGAGAGAGGACAAGGTGGCTTTGGAGGAAGGCCTGGTGGAATGCAGATGGGTGGTC 186  
9-1527    ----TAGCATCGGAGAGACCTATTACTAACATGGAGGTGAAAGTGACACTGATCGTTGCCATTGTGGCTGCTCTTGCTATCTCGGCTCACGCACAAAGAGATTTCAATGAACGACGAGGAAAGGAGAATGACACAGAGAGAGGACAAGGTGGCTTTGGAGGAAGGCCTGGTGGAATGCAGATGGGTGGTC 186  
9-1530    ----TAGCATCGGAGAGACCTATTACTAACATGGAGGTGAAAGTGACACTGATCGTTGCCATTGTGGCTGCTCTTGCTATCTCGGCTCACGCACAAAGAGATTTCAATGAACGACGAGGAAAGGAGAATGACACAGAGAGAGGACAAGGTGGCTTTGGAGGAAGGCCTGGTGGAATGCAGATGGGTGGTC 186  
9-1531    ----TAGCATCGGAGAGACCTATTACTAACATGGAGGTGAAAGTGACACTGATCGTTGCCATTGTGGCTGCTCTTGCTATCTCGGCTCACGCACAAAGAGATTTCAATGAACGACGAGGAAAGGAGAATGACACAGAGAGAGGACAAGGTGGCTTTGGAGGAAGGCCTGGTGGAATGCAGATGGGTGGTC 186  
9-1533    ----TAGCATCGGAGAGACCTATTACTAACATGGAGGTGAAAGTGACACTGATCGTTGCCATTGTGGCTGCTCTTGCTATCTCGGCTCACGCACAAAGAGATTTCAATGAACGACGAGGAAAGGAGAATGACACAGAGAGAGGACAAGGTGGCTTTGGAGGAAGGCCTGGTGGAATGCAGATGGGTGGTC 186  
9-1537    ----TAGCATCGGAGAGACCTACTACTAACATGGAGGTGAAAGTGACACTGATCGTTGCCATTGTGGCTGCTCTTGCTATCTCGGCTCACGCACAAAGAGATTTCAATGAACGACGAGGAAAGGAGAATGACACAGAGAGAGGACAAGGTGGCTTTGGAGGAAGGCCTGGTGGAATGCAGATGGGTGGTC 186  
9-1538    ----TAGCATCGGAGAGACCTATTACTAACATGGAGGTGAAAGTGACACTGATCGTTGCCATTGTGGCTGCTCTTGCTATCTCGGCTCACGCACAAAGAGATTTCAATGAACGACGAGGAAAGGAGAATGACACAGAGAGAGGACAAGGTGGCTTTGGAGGAAGGCCTGGTGGAATGCAGATGGGTGGTC 186  
9-1539    ----TAGCATCGGAGAGACCTATTACTAACATGGAGGTGAAAGTGACACTGATCGTTGCCATTGTGGCTGCTCTTGCTATCTCGGCTCACGCACAAAGAGATTTCAATGAACGACGAGGAAAGGAGAATGACACAGAGAGAGGACAAGGTGGCTTTGGAGGAAGGCCTGGTGGAATGCAGATGGGTGGTC 186  
9-1540    ----TAGCATCGGAGAGACCTATTACTAACATGGAGGTGAAAGTGACACTGATCGTTGCCATTGTGGCTGCTCTTGCTATCTCGGCTCACGCACAAAGAGATTTCAATGAACGACGAGGAAAGGAGAATGACACAGAGAGAGGACAAGGTGGCTTTGGAGGAAGGCCTGGTGGAATGCAGATGGGTGGTC 186  
9-1543    ----TAGCATCGGAGAGACCTATTACTAACATGGAGGTGAAAGTGACACTGATCGTTGCCATTGTGGCTGCTCTTGCTATCTCGGCTCACGCACAAAGAGATTTCAATGAACGACGAGGAAAGGAGAATGACACAGAGAGAGGACAAGGTGGCTTTGGAGGAAGGCCTGGTGGAATGCAGATGGGTGGTC 186  
9-1544    ----TAGCATCGGAGAGACCTATTACTAACATGGAGGTGAAAGTGACACTGATCGTTGCCATTGTGGCTGCTCTTGCTATCTCGGCTCACGCACAAAGAGATTTCAATGAACGACGAGGAAAGGAGAATGACACAGAGAGAGGACAAGGTGGCTTTGGAGGAAGGCCTGGTGGAATGCAGATGGGTGGTC 186  
9-1545    ----TAGCATCGGAGAGACCTATTACTAACATGGAGGTGAAAGTGACACTGATCGTTGCCATTGTGGCTGCTCTTGCTATCTCGGCTCACGCACAAAGAGATTTCAATGAACGACGAGGAAAGGAGAATGACACAGAGAGAGGACAAGGTGGCTTTGGAGGAAGGCCTGGTGGAATGCAGATGGGTGGTC 186  
9-1546    ----TAGCATCGGAGAGACCTATTACTAACATGGAGGTGAAAGTGACACTGATCGTTGCCATTGTGGCTGCTCTTGCTATCTCGGCTCACGCACAAAGAGATTTCAATGAACGACGAGGAAAGGAGAATGACACAGAGAGAGGACAAGGTGGCTTTGGAGGAAGGCCTGGTGGAATGCAGATGGGTGGTC 186  
9-1547    ----TAGCATCGGAGAGACCTATTACTAACATGGAGGTGAAAGTGACACTGATCGTTGCCATTGTGGCTGCTCTTGCTATCTCGGCTCACGCACAAAGAGATTTCAATGAACGACGAGGAAAGGAGAATGACACAGAGAGAGGACAAGGTGGCTTTGGAGGAAGGCCTGGTGGAATGCAGATGGGTGGTC 186  
9-1548    ----TAGCATCGGAGAGACCTATTACTAACATGGAGGTGAAAGTGACACTGATCGTTGCCATTGTGGCTGCTCTTGCTATCTCGGCTCACGCACAAAGAGATTTCAATGAACGACGAGGAAAGGAGAATGACACAGAGAGAGGACAAGGTGGCTTTGGAGGAAGGCCTGGTGGAATGCAGATGGGTGGTC 186  
9-1513    ----TAGCATCGGAGAGACCTATTACTAACATG---GTGAAAGTGACACTGATCGTTGCCATTGTGGCTGCTCTTGCTATCTCAGCTCACGCACAAAGAGATTACAATGAACTACGAGGAAATAAGAATGGCAGAGAGAGAGGACAAGGTCGCTTTGGAGGAAGGCCGGGTGGAATGCAGATGGGTGGAT 183  
9-1541    ----TAGCATCGGAGAGACCTATTACTAACATG---GTGAAAGTGACACTGATCGTTGCCATTGTGGCTGCTCTTGCTATCTCAGCTCACGCACAAAGAGATTACAATGAACTACGAGGAAATAAGAATGGCAGAGAGAGAGGACAAGGTCGCTTTGGAGGAAGGCCGGGTGGAATGCAGATGGGTGGAT 183  
9-1542    ----TAGCATCGGAGAGACCTATTACTAACATG---GTGAAAGTGACACTGATCGTTGCCATTGTGGCTGCTCTTGCTATCTCAGCTCACGCACAAAGAGATTACAATGAACTACGAGGAAATAAGAATGGCAGAGAGAGAGGACAAGGTCGCTTTGGAGGAAGGCCGGGTGGAATGCAGATGGGTGGAT 183  
9-1525    ----TAGCATCGGAGAGACCTATTACTAACATGGAGGTGAAAGTGACACTGATCGTTGCCATTGTGGCTGCTCTTGCTATCTCGGCTCACGCACAAAGCGATTTCAATGAACGACGAGGAAAGGAGAATGGCAGAGAGAGAGGACAAGATCGCTTTGGAGGAAGGCCTGATGGAATGCAGATGGGTGGAC 186  
9-1501    -----AGCATCGGAGAGACCTATTACTAACATGGAGGTGAAAGTGACACTGATCGTTGCCATTGTGGCTGCTCTTGCTATCTCGGCTCACGCACAAAGAGATTTCAATGAACGACGAGGAAAGGAGAATGACACAGAGAGAGGACAAGGTGGCTTTGGAGGAAGGCCTGGTGGAATGCAGATGGGTGGTC 185  
9-1502    ----TAGCATCGGAGAGACCTATTACTAACATGGAGGTGAAAGTGACACTGATCGTTGCCATTGTGGCTGCTCTTGCTATCTCGGCTCACGCACAAAGAGATTTCAATGAACGACGAGGAAAGGAGAATGACACAGAGAGAGGACAAGGTGGCTTTGGAGGAAGGCCTGGTGGAATGCAGATGGGTGGTC 186  
9-1508    ----TAGCATCGGAGAGACCTATTACTAACATGGAGGTGAAAGTGTCACTGATCGTTGCCATTGTGGCTGCTCTTGCTATCTCGGCTCACGCACAAAGAGATTTCAATGAACGACGAGGAAAGGAGAATGACACAGAGAGAGGACAAGGTGGCTTTGGAGGAAGGCCTGGTGGAATGCAGATGGGTGGTC 186  
9-1511    ----TAGCATCGGAGAGACCTATTACTAACATGGAGGTGAAAGTGACACTGATCGTTGCCATTGTGGCTGCTCTTGCTATCTCGGCTCACGTACAAAGAGATTTCAATGAACGACGAGGAAAGGAGAATGACACAGAGAGAGGACAAGGTGGCTTTGGAGGAAGGCCTGGTGGAATGCAGATGGGTGGTC 186  
9-1517    ----TAGCATCGGAGAGACCTATTACTAACATGGAGGTGAAAGTGACACTGATCGTTGCCATTGTGGCTGCTCTTGCTATCTCGGCTCACGCACAAAGAGATTTCAATGAACGACGAGGAAAGGAGAATGACACAGAGAGAGGACAAGGTGGCTTTGGAGGAAGGCCTGGTGGAATGCAGATGGGTGGTC 186  
9-1528    ----TAGCATCGGAGAGACCTATTACTAACATGGAGGTGAAAGTGACACTGATCGTTGCCATTGTGGCTGCTCTTGCTATCTCGGCTCACGCACAAAGAGATTTCAATGAACGACGAGGAAAGGAGAATGACACAGAGAGAGGACAAGGTGGCTTTGGAGGAAGGCCTGGTGGAATGCAGATGGGTGGTC 186  
9-1529    ----TAGCATCGGAGAGACCTATTACTAACATGGAGGTGAAAGTGACACTGATCGTTGCCATTGTGGCTGCTCTTGCTATCTCGGCTCACGCACAAAGAGATTTCAATGAACGACGAGGAAAGGAGAATGACACAGAGAGAGGACAAGGTGGCTTTGGAGGAAGGCCTGGTGGAATGCAGATGGGTGGTC 186  
9-1534    ----TAGCATCGGAGAGACCTATTACTAACATGGAGGTGAAAGTGACACTGATCGTTGCCATTGTGGCTGCTCTTGCTATCTCGGCTCACGCACAAAGAGATTTCAATGAACGACGAGGAAAGGAGAATGACACAGAGAGAGGACAAGGTGGCTTTGGAGGAAGGCCTGGTGGAATGCAGATGGGTGGTC 186  
9-1535    ----TAGCATCGGAGAGACCTATTACTAACATGGAGGTGAAAGTGACACTGATCGTTGCCATTGTGGCTGCTCTTGCTATCTCGGCTCACGCACAAAGAGATTTCAATGAACGACGAGGAAAGGAGAATGACACAGAGAGAGGACAAGGTGGCTTTGGAGGAAGGCCTGGTGGAATGCAGATGGGTGGTC 186  
9-2448    ----TAGCATCGGAGAGACCTATTACTAACATGGAGGTGAAAGTGACACTGATCGTTGCCATTGTGGCTGCTCTTGCTATCTCGGCTCACGCACAAAGAGATTTCAATGAACGACGAGGAAAGGAGAATGACACAGAGAGAGGACAAGGTGGCTTTGGAGGAAGGCCTGGTGGAATGCAGATGGGTGGTC 186  
9-2401    ----TAGCATCGGAGAGACCTATTACTAACATGGAGGTGAAAGTGACACTGATCGTTGCCATTGTGGCTGCTCTTGCTATCTCGGCTCACGCACAAAGAGATTTCAATGAACGACGAGGAAAGGAGAATGACACAGAGAGAGGACAAGGTGGCTTTGGAGGAAGGCCTGGTGGAATGCAGATGGGTGGTC 186  
9-2402    ----TAGCATCGGAGAGACCTATTACTAACATGGAGGTGAAAGTGACACTGATCGTTGCCATTGTGGCTGCTCTTGCTATCTCGGCTCACGCACAAAGAGATTTCAATGAACGACGAGGAAAGGAGAATGACACAGAGAGAGGACAAGGTGGCTTTGGAGGAAGGCCTGGTGGAATGCAGATGGGTGGTC 186  
9-2404    ----TAGCATCGGAGAGACCTATTACTAACATGGAGGTGAAAGTGACACTGATCGTTGCCATTGTGGCTGCTCTTGCTATCTCGGCTCACGCACAAAGAGATTTCAATGAACGACGAGGAAAGGAGAATGACACAGAGAGAGGACAAGGTGGCTTTGGAGGAAGGCCTGGTGGAATGCAGATGGGTGGTC 186  
9-2406    ----TAGCATCGGAGAGACCTATTACTAACACGGAGGTGAAAGTGACACTGATCGTTGCCATTGTGGCTGCTCTTGCTATCTCGGCTCACGCACAAAGAGATTTCAATGAACGACGAGGAAAGGAGAATGACACAGAGAGAGGACAAGGTGGCTTTGGAGGAAGGCCTGGTGGAATGCAGATGGGTGGTC 186  
9-2408    ----TAGCATCGGAGAGACCTATTACTAACATGGAGGTGAAAGTGACACTGATCGTTGCCATTGTGGCTGCTCTTGCTATCTCGGCTCACGCACAAAGAGATTTCAATGAACGACGAGGAAAGGAGAATGACACAGAGAGAGGACAAGGTGGCTTTGGAGGAAGGCCTGGTGGAATGCAGATGGGTGGTC 186  
9-2409    ----TAGCATCGGAGAGACCTATTACTAACATGGAGGTGAAAGTGACACTGATCGTTGCCATTGTGGCTGCTCTTGCTATCTCGGCTCACGCACAAAGAGATTTCAATGAACGACGAGGAAAGGAGAATGACACAGAGAGAGGACAAGGTGGCTTTGGAGGAAGGCCTGGTGGAATGCAGATGGGTGGTC 186  
9-2411    ----TAGCATCGGAGAGACCTATTACTAACATGGAGGTGAAAGTGACACTGATCGTTGCCATTGTGGCTGCTCTTGCTATCTCGGCTCACGCACAAAGAGATTTCAATGAACGACGAGGAAAGGAGAATGACACAGAGAGAGGACAAGGTGGCTTTGGAGGAAGGCCTGGTGGAATGCAGATGGGTGGTC 186  
9-2413    ----TAGCATCGGAGAGACCTATTACTAACATGGAGGTGAAAGTGACACTGATCGTTGCCATTGTGGCTGCTCTTGCTATCTCGGCTCACGCACAAAGAGATTTCAATGAACGACGAGAAAAGGAGAATGACACAGAGAGAGGACAAGGTGGCTTTGGAGGAAGGCCTGGTGGAATGCAGATGGGTGGTC 186  
9-2414    ----TAGCATCGGAGAGACCTATTACTAACATGGAGGTGAAAGTGACACTGATCGTTGCCATTGTGGCTGCTCTTGCTATCTCGGCTCACGCACAAAGAGATTTCAATGAACGACGAGGAAAGGAGAATGACACAGAGAGAGGACAAGGTGGCTTTGGAGGAAGGCCTGGTGGAATGCAGATGGGTGGTC 186  
9-2417    ----TAGCATCGGAGAGACCTATTACTAACATGGAGGTGAAAGTGACACTGATCGTTGCCATTGTGGCTACTCTTGCTATCTCGGCTCACGCACATAGAGATTTCAATGAACGACGAGGAAAGGAGAATGACACAGAGAGAGGACAAGGTGGCTTTGGAGGAAGGCCTGGTGGAATGCAGATGGGTGGTC 186  
9-2418    ----TAGCATCGGAGAGACCTATTACTAACATGGAGGTGAAAGTGACACTGATCGTTGCCATTGTGGCTGCTCTTGCTATCTCGGCTCACGCACAAAGAGATTTCAATGAACGACGAGGAAAGGAGAATGACACAGAGAGAGGACAAGGTGGCTTTGGAGGAAGGCCTGGTGGAATGCAGATGGGTGGTC 186  
9-2419    ----TAGCATCGGAGAGACCTATTACTAACATGGAGGTGAAAGTGACACTGATCGTTGCCATTGTGGCTGCTCTTGCTATCTCGGCTCACGCACAAAGAGATTTCAATGAACGACGAGGAAAGGAGAATGACACAGAGAGAGGACAAGGTGGCTTTGGAGGAAGGCCTGGTGGAATGCAGATGGGTGGTC 186  
9-2420    ----TAGCATCGGAGAGACCTATTACTAACATGGAGGTGAAAGTGACACTGATCGTTGCCATTGTGGCTGCTCTTGCTATCTCGGCTCACGCACAAAGAGATTTCAATGAACGACGAGGAAAGGAGAATGACACAGAGAGAGGACAAGGTGGCTTTGGAGGAAGGCCTGGTGGAATGCAGATGGGTGGTC 186  
9-2421    ----TAGCATCGGAGAGACCTATTACTAACATGGAGGTGAAAGTGACACTGATCGTTGCCATTGTGGCTGCTCTTGCTATCTCGGCTCACGCACAAAGAGATTTCAATGAACGACGAGGAAAGGAGAATGACACAGAGAGAGGACAAGGTGGCTTTGGAGGAAGGCCTGGTGGAATGCAGATGGGTGGTC 186  
9-2424    ----TAGCATCGGAGAGACCTATTACTAACATGGAGGTGAAAGTGACACTGATCGTTGCCATTGTGGCTGCTCTTGCTATCTCGGCTCACGCACAAAGAGATTTCAATGAACGACGAGGAAAGGAGAATGACACAGAGAGAGGACAAGGTGGCTTTGGAGGAAGGCCTGGTGGAATGCAGATGGGTGGTC 186  
9-2427    ----TAGCATCGGAGAGACCTATTACTAACATGGAGGTGAAAGTGACACTGATCGTTGCCATTGTGGCTGCTCTTGCTATCTCGGCTCACGCACAAAGAGATTTCAATGAACGACGAGGAAAGGAGAATGACACAGAGAGAGGACAAGGTGGCTTTGGAGGAAGGCCTGGTGGAATGCAGATGGGTGGTC 186  
9-2429    ----TAGCATCGGAGAGACCTATTACTAACATGGAGGTGAAAGTGACACTGATCGTTGCCATTGTGGCTGCTCTTGCTATCTCGGCTCACGCACAAAGAGATTTCAATGAACGACGAGGAAAGGAGAATGACACAGAGAGAGGACAAGGTGGCTTTGGAGGAAGGCCTGGTGGAATGCAGATGGGTGGTC 186  
9-2430    ----TAGCATCGGAGAGACCTATTACTAACATGGAGGTGAAAGTGACACTGATCGTTGCCATTGTGGCTGCTCTTGCTATCTCGGCTCACGCACAAAGAGATTTCAATGAACGACGAGGAAAGGAGAATGACACAGAGAGAGGACAAGGTGGCTTTGGAGGAAGGCCTGGTGGAATGCAGATGGGTGGTC 186  
9-2433    ----TAGCATCGGAGAGACCTATTACTAACATGGAGGTGAAAGTGACACTGATCGTTGCCATTGTGGCTGCTCTTGCTATCTCGGCTCACGCACAAAGAGATTTCAATGAACGACGAGGAAAGGAGAATGACACAGAGAGAGGACAAGGTGGCTTTGGAGGAAGGCCTGGTGGAATGCAGATGGGTGGTC 186  
9-2435    ----TAGCATCGGAGAGACCTATTACTAACATGGAGGTGAAAGTGACACTGATCGTTGCCATTGTGGCTGCTCTTGCTATCTCGGCTCACGCACAAAGAGATTTCAATGAACGACGAGGAAAGGAGAATGACACAGAGAGAGGACAAGGTGGCTTTGGAGGAAGGCCTGGTGGAATGCAGATGGGTGGTC 186  
9-2438    ----TAGCATCGGAGAGACCTATTACTAACATGGAGGTGAAAGTGACACTGATCGTTGCCATTGTGGCTGCTCTTGCTATCTCGGCTCACGCACAAAGAGATTTCAATGAACGACGAGGAAAGGAGAATGACACAGAGAGGGGACAAGGTGGCTTTGGAGGAAGGCCTGGTGGAATGCAGATGGGTGGTC 186  
9-2439    ----TAGCATCGGAGAGACCTATTACTAACATGGAGGTGAAAGTGACACTGATCGTTGCCATTGTGGCTGCTCTTGCTATCTCGGCTCACGCACAAAGAGATTTCAATGAACGACGAGGAAAGGAGAATGACACAGAGAGAGGACAAGGTGGCTTTGGAGGAAGGCCTGGTGGAATGCAGATGGGTGGTC 186  
9-2440    ----TAGCATCGGAGAGACCTATTACTAACATGGAGGTGAAAGTGACACTGATCGTTGCCATTGTGGCTGCTCTTGCTATCTCGGCTCACGCACAAAGAGATTTCAATGAACGACGAGGAAAGGAGAATGACACAGAGAGAGGACAAGGTGGCTTTGGAGGAAGGCCTGGTGGAATGCAGATGGGTGGTC 186  
9-2441    ----TAGCATCGGAGAGACCTATTACTAACATGGAGGTGAAAGTGACACTGATCGTTGCCATTGTGGCTGCTCTTGCTATCTCGGCTCACGCACAAAGAGATTTCAATGAACGACGAGGAAAGGAGAATGACACAGAGAGAGGACAAGGTGGCTTTGGAGGAAGGCCTGGTGGAATGCAGATGGGTGGTC 186  
9-2442    ----TAGCATCGGAGAGACCTATTACTAACATGGAGGTGAAAGTGACACTGATCGTTGCCATTGTGGCTGCTCTTGCTATCTCGGCTCACGCACAAAGAGATTTCAATGAACGACGAGGAAAGGAGAATGACACAGAGAGAGGACAAGGTGGCTTTGGAGGAAGGCCTGGTGGAATGCAGATGGGTGGTC 186  
9-2443    ----TAGCATCGGAGAGACCTATTACTAACATGGAGGTGAAAGTGACACTGATCGTTGCCATTGTGGCTGCTCTTGCTATCTCGGCTCACGCACAAAGAGATTTCAATGAACGACGAGGAAAGGAGAATGACACAGAGAGAGGACAAGGTGGCTTTGGAGGAAGGCCTGGTGGAATGCAGATGGGTGGTC 186  
9-2445    ----TAGCATCGGAGAGACCTATTACTAACATGGAGGTGAAAGTGACACTGATCGTTGCCATTGTGGCTGCTCTTGCTATCTCGGCTCACGCACAAAGAGATTTCAATGAACGACGAGGAAAGGAGAATGACACAGAGAGAGGACAAGGTGGCTTTGGAGGAAGGCCTGGTGGAATGCAGATGGGTGGTC 186  
9-2446    ----TAGCATCGGAGAGACCTATTACTAACATGGAGGTGAAAGTGACACTGATCGTTGCCATTGTGGCTGCTCTTGCTATCTCGGCTCACGCACAAAGAGATTTCAATGAACGACGAGGAAAGGAGAATGACACAGAGAGAGGACAAGGTGGCTTTGGAGGAAGGCCTGGTGGAATGCAGATGGGTGGTC 186  
9-2416    ----TAGCATCGGAGAGACCTATTACTAACATG---GTGAAAGTGACACTGATCGTTGCCATTGTGGCTGCTCTTGCTATCTCAGCTCACGCACAAAGAGATTACAATGAACTACGAGGAAATAAGAATGGCAGAGAGAGAGGACAAGGTCGCTTTGGAGGAAGGCCGGGTGGAATGCAGATGGGTGGAT 183  
9-2423    ----TAGCATCGGAGAGACCTATTACTAACATG---GTGAAAGTGACACTGATCGTTGCCATTGTGGCTGCTCTTGCTATCTCAGCTCACGCACAAAGAGATTACAATGAACTACGAGGAAATAAGAATGGCAGAGAGAGAGGACAAGGTCGCTTTGGAGGAAGGCCGGGTGGAATGCAGATGGGTGGAT 183  
9-2403    ----TAGCATCGGAGAGACCTATTACTAACATGGAGGTGAAAGTGACACTGATCGTTGCCATTGTGGCTGCTCTTGCTATCTCGGCTCACGCACAAAGAGATTTCAATGAACGACGAGGAAAGGAGAATGACACAGAGAGAGGACAAGGTGGCTTTGGAGGAAGGCCTGGTGGAATGCAGATGGGTGGTC 186  
9-2415    ----TAGCATCGGAGAGACCTATTACTAACATGGAGGTGAAAGTGACACCGATCGTTGCCATTGTGGCTGCTCTTGCTATCTCGGCTCACGCACAAAGAGATTTCAATGAACGACGAGGAAAGGAGAATGACACAGAGAGAGGACAAGGTGGCTTTGGAGGAAGGCCTGGTGGAATGCAGATGGGTGGTC 186  
9-2422    ------TAGCATCGGAGAGACCTTACAAACATGGAGGTGAAAGTGACACTGATCGTTGCCATTGTGGCTGCTCTTGCTATCTCGGCTCACGCACAAAGAGATTTCAATGAACGACGAGGAAAGGAGAATGACACAGAGAGAGGACAAGGTGGCTTTGGAGGAAGGCCGGGTGGAATGCAGATGGGTGGTC 184  
9-2428    ----TAGCATCGGAGAGACCTATTACTAACATGGAGGTGAAAGTGACACTGATCGTTGCCATTGTGGCTGCTCTTGCTATCTCGGCTCACGCACAAAGAGATTTCAATGAACGACGAGGAAAGGAGAATGACACAGAGAGAGGACAAGGTGGCTTTGGAGGAAGGCCTGGTGGAATGCAGATGGGTGGTC 186  
9-2431    ----TAGCATCGGAGAGACCTATTACTAACATGGAGGTGAAAGTGACACTGATCGTTGCCATTGTGGCTGCTCTTGCTATCTCGGCTCACGCACAAAGAGATTTCAATGAACGACGAGGAAAGGAGAATGACACAGAGAGAGGACAAGGTGGCTTTGGAGGAAGGCCTGGTGGAATGCAGATGGGTGGTC 186  
9-2432    ----TAGCATCGGAGAGACCTATTACTAACATGGAGGTGAAAGTGACACTGATCGTTGCCATTGTGGCTGCTCTTGCTATCTCGGCTCACGCACAAAGAGATTTCAATGAACGACGAGGAAAGGAGAATGACACAGAGAGAGGACAAGGTGGCTTTGGAGGAAGGCCTGGTGGAATGCAGATGGGTGGTC 186  
9-2434    ----TAGCATCGGAGAGACCTATTACTAACATGGAGGTGAAAGTGACACTGATCGTTGCCATTGTGGCTGCTCTTGCTATCTCGGCTCACGCACAAAGAGATTTCAATGAACGACGAGGAAAGGAGAATGACACAGAGAGAGGACAAGGTGGCTTTGGAGGAAGGCCTGGTGGAATGCAGATGGGTGGTC 186  
9-2437    ----TAGCATCGGAGAGACCTATTACTAACATGGAGGTGAAAGTGACACTGATCGTTGCCATTGTGGCTGCTCTTGCTATCTCGGCTCACGCACAAAGAGATTTCAATGAACGACGAGGAAAGGAGAATGACACAGAGAGAGGACAAGGTGGCTTTGGAGGAAGGCCTGGTGGAATGCAGATGGGTGGTC 186  
9-2444    ----TAGCATCGGAGAGACCTATTACTAACATGGAGGTGAAAGTGACACTGATCGTTGCCATTGTGGCTGCTCTTGCTATCTCGGCTCACGCACAAAGAGATTTCAATGAACGACGAGGAAAGGAGAATGACACAGAGAGAGGACAAGGTGGCTTTGGAGGAAGGCCTGGTGGAATGCAGATGGGTGGTC 186  
4-1504    ----TAGCATCGGAGAGACCTATTACTAACATG---GTGAAAGTGACACTGATCGTTGCCATTGTGGCTGCTCTTGCTATCTCAGCTCACGCACAAAGAGATTACAATGAACTACGAGGAAATAAGAATGGCAGAGAGAGAGGACAAGGTCGCTTTGGAGGAAGGCCGGGTGGAATGCAGATGGGTGGAT 183  
4-1522    ----TAGCATCGGAGAGACCTATTACTAACATGGAGGTGAAAGTGACACTGATCGTTGCCATTGTGGCTGCTCTTGCTATCTCGGCTCACGCACAAAGAGATTTCAATGAACGACGAGGAAAGGAGAATGACACAGAGAGAGGACAAGGTGGCTTTGGAGGAAGGCCTGGTGGAATGCAGATGGGTGGTC 186  
4-1507    ----TAGCATCGGAGAGACCTATTACTAACATGGAGGTGAAAGTGACACTGATCGTTGCCATTGTGGCTGCTCTTGCTATCTCGGCTCACGCACAAAGAGATTTCAATGAACGACGAGGAAAGGAGAATGACACAGAGAGAGGACAAGGTGGCTTTGGAGGAAGGCCTGGTGGAATGCAGATAGGTGGTC 186  
4-1519    ----TAGCATCGGAGAGACCTATTACTAACATGGAGGTGAAAGTGACACTGATCGTTGCCATTGTGGCTGCTCTTGCTATCTCGGCTCACGCACAAAGAGATTTCAATGAACGACGAGGAAAGGAGAATGACACAGAGAGAGGACAAGGTGGCTTTGGAGGAAGGCCTGGTGGAATGCAGATGGGTGGTC 186  
4-1529    ----TAGCATCGGAGAGACCTATTACTAACATGGAGGTGAAAGTGACACTGATCGTTGCCATTGTGGCTGCTCTTGCTATCTCGGCTCACGCACAAAGAGATTTCAATGAACGACGAGGAAAGGAGAATGACACAGAGAGAGGACAAGGTGGCTTTGGAGGAAGGCCTGGTGGAATGCAGATGGGTGGTC 186  
4-1549    ----TAGCATCGGAGAGACCTATTACTAACATGGAGGTGAAAGTGACACTGATCGTTGCCATTGTGGCTGCTCTTGCTATCTCGGCTCACGCACAAAGAGATTTCAATGAACGACGAGGAAAGGAGAATGACACAGAGAGAGGACAAGGTGGCTTTGGAGGAAGGCCTGGTGGAATGCAGATGGGTGGTC 186  
4-1510    --TATAGCATCGGAGAGACCTATTACTAACATGGAGGTGAAAGTGACACTGATCGTTGCCATTGTGGCTGCTCTTGCTATCTCGGCTCACGCACAAAGAGATTTCAATGAACGACGAGGAAAGGAGAATGACACAGAGAGAGGACAAGGTGGCTTTGGAGGAAGGCCTGGTGGAATGCAGATGGGTGGTC 188  
4-1539    ----TAGCATCGGAGAGACCTATTACTAACATGGAGGTGAAAGTGACACTGATCGTTGCCATTGTGGCTGCTCTTGCTATCTCGGCTCACGCACAAAGAGATTTCAATGAACGACGAGGAAAGGAGAATGACACAGAGAGAGGACAAGGTGGCTTTGGAGGAAGGCCTGGTGGAATGCAGATGGGTGGTC 186  
4-2424    -----------------------------CATG---GTGAAAGTGACACTGATCGTTGCCATTGTGGCTGCTCTTGCTATCTCGGCTCACGCACGAAGAGATTTCAATGAACGACGAGGAAAGGAGAATGGCAGAGAGAGAGGACAAGGTGGCTTTGGAGGAAGGCCTGGTGGAATGCAGACGGGTAGTC 158  
4-2401    ------TAGCATCGGAGAGACCTTACTAACATGGAGGTGAAAGTGACACTGATCGTTGCCATTGTGGCTGCTCTTGCTATCTCGGCTCACGCACAAAGAGATTTCAATGAACGACGAGGAAAGGAGAATGACACAGAGAGAGGACAAGGTGGCTTTGGAGGAAGGCCTGGTGGAATGCAGATGGGTGGTC 184  
4-2405    ------TAGCATCGGAGAGACCTTACAAACATGGAGGTGAAAGTGACACTGATCGTTGCCATTGTGGCTGCTCTTGCTATCTCGGCTCACGCACAAAGAGATTTCAATGAACGACGAGGAAAGGAGAATGACACAGAGAGAGGACAAGGTGGCTTTGGAGGAAGGCCTGGTGGAATGCAGATGGGTAGTC 184  
4-2407    ------TAGCATCGGAGAGACCTTACTAACATGGAGGTGAAAGTGACACTGATCGTTGCCATTGTGGCTGCTCTTGCTATCTCGGCTCACGCACAAAGAGATTTCAATGAACGACGAGGAAAGGAGAATGACACAGAGAGAGGACAAGGTGGCTTTGGAGGAAGGCCTGGTGGAATGCAGATGGGTGGTC 184  
4-2408    ------TAGCATCGGAGAGACCTTACTAACATGGAGGTGAAAGTGACACTGATCGTTGCCATTGTGGCTGCTCTTGCTATCTCGGCTCACGCACAAAGAGATTTCAATGAACGACAAGGAAAGGAGAATGACACAGAGAGAGGACAAGGTGGCTTTGGAGGAAGGCCTGGTGGAATGCAGATGGGTGGTC 184  
4-2411    -------AGCATCGGAGAGACCTTACAAACATGGAGGTGAAAGTGACACTGATCGTTGCCATTGTGGCTGCTCTTGCTATCTCGGCTCACGCACAAAGAGATTTCAATGAACGACGAGGAAAGGAGAATGACACAGAGAGAGGACAAGGTGGCTTTGGAGGAAGGCCTGGTGGAATGCAGATGGGTAGTC 183  
4-2417    ------TAGCATCGGAGAGACCTTACTAACATGGAGGTGAAAGTGACACTGATCGTTGCCATTGTGGCTGCTCTTGCTATCTCGGCTCACGCACAAAGAGATTTCAATGAACGACGAGGAAAGGAGAATGACGCAGAGAGAGGACAAGGTGGCTTTGGAGGAAGGCCTGGTGGAATGCAGATGGGTGGTC 184  
4-2418    ------TAGCATCGGAGAGACCTTACTAACATGGAGGTGAAAGTGACACTGATCGTTGCCATTGTGGCTGCTCTTGCTATCTCGGCTCACGCACAAAGAGATTTCAATGAACGACGAGGAAAGGAGAATGACACAGAGAGAGGACAAGGTGGCTTTGGAGGAAGGCCTGGTGGAATGCAGATGGGTGGTC 184  
4-2419    --------GCATCGGAGAGACCTTACTAACATGGAGGTGAAAGTGACACTGATCGTTGCCATTGTGGCTGCTCTTGCTATCTCGGCTCACGCACAAAGAGATTTCAATGAACGACGAGGAAAGGAGAATGACACAGAGAGAGGACAAGGTGGCTTTGGAGGAAGGCCTGGTGGAATGCAGATGGGTGGTC 182  
4-2421    ------TAGCATCGGAGAGACCTTACTAACATGGAGGTGAAAGTGACACTGATCGTTGCCATTGTGGCTGCTCTTGCTATCTCGGCTCACGCACAAAGAGATTTCAATGAACGACGAGGAAAGGAGAATGACACAGAGAGAGGACAAGGTGGCTTTGGAGGAAGGCCTGGTGGAATGCAGATGGGTGGTC 184  
4-2422    ------TAGCATCGGAGAGACCTTACTAACATGGAGGTGAAAGTGACACTGATCGTTGCCATTGTGGCTGCTCTTGCTATCTCGGCTCACGCACAAAGAGATTTCAATGAACGACGAGGAAAGGAGAATGACACAGAGAGAGGACAAGGTGGCTTTGGAGGAAGGCCTGGTGGAATGCAGATGGGTGGTC 184  
4-2426    ------TAGCATCGGAGAGACCTTACTAACATGGAGGTGAAAGTGACACTGATCGTTGCCATTGTGGCTGCTCTTGCTATCTCGGCTCACGCACAAAGAGATTTCAATGAACGACGAGGAAAGGAGAATGACACAGAGAGAGGACAAGGTGGCTTTGGAGGAAGGCCTGGTGGAATGCAGATGGGTGGTC 184  
4-2428    ------TAGCATCGGAGAGACCTTACTAACATGGAGGTGAAAGTGACACTGATCGTTGCCATTGTGGCTGCTCTTGCTATCTCGGCTCACGCACAAAGAGATTTCAATGAACGACGAGGAAAGGAGAATGACACAGAGAGAGGACAAGGTGGCTTTGGAGGAAGGCCTGGTGGAATGCAGATGGGTGGTC 184  
4-2429    ------TAGCATCGGAGAGACCTTACTAACATGGAGGTGAAAGTGACACTGATCGTTGCCATTGTGGCTGCTCTTGCTATCTCGGCTCACGCACAAAGAGATTTCAATGAACGACGAGGAAAGGAGAATGACACAGAGAGAGGACAAGGTGGCTTTGGAGGAAGGCCTGGTGGAATGCAGATGGGTGGTC 184  
4-2435    ------TAGCATCGGAGAGACCTTACAAACATGGAGGTGAAAGCAACATTGATCGTTGCCATTGTGGCTGCTCTTGCTATCTCGGCTCACGCACGAAGAGATTTCAATGAACGGCGAGGAAATGAGAATGGCAGAGAGAGAGGACAAGGTCGCTTTGGAGGAAGGCCTGGTGGAATGCAGATGGGTGGAT 184  
4-2437    ----TAGCATCGGAGAGACCTATTACTAACATGGAGGTGAAAGTGACACTGATCGTTGCCATTGTGGCTGCTCTTGCTATCTCGGCTCACGCACAAAGAGATTTCAATGAACGACGAGGAAAGGAGAATGACACAGAGAGAGGACAAGGTGGCTTTGGAGGAAGGCCTGGTGGAATGCAGATGGGTGGTC 186  
4-2444    ------TAGCATCGGAGAGACCTTACTAACATGGAGGTGAAAGTGACACTGATCGTTGCCATTGTGGCTGCTCTTGCTATCTCGGCTCACGCACAAAGAGATTTCAATGAACGACGAGGAAAGGAGAATGACACAGAGAGAGGACAAGGTGGCTTTGGAGGAAGGCCTGGTGGAATGCAGATGGGTGGTC 184  
4-2445    ------TAGCATCGGAGAGACCTTACTAACATGGAGGTGAAAGTGACACTGATCGTTGCCATTGTGGCTGCTCTTGCTATCTCGGCTCACGCACAAAGAGATTTCAATGAACGACGAGGAAAGGAGAATGACACAGAGAGAGGACAAGGTGGCTTTGGAGGAAGGCCTGGTGGAATGCAGATGGGTGGTC 184  
4-2447    ------TAGCATCGGAGAGACCTTACTAACATGGAGGTGAAAGTGACACTGATCGTTGCCATTGTGGCTGCTCTTGCTATCTCGGCTCACGCACAAAGAGATTTCAATGAACGACGAGGAAAGGAGAATGACACAGAGAGAGGACAAGGTGGCTTTGGAGGAAGGCCTGGTGGAATGCAGATGGGTGGTC 184  
4-2448    --------------AGAACCATCCACAAACATGGAGGTGAAAGTGACACTGATCGTTGCCATTGTGGCTGCTCTTGCTATCTCGGCTCACGCACAAAGAGATTTCAATGAACGACGAGGAAAGGAGAATGACACAGAGAGAGGACAAGGTGGCTTTGGAGGAAGGCCTGGTGGAATGCAGATGGGTGGTC 176  
4-2404    -----AGCATCGGAGAGACCTATTACTAACATG---GTGAAAGTGACACTGATCGTTGCCATTGTGGCTGCTCTTGCTATCTCAGCTCACGCACAAAGAGATTACAATGAACTACGAGGAAATAAGAATGGCAGAGAGAGAGGACAAGGTCGCTTTGAAGGAAGGCAGGGTGGAATGCAGATGGTAGGAT 182  
4-2413    ----TAGCATCGGAGAGACCTATTACTAACATG---GTGAAAGTGACACTGATCGTTGCCATTGTGGCTGCTCTTGCTATCTCAGCTCACGCACAAAGGGATTACAATGAACTACGAGGAAATAAGAATGGCAGAGAGAGAGGACAAGGTCGCTTTGGAGGAAGGCCGGGTGGAATGCAGATGGGTGGAT 183  
4-2450    ----TAGCATCGGAGAGACCTATTACTAACATG---GTGAAAGTGACACTGATCGTTGCCATTGTGGCTGCTCTTGCTATCTCAGCTCACGCACAAAGAGATTACAATGAACTACGAGGAAATAAGAATGGCAGAGAGAGAGGACAAGGTCGCTTTGGAGGAAGGCCGGGTGGAATGCAGATGGGTGGAT 183  
4-2420    ------TAGACTTCTAGAGACCTACCCAACATGGAGGTGAAAGTGACACTGATCTTTGCCATTGTGGCTGCTCTTGCTATCTCGGCTCACGCACGAAGAGATTACAATGAACGACGAGGAAATGAGAATGGCAGAGAGAGAGGACAAGGTCGCTTTGGAGGAAGGCCTGGTGGAATGCAGATGGGTGGAC 184  
4-2438    ------TAGCATCGGAGAGACCTTACAAACATGGAGGTGAAAGTGACACTGATCGTTGCCATTGTGGCTGCTCTTGCTATCTCAGCTCACGCACAAAGAGATTACAATGAACTACGAGGAAATAAGAATGGCAGAGAGAGAGGACAAGGTCGCTTTGGAGGAAGGCCGGGTGGAATGCAGATGGGTGGAT 184  
4-2430    ------TAGCATCGGAGAGACCTTACTAACATGGAGGTGAAAGTGACACTGATCGTTGCCATTGTGGCTGCTCTTGCTATCTCGGCTCACGCACAAAGAGATTTCAATGAACGACGAGGAAAGGAGAATGACACAGAGAGAGGACAAGGTGGCTTTGGAGGAAGGCCTGGTGGAATGCAGATGGGTGGTC 184  
4-2439    ------TAGCATCGGAGAGACCTTACTAACATGGAGGTGAAAGTGACACTGATCGTTGCCATTGTGGCTGCTCTTGCTATCTCGGCTCACGCACAAAGAGATTTCAATGAACGACGAGGAAAGGAGAATGACACAGAGAGAGGACAAGGTGGCTTTGGAGGAAGGCCTGGTGGAATGCAGATGGGTGGTC 184  
4-2433    ------TAGCATCGGAGAGACCTTACAAACATGGAGGTGAAAGCAACATTGATCGTTGCCATTGTGGCTGCTCTTGCTATCTCGGCTCACGCACGAAGAGATTTCAATGAACGGCGAGGAAATGAGAATGGCAGAGAGAGAGGACAAGGTCGCTTTGGAGGAAGGCCTGGTGGAATGCAGATGGGTGGAT 184  
4-2441    ------TAGCATCGGAGAGACCTTACAAACATGGAGGTGAAAGCAACATTGATCGTTGCCATTGTGGCTGCTCTTGCTATCTCGGCTCACGCACGAAGAGATTTCAATGAACGGCGAGGAAATGAGAATGGCAGAGAGAGAGGACAAGGTCGCTTTGGAGGAAGGCCTGGTGGAATGCAGATGGGTGGAT 184  
4-2406    ------TAGCATCGGAGAGACCTTACAAACATGGAGGTGAAAGCAACACTGATCGTTGCCATTCTGGCTGTTCTTGCTATCTCGGCTCACGCACGAAGAGATTTCAATGAACTACGAGGAAAGGAGAATGGCAGAGAGAGAGGACAAGGCCGCTTTGGAGGAAGGCCTGATGGAATGCAGATGGGTGGAC 184  
4-2425    ----TAGCATCGGAGAGACCTATTACTATCATG---GTGAAAGTGACACTGATCGTTGCCATTGTGGCTGCTCTTGCTATCTCGGCTCACGCACGAAGAGATTTCAATGAACGACGAGGAAAGGAGAATGGCAGAGAGAGAGGACAAGGTGGCTTTGGAGGAAGGCCTGGTGGAATGCAGACGGGTAGTC 183  
4-2432    ----TAGCATCGGAGAGACCTATTACTATCATG---GTGAAAGTGACACTGATCGTTGCCATTGTGGCTGCTCTTGCTATCTCGGCTCACGCACGAAGAGATTTCAATGAACGACGAGGAAAGGAGAATGGCAGAGAGAGAGGACAAGGTGGCTTTGGAGGAAGGCCTGGTGGAATGCAGACGGGTAGTC 183  
4-2440    ----TAGCATCGGAGAGACCTATTACTATCATG---GTGAAAGTGACACTGATCGTTGCCATTGTGGCTGCTCTTGCTATCTCGGCTCACGCACGAAGAGATTTCAATGAACGACGAGGAAAGGAGAATGGCAGAGAGAGAGGACAAGGTGGCTTTGGAGGAAGGCCTGGTGGAATGCAGACGGGTAGTC 183  
4-2402    ------TAGCATCGGAGAGACCTTACAAACATGGAGGTGAAAGTGACACTGATCGTTGCCATTGTGGCTGCTCTTGCTATCTCGGCTCACGCACAAAGAGATTTCAATGAACGACGGAAA---------------------------------------------------------------------- 114  
4-2442    ----TAGCATCGGAGAGACCT------------------------------------------------------------------------------------------------------------------------------------------------------------------------- 17   
4-2443    ----TAGCATCGGAGAGACCTATTACTATCATG---ATGAAAGTGACACTGATCGTTGCCATTGTGGCTGCTCTTGCTATCTCGGCTCACGCACGAAGAGATTTCAATGAACGACGAGGAAAGGAGAATGGCAGAGAGAGAGGACAAGGTGGCTTTGGAGGAAGGCCTGGTGGAATGCAGACGGGTAGTC 183  
4-2416    ----TAGCATCGGAGAGACCTATTACTATCATG---GTGAAAGTGACACTGATCGTTGCCATTGTGGCTGCTCTTGCTATCTCGGCTCACGCACGAAGAGATTTCAATGAACGACGAGGAAAGGAGAATGGCAGAGAGAGAGGACAAGGTGGCTTTGGAGGAAGGCCTGGTGGAATGCAGACGGGTAGTC 183  
4-2446    ----TAGCATCGGAGAGACCTATTACTAACATGGAGGTGAAAGTGACACTGATCGTTGCCATTGTGGCTGCTCTTGCTATCTCGGCTCACGCACAAAGAGATTTCAATGAACGACGAGGAAAGGAGAATGACACAGAGAGAGGACAAGGTGGCTTTGGAGGAAGGCCTGGTGGAATGCAGATGGGTGGTC 186  
4-2403    ----TAGCATCGGAGAGACCTATTACTAACATGGAGGTGAAAGTGACACTGATCGTTGCCATTGTGGCTGCTCTTGCTATCTCGGCTCACGCACAAAGAGATTTCAATGAACGACGAGGAAAGGAGAATGACACAGAGAGAGGACAAGGTGGCTTTGGAGGAAGGCCTGGTGGAATGCAGATGGGTGGTC 186  
4-2423    ----TAGCATCGGAGAGACCTATTACTAACATGGAGGTGAAAGTGACACTGATCGTTGCCATTGTGGCTGCTCTTGCTATCTCGGCTCACGCACAAAGAGATTTCAATGAACGACGAGGAAAGGAGAATGACACAGAGAGAGGACAAGGTGGCTTTGGAGGAAGGCCTGGTGGAATGCAGATGGGTGGTC 186  
4-2409    ----TAGCATCGGAGAGACCTATTACTAACATGGAGGTGAAAGTGACACTGATCGTTGCCATTGTGGCTGCTCTTGCTATCTCGGCTCACGCACAAAGAGATTTCAATGAACGACGAGGAAAGGAGAATGACACAGAGAGAGGACAAGGTGGCTTTGGAGGAAGGCCTGGTGGAATGCAGATGGGTGGTC 186  
4-2434    ----TAGCATCGGAGAGACCTATTACTAACATGGAGGTGGAAGTGACACTGATCGTTGCCATTGTGGCTGCTCTTGCTATCTCGGCTCACGCACAAAGAGATTTCAATGAACGACGAGGAAAGGAGAATGACACAGAGAGAGGACAAGGTGGCTTTGGAGGAAGGCCTGGTGGAATGCAGATGGGTGGTC 186  
5-1502    ------TAGCATCGGAGAGACCTTACTAACATGGAGGTGAAAGTGACACTGATCGTTGCCATTGTGGCTGCTCTTGCTATCTCGGCTCACACACAAAGAGATTACAATGAACGACGAGGAAATGAGAATGGCAGAGAGAGAGGACAAGGTCGCTTTGGAGGAAGGCCTGGTGGAATGCAGATGGGTGGAC 184  
5-1503    ------TAGCATCGGAGAGACCTTACTAACATGGAGGTGAAAGTGACACTGATCGTCGCCATTGTGGCCGCTCTTGCTATCTCGGCTCACACACAAAGAGATTACAATGAACGACGAGGAAATGAGAATGGCAGAGAGAGAGGACAAGGTCGCTTTGGAGGAAGGCCTGGTGGAATGCAGATGGGTGGAC 184  
5-1504    ------TAGCATCGGAGAGACCTTACTAACATGGAGGTGAAAGTGACACTGATCGTTGCCATTGTGGCTGCTCTTGCTATCTCGGCTCACACACAAAGAGATTACAATGAACGACGAGGAAATGAGAATGGCAGAGGGAGAGGACAAGGTCGCTTTGGAGGAAGGCCTGGTGGAATGCAGATGGGTGGAC 184  
5-1505    ------TAGACTCGGAGAGACCTTACTAACATGGAGGTGAAAGTGACACTGATCGTTGCCATTGTGGCTGCTCTTGCTATCTCGGCTCACACACATAGAGATTACAATGAACGACGAGGAAATGAGAATGGCAGAGAGAGAGGACAAGGTCGCTTTGGAGGAAGGCCTGGTGGAATGCAGATGGGTGGAC 184  
5-1507    -----TAGACTCGTGAGAGACCTTACTAACATGGAGGTGAAAGTGACACTGATCGTTGCCATTGTGGCTGCTCTTGCTATCTCGGCTCACACACAAAGAGATTACAATGAACGACGAGGAAATGAGAATGGCAGAGAGAGAGGACAAGGTCGCTTTGGAGGAAGGCCTGGTGGAATGCAGATGGGTGGAC 185  
5-1509    -----TAGACATCGGAGAGACCTTACTAACATGGAGGTGAAAGTGACACTGATCGTTGCCATTGTGGCTGCTCTTGCTATCTCGGCTCACACACAAAGAGATTACAATGAACGACGAGGAAATGAGAATGGCAGAGAGAGAGGACAAGGTCGCTTTGGAGGAAGGCCTGGTGGAATGCAGATGGGTGGAC 185  
5-1511    -----TAGACATCGGAGAGACCTTACTAACATGGAGGTGAAAGTGACACTGATCGTTGCCATTGTGGCTGCTCTTGCTATCTCGGCTCACACACAAAGAGATTACAATGAACGACGAGGAAATGAGAATGGCAGAGAGAGAGGACAAGGTCGCTTTGGAGGAAGGCCTGGTGGAATGCAGATGGGTGGAC 185  
5-1513    ----TAGACATCGTGAGAGACCTTACTAACATGGAGGTGAAAGTGACACTGATCGTTGCCATTGTGGCTGCTCTTGCTATCTCGGCTCACACACAAAGAGATTACAATGAACGACGAGGAAATGAGAATGGCAGAGAGAGAGGACAAGGTCGCTTTGGAGGAAGGCCTGGTGGAATGCAGATGGGTGGAC 186  
5-1514    -----TAGACTCTGGAGAGACCTTACTAACATGGAGGTGAAAGTGACACTGATCGTTGCCATTGTGGCTGCTCTTGCTATCTCGGCTCACACACAAAGAGATTACAATGAACGACGAGGAAATGAGAATGGCAGAGAGAGAGGACAAGGTCGCTTTGGAGGAAGGCCTGGTGGAATGCAGATGGGTGGAC 185  
5-1508    ------TAGCATCGGAGAGACCTTACTAACATGGAGGTGGAAGTGACACTGATCGTTGCCATTGTGGCTGCTCTTGCTATCTCGGCTCACACACAAAGAGATTACAATGAACGACGAGGAAATGAGAATGGCAGAGAGAGAGGACAAGGTCGCTTTGGAGGAAGGCCTGGTGGAATGCAGATGGGTGGAC 184  
5-1510    ------TAGCATCGGAGAGACCTTACTAACATGGAGGTGAAAGTGACACTGATCGTTGCCATTGTGGCTGCTCTTGCTATCTCGGCTCACACACAAAGAGATTACAATGAACGACGAGGAAATGAGAATGGCAGAGAGAGAGGACAAGGTCGCTTTGGAGGAAGGCCTGGTGGAATGCAGATGGGTGGAC 184  
5-2401    ----TAGCATCGGAGAGACCTATTACTAACATG---GTGAAAGTGACACTGATCGTTGCCATTGTGGCTGCTCTTGCTATCTCAGCTCACGCACAAAGAGATTACAATGAACTACGAGGAAATAAGAATGGCAGAGAGAGAGGACAAGGTCGCTTTGGAGGAAGGCCGGGTGGAATGCAGATGGGTGGAT 183  
5-2402    ----TAGCATCGGAGAGACCTATTACTAACATG---GTGAAAGTGACACTGATCGTTGCCATTGTGGCTGCTCTTGCTATCTCAGCTCACGCACAAAGAGATTACAATGAACTACGAGGAAATAAGAATGGCAGAGAGAGAGGACAAGGTCGCTTTGGAGGAAGGCCGGGTGGAATGCAGATGGGTGGAT 183  
5-2404    ---TAGACATCGGAGAGACCTATTACTAACATG---GTGAAAGTGACACTGATCGTTGCCATTGTGGCTGCTCTTGCTATCTCAGCTCACGCACAAAGAGATTACAATGAACTACGAGGAAATAAGAATGGCAGAGAGAGAGGACAAGGTCGCTTTGGAGGAAGGCCGGGTGGAATGCAGATGGGTGGAT 184  
5-2407    ---TAGACTCTTGAGAGACCTATTACTAACATG---GTGAAAGTGACACTGATCGTTGCCATTGTGGCTGCTCTTGCTATCTCAGCTCACGCACAAAGGGATTACAATGAACTACGAGGAAATAAGAATGGCAGAGAGAGAGGACAAGGTCGCTTTGGAGGAAGGCCGGGTGGAATGCAGATGGGTGGAT 184  
5-2409    ----TAGCATCGGAGAGACCTATTACTAACATG---GTGAAAGTGACACTGATCGTTGCCATTGTGGCTGCTCTTGCTATCTCAGCTCACGCACAAAGAGATTACAATGAACTACGAGGAAATAAGAATGGCAGAGAGAGAGGACAAGGTCGCTTTGGAGGAAGGCCGGGTGGAATGCAAATGGGTGGAT 183  
5-2410    -TAGAACTACGTGAGAGACCTTCACTAAACATGGAGGTGAAAGTGACACTGATCGTTGCCATTGTGGCTGCTCTTGCTATCTCGGCTCACGCACAAAGAGATTTCAATGACCGACGAGGAATGGAGAATGACACAGAGAGAGGACAAGGTGGCTTTGGAGGAAGGCCTGGTGGAATGCAGATGGGTGGTC 189  
5-2411    ------TAGCATCGGAGAGACCTTACTAACATGGAGGTGAAAGTGACACTGATCGTTGCCATTGTGGCTGCTCTTGCTATCTCGGCTCACGCACAAAGAGATTTCAATGAACGACGAGGAAAGGAGAATGACACAGAGAGAGGACAAGGTGGCTTTGGAGGAAGGCCTGGTGGAATGCAGATGGGTGGTC 184  
5-2413    ------TAGACTCGGAGAGACCTTACTAACATGGAGGTGAAAGTGACACTGATCGTTGCCATTGTGGCTGCTCTTGCTATCTCGGCTCACGCACAAAGAGATTTCAATGAACGACGAGGAAAGGAGAATGACACAGAGAGAGGACAAGGTGGCTTTGGAGGAAGGCCTGGTGGAATGCAGATGGGTGGTC 184  
5-2414    ------TAGACTCGGAGAGACCTTACTAACATGGAGGTGAAAGTGACACTGATCGTTGCCATTGTGGCTGCTCTTGCTATCTCGGCTCACGCACAAAGAGATTTCAATGAACGACGAGGAAAGGAGAATGACACAGAGAGAGGACAAGGTGGCTTTGGAGGAAGGCCTGGTGGAATGCAGATGGGTGGTC 184  
5-2415    ------TAGCATCGGAGAGACCTTACTAACATGGAGGTGAAAGTGACACTGATCGTTGCCATTGTGGCTGCTCTTGCTATCTCGGCTCACGCACAAAGAGATTTCAATGAACGACGAGGAAAGGAGAATGACACAGAGAGAGGACAAGGTGGCTTTGGAGGAAGGCCTGGTGGAATGCAGATGGGTGGTC 184  
5-2403    ----------------TAGACCTTACAAACATGGAGGTGAAAGCAACATTGATCGTTGCCATTGTGGCTGCTCTTGCTATCTCGGCTCACGCACGAAGAGATTTCAATGAACGGCGAGGAAATGAGAATGGCAGAGAGAGAGGACAAGGTCGCTTTGGAGGAAGGCCTGGTGGAATGCAGATGGGTGGAT 174  
5-2406    ----TAGCATCGGAGAGACCTATTACTATCATG---GTGAAAGTGACACTGATCGTTGCCATTGTGGCTGCTCTTGCTATCTCGGCTCACGCACGAAGAGATTTCAATGAACGACGAGGAAAGGAGAATGGCAGAGAGAGAGGACAAGGTGGCTTTGGAGGAAGGCCTGGTGGAATGCAGACGGGTAGTC 183  
5-2412    ------TAGACTCGGAGAGACCTTACTAACATGGAGGTGAAAGTGACACTGATCGTTGCCATTGTGGCTGCTCTTGCTATCTCGGCTCACGCAGAAAGAGATTTCAATGAACGACGAGGAAAGGAGAATGGCAGAGAGAGAGGACAAGGTGGCTTTGGAGGAAGGCCTGGTGGAATGCAGACGGGTAGTC 184  
5-2408    ----TAGCATCGGAGAGACCTATTACTAACATG---GTGAAAGTGACACTGATCGTTGCCATTGTGGCTGCTCTTGCTATCTCAGCTCACGCACAAAGAGATTACAATGAACTACGAGGAAATAAGAATGGCAGAGAGAGGACAAGGTCGCTTTGGAGGAAGGCCGGGTGGAATGCAGATGGGTGGATCG 183  


                  200       210       220       230       240       250       260       270       280       290       300       310       320       330       340       350       360       370       380                  
          ....|....|....|....|....|....|....|....|....|....|....|....|....|....|....|....|....|....|....|....|....|....|....|....|....|....|....|....|....|....|....|....|....|....|....|....|....|....|
1-1515    CTAGGCAAGATGGTGGACCAATGGGTGGAAGGAGGTTCGATGGACCTGAATCTGGTGCCCCACAAATGGAAGGACGCAGACAAAATGGCGGTCCGATGGGTGGTAGG----------------------------------------------------------------------------------- 293  
1-1523    CTAGGCAAGATGGTGGACCAATGGGTGGAAGGAGGTTCGATGGACCTGAATCTGGTGCCCCACAAATGGAAGGACGCAGACAAAATGGCGGTCCGATGGGTGGTAGG----------------------------------------------------------------------------------- 293  
1-1504    CTAGGCAAGATGGTGGACCAATGGGTGGAAGGAGGTTCGATGGACCTGAATCTGGTGCCCCACAAATGGAAGGACGCAGACAAAATGGCGGTCCGATGGGTGGTAGG----------------------------------------------------------------------------------- 293  
1-1533    CTAGGCAAGATGGTGGACCAATGGGTGGAAGGAGGTTCGATGGACCTGAATCTGGTGCCCCACAAATGGAAGGACGCAGACAAAATGGCGGTCCGATGGGTGGTAGG----------------------------------------------------------------------------------- 268  
1-1547    CTAGGCAAGATGGTGGACCAATGGGTGGAGGGAGGTTCGATGGACCTGAATCTGGTGCCCCACAAATGGAAGGACGCAGACAAAATGGCGGTCCGATGGGTGGTAGG----------------------------------------------------------------------------------- 293  
1-1549    CTAGGCAAGATGGTGGACCAATGGGTGGAGGGAGGTTCGATGGACCTGAATCTGGTGCCCCACAAATGGAAGGACGCAGACAAAATGGCGGTCCGATGGGTGGTAGG----------------------------------------------------------------------------------- 295  
1-1505    CTAGGCAAGATGGTGGACCAATGGGTGGAGGGAGGTTCGATGGACCTGAATCTGGTGCCCCACAAATGGAAGGACGCAGACAAAATGGCGGTCCGATGGGTGGTAGG----------------------------------------------------------------------------------- 293  
1-1512    CTAGGCAAGATGGTGGACCAATGGGTGGAGGGAGGTTCGATGGACCTGAATCTGGTGCCCCACAAATGGAAGGACGCAGACAAAATGGCGGTCCGATGGGTGGTAGG----------------------------------------------------------------------------------- 293  
1-1514    CTAGGCAAGATGGTGGACCAATGGGTGGAGGGAGGTTCGATGGACCTGAATCTGGTGCCCCACAAATGGAAGGACGCAGACAAAATGGCGGTCCGATGGGTGGTAGG----------------------------------------------------------------------------------- 293  
1-1528    CTAGGCAAGATGGTGGACCAATGGGTGGAGGGAGGTTCGATGGACCTGAATCTGGTGCCCCACAAATGGAAGGACGCAGACAAAATGGCGGTCCGATGGGTGGTAGG----------------------------------------------------------------------------------- 293  
1-1532    CTAGGCAAGATGGTGGACCAATGGGTGGAGGGAGGTTCGATGGACCTGAATCTGGTGCCCCACAAATGGAAGGACGCAGACAAAATGGCGGTCCGATGGGTGGTAGG----------------------------------------------------------------------------------- 293  
1-1539    CTAGGCAAGATGGTGGACCAATGGGTGGAGGGAGGTTCGATGGACCTGAATCTGGTGCCCCACAAATGGAAGGACGCAGACAAAATGGCGGTCCGATGGGTGGTAGG----------------------------------------------------------------------------------- 293  
1-1536    CTAGGCAAGATGGTGGACCAATGGGTGGAGGGAGGTTCGATGGACCTGAATCTGGTGCCCCACAAATGGAAGGACGCAGACAAAATGGCGGTCCGATGGGTGGTAGG----------------------------------------------------------------------------------- 292  
1-1535    CTAGGCAAGATGGTGGACCAATGGGTGGAGGGAGGTTCGACGGACCTGAATCTGGTGCCCCACAAATGGAAGGACGCAGACAAAATGGCGGTCCGATGGGTGGTAGG----------------------------------------------------------------------------------- 293  
1-1534    CTAGGCAAGATGGTGGACCAATGGGTGGAGGGAGGTTCGATGGACCTGAATCTGGTGCCCCACAAATGGAAGGACGCAGACAAAATGGCGGTCCGATGGGTGGTAGG----------------------------------------------------------------------------------- 293  
1-2402    CTAGGCAAGATGGTGGACCAATGGGTGGAAGGAGGTTCGATGGACCTGAATCTGGTGCCCCACAAATGGAAGGACGCAGACAAAATGGCGGTCCGATGGGTGGTAGG----------------------------------------------------------------------------------- 293  
1-2404    CAAGGCAAGATGGTGGACCAATGGGTGGAAGGAGATTCGATGGACCTGAATCTGGTGCCCCACAAATGGAAGGACGCAGACAAAATGGCGGTCCGATGGGTGGTAGG----------------------------------------------------------------------------------- 291  
1-2405    CTAGGCAAGATGGTGGACCAATGGGTGGAAGGAGGTTCGATGGACCTGAATCTGGTGCCCCACAAATGGAAGGACGCAGACAAAATGGCGGTCCGATGGGTGGTAGG----------------------------------------------------------------------------------- 293  
1-2406    CTAGGCAAGATGGTGGACCAATGGGTGGAAGGAGGTTCGATGGACCTGAATCTGGTGCCCCACAAATGGAAGGACGCAGACAAAATGGCGGTCCGATGGGTGGTAGG----------------------------------------------------------------------------------- 293  
1-2407    CAAGGCAAGATGGTGGACCAATGGGTGGAAGGAGATTCGATGGACCTGAATCTGGTGCCCCACAAATGGAAGGACGCAGACAAAATGGCGGTCCGATGGGTGGTAGG----------------------------------------------------------------------------------- 291  
1-2412    CTAGGCAAGATGGTGGACCAATGGGTGGAAGGAGGTTCGATGGACCTGAATCTGGTGCCCCACAAATGGAAGGACGCAGACAAAATGGCGGTCCGATGGGTGGTAGG----------------------------------------------------------------------------------- 293  
1-2413    CTAGGCAAGATGGTGGACCAATGGGTGGAAGGAGGTTCGATGGACCTGAATCTGGTGCCCCACAAATGGAAGGACGCAGACAAAATGGCGGTCCGATGGGTGGTAGG----------------------------------------------------------------------------------- 293  
1-2416    CAAGGCAAGATGGTGGACCAATGGGTGGAAGGAGATTCGATGGACCTGAATCTGGTGCCCCACAAATGGAAGGACGCAGACAAAATGGCGGTCCGATGGGTGGTAGG----------------------------------------------------------------------------------- 291  
1-2417    CAAGGCAAGATGGTGGACCAATGGGTGGAAGGAGATTCGATGGACCTGAATCTGGTGCCCCACAAATGGAAGGACGCAGACAAAATGGCGGTCCGATGGGTGGTAGG----------------------------------------------------------------------------------- 291  
1-2418    CTAGGCAAGATGGTGGACCAATGGGTGGAAGGAGGTTCGATGGACCTGAATCTGGTGCCCCACAAATGGAAGGACGCAGACAAAATGGCGGTCCGATGGGTGGTAGG----------------------------------------------------------------------------------- 293  
1-2420    CAAGGCAAGATGGTGGACCAATGGGTGGAAGGAGATTCGATGGACCTGAATCTGGTGCCCCACAAATGGAAGGACGCAGACAAAATGGCGGTCCGATGGGTGGTAGG----------------------------------------------------------------------------------- 291  
1-2421    CAAGGCAAGATGGTGGACCAATGGGTGGAAGGAGATTCGATGGACCTGAATCTGGTGCCCCACAAATGGAAGGACGCAGACAAAATGGCGGTCCGATGGGTGGTAGG----------------------------------------------------------------------------------- 291  
1-2422    CTAGGCAAGATGGTGGACCAATGGGTGGAAGGAGGTTCGATGGACCTGAATCTGGTGCCCCACAAATGGAAGGACGCAGACAAAATGGCGGTCCGATGGGTGGTAGG----------------------------------------------------------------------------------- 293  
1-2425    CTAGGCAAGATGGTGGACCAATGGGTGGAAGGAGGTTCGATGGACCTGAATCTGGTGCCCCACAAATGGAAGGACGCAGACAAAATGGCGGTCCGATGGGTGGTAGG----------------------------------------------------------------------------------- 293  
1-2426    CTAGGCAAGATGGTGGACCAATGGGTGGAAGGAGGTTCGATGGACCTGAATCTGGTGCCCCACAAATGGAAGGACGCAGACAAAATGGCGGTCCGATGGGTGGTAGG----------------------------------------------------------------------------------- 293  
1-2427    CTAGGCAAGATGGTGGACCAATGGGTGGAAGGAGGTTCGATGGACCTGAATCTGGTGCCCCACAAATGGAAGGACGCAGACAAAATGGCGGTCCGATGGGTGGTAGG----------------------------------------------------------------------------------- 293  
1-2428    CTAGGCAAGATGGTGGACCAATGGGTGGAAGGAGGTTCGATGGACCTGAATCTGGTGCCCCACAAATGGAAGGACGCAGACAAAATGGCGGTCCGATGGGTGGTAGG----------------------------------------------------------------------------------- 293  
1-2430    CTAGGCAAGATGGTGGACCAATGGGTGGAAGGAGGTTCGATGGACCTGAATCTGGTGCCCCACAAATGGAAGGACGCAGACAAAATGGCGGTCCGATGGGTGGTAGG----------------------------------------------------------------------------------- 293  
1-2431    CAAGGCAAGATGGTGGACCAATGGGTGGAAGGAGATTCGATGGACCTGAATCTGGTGCCCCACAAATGGAAGGACGCAGACAAAATGGCGGTCCGATGGGTGGTAGG----------------------------------------------------------------------------------- 291  
1-2432    CTAGGCAAGATGGTGGACCAATGGGTGGAAGGAGGTTCGATGGACCTGAATCTGGTGCCCCACAAATGGAAGGACGCAGACAAAATGGCGGTCCGATGGGTGGTAGG----------------------------------------------------------------------------------- 293  
1-2433    CTAGGCAAGATGGTGGACCAATGGGTGGAAGGAGGTTCGATGGACCTGAATCTGGTGCCCCACAAATGGAAGGACGCAGACAAAATGGCGGTCCGATGGGTGGTAGG----------------------------------------------------------------------------------- 293  
1-2434    CAAGGCAAGATGGTGGACCAATGGGTGGAAGGAGATTCGATGGACCTGAATCTGGTGCCCCACAAATGGAAGGACGCAGACAAAATGGCGGTCCGATGGGTGGTAGG----------------------------------------------------------------------------------- 291  
1-2435    CAAGGCAAGATGGTGGACCAATGGGTGGAAGGAGATTCGATGGACCTGAATCTGGTGCCCCACAAATGGAAGGACGCAGACAAAATGGCGGTCCGATGGGTGGTAGG----------------------------------------------------------------------------------- 291  
1-2436    CTAGGCAAGATGGTGGACCAATGGGTGGAAGGAGGTTCGATGGACCTGAATCTGGTGCCCCACAAATGGAAGGACGCAGACAAAATGGCGGTCCGATGGGTGGTAGG----------------------------------------------------------------------------------- 291  
1-2437    CTAGGCAAGATGGTGGACCAATGGGTGGAAGGAGGTTCGATGGACCTGAATCTGGTGCCCCACAAATGGAAGGACGCAGACAAAATGGCGGTCCGATGGGTGGTAGG----------------------------------------------------------------------------------- 293  
1-2439    CAAGGCAAGATGGTGGACCAATGGGTGGAAGGAGATTCGATGGACCTGAATCTGGTGCCCCACAAATGGAAGGACGCAGACAAAATGGCGGTCCGATGGGTGGTAGG----------------------------------------------------------------------------------- 291  
1-2440    CAAGGCAAGATGGTGGACCAATGGGTGGAAGGAGATTCGATGGACCTGAATCTGGTGCCCCACAAATGGAAGGACGCAGACAAAATGGCGGTCCGATGGGTGGTAGG----------------------------------------------------------------------------------- 291  
1-2441    CTAGGCAAGATGGTGGACCAATGGGTGGAAGGAGGTTCGATGGACCTGAATCTGGTGCCCCACAAATGGAAGGACGCAGACAAAATGGCGGTCCGATGGGTGGTAGG----------------------------------------------------------------------------------- 293  
1-2442    CAAGGCAAGATGGTGGACCAATGGGTGGAAGGAGATTCGATGGACCTGAATCTGGTGCCCCACAAATGGAAGGACGCAGACAAAATGGCGGTCCGATGGGTGGTAGG----------------------------------------------------------------------------------- 291  
1-2414    CGAGGCAAGATGGTGGACCAATGGGTGGAAGAAGGTTCGATGGACCTGACTCTGGTGCCCCACAAATGGATGGACGGAGACAAGATGGTGGACCAATGGGTGGAAGG---------------------------------------------------------------------------AGGTTCGA 296  
1-2424    GGTCCGATGGGTGGTAG----------------------------------------------------------------------------------------------------------------------------------------------------------------------------- 202  
1-2429    GGTCCGATGGGTGGTAG----------------------------------------------------------------------------------------------------------------------------------------------------------------------------- 201  
2-1501    CAAGGACAGATGGCGGTCCGATGGGTGGTAGG---------------------------------------------------------------------------AGATTCGACGGACATGGATTTGGTGCCCCGCCGATGGGTGGACCAAGGCAAGATGGTGGACCAATGGGTGGAAGGAGGTTCGA 299  
2-1502    CAAGGCAAGATGGCGGTCCGATGGGTGGTAGG---------------------------------------------------------------------------AGATTCGACGGACATGGATTTGGTGCCCCGCCGATGGGTGGACCAAGGCAAGATGGTGGACTAATGGGTGGAAGGAGGCTCGA 299  
2-1505    CAAGGCAAGATGGCGGTCCGATGGGTGGTAGG---------------------------------------------------------------------------AGATTCGACGGACATGGATTTGGTGCCCCGCCGATGGGTGGACCAAGGCAAGATGGTGGACCAATGGGTGGAAGGAGGTTCGA 299  
2-1506    CAAGGCAAGATGGCGGTCCGATGGGTGGTAGG---------------------------------------------------------------------------AGATTCGACGGACATGGATTTGGTGCCCCGCCGATGGGTGGACCAAGGCAAGATGGTGGACCAATGGGTGGAAGGAGGTTCGA 299  
2-1507    CAAGGCAAGATGGCGGTCCGATGGGTGGTAGG---------------------------------------------------------------------------AGATTCGACGGACATGGGTTTGGTGCCCCGCCGATGGGTGGACCAAGGCAAGATGGTGGACCAATGGGTGGAAGGAGGTTCGA 300  
2-1508    CAAGGCAAGATGGCGGTCCGATGGGTGGTAGG---------------------------------------------------------------------------AGATTCGACGGACATGGATTTGGTGCCCCGCCGATGGGTGGACCAAGGCAAGATGGTGGACCAATGGGTGGAAGGAGGTTCGA 299  
2-1509    CAAGGCAAGATGGCGGTCCGATGGGTGGTAGG---------------------------------------------------------------------------AGATTCGACGGACATGGATTTGGTGCCCCGCCGATGGGTGGACCAAGGCAAGATGGTGGACCAATGGGTGGAAGGAGGTTCGA 299  
2-1510    CAAGGCAAGATGGCGGTCCGATGGGTGGTAGG---------------------------------------------------------------------------AGATTCGACGGACATGGATTTGGTGCCCCGCCGATGGGTGGACCAAGGCAAGATGGTGGACCAATGGGTGGAAGGAGGTTCGA 291  
2-1514    CAAGGCAAGATGGCGGTCCGATGGGTGGTAGG---------------------------------------------------------------------------AGATTCGACGGACATGGATTTGGTGCCCCGCCGATGGGTGGACCAAGGCAAGATGGTGGACCAATGGGTGGAAGGAGGTTCGA 299  
2-1511    CAAGGCAAGATGGCGGTCCGATGGGTGGTAGG---------------------------------------------------------------------------AGATTCGACGGACATGGATTTGGTGCCCCGCCGATGGGTGGACCAAGGCAAGATGGTGGACCAATGGGTGGAAGGAGGTTCGA 299  
2-2401    CTAGGCAAGATGGTGGACCAATGGGTGGAAGGAGATTCGATGGACCTGAATCTGGTGCCCCACAAATGGAAGGACGCAGACAAAATGGCGGTCCGATGGGTGGTAGG----------------------------------------------------------------------------------- 290  
2-2404    CTAGGCAAGATGGTGGACCAATGGGTGGAAGGAGATTCGATGGACCTGAATCTGGTGCCCCACAAATGGAAGGACGCAGACAAAATGGCGGTCCGATGGGTGGTAGG----------------------------------------------------------------------------------- 290  
2-2406    CTAGGCAAGATGGTGGACCAATGGGTGGAATGAGGTTCGATGGACCTGAATCTGGTGCCCCACAAATGGATGGACGCAGACAAAATGGCGGTCCGATGGGTGGTAGG----------------------------------------------------------------------------------- 290  
2-2407    CTAGGCAAGATGGTGGACCAATGGGTGGAAGGAGATTCGATGGACCCGAATCTGGTGCCCCACAAATGGAAGGACGCAGACAAAATGGCGGTCCGATGGGTGGTAGG----------------------------------------------------------------------------------- 290  
2-2408    CTAGGCAAGATGGTGGACCAATGGGTGGAAGGAGATTCGATGGACCTGAATCTGGTGCCCCACAAATGGAAGGACGCAGACAAAATGGCGGTCCGATGGGTGGTAGG----------------------------------------------------------------------------------- 290  
2-2409    CTAGGCAAGATGGTGGACCAATGGGTGGAAGGAGATTCGATGGACCTGAATCTGGTGCCCCACAAATGGAAGGACGCAGACAAAATGGCGGTCCGATGGGTGGTAGG----------------------------------------------------------------------------------- 290  
2-2411    CTAGGCAAGATGGTGGACCAATGGGTGGAAGGAGATTCGATGGACCTGAATCTGGTGCCCCACAAATGGAAGGACGCAGACAAAATGGCGGTCCGATGGGTGGTAGG----------------------------------------------------------------------------------- 291  
2-2405    CTAGGCAAGATGGTGGACCAGTGGTTGGAAGG------------------------------------------------------------------------------------------------------------------------------------------------------AGGTTCGA 224  
2-2413    CTAGGCAAGATGGTGGACCAGTGGTTGGAAGG------------------------------------------------------------------------------------------------------------------------------------------------------AGGTTCGA 224  
2-2403    CTAGGCAAGATGGTGGACCAATGGGTGGAATGAGGTTCGATGGACCTGAATCTGGTGCCCCACAAATGGATGGACGCAGACAAAATGGCGGTCCGATGGGTGGTAGG----------------------------------------------------------------------------------- 293  
2-2415    CGAGGCAAGATGGTGGACCAATGGGTGGAAGAAGGTTCGATGGACCTGACTCTGGTGCCCCACAAATGGATGGACGGAGACAAGATGGTGGACCAATGGGTGGAAGG---------------------------------------------------------------------------AGGTTCGA 298  
2-2414    CGAGGCAAGATGGTGGACCAATGGGTGGAAGAAGGTTCGATGGACCTGACTCTGGTGCCCCACAAATGGATGGACGGAGACAAGATGGTGGACCAATGGGTGGAAGG---------------------------------------------------------------------------AGGTTCGA 297  
3-15-100  CAAGGCAAGATGGCGGTCCGATGGGTGGTAGG---------------------------------------------------------------------------AGATTCGACGGACATGGATTTGGTGCCCCGCCGATGGGTGGACCAAGGCAAGATGGTGGACCAATGGGTGGAAGGAGGTTCGA 300  
3-15-400  CAAGGCAAGATGGCGGTCCGATGGGTGGTAGG---------------------------------------------------------------------------AGATTCGACGGACATGGATTTGGTGCCCCGCCGATGGGTGGACCAAGGCAAGATGGTGGACCAATGGGTGGAAGGAGGTTCGA 299  
3-15-400  CAAGGCAAGATGGCGGTCCGATGGGTGGTAGG---------------------------------------------------------------------------AGATTCGACGGACATGGATTTGGTGCCCCGCCGATGGGTGGACCAAGGCAAGATGGTGGACCAATGGGTGGAAGGAGGTTCGA 299  
3-15-400  CAAGGCAAGATGGCGGTCCGATGGGCGGTAGG---------------------------------------------------------------------------AGATTCGACGGACATGGATTTGGTGCCCCGCCGATGGGTGGACCAAGGCAAGATGGTGGACCAATGGGTGGAAGGAGGTTCGA 300  
3-15-400  CAAGGCAAGATGGCGGTCCGATGGGTGGTAGG---------------------------------------------------------------------------AGATTCGACGGACATGGATTTGGTGCCCCGCCGATGGGTGGACCAAGGCAAGATGGTGGACCAATGGGTGGAAGGAGGTTCGA 298  
3-15-401  CAAGGCAAGATGGCGGTCCGATGGGTGGTAGG---------------------------------------------------------------------------AGATTCGACGGACATGGATTTGGTGCCCCGCCGATGGGTGGACCAAGGCAAGATGGTGGACCAATGGGTGGAAGGAGGTTCGA 299  
3-15-401  CAAGGCAAGATGGCGGTCCGATGGGCGGTAGG---------------------------------------------------------------------------AGATTCGACGGACATGGATTTGGTGCCCCGCCGATGGGTGGACCAAGGCAAGATGGTGGACCAATGGGTGGAAGGAGGTTCGA 300  
3-15-401  CAAGGCAAGATGGCGGTCCGATGGGTGGTAGG---------------------------------------------------------------------------AGATTCGACGGACATGGATTTGGTGCCCCGCCGATGGGTGGACCAAGGCAAGATGGTGGACCAATGGGTGGAAGGAGGTTCGA 299  
3-15-401  CAAGGCAAGATGGCGGTCCGATGGGTGGTAGG---------------------------------------------------------------------------AGATTCGACGGACATGGATTTGGTGCCCCGCCGATGGGTGGACCAAGGCAAGATGGTGGACCAATGGGTGGAAGGAGGTTCGA 299  
3-15-401  CAAGGCAAGATGGCGGTCCGATGGGTGGTAGG---------------------------------------------------------------------------AGATTCGACGGACATGGATTTGGTGCCCCGCCGATGGGTGGACCAAGGCAAGATGGTGGACCAATGGGTGGAAGGAGGTTCGA 299  
3-15-402  CAAGGCAAGATGGCGGTCCGATGGGTGGTAGG---------------------------------------------------------------------------AGATTCGACGGACATGGATTTGGTGCCCCGCCGATGGGTGGACCAAGGCAAGATGGTGGACCAATGGGTGGAAGGAGGTTCGA 300  
3-15-402  CAAGGCAAGATGGCGGTCCGATGGGTGGTAGG---------------------------------------------------------------------------AGATTCGACGGACATGGATTTGGTGCCCCGCCGATGGGTGGACCAAGGCAAGATGGTGGACCAATGGGTGGAAGGAGGTTCGA 299  
3-15-401  CAAGGCAAGATGGCGGTCCGATGGGTGGTAGG---------------------------------------------------------------------------AGATTCGACGGACATGGATTTGGTGCCCCGCCGATGGGTGGACCAAGGCAAGATGGTGGACCAATGGGTGGAAGGAGGTTCGA 300  
3-15-100  CAAGGCAAGATGGCGGTCCGATGGGTGGTAGG---------------------------------------------------------------------------AGATTCGACGGGCATGGATTTGGTGCCCCGCCGATGGGTGGACCAAGGCAAGATGGTGGACCAATGGGTGGAAGGAGGTTCGA 299  
3-15-402  CTAGGCAAGATGGTGGACCAATGGGTGGAATGAGGTTCGATGGACCTGAATCTGGTGCCCCACAAATGGATGGACGCAGACAAATGGCGGTCCGATGGGTGGTAGGAGATTCGACGGACCTCGATTTGGTGGCTCCAGACCAGATGGTGCTGGAGGAAGACCTTTCTTCGGCCAAGGAGGCAGGCGTGGT 373  
3-15-100  CTAGGCAAGATGGTGGACCAATGGGTGGAATGAGGTTCGATGGACCTGAATCTGGTGCCCCACAAATGGATGGACGCAGACAAATGGCGGTCCGATGGGTGGTAGGAGATTCGACGGACCTCGATTTGGTGGCTCCAGACCAGATGGTGCTGGAGGAAGACCTTTCTTCGGCCAAGGAGGCAGGCGTGGT 373  
3-15-100  CTAGGCAAGATGGTGGACCAATGGGTGGAATGAGGTTCGATGGACCTGAATCTGGTGCCCCACAAATGGATGGACGCAGACAAATGGCGGTCCGATGGGTGGTGGGAGATTCGACGGACCTCGATTTGGTGGCTCCAGACCAGATGGTGCTGGAGGAAGACCTTTCTTCGGCCAAGGAGGCAGGCGTGGT 373  
3-15-400  AGGCAAGATGGCGGTCCGATGGGTGGTAG-----------------------------------------------------------------------------GAGACTCGACGGACATGGATTTGGTGCCCCGCCGATGGGTGGACCAAGGCAAGATGGTGGACCAATGGGTGGAAGGAGGTTCGA 297  
3-24-400  CTAGGCAAGATGGTGGACCAATGGGTGGAAGGAGATTCGATGGACCTGAATCTGGTGCCCCACAAATGGAAGGACGCAGACAAAATGGCGGTCCGATGGGTGGTAGG----------------------------------------------------------------------------------- 291  
3-24-400  CTAGGCAAGATGGTGGACCAATGGGTGGAAGGAGGTTCGATGGACCTGAATCTGGTGCCCCACAAATGGAAGGACGCAGACAAAATGGCGGTCCGATGGGTGGTAGG----------------------------------------------------------------------------------- 291  
3-24-100  CTAGGCAAGATGGTGGACCAATGGGTGGAAGGAGATTCGATGGACCTGAATCTGGTGCCCCACAAATGGAAGGACGCAGACAAAATGGCGGTCCGATGGGTGGTAGG----------------------------------------------------------------------------------- 291  
3-24-400  CTAGGCAAGATGGTGGACCAATGGGTGGAAGGAGGTTCGATGGACCTGAATCTGGTGCCCCACAAATGGAAGGACGCAGACAAAATGGCGGTCCGATGGGTGGTAGG----------------------------------------------------------------------------------- 291  
3-24-401  CTAGGCAAGATGGTGGACCAATGGGTGGAAGGAGATTCGATGGACCTGAATCTGGTGCCCCACAAATGGAAGGACGCAGACAAAATGGCGGTCCGATGGGTGGTAGG----------------------------------------------------------------------------------- 291  
3-24-402  CTAGGCAAGATGGTGGACCAATGGGTGGAAGGAGGTTCGATGGACCTGAATCTGGTGCCCCACAAATGGAAGGACGCAGACAAAATGGCGGTCCGATGGGTGGTAGG----------------------------------------------------------------------------------- 291  
3-24-402  CTAGGCAAGATGGTGGACCAATGGGTGGAAGGAGGTTCGATGGACCTGAATCTGGTGCCCCACAAATGGAAGGACGCAGACAAAATGGCGGTCCGATGGGTGGTAGG----------------------------------------------------------------------------------- 290  
3-24-402  CTAGGCAAGATGGTGGACCAATGGGTGGAAGGAGATTCGATGGACCTGAATCTGGTGCCCCACAAATGGAAGGACGCAGACAAAATGGCGGTCCGATGGGTGGTAGG----------------------------------------------------------------------------------- 291  
3-24-400  CTAGGCAAGATGGTGGACCAATGGGTGGAAGGAGGTTCGATGGACCTGAATCTGGTGCCCCACAAATGGATGGACGCAGACAAAATGGCGGTCCGATGGGTGGTAGG----------------------------------------------------------------------------------- 290  
3-24-401  CTAGGCAAGATGGTGGACCAATGGGTGGAAGGAGGTTCGATGGACCTGAATCTGGTGCCCCACAAATGGATGGACGCAGACAAAATGGCGGTCCGATGGGTGGTAGG----------------------------------------------------------------------------------- 290  
3-24-100  CTAGGCAAGATGGTGGACCAATGGGTGGAATGAGGTTCGATGGACCTGAATCTGGTGCCCCACAAATGGATGGACGCAGACAAAATGGCGGTCCGATGGGTGGTAGG----------------------------------------------------------------------------------- 290  
3-24-401  TGAGGCAAGATGGTGGACCAATGGGTGGAAGAAGGTTCGATGGACCTGACTCTGGTGCCCCACAAATGGATGGACGGAGACAAGATGGTGGACCAATGGGTGGAAGG---------------------------------------------------------------------------AGGTTCGA 298  
3-24-401  TGAGGCAAGATGGTGGACCAATGGGCGGAAGAAGGTTCGATGGACCTGACTCTGGTGCCCCACAAATGGATGGACGGAGACAAGATGGTGGACCAATGGGTGGAAGG---------------------------------------------------------------------------AGGTTCGA 298  
3-24-401  CAAGGCAAGATGGCGGTCCGATGGGTGGTAGG---------------------------------------------------------------------------AGATTCGACGGACATGGATTTGGTGCCCCGCCGATGGGTGGACCAAGGCAAGATGGTGGACCAATGGGTGGAAGGAGGTTCGA 299  
3-24-400  CAAGGCAAGATGGCGGTCCGATGGGTGGTAGG---------------------------------------------------------------------------AGATTCGACGGACATGGATTTGGTGCCCCGCCGATGGGTGGACCAAGGCAAGATGGTGGACCAATGGGTGGAAGGAGGTTCGA 300  
3-24-401  CAAGGCAAGATGGCGGTCCGATGGGTGGTAGG---------------------------------------------------------------------------AGATTCGACGGACATGGATTTGGTGCCCCGCCGATGGGTGGACCAAGGCAAGATGGTGGACCAATGGGTGGAAGGAGGTTCGA 300  
3-24-402  CAAGGCAAGATGGCGGTCCGATGGGTGGTAGG---------------------------------------------------------------------------AGATTCGACGGACATGGATTTGGTGCCCCGCCGATGGGTGGACCAAGGCAAGATGGTGGACCAATGGGTGGAAGGAGGTTCGA 299  
6-2415    CTAGGCAAGATGGTGGACCAATGGATGGAATGAGGTTCGATGGACCTGAATCTGGTGCCCCACAAATGGAAGGACGCAGACAAAATGGCGGTCCGATGGGTGGTAGG----------------------------------------------------------------------------------- 290  
6-2426    CTAGGCAAGATGGTGGACCAATGGATGGAATGAGGTTCGATGGACCTGAATCTGGTGCCCCACAAATGGAAGGACGCAGACAAAATGGCGGTCCGATGGGTGGTAGG----------------------------------------------------------------------------------- 290  
6-2446    CTAGGCAAGATGGTGGACCAATGGATGGAATGAGGTTCGATGGACCTGAATCTGGTGCCCCACAAATGGAAGGACGCAGACAAAATGGCGGTCCGATGGGTGGTAGG----------------------------------------------------------------------------------- 290  
6-2401    CTAGGCAAGATGGTGGACAAATGGGTGGAAGGAGGTTCGATGGACCTGAATCTGGTGCCCCACAAATGGAAGGACGCAGACAAAATGGCGGTCCGATGGGTGGTAGG----------------------------------------------------------------------------------- 291  
6-2402    CTAGGCAAGATGGTGGACCAATGGGTGGAAGGAGGTTCGATGGACCTGAATCTGGTGCCCCACAAATGGAAGGACGCAGACAAAATGGCGGTCCGATGGGTGGTAGG----------------------------------------------------------------------------------- 293  
6-2404    CTAGGCAAGATGGTGGACCAATGGGTGGAAGGAGGTTCGATGGACCTGAATCTGGTGCCCCACAAATGGAAGGACGCAGACAAAATGGCGGTCCGATGGGTGGTAGG----------------------------------------------------------------------------------- 293  
6-2407    CTAGGCAAGATGGTGGACAAATGGGTGGAAGGAGGTTCGATGGACCTGAATCTGGTGCCCCACAAATGGAAGGACGCAGACAAAATGGCGGTCCGATGGGTGGTAGG----------------------------------------------------------------------------------- 291  
6-2408    CTAGGCAAGATGGTGGACCAATGGGTGGAAGGAGGTTCGATGGACCTGAATCTGGTGCCCCACAAATGGAAGGACGCAGACAAAATGGCGGTCCGATGGGTGGTAGG----------------------------------------------------------------------------------- 293  
6-2409    CTAGGCAAGATGGTGGACAAATGGGTGGAAGGAGGTTCGATGGACCTGAATCTGGTGCCCCACAAATGGAAGGACGCAGACAAAATGGCGGTCCGATGGGTGGTAGG----------------------------------------------------------------------------------- 291  
6-2410    CTAGGCAAGATGGTGGACCAATGGGCGGAAGGAGGTTCGATGGACCTGAATCTGGTGCCCCACAAATGGAAGGACGCAGACAAAATGGCGGTCCGATGGGTGGTAGG----------------------------------------------------------------------------------- 293  
6-2411    CTAGGCAAGATGGTGGACAAATGGGTGGAAGGAGGTTCGATGGACCTGAATCTGGTGCCCCACAAATGGAAGGACGCAGACAAAATGGCGGTCCGATGGGTGGTAGG----------------------------------------------------------------------------------- 293  
6-2412    CTAGGCAAGATGGTGGACCAATGGGTGGAAGGAGGTTCGATGGACCTGAATCTGGTGCCCCACAAATGGAAGGACGCAGACAAAATGGCGGTCCGATGGGTGGTAGG----------------------------------------------------------------------------------- 293  
6-2413    CTAGGCAAGATGGTGGACAAATGGGTGGAAGGAGGTTCGATGGACCTGAATCTGGTGCCCCACAAATGGAAGGACGCAGACAAAATGGCGGTCCGATGGGTGGTAGG----------------------------------------------------------------------------------- 291  
6-2414    CTAGGCAAGATGGTGGACCAATGGGTGGAAGGAGGTTCGATGGACCTGAATCTGGTGCCCCACAAATGGAAGGACGCAGACAAAATGGCGGTCCGATGGGTGGTAGG----------------------------------------------------------------------------------- 293  
6-2416    CTAGGCAAGATGGTGGACAAATGGGTGGAAGGAGGTTCGATGGACCTGAATCTGGTGCCCCACAAATGGAAGGACGCAGACAAAATGGCGGTCCGATGGGTGGTAGG----------------------------------------------------------------------------------- 291  
6-2417    CTAGGCAAGATGGTGGACCAATGGGTGGAAGGAGGTTCGATGGACCTGAATCTGGTGCCCCACAAATGGAAGGACGCAGACAAAATGGCGGTCCGATGGGTGGTAGG----------------------------------------------------------------------------------- 293  
6-2420    CTAGGCAAGATGGTGGACAAATGGGTGGAAGGAGGTTCGATGGACCTGAATCTGGTGCCCCACAAATGGAAGGACGCAGACAAAATGGCGGTCCGATGGGTGGTAGG----------------------------------------------------------------------------------- 291  
6-2421    CTAGGCAAGATGGTGGACAAATGGGTGGAAGGAGGTTCGATGGACCTGAATCTGGTGCCCCACAAATGGAAGGACGCAGACAAAATGGCGGTCCGATGGGTGGTAGG----------------------------------------------------------------------------------- 291  
6-2422    CTAGGCAAGATGGTGGACCAATGGGTGGAAGGAGGTTCGATGGACCTGAATCTGGTGCCCCACAAATGGAAGGACGCAGACAAAATGGCGGTCCGATGGGTGGTAGG----------------------------------------------------------------------------------- 293  
6-2424    CTAGGCAAGATGGTGGACAAATGGGTGGAAGGAGGTTCGATGGACCTGAATCTGGTGCCCCACAAATGGAAGGACGCAGACAAAATGGCGGTCCGATGGGTGGTAGG----------------------------------------------------------------------------------- 291  
6-2425    CTAGGCAAGATGGTGGACAAATGGGTGGAAGGAGGTTCGATGGACCTGAATCTGGTGCCCCACAAATGGAAGGACGCAGACAAAATGGCGGTCCGATGGGTGGTAGG----------------------------------------------------------------------------------- 293  
6-2427    CTAGGCAAGATGGTGGACAAATGGGTGGAAGGAGGTTCGATGGACCTGAATCTGGTGCCCCACAAATGGAAGGACGCAGACAAAATGGCGGTCCGATGGGTGGTAGG----------------------------------------------------------------------------------- 291  
6-2428    CTAGGCAAGATGGTGGACCAATGGGTGGAAGGAGGTTCGATGGACCTGAATCTGGTGCCCCACAAATGGAAGGACGCAGACAAAATGGCGGTCCGATGGGTGGTAGG----------------------------------------------------------------------------------- 291  
6-2430    CTAGGCAAGATGGTGGACCAATGGGTGGAAGGAGGTTCGATGGACCTGAATCTGGTGCCCCACAAATGGAGGGACGCAGACAAAATGGCGGTCCGATGGGTGGTAGG----------------------------------------------------------------------------------- 293  
6-2431    CTAGGCAAGATGGTGGACAAATGGGTGGAAGGAGGTTCGATGGACCTGAATCTGGTGCCCCACAAATGGAAGGACGCAGACAAAATGGCGGTCCGATGGGTGGTAGG----------------------------------------------------------------------------------- 291  
6-2432    CTAGGCAAGATGGTGGACAAATGGGTGGAAGGAGGTTCGATGGACCTGAATCTGGTGCCCCACAAATGGAAGGACGCAGACAAAATGGCGGTCCGATGGGTGGTAGG----------------------------------------------------------------------------------- 291  
6-2433    CTAGGCAAGATGGTGGACAAATGGGTGGAAGGAGGTTCGATGGACCTGAATCTGGTGCCCCACAAATGGAAGGACGCAGACAAAATGGCGGTCCGATGGGTGGTAGG----------------------------------------------------------------------------------- 291  
6-2434    CTAGGCAAGATGGTGGACAAATGGGTGGAAGGAGGTTCGATGGACCTGAATCTGGTGCCCCACAAATGGAAGGACGCAGACAAAATGGCGGTCCGATGGGTGGTAGG----------------------------------------------------------------------------------- 291  
6-2439    CTAGGCAAGATGGTGGACAAATGGGTGGAAGGAGGTTCGATGGACCTGAATCTGGTGCCCCACAAATGGAAGGACGCAGACAAAATGGCGGTCCGATGGGTGGTAGG----------------------------------------------------------------------------------- 291  
6-2440    CTAGGCAAGATGGTGGACCAATGGGTGGAAGGAGGTTCGATGGACCTGAATCTGGTGCCCCACAAATGGAAGGACGCAGACAAAATGGCGGTCCGATGGGTGGTAGG----------------------------------------------------------------------------------- 293  
6-2441    CTAGGCAAGATGGTGGACCAATGGGTGGAAGGAGGTTCGATGGACCTGAATCTGGTGCCCCACAAATGGAAGGACGCAGACAAAATGGCGGTCCGATGGGTGGTAGG----------------------------------------------------------------------------------- 293  
6-2435    CTAGGCAAGATGGTGGACAAATGGGTGGAAGGAGGTTCGATGGACCTGAATCTGGTGCCCCACAAATGGAAGGACGCAGACAAAATGGCGGTCCGATGGGTGGTAGG----------------------------------------------------------------------------------- 290  
6-2447    CTAGGCAAGATGGTGGACAAATGGGTGGAAGGAGGTTCGATGGACCTGAATCTGGTGCCCCACAAATGGAAGGACGCAGACAAAATGGCGGTCCGATGGGTGGTAGG----------------------------------------------------------------------------------- 291  
6-2448    CTAGGCAAGATGGTGGACCAATGGGTGGAAGGAGGTTCGATGGACCTGAATCTGGTGCCCCACAAATGGAAGGACGCAGACAAAATGGCGGTCCGATGGGTGGTAGG----------------------------------------------------------------------------------- 293  
6-2436    TTAGGCAAGATGGTGGACCAATGGGTGGAAGGAGGTTCGATGGACCTGAATCTGGTGCCCCACAAATGGAAGGACGCAGACAAAATGGCGGTCCGATGGGTGGTAGG----------------------------------------------------------------------------------- 292  
6-2450    CTAGGCAAGATGGTGGACAAATGGGTGGAAGGAGGTTCGATGGACCTGAATCTGGTGCCCCACAAATGGAAGGACGCAGACAAAATGGCGGTCCGATGGGTGGTAGG----------------------------------------------------------------------------------- 291  
6-2429    CTAGGCAAGATGGTGGACCAATGGGTGGAAGG------------------------------------------------------------------------------------------------------------------------------------------------------AGGTTCGA 224  
6-2438    CTAGGCAAGATGGTGGACCAATGGGTGGAAGG------------------------------------------------------------------------------------------------------------------------------------------------------AGGTTCGA 224  
6-2423    CTAGGCAAGATGGTGGACCAATGGGTGGAAGG------------------------------------------------------------------------------------------------------------------------------------------------------AGGTTCGA 224  
6-2403    CTAGGCAAGATGGTGGACCAATGGGTGGAAGGAGGTTCGATGGACCTGAATCTGGTGCCCCACAAATGGAAGGACGCAGACAAAATGGCGGTCCGATGGGTGGTAGG----------------------------------------------------------------------------------- 293  
6-2444    CTAGGCAAGATGGTGGACCAATGGGTGGAGGGAGGTTCGATGGACCTGAATCTGGTGCCCCACAAATGGAAGGACGCAGACAAAATGGCGGTCCGATGGGTGGTAGG----------------------------------------------------------------------------------- 293  
6-2449    CTAGGCAAGATGGTGGACAAATGGGTGGAAGGAGGTTCGATGGACCTGAATCTGGTGCCCCACAAATGGAAGGACGCAGACAAAATGGCGGTCCGATGGGTGGTAGG----------------------------------------------------------------------------------- 295  
2-1503    CTAGGCAAGATGGTGGACCAATGGGTGGAGGGAGGTTCGATGGACCTGAATCTGGCGCCCCACAAATGGAAGGACGCAGACAAAATGGCGGTCCGATGGGTGGTAGG----------------------------------------------------------------------------------- 296  
2-1509    CTAGGCAAGATGGTGGACCAATGGGTGGAGGGAGGTTCGATGGACCCGAATCTGGTGCCCCACAAATGGAAGGACGCAGACAAAATGGCGGTCCGATGGGTGGTAGG----------------------------------------------------------------------------------- 296  
2-1513    CTAGGCAAGATGGTGGACCAATGGGTGGAGGGAGGTTCGATGGACCTGAATCTGGTGCCCCACAAATGGAAGGACGCAGACAAAATGGCGGTCCGATGGGTGGTAGG----------------------------------------------------------------------------------- 296  
2-1523    CTAGGCAAGATGGTGGACCAATGGGTGGAGGGAGGTTCGATGGACCTGAATCTGGTGCCCCACAAATGGAAGGACGCAGACAAAATGGCGGTCCGATGGGTGGTAGG----------------------------------------------------------------------------------- 296  
2-1524    CTAGGCAAGATGGTGGACCAATGGGTGGAGGGAGGTTCGATGGACCTGAATCTGGTGCCCCACAAATGGAAGGACGCAGACAAAATGGCGGTCCGATGGGTGGTAGG----------------------------------------------------------------------------------- 296  
2-1531    CTAGGCAAGATGGTGGACCAATGGGTGGAGGGAGGTTCGATGGACCTGAATCTGGTGCCCCACAAATGGAAGGACGCAGACAAAATGGCGGTCCGATGGGTGGTAGG----------------------------------------------------------------------------------- 296  
2-1533    CTAGGCAAGATGGTGGACCAATGGGTGGAGGGAGGTTCGATGGACCTGAATCTGGTGCCCCACAAATGGAAGGACGCAGACAAAATGGCGGTCCGATGGGTGGTAGG----------------------------------------------------------------------------------- 296  
2-1536    CTAGGCAAGATGGTGGACCAATGGGTGGAGGGAGGTTCGATGGACCTGAATCTGGTGCCCCACAAATGGAAGGACGCAGACAAAATGGCGGTCCGATGGGTGGTAGG----------------------------------------------------------------------------------- 295  
2-1502    CTAGGCAAGATGGTGGACCAATGGGTGGAAGGAGGTTCGATGGACCTGAATCTGGTGCCCCACAAATGGAAGGACGCAGACAAAATGGCGGTCCGATGGGTGGTAGG----------------------------------------------------------------------------------- 296  
2-1518    CTAGGCAAGATGGTGGACAAATGGGTGGAAGGAGGTTCGATGGACCTGAATCTGGTGCCCCACAAATGGAAGGACGCAGACAAAATGGCGGTCCGATGGGTGGTAGG----------------------------------------------------------------------------------- 294  
2-1519    CTAGGCAAGATGGTGGACAAATGGGTGGAAGGAGGTTCGATGGACCTGAACCTGGTGCCCCACAAATGGAAGGACGCAGACAAAATGGCGGTCCGATGGGTGGTAGG----------------------------------------------------------------------------------- 294  
2-1511    CTAGGCAAGATGGTGGACAAATGGGTGGAAGGAGGTTCGATGGACCTGAATCTGGTGCCCCACAAATGGAAGGACGCAGACAAAATGGCGGTCCGATGGGTGGTAGG----------------------------------------------------------------------------------- 294  
2-1546    CTAGGCAAGATGGTGGACAAATGGGTGGAAGGAGGTTCGATGGACCTGAATCTGGTGCCCCACAAATGGAAGGACGCAGACAAAATGGCGGTCCGATGGGTGGTAGG----------------------------------------------------------------------------------- 294  
2-1548    CTAGGCAAGATGGTGGACAAATGGGTGGAAGGAGGTTCGATGGACCTGAATCTGGTGCCCCACAAATGGAAGGACGCAGACAAAATGGCGGTCCGATGGGTGGTAGG----------------------------------------------------------------------------------- 294  
2-1540    CTAGGCAAGATGGTGGACCAATGGGTGGAGGGAGGTTCGATGGACCCGAATCTGGTGCCCCACAAATGGAAGGACGCAGACAAAATGGCGGTCCGATGGGTGGTAGG----------------------------------------------------------------------------------- 296  
2-2423    CTAGGCAAGATGGTGGACCAATGGGTGGAGGGAGGTTCGATGGACCTGAATCTGGTGCCCCACAAATGGAAGGACGCAGACAAAATGGCGGTCCGATGGGTGGTAGG----------------------------------------------------------------------------------- 293  
2-2436    CTAGGCAAGATGGTGGACCAATGGGTGGAGGGAGGTTCGATGGACCTAAATCTGGTGCCCCACAAATGGAAGGACGCAGACAAAATGGCGGTCCGATGGGTGGTAGG----------------------------------------------------------------------------------- 293  
2-2403    CTAGGCAAGATGGTGGACAAATGGGTGGAAGGAGGTTCGATGGACCTGAATCTGGTGCCCCACAAATGGAAGGACGCAGACAAAATGGCGGTCCGATGGGTGGTAGG----------------------------------------------------------------------------------- 294  
2-2448    CTAGGCAAGATGGTGGACCAATGGGTGGAAGGAGGTTCGATGGACCTGAATCTGGTGCCCCACAAATGGAAGGACGCAGACAAAATGGCGGTCCGATGGGTGGTAGG----------------------------------------------------------------------------------- 293  
2-2404    CTAGGCAAGATGGTGGACCAATGGGTGGAAGGAGGTTCGATGGACCTGAATCTGGTGCCCCACAAATGGAAGGACGCAGACAAAATGGCGGTCCGATGGGTGGTAGG----------------------------------------------------------------------------------- 294  
2-2405    CTAGGCAAGATGGTGGACCAATGGGTGGAAGG------------------------------------------------------------------------------------------------------------------------------------------------------AGGTTCGA 230  
2-2406    CTAGGCAAGATGGTGGACCAATGGGTGGAAGGAGGTTCGATGGACCTGAATCTGGTGCCCCACAAATGGAAGGACGCAGACAAAATGGCGGTCCGATGGGTGGTAGG----------------------------------------------------------------------------------- 291  
2-2409    CTAGGCAAGATGGTGGACCAATGGGTGGAAGGAGGTTCGATGGACCTGAATCTGGTGCCCCACAAATGGAAGGACGCAGACAAAATGGCGGTCCGATGGGTGGTAGG----------------------------------------------------------------------------------- 293  
2-2410    CTAGGCAAGATGGTGGACCAATGGGTGGAAGGAGGTTCGATGGACCTGAATCTGGTGCCCCACAAATGGAAGGACGCAGACAAAATGGCGGTCCGATGGGTGGTAGG----------------------------------------------------------------------------------- 294  
2-2411    CTAGGCAAGATGGTGGACCAATGGGTGGAAGGAGGTTCGATGGACCTGAATCTGGTGCCCCACAAATGGAAGGACGCAGACAAAATGGCGGTCCGATGGGTGGTAGG----------------------------------------------------------------------------------- 294  
2-2412    CTAGGCAAGATGGTGGACCAATGGGTGGAAGGAGGTTCGATGGACCTGAATCTGGTGCCCCACAAATGGAAGGACGCAGACAAAATGGCGGTCCGATGGGTGGTAGG----------------------------------------------------------------------------------- 293  
2-2413    CTAGGCAAGATGGTGGACCAATGGGTGGAAGGAGGTTCGATGGACCTGAATCTGGTGCCCCACAAATGGAAGGACGCAGACAAAATGGCGGTCCGATGGGTGGTAGG----------------------------------------------------------------------------------- 293  
2-2415    CTAGGCAAGATGGTGGACAAATGGGTGGAAGGAGGTTCGATGGACCTGAATCTGGTGCCCCACAAATGGAAGGACGCAGACAAAATGGCGGTCCGATGGGTGGTAGG----------------------------------------------------------------------------------- 294  
2-2416    CTAGGCAAGATGGTGGACAAATGGGTGGAAGGAGGTTCGATGGACCTGAATCTGGTGCCCCACAAATGGAAGGACGCAGACAAAATGGCGGTCCGATGGGTGGTAGG----------------------------------------------------------------------------------- 293  
2-2417    CTAGGCAAGATGGTGGACCAATGGGTGGAAGGAGGTTCGATGGACCTGAATCTGGTGCCCCACAAATGGAAGGACGCAGACAAAATGGCGGTCCGATGGGTGGTAGG----------------------------------------------------------------------------------- 293  
2-2418    CTAGGCAAGATGGTGGACCAATGGGTGGAAGGAGGTTCGATGGACCTGAATCTGGTGCCCCACAAATGGAAGGACGCAGACAAAATGGCGGTCCGATGGGTGGTAGG----------------------------------------------------------------------------------- 293  
2-2419    CTAGGCAAGATGGTGGACCAATGGGTGGAAGGAGGTTCGATGGACCTGAATCTGGTGCCCCACAAATGGATGGACGCAGACAAAATGGCGGTCCGATGGGTGGTAGG----------------------------------------------------------------------------------- 293  
2-2420    CTAGGCAAGATGGTGGACCAATGGGTGGAAGGAGGTTCGATGGACCTGAATCTGGTGCCCCACAAATGGAAGGACGCAGACAAAATGGCGGTCCGATGGGTGGTAGG----------------------------------------------------------------------------------- 294  
2-2421    CTAGGCAAGATGGTGGACCAATGGGTGGAAGGAGGTTCGATGGACCTGAATCTGGTGCCCCACAAATGGAAGGACGCAGACAAAATGGCGGTCCGATGGGTGGTAGG----------------------------------------------------------------------------------- 293  
2-2422    CTAGGCAAGATGGTGGACCAATGGGTGGAAGGAGGTTCGATGGACCTGAATCTGGTGCCCCACAAATGGAAGGACGCAGACAAAATGGCGGTCCGATGGGTGGTAGG----------------------------------------------------------------------------------- 293  
2-2424    CTAGGCAAGATGGTGGACCAATGGGTGGAAGGAGGTTCGATGGACCTGAATCTGGTGCCCCACAAATGGAAGGACGCAGACAAAATGGCGGTCCGATGGGTGGTAGG----------------------------------------------------------------------------------- 295  
2-2425    CTAGGCAAGATGGTGGACCAATGGGTGGAAGGAGGTTCGATGGACCTGAATCTGGTGCCCCACAAATGGAAGGACGCAGACAAAATGGCGGTCCGATGGGTGGTAGG----------------------------------------------------------------------------------- 293  
2-2426    CTAGGCAAGATGGTGGACCAATGGGTGGAAGGAGGTTCGATGGACCTGAATCTGGTGCCCCACAAATGGAAGGACGCAGACAAAATGGCGGTCCGATGGGTGGTAGG----------------------------------------------------------------------------------- 294  
2-2427    CTAGGCAAGATGGTGGACCAATGGGTGGAAGGAGGTTCGATGGACCTGAATCTGGTGCCCCACAAATGGAAGGACGCAGACAAAATGGCGGTCCGATGGGTGGTAGG----------------------------------------------------------------------------------- 294  
2-2430    CTAGGCAAGATGGTGGACCAATGGGTGGAAGGAGGTTCGATGGACCTGAATCTGGTGCCCCACAAATGGAAGGACGCAGACAAAATGGCGGTCCGATGGGTGGTAGG----------------------------------------------------------------------------------- 293  
2-2431    CTAGGCAAGATGGTGGACCAATGGGTGGAAGGAGGTTCGATGGACCTGAATCTGGTGCCCCACAAATGGAAGGACGCAGACAAAATGGCGGTCCGATGGGTGGTAGG----------------------------------------------------------------------------------- 286  
2-2432    CTAGGCAAGATGGTGGACCAATGGGTGGAAGGAGGTTCGATGGACCTGAATCTGGTGCCCCACAAATGGAAGGACGCAGACAAAATGGCGGTCCGATGGGTGGTAGG----------------------------------------------------------------------------------- 293  
2-2434    CTAGGCAAGATGGTGGACCAATGGGTGGAAGGAGGTTCGATGGACCTGAATCTGGTGCCCCACAAATGGAAGGACGCAGACAAAATGGCGGTCCGATGGGTGGTAGG----------------------------------------------------------------------------------- 293  
2-2437    CTAGGCAAGATGGTGGACCAATGGGTGGAAGGAGGTTCGATGGACCTGAATCTGGTGCCCCACAAATGGAAGGACGCAGACAAAATGGCGGTCCGATGGGTGGTAGG----------------------------------------------------------------------------------- 294  
2-2438    CTAGGCAAGATGGTGGACAAATGGGTGGAAGGAGGTTCGATGGACCTGAATCTGGTGCCCCACAAATGGAAGGACGCAGACAAAATGGCGGTCCGATGGGTGGTAGG----------------------------------------------------------------------------------- 293  
2-2439    CTAGGCAAGATGGTGGACCAATGGGTGGAAGGAGGTTCGATGGACCTGAATCTGGTGCCCCACAAATGGAAGGACGCAGACAAAATGGCGGTCCGATGGGTGGTAGG----------------------------------------------------------------------------------- 293  
2-2440    CTAGGCAAGATGGTGGACCAATGGGTGGAAGGAGGTTCGATGGACCTGAATCTGGTGCCCCACAAATGGAAGGACGCAGACAAAATGGCGGTCCGATGGGTGGTAGG----------------------------------------------------------------------------------- 291  
2-2442    CTAGGCAAGATGGTGGACCAATGGGTGGAAGGAGGTTCGATGGACCTGAATCTGGTGCCCCACAAATGGAAGGACGCAGACAAAATGGCGGTCCGATGGGTGGTAGG----------------------------------------------------------------------------------- 292  
2-2445    CTAGGCAAGATGGTGGACAAATGGGTGGAAGGAGGTTCGATGGACCTGAATCTGGTGCCCCACAAATGGAAGGACGCAGACAAAATGGCGGTCCGATGGGTGGTAGG----------------------------------------------------------------------------------- 290  
2-2446    CTAGGCAAGATGGTGGACAAATGGGTGGAAGGAGGTTCGATGGACCTGAATCTGGTGCCCCACAAATGGAAGGACGCAGACAAAATGGCGGTCCGATGGGTGGTAGG----------------------------------------------------------------------------------- 293  
7-1501    CTAGGCAAGATGGTGGACCAATGGGTGGAGGGAGGTTCGATGGACCTGAATCTGGTGCCCCACAAATGGAAGGACGCAGACAAAATGGCGGTCCGATGGGTGGTAGG----------------------------------------------------------------------------------- 293  
7-1502    CTAGGCAAGATGGTGGACCAATGGGTGGAGGGAGGTTCGATGGACCTGAATCTGGTGCCCCACAAATGGAAGGACGCAGACAAAATGGCGGTCCGATGGGTGGTAGG----------------------------------------------------------------------------------- 293  
7-1503    CTAGGCAAGATGGTGGACCAATGGGTGGAGGGAGGTTCGATGGACCTGAATCTGGTGCCCCACAAATGGAAGGACGCAGACAAAATGGCGGTCCGATGGGTGGTAGG----------------------------------------------------------------------------------- 293  
7-1504    CTAGGCAAGATGGTGGACCAATGGGTGGAGGGAGGTTCGATGGACCTGAATCTGGTGCCCCACAAATGGAAGGACGCAGACAAAATGGCGGTCCGATGGGTGGTAGG----------------------------------------------------------------------------------- 293  
7-1505    CTAGGCAAGATGGTGGACCAATGGGTGGAGGGAGGTTCGATGGACCTGAATCTGGTGCCCCACAAATGGAAGGACGCAGACAAAATGGCGGTCCGATGGGTGGTAGG----------------------------------------------------------------------------------- 293  
7-1506    CTAGGCAAGATGGTGGACCAATGGGTGGAGGGAGGTTCGATGGACCTGAATCTGGTGCCCCACAAATGGAAGGACGCAGACAAAATGGCGGTCCGATGGGTGGTAGG----------------------------------------------------------------------------------- 293  
7-1508    CTAGGCAAGATGGTGGACCAATGGGTGGAGGGAGGTTCGATGGACCTGAATCTGGTGCCCCACAAATGGAAGGACGCAGACAAAATGGCGGTCCGATGGGCGGTAGG----------------------------------------------------------------------------------- 293  
7-1509    CTAGGCAAGATGGTGGACCAATGGGTGGAGGGAGGTTCGATGGACCTGAATCTGGTGCCCCACAAATGGAAGGACGCAGACAAAATGGCGGTCCGATGGGTGGTAGG----------------------------------------------------------------------------------- 293  
7-1510    CTAGGCAAGATGGTGGACCAATGGGTGGAGGGAGGTTCGATGGACCTGAATCTGGTGCCCCACAAATGGAAGGACGCAGACAAAATGGCGGTCCGATGGGTGGTAGG----------------------------------------------------------------------------------- 293  
7-1511    CTAGGCAAGATGGTGGACCAATGGGTGGAGGGAGGTTCGATGGACCTGAATCTGGTGCCCCACAAATGGAAGGACGCAGACAAAATGGCGGTCCGATGGGTGGTAGG----------------------------------------------------------------------------------- 293  
7-1512    CTAGGCAAGATGGTGGACCAATGGGTGGAGGGAGGTTCGATGGACCTGAATCTGGTGCCCCACAAATGGAAGGACGCAGACAAAATGGCGGTCCGATGGGTGGTAGG----------------------------------------------------------------------------------- 293  
7-1513    CTAGGCAAGATGGTGGACCAATGGGTGGAGGGAGGTTCGATGGACCTGAATCTGGTGCCCCACAAATGGAAGGACGCAGACAAAATGGCGGTCCGATGGGTGGTAGG----------------------------------------------------------------------------------- 293  
7-1515    CTAGGCAAGATGGTGGACCAATGGGTGGAGGGAGGTTCGATGGACCTGAATCTGGTGCCCCACAAATGGAAGGACGCAGACAAAATGGCGGTCCGATGGGTGGTAGG----------------------------------------------------------------------------------- 293  
7-1516    CTAGGCAAGATGGTGGACCAATGGGTGGAGGGAGGTTCGATGGACCTGAATCTGGTGCCCCACAAATGGAAGGACGCAGACAAAATGGCGGTCCGATGGGTGGTAGG----------------------------------------------------------------------------------- 293  
7-1517    CTAGGCAAGATGGTGGACCAATGGGTGGAGGGAGGTTCGATGGACCTGAATCTGGTGCCCCACAAATGGAAGGACGCAGACAAAATGGCGGTCCGATGGGTGGTAGG----------------------------------------------------------------------------------- 291  
7-1519    CTAGGCAGGATGGTGGACCAATGGGTGGAGGGAGGTTCGATGGACCTGAATCTGGTGCCCCACAAATGGAAGGACGCAGACAAAATGGCGGTCCGATGGGTGGTAGG----------------------------------------------------------------------------------- 293  
7-1520    CTAGGCAAGATGGTGGACCAATGGGTGGAGGGAGGTTCGATGGACCTGAATCTGGTGCCCCTCAAATGGAAGGACGCAGACAAAATGGCGGTCCGATGGGTGGTAGG----------------------------------------------------------------------------------- 293  
7-1521    CTAGGCAAGATGGTGGACCAATGGGTGGAGGGAGGTTCGATGGACCTGAATCTGGTGCCCCACAAATGGAAGGACGCAGACAAAATGGCGGTCCGATGGGTGGTAGG----------------------------------------------------------------------------------- 293  
7-1523    CTAGGCAAGATGGTGGACCAATGGGTGGAGGGAGGTTCGATGGACCTGAATCTGGTGCCCCACAAATGGAAGGACGCAGACAAAATGGCGGTCCGATGGGTGGTAGG----------------------------------------------------------------------------------- 293  
7-1524    CTAGGCAAGATGGTGGACCAATGGGTGGAGGGAGGTTCGATGGACCTGAATCTGGTGCCCCACAAATGGAAGGACGCAGACAAAATGGCGGTCCGATGGGTGGTAGG----------------------------------------------------------------------------------- 293  
7-1525    CTAGGCAAGATGGTGGACCAATGGGTGGAGGGAGGTTCGATGGACCTGAATCTGGTGCCCCACAAATGGAAGGACGCAGACAAAATGGCGGTCCGATGGGTGGTAGG----------------------------------------------------------------------------------- 293  
7-1526    CTAGGCAAGATGGTGGACCAATGGGTGGAGGGAGGTTCGATGGACCTGAATCTGGTGCCCCACAAATGGAAGGACGCAGACAAAATGGCGGTCCGATGGGTGGTAGG----------------------------------------------------------------------------------- 293  
7-1527    CTAGGCAAGATGGTGGACCAATGGGTGGAGGGAGGTTCGATGGACCTGAATCTGGTGCCCCACAAATGGAAGGACGCAGACAAAATGGCGGTCCGATGGGTGGTAGG----------------------------------------------------------------------------------- 292  
7-1528    CTAGGCAAGATGGTGGACCAATGGGTGGAGGGAGGTTCGATGGACCTGAATCTGGTGCCCCACAAATGGAAGGACGCAGACAAAATGGCGGTCCGATGGGTGGTAGG----------------------------------------------------------------------------------- 293  
7-1529    CTAGGCAAGATGGTGGACCAATGGGTGGAGGGAGGTTCGATGGACCTGAATCTGGTGCCCCACAAATGGAAGGACGCAGACAAAATGGCGGTCCGATGGGTGGTAGG----------------------------------------------------------------------------------- 293  
7-1530    CTAGGCAAGATGGTGGACCAATGGGTGGAGGGAGGTTCGATGGACCTGAATCTGGTGCCCCACAAATGGAAGGACGCAGACAAAATGGCGGTCCGATGGGTGGTAGG----------------------------------------------------------------------------------- 293  
7-1533    CTAGGCAAGATGGTGGACCAATGGGTGGAGGGAGGTCCGATGGACCTGAATCTGGTGCCCCACAAATGGAAGGACGCAGACAAAATGGCGGTCCGATGGGTGGTAGG----------------------------------------------------------------------------------- 293  
7-1534    CTAGGCAAGATGGTGGACCAATGGGTGGAGGGAGGTTCGATGGACCTGAATCTGGTGCCCCACAAATGGAAGGACGCAGACAAAATGGCGGTCCGATGGGTGGTAGG----------------------------------------------------------------------------------- 293  
7-1536    CTAGGCAAGATGGTGGACCAATGGGTGGAGGGAGGTTCGATGGACCTGAATCTGGTGCCCCACAAATGGAAGGACGCAGACAAAATGGCGGTCCGATGGGTGGTAGG----------------------------------------------------------------------------------- 293  
7-1537    CTAGGCAAGATGGTGGACCAATGGGTGGAGGGAGGTTCGATGGACCTGAATCTGGTGCCCCACAAATGGAAGGACGCAGACAAAATGGCGGTCCGATGGGTGGTAGG----------------------------------------------------------------------------------- 293  
7-1538    CTAGGCAAGATGGTGGACCAATGGGTGGAGGGAGGTTCGATGGACCTGAATCTGGTGCCCCACAAATGGAAGGACGCAGACAAAATGGCGGTCCGATGGGTGGTAGG----------------------------------------------------------------------------------- 293  
7-1542    CTAGGCAAGATGGTGGACCAATGGGTGGAGGGAGGTTCGATGGACCTGAATCTGGTGCCCCACAAATGGAAGGACGCAGACAAAATGGCGGTCCGATGGGTGGTAGG----------------------------------------------------------------------------------- 293  
7-1544    CTAGGCAAGATGGTGGACCAATGGGTGGAGGGAGGTTCGATGGACCTGAATCTGGTGCCCCACAAATGGAAGGACGCAGACAAAATGGCGGTCCGATGGGTGGTAGG----------------------------------------------------------------------------------- 293  
7-1545    CTAGGCAAGATGGTGGACCAATGGGTGGAGGGAGGTTCGATGGACCTGAATCTGGTGCCCCACAAATGGAAGGACGCAGACAAAATGGCGGTCCGATGGGTGGTAGG----------------------------------------------------------------------------------- 293  
7-1546    CTAGGCAAGATGGTGGACCAATGGGTGGAGGGAGGTTCGATGGACCTGAATCTGGTGCCCCACAAATGGAAGGACGCAGACAAAATGGCGGTCCGATGGGTGGTAGG----------------------------------------------------------------------------------- 293  
7-1548    CTAGGCAAGATGGTGGACCAATGGGTGGAGGGAGGTTCGATGGACCTGAATCTGGTGCCCCACAAATGGAAGGACGCAGACAAAATGGCGGTCCGATGGGTGGTAGG----------------------------------------------------------------------------------- 293  
7-1549    CTAGGCAAGATGGTGGACCAATGGGTGGAGGGAGGTTCGATGGACCTGAATCTGGTGCCCCACAAATGGAAGGACGCAGACAAAATGGCGGTCCGATGGGTGGTAGG----------------------------------------------------------------------------------- 293  
7-1550    CTAGGCAAGATGGTGGACCAATGGGTGGAGGGAGGTTCGATGGACCTGAATCTGGTGCCCCACAAATGGAAGGACGCAGACAAAATGGCGGTCCGATGGGTGGTAGG----------------------------------------------------------------------------------- 293  
7-1539    CTAGGCAAGATGGTGGACCAATGGGTGGAGGGAGGTTCGATGGACCTGAATCTGGTGCCCCACAAATGGAAGGACGCAGACAAAATGGCGGTCCGATGGGTGGTAGG----------------------------------------------------------------------------------- 293  
7-1540    CTAGGCAAGATGGTGGACCAATGGGTGGAGGGAGGTTCGATGGACCTGAATCTGGTGCCCCACAAATGGAAGGACGCTGACAAAATGGCGGTCCGATGGGTGGTAGG----------------------------------------------------------------------------------- 293  
7-1514    CTAGGCAAGATGGTGGACAAATGGGTGGAAGGAGGTTCGATGGACCTGAATCTGGTGCCCCACAAATGGAAGGACGCAGACAAAATGGCGGTCCGATGGGTGGTAGG----------------------------------------------------------------------------------- 291  
7-1507    TTAGGCAAGATGGTGGACCAATGGGTGGAAGGAGGTTCGATGGACCTGAATCTGGTGCCCCACAAATGGAAGGACGCAGACAAAATGGCGGTCCGATGGGTGGTAGG----------------------------------------------------------------------------------- 293  
7-1518    CTAGGCAAGATGGTGGACAAATGGGTGGAAGGAGGTTCGATGGACCTGAATCTGGTGCCCCACAAATGGAAGGACGCAGACAAAATGGCGGTCCGATGGGTGGTAGG----------------------------------------------------------------------------------- 291  
7-1522    CTAGGCAAGATGGTGGACAAATGGGTGGAAGGAGGTTCGATGGACCTGAATCTGGTGCCCCACAAATGGAAGGACGCAGACAAAATGGCGGTCCGATGGGTGGTAGG----------------------------------------------------------------------------------- 291  
7-1532    CTAGGCAAGATGGTGGACAAATGGGTGGAAGGAGGTTCGATGGACCTGAATCTGGTGCCCCACAAATGGAAGGACGCAGACAAAATGGCGGTCCGATGGGTGGTAGG----------------------------------------------------------------------------------- 293  
7-1543    CTAGGCAAGATGGTGGACAAATGGGTGGAAGGAGGTTCGATGGACCTGAATCTGGTGCCCCACAAATGGAAGGACGCAGACAAAATGGCGGTCCGATGGGTGGTAGG----------------------------------------------------------------------------------- 291  
7-1547    TTAGGCAAGATGGTGGACCAATGGGTGGAAGGAGGTTCGATGGACCTGAATCTGGTGCCCCACAAATGGAAGGACGCAGACAAAATGGCGGTCCGATGGGTGGTAGG----------------------------------------------------------------------------------- 293  
7-2401    CTAGGCAAGATGGTGGACCAATGGGTGGAGGGAGGTTCGATGGACCTGAATCTGGTGCCCCACAAATGGAAGGACGCAGACAAAATGGCGGTCCGATGGGTGGTAGG----------------------------------------------------------------------------------- 293  
7-2402    CTAGGCAAGATGGTGGACCAATGGGTGGAGGGAGGTTCGATGGACCTGAATCTGGTGCCCCACAAATGGAAGGACGCAGACAAAATGGCGGTCCGATGGGTGGTAGG----------------------------------------------------------------------------------- 293  
7-2403    CTAGGCAAGATGGTGGACCAATGGGTGGAGGGAGGTTCGATGGACCTGAATCTGGTGCCCCACAAATGGAAGGACGCAGACAAAATGGCGGTCCGATGGGTGGTAGG----------------------------------------------------------------------------------- 291  
7-2404    CTAGGCAAGATGGTGGACCAATGGGTGGAGGGAGGTTCGATGGACCTGAATCTGGTGCCCCACAAATGGAAGGACGCAGACAAAATGGCGGTCCGATGGGTGGTAGG----------------------------------------------------------------------------------- 293  
7-2405    CTAGGCAAGATGGTGGACCAATGGGTGGAGGGAGGTTCGATGGACCTGAATCTGGTGCCCCACAAATGGAAGGACGCAGACAAAATGGCGGTCCGATGGGTGGTAGG----------------------------------------------------------------------------------- 293  
7-2406    CTAGGCAAGATGGTGGACCAATGGGTGGAGGGAGGTTCGATGGACCTGAATCTGGTGCCCCACAAATGGAAGGACGCAGACAAAATGGCGGTCCGATGGGTGGTAGG----------------------------------------------------------------------------------- 293  
7-2407    CTAGGCAAGATGGTGGACCAATGGGTGGAGGGAGGTTCGATGGACCTGAATCTGGTGCCCCACAAATGGAAGGACGCAGACAAAATGGCGGTCCGATGGGTGGTAGG----------------------------------------------------------------------------------- 293  
7-2408    CTAGGCAAGATGGTGGACCAATGGGTGGAGGGAGGTTCGATGGACCTGAATCTGGTGCCCCACAAATGGAAGGACGCAGACAAAATGGCGGTCCGATGGGTGGTAGG----------------------------------------------------------------------------------- 293  
7-2409    CTAGGCAAGATGGTGGACCAATGGGTGGAGGGAGGTTCGATGGACCTGAATCTGGTGCCCCACAAATGGAAGGACGCAGACAAAATGGCGGTCCGATGGGTGGTAGG----------------------------------------------------------------------------------- 293  
7-2410    CTAGGCAAGATGGTGGACCAATGGGTGGAGGGAGGTTCGATGGACCTGAATCTGGTGCCCCACAAATGGAAGGACGCAGACAAAATGGCGGTCCGATGGGTGGTAGG----------------------------------------------------------------------------------- 293  
7-2411    CTAGGCAAGATGGTGGACCAATGGGTGGAGGGAGGTTCGATGGACCCGAATCTGGTGCCCCACAAATGGAAGGACGCAGACAAAATGGCGGTCCGATGGGTGGTAGG----------------------------------------------------------------------------------- 293  
7-2412    CTAGGCAAGATGGTGGACCAATGGGTGGAGGGAGGTTCGATGGACCTGAATCTGGTGCCCCACAAATGGAAGGACGCAGACAAAATGGCGGTCCGATGGGTGGTAGG----------------------------------------------------------------------------------- 293  
7-2413    CTAGGCAAGATGGTGGACCAATGGGTGGAGGGAGGTTCGATGGACCTGAATCTGGTGCCCCACAAATGGAAGGACGCAGACAAAATGGCGGTCCGATGGGTGGTAGG----------------------------------------------------------------------------------- 293  
7-2414    CTAGGCAAGATGGTGGACCAATGGGTGGAGGGAGGTTCGATGGACCTGAATCTGGTGCCCCACAAATGGAAGGACGCAGACAAAATGGCGGTCCGATGGGTGGTAGG----------------------------------------------------------------------------------- 293  
7-2416    CTAGGCAAGATGGTGGACCAATGGGTGGAGGGAGGTTCGATGGACCTGAATCTGGTGCCCCACAAATGGAAGGACGCAGACAAAATGGCGGTCCGATGGGTGGTAGG----------------------------------------------------------------------------------- 293  
7-2417    CTAGGCAAGATGGTGGACCAATGGGTGGAGGGAGGTTCGATGGACCTGAGTCTGGTGCCCCACAAATGGAAGGACGCAGACAAAATGGCGGTCCGATGGGTGGTAGG----------------------------------------------------------------------------------- 293  
7-2418    CTAGGCAAGATGGTGGACCAATGGGTGGAGGGAGGTTCGATGGACCTGAATCTGGTGCCCCACAAATGGAAGGACGCAGACAAAATGGCGGTCCGATGGGTGGTAGG----------------------------------------------------------------------------------- 293  
7-2420    CTAGGCAAGATGGTGGACCAATGGGTGGAGGGAGGTTCGATGGACCTGAATCTGGTGCCCCACAAATGGAAGGACGCAGACAAAATGGCGGTCCGATGGGTGGTAGG----------------------------------------------------------------------------------- 293  
7-2421    CTAGGCAAGATGGTGGACCAATGGGTGGAGGGAGGTTCGATGGACCTGAATCTGGTGCCCCACAAATGGAAGGACGCAGACAAAATGGCGGTCCGATGGGTGGTAGG----------------------------------------------------------------------------------- 293  
7-2423    CTAGGCAAGATGGTGGACCAATGGGTGGAGGGAGGTTCGATGGACCTGAATCTGGTGCCCCACAAATGGAAGGACGCAGACAAAATGGCGGTCCGATGGGTGGTAGG----------------------------------------------------------------------------------- 293  
7-2424    CTAGGCAAGATGGTGGACCAATGGGTGGAGGGAGGTTCGATGGACCTGAATCTGGTGCCCCACAAATGGAAGGACGCAGACAAAATGGCGGTCCGATGGGTGGTAGG----------------------------------------------------------------------------------- 293  
7-2425    CTAGGCAAGATGGTGGACCAATGGGTGGAGGGAGGTTCGATGGACCTGAATCTGGTGCCCCACAAATGGAAGGACGCAGACAAAATGGCGGTCCGATGGGTGGTAGG----------------------------------------------------------------------------------- 293  
7-2426    CTAGGCAAGATGGTGGACCAATGGGTGGAGGGAGGTTCGATGGACCTGAATCTGGTGCCCCACAAATGGAAGGACGCAGACAAAATGGCGGTCCGATGGGTGGTAGG----------------------------------------------------------------------------------- 293  
7-2430    CTAGGCAAGATGGTGGACCAATGGGTGGAGGGAGGTTCGATGGACCTGAATCTGGTGCCCCACAAATGGAAGGACGCAGACAAAATGGCGGTCCGATGGGTGGTAGG----------------------------------------------------------------------------------- 293  
7-2431    CTAGGCAAGATGGTGGACCAATGGGTGGAGGGAGGTTCGATGGACCTGAATCTGGTGCCCCACAAATGGAAGGACGCAGACAAAATGGCGGTCCGATGGGTGGTAGG----------------------------------------------------------------------------------- 293  
7-2432    CTAGGCAAGATGGTGGACCAATGGGTGGAGGGAGGTTCGATGGACCTGAATCTGGTGCCCCACAAATGGAAGGACGCAGACAAAATGGCGGTCCGATGGGTGGTAGG----------------------------------------------------------------------------------- 293  
7-2436    CTAGGCAAGATGGTGGACCAATGGGTGGAGGGAGGTTCGATGGACCTAAATCTGGTGCCCCACAAATGGAAGGACGCAGACAAAATGGCGGTCCGATGGGTGGTAGG----------------------------------------------------------------------------------- 293  
7-2437    CTAGGCAAGATGGTGGACCAATGGGTGGAGGGAGGTTCGATGGACCTGAATCTGGTGCCCCACAAATGGAAGGACGCAGACAAAATGGCGGTCCGATGGGTGGTAGG----------------------------------------------------------------------------------- 293  
7-2439    CTAGGCAAGATGGTGGACCAATGGGTGGAGGGAGGTTCGATGGACCTGAATCTGGTGCCCCACAAATGGAAGGACGCAGACAAAATGGCGGTCCGATGGGTGGTTGG----------------------------------------------------------------------------------- 293  
7-2442    CTAGGCAAGATGGTGGACCAATGGGTGGAGGGAGGTTCGATGGACCTGAATCTGGTGCCCCACAAATGGAAGGACGCAGACAAAATGGCGGTCCGATGGGTGGTAGG----------------------------------------------------------------------------------- 293  
7-2443    CTAGGCAAGATGGTGGACCAATGGGTGGAGGGAGGTTCGATGGACCTGAATCTGGTGCCCCACAAATGGAAGGACGCAGACAAAATGGCGGTCCGATGGGTGGTAGG----------------------------------------------------------------------------------- 293  
7-2444    CTAGGCAAGATGGTGGACCAATGGGTGGAGGGAGGTTCGATGGACCTGAATCTGGTGCCCCACAAATGGAAGGACGCAGACAAAATGGCGGTCCGATGGGTGGTAGG----------------------------------------------------------------------------------- 293  
7-2445    CTAGGCAAGATGGTGGACCAATGGGTGGAGGGAGGTTCGATGGACCTGAATCTGGTGCCCCACAAATGGAAGGACGCAGACAAAATGGCGGTCCGATGGGTGGTAGG----------------------------------------------------------------------------------- 293  
7-2446    CTAGGCAAGATGGTGGACCAATGGGTGGAGGGAGGTTCGATGGACCTGAATCTGGTGCCCCACAAATGGAAGGACGCAGACAAAATGGCGGTCCGATGGGTGGTAGG----------------------------------------------------------------------------------- 293  
7-2447    CTAGGCAAGATGGTGGACCAATGGGTGGAGGGAGGTTCGATGGACCTGAATCTGGTGCCCCACAAATGGAAGGACGCAGACAAAATGGCGGTCCGATGGGTGGTAGG----------------------------------------------------------------------------------- 293  
7-2448    CTAGGCAAGATGGTGGACCAATGGGTGGAGGGAGGTTCGATGGACCTGAATCTGGTGCCCCACAAATGGAAGGACGCAGACAAAATGGCGGTCCGATGGGTGGTAGG----------------------------------------------------------------------------------- 293  
7-2450    CTAGGCAAGATGGTGGACCAATGGGTGGAGGGAGGTTCGATGGACCTGAATCTGGTGCCCCACAAATGGAAGGACGCAGACAAAATGGCGGTCCGATGGGTGGTAGG----------------------------------------------------------------------------------- 293  
7-2415    TTAGGCAAGATGGTGGACCAATGGGTGGAAGGAGGTTCGATGGACCTGAATCTGGTGCCCCACAAATGGAAGGACGCAGACAAAATGGCGGTCCGATGGGTGGTAGG----------------------------------------------------------------------------------- 293  
7-2427    CTAGGCAAGATGGTGGACCAATGGGTGGAAGGAGGTTCGATGGACCTGAATCTGGTGCCCCACAAATGGAAGGACGCAGACAAAATGGCGGTCCGATGGGTGGTAGG----------------------------------------------------------------------------------- 293  
7-2428    CTAGGCAAGATGGTGGACCAATGGGTGGAAGGAGATTCGATGGACCTGAATCTGGTGCCCCACAAATGGAAGGACGCAGACAAAATGGCGGTCCGATGGGTGGTAGG----------------------------------------------------------------------------------- 291  
7-2435    CTAGGCAAGATGGTGGACAAATGGGTGGAAGGAGGTTCGATGGACCTGAATCTGGTGCCCCACAAATGGAAGGACGCAGACAAAATGGCGGTCCGATGGGTGGTAGG----------------------------------------------------------------------------------- 291  
7-2440    CTAGGCAAGATGGTGGACCAATGGGTGGAAGGAGGTTCGATGGACCTGAATCTGGTGCCCCACAAATGGAAGGACGCAGACAAAATGGCGGTCCGATGGGTGGTAGG----------------------------------------------------------------------------------- 293  
7-2441    CTAGGCAAGATGGTGGACAAATGGGTGGAAGGAGGTTCGATGGACCTGAATCTGGTGCCCCACAAATGGAAGGACGCAGACAAAATGGCGGTCCGATGGGTGGTAGG----------------------------------------------------------------------------------- 291  
7-2449    CTAGGCAAGATGGTGGACAAATGGGTGGAAGGAGGTTCGATGGACCTGAATCTGGTGCCCCACAAATGGAAGGACGCAGACAAAATGGCGGTCCGATGGGTGGTAGG----------------------------------------------------------------------------------- 291  
8-1501    CTAGGCAAGATGGTGGACCAATGGGTGGAGGGAGGTTCGATGGACCTGAATCTGGTGCCCCACAAATGGAAGGACGCAGACAAAATGGCGGTCCGATGGGTGGTAGG----------------------------------------------------------------------------------- 293  
8-1502    CTAGGCAAGATGGTGGACCAATGGGTGGAGGGAGGTTCGATGGACCTGAATCTGGTGCCCCACAAATGGAAGGACGCAGACAAAATGGCGGTCCGATGGGTGGTAGG----------------------------------------------------------------------------------- 293  
8-1503    CTAGGCAAGATGGTGGACCAATGGGTGGAGGGAGGTTCGATGGACCTGAATCTGGTGCCCCACAAATGGAAGGACGCAGACAAAATGGCGGTCCGATGGGTGGTAGG----------------------------------------------------------------------------------- 292  
8-1504    CTAGGCAAGATGGTGGACCAATGGGTGGAGGGAGGTTCGATGGACCTGAATCTGGTGCCCCACAAATGGAAGGACGCAGACAAAATGGCGGTCCGATGGGTGGTAGG----------------------------------------------------------------------------------- 293  
8-1505    CTAGGCAAGATGGTGGACCAATGGGTGGAGGGAGGTTCGATGGACCTGAATCTGGTGCCCCACAAATGGAAGGACGCAGACAAAATGGCGGTCCGATGGGTGGTAGG----------------------------------------------------------------------------------- 293  
8-1506    CTAGGCAAGATGGTGGACCAATGGGTGGAGGGAGGTTCGATGGACCTGAATCTGGTGCCCCACAAATGGAAGGACGCAGACAAAATGGCGGTCCGATGGGTGGTAGG----------------------------------------------------------------------------------- 293  
8-1507    CTAGGCAAGATGGTGGACCAATGGGTGGAGGGAGGTTCGATGGACCTGAATCTGGTGCCCCACAAATGGAAGGACGCAGACAAAATGGCGGTCCGATGGGTGGTAGG----------------------------------------------------------------------------------- 293  
8-1508    CTAGGCAAGATGGTGGACCAATGGGTGGAGGGAGGTTCGATGGACCTGAATCTGGTGCCCCACAAATGGAAGGACGCAGACAAAATGGCGGTCCGATGGGTGGTAGG----------------------------------------------------------------------------------- 293  
8-1509    CTAGGCAAGATGGTGGACCAATGGGTGGAGGGAGGTTCGATGGACCTGAATCTGGTGCCCCACAAATGGAAGGACGCAGACAAAATGGCGGTCCGATGGGTGGTAGG----------------------------------------------------------------------------------- 293  
8-1510    CTAGGCAAGATGGTGGACCAATGGGTGGAGGGAGGTTCGATGGACCTGAATCTGGTGCCCCACAAATGGAAGGACGCAGACAAAATGGCGGTCCGATGGGTGGTAGG----------------------------------------------------------------------------------- 293  
8-1511    CTAGGCAAGATGGTGGACCAATGGGTGGAGGGAGGTTCGATGGACCTGAATCTGGTGCCCCACAAATGGAAGGACGCAGACAAAATGGCGGTCCGATGGGTGGTAGG----------------------------------------------------------------------------------- 293  
8-1512    CTAGGCAAGATGGTGGACCAATGGGTGGAGGGAGGTTCGATGGACCTGAATCTGGTGCCCCACAAATGGAAGGACGCAGACAAAATGGCGGTCCGATGGGTGGTAGG----------------------------------------------------------------------------------- 293  
8-1513    CTAGGCAAGATGGTGGACCAATGGGTGGAGGGAGGTTCGATGGACCTGAATCTGGTGCCCCACAAATGGAAGGACGCAGACAAAATGGCGGTCCGATGGGTGGTAGG----------------------------------------------------------------------------------- 293  
8-1514    CTAGGCAAGATGGTGGACCAATGGGTGGAGGGAGGTTCGATGGACCTGAATCTGGTGCCCCACAAATGGAAGGACGCAGACAAAATGGCGGTCCGATGGGTGGTAGG----------------------------------------------------------------------------------- 293  
8-1515    CTAGGCAAGATGGTGGACCAATGGGTGGAGGGAGGTTCGATGGACCTGAATCTGGTGCCCCACAAATGGAAGGACGCAGACAAAATGGCGGTCCGATGGGTGGTAGG----------------------------------------------------------------------------------- 293  
8-1516    CTAGGCAAGATGGTGGACCAATGGGTGGAGGGAGGTTCGATGGACCTGAATCTGGTGCCCCACAAATGGAAGGACGCAGACAAAATGGCGGTCCGATGGGTGGTAGG----------------------------------------------------------------------------------- 293  
8-1517    CTAGGCAAGATGGTGGACCAATGGGTGGAGGGAGGTTCGATGGACCTGAATCTGGTGCCCCACAAATGGAAGGACGCAGACAAAATGGCGGTCCGATGGGTGGTAGG----------------------------------------------------------------------------------- 293  
8-1518    CTAGGCAAGATGGAGGACCAATGGGTGGAGGGAGGTTCGATGGACCTGAATCTGGTGCCCCACAAATGGAAGGACGCAGACAAAATGGCGGTCCGATGGGTGGTAGG----------------------------------------------------------------------------------- 293  
8-1519    CTAGGCAAGATGGTGGACCAATGGGTGGAGGGAGGTTCGATGGACCTGAATCTGGTGCCCCACAAATGGAAGGACGCAGACAAAATGGCGGTCCGATGGGTGGTAGG----------------------------------------------------------------------------------- 293  
8-1520    CTAGGCAAGATGGTGGACCAATGGGTGGAGGGAGGTTCGATGGACCTGAATCTGGTGCCCCACAAATGGAAGGACGCAGACAAAATGGCGGTCCGATGGGTGGTAGG----------------------------------------------------------------------------------- 293  
8-1521    CTAGGCAAGATGGTGGACCAATGGGTGGAGGGAGGTTCGATGGACCTGAATCTGGTGCCCCACAAATGGAAGGACGCAGACAAAATGGCGGTCCGATGGGTGGTAGG----------------------------------------------------------------------------------- 293  
8-1522    CTAGGCAAGATGGTGGACCAATGGGTGGAGGGAGGTTCGATGGACCTGAATCTGGTGCCCCACAAATGGAAGGACGCAGACAAAATGGCGGTCCGATGGGTGGTAGG----------------------------------------------------------------------------------- 293  
8-1524    CTAGGCAAGATGGTGGACCAATGGGTGGAGGGAGGTTCGATGGACCTGAATCTGGTGCCCCACAAATGGAAGGACGCAGACAAAATGGCGGTCCGATGGGTGGTAGG----------------------------------------------------------------------------------- 293  
8-1525    CTAGGCAAGATGGTGGACCAATGGGTGGAGGGAGGTTCGATGGACCTGAATCTGGTGCCCCACAAATGGAAGGACGCAGACAAAATGGCGGTCCGATGGGTGGTAGG----------------------------------------------------------------------------------- 293  
8-1526    CTAGGCAAGATGGTGGACCAATGGGTGGAGGGAGGTTCGATGGACCTGAATCTGGTGCCCCACAAATGGAAGGACGCAGACGAAATGGCGGTCCGATGGGTGGTAGG----------------------------------------------------------------------------------- 293  
8-1527    CTAGGCAAGATGGTGGACCAATGGGTGGAGGGAGGTTCGATGGACCTGAATCTGGTGCCCCACAAATGGAAGGACGCAGACAAAATGGCGGTCCGATGGGCGGTAGG----------------------------------------------------------------------------------- 293  
8-1528    CTAGGCAAGATGGTGGACCAATGGGTGGAGGGAGGTTCGATGGACCTGAATCTGGTGCCCCACAAATGGAAGGACGCAGACAAAATGGCGGTCCGATGGGTGGTAGG----------------------------------------------------------------------------------- 293  
8-1529    CTAGGCAAGATGGTGGACCAATGGGTGGAGGGAGGTTCGATGGACCTGAATCTGGTGCCCCACAAATGGAAGGACGCAGACAAAATGGCGGTCCGATGGGTGGTAGG----------------------------------------------------------------------------------- 293  
8-1530    CTAGGCAAGATGGTGGACCAATGGGTGGAGGGAGGTTCGATGGACCTGAATCTGGTGCCCCACAAATGGAAGGACGCAGACAAAATGGCGGTCCGATGGGTGGTAGG----------------------------------------------------------------------------------- 293  
8-1531    CTAGGCAAGATGGTGGACCAATGGGTGGAGGGAGGTTCGATGGACCTGAATCTGGTGCCCCACAAATGGAAGGACGCAGACAAAATGGCGGTCCGATGGGTGGTAGG----------------------------------------------------------------------------------- 293  
8-1532    CTAGGCAAGATGGTGGACCAATGGGTGGAGGGAGGTTCGATGGACCTGAATCTGGTGCCCCACAAATGGAAGGACGCAGACAAAATGGCGGTCCGATGGGTGGTAGG----------------------------------------------------------------------------------- 293  
8-1533    CTAGGCAAGATGGTGGACCAATGGGTGGAGGGAGGTTCGATGGACCTGAATCTGGTGCCCCACAAATGGAAGGACGCAGACAAAATGGCGGTCCGATGGGTGGTAGG----------------------------------------------------------------------------------- 293  
8-1534    CTAGGCAAGATGGTGGACCAATGGGTGGAGGGAGGTTCGATGGACCTGAATCTGGTGCCCCACAAATGGAAGGACGCAGACAAAATGGCGGTCCGATGGGTGGTAGG----------------------------------------------------------------------------------- 293  
8-1535    CTAGGCAAGATGGTGGACCAATGGGTGGAGGGAGGTTCGATGGACCTGAATCTGGTGCCCCACAAATGGAAGGACGCAGACAAAATGGCGGTCCGATGGGTGGTAGG----------------------------------------------------------------------------------- 293  
8-1536    CTAGGCAAGATGGTGGACCAATGGGTGGAGGGAGGTTCGATGGACCTGAATCTGGTGCCCCACAAATGGAAGGACGCAGACAAAATGGCGGTCCGATGGGTGGTAGG----------------------------------------------------------------------------------- 293  
8-1537    CTAGGCAAGATGGTGGACCAATGGGTGGAGGGAGGTTCGATGGACCTGAATCTGGTGCCCCACAAATGGAAGGACGCAGACAAAATGGCGGTCCGATGGGTGGTAGG----------------------------------------------------------------------------------- 293  
8-1538    CTAGGCAAGATGGTGGACCAATGGGTGGAGGGAGGTTCGATGGACCTGAATCTGGTGCCCCACAAATGGAAGGACGCAGACAAAATGGCGGTCCGATGGGTGGTAGG----------------------------------------------------------------------------------- 293  
8-1539    CTAGGCAAGATGGTGGACCAATGGGTGGAGGGAGGTTCGATGGACCTGAATCTGGTGCCCCACAAATGGAAGGACGCAGACAAAATGGCGGTCCGATGGGTGGTAGG----------------------------------------------------------------------------------- 293  
8-1540    CTAGGCAAGATGGTGGACCAATGGGTGGAGGGAGGTTCGATGGACCTGAATCTGGTGCCCCACAAATGGAAGGACGCAGACAAAATGGCGGTCCGATGGGTGGTAGG----------------------------------------------------------------------------------- 293  
8-1542    CTAGGCAAGATGGTGGACCAATGGGTGGAGGGAGGTTCGATGGACCTGAATCTGGTGCCCCACAAATGGAAGGACGCAGACAAAATGGCGGTCCGATGGGTGGTAGG----------------------------------------------------------------------------------- 293  
8-1546    CTAGGCAAGATGGTGGACCAATGGGTGGAGGGAGGTTCGATGGACCTGAATCTGGTGCCCCACAAATGGAAGGACGCAGACAAAATGGCGGTCCGATGGGTGGTAGG----------------------------------------------------------------------------------- 293  
8-1548    CTAGGCAAGATGGTGGACCAATGGGTGGAGGGAGGTTCGATGGACCTGAATCTGGTGCCCCACAAATGGAAGGACGCAGACAAAATGGCGGTCCGATGGGTGGTAGG----------------------------------------------------------------------------------- 293  
8-1549    CTAGGCAAGATGGTGGACCAATGGGTGGAGGGAGGTTCGATGGACCTGAATCTGGTGCCCCACAAATGGAAGGACGCAGACAAAATGGCGGTCCGATGGGTGGTAGG----------------------------------------------------------------------------------- 293  
8-1550    CTAGGCAAGATGGTGGACCAATGGGTGGAGGGAGGTTCGATGGACCTGAATCTGGTGCCCCACAAATGGAAGGACGCAGACAAAATGGCGGTCCGATGGGTGGTAGG----------------------------------------------------------------------------------- 291  
8-1541    CTAGGCAAGATGGTGGACCAATGGGTGGAAGGAGGTTCGATGGACCTGAATCTGGTGCCCCACAAATGGAAGGACGCAGACAAAATGGCGGTCCGATGGGTGGTAGG----------------------------------------------------------------------------------- 293  
8-1543    CTAGGCAAGATGGTGGACCAATGGGTGGAAGGAGGTTCGATGGACCTGAATCTGGTGCCCCACAAATGGAAGGACGCAGACAAAATGGCGGTCCGATGGGTGGTAGG----------------------------------------------------------------------------------- 293  
8-2401    CTAGGCAAGATGGTGGACCAATGGGTGGAGGGAGGTTCGATGGACCTGAATCTGGTGCCCCACAAATGGAAGGACGCAGACAAAATGGCGGTCCGATGGGTGGTAGG----------------------------------------------------------------------------------- 293  
8-2406    CTAGGCAAGATGGTGGACCAATGGGTGGAGGGAGGTTCGATGGACCTGAATCTGGTGCCCCACAAATGGAAGGACGCAGACAAAATGGCGGTCCGATGGGTGGTAGG----------------------------------------------------------------------------------- 293  
8-2407    CTAGGCAAGATGGTGGACCAATGGGTGGAGGGAGGTTCGATGGACCTGAATCTGGTGCCCCACAAATGGAAGGACGCAGACAAAATGGCGGTCCGATGGGTGGTAGG----------------------------------------------------------------------------------- 293  
8-2409    CTAGGCAAGATGGTGGACCAATGGGTGGAGGGAGGTTCGATGGACCTGAATCTGGTGCCCCACAAATGGAAGGACGCAGACAAAATGGCGGTCCGATGGGTGGTAGG----------------------------------------------------------------------------------- 293  
8-2410    CTAGGCAAGATGGTGGACCAATGGGTGGAGGGAGGTTCGATGGACCTGAATCTGGTGCCCCACAAATGGAAGGACGCAGACAAAATGGCGGTCCGATGGGTGGTAGG----------------------------------------------------------------------------------- 293  
8-2411    CTAGGCAAGATGGTGGACCAATGGGTGGAGGGAGGTTCGATGGACCTGAATCTGGTGCCCCACAAATGGAAGGACGCAGACAAAATGGCGGTCCGATGGGTGGTAGG----------------------------------------------------------------------------------- 293  
8-2414    CTAGGCAAGATGGTGGACCAATGGGTGGAGGGAGGTTCGATGGACCTGAATCTGGTGCCCCACAAATGGAAGGACGCAGACAAAATGGCGGTCCGATGGGTGGTAGG----------------------------------------------------------------------------------- 293  
8-2417    CTAGGCAAGATGGTGGACCAATGGGTGGAGGGAGGTTCGATGGACCTGAATCTGGTGCCCCACAAATGGAAGGACGCAGACAAAATGGCGGTCCGATGGGTGGTAGG----------------------------------------------------------------------------------- 293  
8-2420    CTAGGCAAGATGGTGGACCAATGGGTGGAGGGAGGTTCGATGGACCTGAATCTGGTGCCCCACAAATGGAAGGACGCAGACAAAATGGCGGTCCGATGGGTGGTAGG----------------------------------------------------------------------------------- 293  
8-2421    CTAGGCAAGATGGTGGACCAATGGGTGGAGGGAGGTTCGATGGACCTGAATCTGGTGCCCCACAAATGGAAGGACGCAGACAAAATGGCGGTCCGATGGGTGGTAGG----------------------------------------------------------------------------------- 293  
8-2422    CTAGGCAAGATGGTGGACCAATGGGTGGAGGGAGGTTCGATGGACCTGAATCTGGTGCCCCACAAATGGAAGGACGCAGACAAAATGGCGGTCCGATGGGTGGTAGG----------------------------------------------------------------------------------- 293  
8-2424    CTAGGCAAGATGGTGGACCAATGGGTGGAGGGAGGTTCGATGGACCTGAATCTGGTGCCCCACAAATGGAAGGACGCAGACAAAATGGCGGTCCGATGGGTGGTAGG----------------------------------------------------------------------------------- 293  
8-2425    CTAGGCAAGATGGTGGACCAATGGGTGGAGGGAGGTTCGATGGACCTGAATCTGGTGCCCCACAAATGGAAGGACGCAGACAAAATGGCGGTCCGATGGGTGGTAGG----------------------------------------------------------------------------------- 293  
8-2427    CTAGGCAAGATGGTGGACCAATGGGTGGAGGGAGGTTCGATGGACCTGAATCTGGTGCCCCACAAATGGAAGGACGCAGACAAAATGGCGGTCCGATGGGTGGTAGG----------------------------------------------------------------------------------- 293  
8-2433    CTAGGCAAGATGGTGGACCAATGGGTGGAGGGAGGTTCGATGGACCTGAATCTGGTGCCCCACAAATGGAAGGACGCAGACAAAATGGCGGTCCGATGGGTGGTAGG----------------------------------------------------------------------------------- 293  
8-2434    CTAGGCAAGATGGTGGACCAATGGGTGGAGGGAGGTTCGATGGACCTGAATCTGGTGCCCCACAAATGGAAGGACGCAGACAAAATGGCGGTCCGATGGGTGGTAGG----------------------------------------------------------------------------------- 293  
8-2435    CTAGGCAAGATGGTGGACCAATGGGTGGAGGGAGGTTCGATGGACCTGAATCTGGTGCCCCACAAATGGAAGGACGCAGACAAAATGGCGGTCCGATGGGTGGTAGG----------------------------------------------------------------------------------- 293  
8-2436    CTAGGCAAGATGGTGGACCAATGGGTGGAGGGAGGTTCGATGGACCTGAATCTGGTGCCCCACAAATGGAAGGACGCAGACAAAATGGCGGTCCGATGGGTGGTAGG----------------------------------------------------------------------------------- 293  
8-2437    CTAGGCAAGATGGTGGACCAATGGGTGGAGGGAGGTTCGATGGACCTGAATCTGGTGCCCCACAAATGGAAGGACGCAGACAAAATGGCGGTCCGATGGGTGGTAGG----------------------------------------------------------------------------------- 293  
8-2438    CTAGGCAAGATGGTGGACCAATGGGTGGAGGGAGGTTCGATGGACCTGAATCTGGTGCCCCACAAATGGAAGGACGCAGACAAAATGGCGGTCCGATGGGTGGTAGG----------------------------------------------------------------------------------- 293  
8-2439    CTAGGCAAGATGGTGGACCAATGGGTGGAGGGAGGTTCGATGGACCTGAATCTGGTGCCCCACAAATGGAAGGACGCAGACAAAATGGCGGTCCGATGGGTGGTAGG----------------------------------------------------------------------------------- 293  
8-2440    CTAGGCAAGATGGTGGACCAATGGGTGGAGGGAGGTTCGATGGACCTGAATCTGGTGCCCCACAAATGGAAGGACGCAGACAAAATGGCGGTCCGATGGGTGGTAGG----------------------------------------------------------------------------------- 293  
8-2442    CTAGGCAAGATGGTGGACCAATGGGTGGAGGGAGGTTCGATGGACCTGAATCTGGTGCCCCACAAATGGAAGGACGCAGACAAAATGGCGGTCCGATGGGTGGTAGG----------------------------------------------------------------------------------- 293  
8-2444    CTAGGCAAGATGGTGGACCAATGGGTGGAGGGAGGTTCGATGGACCTGAATCTGGTGCCCCACAAATGGAAGGACGCAGACAAAATGGCAGTCCGATGGGTGGTAGG----------------------------------------------------------------------------------- 293  
8-2445    CTAGGCAAGATGGTGGACCAATGGGTGGAGGGAGGTTCGATGGACCTGAATCTGGTGCCCCACAAATGGAAGGACGCAGACAAAATGGCGGTCCGATGGGTGGTAGG----------------------------------------------------------------------------------- 293  
8-2448    CTAGGCAAGATGGTGGACCAATGGGTGGAGGGAGGTTCGATGGACCTGAATCTGGTGCCCCACAAATGGAAGGACGCAGACAAAATGGCGGTCCGATGGGTGGTAGG----------------------------------------------------------------------------------- 293  
8-2449    CTAGGCAAGATGGTGGACCAATGGGTGGAGGGAGGTTCGATGGACCTGAATCTGGTGCCCCACAAATGGAAGGACGCAGACAAAATGGCGGTCCGATGGGTGGTAGG----------------------------------------------------------------------------------- 293  
8-2450    CTAGGCAAGATGGTGGACCAATGGGTGGAGGGAGGTTCGATGGACCTGAATCTGGTGCCCCACAAATGGAAGGACGCAGACAAAATGGCGGTCCGATGGGTGGTAGG----------------------------------------------------------------------------------- 293  
8-2432    CGAGGCAAGATGGTGGACCAATGGGTGGAAGAAGGTTCGATGGACCTGACTCTGGTGCCCCACAAATGGATGGACGGAGACAAGATGGTGGACCAATGGGTGGAAGG---------------------------------------------------------------------------AGGTTCGA 298  
8-2405    CGAGGCAAGATGGTGGACCAGTGGGTGGAAGAAGGTTCGATGGACCTGACTCTGGTGCCCCACAAATGGATGGACGGAGACAAGATGGTGGACCAATGGGTGGAAGG---------------------------------------------------------------------------AGGTTCGA 298  
8-2443    CTAGGCAAGATGGTGGACCAATGGGTGGAAGGAGGTTCGATGGACCTGAATCTGGTGCCCCACAAATGGAAGGACGCAGACAAAATGGCGGTCCGATGGGTGGTAGG----------------------------------------------------------------------------------- 293  
8-2413    CTAGGCAAGATGGTGGACAAATGGGTGGAAGGAGGTTCGATGGACCTGAATCTGGTGCCCCACAAATGGAAGGACGCAGACAAAATGGCGGTCCGATGGGTGGTAGG----------------------------------------------------------------------------------- 293  
8-2418    TTAGGCAAGATGGTGGACCAATGGGTGGAAGGAGGTTCGATGGACCTGAATCTGGTGCCCCACAAATGGAAGGACGCAGACAAAATGGCGGTCCGATGGGTGGTAGG----------------------------------------------------------------------------------- 293  
8-2419    CTAGGCAAGATGGTGGACCAATGGGTGGAAGGAGGTTCGATGGACCTGAATCTGGTGCCCCACAAATGGAAGGACGCAGACAAAATGGCGGTCCGATGGGTGGTAGG----------------------------------------------------------------------------------- 293  
8-2423    CTAGGCAAGATGGTGGACCAATGGGTGGAAGGAGGTTCGATGGACCTGAATCTGGTGCCCCACAAATGGAAGGACGCAGACAAAATGGCGGTCCGATGGGTGGTAGG----------------------------------------------------------------------------------- 293  
8-2430    CTAGGCAAGATGGTGGACAAATGGGTGGAAGGAGGTTCGATGGACCTGAATCTGGTGCCCCACAAATGGAAGGACGCAGACAAAATGGCGGTCCGATGGGTGGTAGG----------------------------------------------------------------------------------- 291  
8-2446    CTAGGCAAGATGGTGGACCAATGGGTGGAAGGAGGTTCGATGGACCTGAATCTGGTGCCCCACAAATGGAAGGACGCAGACAAAATGGCGGTCCGATGGGTGGTAGG----------------------------------------------------------------------------------- 293  
8-2447    CTAGGCAAGATGGTGGACCAATGGGTGGAAGGAGGTTCGATGGACCTGAATCTGGTGCCCCACAAATGGAAGGACGCAGACAAAATGGCGGTCCGATGGGTGGTAGG----------------------------------------------------------------------------------- 293  
8-2441    CTAGGCAAGATGGTGGACCAATGGGTGGAATGAGGTTCGATGGACCTGAATCTGGTGCCCCACAAATGGATGGACGCAGACAAAATGGCGGTCCGATGGGTGGTAGG----------------------------------------------------------------------------------- 290  
8-2404    CTAGGCAAGATGGTGGACCAATGGGTGGAAGGAGATTCGATGGACCTGAATCTGGTGCCCCACAAATGGATGGACGCAGACAAAATGGCGGTCCGATGGGTGGTAGG----------------------------------------------------------------------------------- 291  
8-2415    CTAGGCAAGATGGTGGACCAATGGGTGGAAGGAGGTTCGATGGACCTGAATCTGGTGCCCCACAAATGGAAGGACGCAGACAAAATGGCGGTCCGATGGGTGGTAGG----------------------------------------------------------------------------------- 293  
2-1517    CTAGGCAAGATGGTGGACCAATGGGTGGAGGGAGGTTCGATGGACCTGAATCTGGTGCCCCACAAATGGAAGGACGCAGACAAAATGGCGGTCCGATGGGTGGTAGG----------------------------------------------------------------------------------- 293  
2-1501    CTAGGCAAGATGGTGGACCAATGGGTGGAGGGAGGTTCGATGGACCTGAATCTGGTGCCCCACAAATGGAAGGACGCAGACAAAATGGCGGTCCGATGGGTGGTAGG----------------------------------------------------------------------------------- 293  
2-1503    CTAGGCAAGATGGTGGACCAATGGGTGGAGGGAGGTTCGATGGACCTGAATCTGGTGCCCCACAAATGGAAGGACGCAGACAAAATGGCGGTCCGATGGGTGGTAGG----------------------------------------------------------------------------------- 293  
2-1505    CTAGGCAAGATGGTGGACCAATGGGTGGAGGGAGGTTCGATGGACCTGAATCTGGTGCCCCACAAATGGAAGGACGCAGACAAAATGGCGGTCCGATGGGTGGTAGG----------------------------------------------------------------------------------- 293  
2-1508    CTAGGCAAGATGGTGGACCAATGGGTGGAGGGAGGTTCGATGGACCTGAATCTGGTGCCCCACAAATGGAAGGACGCAGACAAAATGGCGGTCCGATGGGTGGTAGG----------------------------------------------------------------------------------- 293  
2-1509    CTAGGCAAGATGGTGGACCAATGGGTGGAGGGAGGTTCGATGGACCTGAATCTGGTGCCCCACAAATGGAAGGACGCAGACAAAATGGCGGTCCGATGGGTGGTAGG----------------------------------------------------------------------------------- 293  
2-1510    CTAGGCAAGATGGTGGACCAATGGGTGGAGGGAGGTTCGATGGACCTGAATCTGGTGCCCCACAAATGGAAGGACGCAGACAAAATGGCGGTCCGATGGGTGGTAGG----------------------------------------------------------------------------------- 293  
2-1519    CTAGGCAAGATGGTGGACCAATGGGTGGAGGGAGGTTCGATGGACCTGAATCTGGTGCCCCACAAATGGAAGGACGCAGACAAAATGGCGGTCCGATGGGTGGTAGG----------------------------------------------------------------------------------- 293  
2-1521    CTAGGCAAGATGGTGGACCAATGGGTGGAGGGAGGTTCGATGGACCTGAATCTGGTGCCCCACAAATGGAAGGACGCAGACAAAATGGCGGTCCGATGGGTGGTAGG----------------------------------------------------------------------------------- 293  
2-1522    CTAGGCAAGATGGTGGACCAATGGGTGGAGGGAGGTTCGATGGACCTGAATCTGGTGCCCCACAAATGGAAGGACGCAGACAAAATGGCGGTCCGATGGGTGGTAGG----------------------------------------------------------------------------------- 293  
2-1523    CTAGGCAAGATGGTGGACCAATGGGTGGAGGGAGGTTCGATGGACCTGAATCTGGTGCCCCACAAATGGAAGGACGCAGACAAAATGGCGGTCCGATGGGTGGTAGG----------------------------------------------------------------------------------- 293  
2-1527    CTAGGCAAGATGGTGGACCAATGGGTGGAGGGAGGTTCGATGGACCTGAATCTGGTGCCCCACAAATGGAAGGACGCAGACAAAATGGCGGTCCGATGGGTGGTAGG----------------------------------------------------------------------------------- 293  
2-1533    CTAGGCAAGATGGTGGACCAATGGGTGGAGGGAGGTTCGATGGACCTGAATCTGGTGCCCCACAAATGGAAGGGCGCAGACAAAATGGCGGTCCGATGGGTGGTAGG----------------------------------------------------------------------------------- 293  
2-1535    CTAGGCAAGATGGTGGACCAATGGGTGGAGGGAGGTTCGATGGACCTGAATCTGGTGCCCCACAAATGGAAGGACGCAGACAAAATGGCGGTCCGATGGGTGGTAGG----------------------------------------------------------------------------------- 293  
2-1536    CTAGGCAAGATGGTGGACCAATGGGTGGAGGGAGGTTCGATGGACCTGAATCTGGTGCCCCACAAATGGAAGGACGCAGACAAAATGGCGGTCCGATGGGTGGTAGG----------------------------------------------------------------------------------- 293  
2-1537    CTAGGCAAGATGGTGGACCAATTGGTGGAGGGAGGTTCGATGGACCTGAATCTGGTGCCCCACAAATGGAAGGACGCAGACAAAATGGCGGTCCGATGGGTGGTAGG----------------------------------------------------------------------------------- 292  
2-1538    CTAGGCAAGATGGTGGACCAATGGGTGGAGGGAGGTTCGATGGACCTGAATCTGGTGCCCCACAAATGGAAGGACGCAGACAAAATGGCGGTCCGATGGGTGGTAGG----------------------------------------------------------------------------------- 293  
2-1539    CTAGGCAAGATGGTGGACCAATGGGTGGAGGGAGGTTCGATGGACCTGAATCTGGTGCCCCACAAATGGAAGGACGCAGACAAAATGGCGGTCCGATGGGTGGTAGG----------------------------------------------------------------------------------- 293  
2-1543    CTAGGCAAGATGGTGGACCAATGGGTGGAGGGAGGTTCGATGGACCTGAATCTGGTGCCCCACAAATGGAAGGACGCAGACAAAATGGCGGTCCGATGGGTGGTAGG----------------------------------------------------------------------------------- 293  
2-1546    CTAGGCAAGATGGTGGACCAATGGGTGGAGGGAGGTTCGATGGACCTGAATCTGGTGCCCCACAAATGGAAGGACGCAGACAAAATGGCGGTCCGATGGGTGGTAGG----------------------------------------------------------------------------------- 293  
2-1547    CTAGGCAAGATGGTGGACCAATGGGTGGAGGGAGGTTCGATGGACCTGAATCTGGTGCCCCACAAATGGAAGGACGCAGACAAAATGGCGGTCCGATGGGTGGTAGG----------------------------------------------------------------------------------- 293  
2-1529    CAAGGCAAGATGGCGGTCCGATGGGTGGTAGG---------------------------------------------------------------------------AGATTCGACGGACATGGATTTGGTGCCCCGCCGATGGGTGGACCAAGGCAAGATGGTGGACCAATGGGTGGAAGGAGGTTCGA 299  
2-1540    CGAGGCAAGATGGTGGACCAATGGGTGGAAGAAGGTTCGATGGACCTGACTCTGGTGCCCCACAAATGGATGGACGGAGACAAGATGGTGGACCAATGGGTGGAAGG---------------------------------------------------------------------------AGGTTCGA 298  
2-1506    CTAGGCAAGATGGTGGACCAATGGGTGGAAGGAGGTTCGATGGACCTGAATCTGGTGCCCCACAAATGGAAGGACGCAGACAAAATGGCGGTCCGATGGGTGGTAGG----------------------------------------------------------------------------------- 293  
2-1507    CTAGGCAAGATGGTGGACAAATGGGTGGAAGGAGGTTCGATGGACCTGAATCTGGTGCCCCACAAATGGAAGGACGCAGACAAAATGGCGGTCCGATGGGTGGTAGG----------------------------------------------------------------------------------- 291  
2-1511    CTAGGCAAGATGGTGGACAAATGGGTGGAAGGAGGTTCGATGGACCTGAATCTGGTGCCCCACAAATGGAAGGACGCAGACAAAATGGCGGCCCGATGGGTGGTAGG----------------------------------------------------------------------------------- 291  
2-1514    CTAGGCAAGATGGTGGACCAATGGGTGGAAGGAGGTTCGATGGACCTGAATCTGGTGCCCCACAAATGGATGGACGCAGACAAAATGGCGGTCCGATGGGTGGTAGG----------------------------------------------------------------------------------- 293  
2-1516    CTAGGCAAGATGGTGGACCAATGGGTGGAAGGAGGTTCGATGGACCTGAATCTGGTGCCCCACAAATGGAAGGACGCAGACAAAATGGCGGTCCGATGGGTGGTAGG----------------------------------------------------------------------------------- 293  
2-1528    CTAGGCAAGATGGTGGACCAATGGGTGGAAGGAGGTTCGATGGACCTGAATCTGGTGCCCCACAAATGGAAGGACGCAGACAAAATGGCGGTCCGATGGGTGGTAGG----------------------------------------------------------------------------------- 293  
2-1532    CTAGGCAAGATGGTGGACCAATGGGTGGAAGGAGGTTCGATGGACCTGAATCTGGTGCCCCACAAATGGAAGGACGCAGACAAAATGGCGGTCCGATGGGTGGTAGG----------------------------------------------------------------------------------- 293  
2-1541    CTAGGCAAGATGGTGGACAAATGGGTGGAAGGAGGTTCGATGGACCTGAATCTGGTGCCCCACAAATGGAAGGACGCAGACAAAATGGCGGTCCGATGGGTGGTAGG----------------------------------------------------------------------------------- 291  
2-1542    CTAGGCAAGATGGTGGACCAATGGGTGGAAGGAGGTTCGATGGACCTGAATCTGGTGCCCCACAAATGGAAGGACGCAGACAAAATGGCGGTCCGATGGGTGGTAGG----------------------------------------------------------------------------------- 293  
2-2448    CTAGGCAAGATGGTGGACCAATGGGTGGAGGGAGGTTCGATGGACCTGAATCTGGTGCCCCACAAATGGAAGGACGCAGACAAAATGGCGGTCCGATGGGTGGTAGG----------------------------------------------------------------------------------- 293  
2-2405    CTAGGCAAGATGGTGGACCAATGGGTGGAGGGAGGTTCGATGGACCTGAATCTGGTGCCCCACAAATGGAAGGACGCAGACAAAATGGCGGTCCGATGGGTGGTAGG----------------------------------------------------------------------------------- 293  
2-2406    CTAGGCAAGATGGTGGACCAATGGGTGGAGGGAGGTTCGATGGACCTGAATCTGGTGCCCCACAAATGGAAGGACGCAGACAAAATGGCGGTCCGATGGGTGGTAGG----------------------------------------------------------------------------------- 293  
2-2407    CTAGGCAAGATGGTGGACCAATGGGTGGAGGGAGGTTCGATGGACCTGAATCTGGTGCCCCACAAATGGAAGGACGCAGACAAAATGGCGGTCCGATGGGTGGTAGG----------------------------------------------------------------------------------- 293  
2-2408    CTAGGCAAGATGGTGGACCAATGGGTGGAGGGAGGTTCGATGGACCTGAATCTGGTGCCCCACAAATGGAAGGACGCAGACAAAATGGCGGTCCGATGGGTGGTAGG----------------------------------------------------------------------------------- 293  
2-2410    CTAGGCAAGATGGTGGACCAATGGGTGGAGGGAGGTTCGATGGACCTGAATCTGGTGCCCCACAAATGGAAGGACGCAGACAAAATGGCGGTCCGATGGGTGGTAGG----------------------------------------------------------------------------------- 293  
2-2412    CTAGGCAAGATGGTGGACCAATGGGTGGAGGGAGGTTCGATGGACCTGAATCTGGTGCCCCACAAATGGAAGGACGCAGACAAAATGGCGGTCCGATGGGTGGTAGG----------------------------------------------------------------------------------- 293  
2-2413    CTAGGCAAGATGGTGGACCAATGGGTGGAGGGAGGTTCGATGGACCTGAATCTGGTGCCCCACAAATGGAAGGACGCAGACAAAATGGCGGTCCGATGGGTGGTAGG----------------------------------------------------------------------------------- 293  
2-2419    CTAGGCAAGATGGTGGACCAATGGGTGGAGGGAGGTTCGATGGACCTGAATCTGGTGCCCCACAAATGGAAGGACGCAGACAAAATGGCGGTCCGATGGGTGGTAGG----------------------------------------------------------------------------------- 293  
2-2420    CTAGGCAAGATGGTGGACCAATGGGTGGAGGGAGGTTCGATGGACCTGAATCTGGTGCCCCACAAATGGAAGGACGCAGACAAAATGGCGGTCCGATGGGTGGTAGG----------------------------------------------------------------------------------- 293  
2-2422    CTAGGCAAGATGGTGGACCAATGGGTGGAGGGAGGTTCGATGGACCTGAATCTGGTGCCCCACAAATGGAAGGACGCAGACAAAATGGCGGTCCGATGGGTGGTAGG----------------------------------------------------------------------------------- 293  
2-2424    CTAGGCAAGATGGTGGACCAATGGGTGGAGGGAGGTTCGATGGACCTGAATCTGGTGCCCCACAAATGGAAGGACGCAGACAAAATGGCGGTCCGATGGGTGGTAGG----------------------------------------------------------------------------------- 293  
2-2427    CTAGGCAAGATGGTGGACCAATGGGTGGAGGGAGGTTCGATGGACCTGAATCTGGTGCCCCACAAATGGAAGGACGCAGACAAAATGGCGGTCCGATGGGTGGTAGG----------------------------------------------------------------------------------- 293  
2-2428    CTAGGCAAGATGGTGGACCAATGGGTGGAGGGAGGTTCGATGGACCTGAATCTGGTGCCCCACAAATGGAAGGACGCAGACAAAATGGCGGTCCGATGGGTGGTAGG----------------------------------------------------------------------------------- 293  
2-2429    CTAGGCAAGATGGTGGACCAATGGGTGGAGGGAGGTTCGATGGACCTGAATCTGGTGCCCCACAAATGGAAGGACGCAGACAAAATGGCGGTCCGATGGGTGGTAGG----------------------------------------------------------------------------------- 293  
2-2431    CTAGGCAAGATGGTGGACCAATGGGTGGAGGGAGGTTCGATGGACCTGAATCTGGTGCCCCACAAATGGAAGGACGCAGACAAAATGGCGGTCCGATGGGTGGTAGG----------------------------------------------------------------------------------- 293  
2-2436    CTAGGCAAGATGGTGGACCAATGGGTGGAGGGAGGTTCGATGGACCTGAATCTGGTGCCCCACAAATGGAAGGACGCAGACAAAATGGCGGTCCGATGGGTGGTAGG----------------------------------------------------------------------------------- 293  
2-2441    CTAGGCAAGATGGTGGACCAATGGGTGGAGGGAGGTTCGATGGACCTGAATCTGGTGCCCCACAAATGGAAGGACGCAGACAAAATGGCGGTCCGATGGGTGGTAGG----------------------------------------------------------------------------------- 292  
2-2446    CTAGGCAAGATGGTGGACCAATGGGTGGAGGGAGGTTCGATGGACCTGAATCTGGTGCCCCACAAATGGAAGGACGCAGACAAAATGGCGGTCCGATGGGTGGTAGG----------------------------------------------------------------------------------- 293  
2-2416    CTAGGCAAGATGGTGGACCAATGGGTGGAGGGAGGTTCGATGGACCTGAATCTGGTGCCCCACAAATGGAAGGACGCAGACAAAATGGCGGTCCGATGGGTGGTAGG----------------------------------------------------------------------------------- 292  
2-2411    ---------------------------------------------------------------------------------------------------------------------------------------------------------------------------------------------- 161  
2-2415    ---------------------------------------------------------------------------------------------------------------------------------------------------------------------------------------------- 161  
2-2440    ---------------------------------------------------------------------------------------------------------------------------------------------------------------------------------------------- 161  
2-2423    ---------------------------------------------------------------------------------------------------------------------------------------------------------------------------------------------- 17   
2-2418    CTAGGCAAGATGGTGGACCAATGGGTGGAATGAGGTTCGATGGACCTGAATCTGGTGCCCCACAAATGGATGGACGCAGACAAAATGGCGGTCCGATGGGTGGTAGG----------------------------------------------------------------------------------- 290  
2-2421    CTAGGCAAGATGGTGGACCAATGGGTGGAATGAGGTTCGATGGACCTGAATCTGGTGCCCCACAAATGGATGGACGCAGACAAAATGGCGGTCCGATGGGTGGTAGG----------------------------------------------------------------------------------- 293  
2-2438    CTAGGCAAGATGGTGGACCAATGGGTGGAGGGAGGTTCGATGGACCTGAATCTGGTGCCCCGCAAATGGAAGGACGCAGACAAAATGGCGGTCCGATGGGTGGTAGG----------------------------------------------------------------------------------- 293  
2-2409    CGAGGCAAGATGGTGGACCAATGGGTGGAAGAAGGTTCGATGGACCTGACTCTGGTGCCCCACAAATGGATGGACGGAGACAAGATGGTGGACCAATGGGTGGAAGG---------------------------------------------------------------------------AGGTTCGA 297  
2-2444    CGAGGCAAGATGGTGGACCAATGGGTGGAAGAAGGTTCGATGGACCTGACTCTGGTGCCCCACAAATGGATGGACGGAGACAAGATGGTGGACCAATGGGTGGAAGG---------------------------------------------------------------------------AGGTTCGA 298  
2-2414    CTAGGCAAGATGGTGGACCAATGGGTGGAAGGAGGTTCGATGGACCTGAATCTGGTGCCCCACAAATGGAAGGACGCAGACAAAATGGCGGTCCGATGGGTGGTAGG----------------------------------------------------------------------------------- 293  
2-2403    CTAGGCAAGATGGTGGACCAATGGGTGGAAGGAGGTTCGATGGACCTGAATCTGGTGCCCCACAAATGGAAGGACGCAGACAAAATGGCGGTCCGATGGGTGGTAGG----------------------------------------------------------------------------------- 293  
2-2404    CTAGGCAAGATGGTGGACCAATGGGTGGAATGAGGTTCGATGGACCTGAATCTGGTGCCCCACAAATGGATGGACGCAGACAAAATGGCGGTCCGATGGGTGGTAGG----------------------------------------------------------------------------------- 290  
2-2425    CTAGGCAAGATGGTGGACCAATGGGTGGAATGAGGTTCGATGGACCTGAATCTGGTGCCCCACAAATGGATGGACGCAGACAAAATGGCGGTCCGATGGGTGGTAGG----------------------------------------------------------------------------------- 290  
2-2426    CTAGGCAAGATGGTGGACCAATGGGTGGAAGGAGGTTCGATGGACCTGAATCTGGTGCCCCACAAATGGAAGGACGCAGACAAAATGGCGGTCCGATGGGTGGTAGG----------------------------------------------------------------------------------- 293  
2-2430    CTAGGCAAGATGGTGGACCAATGGGTGGAAGGAGGTTCGATGGACCTGAATCTGGTGCCCCACAAATGGAAGGACGCAGACAAAATGGCGGTCCGATGGGTGGTAGG----------------------------------------------------------------------------------- 293  
2-2432    CTAGGCAAGATGGTGGACCAATGGGTGGAAGGAGGTTCGATGGACCTGAATCTGGTGCCCCACAAATGGAAGGACGCAGACAAAATGGCGGTCCGATGGGTGGTAGG----------------------------------------------------------------------------------- 290  
2-2437    CTAGGCAAGATGGTGGACCAATGGGTGGAATGAGGTTCGATGGACCTGAATCTGGTGCCCCACAAATGGATGGACGCAGACAAAATGGCGGTCCGATGGGTGGTAGG----------------------------------------------------------------------------------- 290  
2-2439    CTAGGCAAGATGGTGGACCAATGGGTGGAAGGAGGTTCGATGGACCTGAATCTGGTGCCCCACAAATGGAAGGACGCAGACAAAATGGCGGTCCGATGGGTGGTAGG----------------------------------------------------------------------------------- 293  
2-2442    CTAGGCAAGATGGTGGACCAATGGGTGGAAGGAGGTTCGATGGACCTGAATCTGGTGCCCCACAAATGGAAGGACGCAGACAAAATGGCGGTCCGATGGGTGGTAGG----------------------------------------------------------------------------------- 293  
2-2443    CTAGGCAAGATGGTGGACCAATGGGTGGAAGGAGGTTCGATGGACCTGAATCTGGTGCCCCACAAATGGAAGGACGCAGACAAAATGGCGGTCCGATGGGTGGTAGG----------------------------------------------------------------------------------- 293  
2-2445    CTAGGCAAGATGGTGGACCAATGGGTGGAAGGAGGTTCGATGGACCTGAATCTGGTGCCCCACAAATGGAAGGACGCAGACAAAATGGCGGTCCGATGGGTGGTAGG----------------------------------------------------------------------------------- 293  
2-2435    CTAGGCAAGATGGTGGACCAATGGGTGGAATGAGGTTCGATGGACCTGAATCTGGTGCCCCACAAATGGATGGACGCAGACAAAATGGCGGTCCGATGGGTGGTAGG----------------------------------------------------------------------------------- 293  
2-2447    CTAGGCAAGATGGTGGACCAATGGGTGGAATGAGGTTCGATGGACCTGAATCTGGTGCCCCACAAATGGATGGACGCAGACAAAATGGCGGTCCGATGGGTGGTAGG----------------------------------------------------------------------------------- 293  
2-2401    CTAGGCAAGATGGTGGACCAATGGGTGGAATGAGGTTCGATGGACCTGAATCTGGTGCCCCACAAATGGATGGACGCAGACAAAATGGCGGTCCGATGGGTGGTAGG----------------------------------------------------------------------------------- 293  
9-1504    CTAGGCAAGATGGTGGACCAATGGGTGGAGGGAGGTTCGATGGACCTGAATCTGGTGCCCCACAAATGGAAGGACGCAGACAAAATGGCGGTCCGATGGGTGGTAGG----------------------------------------------------------------------------------- 293  
9-1505    CTAGGCAAGATGGTGGACCAATGGGTGGAGGGAGGTTCGATGGACCTGAATCTGGTGCCCCACAAATGGAAGGACGCAGACAAAATGGCGGTCCGATGGGTGGTAGG----------------------------------------------------------------------------------- 293  
9-1506    CTAGGCAAGATGGTGGACCAATGGGTGGAGGGAGGTTCGATGGACCTGAATCTGGTGCCCCACAAATGGAAGGACGCAGACAAAATGGCGGTCCGATGGGTGGTAGG----------------------------------------------------------------------------------- 293  
9-1507    CTAGGCAAGATGGTGGACCAATGGGTGGAGGGAGGTTCGATGGACCTGAATCTGGTGCCCCACAAATGGAAGGACGCAGACAAAATGGCGGTCCGATGGGTGGTAGG----------------------------------------------------------------------------------- 293  
9-1509    CTAGGCAAGATGGTGGACCAATGGGTGGGGGGAGGTTCGATGGACCTGAATCTGGTGCCCCACAAATGGAAGGACGCAGACAAAATGGCGGTCCGATGGGTGGTAGG----------------------------------------------------------------------------------- 293  
9-1512    CTAGGCAAGATGGTGGACCAATGGGTGGAGGGAGGTTCGATGGACCTGAATCTGGTGCCCCACAAATGGAAGGACGCAGACAAAATGGCGGTCCGATGGGTGGCAGG----------------------------------------------------------------------------------- 293  
9-1514    CTAGGCAAGATGGTGGACCAATGGGTGGAGGGAGGTTCGATGGACCTGAATCTGGTGCCCCACAAATGGAAGGACGCAGACAAAATGGCGGTCCGATGGGTGGTAGG----------------------------------------------------------------------------------- 293  
9-1515    CTAGGCAAGATGGTGGACCAATGGGTGGAGGGAGGTTCGATGGACCTGAATCTGGTGCCCCACAAATGGAAGGACGCAGACAAAATGGCGGTCCGATGGGTGGTAGG----------------------------------------------------------------------------------- 293  
9-1516    CTAGGCAAGATGGTGGACCAATGGGTGGAGGGAGGTTCGATGGACCTGAATCTGGTGCCCCACAAATGGAAGGACGCAGACAAAATGGCGGTCCGATGGGTGGTAGG----------------------------------------------------------------------------------- 293  
9-1518    CTAGGCAAGATGGTGGACCAATGGGTGGAGGGAGGTTCGATGGACCTGAATCTGGTGCCCCACAAATGGAAGGACGCAGACAAAATGGCGGTCCGATGGGTGGTAGG----------------------------------------------------------------------------------- 293  
9-1519    CTAGGCAAGATGGTGGACCAATGGGTGGAGGGAGGTTCGATGGACCTGAATCTGGTGCCCCACAAATGGAAGGACGCAGACAAAATGGCGGTCCGATGGGTGGTAGG----------------------------------------------------------------------------------- 293  
9-1520    CTAGGCAAGATGGTGGACCAATGGGTGGAGGGAGGTTCGATGGACCTGAATCTGGTGCCCCACAAATGGAAGGACGCAGACAAAATGGCGGTCCGATGGGTGGTAGG----------------------------------------------------------------------------------- 293  
9-1521    CTAGGCAAGATGGTGGACCAATGGGTGGAGGGAGGTTCGATGGACCTGAATCTGGTGCCCCACAAATGGAAGGACGCAGACAAAATGGCGGTCCGATGGGTGGTAGG----------------------------------------------------------------------------------- 293  
9-1523    CTAGGCAAGATGGTGGACCAATGGGTGGAGGGAGGTTCGATGGACCTGAATCTGGTGCCCCACAAATGGAAGGACGCAGACAAAATGGCGGTCCGATGGGTGGTAGG----------------------------------------------------------------------------------- 293  
9-1524    CTAGGCAAGATGGTGGACCAATGGGTGGAGGGAGGTTCGATGGACCTGAATCTGGTGCCCCACAAATGGAAGGACGCAGACAAAATGGCGGTCCGATGGGTGGTAGG----------------------------------------------------------------------------------- 291  
9-1526    CTAGGCAAGATGGTGGACCAATGGGTGGAGGGAGGTTCGATGGACCTGAATCTGGTGCCCCACAAATGGAAGGACGCAGACAAAATGGCGGTCCGATGGGTGGTAGG----------------------------------------------------------------------------------- 293  
9-1527    CTAGGCAAGATGGTGGACCAATGGGTGGAGGGAGGTTCGATGGACCTGAATCTGGTGCCCCACAAATGGAAGGACGCAGACAAAATGGCGGTCCGATGGGTGGTAGG----------------------------------------------------------------------------------- 293  
9-1530    CTAGGCAAGATGGTGGACCAATGGGTGGAGGGAGGTTCGATGGACCTGAATCTGGTGCCCCACAAATGGAAGGACGCAGACAAAATGGCGGTCCGATGGGTGGTAGG----------------------------------------------------------------------------------- 293  
9-1531    CTAGGCAAGATGGTGGACCAATGGGTGGAGGGAGGTTCGATGGACCTGAATCTGGTGCCCCACAAATGGAAGGACGCAGACAAAATGGCGGTCCGATGGGTGGTAGG----------------------------------------------------------------------------------- 293  
9-1533    CTAGGCAAGATGGTGGACCAATGGGTGGAGGGAGGTTCGATGGACCTGAATCTGGTGCCCCACAAATGGAAGGACGCAGACAAAATGGCGGTCCGATGGGTGGTAGG----------------------------------------------------------------------------------- 293  
9-1537    CTAGGCAAGATGGTGGACCAATGGGTGGAGGGAGGTTCGATGGACCTGAATCTGGTGCCCCACAAATGGAAGGACGCAGACAAAATGGCGGTCCGATGGGTGGTAGG----------------------------------------------------------------------------------- 293  
9-1538    CTAGGCAAGATGGTGGACCAATGGGTGGAGGGAGGTTCGATGGACCTGAATCTGGTGCCCCACAAATGGAAGGACGCAGACAAAATGGCGGTCCGATGGGTGGTAGG----------------------------------------------------------------------------------- 293  
9-1539    CTAGGCAAGATGGTGGACCAATGGGTGGAGGGAGGTTCGATGGACCTGAATCTGGTGCCCCACAAATGGAAGGACGCAGACAAAATGGCGGTCCGATGGGTGGTAGG----------------------------------------------------------------------------------- 293  
9-1540    CTAGGCAAGATGGTGGACCAATGGGTGGAGGGAGGTTCGATGGACCTGAATCTGGTGCCCCACAAATGGAAGGACGCAGACAAAATGGCGGTCCGATGGGTGGTAGG----------------------------------------------------------------------------------- 293  
9-1543    CTAGGCAAGATGGTGGACCAATGGGTGGAGGGAGGTTCGATGGACCTGAATCTGGTGCCCCACAAATGGAAGGACGCAGACAAAATGGCGGTCCGATGGGTGGTAGG----------------------------------------------------------------------------------- 293  
9-1544    CTAGGCAAGATGGTGGACCAATGGGTGGAGGGAGGTTCGATGGACCTGAATCTGGTGCCCCACAAATGGAAGGACGCAGACAAAATGGCGGTCCGATGGGTGGTAGG----------------------------------------------------------------------------------- 293  
9-1545    CTAGGCAAGATGGTGGACCAATGGGTGGAGGGAGGTTCGATGGACCTGAATCTGGTGCCCCACAAATGGAAGGACGCAGACAAAATGGCGGTCCGATGGGTGGTAGG----------------------------------------------------------------------------------- 293  
9-1546    CTAGGCAAGATGGTGGACCAATGGGTGGAGGGAGGTTCGATGGACCTGAATCTGGTGCCCCACAAATGGAAGGACGCAGACAAAATGGCGGTCCGATGGGTGGTAGG----------------------------------------------------------------------------------- 293  
9-1547    CTAGGCAAGATGGTGGACCAATGGGTGGAGGGAGGTTCGATGGACCTGAATCTGGTGCCCCACAAATGGAAGGACGCAGACAAAATGGCGGTCCGATGGGTGGTAGG----------------------------------------------------------------------------------- 293  
9-1548    CTAGGCAAGATGGTGGACCAATGGGTGGAGGGAGGTTCGATGGACCTGAATCTGGTGCCCCACAAATGGAAGGACGCAGACAAAATGGCGGTCCGATGGGTGGTAGG----------------------------------------------------------------------------------- 293  
9-1513    CGAGGCAAGATGGTGGACCAATGGGTGGAAGAAGGTTCGATGGACCTGACTCTGGTGCCCCACAAATGGATGGACGGAGACAAGATGGTGGACCAATGGGTGGAAGG---------------------------------------------------------------------------AGGTTCGA 298  
9-1541    CGAGGCAAGATGGTGGACCAATGGGTGGAAGAAGGTTCGATGGACCTGACTCTGGTGCCCCACAAATGGATGGACGGAGACAAGATGGTGGACCAATGGGTGGAAGG---------------------------------------------------------------------------AGGTTCGA 298  
9-1542    CGAGGCAAGATGGTGGACCAATGGGTGGAAGAAGGTTCGATGGACCTGACTCTGGTGCCCCACAAATGGATGGACGGAGACAAGATGGTGGACCAATGGGTGGAAGG---------------------------------------------------------------------------AGGTTCGA 298  
9-1525    CTAGGCAAGATGGCGGTCCGATGGGTGGTAGGAGATTCGACGGACCTAGATTTGGTGCCCCGCAGATGGGTGGACCTAGGCAAAATGGTGGACCAATGGGTGGCAGAAGGTTCGATGGACCTGGATTTGGTGCCCCGCCGATGGGTGGACCAAGGCAAGATGGTGGACCAATGGGTGGAAGAAGGTTCGA 376  
9-1501    CTAGGCAAGATGGTGGACCAATGGGTGGAAGGAGGTTCGATGGACCTGAATCTGGTGCCCCACAAATGGAAGGACGCAGACAAAATGGCGGTCCGATGGGTGGTAGG----------------------------------------------------------------------------------- 292  
9-1502    CTAGGCAAGATGGTGGACCAATGGGTGGAAGGAGGTTCGATGGACCTGAATCTGGTGCCCCACAAATGGAAGGACGCAGACAAAATGGCGGTCCGATGGGTGGTAGG----------------------------------------------------------------------------------- 293  
9-1508    CTAGGCAAGATGGTGGACCAATGGGTGGAAGGAGGTTCGATGGACCTGAATCTGGTGCCCCACAAATGGAAGGACGCAGACAAAATGGCGGTCCGATGGGTGGTAGG----------------------------------------------------------------------------------- 293  
9-1511    CTAGGCAAGATGGTGGACCAATGGGTGGAAGGAGGTTCGATGGACCTGAATCTGGTGCCCCACAAATGGAAGGACGCAGACAAAATGGCGGTCCGATGGGTGGTAGG----------------------------------------------------------------------------------- 293  
9-1517    CTAGGCAAGATGGTGGACCAATGGGTGGAAGGAGGTTCGATGGACCTGAATCTGGTGCCCCACAAATGGAAGGACGCAGACAAAATGGCGGTCCGATGGGTGGTAGG----------------------------------------------------------------------------------- 293  
9-1528    CTAGGCAAGATGGTGGACCAATGGGTGGAAGGAGGTTCGATGGACCTGAATCTGGTGCCCCACAAATGGAAGGACGCAGACAAAATGGCGGTCCGATGGGTGGTAGG----------------------------------------------------------------------------------- 293  
9-1529    CTAGGCAAGATGGTGGACCAATGGGTGGAAGGAGGTTCGATGGACCTGAATCTGGTGCCCCACAAATGGAAGGACGCAGACAAAATGGCGGTCCGATGGGTGGTAGG----------------------------------------------------------------------------------- 293  
9-1534    CTAGGCAAGATGGTGGACCAATGGGTGGAAGGAGGTTCGATGGACCTGAATCTGGTGCCCCACAAATGGAAGGACGCAGACAAAATAGCGGTCCGATGGGTGGTAGG----------------------------------------------------------------------------------- 293  
9-1535    CTAGGCAAGATGGTGGACCAATGGGTGGAAGGAGGTTCGATGGACCTGAATCTGGTGCCCCACAAATGGAAGGACGCAGACAAAATGGCGGTCCGATGGGTGGTAGG----------------------------------------------------------------------------------- 293  
9-2448    CTAGGCAAGATGGTGGACCAATGGGTGGAGGGAGGTTCGATGGACCTGAATCTGGTGCCCCACAAATGGAAGGACGCAGACAAAATGGCGGTCCGATGGGTGGTAGG----------------------------------------------------------------------------------- 293  
9-2401    CTAGGCAAGATGGTGGACCAATGGGTGGAGGGAGGTTCGATGGACCTGAATCTGGTGCCCCACAAATGGAAGGACGCAGACAAAATGGCGGTCCGATGGGTGGTAGG----------------------------------------------------------------------------------- 293  
9-2402    CTAGGCAAGATGGTGGACCAATGGGTGGAGGGAGGTTCGATGGACCTGAATCTGGTGCCCCACAAATGGAAGGACGCAGACAAAATGGCGGTCCGATGGGTGGTAGG----------------------------------------------------------------------------------- 293  
9-2404    CTAGGCAAGATGGTGGACCAATGGGTGGAGGGAGGTTCGATGGACCTGAATCTGGTGCCCCACAAATGGAAGGACGCAGACAAAATGGCGGTCCGATGGGTGGTAGG----------------------------------------------------------------------------------- 293  
9-2406    CTAGGCAAGATGGTGGACCAATGGGTGGAGGGAGGTTCGATGGACCTGAATCTGGTGCCCCACAAATGGAAGGACGCAGACAAAATGGCGGTCCGATGGGTGGTAGG----------------------------------------------------------------------------------- 293  
9-2408    CTAGGCAAGATGGTGGACCAATGGGTGGAGGGAGGTTCGATGGACCTGAATCTGGTGCCCCACAAATGGAAGGACGCAGACAAAATGGCGGTCCGATGGGTGGTAGG----------------------------------------------------------------------------------- 293  
9-2409    CTAGGCAAGATGGTGGACCAATGGGTGGAGGGAGGTTCGATGGACCTGAATCTGGTGCCCCACAAATGGAAGGACGCAGACAAAATGGCGGTCCGATGGGTGGTAGG----------------------------------------------------------------------------------- 293  
9-2411    CTAGGCAAGATGGTGGACCAATGGGTGGAGGGAGGTTCGATGGACCTGAATCTGGTGCCCCACAAATGGAAGGACGCAGACAAAATGGCGGTCCGATGGGTGGTAGG----------------------------------------------------------------------------------- 293  
9-2413    CTAGGCAAGATGGTGGACCAATGGGTGGAGGGAGGTTCGATGGACCTGAATCTGGTGCCCCACAAATGGAAGGACGCAGACAAAATGGCGGTCCGATGGGTGGTAGG----------------------------------------------------------------------------------- 293  
9-2414    CTAGGCAAGATGGTGGACCAATGGGTGGAGGGAGGTTCGATGGACCTGAATCTGGTGCCCCACAAATGGAAGGACGCAGACAAAATGGCGGTCCGATGGGTGGTAGG----------------------------------------------------------------------------------- 293  
9-2417    CTAGGCAAGATGGTGGACCAATGGGTGGAGGGAGGTTCGATGGACCTGAATCTGGTGCCCCACAAATGGAAGGACGCAGACAAAATGGCGGTCCGATGGGTGGTAGG----------------------------------------------------------------------------------- 293  
9-2418    CTAGGCAAGATGGTGGACCAATGGGTGGAGGGAGGTTCGATGGACCTGAATCTGGTGCCCCACAAATGGAAGGACGCAGACAAAATGGCGGTCCGATGGGTGGTAGG----------------------------------------------------------------------------------- 293  
9-2419    CTAGGCAAGATGGTGGACCAATGGGTGGAGGGAGGTTCGATGGACCTGAATCTGGTGCCCCACAAATGGAAGGACGCAGACAAAATGGCGGTCCGATGGGTGGTAGG----------------------------------------------------------------------------------- 293  
9-2420    CTAGGCAAGATGGTGGACCAATGGGTGGAGGGAGGTTCGATGGACCTGAATCTGGTGCCCCACAAATGGAAGGACGCAGACAAAATGGCGGTCCGATGGGTGGTAGG----------------------------------------------------------------------------------- 293  
9-2421    CTAGGCAAGATGGTGGACCAATGGGTGGAGGGAGGTTCGATGGACCTGAATCTGGTGCCCCACAAATGGAAGGACGCAGACAAAATGGCGGTCCGATGGGTGGTAGG----------------------------------------------------------------------------------- 293  
9-2424    CTAGGCAAGATGGTGGACCAATGGGTGGAGGGAGGTTCGATGGACCTGAATCTGGTGCCCCACAAATGGAAGGACGCAGACAAAATGGCGGTCCGATGGGTGGTAGG----------------------------------------------------------------------------------- 293  
9-2427    CTAGGCAAGATGGTGGACCAATGGGTGGAGGGAGGTTCGATGGACCTGAATCTGGTGCCCCACAAATGGAAGGACGCAGACAAAATGGCGGTCCGATGGGTGGTAGG----------------------------------------------------------------------------------- 293  
9-2429    CTAGGCAAGATGGTGGACCAATGGGTGGAGGGAGGTTCGATGGACCTGAATCTGGTGCCCCACAAATGGAAGGACGCAGACAAAATGGCGGTCCGATGGGTGGTAGG----------------------------------------------------------------------------------- 293  
9-2430    CTAGGCAAGATGGTGGACCAATGGGTGGAGGGAGGTTCGATGGACCTGAATCTGGTGCCCCACAAATGGAAGGACGCAGACAAAATGGCGGTCCGATGGGTGGTAGG----------------------------------------------------------------------------------- 293  
9-2433    CTAGGCAAGATGGTGGACCAATGGGTGGAGGGAGGTTCGATGGACCTGAATCTGGTGCCCCACAAATGGAAGGACGCAGACAAAATGGCGGTCCGATGGGTGGTAGG----------------------------------------------------------------------------------- 293  
9-2435    CTAGGCAAGATGGTGGACCAATGGGTGGAGGGAGGTTCGATGGACCTGAATCTGGTGCCCCACAAATGGAAGGACGCAGACAAAATGGCGGTCCGATGGGTGGTAGG----------------------------------------------------------------------------------- 293  
9-2438    CTAGGCAAGATGGTGGACCAATGGGTGGAGGGAGGTTCGATGGACCTGAATCTGGTGCCCCACAAATGGAAGGACGCAGACAAAATGGCGGTCCGATGGGTGGTAGG----------------------------------------------------------------------------------- 293  
9-2439    CTAGGCAAGATGGTGGACCAATGGGTGGAGGGAGGTTCGATGGACCTGAATCTGGTGCCCCACAAATGGAAGGACGCAGACAAAATGGCGGTCCGATGGGTGGTAGG----------------------------------------------------------------------------------- 293  
9-2440    CTAGGCAAGATGGTGGACCAATGGGTGGAGGGAGGTTCGATGGACCTGAATCTGGTGCCCCACAAATGGAAGGACGCAGACAAAATGGCGGTCCGATGGGTGGTAGG----------------------------------------------------------------------------------- 293  
9-2441    CTAGGCAAGATGGTGGACCAATGGGTGGAGGGAGGTTCGATGGACCTGAATCTGGTGCCCCACAAATGGAAGGACGCAGACAAAATGGCGGTCCGATGGGTGGTAGG----------------------------------------------------------------------------------- 293  
9-2442    CTAGGCAAGATGGTGGACCAATGGGTGGAGGGAGGTTCGATGGACCTGAATCTGGTGCCCCACAAATGGAAGGACGCAGACAAAATGGCGGTCCGATGGGTGGTAGG----------------------------------------------------------------------------------- 293  
9-2443    CTAGGCAAGATGGTGGACCAATGGGTGGAGGGAGGTTCGATGGACCTGAATCTGGTGCCCCACAAATGGAAGGACGCAGACAAAATGGCGGTCCGATGGGTGGTAGG----------------------------------------------------------------------------------- 293  
9-2445    CTAGGCAAGATGGTGGACCAATGGGTGGAGGGAGGTTCGATGGACCTGAATCTGGTGCCCCACAAATGGAAGGACGCAGACAAAATGGCGGTCCGATGGGTGGTAGG----------------------------------------------------------------------------------- 293  
9-2446    CTAGGCAAGATGGTGGACCAATGGGTGGAGGGAGGTTCGATGGACCTGAATCTGGTGCCCCACAAATGGAAGGACGCAGACAAAATGGCGGTCCGATGGGTGGTAGG----------------------------------------------------------------------------------- 293  
9-2416    CGAGGCAAGATGGTGGACCAATGGGTGGAAGAAGGTTCGATGGACCTGACTCTGGTGCCCCACAAATGGATGGACGGAGACAAGATGGTGGACCAATGGGTGGAAGG---------------------------------------------------------------------------AGGTTCGA 298  
9-2423    CGAGGCAAGATGGTGGACCAATGGGTGGAAGAAGGTTCGATGGACCTGACTCTGGTGCCCCACAAATGGATGGACGGAGACAAGATGGTGGACCAATGGGTGGAAGG---------------------------------------------------------------------------AGGTTCGA 298  
9-2403    CTAGGCAAGATGGTGGACCAATGGGTGGAAGGAGGTTCGATGGACCTGAATCTGGTGCCCCACAAATGGAAGGACGCAGACAAAATGGCGGTCCGATGGGTGGTAGG----------------------------------------------------------------------------------- 293  
9-2415    CTAGGCAAGATGGTGGACCAATGGGTGGAAGGAGGTTCGATGGACCTGAATCTGGTGCCCCACAAATGGAAGGACGCAGACAAAATGGCGGTCCGATGGGTGGTAGG----------------------------------------------------------------------------------- 293  
9-2422    CTAGGCAAGATGGTGGACAAATGGGTGGAAGGAGGTTCGATGGACCTGAATCTGGTGCCCCACAAATGGAAGGACGCAGACAAAATGGCGGTCCGATGGGTGGTAGG----------------------------------------------------------------------------------- 291  
9-2428    CTAGGCAAGATGGTGGACCAATGGGTGGAAGGAGGTTCGATGGACCTGAATCTGGTGCCCCACAAATGGAAGGACGCAGACAAAATGGCGGTCCGATGGGTGGTAGG----------------------------------------------------------------------------------- 293  
9-2431    CTAGGCAAGATGGTGGACCAATGGGTGGAAGGAGGTTCGATGGACCTGAATCTGGTGCCCCACAAATGGAAGGACGCAGACAAAATGGCGGTCCGATGGGTGGTAGG----------------------------------------------------------------------------------- 293  
9-2432    CTAGGCAAGATGGTGGACCAATGGGTGGAAGGAGGTTCGATGGACCTGAATCTGGTGCCCCACAAATGGAAGGACGCAGACAAAATGGCGGTCCGATGGGTGGTAGG----------------------------------------------------------------------------------- 293  
9-2434    CTAGGCAAGATGGTGGACCAATGGGTGGAGGGAGGTTCGATGGACCTGAATCTGGTGCCCCACAAATGGAAGGACGCAGACAAAATGGCGGTCCGATGGGTGGTAGG----------------------------------------------------------------------------------- 293  
9-2437    CTAGGCAAGATGGTGGACCAATGGGTGGAAGGAGGTTCGATGGACCTGAATCTGGTGCCCCACAAATGGAAGGACGCAGACAAAATGGCGGTCCGATGGGTGGTAGG----------------------------------------------------------------------------------- 293  
9-2444    CTAGGCAAGATGGTGGACCAATGGGTGGAAGGAGGTTCGATGGACCTGAATCTGGTGCCCCACAAATGGAAGGACGCAGACAAAATGGCGGTCCGATGGGTGGTAGG----------------------------------------------------------------------------------- 293  
4-1504    CGAGGCAAGATGGTGGACCAATGGGTGGAAGAAGGTTCGATGGACCTGACTCTGGTGCCCCACAAATGGATGGACGGAGACAAGATGGTGGACCAATGGGTGGAAGG---------------------------------------------------------------------------AGGTTCGA 298  
4-1522    CTAGGCAAGATGGTGGACCAATGGGTGGAGGGAGGTTCGATGGACCTGAATCTGGTGCCCCACAAATGGAAGGACGCAGACAAAATGGCGGTCCGATGGGTGGTAGG----------------------------------------------------------------------------------- 293  
4-1507    CTAGGCAAGATGGTGGACCAATGGGTGGAAGGAGGTTCGATGGACCTGAATCTGGTGCCCCACAAATGGAAGGACGTAGACAAAATGGCGGTCCGATGGGTGGTAGG----------------------------------------------------------------------------------- 293  
4-1519    CTAGGCAAGATGGTGGACCAATGGGTGGAGGGAGGTTCGATGGACCTGAATCTGGTGCCCCACAAATGGAAGGACGCAGACAAAATGGCGGTCCGATGGGTGGTAGG----------------------------------------------------------------------------------- 293  
4-1529    CTAGGCAAGATGGTGGACCAATGGGTGGAGGGAGGTTCGATGGACCTGAATCTGGTGCCCCACAAATGGAAGGACGCAGACAAAATGGCGGTCCGATGGGTGGTAGG----------------------------------------------------------------------------------- 293  
4-1549    CTAGGCAAGATGGTGGACCAATGGGTGGAGGGAGGTTCGATGGACCTGAATCTGGTGCCCCACAAATGGAAGGACGCAGACAAAATGGCGGTCCGATGGGTGGTAGG----------------------------------------------------------------------------------- 293  
4-1510    CTAGGCAAGATGGTGGACCAATGGGTGGAGGGAGGTTCGATGGACCTGAATCTGGTGCCCCACAAATGGAAGGACGCAGACAAAATGGCGGTCCGATGGGTGGTAGG----------------------------------------------------------------------------------- 295  
4-1539    CTAGGCAAGATGGTGGACCAATGGGTGGAGGGAGGTTCGATGGACCTGAATCTGGTGCCCCACAAATGGAAGGACGCAGACAAAATGGCGGTCCGATGGGTGGTAGG----------------------------------------------------------------------------------- 293  
4-2424    CTAGGCAAGATGATGGACCAATGGGTGGAAGGAGGTTCGATGGACCTGAATCTGGTGCCCCACAAATGGATGGACGCAGACAAAATGGCGGTCCGATGGGTGGTAGG----------------------------------------------------------------------------------- 265  
4-2401    CTAGGCAAGATGGTGGACCAATGGGTGGAAGGAGGTTCGATGGACCTGAATCTGGTGCCCCACAAATGGAAGGACGCAGACAAAATGGCGGTCCGATGGGTGGTAGG----------------------------------------------------------------------------------- 291  
4-2405    CTAGGCAAGATGGTGGACAAATGGGTGGAAGGAGGTTCGATGGACCTGAATCTGGTGCCCCACAAATGGAAGGACGCAGACAAAATGGCGGTCCGATGGGTGGTAGG----------------------------------------------------------------------------------- 291  
4-2407    CTAGGCAAGATGGTGGACCAATGGGTGGAAGGAGGTTCGATGGACCTGAATCTGGTGCCCCACAAATGGAAGGACGCAGACAAAATGGCGGTCCGATGGGTGGTAGG----------------------------------------------------------------------------------- 291  
4-2408    CTAGGCAAGATGGTGGACCAATGGGTGGAAGGAGGTTCGATGGACCTGAATCTGGTGCCCCACAAATGGAAGGACGCAGACAAAATGGCGGTCCGATGGGTGGTAGG----------------------------------------------------------------------------------- 291  
4-2411    CTAGGCAAGATGGTGGACAAATGGGTGGAAGGAGGTTCGATGGACCTGAATCTGGTGCCCCACAAATGGAAGGACGCAGACAAAATGGCGGTCCGATGGGTGGTAGG----------------------------------------------------------------------------------- 290  
4-2417    CTAGGCAAGATGGTGGACCAATGGGTGGAAGGAGGTTCGATGGACCTGAATCTGGTGCCCCACAAATGGAAGGACGCAGACAAAATGGCGGTCCGATGGGTGGTAGG----------------------------------------------------------------------------------- 291  
4-2418    CTAGGCAAGATGGTGGACCAATGGGTGGAAGGAGGTTCGATGGACCTGAATCTGGTGCCCCACAAATGGAAGGACGCAGACAAAATGGCGGTCCGATGGGTGGTAGG----------------------------------------------------------------------------------- 291  
4-2419    CTAGGCAAGATGGTGGACCAATGGGTGGAAGGAGGTTCGATGGACCTGAATCTGGTGCCCCACAAATGGAAGGACGCAGACAAAATGGCGGTCCGATGGGTGGTAGG----------------------------------------------------------------------------------- 289  
4-2421    CTAGGCAAGATGGTGGACCAATGGGTGGAAGGAGGTTCGATGGACCTGAATCTGGTGCCCCACAAATGGAAGGACGCAGACAAAATGGCGGTCCGATGGGTGGTAGG----------------------------------------------------------------------------------- 291  
4-2422    CTAGGCAAGATGGTGGACCAATGGGTGGAAGGAGGTTCGATGGACCTGAATCTGGTGCCCCACAAATGGAAGGACGCAGACAAAATGGCGGTCCGATGGGTGGTAGG----------------------------------------------------------------------------------- 291  
4-2426    CTAGGCAAGATGGTGGACCAATGGGTGGAAGGAGGTTCGATGGACCTGAATCTGGTGCCCCACAAATGGAAGGACGCAGACAAAATGGCGGTCCGATGGGTGGTAGG----------------------------------------------------------------------------------- 291  
4-2428    CTAGGCAAGATGGTGGACCAATGGGTGGAAGGAGGTTCGATGGACCTGAATCTGGTGCCCCACAAATGGAAGGACGCAGACAAAATGGCGGTCCGATGGGTGGTAGG----------------------------------------------------------------------------------- 291  
4-2429    CTAGGCAAGATGGTGGACCAATGGGTGGAAGGAGGTTCGATGGACCTGAATCTGGTGCCCCACAAATGGAAGGACGCAGACAAAATGGCGGTCCGATGGGTGGTAGG----------------------------------------------------------------------------------- 291  
4-2435    CTAGGCAAGATGGTGGACCAATGGGTGGAAGGAGGTTCGATGGACCTGGATTTGGTGCCCCACATATGGATGGACGCAGACAAAATGGCGGTCCGATGGGTGGTAGG----------------------------------------------------------------------------------- 291  
4-2437    CTAGGCAAGATGGTGGACCAATGGGTGGAAGGAGGTTCGATGGACCTGAATCTGGTGCCCCACAAATGGAAGGACGCAGACAAAATGGCGGTCCGATGGGTGGTAGG----------------------------------------------------------------------------------- 293  
4-2444    CTAGGCAAGATGGTGGACCAATGGGTGGAAGGAGGTTCGATGGACCTGAATCTGGTGCCCCACAAATGGAAGGACGCAGACAAAATGGCGGTCCGATGGGTGGTAGG----------------------------------------------------------------------------------- 291  
4-2445    CTAGGCAAGATGGTGGACCAATGGGTGGAAGGAGGTTCGATGGACCTGAATCTGGTGCCCCACAAATGGAAGGACGCAGACAAAATGGCGTTCCGATGGGTGGTAGG----------------------------------------------------------------------------------- 291  
4-2447    CTAGGCAAGATGGTGGACCAATGGGTGGAAGGAGGTTCGATGGACCTGAATCTGGTGCCCCACAAATGGAAGGACGCAGACAAAATGGCGGTCCGATGGGTGGTAGG----------------------------------------------------------------------------------- 291  
4-2448    CTAGGCAAGATGGTGGACCAATGGGTGGAAGGAGGTTCGATGGACCTGAATCTGGTGCCCCACAAATGGAAGGACGCAGACAAAATGGCGGTCCGATGGGTGGTAGG----------------------------------------------------------------------------------- 283  
4-2404    CGAGGCAAGATGGTGGACCAATGGGTGGAAGAAGGTTCGATGGACCTGACTCTGGTGCCCCACAAATGGATGGACGGAGACAAGATGGTGGACCAATGGGTGGAAGG---------------------------------------------------------------------------AGGTTCGA 297  
4-2413    CGAGGCAAGATGGTGGACCAATGGGTGGAAGAAGGTTCGATGGACCTGACTCTGGTGCCCCACAAATGGATGGACGGAGACAAGATGGTGGACCAATGGGTGGAAGG---------------------------------------------------------------------------AGGTTCGA 298  
4-2450    CGAGGCAAGATGGTGGACCAATGGGTGGAAGAAGGTTCGATGGACCTGACTCTGGTGCCCCACAAATGGATGGACGGAGACAAGATGGTGGACCAATGGGTGGAAGG---------------------------------------------------------------------------AGGTTCGA 298  
4-2420    CGAGGCAAGATGGTGGACCAATGGGTGGAAGAAGGTTCGATGGACCTGACTCTGGTTCCCCACAAATGGATGGACGGAGACAAGATGGTGGACCAATGGGTGGAAGG---------------------------------------------------------------------------AGGTTCGA 299  
4-2438    CGAGGCAAGATGGTGGACCAATGGGTGGAAGAAGGTTCGATGGACCTGACTCTGGTGCCCCACAAATGGATGGACGGAGACAAGATGGTGGACCAATGGGTGGAAGG---------------------------------------------------------------------------AGGTTCGA 299  
4-2430    CTAGGCAAGATGGTGGACCAATGGGTGGAAGAAGGTTCGATGGACCTGACTCTGGTTCCCCACAAATGGATGGACGGAGACAAGATGGTGGACCAATGGGTGGAAGG---------------------------------------------------------------------------AGGTTCGA 299  
4-2439    CTAGGCAAGATGGTGGACCAATGGGTGGAAGGAGGTTCGATGGACCTGAATCTGGTGCCCCACAAATGGAAGGACGCAGACAAAATGGCGGTCCGATGGGTGGTAGG----------------------------------------------------------------------------------- 291  
4-2433    CTAGGCAAGATGGTGGACCAATGGGTGGAAGGAGGTTCGATGGACCTGGATTTGGTGCCCCACATATGGATGGACGCAGACAAAATGGCGGTCCGATGGGTGGTAGG----------------------------------------------------------------------------------- 291  
4-2441    CTAGGCAAGATGGTGGACCAATGGGTGGAAGGAGGTTCGATGGACCTGGATTTGGTGCCCCACATATGGATGGACGCAGACAAAATGGCGGTCCGATGGGTGGTAGG----------------------------------------------------------------------------------- 291  
4-2406    CTAGGCAAGATGGCGGTCCGATGGGTGGTAGGAGATTCGACGGACCTGGATTTGGTGCCCCGCAGATGGGTGGACCTAGGCAAAATGGTGGACCAATGGGTGGAAGAAGGTTCGATGGACCTAGATTTGGTGCCCCACCGATGGGTGGACCAAGGCAAGATGGTGGACCAATGGGTGGTAGG-------- 366  
4-2425    CTAGGCAAGATGGTGGACCAATGGGTGGAATGAGGTTCGATGGACCTGAATCTGGTGCCCCACAAATGGATGGACGCAGACAAAATGGCGGTCCGATGGGTGGTAGG----------------------------------------------------------------------------------- 290  
4-2432    CTAGGCAAGATGGTGGACCAATGGGTGGAATGAGGTTCGATGGACCTGAATCTGGTGCCCCACAAATGGATGGACGCAGACAAAATGGCGGTCCGATGGGTGGTAGG----------------------------------------------------------------------------------- 290  
4-2440    CTAGGCAAGATGGTGGACCAATGGGTGGAATGAGGTTCGATGGACCTGAATCTGGTGCCCCACAAATGGATGGACGCAGACAAAATGGCGGTCCGATGGGTGGTAGG----------------------------------------------------------------------------------- 290  
4-2402    ---------------------------------------------------------------------------------------------------------------------------------------------------------------------------------------------- 114  
4-2442    ---------------------------------------------------------------------------------------------------------------------------------------------------------------------------------------------- 17   
4-2443    CTAGGCAAGATGGTGGACCAATGGGTTGAATGAGGTTCGATGGACCTGAATCTGGTGCCCCACAAATGGATGGACGCAGACAAAATGGCGGTCCGATGGGTGGTAGG----------------------------------------------------------------------------------- 290  
4-2416    CTAGGCAAGATGATGGACCAATGGGTGGAAGG---------------------------------------------------------------------------AGGTTCGATGGACCTGAATCTGGTGCCCCACCGTCATCACAACAAGACCGGAGATGGAGATCAGGACAGACCAATGTTTGA-- 296  
4-2446    CTAGGCAAGATGGTGGACCAATGGGTGGAGGGAGGTTCGATGGACCTGAATCTGGTGCCCCACAAATGGAAGGACGCAGACAAAATGGCGGTCCGATGGGTGGTAGG----------------------------------------------------------------------------------- 293  
4-2403    CTAGGCAAGATGGTGGACCAATGGGTGGAGGGAGGTTCGATGGACCTGAATCTGGTGCCCCACAAATGGAAGGACGCAGACAAAATGGCGGTCCGATGGGTGGTAGG----------------------------------------------------------------------------------- 293  
4-2423    CTAGGCAAGATGGTGGACCAATGGGTGGAGGGAGGTTCGATGGACCTGAATCTGGTGCCCCACAAATGGAAGGACGCAGACAAAATGGCGGTCCGATGGGTGGTAGG----------------------------------------------------------------------------------- 293  
4-2409    CTAGGCAAGATGGTGGACCAACGGGTGGAGGGAGGTTCGATGGACCTGAATCTGGTGCCCCACAAATGGAAGGACGCAGACAAAATGGCGGTCCGATGGGTGGTAGG----------------------------------------------------------------------------------- 293  
4-2434    CTAGGCAAGATGGTGGACCAATGGGTGGAGGGAGGTTCGATGGACCTGAATCTGGTGCCCCACAAATGGAAGGACGCAGACAAAATGGCGGTCCGATGGGTGGTAGG----------------------------------------------------------------------------------- 293  
5-1502    CAAGGCAAGATGGCGGTCCGATGGGTGGTAGG---------------------------------------------------------------------------AGATTCGACGGACATGGATTTGGTGCCCCGCCGATGGGTGGACCAAGGCAAGATGGTGGACCAATGGGTGGAAGGAGGTTCGA 299  
5-1503    CAAGGCAAGATGGCGGTCCGATGGGTGGTAGG---------------------------------------------------------------------------AGATTCGACGGACATGGATTTGGTGCCCCGCCGATGGGTGGACCAAGGCAAGATGGTGGACCAATGGGTGGAAGGAGGTTCGA 299  
5-1504    CAAGGCAAGATGGCGGTCCGATGGGTGGTAGG---------------------------------------------------------------------------AGATTCGACGGACATGGATTTGGTGCCCCGCCGATGGGTGGACCAAGGCAAGATGGTGGACCAATGGGTGGAAGGAGGTTCGA 299  
5-1505    CAAGGCAAGATGGCGGTCCGATGGGTGGTAGG---------------------------------------------------------------------------AGATTCGACGGACATGGATTTGGTGCCCCGCCGATGGGTGGACCAAGGCAAGATGGTGGACCAATGGGTGGAAGGAGGTTCGA 299  
5-1507    CAAGGCAAGATGGCGGTCCGATGGGTGGTAGG---------------------------------------------------------------------------AGATTCGACGGACATGGATTTGGTGCCCCGCCGATGGGTGGACCAAGGCAAGATGGTGGACCAATGGGTGGAAGGAGGTTCGA 300  
5-1509    CAAGGCAAGATGGCGGTCCGATGGGTGGTAGG---------------------------------------------------------------------------AGATTCGACGGACATGGATTTGGTGCCCCGCCGATGGGTGGACCAAGGCAAGATGGTGGACCAATGGGTGGAAGGAGGTTCGA 300  
5-1511    CAAGGCAAGATGGCGGTCCGATGGGTGGTAGG---------------------------------------------------------------------------AGATTCGACGGACATGGATTTGGTGCCCCGCCGATGGGTGGACCAAGGCAAGATGGTGGACCAATGGGTGGAAGGAGGTTCGA 300  
5-1513    CAAGGCAAGATGGCGGTCCGATGGGTGGTAGG---------------------------------------------------------------------------AGATTCGACGGACATGGATTTGGTGCCCCGCCGATGGGTGGACCAAGGCAAGATGGTGGACCAATGGGTGGAAGGAGGTTCGA 301  
5-1514    CAAGGCAAGATGGCGGTCCGATGGGTGGTAGG---------------------------------------------------------------------------AGATTCGACGGACATGGATTTGGTGCCCCGCCGATGGGTGGACCAAGGCAAGATGGTGGACCAATGGGTGGAAGGAGGTTCGA 300  
5-1508    CAAGGCAAGATGGCGGTCCGATGGGTGGTAGG---------------------------------------------------------------------------AGATTCGACGGACATGGATTTGGTGCCCCGCCGATGGGTGGACCAAGGCAAGATGGTGGACCAATGGGTGGAAGGAGGTTCGA 299  
5-1510    CAAGGCAAGATGGCGGTCCGATGGGTGGTAGG---------------------------------------------------------------------------AGATTCGACGGACATGGATTTGGTGCCCCGCCGATGGGTGGACCAAGGCAAGATGGTGGACCAATGGGTGGAAGGAGGTTCGA 299  
5-2401    CGAGGCAAGATGGTGGACCAATGGGTGGAAGAAGGTTCGATGGACCTGACTCTGGTGCCCCACAAATGGATGGACGGAGACAAGATGGTGGACCAATGGGTGGAAGG---------------------------------------------------------------------------AGGTTCGA 298  
5-2402    CGAGGCAAGATGGTGGACCAATGGGTGGAAGAAGGTTCGATGGACCTGACTCTGGTGCCCCACAAATGGATGGACGGAGACAAGATGGTGGACCAATGGGTGGAAGG---------------------------------------------------------------------------AGGTTCGA 298  
5-2404    CGAGGCAAGATGGTGGACCAATGGGTGGAAGAAGGTTCGATGGACCTGACTCTGGTGCCCCACAAATGGATGGACGGAGACAAGATGGTGGACCAATGGGTGGAAGG---------------------------------------------------------------------------AGGTTCGA 299  
5-2407    CGAGGCAAGATGGTGGACCAATGGGTGGAAGAAGGTTCGATGGACCTGACTCTGGTGCCCCACAAATGGATGGACGGAGACAAGATGGTGGACCAATGGGTGGAAGG---------------------------------------------------------------------------AGGTTCGA 299  
5-2409    CGAGGCAAGATGGTGGACCAATGGGTGGAAGAAGGTTCGATGGACCTGACTCTGGTGCCCCACAAATGGATGGACGGAGACAAGATGGTGGACCAATGGGTGGAAGG---------------------------------------------------------------------------AGGTTCGA 298  
5-2410    CTAGGCAAGATGGTGGACCAATGGGTGGAAGGAGGTTCGATGGACCTGAATCTGGTGCCCCACAAATGGAAGGACGCAGACAAAATGGCGGTCCGATGGGTGGTAGG----------------------------------------------------------------------------------- 296  
5-2411    CTAGGCAAGATGGTGGACCAATGGGTGGAAGGAGGTTCGATGGACCTGAATCTGGTGCCCCACAAATGGAAGGACGCAGACAAAATGGCGGTCCGATGGGTGGTAGG----------------------------------------------------------------------------------- 291  
5-2413    CTAGGCAAGATGGTGGACCAATGGGTGGAAGGAGGTTCGATGGACCTGAATCTGGTGCCCCACAAATGGAAGGACGCAGACAAAATGGCGGTCCGATGGGTGGTAGG----------------------------------------------------------------------------------- 291  
5-2414    CTAGGCAAGATGGTGGACCAATGGGTGGAAGGAGGTTCGATGGACCTGAATCTGGTGCCCCACAAATGGAAGGACGCAGACAAAATGGCGGTCCGATGGGTGGTAGG----------------------------------------------------------------------------------- 291  
5-2415    CTAGGCAAGATGGTGGACCAATGGGTGGAAGGAGGTTCGATGGACCTGAATCTGGTGCCCCACAAATGGAAGGACGCAGACAAAATGGCGGTCCGATGGGTGGTAGG----------------------------------------------------------------------------------- 291  
5-2403    CTAGGCAAGATGGTGGACCAATGGGTGGAAGGAGGTTCGATGGACCTGGATTTGGTGCCCCACATATGGATGGACGCAGACAAAATGGCGGTCCGATGGGTGGTAGG----------------------------------------------------------------------------------- 281  
5-2406    CTAGGCAAGATGGTGGACCAATGGGTGGAATGAGGTTCGATGGACCTGAATCTGGTGCCCCACAAATGGATGGACGCAGACAAAATGGCGGTCCGATGGGTGGTAGA----------------------------------------------------------------------------------- 290  
5-2412    CTAGGCAAGATGGTGGACCAATTGGTGGAATGAGGTTCGATGGACCTGAATCTGGTGCCCCACAAATGGATGGACGCAGACAAATGGCGGTCCGATGGGTGGTAGGAGATTCGACGGACCTCGATTTGGTGGCTCCAGACCAGATGGTGCTGGAGGAAGACCTTTCTTCGGCCAAGGAGGCAGGCGTGGT 374  
5-2408    AGGCAAGATGGTGGACCAATGGGTGGAAGAAGGTTCGATGGACCTGA--CTCTGGTGCCCCACAAATGGATGGACGGAGACAAGATGGTGGACCAATGGGTGGAAGG---------------------------------------------------------------------------AGGTTCGA 296  


                  390       400       410       420       430       440       450       460       470       480       490       500       510       520       530       540       550       560       570                  
          ....|....|....|....|....|....|....|....|....|....|....|....|....|....|....|....|....|....|....|....|....|....|....|....|....|....|....|....|....|....|....|....|....|....|....|....|....|....|
1-1515    -------------------------------------------------------------------AGATTCGACGGACCTCGATTTGGTGGCTCCAGACCAGATGGTGCTGGAGGGAGACCTTTCTTCGGCCAAGGAGGCAGGCGTGGTGATGGAGAAGAAGAAACTGATGCTGCCCAACAAATTGGT 416  
1-1523    -------------------------------------------------------------------AGATTCGACGGACCTCGATTTGGTGGCTCCAGACCAGATGGTGCTGGAGGGAGACCTTTCTTCGGCCAAGGAGGCAGGCGTGGTGATGGAGAAGAAGAAACTGATGCTGCCCAACAAATTGGT 416  
1-1504    -------------------------------------------------------------------AGATTCGACGGACCTCGATTTGGTGGCTCCAGACCAGATGGTGCTGGAGGGAGACCTTTCTTCGGCCAAGGAGGCAGGCGTGGTGATGGAGAAGAAGAAACTGATGCTGCCCAACAAATTGGT 416  
1-1533    -------------------------------------------------------------------AGATTCGACGGACCTCGATTTGGTGGCTCCAGACCAGATGGTGCTGGAGGGAGACCTTTCTTCGGCCAAGGAGGCAGGCGCGGTGATGGAGAAGAAGAAACTGATGCTGCCCAACAAATTGGT 391  
1-1547    -------------------------------------------------------------------AGATTCGACGGACCTCGATTTGGTGGCTCCAGACCAGATGGTGCTGGAGGGAGACCTTTCTTCGGCCAAGGAGGCAGGCGTGGTGATGGAGAAGAAGAAACTGATGCTGCCCAACAAATTGGT 416  
1-1549    -------------------------------------------------------------------AGATTCGACGGACCTCGATTTGGTGGCTCCAGACCAGATGGTGCTGGAGGGAGACCTTTCTTCGGCCAAGGAGGCAGGCGTGGTGATGGAGAAGAAGAAACTGATGCTGCCCAACAAATTGGT 418  
1-1505    -------------------------------------------------------------------AGATTCGACGGACCTCGATTTGGTGGCTCCAGACCAGATGGTGCTGGAGGGAGACCTTTCTTCGGCCAAGGAGGCAGGCGTGGTGATGGAGAAGAAGAAACTGATGCTGCCCAACAAATTGGT 416  
1-1512    -------------------------------------------------------------------AGATTCGACGGACCTCGATTTGGTGGCTCCAGACCAGATGGTGCTGGAGGGAGACCTTTCTTCGGCCAAGGAGGCAGGCGTGGTGATGGAGAAGAAGAAACTGATGCTGCCCAACAAATTGGT 416  
1-1514    -------------------------------------------------------------------AGATTCGACGGACCTCGATTTGGTGGCTCCAGACCAGATGGTGCTGGAGGGAGACCTTTCTTCGGCCAAGGAGGCAGGCGTGGTGATGGAGAAGAAGAAACTGATGCTGCCCAACAAATTGGT 416  
1-1528    -------------------------------------------------------------------AGATTCGACGGACCTCGATTTGGTGGCTCCAGACCAGATGGTGCTGGAGGGAGACCTTTCTTCGGCCAAGGAGGCAGGCGTGGTGATGGAGAAGAAGAAACTGATGCTGCCCAACAAATTGGT 416  
1-1532    -------------------------------------------------------------------AGATTCGACGGACCTCGATTTGGTGGCTCCAGACCAGATGGTGCTGGAGGGAGACCTTTCTTCGGCCAAGGAGGCAGGCGTGGTGATGGAGAAGAAGAAACTGATGCTGCCCAACAAATTGGT 416  
1-1539    -------------------------------------------------------------------AGATTCGACGGACCTCGATTTGGTGGCTCCAGACCAGATGGTGCTGGAGGGAGACCTTTCTTCGGCCAAGGAGGCAGGCGTGGTGATGGAGAAGAAGAAACTGATGCTGCCCAACAAATTGGT 416  
1-1536    -------------------------------------------------------------------AGATTCGACGGACCTCGATTTGGTGGCTCCAGACCAGATGGTGCTGGAGGGAGACCTTTCTTCGGCCAAGGAGGCAGGCGTGGTGATGGAGAAGAAGAAACTGATGCTGCCCAACAAATTGGT 415  
1-1535    -------------------------------------------------------------------AGATTCGACGGACCTCGATTTGGTGGCTCCAGACCAGATGGTGCTGGAGGGAGACCTTTCTTCGGCCAAGGAGGCAGGCGTGGTGATGGAGAAGAAGAAACTGATGCTGCCCAACAAATTGGT 416  
1-1534    -------------------------------------------------------------------AGATTCGACGGACCTCGATTTGGTGGCTCCAGACCAGATGGTGCTGGAGGGAGACCTTTCTTCGGCCAAGGAGGCAGGCGTGGTGATGGAGAAGAAGAAACTGATGCTGCCCAACAAATTGGT 416  
1-2402    -------------------------------------------------------------------AGATTCGACGGACCTCGATTTGGTGGCTCCAGACCAGATGGTGCTGGAGGGAGACCTTTCTTCGGCCAAGGAGGCAGGCGTGGTGATGGAGAAGAAGAAACTGATGCTGCCCAACAAATTGGT 416  
1-2404    -------------------------------------------------------------------AGATTCGACGGACCTCGATTTGGTGGCTCCAGACCAGATGGTGCTGGAGGGAGACCTTTCTTCGGCCAAGGAGGCAGGCGTGGTGATGGAGAAGAAGAAACTGATGCTGCCCAACAAATTGGT 414  
1-2405    -------------------------------------------------------------------AGATTCGACGGACCTCGATTTGGTGGCTCCAGACCAGATGGTGCTGGAGGGAGACCTTTCTTCGGCCAAGGAGGCAGGCGTGGTGATGGAGAAGAAGAAACTGATGCTGCCCAACAAATTGGT 416  
1-2406    -------------------------------------------------------------------AGATTCGACGGACCTCGATTTGGTGGCTCCAGACCAGATGGTGCTGGAGGGAGACCTTTCTTCGGCCAAGGAGGCAGGCGTGGTGATGGAGAAGAAGAAACTGATGCTGCCCAACAAATTGGT 416  
1-2407    -------------------------------------------------------------------AGATTCGACGGACCTCGATTTGGTGGCTCCAGACCAGATGGTGCTGGAGGGAGACCTTTCTTCGGCCAAGGAGGCAGGCGTGGTGATGGAGAAGAAGAAACTGATGCTGCCCAACAAATTGGT 414  
1-2412    -------------------------------------------------------------------AGATTCGACGGACCTCGATTTGGTGGCTCCAGACCAGATGGTGCTGGAGGGAGACCTTTCTTCGGCCAAGGAGGCAGGCGTGGTGATGGAGAAGAAGAAACTGATGCTGCCCAACAAATTGGT 416  
1-2413    -------------------------------------------------------------------AGATTCGACGGACCTCGATTTGGTGGCTCCAGACCAGATGGTGCTGGAGGGAGACCTTTCTTCGGCCAAGGAGGCAGGCGTGGTGATGGAGAAGAAGAAACTGATGCTGCCCAACAAATTGGT 416  
1-2416    -------------------------------------------------------------------AGATTCGACGGACCTCGATTTGGTGGCTCCAGACCAGATGGTGCTGGAGGGAGACCTTTCTTCGGCCAAGGAGGCAGGCGTGGTGATGGAGAAGAAGAAACTGATGCTGCCCAACAAATTGGT 414  
1-2417    -------------------------------------------------------------------AGATTCGACGGACCTCGATTTGGTGGCTCCAGACCAGATGGTGCTGGAGGGAGACCTTTCTTCGGCCAAGGAGGCAGGCGTGGTGATGGAGAAGAAGAAACTGATGCTGCCCAACAAATTGGT 414  
1-2418    -------------------------------------------------------------------AGATTCGACGGACCTCGATTTGGTGGCTCCAGACCAGATGGTGCTGGAGGGAGACCTTTCTTCGGCCAAGGAGGCAGGCGTGGTGATGGAGAAGAAGAAACTGATGCTGCCCAACAAATTGGT 416  
1-2420    -------------------------------------------------------------------AGATTCGACGGACCTCGATTTGGTGGCTCCAGACCAGATGGTGCTGGAGGGAGACCTTTCTTCGGCCAAGGAGGCAGGCGTGGTGATGGAGAAGAAGAAACTGATGCTGCCCAACAAATTGGT 414  
1-2421    -------------------------------------------------------------------AGATTCGACGGACCTCGATTTGGTGGCTCCAGACCAGATGGTGCTGGAGGGAGACCTTTCTTCGGCCAAGGAGGCAGGCGTGGTGATGGAGAAGAAGAAACTGATGCTGCCCAACAAATTGGT 414  
1-2422    -------------------------------------------------------------------AGATTCGACGGACCTCGATTTGGTGGCTCCAGACCAGATGGTGCTGGAGGGAGACCTTTCTTCGGCCAAGGAGGCAGGCGTGGTGATGGAGAAGAAGAAACTGATGCTGCCCAACAAATTGGT 416  
1-2425    -------------------------------------------------------------------AGATTCGACGGACCTCGATTTGGTGGCTCCAGACCAGATGGTGCTGGAGGGAGACCTTTCTTCGGCCAAGGAGGCAGGCGTGGTGATGGAGAAGAAGAAACTGATGCTGCCCAACAAATTGGT 416  
1-2426    -------------------------------------------------------------------AGATTCGACGGACCTCGATTTGGTGGCTCCAGACCAGATGGTGCTGGAGGGAGACCTTTCTTCGGCCAAGGAGGCAGGCGTGGTGATGGAGAAGAAGAAACTGATGCTGCCCAACAAATTGGT 416  
1-2427    -------------------------------------------------------------------AGATTCGACGGACCTCGATTTGGTGGCTCCAGACCAGATGGTGCTGGAGGGAGACCTTTCTTCGGCCAAGGAGGCAGGCGTGGTGATGGAGAAGAAGAAACTGATGCTGCCCAACAAATTGGT 416  
1-2428    -------------------------------------------------------------------AGATTCGACGGACCTCGATTTGGTGGCTCCAGACCAGATGGTGCTGGAGGGAGACCTTTCTTCGGCCAAGGAGGCAGGCGTGGTGATGGAGAAGAAGAAACTGATGCTGCCCAACAAATTGGT 416  
1-2430    -------------------------------------------------------------------AGATTCGACGGACCTCGATTTGGTGGCTCCAGACCAGATGGTGCTGGAGGGAGACCTTTCTTCGGCCAAGGAGGCAGGCGTGGTGATGGAGAAGAAGAAACTGATGCTGCCCAACAAATTGGT 416  
1-2431    -------------------------------------------------------------------AGATTCGACGGACCTCGATTTGGTGGCTCCAGACCAGATGGTGCTGGAGGGAGACCTTTCTTCGGCCAAGGAGGCAGGCGTGGTGATGGAGAAGAAGAAACTGATGCTGCCCAACAAATTGGT 414  
1-2432    -------------------------------------------------------------------AGATTCGACGGACCTCGATTTGGTGGCTCCAGACCAGATGGTGCTGGAGGGAGACCTTTCTTCGGCCAAGGAGGCAGGCGTGGTGATGGAGAAGAAGAAACTGATGCTGCCCAACAAATTGGT 416  
1-2433    -------------------------------------------------------------------AGATTCGACGGACCTCGATTTGGTGGCTCCAGACCAGATGGTGCTGGAGGGAGACCTTTCTTCGGCCAAGGAGGCAGGCGTGGTGATGGAGAAGAAGAAACTGATGCTGCCCAACAAATTGGT 416  
1-2434    -------------------------------------------------------------------AGATTCGACGGACCTCGATTTGGTGGCTCCAGACCAGATGGTGCTGGAGGGAGACCTTTCTTCGGCCAAGGAGGCAGGCGTGGTGATGGAGAAGAAGAAACTGATGCTGCCCAACAAATTGGT 414  
1-2435    -------------------------------------------------------------------AGATTCGACGGACCTCGATTTGGTGGCTCCAGACCAGATGGTGCTGGAGGGAGACCTTTCTTCGGCCAAGGAGGCAGGCGTGGTGATGGAGAAGAAGAAACTGATGCTGCCCAACAAATTGGT 414  
1-2436    -------------------------------------------------------------------AGATTCGACGGACCTCGATTTGGTGGCTCCAGACCAGATGGTGCTGGAGGGAGACCTTTCTTCGGCCAAGGAGGCAGGCGTGGTGATGGAGAAGAAGAAACTGATGCTGCCCAACAAATTGGT 414  
1-2437    -------------------------------------------------------------------AGATTCGACGGACCTCGATTTGGTGGCTCCAGACCAGATGGTGCTGGAGGGAGACCTTTCTTCGGCCAAGGAGGCAGGCGTGGTGATGGAGAAGAAGAAACTGATGCTGCCCAACAAATTGGT 416  
1-2439    -------------------------------------------------------------------AGATTCGACGGACCTCGATTTGGTGGCTCCAGACCAGATGGTGCTGGAGGGAGACCTTTCTTCGGCCAAGGAGGCAGGCGTGGTGATGGAGAAGAAGAAACTGATGCTGCCCAACAAATTGGT 414  
1-2440    -------------------------------------------------------------------AGATTCGACGGACCTCGATTTGGTGGCTCCAGACCAGATGGTGCTGGAGGGAGACCTTTCTTCGGCCAAGGAGGCAGGCGTGGTGATGGAGAAGAAGAAACTGATGCTGCCCAACAAATTGGT 414  
1-2441    -------------------------------------------------------------------AGATTCGACGGACCTCGATTTGGTGGCTCCAGACCAGATGGTGCTGGAGGGAGACCTTTCTTCGGCCAAGGAGGCAGGCGTGGTGATGGAGAAGAAGAAACTGATGCTGCCCAACAAATTGGT 416  
1-2442    -------------------------------------------------------------------AGATTCGACGGACCTCGATTTGGTGGCTCCAGACCAGATGGTGCTGGAGGGAGACCTTTCTTCGGCCAAGGAGGTAGGCGTGGTGATGGAGAAGAAGAAACTGATGCTGCCCAACAAATTGGT 414  
1-2414    TGGACCTGGATTTGGTGCCCCGGAGATGGATGGACGGAGACAAAATGGCGGTCCGATGGGTGGAAGGAGATTCGACGGACCTGGATTTGGTGGCTCCAGACCAGATGGTGCTGGAGGAAGACCTTTCTTCGGCCAAGGAGGCAGGCGTGGTGATGGAGAAGAAGAAACTGATGCTGCCCAACAAATTGGT 486  
1-2424    ------------------------------------------------------------------GAGATTCGACGGACCTCGATTTGGTGGCTCCAGACCAGATGGTGCTGGAGGGAGACCTTTCTTCGGCCAAGGAGGCAGGCGTGGTGATGGAGAAGAAGAAACTGATGCTGCCCAACAAATTGGT 326  
1-2429    ------------------------------------------------------------------GAGATTCGACGGACCTCGATTTGGTGGCTCCAGACCAGATGGTGCTGGAGGGAGACCTTTCTTCGGCCAAGGAGGCAGGCGTGGTGATGGAGAAGAAGAAACTGATGCTGCCCAACAAATTGGT 325  
2-1501    TGGACCTGGATTTGGTACCCCGCAGATGGATGGACGGAGACAAAATGGCGGTCCGATGGGTGGTAGGAGATTCGACGGACCTCGATTTGGTGGCTCCGACCAGATGGTGACTGGAGGGAGACCTTTCTTCGGCCAAGGAGGAAGACGTGGTGATGGAGAAGAAGAAACTGATGCTGCCCAACAAATTGGT 489  
2-1502    TGGACCTGGATTTGGTACCCCGCAGATGGATGGACGGAGACAAAATGGCGGTCCGATGGGTGGTAGGAGATTCGACGGACCTCGATTTGGTGGCTCCAGACCAGATGGTGCTGGAGGGAGACCTTTCTTCGGCCAAGGAGGAAGACGTGGTGATGGAGAAGAAGAAACTGATGCTGCCCAACAAATTGGT 489  
2-1505    TGGACCTGGATTTGGTACCCCGCAGATGGATGGACGGAGACAAAATGGCGGTCCGATGGGTGGTAGGAGATTCGACGGACCTCGATTTGGTGGCTCCAGACCAGATGGTGCTGGAGGGAGACCTTTCTTCGGCCAAGGAGGAAGACGTGGTGATGGAGAAGAAGAAACTGATGCTGCCCAACAAATTGGT 489  
2-1506    TGGACCTGGATTTGGTACCCCGCAGATGGATGGACGGAGACAAAATGGCGGTCCGATGGGTGGTAGGAGATTCGACGGACCTCGATTTGGTGGCTCCAGACCAGATGGTGCTGGAGGGAGACCTTTCTTCGGCCAAGGAGGAAGACGTGGTGATGGAGAAGAAGAAACTGATGCTGCCCAACAAATTGGT 489  
2-1507    TGGACCTGGATTTGGTACCCCGCAGATGGATGGACGGAGACAAAATGGCGGTCCGATGGGTGGTAGGAGATTCGACGGACCCCGATTTGGTGGCTCCAGACCAGATGGTGCTGGAGGGAGACCTTTCTTCGGCCAAGGAGGAAGACGTGGTGATGGAGAAGAAGAAACTGATGCTGCCCAACAAATTGGT 490  
2-1508    TGGACCTGGATTTGGTACCCCGCAGATGGATGGACGGAGACAAAATGGCGGTCCGATGGGTGGTAGGAGATTCGACGGACCTCGATTTGGTGGCTCCAGACCAGATGGTGCTGGAGGGAGACCTTTCTTCGGCCAAGGAGGAAGACGTGGTGATGGAGAAGAAGAAACTGATGCTGCCCAACAAATTGGT 489  
2-1509    TGGACCTGGATTTGGTACCCCGCAGATGGATGGACGGAGACAAAATGGCGGTCCGATGGGTGGTAGGAGATTCGACGGACCTCGATTTGGTGGCTCCAGACCAGATGGTGCTGGAGGGAGACCTTTCTTCGGCCAAGGAGGAAGACGTGGTGATGGAGAAGAAGAAACTGATGCTGCCCAACAAATTGGT 489  
2-1510    TGGACCTGGATTTGGTACCCCGCAGATGGATGGACGGAGACAAAATGGCGGTCCGATGGGTGGTAGGAGATTCGACGGACCTCGATTTGGTGGCTCCAGACCAGATGGTGCTGGAGGGAGACCTTTCTTCGGCCAAGGAGGAAGACGTGGTGATGGAGAAGAAGAAACTGATGCTGCCCAACAAATTGGT 481  
2-1514    TGGACCTGGATTTGGTACCCCGCAGATGGATGGACGGAGACAAAATGGCGGTCCGATGGGTGGTAGGAGATTCGACGGACCTCGATTTGGTGGCTCCAGACCAGATGGTGCTGGAGGGAGACCTTTCTTCGGCCAAGGAGGAAGACGTGGTGATGGAGAAGAAGAAACTGATGCTGCCCAACAAATTGGT 489  
2-1511    TGGACCTGGATTTGGTACCCCGCAGATGGATGGACGGAGACAAAATGGCGGTCCGATGGGTGGTAGGAGATTCGACGGACCTCGATTTGGTGGCTCCAGACCAGATGGTGCTGGAGGGAGACCTTTCTTCGGCCAAGGAGGAAGACGTGGTGATGGAGAAGAAGAAACTGATGCCGCCCAACAAATTGGT 489  
2-2401    -------------------------------------------------------------------GGATTCGACGGACCTGGATTTGGTGGCTCCAGACCAGATGGTGCTGGAGGGAGACCTTTCTTCGGCCAAGGAGGCAGGCGTGGTGATGGAGAAGAAGAAACTGATGCTGCCCAACAAATTGGT 413  
2-2404    -------------------------------------------------------------------AGATTCGACGGACCTGGATTTGGTGGCTCCAGACCAGATGGTGCTGGAGGGAGACCTTTCTTCGGCCAAGGAGGCAGGCGTGGTGATGGAGAAGAAGAAACTGATGCTGCCCAACAAATTGGT 413  
2-2406    -------------------------------------------------------------------AGATTCGACGGACCTCGATTTGGTGGCTCCAGACCAGATGGTGCTGGAGGGAGACCTTTCTTCGGCCAAGGAGGAAGACGTGGTGATGGAAAAGAAGAAACTGATGCTGCCCAACAAATTGGT 413  
2-2407    -------------------------------------------------------------------AGATTCGACGGACCTGGATTTGGTGGCTCCAGACCAGATGGTGCTGGAGGGAGACCTTTCTTCGGCCAAGGAGGCAGGCGTGGTGATGGAGAAGAAGAAACTGATGCTGCCCAACAAATTGGT 413  
2-2408    -------------------------------------------------------------------AGATTCGACGGACCTGGATTTGGTGGCTCCCGACCAGATGGTGCTGGAGGGAGACCTTTCTTCGGCCAAGGAGGCAGGCGTGGTGATGGAGAAGAAGAAACTGATGCTGCCCAACAAATTGGT 413  
2-2409    -------------------------------------------------------------------AGATTCGACGGACCTGGATTTGGTGGCTCCAGACCAGATGGTGCTGGAGGGAGACCTTTCTTCGGCCAAGGAGGCAGGCGTGGTGATGGAGAAGAAGAAACTGATGCTGCCCAACAAATTGGT 413  
2-2411    -------------------------------------------------------------------AGATTCGACGGACCTGGATTTGGTGGCTCCAGACCAGATGGTGCTGGAGGGAGACCTTTCTTCGGCCAAGGAGGCAGGCGTGGTGATGGAGAAGAAGAAACTGATGCTGCCCAACAAATTGGT 414  
2-2405    TGGACCTGGATTTGGTGCCCCACATATGGATGGACGCAGACAAAATGGCGGTCCGATGGGTGGTAGGAGATTCGACGGACCTGGATTTGGTGGCTCCAGACCAGATGGTGCTGGAGGAAGACCTTTCTTCGGCCAAGGAGGAAGACGTGGTGATGGAGAAGAAGAAACTGATGCTGCCCAACAAATTGGT 414  
2-2413    TGGACCTGGATTTGGTGCCCCACATATGGATGGACGCAGACAAAATGGCGGTCCGATGGGTGGTAGGAGATTCGACGGACCTGGATTTGGTGGCTCCAGACCAGATGGTGCTGGAGGAAGACCTTTCTTCGGCCAAGGAGGAAGACGTGGTGATGGAGAAGAAGAAACTGATGCTGCCCAACAAATTGGT 414  
2-2403    -------------------------------------------------------------------AGATTCGACGGACCTCGATTTGGTGGCTCCAGACCAGATGGTGCTGGAGGAAGACCTTTCTTCGGCCAAGGAGGCAGGCGTGGTGATGGAGAAGAAGAAACTGATGCTGCCCAACAAATTGGT 416  
2-2415    TGGACCTGGATTTGGTGCCCCGGAGATGGATGGACGGAGACAAAATGGCGGTCCGATGGGTGGAAGGAGATTCGACGGACCTGGATTTGGTGGCTCCAGACCAGTTGGTGCTGGAGGAAGACCTTTCTTCGGCCAAGGAGGCAGGCGTGGTGATGAAGAAGAAGAAACTGATGCTGCCCAACAAATTGGT 488  
2-2414    TGGACCTGGATTTGGTGCCCCGGAGATGGATGGACGGAGACAAAATGGCGGTCCGATGGGTGGAAGGAGATTCGACGGACCTGGATTTGGTGGCTCCAGACCAGATGGTGCTGGAGGAAGACCTTTCTTCGGCCAAGGAGGCAGGCGTGGTGATGGAGAAGAAGAAACTGATGCTGCCCAACAAATCGGT 487  
3-15-1006 TGGACCTGGATTTGGTACCCCGCAGATGGATGGACGGAGACAAAATGGCGGTCCGATGGGTGGTAGGAGATTCGACGGACCTCGATTTGGTGGCTCCAGACCAGATGGTGCTGGAGGGAGACCTTTCTTCGGCCAAGGAGGAAGACGTGGTGATGGAGAAGAAGAAACTGATGCTGCCCAACAAATTGGT 490  
3-15-4003 TGGACCTGGATTTGGTACCCCGCAGATGGATGGACGGAGACAAAATGGCGGTCCGATGGGTGGTAGGAGATTCGACGGACCTCGATTTGGTGGCTCCAGACCAGATGGTGCTGGAGGGAGACCTTTCTTCGGCCAAGGAGGAAGACGTGGTGATGGAGAAGAAGAAACTGATGCTGCCCAACAAATTGGT 489  
3-15-4004 TGGACCTGGATTTGGTACCCCGCAGATGGATGGACGGAGACAAAATGGCGGTCCGATGGGTGGTAGGAGATTCGACGGACCTCGATTTGGTGGCTCCAGACCAGATGGTGCTGGAGGGAGACCTTTCTTCGGCCAAGGAGGAAGACGTGGTGATGGAGAAGAAGACACTGATGCTGCCCAACAAATCGGT 489  
3-15-4005 TGGACCTGGATTTGGTACCCCGCAGATGGATGGACGGAGACAAAATGGCGGTCCGATGGGTGGTAGGAGATTCGACGGACTTCGATTTGGTGGCTCCAGACCAGATGGTGCTGGAGGGAGACCTTTCTTCGGCCAAGGAGGAAGACGTGGTGATGGAGAAGAAGAAACTGATGCTGCCCAACAAATTGGT 490  
3-15-4007 TGGACCTGGATTTGGTACCCCGCAGATGGATGGACGGAGACAAAATGGCGGTCCGATGGGTGGTAGGAGATTCGACGGACCTCGATTTGGTGGCTCCAGACCAGATGGTGCTGGAGGGAGACCTTTCTTCGGCCAAGGAGGAAGACGTGGTGATGGAGAAGAAGAAACTGATGCTGCCCAACAAATTGGT 488  
3-15-4011 TGGACCTGGATTTGGTACCCCGCAGATGGATGGACGGAGACAAAATGGCGGTCCGATGGGTGGTAGGAGATTCGACGGACCTCGATTTGGTGGCTCCAGACCAGATGGTGCTGGAGGGAGACCTTTCTTCGGCCAAGGAGGAAGACGTGGTGATGGAGAAGAAGAAACTGATGCTGCCCAACAAATTGGT 489  
3-15-4013 TGGACCTGGATTTGGTACCCCGCAGATGGATGGACGGAGACAAAATGGCGGTCCGATGGGTGGTAGGAGATTCGACGGACTTCGATTTGGTGGCTCCAGACCAGATGGTGCTGGAGGGAGACCTTTCTTCGGCCAAGGAGGAAGACGTGGTGATGGAGAAGAAGAAACTGATGCTGCCCAACAAATTGGT 490  
3-15-4015 TGGACCTGGATTTGGTACCCCGCAGATGGATGGACGGAGACAAAATGGCGGTCCGATGGGTGGTAGGAGATTCGACGGACCTCGATTTGGTGGCTCCAGACCAGATGGTGCTGGAGGGAGACCTTTCTTCGGCCAAGGAGGAAGACGTGGTGATGGAGAAGAAGAAACTGATGCTGCCCAACAAATTGGT 489  
3-15-4018 TGGACCTGGATTTGGTACCCCGCAGATGGATGGACGGAGACAAAATGGCGGTCCGATGGGTGGTAGGAGATTCGACGGACCTCGATTTGGTGGCTCCAGACCAGATGGTGCTGGAGGGAGACCTTTCTTCGGCCAAGGAGGAAGACGTGGTGATGGAGAAGAAGAAACTGATGCTGCCCAACAAATTGGT 489  
3-15-4019 TGGACCTGGATTTGGTACCCCGCAGATGGATGGACGGAGACAAAATGGCGGTCCGATGGGTGGTAGGAGATTCGACGGACCTCGATTTGGTGGCTCCAGACCAGATGGTGCTGGAGGGAGACCTTTCTTCGGCCAAGGAGGAAGACGTGGTGATGGAGAAGAAGAAACTGATGCTGCCCAACAAATTGGT 489  
3-15-4022 TGGACCTGGATTTGGTACCCCGCAGATGGATGGACGGAGACAAAATGGCGGTCCGATGGGTGGTAGGAGATTCGACGGACCTCGATTTGGTGGCTCCAGACCAGATGGTGCTGGAGGGAGACCTTTCTTCGGCCAAGGAGGAAGACGTGGTGATGGAGAAGAAGAAACTGATGCTGCCCAACAAATTGGT 490  
3-15-4024 TGGACCTGGATTTGGTACCCCGCAGATGGATGGACGGAGACAAAATGGCGGTCCGATGGGTGGTAGGAGATTCGACGGACCTCGATTTGGTGGCTCCAGACCAGATGGTGCTGGAGGGAGACCTTTCTTCGGCCAAGGAGGAAGACGTGGTGATGGAGAAGAAGAAACTGATGCTGCCCAACAAATTGGT 489  
3-15-4017 TGGACCTGGATTTGGTACCCCGCAGATGGATGGACGGAGACAAAATGGCGGTCCGATGGGTGGTAGGAGATTCGACGGACCTCGATTTGGTGGCTCCAGACCAGATGGTGCTGGAGGGAGACCTTTCTTCGGCCAAGGAGGAAGACGTGGTGATGGAGAAGAAGAAACTGATGCTGCCCAACAAATTGGT 490  
3-15-1003 TGGACCTGGATTTGGTACCCCGCAGATGGATGGACGGAGACAAATGGCGGTCCGATGGGTGGTAGGAGATTCGACGGACCTCGATTTGGTGGCTCCAGACCAGATGGTGCTGGAGGGAGACCTTTCTTCGGCCAAGGAGGAAGACGTGGTGATGGAGAAGAAGAAACTGATGCTGCCCAACAAATTGGTG 489  
3-15-4021 GATGGAGAAGAAGAAACTGATGCTGCCCAACAAATTGGTGATGGTCTAG--------------------------------------------------------------------------------------------------------------------------------------------- 422  
3-15-1002 GATGGAGAAGAAGTAA-------------------------------------------------------------------------------------------------------------------------------------------------------CTGATGCTGCCCAACAAATTGGT 412  
3-15-1004 GATGGAGAAGAAGAAACTGATACTGCCCAACAAATTGGTGATGGTCTAG--------------------------------------------------------------------------------------------------------------------------------------------- 422  
3-15-4008 TGGACCTGGATTTGGTACCCCGCAGATGGATGGACGGAGACAAAATGGCGGTCCGATGGGTGGTAGGAGATTCGACGGACCTCGATTTGGTGGCTCCAGACCAGATGGTGCTGGAGGGAGACCTTTCTTCGGCCAAGGAGGAAGACGTGGTGATGGAGAAGAAGAAACTGATGCTGCCCAACAAATTGGT 487  
3-24-4003 -------------------------------------------------------------------AGATTCGACGGACCTCGATTTGGTGGCTCCAGACCAGATGGTGCTGGAGGGAGACCTTTCTTCGGCCAAGGAGGCAGGCGTGGTGATGGAGAAGAAGAAACTGATGCTGCCCAACAAATTGGT 414  
3-24-4004 -------------------------------------------------------------------AGATTCGACGGACCTCGATTTGGTGGCTCCAGACCAGATGGTGCTAGAGGGAGACCTTTCTTCGGCCAAGGAGGCAGGCGTGGTGATGGAGAAGAAGAAACTGATGCTGCCCAACAAATTGGT 414  
3-24-1006 -------------------------------------------------------------------AGATTCGACGGACCTCGATTTGGTGGCTCCAGACCAGATGGTGCTGGAGGGAGACCTTTCTTCGGCCAAGGAGGCAGGCGTGGTGATGGAGAAGAAGAAACTGATGCTGCCCAACAAATTGGT 414  
3-24-4006 -------------------------------------------------------------------AGATTCGACGGACCTCGATTTGGTGGCTCCAGACCAGATGGTGCTGGAGGGAGACCTTTCTTCGGCCAAGGAGGCAGGCGTGGTGATGGAGAAGAAGAAACTGATGCTGCCCAACAAATTGGT 414  
3-24-4015 -------------------------------------------------------------------AGATTCGACGGACCTCGATTTGGTGGCTCCAGACCAGATGGTGCTGGAGGGAGACCTTTCTTCGGCCAAGGAGGCAGGCGTGGTGATGGAGAAGAAGAAACTGATGCTGCCCAACAAATTGGT 414  
3-24-4021 -------------------------------------------------------------------AGATTCGACGGACCTCGATTTGGTGGCTCCAGACCAGATGGTGCTGGAGGGAGACCTTTCTTCGGCCAAGGAGGCAGGCGTGGTGATGGAGAAGAAGAAACTGATGCTGCCCAACAAATTGGT 414  
3-24-4024 -------------------------------------------------------------------AGATTCGACGGACCTCGATTTGGTGGCTCCAGACCAGATGGTGCTGGAGGGAGACCTTTCTTCGGCCAAGGAGGCAGGCGTGGTGATGGAGAAGAAGAAACTGATGCTGCCCAACAAATTGGT 413  
3-24-4023 -------------------------------------------------------------------AGATTCGACGGACCTCGATTTGGTGGCTCCAGACCAGATGGTGCTGGAGGGAGACCTTTCTTCGGCCAAGGAGGCAGGCGTGGTGATGGAGAAGAAGAAACTGATGCTGCCCAACAAATTGGT 414  
3-24-4001 -------------------------------------------------------------------AGATTCGACGGACCTCGATTTGGTGGCTCCAGACCAGATGGTGCTGGAGGAAGACCTTTCTTCGGCCAAGGAGGCAGGCGTGGTGATGGAGAAGAAGAAACTGATGCTGCCCAACAAATTGGT 413  
3-24-4019 -------------------------------------------------------------------AGATTCGACGGACCTCGATTTGGTGGCTCCAGACCAGATGGTGCTGGAGGAAGACCTTTCTTCGGCCAAGGAGGCAGGCGTGGTGATGGAGAAGAAGAAACTGATGCTGCCCAACAAATTGGT 413  
3-24-1003 -------------------------------------------------------------------AGATTCGACGGACCTCGATTTGGTGGCTCCAGACCAGATGGTGCTGGAGGAAGACCTTTCTTCGGCCAAGGAGGCAGACGTAGTGATGGAGAAGAAGAAACTGATGCTGCCCAACAAATTGGT 413  
3-24-4017 TGGAAATGGATTTGGTGCCCCGGAGATGGATGGACGGAGACAAAATGGCGGTCCGATGGGTGGAAGGAGATTCGACGGACCTGGATCTGGTGGCTCCAGACCAGATGGTGCTGGAGGAAGACCTTTCTTCGGCCAAGGAGGCAGGCGTGGTGATGGAGAAGAAGAAACTGATGCTGCCCAACAAATTGGT 488  
3-24-4016 TGGACCTGGATTTGGTGCCCCGGAGATGGATGGACGGAGACAAAATGGCGGTCCGATGGGTGGAAGGAGATTCGACGGACCTGGATTTGGTGGCTCCAGACCAGATGGTGCTGGAGGAAGACCTTTCTTCGGCCAAGGAGGCAGGCGTGGTGATGGAGAAGAAGAAACTGATGCTGCCCAACAAATTGGT 488  
3-24-4011 TGGACCTGGATTTGGTACCCCGCAGATGGATGGACGGAGACAAAATGGCGGTCCGATGGGTGGTAGGAGATTCGACGGACCTCGATTTGGTGGCTCCAGACCAGATGGTGCTGGAGGGAGACCTTTCTTCGGCCAAGGAGGAAGACCTGGTGATGGAGAAGAAGAAACTGATGCTGCCCAACAAATTGGT 489  
3-24-4005 TGGACCTGGATTTGGTACCCCGCAGATGGATGGACGGAGACAAAATGGCGGTCCGATGGGTGGTAGGAGATTCGACGGACCTCGATTTGGTGGCTCCAGACCAGATGGTGCTGGAGGGAGACCTTTCTTCGGCCAAGGAGGAAGACGTGGTGATGGAGAAGAAGAAACTGATGCTGCCCAACAAATTGGT 490  
3-24-4018 TGGACCTGGATTTGGTACCCCGCAGATGGATGGACGGAGACAAAATGGCGGTCCGATGGGTGGTAGGAGATTCGACGGACCTCGATTTGGTGGCTCCAGACCAGATGGTGCTGGAGGGAGACCTTTCTTCGGCCAAGGAGGAAGACGTGGTGATGGAGAAGAAGAAACTGATGCTGCCCAACAAATTGGT 490  
3-24-4022 TGGACCTGGATTTGGTACCCCGCAGATGATGGACGGAGACAAAATGGCGGTCCGATGGGTGGTAGGAGA-TTCGACGGACCTCGATTTGGTGGCTCCAGACCAGATGGTGCTGGAGGGAGACCTTTCTTCGGCCAAGGAGGAAGACGTGGTGATGGAGAAGAAGAAACTGATGCTGCCCAACAAATTGGTG 489  
6-2415    -------------------------------------------------------------------AGATTCGACGGACCTCGATTTGGTGGCTCCAGACCAGATGGTGCTGGAGGAAGACCTTTCTTCGGCCAAGGAGGCAGGCGTGGTGATGGAGAAGAAGAAACTGATGCTGCCCAACAAATTGGT 413  
6-2426    -------------------------------------------------------------------AGATTCGACGGACCACGATTTGGTGGCTCCAGACCAGATGGTGCTGGAGGAAGACCTTTCTTCGGCCAAGGAGGCAGGCGTGGTGATGGAGAAGAAGAAACTGATGCTGCCCAACAAATTGGT 413  
6-2446    -------------------------------------------------------------------AGATTCGACGGACCTCGATTTGGTGGCTCCAGACCAGATGGTGCTGGAGGAAGACCTTTCTTCGGCCAAGGAGGCAGGCGTGGTGATGGAGAAGAAGAAACTGATGCTGCCCAACAAATTGGT 413  
6-2401    -------------------------------------------------------------------AGATTCGACGGACCTCGATTTGGTGGCTCCAGACCAGATGGTGCTGGAGGGAGACCTTTCTTCGGCCAAGGAGGCAGGCGTGGTGATGGAGAAGAAGAAACTGATGCTGCCCAACAAATTGGT 414  
6-2402    -------------------------------------------------------------------AGATTCGACGGACCTCGATTTGGTGGCTCCAGACCAGATGGTGCTGGAGGGAGACCTTTCTTCGGCCAAGGAGGCAGGCGTGGTGATGGAGAAGAAGAAACTGATGCTGCCCAACAAATTGGT 416  
6-2404    -------------------------------------------------------------------AGATTCGACGGACCTCGATTTGGTGGCTCCAGACCAGATGGTGCTGGAGGGAGACCTTTCTTCGGCCAAGGAGGCAGGCGTGGTGATGGAGAAGAAGAAACTGATGCTGCCCAACAAATTGGT 416  
6-2407    -------------------------------------------------------------------AGATTCGACGGACCTCGATTTGGTGGCTCCAGACCAGATGGTGCTGGAGGGAGACCTTTCTTCGGCCAAGGAGGCAGGCGTGGTGATGGAGAAGAAGAAACTGATGCTGCCCAACAAATTGGT 414  
6-2408    -------------------------------------------------------------------AGATTCGACGGACCTCGATTTGGTGGCTCCAGACCAGATGGTGCTGGAGGGAGACCTTTCTTCGGCCAAGGAGGCAGGCGTGGTGATGGAGAAGAAGAAACTGATGCTGCCCAACAAATTGGT 416  
6-2409    -------------------------------------------------------------------AGATTCGACGGACCTCGATTTGGTGGCTCCAGACCAGATGGTGCTGGAGGGAGACCTTTCTTCGGCCAAGGAGGCAGGCGTGGTGATGGAGAAGAAGTAACTGATGCTGCCCAACAAATTGGT 414  
6-2410    -------------------------------------------------------------------AGATTCGACGGACCTCGATTTGGTGGCTCCAGACCAGATGGTGCTGGAGGGAGACCTTTCTTCGGCCAAGGAGGCAGGCGTGGTGATGGAGAAGAAGAAACTGATGCTGCCCAACAAATTGGT 416  
6-2411    -------------------------------------------------------------------AGATTCGACGGACCTCGATTTGGTGGCTCCAGACCAGATGGTGCTGGAGGGAGACCTTTCTTCGGCCAAGGAGGCAGGCGTGGTGATGGAGAAGAAGAAACTGATGCTGCCCAACAAATTGGT 416  
6-2412    -------------------------------------------------------------------AGATTCGACGGACCTCGATTTGGTGGCTCCAGACCAGATGGTGCTGGAGGGAGACCTTTCTTCGGCCAAGGAGGCAGGCGTGGTGATGGAGAAGAAGAAACTGATGCTGCCCAACAAATTGGT 416  
6-2413    -------------------------------------------------------------------AGATTCGACGGACCTCGATTTGGTGGCTCCAGACCAGATGGTGCTGGAGGGAGACCTTTCTTCGGCCAAGGAGGCAGGCGTGGTGATGGAGAAGAAGAAACTGATGCTGCCCAACAAATTGGT 414  
6-2414    -------------------------------------------------------------------AGATTCGACGGACCTCGATTTGGTGGCTCCAGACCAGATGGTGCTGGAGGGAGACCTTTCTTCGGCCAAGGAGGCAGGCGTGGTGATGGAGAAGAAGAAACTGATGCTGCCCAACAAGTTGGT 416  
6-2416    -------------------------------------------------------------------AGATTCGACGGACCTCGATTTGGTGGCTCCAGACCAGATGGTGCTGGAGGGAGACCTTTCTTCGGCCAAGGAGGCAGGCGTGGTGATGGAGAAGAAGAAACTGATGCTGCCCAACAAATTGGT 414  
6-2417    -------------------------------------------------------------------AGATTCGACGGACCTCGATTTGGTGGCTCCAGACCAGATGGTGCTGGAGGGAGACCTTTCTTCGGCCAAGGAGGCAGGCGTGGTGATGGAGAAGAAGAAACTGATGCTGCCCAACAAATTGGT 416  
6-2420    -------------------------------------------------------------------AGATTCGACGGACCTCGATTTGGTGGCTCCAGACCAGATGGTGCTGGAGGGAGACCTTTCTTCGGCCAAGGAGGCAGGCGTGGTGATGGAGAAGAAGAAACTGATGCTGCCCAACAAATTGGT 414  
6-2421    -------------------------------------------------------------------AGATTCGACGGACCTCGATTTGGTGGCTCCAGACCAGATGGTGCTGGAGGGAGACCTTTCTTCGGCCAAGGAGGCAGGCGTGGTGATGGAGAAGAAGAAACTGATGCTGCCCAACAAATTGGT 414  
6-2422    -------------------------------------------------------------------AGATTCGACGGACCTCGATTTGGTGGCTCCAGACCAGATGGTGCTGGAGGGAGACCTTTCTTCGGCCAAGGAGGCAGGCGTGGTGATGGAGAAGAAGAAACTGATGCTGCCCAACAAATTGGT 416  
6-2424    -------------------------------------------------------------------AGATTCGACGGACCTCGATTTGGTGGCTCCAGACCAGATGGTGCTGGAGGGAGACCTTTCTTCGGCCAAGGAGGCAGGCGTGGTGATGGAGAAGAAGAAACTGATGCTGCCCAACAAATTGGT 414  
6-2425    -------------------------------------------------------------------AGATTCGACGGACCTCGATTTGGTGGCTCCAGACCAGATGGTGCTGGAGGGAGACCTTTCTTCGGCCAAGGAGGCAGGCGTGGTGATGGAGAAGAAGAAACTGATGCTGCCCAACAAATTGGT 416  
6-2427    -------------------------------------------------------------------AGATTCGACGGACCTCGATTTGGTGGCTCCAGACCAGATGGTGCTGGAGGGAGACCTTTCTTCGGCCAAGGAGGCAGGCGTGGTGATGGAGAAGAAGAAACTGATGCTGCCCAACAAATTGGT 414  
6-2428    -------------------------------------------------------------------AGATTCGACGGACCTCGATTTGGTGGCTCCAGACCAGATGGTGCTGGAGGGAGACCTTTCTTCGGCCAAGGAGGCAGGCGTGGTGATGGAGAAGAAGAAACTGATGCTGCCCAACAAATTGGT 414  
6-2430    -------------------------------------------------------------------AGATTCGACGGACCTCGATTTGGTGGCTCCAGACCAGATGGTGCTGGAGGGAGACCTTTCTTCGGCCAAGGAGGCAGGCGTGGTGATGGAGAAGAAGAAACTGATGCTGCCCAACAAATTGGT 416  
6-2431    -------------------------------------------------------------------AGATTCGACGGACCTCGATTTGGTGGCTCCAGACCAGATGGTGCTGGAGGGAGACCTTTCTTCGGCCAAGGAGGCAGGCGTGGTGATGGAGAAGAAGAAACTGATGCTGCCCAACAAATTGGT 414  
6-2432    -------------------------------------------------------------------AGATTCGACGGACCTCGATTTGGTGGCTCCAGACCAGATGGTGCTGGAGGGAGACCTTTCTTCGGCCAAGGAGGCAGGCGTGGTGATGGAGAAGAAGAAACTGATGCTGCCCAACAAATTGGT 414  
6-2433    -------------------------------------------------------------------AGATTCGACGGACCTCGATTTGGTGGCTCCAGACCAGATGGTGCTGGAGGGAGACCTTTCTTCGGCCAAGGAGGCAGGCGTGGTGATGGAGAAGAAGAAACTGATGCTGCCCAACAAATTGGT 414  
6-2434    -------------------------------------------------------------------AGATTCGACGGACCTCGATTTGGTGGCTCCAGACCAGATGGTGCTGGAGGGAGACCTTTCTTCGGCCAAGGAGGCAGGCGTGGTGATGGAGAAGAAGAAACTGATGCTGCCCAACAAATTGGT 414  
6-2439    -------------------------------------------------------------------AGATTCGACGGACCTCGATTTGGTGGCTCCAGACCAGATGGTGCTGGAGGGAGACCTTTCTTCGGCCAAGGAGGCAGGCGTGGTGATGGAGAAGAAGAAACTGATGCTGCCCAACAAATTGGT 414  
6-2440    -------------------------------------------------------------------AGATTCGACGGACCTCGATTTGGTGGCTCCAGACCAGATGGTGCTGGAGGGAGACCTTTCTTCGGCCAAGGAGGCAGGCGTGGTGATGGAGAAGAAGAAACTGATGCTGCCCAACAAATTGGT 416  
6-2441    -------------------------------------------------------------------AGATTCGACGGACCTCGATTTGGTGGCTCCAGACCAGATGGTGCTGGAGGGAGACCTTTCTTCGGCCAAGGAGGCAGGCGTGGTGATGGAGAAGAAGAAACTGATGCTGCCCAACAAATTGGT 416  
6-2435    -------------------------------------------------------------------AGATTCGACGGACCTCGATTTGGTGGCTCCAGACCAGATGGTGCTGGAGGGAGACCTTTCTTCGGCCAAGGAGGCAGGCGTGGTGATGGAGAAGAAGAAACTGATGCTGCCCAACAAATTGGT 413  
6-2447    -------------------------------------------------------------------AGATTCGACGGACCTCGATTTGGTGGCTCCAGACCAGATGGTGCTGGAGGGAGACCTTTCTTCGGCCAAGGAGGCAGGCGTGGTGATGGAGAAGAAGAAACTGATGCTGCCCAACAAATTGGT 414  
6-2448    -------------------------------------------------------------------AGATTCGACGGACCTCGATTTGGTGGCTCCAGACCAGATGGTGCTGGAGGGAGACCTTTCTTCGGCCAAGGAGGCAGGCGTGGTGATGGAGAAGAAGAAACTGATGCTGCCCAACAAATTGGT 416  
6-2436    -------------------------------------------------------------------AGATTCGACGGACCTCGATTTGGTGGCTCCAGACCAGATGGTGCTGGAGGGAGACCTTTCTTCGGCCAAGGAGGCAGGCGTGGTGATGGAGAAGAAGAAACTGATGCTGCCCAACAAATTGGT 415  
6-2450    -------------------------------------------------------------------AGATTCGACGGACCTCGATTTGGTGGCTCCAGACCAGATGGTGCTGGAGGGAGACCTTTCTTCGGCCAAGGAGGCAGGCGTGGTGATGGAGAAGAAGAAACTGATGCTGCCCAACAAATTGGT 414  
6-2429    TGGACCTGGATTTGGTGCCCCGGAGATGGATGGACGGAGACAAAATGGCGGTCCGATGGGTGGAAGGAGATTCGACGGACCTGGATTTGGTGGCTCCAGACCAGATGGTGCTGGAGGAAGACCTTTCTTCGGCCAAGGAGGCAGGCGTGGTGATGGAGAAGAAGAAACTGATGCTGCCCAACAAATTGGT 414  
6-2438    TGGACCTGGATTTGGTGCCCCACATATGGATGGACGCAGACAAAATGGCGGTCCGATGGGTGGTAGGAGATTCGACGGACCTCGATTTGGTGGCTCCAGACCAGATGGTGCTGGAGGAAGACCTTTCTTCGGCCAAGGAGGAAGACGTGGTGATGGAGAAGAAGAAACTGATGCTGCCCAACAAATTGGT 414  
6-2423    TGGACCTGGATTTGGTGCCCCACATATGGATGGACGCAGACAAAATGGCGGTCCGATGGGTGGTAGGAGATTCGACGGACCTCGATTTGGTGGCTCCAGACCAGATGGTGCTGGAGGAAGACCTTTCTTCGGCCAAGGAGGAAGACGTGGTGATGGAGAAGAAGAAACTGATGCTGCCCAACAAATTGGT 414  
6-2403    -------------------------------------------------------------------AGATTCGACGGACCTCGATTTGGTGGCTCCAGACCAGATGGTGCTGGAGGGAGACCTTTCTTCGGCCAAGGAGGCAGGCGTGGTGATGGAGAAGAAGAAACTGATGCTGCCCAACAAATTGGT 416  
6-2444    -------------------------------------------------------------------AGATTCGACGGACCTCGATTTGGTGGCTCCAGACCAGATGGTGCTGGAGGGAGACCTTTCTTCGGCCAAGGAGGCAGGCGTGGTGATGGAGAAGAAGAAACTGATGCTGCCCAACAAATTGGT 416  
6-2449    -------------------------------------------------------------------AGATTCGACGGACCTCGATTTGGTGGCTCCAGACCAGATGGTGCTGGAGGGAGACCTTTCTTCGGCCAAGGAGGCAGGCGTGGTGATGGAGAAGAAGAAACTGATGCTGCCCAACAAATTGGT 418  
2-1503    -------------------------------------------------------------------AGATTCGACGGACCTCGATTTGGTGGCTCCAGACCAGATGGTGCTGGAGGGAGACCTTTCTTCGGCCAAGGAGGCAGGCGTGGTGATGGAGAAGAAGAAACTGATGCTGCCCAACAAATTGGT 419  
2-1509    -------------------------------------------------------------------AGATTCGACGGACCTCGATTTGGTGGCTCCAGACCAGATGGTGCTGGAGGGAGACCTTTCTTCGGCCAAGGAGGCAGGCGTGGTGATGGAGAAGAAGAAACTGATGCTGCCCAACAAATTGGT 419  
2-1513    -------------------------------------------------------------------AGATTCGACGGACCTCGATTTGGTGGCTCCAGACCAGATGGTGCTGGAGGGAGACCTTTCTTCGGCCAAGGAGGCAGGCGTGGTGATGGAGAAGAAGAAACTGATGCTGCCCAACAAATTGGT 419  
2-1523    -------------------------------------------------------------------AGATTCGACGGACCTCGATTTGGTGGCTCCAGACCAGATGGTGCTGGAGGGAGACCTTTCTTCGGCCAAGGAGGCAGGCGTGGTGATGGAGAAGAAGAAACTGATGCTGCCCAACAAATTGGT 419  
2-1524    -------------------------------------------------------------------AGATTCGACGGACCTCGATTTGGTGGCTCCAGACCAGATGGTGCTGGAGGGAGACCTTTCTTCGGCCAAGGAGGCAGGCGTGGTGATGGAGAAGAAGAAACTGATGCTGCCCAACAAATTGGT 419  
2-1531    -------------------------------------------------------------------AGATTCGACGGACCTCGATTTGGTGGCTCCAGACCAGATGGTGCTGGAGGGAGACCTTTCTTCGGCCAAGGAGGCAGGCGTGGTGATGGAGAAGAAGAAACTGATGCTGCCCAACAAATTGGT 419  
2-1533    -------------------------------------------------------------------AGATTCGACGGACCTCGATTTGGTGGCTCCAGACCAGATGGTGCTGGAGGGAGACCTTTCTTCGGCCAAGGAGGCAGGCGTGGTGATGGAGAAGAAGAAACTGATGCTGCCCAACAAATTGGT 419  
2-1536    -------------------------------------------------------------------AGATTCGACGGACCTCGATTTGGTGGCTCCAGACCAGATGGTGCTGGAGGGAGACCTTTCTTCGGCCAAGGAGGCAGGCGTGGTGATGGAGAAGAAGAAACTGATGCTGCCCAACAAATTGGT 418  
2-1502    -------------------------------------------------------------------AGATTCGACGGACCTCGATTTGGTGGCTCCAGACCAGATGGTGCTGGAGGGAGACCTTTCTTCGGCCAAGGAGGCAGGCGTGGTGATGGAGAAGAAGAAACTGATGCTGCCCAACAAATTGGT 419  
2-1518    -------------------------------------------------------------------AGATTCGACGGACCTCGATTTGGTGGCTCCAGACCAGATGGTGCTGGAGGGAGACCTTTCTTCGGCCAAGGAGGCAGGCGTGGTGATGGAGAAGAAGAAACTGATGCTGCCCAACAAATTGGT 417  
2-1519    -------------------------------------------------------------------AGATTCGACGGACCTCGATTTGGTGGCTCCAGACCAGATGGTGCTGGAGGGAGACCTTTCTTCGGCCAAGGAGGCAGGCGTGGTGATGGAGAAGAAGAAACTGATGCTGCCCAACAAATTGGT 417  
2-1511    -------------------------------------------------------------------AGATTCGACGGACCTCGATTTGGTGGCTCCAGACCAGATGGTGCTGGAGGGAGACCTTTCTTCGGCCAAGGAGGCAGGCGTGGTGATGGAGAAGAAGAAACTGATGCTGCCCAACAAATTGGT 417  
2-1546    -------------------------------------------------------------------AGATTCGACGGACCTCGATTTGGTGGCTCCAGACCAGATGGTGCTGGAGGGAGACCTTTCTTCGGCCAAGGAGGCAGGCGTGGTGATGGAGAAGAAGAAACTGATGCTGCCCAACAAATTGGT 417  
2-1548    -------------------------------------------------------------------AGATTCGACGGACCTCGATTTGGTGGCTCCAGACCAGATGGTGCTGGAGGGAGACCTTTCTTCGGCCAAGGAGGCAGGCGTGGTGATGGAGAAGAAGAAACTGATGCTGCCCAACAAATTGGT 417  
2-1540    -------------------------------------------------------------------AGATTCGACGGACCTCGATTTGGTGGCTCCAGACCAGATGGTGCTGGAGGGAGACCTTCTTCGGCCAAGGAGGCAGGCGTGGTGATGGAGAAGAAGAAACTGATGCTGCCCAACAAATTGGTG 419  
2-2423    -------------------------------------------------------------------AGATTCGACGGACCTCGATTTGGTGGCTCCAGACCAGATGGTGCTGGAGGGAGACCTTTCTTCGGCCAAGGAGGCAGGCGTGGTGATGGAGAAGAAGAAACTGATGCTGCCCAACAAATTGGT 416  
2-2436    -------------------------------------------------------------------AGATTCGACGGACCTCGATTTGGTGGCTCCAGACCAGATGGTGCTGGAGGGAGACCTTTCTTCGGCCGAGGAGGCAGGCGTGGTGATGGAGAAGAAGAAACTGATGCTGCCCAACAAATTGGT 416  
2-2403    -------------------------------------------------------------------AGATTCGACGGACCTCGATTTGGTGGCTCCAGACCAGATGGTGCTGGAGGGAGACCTTTCTTCGGCCAAGGAGGCAGGCGTGGTGATGGAGAAGAAGAAACTGATGCTGCCCAACAAATTGGT 417  
2-2448    -------------------------------------------------------------------AGATTCGACGGACCTCGATTTGGTGGCTCCAGACCAGATGGTGCTGGAGGGAGACCTTTCTTCGGCCAAGGAGGCAGGCGTGGTGATGGAGAAGAAGAAACTGATGCTGCCCAACAAATTGGT 416  
2-2404    -------------------------------------------------------------------AGATTCGACGGACCTCGATTTGGTGGCTCCAGACCAGATGGTGCTGGAGGGAGACCTTTCTTCGGCCAAGGAGGCAGGCGTGGTGATGGAGAAGAAGAAACTGATGCTGCCCAACAAATTGGT 417  
2-2405    TGGACCTGGATTTGGTGCCCCGGAGATGGATGGACGGAGACAAAATGGCGGTCCGATGGGTGGAAGGAGATTCGACGGACCTGGATTTGGTGGCTCCAGACCAGATGGTGCTGGAGGAAGACCTTTCTTCGGCCAAGGAGGCAGGCGTGGTGATGGAGAAGAAGAAACTGATGCTGCCCAACAAATTGGT 420  
2-2406    -------------------------------------------------------------------AGATTCGACGGACCTCGATTTGGTGGCTCCAGACCAGATGGTGCTGGAGGGAGACCTTTCTTCGGCCAAGGAGGCAGGCGTGGTGATGGAGAAGAAGAAACTGATGCTGCCCAACAAATTGGT 414  
2-2409    -------------------------------------------------------------------AGATTCGACGGACCTCGATTTGGTGGCTCCAGACCAGATGGTGCTGGAGGGAGACCTTTCTTCGGCCAAGGAGGCAGGCGTGGTGATGGAGAAGAAGAAACTGATGCTGCCCAACAAATTGGT 416  
2-2410    -------------------------------------------------------------------AGATTCGACGGACCTCGATTTGGTGGCTCCAGACCAGATGGTGCTGGAGGGAGACCTTTCTTCGGCCAAGGAGGCAGGCGTGGTGATGGAGAAGAAGAAACTGATGCTGCCCAACAAATTGGT 417  
2-2411    -------------------------------------------------------------------AGATTCGACGGACCTCGATTTGGTGGCTCCAGACCAGATGGTGCTGGAGGGAGACCTTTCTTCGGCCAAGGAGGCAGGCGTGGTGATGGAGAAGAAGAAACTGATGCTGCCCAACAAATTGGT 417  
2-2412    -------------------------------------------------------------------AGATTCGACGGACCTCGATTTGGTGGCTCCAGACCAGATGGTGCTGGAGGGAGACCTTTCTTCGGCCAAGGAGGCAGGCGTGGTGATGGAGAAGAAGAAACTGATGCTGCCCAACAAATTGGT 416  
2-2413    -------------------------------------------------------------------AGATTCGACGGACCTCGATTTGGTGGCTCCAGACCAGATGGTGCTGGAGGGAGACCTTTCTTCGGCCAAGGAGGCAGGCGTGGTGATGGAGAAGAAGAAACTGATGCTGCCCAACAAATTGGT 416  
2-2415    -------------------------------------------------------------------AGATTCGACGGACCTCGATTTGGTGGCTCCAGACCAGATGGTGCTGGAGGGAGACCTTTCTTCGGCCAAGGAGGCAGGCGTGGTGATGGAGAAGAAGAAACTGATGCTGCCCAACAAATTGGT 417  
2-2416    -------------------------------------------------------------------AGATTCGACGGACCTCGATTTGGTGGCTCCAGACCAGATGGTGCTGGAGGGAGACCTTTCTTCGGCCAAGGAGGCAGGCGTGGTGATGGAGAAGAAGAAACTGATGCTGCCCAACAAATTGGT 416  
2-2417    -------------------------------------------------------------------AGATTCGACGGACCTCGATTTGGTGGCTCCAGACCAGATGGTGCTGGAGGGAGACCTTTCTTCGGCCAAGGAGGCAGGCGTGGTGATGGAGAAGAAGAAACTGATGCTGCCCAACAAATTGGT 416  
2-2418    -------------------------------------------------------------------AGATTCGACGGACCTCGATTTGGTGGCTCCAGACCAGATGGTGCTGGAGGGAGACCTTTCTTCGGCCAAGGAGGCAGGCGTGGTGATGGAGAAGAAGAAACTGATGCTGCCCAACAAATTGGT 416  
2-2419    -------------------------------------------------------------------AGATTCGACGGACCTCGATTTGGTGGCTCCAGACCAGATGGTGCTGGAGGGAGACCTTTCTTCGGCCAAGGAGGAAGACGTGGTGATGGAAAAGAAGAAACTGATGCTGCCCAACAAATTGGT 416  
2-2420    -------------------------------------------------------------------AGATTCGACGGACCTCGATTTGGTGGCTCCAGACCAGATGGTGCTGGAGGGAGACCTTTCTTCGGCCAAGGAGGCAGGCGTGGTGATGGAGAAGAAGAAACTGATGCTGCCCAACAAATTGGT 417  
2-2421    -------------------------------------------------------------------AGATTCGACGGACCTCGATTTGGTGGCTCCAGACCAGATGGTGCTGGAGGGAGACCTTTCTTCGGCCAAGGAGGCAGGCGTGGTGATGGAGAAGAAGAAACTGATGCTGCCCAACAAATTGGT 416  
2-2422    -------------------------------------------------------------------AGATTCGACGGACCTCGATTTGGTGGCTCCAGACCAGATGGTGCTGGAGGGAGACCTTTCTTCGGCCAAGGAGGCAGGCGTGGTGATGGAGAAGAAGAAACTGATGCTGCCCAACAAATTGGT 416  
2-2424    -------------------------------------------------------------------AGATTCGACGGACCTCGATTTGGTGGCTCCAGACCAGATGGTGCTGGAGGGAGACCTTTCTTCGGCCAAGGAGGCAGGCGTGGTGATGGAGAAGAAGAAACTGATGCTGCCCAACAAATTGGT 418  
2-2425    -------------------------------------------------------------------AGATTCGACGGACCTCGATTTGGTGGCTCCAGACCAGATGGTGCTGGAGGGAGACCTTTCTTCGGCCAAGGAGGCAGGCGTGGTGATGGAGAAGAAGAAACTGATGCTGCCCAACAAATTGGT 416  
2-2426    -------------------------------------------------------------------AGATTCGACGGACCTCGATTTGGTGGCTCCAGACCAGATGGTGCTGGAGGGAGACCTTTCTTCGGCCAAGGAGGCAGGCGTGGTGATGGAGAAGAAGAAACTGATGCTGCCCAACAAATTGGT 417  
2-2427    -------------------------------------------------------------------AGATTCGACGGACCTCGATTTGGTGGCTCCAGACCAGATGGTGCTGGAGGGAGACCTTTCTTCGGCCAAGGAGGCAGGCGTGGTGATGGAGAAGAAGAAACTGATGCTGCCCAACAAATTGGT 417  
2-2430    -------------------------------------------------------------------AGATTCGACGGACCTCGATTTGGTGGCTCCAGACCAGATGGTGCTGGAGGGAGACCTTTCTTCGGCCAAGGAGGCAGGCGTGGTGATGGAGAAGAAGAAACTGATGCTGCCCAACAAATTGGT 416  
2-2431    -------------------------------------------------------------------AGATTCGACGGACCTCGATTTGGTGGCTCCAGACCAGATGGTGCTGGAGGGAGACCTTTCTTCGGCCAAGGAGGCAGGCGTGGTGATGGAGAAGAAGAAACTGATGCTGCCCAACAAATTGGT 409  
2-2432    -------------------------------------------------------------------AGATTCGACGGACCTCGATTTGGTGGCTCCAGACCAGATGGTGCTGGAGGGAGACCTTTCTTCGGCCAAGGAGGCAGGCGTGGTGATGGAGAAGAAGAAACTGATGCTGCCCAACAAATTGGT 416  
2-2434    -------------------------------------------------------------------AGATTCGACGGACCTCGATTTGGTGGCTCCAGACCAGATGGTGCTGGAGGGAGACCTTTCTTCGGCCAAGGAGGCAGGCGTGGTGATGGAGAAGAAGAAACTGATGCTGCCCAACAAATTGGT 416  
2-2437    -------------------------------------------------------------------AGATTCGACGGACCTCGATTTGGTGGCTCCAGACCAGATGGTGCTGGAGGGAGACCTTTCTTCGGCCAAGGAGGCAGGCGTGGTGATGGAGAAGAAGAAACTGATGCTGCCCAACAAATTGGT 417  
2-2438    -------------------------------------------------------------------AGATTCGACGGACCTCGATTTGGTGGCTCCAGACCAGATGGTGCTGGAGGGAGACCTTTCTTCGGCCAAGGAGGCAGGCGTGGTGATGGAGAAGAAGAAACTGATGCTGCCCAACAAATTGGT 416  
2-2439    -------------------------------------------------------------------AGATTCGACGGACCTCGATTTGGTGGCTCCAGACCAGATGGTGCTGGAGGGAGACCTTTCTTCGGCCAAGGAGGCAGGCGTGGTGATGGAGAAGAAGAAACTGATGCTGCCCAACAAATTGGT 416  
2-2440    -------------------------------------------------------------------AGATTCGACGGACCTCGATTTGGTGGCTCCAGACCAGATGGTGCTGGAGGGAGACCTTTCTTCGGCCAAGGAGGCAGGCGTGGTGATGGAGAAGAAGAAACTGATGCTGCCCAACAAATTGGT 414  
2-2442    -------------------------------------------------------------------AGATTCGACGGACCTCGATTTGGTGGCTCCAGACCAGATGGTGCTGGAGGGAGACCTTTCTTCGGCCAAGGAGGCAGGCGTGGTGAAGGAGAAGAAGAAACTGATGCTGCCCAACAAATTGGT 415  
2-2445    -------------------------------------------------------------------AGATTCGACGGACCTCGATTTGGTGGCTCCAGACCAGATGGTGCTGGAGGGAGACCTTTCTTCGGCCAAGGAGGCAGGCGTGGTGATGGAGAAGAAGAAACTGATGCTGCCCAACAAATTGGT 413  
2-2446    -------------------------------------------------------------------AGATTCGACGGACCTCGATTTGGTGGCTCCAGACCAGATGGTGCTGGAGGGAGACCTTTCTTCGGCCAAGGAGGCAGGCGTGGTGATGGAGAAGAAGAAACTGATGCTGCCCAACAAATTGGT 416  
7-1501    -------------------------------------------------------------------AGATTCGACGGACCTCGATTTGGTGGCTCCAGACCAGATGGTGCTGGAGGGAGACCTTTCTTCGGCCAAGGAGGCAGGCGTGGTGATGGAGAAGAAGAAACTGATGCTGCCCAACAAATTGGT 416  
7-1502    -------------------------------------------------------------------AGATTCGACGGACCTCGATTTGGTGGCTCCAGACCAGATGGTGCTGGAGGGAGACCTTTCTTCGGCCAAGGAGGCAGGCGTGGTGATGGAGAAGAAGAAACTGATGCTGCCCAACAAATTGGT 416  
7-1503    -------------------------------------------------------------------AGATTCGACGGACCTCGATTTGGTGGCTCCAGACCAGATGGTGCTGGAGGGAGACCTTTCTTCGGCCAAGGAGGCAGGCGTGGTGATGGAGAAGAAGAAACTGATGCTGCCCAACAAATTGGT 416  
7-1504    -------------------------------------------------------------------AGATTCGACGGACCTCGATTTGGTGGCTCCAGACCAGATGGTGCTGGAGGGAGACCTTTCTTCGGCCAAGGAGGCAGGCGTGGTGATGGAGAAGAAGAAACTGATGCTGCCCAACAAATTGGT 416  
7-1505    -------------------------------------------------------------------AGATTCGACGGACCTCGATTTGGTGGCTCCAGACCAGATGGTGCTGGAGGGAGACCTTTCTTCGGCCAAGGAGGCAGGCGTGGTGATGGAGAAGAAGAAACTGATGCTGCCCAACAAATTGGT 416  
7-1506    -------------------------------------------------------------------AGATTCGACGGACCTCGATTTGGTGGCTCCAGACCAGATGGTGCTGGAGGGAGACCTTTCTTCGGCCAAGGAGGCAGGCGTGGTGATGGAGAAGAAGAAACTGATGCTGCCCAACAAATTGGT 416  
7-1508    -------------------------------------------------------------------AGATTCGACGGACCTCGATTTGGTGGCTCCAGACCAGATGGTGCTGGAGGGAGACCTTTCTTCGGCCAAGGAGGCAGGCGTGGTGATGGAGAAGAAGAAACTGATGCTGCCCAACAAATTGGT 416  
7-1509    -------------------------------------------------------------------AGATTCGACGGACCTCGATTTGGTGGCTCCAGACCAGATGGTGCTGGAGGGAGACCTTTCTTCGGCCAAGGAGGCAGGCGTGGTGATGGAGAAGAAGAAACTGATGCTGCCCAACAAATTGGT 416  
7-1510    -------------------------------------------------------------------AGATTCGACGGACCTCGATTTGGTGGCTCCAGACCAGATGGTGCTGGAGGGAGACCTTTCTTCGGCCAAGGAGGCAGGCGTGGTGATGGAGAAGAAGAAACTGATGCTGCCCAACAAATTGGT 416  
7-1511    -------------------------------------------------------------------AGATTCGACGGACCTCGATTTGGTGGCTCCAGACCAGATGGTGCTGGAGGGAGACCTTTCTTCGGCCAAGGAGGCAGGCGTGGTGATGGAGAAGAAGAAACTGATGCTGCCCAACAAATTGGT 416  
7-1512    -------------------------------------------------------------------AGATTCGACGGACCTCGATTTGGTGGCTCCAGACCAGATGGTGCTGGAGGGAGACCTTTCTTCGGCCAAGGAGGCAGGCGTGGTGATGGAGAAGAAGAAACTGATGCTGCCCAACAAATTGGT 416  
7-1513    -------------------------------------------------------------------AGATTCGACGGACCTCGATTTGGTGGCTCCAGACCAGATGGTGCTGGAGGGAGACCTTTCTTCGGCCAAGGAGGCAGGCGTGGTGATGGAGAAGAAGAAACTGATGCTGCCCAACAAATTGGT 416  
7-1515    -------------------------------------------------------------------AGATTCGACGGACCTCGATTTGGTGGCTCCAGACCAGATGGTGCTGGAGGGAGACCTTTCTTCGGCCAAGGAGGCAGGCGTGGTGATGGAGAAGAAGAAACTGATGCTGCCCAACAAATTGGT 416  
7-1516    -------------------------------------------------------------------AGATTCGACGGACCTCGATTTGGTGGCTCCAGACCAGATGGTGCTGGAGGGAGACCTTTCTTCGGCCAAGGAGGCAGGCGTGGTGATGGAGAAGAAGAAACTGATGCTGCCCAACAAATTGGT 416  
7-1517    -------------------------------------------------------------------AGATTCGACGGACCTCGATTTGGTGGCTCCAGACCAGATGGTGCTGGAGGGAGACCTTTCTTCGGCCAAGGAGGCAGGCGTGGTGATGGAGAAGAAGAAACTGATGCTGCCCAACAAATTGGT 414  
7-1519    -------------------------------------------------------------------AGATTCGACGGACCTCGATTTGGTGGCTCCAGACCAGATGGTGCTGGAGGGAGACCTTTCTTCGGCCAAGGAGGCAGGCGTGGTGATGGAGAAGAAGAAACTGATGCTGCCCAACAAATTGGT 416  
7-1520    -------------------------------------------------------------------AGATTCGACGGACCTCGATTTGGTGGCTCCAGACCAGATGGTGCTGGAGGGAGACCTTTCTTCGGCCAAGGAGGCAGGCGTGGTGATGGAGAAGAAGAAACTGATGCTGCCCAACAAATTGGT 416  
7-1521    -------------------------------------------------------------------AGATTCGACGGACCTCGATTTGGTGGCTCCAGACCAGATGGTGCTGGAGGGAGACCTTTCTTCGGCCAAGGAGGCAGGCGTGGTGATGGAGAAGAAGAAACTGATGCTGCCCAACAAATTGGT 416  
7-1523    -------------------------------------------------------------------AGATTCGACGGACCTCGATTTGGTGGCTCCAGACCAGATGGTGCTGGAGGGAGACCTTTCTTCGGCCAAGGAGGCAGGCGTGGTGATGGAGAAGAAGAAACTGATGCTGCCCAACAAATTGGT 416  
7-1524    -------------------------------------------------------------------AGATTCGACGGACCTCGATTTGGTGGCTCCAGACCAGATGGTGCTGGAGGGAGACCTTTCTTCGGCCAAGGAGGCAGGCGTGGTGATGGAGAAGAAGAAACTGATGCTGCCCAACAAATTGGT 416  
7-1525    -------------------------------------------------------------------AGATTCGACGGACCTCGATTTGGTGGCTCCAGACCAGATGGTGCTGGAGGGAGACCTTTCTTCGGCCAAGGAGGCAGGCGTGGTGATGGAGAAGAAGAAACTGATGCTGCCCAACAAATTGGT 416  
7-1526    -------------------------------------------------------------------AGATTCGACGGACCTCGATTTGGTGGCTCCAGACCAGATGGTGCTGGAGGGAGACCTTTCTTCGGCCAAGGAGGCAGGCGTGGTGATGGAGAAGAAGAAACTGATGCTGCCCAACAAATTGGT 416  
7-1527    -------------------------------------------------------------------AGATTCGACGGACCTCGATTTGGTGGCTCCAGACCAGATGGTGCTGGAGGGAGACCTTTCTTCGGCCAAGGAGGCAGGCGTGGTGATGGAGAAGAAGAAACTGATGCTGCCCAACAAATTGGT 415  
7-1528    -------------------------------------------------------------------AGATTCGACGGACCTCGATTTGGTGGCTCCAGACCAGATGGTGCTGGAGGGAGACCTTTCTTCGGCCAAGGAGGCAGGCGTGGTGATGGAGAAGAAGAAACTGATGCTGCCCAACAAATTGGT 416  
7-1529    -------------------------------------------------------------------AGATTCGACGGACCTCGATTTGGTGGCTCCAGACCAGATGGTGCTGGAGGGAGACCTTTCTTCGGCCAAGGAGGCAGGCGTGGTGATGGAGAAGAAGAAACTGATGCTGCCCAACAAATTGGT 416  
7-1530    -------------------------------------------------------------------AGATTCGACGGACCTCGATTTGGTGGCTCCAGACCAGATGGTGCTGGAGGGAGACCTTTCTTCGGCCAAGGAGGCAGGCGTGGTGATGGAGAAGAAGAAACTGATGCTGCCCAACAAATTGGT 416  
7-1533    -------------------------------------------------------------------AGATTCGACGGACCTCGATTTGGTGGCTCCAGACCAGATGGTGCTGGAGGGAGACCTTTCTTCGGCCAAGGAGGCAGGCGTGGTGATGGAGAAGAAGAAACTGATGCTGCCCAACAAATTGGT 416  
7-1534    -------------------------------------------------------------------AGATTCGACGGACCTCGATTTGGTGGCTCCAGACCAGATGGTGCTGGAGGGAGACCTTTCTTCGGCCAAGGAGGCAGGCGTGGTGATGGAGAAGAAGAAACTGATGCTGCCCAACAAATTGGT 416  
7-1536    -------------------------------------------------------------------AGATTCGACGGACCTCGATTTGGTGGCTCCAGACCAGATGGTGCTGGAGGGAGACCTTTCTTCGGCCAAGGAGGCAGGCGTGGTGATGGAGAAGAAGAAACTGATGCTGCCCAACAAATTGGT 416  
7-1537    -------------------------------------------------------------------AGATTCGACGGACCTCGATTTGGTGGCTCCAGACCAGATGGTGCTGGAGGGAGACCTTTCTTCGGCCAAGGAGGCAGGCGTGGTGATGGAGAAGAAGAAACTGATGCTGCCCAACAAATTGGT 416  
7-1538    -------------------------------------------------------------------AGATTCGACGGACCTCGATTTGGTGGCTCCAGACCAGATGGTGCTGGAGGGAGACCTTTCTTCGGCCAAGGAGGCAGGCGTGGTGATGGAGAAGAAGAAACTGATGCTGCCCAACAAATTGGT 416  
7-1542    -------------------------------------------------------------------AGATTCGACGGACCTCGATTTGGTGGCTCCAGACCAGATGGTGCTGGAGGGAGACCTTTCTTCGGCCAAGGAGGCAGGCGTGGTGATGGAGAAGAAGAAACTGATGCTGCCCAACAAATTGGT 416  
7-1544    -------------------------------------------------------------------AGATTCGACGGACCTCGATTTGGTGGCTCCAGACCAGATGGTGCTGGAGGGAGACCTTTCTTCGGCCAAGGAGGCAGGCGTGGTGATGGAGAAGAAGAAACTGATGCTGCCCAACAAATTGGT 416  
7-1545    -------------------------------------------------------------------AGATTCGACGGACCTCGATTTGGTGGCTCCAGACCAGATGGTGCTGGAGGGAGACCTTTCTTCGGCCAAGGAGGCAGGCGTGGTGATGGAGAAGAAGAAACTGATGCTGCCCAACAAATTGGT 416  
7-1546    -------------------------------------------------------------------AGATTCGACGGACCTCGATTTGGTGGCTCCAGACCAGATGGTGCTGGAGGGAGACCTTTCTTCGGCCAAGGAGGCAGGCGTGGTGATGGAGAAGAAGAAACTGATGCTGCCCAACAAATTGGT 416  
7-1548    -------------------------------------------------------------------AGATTCGACGGACCTCGATTTGGTGGCTCCAGACCAGATGGTGCTGGAGGGAGACCTTTCTTCGGCCAAGGAGGCAGGCGTGGTGATGGAGAAGAAGAAACTGATGCTGCCCAACAAATTGGT 416  
7-1549    -------------------------------------------------------------------AGATTCGACGGACCTCGATTTGGTGGCTCCAGACCAGATGGTGCTGGAGGGAGACCTTTCTTCGGCCGAGGAGGCAGGCGTGGTGATGGAGAAGAAGAAACTGATGCTGCCCAACAAATTGGT 416  
7-1550    -------------------------------------------------------------------AGATTCGACGGACCTCGATTTGGTGGCTCCAGACCAGATGGTGCTGGAGGGAGACCTTTCTTCGGCCAAGGAGGCAGGCGTGGTGATGGAGAAGAAGAAACTGATGCTGCCCAACAAATTGGT 416  
7-1539    -------------------------------------------------------------------AGATTCGACGGACCTCGATTTGGTGGCTCCAGACCAGATGGTGCTGGAGGGAGACCTTTCTTCGGCCAAGGAGGCAGGCGTGGTGATGGAGAAGAAGAAACTGATGCTGCCCAACAAATTGGT 416  
7-1540    -------------------------------------------------------------------AGATTCGACGGACCTCGATTTGGTGGCTCCAGACCAGATGGTGCTGGAGGGAGACCTTTCTTCGGCCAAGGAGGCAGGCGTGGTGATGGAGAAGAAGAAACTGATGCTGCCCAACAAATTGGT 416  
7-1514    -------------------------------------------------------------------AGATTCGACGGACCTCGATTTGGTGGCTCCAGACCAGATGGTGCTGGAGGGAGACCTTTCTTCGGCCAAGGAGGCAGGCGTGGTGATGGAGAAGAAGAAACTGATGCTGCCCAACAAATTGGT 414  
7-1507    -------------------------------------------------------------------AGATTCGACGGACCTCGATTTGGTGGCTCCAGACCAGATGGTGCTGGAGGGAGACCTTTCTTCGGCCAAGGAGGCAGGCGTGGTGATGGAGAAGAAGAAACTGATGCTGCCCAACAAATTGGT 416  
7-1518    -------------------------------------------------------------------AGATTCGACGGACCTCGATTTGGTGGCTCCAGACCAGATGGTGCTGGAGGGAGACCTTTCTTCGGCCAAGGAGGCAGGCGTGGTGATGGAGAAGAAGAAACTGATGCTGCCCAACAAATTGGT 414  
7-1522    -------------------------------------------------------------------AGATTCGACGGACCTCGATTTGGTGGCTCCAGACCAGATGGTGCTGGAGGGAGACCTTTCTTCGGCCAAGGAGGCAGGCGTGGTGATGGAGAAGAAGAAACTGATGCTGCCCAACAAATTGGT 414  
7-1532    -------------------------------------------------------------------AGATTCGACGGACCTCGATTTGGTGGCTCCAGACCAGATGGTGCTGGAGGGAGACCTTTCTTCGGCCAAGGAGGCAGGCGTGGTGATGGAGAAGAAGAAACTGATGCTGCCCAACAAATTGGT 416  
7-1543    -------------------------------------------------------------------AGATTCGACGGACCTCGATTTGGTGGCTCCAGACCAGATGGTGCTGGAGGGAGACCTTTCTTCGGCCAAGGAGGCAGGCGTGGTGATGGAGAAGAAGAAACTGATGCTGCCCAACAAATTGGT 414  
7-1547    -------------------------------------------------------------------AGATTCGACGGACCTCGATTTGGTGGCTCCAGACCAGATGGTGCTGGAGGGAGACCTTTCTTCGGCCAAGGAGGCAGGCGTGGTGATGGAGAAGAAGAAACTGATGCTGCCCAACAAATTGGT 416  
7-2401    -------------------------------------------------------------------AGATTCGACGGACCTCGATTTGGTGGCTCCAGACCAGATGGTGCTGGAGGGAGACCTTTCTTCGGCCAAGGAGGCAGGCGTGGTGATGGAGAAGAAGAAACTGATGCTGCCCAACAAATTGGT 416  
7-2402    -------------------------------------------------------------------AGATTCGACGGACCTCGATTTGGTGGCTCCAGACCAGATGGTGCTGGAGGGAGACCTTTCTTCGGCCAAGGAGGCAGGCGTGGTGATGGAGAAGAAGAAACTGATGCTGCCCAACAAATTGGT 416  
7-2403    -------------------------------------------------------------------AGATTCGACGGACCTCGATTTGGTGGCTCCAGACCAGATGGTGCTGGAGGGAGACCTTTCTTCGGCCAAGGAGGCAGGCGTGGTGATGGAGAAGAAGAAACTGATGCTGCCCAACAAATTGGT 414  
7-2404    -------------------------------------------------------------------AGATTCGACGGACCTCGATTTGGTGGCTCCAGACCAGATGGTGCTGGAGGGAGACCTTTCTTCGGCCAAGGAGGCAGGCGTGGTGATGGAGAAGAAGAAACTGATGCTGCCCAACAAATTGGT 416  
7-2405    -------------------------------------------------------------------AGATTCGACGGACCTCGATTTGGTGGCTCCAGACCAGATGGTGCTGGAGGGAGACCTTTCTTCGGCCAAGGAGGCAGGCGTGGTGATGGAGAAGAAGAAACTGATGCTGCCCAACAAATTGGT 416  
7-2406    -------------------------------------------------------------------AGATTCGACGGACCTCGATTTGGTGGCTCCAGACCAGATGGTGCTGGAGGGAGACCTTTCTTCGGCCAAGGAGGCAGGCGTGGTGATGGAGAAGAAGAAACTGATGCTGCCCAACAAATTGGT 416  
7-2407    -------------------------------------------------------------------AGATTCGACGGACCTCGATTTGGTGGCTCCAGACCAGATGGTGCTGGAGGGAGACCTTTCTTCGGCCAAGGAGGCAGGCGTGGTGATGGAGAAGAAGAAACTGATGCTGCCCAACAAATTGGT 416  
7-2408    -------------------------------------------------------------------AGATTCGACGGACCTCGATTTGGTGGCTCCAGACCAGATGGTGCTGGAGGGAGACCTTTCTTCGGCCAAGGAGGCAGGCGTGGTGATGGAGAAGAAGAAACTGATGCTGCCCAACAAATTGGT 416  
7-2409    -------------------------------------------------------------------AGATTCGACGGACCTCGATTTGGTGGCTCCAGACCAGATGGTGCTGGAGGGAGACCTTTCTTCGGCCAAGGAGGCAGGCGTGGTGATGGAGAAGAAGAAACTGATGCTGCCCAACAAATTGGT 416  
7-2410    -------------------------------------------------------------------AGATTCGACGGACCTCGATTTGGTGGCTCCAGACCAGATGGTGCTGGAGGGAGACCTTTCTTCGGCCAAGGAGGCAGGCGTGGTGATGGAGAAGAAGAAACTGATGCTGCCCAACAAATTGGT 416  
7-2411    -------------------------------------------------------------------AGATTCGACGGACCTCGATTTGGTGGCTCCAGACCAGATGGTGCTGGAGGGAGACCTTTCTTCGGCCAAGGAGGCAGGCGTGGTGATGGAGAAGAAGAAACTGATGCTGCCCAACAAATTGGT 416  
7-2412    -------------------------------------------------------------------AGATTCGACGGACCTCGATTTGGTGGCTCCAGACCAGATGGTGCTGGAGGGAGACCTTTCTTCGGCCAAGGAGGCAGGCGTGGTGATGGAGAAGAAGAAACTGATGCTGCCCAACAAATTGGT 416  
7-2413    -------------------------------------------------------------------AGATTCGACGGACCTCGATTTGGTGGCTCCAGACCAGATGGTGCTGGAGGGAGACCTTTCTTCGGCCAAGGAGGCAGGCGTGGTGATGGAGAAGAAGAAACTGATGCTGCCCAACAAATTGGT 416  
7-2414    -------------------------------------------------------------------AGATTCGACGGACCTCGATTTGGTGGCTCCAGACCAGATGGTGCTGGAGGGAGACCTTTCTTTGGCCAAGGAGGCAGGCGTGGTGATGGAGAAGAAGAAACTGATGCTGCCCAACAAATTGGT 416  
7-2416    -------------------------------------------------------------------AGATTCGACGGACCTCGATTTGGTGGCTCCAGACCAGATGGTGCTGGAGGGAGACCTTTCTTCGGCCAAGGAGGCAGGCGTGGTGATGGAGAAGAAGAAACTGATGCTGCCCAACAAATTGGT 416  
7-2417    -------------------------------------------------------------------AGATTCGACGGACCTCGATTTGGTGGCTCCAGACCAGATGGTGCTGGAGGGAGACCTTTCTTCGGCCAAGGAGGCAGGCGTGGTGATGGAGAAGAAGAAACTGATGCTGCCCAACAAATTGGT 416  
7-2418    -------------------------------------------------------------------AGATTCGACGGACCTCGATTTGGTGGCTCCAGACCAGATGGTGCTGGAGGGAGACCTTTCTTCGGCCAAGGAGGCAGGCGTGGTGATGGAGAAGAAGAAACTGATGCTGCCCAACAAATTGGT 416  
7-2420    -------------------------------------------------------------------AGATTCGACGGACCTCGATTTGGTGGCTCCAGACCAGATGGTGCTGGAGGGAGACCTTTCTTCGGCCAAGGAGGCAGGCGTGGTGATGGAGAAGAAGAAACTGATGCTGCCCAACAAATTGGT 416  
7-2421    -------------------------------------------------------------------AGATTCGACGGACCTCGATTTGGTGGCTCCAGACCAGATGGTGCTGGAGGGAGACCTTTCTTCGGCCAAGGAGGCAGGCGTGGTGATGGAGAAGAAGAAACTGATGCTGCCCAACAAATTGGT 416  
7-2423    -------------------------------------------------------------------AGATTCGACGGACCTCGATTTGGTGGCTCCAGACCAGATGGTGCTGGAGGGAGACCTTTCTTCGGCCAAGGAGGCAGGCGTGGTGATGGAGAAGAAGAAACTGATGCTGCCCAACAAATTGGT 416  
7-2424    -------------------------------------------------------------------AGATTCGACGGACCTCGATTTGGTGGCTCCAGACCAGATGGTGCTGGAGGGAGACCTTTCTTCGGCCAAGGAGGCAGGCGTGGTGATGGAGAAGAAGAAACTGATGCTGCCCAACAAATTGGT 416  
7-2425    -------------------------------------------------------------------AGATTCGACGGACCTCGATTTGGTAGCTCCAGACCAGATGGTGCTGGAGGGAGACCTTTCTTCGGCCAAGGAGGCAGGCGTGGTGATGGAGAAGAAGAAACTGATGCTGCCCAACAAATTGGT 416  
7-2426    -------------------------------------------------------------------AGATTCGACGGACCTCGATTTGGTGGCTCCAGACCAGATGGTGCTGGAGGGAGACCTTTCTTCGGCCAAGGAGGCAGGCGTGGTGATGGAGAAGAAGAAACTGATGCTGCCCAACAAATTGGT 416  
7-2430    -------------------------------------------------------------------AGATTCGACGGACCTCGATTTGGTGGCTCCAGACCAGATGGTGCTGGAGGGAGACCTTTCTTCGGCCAAGGAGGCAGGCGTGGTGATGGAGAAGAAGAAACTGATGCTGCCCAACAAATTGGT 416  
7-2431    -------------------------------------------------------------------AGATTCGACGGACCTCGATTTGGTGGCTCCAGACCAGATGGTGCTGGAGGGAGACCTTTCTTCGGCCAAGGAGGCAGGCGTGGTGATGGAGAAGAAGAAACTGATGCTGCCCAACAAATTGGT 416  
7-2432    -------------------------------------------------------------------AGATTCGACGGACCTCGATTTGGTGGCTCCAGACCAGATGGTGCTGGAGGGAGACCTTTCTTCGGCCAAGGAGGCAGGCGCGGTGATGGAGAAGAAGAAACTGATGCTGCCCAACAAATTGGT 416  
7-2436    -------------------------------------------------------------------AGATTCGACGGACCTCGATTTGGTGGCTCCAGACCAGATGGTGCTGGAGGGAGACCTTTCTTCGGCCGAGGAGGCAGGCGTGGTGATGGAGAAGAAGAAACTGATGCTGCCCAACAAATTGGT 416  
7-2437    -------------------------------------------------------------------AGATTCGACGGACCTCGATTTGGTGGCTCCAGACCAGATGGTGCTGGAGGGAGACCTTTCTTCGGCCAAGGAGGCAGGCGTGGTGATGGAGAAGAAGAAACTGATGCTGCCCAACAAATTGGT 416  
7-2439    -------------------------------------------------------------------AGATTCGACGGACCTCGATTTGGTGGCTCCAGACCAGATGGTGCTGGAGGGAGACCTTTCTTCGGCCAAGGAGGCAGGCGTGGTGATGGAGAAGAAGAAACTGATGCTGCCCAACAAATTGGT 416  
7-2442    -------------------------------------------------------------------AGATTCGACGGACCTCGATTTGGTGGCTCCAGACCAGATGGTGCTGGAGGGAGACCTTTCTTCGGCCAAGGAGGCAGGCGTGGTGATGGAGAAGAAGAAACTGATGCTGCCCAACAAATTGGT 416  
7-2443    -------------------------------------------------------------------AGATTCGACGGACCTCGATTTGGTGGCTCCAGACCAGATGGTGCTGGAGGGAGACCTTTCTTCGGCCGAGGAGGCAGGCGTGGTGATGGAGAAGAAGAAACTGATGCTGCCCAACAAATTGGT 416  
7-2444    -------------------------------------------------------------------AGATTCGACGGACCTCGATTTGGTGGCTCCAGACCAGATGGTGCTGGAGGGAGACCTTTCTTCGGCCAAGGAGGCAGGCGTGGTGATGGAGAAGAAGAAACTGATGCTGCCCAACAAATTGGT 416  
7-2445    -------------------------------------------------------------------AGATTCGACGGACCTCGATTTGGTGGCTCCAGACCAGATGGTGCTGGAGGGAGACCTTTCTTCGGCCAAGGAGGCAGGCGTGGTGATGGAGAAGAAGAAACTGATGCTGCCCAACAAATTGGT 416  
7-2446    -------------------------------------------------------------------AGATTCGACGGACCTCGATTTGGTGGCTCCAGACCAGATGGTGCTGGAGGGAGACCTTTCTTCGGCCAAGGAGGCAGGCGTGGTGATGGAGAAGAAGAAACTGATGCTGCCCAACAAATTGGT 416  
7-2447    -------------------------------------------------------------------AGATTCGACGGACCTCGATTTGGTGGCTCCAGACCAGATGGTGCTGGAGGGAGACCTTTCTTCGGCCAAGGAGGCAGGCGTGGTGATGGAGAAGAAGAAACTGATGCTGCCCAACAAATTGGT 416  
7-2448    -------------------------------------------------------------------AGATTCGACGGACCTCGATTTGGTGGCTCCAGACCAGATGGTGCTGGAGGGAGACCTTTCTTCGGCCAAGGAGGCAGGCGTGGTGATGGAGAAGAAGAAACTGATGCTGCCCAACAAATTGGT 416  
7-2450    -------------------------------------------------------------------AGATTCGACGGACCTCGATTTGGTGGCTCCAGACCAGATGGTGCTGGAGGGAGACCTTTCTTCGGCCAAGGAGGCAGGCGTGGTGATGGAGAAGAAGAAACTGATGCTGCCCAACAAATTGGT 416  
7-2415    -------------------------------------------------------------------AGATTCGACGGACCTCGATTTGGTGGCTCCAGACCAGATGGTGCTGGAGGGAGACCTTTCTTCGGCCAAGGAGGCAGGCGTGGTGATGGAGAAGAAGAAACTGATGCTGCCCAACAAATTGGT 416  
7-2427    -------------------------------------------------------------------AGATTCGACGGACCTCGATTTGGTGGCTCCAGACCAGATGGTGCTGGAGGGAGACCTTTCTTCGGCCAAGGAGGCAGGCGTGGTGATGGAGAAGAAGAAACTGATGCTGCCCAACAAATTGGT 416  
7-2428    -------------------------------------------------------------------AGATTCGACGGACCTCGATTTGGTGGCTCCAGACCAGATGGTGCTGGAGGGAGACCTTTCTTCGGCCAAGGAGGCAGGCGTGGTGATGGAGAAGAAGAAACTGATGCTGCCCAACAAATTGGT 414  
7-2435    -------------------------------------------------------------------AGATTCGACGGACCTCGATTTGGTGGCTCCAGACCAGATGGTGCTGGAGGGAGACCTTTCTTCGGCCAAGGAGGCAGGCGTGGTGATGGAGAAGAAGAAACTGATGCTGCCCAACAAATTGGT 414  
7-2440    -------------------------------------------------------------------AGATTCGACGGACCTCAATTTGGTGGCTCCAGACCAGATGGTGCTGGAGGGAGACCTTTCTTCGGCCAAGGAGGCAGGCGTGGTGATGGAGAAGAAGAAACTGATGCTGCCCAACAAATTGGT 416  
7-2441    -------------------------------------------------------------------AGGTTCGACGGACCTCGATTTGGTGGCTCCAGACCAGATGGTGCTGGAGGGAGACCTTTCTTCGGCCAAGGAGGCAGGCGTGGTGATGGAGAAGAAGAAACTGATGCTGCCCAACAAATTGGT 414  
7-2449    -------------------------------------------------------------------AGATTCGACGGACCTCGATTTGGTGGCTCCAGACCAGATGGTGCTGGAGGGAGACCTTTCTTCGGCCAAGGAGGCAGGCGTGGTGATGGAGAAGAAGAAACTGATGCTGCCCAACAAATTGGT 414  
8-1501    -------------------------------------------------------------------AGATTCGACGGACCTCGATTTGGTGGCTCCAGACCAGATGGTGCTGGAGGGAGACCTTTCTTCGGCCAAGGAGGCAGGCGTGGTGATGGAGAAGAAGAAACTGATGCTGCCCAACAAATTGGT 416  
8-1502    -------------------------------------------------------------------AGATTCGACGGACCTCGATTTGGTGGCTCCAGACCAGATGGTGCTGGAGGGAGACCTTTCTTCGGCCAAGGAGGCAGGCGTGGTGATGGAGAAGAAGAAACTGATGCTGCCCAACAAATTGGT 416  
8-1503    -------------------------------------------------------------------AGATTCGACGGACCTCGATTTGGTGGCTCCAGACCAGATGGTGCTGGAGGGAGACCTTTCTTCGGCCAAGGAGGCAGGCGTGGTGATGGAGAAGAAGAAACTGATGCTGCCCAACAAATTGGT 415  
8-1504    -------------------------------------------------------------------AGATTCGACGGACCTCGATTTGGTGGCTCCAGACCAGATGGTGCTGGAGGGAGACCTTTCTTCGGCCAAGGAGGCAGGCGTGGTGATGGAGAAGAAGAAACTGATGCTGCCCAACAAATTGGT 416  
8-1505    -------------------------------------------------------------------AGATTCGACGGACCTCGATTTGGTGGCTCCAGACCAGATGGTGCTGGAGGGAGACCTTTCTTCGGCCAAGGAGGCAGGCGTGGTGATGGAGAAGAAGAAACTGATGCTGCCCAACAAATTGGT 416  
8-1506    -------------------------------------------------------------------AGATTCGACGGACCTCGATTTGGTGGCTCCAGACCAGATGGTGCTGGAGGGAGACCTTTCTTCGGCCAAGGAGGCAGGCGTGGTGATGGAGAAGAAGAAACTGATGCTGCCCAACAAATTGGT 416  
8-1507    -------------------------------------------------------------------AGATTCGACGGACCTCGATTTGGTGGCTCCAGACCAGATGGTGCTGGAGGGAGACCTTTCTTCGGCCAAGGAGGCAGGCGTGGTGATGGAGAAGAAGAAACTGATGCTGCCCAACAAATTGGT 416  
8-1508    -------------------------------------------------------------------AGATTCGACGGACCTCGATTTGGTGGCTCCAGACCAGATGGTGCTGGAGGGAGACCTTTCTTCGGCCAAGGAGGCAGGCGTGGTGATGGAGAAGAAGAAACTGATGCTGCCCAACAAATTGGT 416  
8-1509    -------------------------------------------------------------------AGATTCGACGGACCTCGATTTGGTGGCTCCAGACCAGATGGTGCTGGAGGGAGACCTTTCTTCGGCCAAGGAGGCAGGCGTGGTGATGGAGAAGAAGAAACTGATGCTGCCCAACAAATTGGT 416  
8-1510    -------------------------------------------------------------------AGATTCGACGGACCTCGATTTGGTGGCTCCAGACCAGATGGTGCTGGAGGGAGACCTTTCTTCGGCCAAGGAGGCAGGCGTGGTGATGGAGAAGAAGAAACTGATGCTGCCCAACAAATTGGT 416  
8-1511    -------------------------------------------------------------------AGATTCGACGGACCTCGATTTGGTGGCTCCAGACCAGATGGTGCTGGAGGGAGACCTTTCTTCGGCCAAGGAGGCAGGCGTGGTGATGGAGAAGAAGAAACTGATGCTGCCCAACAAATTGGT 416  
8-1512    -------------------------------------------------------------------AGATTCGACGGACCTCGATTTGGTGGCTCCAGACCAGATGGTGCTGGAGGGAGACCTTTCTTCGGCCAAGGAGGCAGGCGTGGTGATGGAGAAGAAGAAACTGATGCTGCCCAACAAATTGGT 416  
8-1513    -------------------------------------------------------------------AGATTCGACGGACCTCGATTTGGTGGCTCCAGACCAGATGGTGCTGGAGGGAGACCTTTCTTCGGCCAAGGAGGCAGGCGTGGTGATGGAGAAGAAGAAACTGATGCTGCCCAACAAATTGGT 416  
8-1514    -------------------------------------------------------------------AGATTCGACGGACCTCGATTTGGTGGCTCCAGACCAGATGGTGCTGGAGGGAGACCTTTCTTCGGCCAAGGAGGCAGGCGTGGTGATGGAGAAGAAGAAACTGATGCTGCCCAACAAATTGGT 416  
8-1515    -------------------------------------------------------------------AGATTCGACGGACCTCGATTTGGTGGCTCCAGACCAGATGGTGCTGGAGGGAGACCTTTCTTCGGCCAAGGAGGCAGGCGTGGTGATGGAGAAGAAGAAACTGATGCTGCCCAACAAATTGGT 416  
8-1516    -------------------------------------------------------------------AGATTCGACGGACCTCGATTTGGTGGCTCCAGACCAGATGGTGCTGGAGGGAGACCTTTCTTCGGCCAAGGAGGCAGGCGTGGTGATGGAGAAGAAGAAACTGATGCTGCCCAACAAATTGGT 416  
8-1517    -------------------------------------------------------------------AGATTCGACGGACCTCGATTTGGTGGCTCCAGACCAGATGGTGCTGGAGGGAGACCTTTCTTCGGCCAAGGAGGCAGGCGTGGTGATGGAGAAGAAGAAACTGATGCTGCCCAACAAATTGGT 416  
8-1518    -------------------------------------------------------------------AGATTCGACGGACCTCGATTTGGTGGCTCCAGACCAGATGGTGCTGGAGGGAGACCTTTCTTCGGCCAAGGAGGCAGGCGTGGTGATGGAGAAGAAGGAACTGATGCTGCCCAACAAATTGGT 416  
8-1519    -------------------------------------------------------------------AGATTCGACGGACCTCGATTTGGTGGCTCCAGACCAGATGGTGCTGGAGGGAGACCTTTCTTCGGCCAAGGAGGCAGGCGTGGTGATGGAGAAGAAGAAACTGATGCTGCCCAACAAATTGGT 416  
8-1520    -------------------------------------------------------------------AGATTCGACGGACCTCGATTTGGTGGCTCCAGACCAGATGGTGCTGGAGGGAGACCTTTCTTCGGCCAAGGAGGCAGGCGTGGTGATGGAGAAGAAGAAACTGATGCTGCCCAACAAATTGGT 416  
8-1521    -------------------------------------------------------------------AGATTCGACGGACCTCGATTTGGTGGCTCCAGACCAGATGGTGCTGGAGGGAGACCTTTCTTCGGCCAAGGAGGCAGGCGTGGTGATGGAGAAGAAGAAACAGATGCTGCCCAACAAATTGGT 416  
8-1522    -------------------------------------------------------------------AGATTCGACGGACCTCGATTTGGTGGCTCCAGACCAGATGGTGCTGGAGGGAGACCTTTCTTCGGCCAAGGAGGCAGGCGTGGTGATGGAGAAGAAGAAACTGATGCTGCCCAACAAATTGGT 416  
8-1524    -------------------------------------------------------------------AGATTCGACGGACCTCGATTTGGTGGCTCCAGACCAGATGGTGCTGGAGGGAGACCTTTCTTCGGCCAAGGAGGCAGGCGTGGTGATGGAGAAGAAGAAACTGATGCTGCCCAACAAATTGGT 416  
8-1525    -------------------------------------------------------------------AGATTCGACGGACCTCGATTTGGTGGCTCCAGACCAGATGGTGCTGGAGGGAGACCTTTCTTCGGCCAAGGAGGCAGGCGTGGTGATGGAGAAGAAGAAACTGATGCTGCCCAACAAATTGGT 416  
8-1526    -------------------------------------------------------------------AGATTCGACGGACCTCGATTTGGTGGCTCCAGACCAGATGGTGCTGGAGGGAGACCTTTCTTCGGCCAAGGAGGCAGGCGTGGTGATGGAGAAGAAGAAACTGATGCTGCCCAACAAATTGGT 416  
8-1527    -------------------------------------------------------------------AGATTCGACGGACCTCGATTTGGTGGCTCCAGACCAGATGGTGCTGGAGGGAGACCTTTCTTCGGCCAAGGAGGCAGGCGTGGTGATGGAGAAGAAGAAACTGATGCTGCCCAACAAATTGGT 416  
8-1528    -------------------------------------------------------------------AGATTCGACGGACCTCGATTTGGTGGCTCCAGACCAGATGGTGCTGGAGGGAGACCTTTCTTCGGCCAAGGAGGCAGGCGTGGTGATGGAGAAGAAGAAACTGATGCTGCCCAACAAATTGGT 416  
8-1529    -------------------------------------------------------------------AGATTCGACGGACCTCGATTTGGTGGCTCCAGACCAGATGGTGCTGGAGGGAGACCTTTCTTCGGCCAAGGAGGCAGGCGTGGTGATGGAGAAGAAGAAACTGATGCTGCCCAACAAATTGGT 416  
8-1530    -------------------------------------------------------------------AGATTCGACGGACCTCGATTTGGTGGCTCCAGACCAGATGGTGCTGGAGGGAGACCTTTCTTCGGCCAAGGAGGCAGGCGTGGTGATGGAGAAGAAGAAACTGATGCTGCCCAACAAATTGGT 416  
8-1531    -------------------------------------------------------------------AGATTCGACGGACCTCGATTTGGTGGCTCCAGACCAGATGGTGCTGGAGGGAGACCTTTCTTCGGCCAAGGAGGCAGGCGTGGTGATGGAGAAGAAGAAACTGATGCTGCCCAACAAATTGGT 416  
8-1532    -------------------------------------------------------------------AGATTCGACGGACCTCGATTTGGTGGCTCCAGACCAGATGGTGCTGGAGGGAGACCTTTCTTCGGCCAAGGAGGCAGGCGTGGTGATGGAGAAGAAGAAACTGATGCTGCCCAACAAATTGGT 416  
8-1533    -------------------------------------------------------------------AGATTCGACGGACCTCGATTTGGTGGCTCCAGACCAGATGGTGCTGGAGGGAGACCTTTCTTCGGCCAAGGAGGCAGGCGTGGTGATGGAGAAGAAGAAACTGATGCTGCCCAACAAATTGGT 416  
8-1534    -------------------------------------------------------------------AGATTCGACGGACCTCGATTTGGTGGCTCCAGACCAGATGGTGCTGGAGGGAGACCTTTCTTCGGCCAAGGAGGCAGGCGTGGTGATGGAGAAGAAGAAACTGATGCTGCCCAACAAATTGGT 416  
8-1535    -------------------------------------------------------------------AGATTCGACGGACCTCGATTTGGTGGCTCCAGACCAGATGGTGCTGGAGGGAGACCTTTCTTCGGCCAAGGAGGCAGGCGTGGTGATGGAGAAGAAGAAACTGATGCTGCCCAACAAATTGGT 416  
8-1536    -------------------------------------------------------------------AGATTCGACGGACCTCGATTTGGTGGCTCCAGACCAGATGGTGCTGGAGGGAGACCTTTCTTCGGCCAAGGAGGCAGGCGTGGTGATGGAGAAGAAGAAACTGATGCTGCCCAACAAATTGGT 416  
8-1537    -------------------------------------------------------------------AGATTCGACGGACCTCGATTTGGTGGCTCCAGACCAGATGGTGCTGGAGGGAGACCTTTCTTCGGCCAAGGAGGCAGGCGTGGTGATGGAGAAGAAGAAACTGATGCTGCCCAACAAATTGGT 416  
8-1538    -------------------------------------------------------------------AGATTCGACGGACCTCGATTTGGTGGCTCCAGACCAGATGGTGCTGGAGGGAGACCTTTCTTCGGCCAAGGAGGCAGGCGTGGTGATGGAGAAGAAGAAACTGATGCTGCCCAACAAATTGGT 416  
8-1539    -------------------------------------------------------------------AGATTCGACGGACCTCGATTTGGTGGCTCCGGACCAGATGGTGCTGGAGGGAGACCTTTCTTCGGCCAAGGAGGCAGGCGTGGTGATGGAGAAGAAGAAACTGATGCTGCCCAACAAATTGGT 416  
8-1540    -------------------------------------------------------------------AGATTCGACGGACCTCGATTTGGTGGCTCCAGACCAGATGGTGCTGGAGGGAGACCTTTCTTCGGCCAAGGAGGCAGGCGCGGTGATGGAGAAGAAGAAACTGATGCTGCCCAACAAATTGGT 416  
8-1542    -------------------------------------------------------------------AGATTCGACGGACCTCGATTTGGTGGCTCCAGACCAGATGGTGCTGGAGGGAGACCTTTCTTCGGCCAAGGAGGCAGGCGTGGTGATGGAGAAGAAGAAACTGATGCTGTCCAACAAATTGGT 416  
8-1546    -------------------------------------------------------------------AGATTCGACGGACCTCGATTTGGTGGCTCCAGACCAGATGGTGCTGGAGGGAGACCTTTCTTCGGCCAAGGAGGCAGGCGTGGTGATGGAGAAGAAGAAACTGATGCTGCCCAACAAATTGGT 416  
8-1548    -------------------------------------------------------------------AGATTCGACGGACCTCGATTTGGTGGCTCCAGACCAGATGGTGCTGGAGGGAGACCTTTCTTCGGCCAAGGAGGCAGGCGTGGTGATGGAGAAGAAGAAACTGATGCTGCCCAACAAATTGGT 416  
8-1549    -------------------------------------------------------------------AGATTCGACGGACCTCGATTTGGTGGCTCCAGACCAGATGGTGCTGGAGGGAGACCTTTCTTCGGCCAAGGAGGCAGGCGTGGTGATGGAGAAGAAGAAACTGATGCTGCCCAACAAATTGGT 416  
8-1550    -------------------------------------------------------------------AGATTCGACGGACCTCGATTTGGTGGCTCCAGACCAGATGGTGCTGGAGGGAGACCTTTCTTCGGCCAAGGAGGCAGGCGTGGTGATGGAGAAGAAGAAACTGATGCTGCCCAACAAATTGGT 414  
8-1541    -------------------------------------------------------------------AGATTCGACGGACCTCGATTTGGTGGCTCCAGACCAGATGGTGCTGGAGGGAGACCTTTCTTCGGCCAAGGAGGCAGGCGTGGTGATGGAGAAGAAGAAACTGATGCTGCCCAACAAATTGGT 416  
8-1543    -------------------------------------------------------------------AGATTCGACGGACCTCGATTTGGTGGCTCCAGACCAGATGGTGCTGGAGGGAGACCTTTCTTCGGCCAAGGAGGCAGGCGTGGTGATGGAGAAGAAGAAACTGATGCTGCCCAACAAATTGGT 416  
8-2401    -------------------------------------------------------------------AGATTCGACGGACCTCGATTTGGTGGCTCCAGACCAGATGGTGCTGGAGGGAGACCTTTCTTCGGCCAAGGAGGCAGGCGTGGTGATGGAGAAGAAGAAACTGATGCTGCCCAACAAATTGGT 416  
8-2406    -------------------------------------------------------------------AGATTCGACGGACCTCGATTTGGTGGCTCCAGACCAGATGGTGCTGGAGGGAGACCTTTCTTCGGCCAAGGAGGCAGGCGTGGTGATGGAGAAGAAGAAACTGATGCTGCCCAACAAATTGGT 416  
8-2407    -------------------------------------------------------------------AGATTCGACGGACCTCGATTTGGTGGCTCCAGACCAGATGGTGCTGGAGGGAGACCTTTCTTCGGCCAAGGAGGCAGGCGTGGTGATGGAGAAGAAGAAACTGATGCTGCCCAACAAATTGGT 416  
8-2409    -------------------------------------------------------------------AGATTCGACGGACCTCGATTTGGTGGCTCCAGACCAGATGGTGCTGGAGGGAGACCTTTCTTCGGCCAAGGAGGCAGGCGTGGTGACGGAGAAGAAGAAACTGATGCTGCCCAACAAATTGGT 416  
8-2410    -------------------------------------------------------------------AGATTCGACGGACCTCGATTTGGTGGCTCCAGACCAGATGGTGCTGGAGGGAGACCTTTCTTCGGCCAAGGAGGCAGGCGTGGTGATGGAGAAGAAGAAACTGATGCTGCCCAACAAATTGGT 416  
8-2411    -------------------------------------------------------------------AGATTCGACGGACCTCGATTTGGTGGCTCCAGACCAGATGGTGCTGGAGGGAGACCTTTCTTCGGCCAAGGAGGCAGGCGTGGTGATGGAGAAGAAGAAACTGATGCTGCCCAACAAATTGGT 416  
8-2414    -------------------------------------------------------------------AGATTCGACGGACCTCGATTTGGTGGCTCCAGACCAGATGGTGCTGGAGGGAGACCTTTCTTCGGCCAAGGAGGCAGGCGTGGTGATGGAGAAGAAGAAACTGATGCTGCCCAACAAATTGGT 416  
8-2417    -------------------------------------------------------------------AGATTCGACGGACCTCGATTTGGTGGCTCCAGACCAGATGGTGCTGGAGGGAGACCTTTCTTCGGCCAAGGAGGCAGGCGTGGTGATGGAGAAGAAGAAACTGATGCTGCCCAACAAATTGGT 416  
8-2420    -------------------------------------------------------------------AGATTCGACGGACCTCGATTTGGTGGCTCCAGACCAGATGGTGCTGGAGGGAGACCTTTCTTCGGCCAAGGAGGCAGGCGTGGTGATGGAGAAGAAGAAACTGATGCTGCCCAACAAATTGGT 416  
8-2421    -------------------------------------------------------------------AGATTCGACGGACCTCGATTTGGTGGCTCCAGACCAGATGGTGCTGGAGGGAGACCTTTCTTCGGCCAAGGAGGCAGGCGTGGTGATGGAGAAGAAGAAACTGATGCTGCCCAACAAATTGGT 416  
8-2422    -------------------------------------------------------------------AGATTCGACGGACCTCGATTTGGTGGCTCCAGACCAGATGGTGCTGGAGGGAGACCTTTCTTCGGCCAAGGAGGCAGGCGTGGTGATGGAGAAGAAGAAACTGATGCTGCCCAACAAATTGGT 416  
8-2424    -------------------------------------------------------------------AGATTCGACGGACCTCGATTTGGTGGCTCCAGACCAGATGGTGCTGGAGGGAGACCTTTCTTCGGCCAAGGAGGCAGGCGTGGTGATGGAGAAGAAGAAACTGATGCTGCCCAACAAATTGGT 416  
8-2425    -------------------------------------------------------------------AGATTCGACGGACCTCGATTTGGTGGCTCCAGACCAGATGGTGCTGGAGGGAGACCTTTCTTCGGCCAAGGAGGCAGGCGTGGTGATGGAGAAGAAGAAACTGATGCTGCCCAACAAATTGGT 416  
8-2427    -------------------------------------------------------------------AGATTCGACGGACCTCGATTTGGTGGCTCCAGACCAGATGGTGCTGGAGGGAGACCTTTCTTCGGCCAAGGAGGCAGGCGTGGTGATGGAGAAGAAGAAACTGATGCTGCCCAACAAATTGGT 416  
8-2433    -------------------------------------------------------------------AGATTCGACGGACCTCGATTTGGTGGCTCCAGACCAGATGGTGCTGGAGGGAGACCTTTCTTCGGCCAAGGAGGCAGGCGTGGTGGTGGAGAAGAAGAAACTGATGCTGCCCAACAAATTGGT 416  
8-2434    -------------------------------------------------------------------AGATTCGACGGACCTCGATTTGGTGGCTCCAGACCAGATGGTGCTGGAGGGAGACCTTTCTTCGGCCAAGGAGGCAGGCGTGGTGATGGAGAAGAGGAAACTGATGCTGCCCAACAAATTGGT 416  
8-2435    -------------------------------------------------------------------AGATTCGACGGACCTCGATTTGGTGGCTCCAGACCAGATGGTGCTGGAGGGAGACCTTTCTTCGGCCAAGGAGGCAGGCGTGGTGATGGAGAAGAAGAAACTGATGCTGCCCAACAAATTGGT 416  
8-2436    -------------------------------------------------------------------AGATTCGACGGACCTCGATTTGGTGGCTCCAGACCAGATGGTGCTGGAGGGAGACCTTTCTTCGGCCAAGGAGGCAGGCGTGGTGATGGAGAAGAAGAAACTGATGCTGCCCAACAAATTGGT 416  
8-2437    -------------------------------------------------------------------AGATTCGACGGACCTCGATTTGGTGGCTCCAGACCAGATGGTGCTGGAGGGAGACCTTTCTTCGGCCAAGGAGGCAGGCGTGGTGATGGAGAAGAAGAAACTGATGCTGCCCAACAAATTGGT 416  
8-2438    -------------------------------------------------------------------AGATTCGACGGACCTCGATTTGGTGGCTCCAGACCAGATGGTGCTGGAGGGAGACCTTTCTTCGGCCAAGGAGGCAGGCGTGGTGATGGAGAAGAAGAAACTGATGCTGCCCAACAAATTGGT 416  
8-2439    -------------------------------------------------------------------AGATTCGACGGACCTCGATTTGGTGGCTCCAGACCAGATGGTGCTGGAGGGAGACCTTTCTTCGGCCAAGGAGGCAGGCGTGGTGATGGAGAGGAAGAAACTGATGCTGCCCAACAAATTGGT 416  
8-2440    -------------------------------------------------------------------AGATTCGACGGACCTCGATTTGGTGGCTCCAGACCAGATGGTGCTGGAGGGAGACCTTTCTTCGGCCAAGGAGGCAGGCGTGGTGATGGAGAAGAAGAAACTGATGCTGCCCAACAAATTGGT 416  
8-2442    -------------------------------------------------------------------AGATTCGACGGACCTCGATTTGGTGGCTCCAGACCAGATGGTGCTGGAGGGAGACCTTTCTTCGGCCAAGGAGGCAGGCGTGGTGATGGAGAAGAAGAAACTGATGCTGCCCAACAAATTGGT 416  
8-2444    -------------------------------------------------------------------AGATTCGACGGACCTCGATTTGGTGGCTCCAGACCAGATGGTGCTGGAGGGAGACCTTTCTTCGGCCAAGGAGGCAGGCGTGGTGATGGAGAAGAAGAAACTGATGCTGCCCAACAAATTGGT 416  
8-2445    -------------------------------------------------------------------AGATTCGACGGACCTCGATTTGGTGGCTCCAGACCAGATGGTGCTGGAGGGAGACCTTTCTTCGGCCAAGGAGGCAGGCGTGGTGATGGAGAAGAAGAAACTGATGCTGCCCAACAAATTGGT 416  
8-2448    -------------------------------------------------------------------AGATTCGACGGACCTCGATTTGGTGGCTCCAGACCAGATGGTGCTGGAGGGAGACCTTTCTTCGGCCAAGGAGGCAGGCGTGGTGATGGAGAAGAAGAAACTGATGCTGCCCAACAAATTGGT 416  
8-2449    -------------------------------------------------------------------AGATTCGACGGACCTCGATTTGGTGGCTCCAGACCAGATGGTGCTGGAGGGAGACCTTTCTTCGGCCAAGGAGGCAGGCGTGGTGATGGAGAAGAAGAAACTGATGCTGCCCAACAAATTGGT 416  
8-2450    -------------------------------------------------------------------AGATTCGACGGACCTCGATTTGGTGGCTCCAGACCAGATGGTGCTGGAGGGAGACCTTTCTTCGGCCAAGGAGGCAGGCGTGGTGATGGAGAAGAAGAAACTGATGCTGCCCAACAAATTGGT 416  
8-2432    TGGACCTGGATTTGGTGCCCCGGAGATGGATGGACGGAGACAAAATGGCGGTCCGATGGGTGGAAGGAGATTCGACGGACCTGGATTTGGTGGCTCCAGACCAGATGGTGCTGGAGGAAGACCTTTCTTCGGCCAAGGAGGCAGGCGTGGTGATGGAGAAGAAGAAACTGATGCTGCCCAACAAATGGGT 488  
8-2405    TGGACCTGGATTTGGTGCCCCGGAGATGGATGGACGGAGACAAAATGGCGGTCCGATGGGTGGAAGGAGATTCGACGGACCTGGATTTGGTGGCTCCAGACCAGATGGTGCTGGAGGAAGACCTTTCTTCGGCCAAGGAGGCAGGCGTGGTGATGGAGAAGAAGAAACTGATGCTGCCCAACAAATTGGT 488  
8-2443    -------------------------------------------------------------------AGATTCGACGGACCTCGATTTGGTGGCTCCAGACCAGATGGTGCTGGAGGGAGACCTTTCTTCGGCCAAGGAGGCAGGCGTGGTGATGGAGAAGAAGAAACTGATGCTGCCCAACAAATTGGT 416  
8-2413    -------------------------------------------------------------------AGATTCGACGGACCTCGATTTGGTGGCTCCAGACCAGATGGTGCTGGAGGGAGACCTTTCTTCGGCCAAGGAGGCAGGCGTGGTGATGGAGAAGAAGAAACTGATGCTGCCCAACAAATTGGT 416  
8-2418    -------------------------------------------------------------------AGATTCGACGGACCTCGATTTGGTGGCTCCAGACCAGATGGTGCTGGAGGGAGACCTTTCTTCGGCCAAGGAGGCAGGCGTGGTGATGGAGAAGAAGAAACTGATGCTGCCCAACAAATTGGT 416  
8-2419    -------------------------------------------------------------------AGATTCGACGGACCTCGATTTGGTGGCTCCAGACCAGATGGTGCTGGAGGGAGACCTTTCTTCGGCCAAGGAGGCAGGCGTGGTGATGGAGAAGAAGAAACTGATGCTGCCCAACAAATTGGT 416  
8-2423    -------------------------------------------------------------------AGATTCGACGGACCTCGATTTGGTGGCTCCAGACCAGATGGTGCTGGAGGGAGACCTTTCTTCGGCCAAGGAGGCAGGCGTGGTGATGGAGAAGAAGAAACTGATGCTGCCCAACAAATTGGT 416  
8-2430    -------------------------------------------------------------------AGATTCGACGGACCTCGATTTGGTGGCTCCAGACCAGATGGTGCTGGAGGGAGACCTTTCTTCGGCCAAGGAGGCAGGCGTGGTGATGGAGAAGAAGAAACTGATGCTGCCCAACAAATTGGT 414  
8-2446    -------------------------------------------------------------------AGATTCGACGGACCTCGATTTGGTGGCTCCAGACCAGATGGTGCTGGAGGGAGACCTTTCTTCGGCCAAGGAGGCAGGCGTGGTGATGGAGAAGAAGAAACTGATGCTGCCCAACAAATTGGT 416  
8-2447    -------------------------------------------------------------------AGATTCGACGGACCTCGATTTGGTGGCTCCAGACCAGATGGTGCTGGAGGGAGACCTTTCTTCGGCCAAGGAGGCAGGTGTGGTGATGGAGAAGAAGAAACTGATGCTGCCCAACAAATTGGT 416  
8-2441    -------------------------------------------------------------------AGATTCGACGGACCTCGATTTGGTGGCTCCAGACCAGATGGTACCGGAGGAAGACCTTTCTTCGGCCAAGGAGGAAGACGTGGTGATGGAGAAGAAGAAACTGATGCTGCCCAACAAATTGGT 413  
8-2404    -------------------------------------------------------------------AGATTCGACGGACCTCGATTTGGTGGCTCCAGACCAGATGGTGCTGGAGGGAGACCTTTCTTCGGCCAAGGAGGCAGGCGTGGTGATGGAGAAGAAGAAACTGATGCTGCCCAACAAATTGGT 414  
8-2415    -------------------------------------------------------------------AGATTCGACGGACCTCGATTTGGTGGCTCCAGACCAGATGGTGCTGGAGGGAGACCTTTCTTCGGCCAAGGAGGCAGGCGTGGTGATGGAGAAGAAGAAACTGATGCTGCCCAACAAATTGGT 416  
2-1517    -------------------------------------------------------------------AGATTCGACGGACCTCGATTTGGTGGCTCCAGACCAGATGGTGCTGGAGGGAGACCTTTCTTCGGCCAAGGAGGCAGGCGTGGTGATGGAGAAGAAGAAACTGATGCTGCCCAACAAATTGGT 416  
2-1501    -------------------------------------------------------------------AGATTCGACGGACCTCGATTTGGTGGCTCCAGACCAGATGGTGCTGGAGGGAGACCTTTCTTCGGCCAAGGAGGCAGGCGTGGTGATGGAGAAGAAGAAACTGATGCTGCCCAACAAATTGGT 416  
2-1503    -------------------------------------------------------------------AGATTCGACGGACCTCGATTTGGTGGCTCCAGACCAGATGGTGCTGGAGGGAGACCTTTCTTCGGCCAAGGAGGCAGGCGTGGTGATGGAGAAGAAGAAACTGATGCTGCCCAACAAATTGGT 416  
2-1505    -------------------------------------------------------------------AGATTCGACGGACCTCGATTTGGTGGCTCCAGACCAGATGGTGCTGGAGGGAGACCTTTCTTCGGCCAAGGAGGCAGGCGTGGTGATGGAGAAGAAGAAACTGATGCTGCCCAACAAATTGGT 416  
2-1508    -------------------------------------------------------------------AGATTCGACGGACCTCGATTTGGTGGCTCCAGACCAGATGGTGCTGGAGGGAGACCTTTCTTCGGCCAAGGAGGCAGGCGTGGTGATGGAGAAGAAGAAACTGATGCTGCCCAACAAATTGGT 416  
2-1509    -------------------------------------------------------------------AGATTCGACGGACCTCGATTTGGTGGCTCCAGACCAGATGGTGCTGGAGGGAGACCTTTCTTCGGCCAAGGAGGCAGGCGTGGTGATGGAGAAGAAGAAACTGATGCTGCCCAACAAATTGGT 416  
2-1510    -------------------------------------------------------------------AGATTCGACGGACCTCGATTTGGTGGCTCCAGACCAGATGGTGCTGGAGGGAGACCTTTCTTCGGCCAAGGAGGCAGGCGTGGTGATGGAGAAGAAGAAACTGATGCTGCCCAACAAATTGGT 416  
2-1519    -------------------------------------------------------------------AGATTCGACGGACCTCGATTTGGTGGCTCCAGACCAGATGGTGCTGGAGGGAGACCTTTCTTCGGCCAAGGAGGCAGGCGTGGTGATGGAGAAGAAGAAACTGATGCTGCCCAACAAATTGGT 416  
2-1521    -------------------------------------------------------------------AGATTCGACGGACCTCGATTTGGTGGCTCCAGACCAGATGGTGCTGGAGGGAGACCTTTCTTCGGCCAAGGAGGCAGGCGTGGTGATGGAGAAGAAGAAACTGATGCTGCCCAACAAATTGGT 416  
2-1522    -------------------------------------------------------------------AGATTCGACGGACCTCGATTTGGTGGCTCCAGACCAGATGGTGCTGGAGGGAGACCTTTCTTCGGCCAAGGAGGCAGGCGTGGTGATGGAGAAGAAGAAACTGATGCTGCCCAACAAATTGGT 416  
2-1523    -------------------------------------------------------------------AGATTCGACGGACCTCGATTTGGTGGCTCCAGACCAGATGGTGCTGGAGGGAGACCTTTCTTCGGCCAAGGAGGCAGGCGTGGTGATGGAGAAGAAGAAACTGATGCTGCCCAACAAATTGGT 416  
2-1527    -------------------------------------------------------------------AGATTCGACGGACCTCGATTTGGTGGCTCCAGACCAGATGGTGCTGGAGGGAGACCTTTCTTCGGCCAAGGAGGCAGGCGTGGTGATGGAGAAGAAGAAACTGATGCTGCCCAACAAATTGGT 416  
2-1533    -------------------------------------------------------------------AGATTCGACGGACCTCGATTTGGTGGCTCCAGACCAGATGGTGCTGGAGGGAGACCTTTCTTCGGCCAAGGAGGCAGGCGTGGTGATGGAGAAGAAGAAACTGATGCTGCCCAACAAATTGGT 416  
2-1535    -------------------------------------------------------------------AGATTCGACGGACCTCGATTTGGTGGCTCCAGACCAGATGGTGCTGGAGGGAGACCTTTCTTCGGCCAAGGAGGCAGGCGTGGTGATGGAGAAGAAGAAACTGATGCTGCCCAACAAATTGGT 416  
2-1536    -------------------------------------------------------------------AGATTCGACGGACCTCGATTTGGTGGCTCCAGACCAGATGGTGCTGGAGGGAGACCTTTCTTCGGCCAAGGAGGCAGGCGTGGTGATGGAGAAGAAGAAACTGATGCTGCCCAACAAATTGGT 416  
2-1537    -------------------------------------------------------------------AGATTCGACGGACCTCGATTTGGTGGCTCCAGACCAGATGGTGCTGGAGGGAGACCTTTCTTCGGCCAAGGAGGCAGGCGTGGTGATGGAGAAGAAGAAACTGATGCTGCCCAACAAATTGGT 415  
2-1538    -------------------------------------------------------------------AGATTCGACGGACCTCGATTTGGTGGCTCCAGACCAGATGGTGCTGGAGGGAGACCTTTCTTCGGCCAAGGAGGCAGGCGTGGTGATGGAGAAGAAGAAACTGATGCTGCCCAACAAATTGGT 416  
2-1539    -------------------------------------------------------------------AGATTCGACGGACCTCGATTTGGTGGCTCCAGACCAGATGGTGCTGGAGGGAGACCTTTCTTCGGCCAAGGAGGCAGGCGTGGTGATGGAGAAGAAGAAACTGATGCTGCCCAACAAATTGGT 416  
2-1543    -------------------------------------------------------------------AGATTCGACGGACCTCGATTTGGTGGCTCCAGACCAGATGGTGCTGGAGGGAGACCTTTCTTCGGCCAAGGAGGCAGGCGTGGTGATGGAGAAGAAGAAACTGATGCTGCCCAACAAATTGGT 416  
2-1546    -------------------------------------------------------------------AGATTCGACGGACCTCGATTTGGTGGCTCCAGACCAGATGGTGCTGGAGGGAGACCTTTCTTCGGCCAAGGAGGCAGGCGTGGTGATGGAGAAGAAGAAACTGATGCTGCCCAACAAATTGGT 416  
2-1547    -------------------------------------------------------------------AGATTCGACGGACCTCGATTTGGTGGCTCCAGACCAGATGGTGCTGGAGGGAGACCTTTCTTCGGCCAAGGAGGCAGGCGTGGTGATGGAGAAGAAGAAACTGATGCTGCCCAACAAATTGGT 416  
2-1529    TGGACCTGGATTTGGTACCCCGCAGATGGATGGACGGAGACAAAATGGCGGTCCGATGGGTGGTAGGAGATTCGACGGACCTCGATTTGGTGGCTCCAGACCAGATGGTGCTGGAGGGAGACCTTTCTTCGGCCAAGGAGGAAGACGTGGTGATGGAGAAGAAGAAACTGATGCTGCCCAACAAATTGGT 489  
2-1540    TGGACCTGGATTTGGTGCCCCGGAGATGGATGGACGGAGACAAAATGGCGGTCCGATGGGTGGAAGGAGATTCGACAGACCTGGATTTGGTGGCTCCAGACCAGATGGTGCTGGAGGAAGACCTTTCTTCGGCCAAGGAGGCAGGTGTGGTGATGGAGAAGAAGAAACTGATGCTGCCCAACAAATTGGT 488  
2-1506    -------------------------------------------------------------------AGATTCGACGGACCTCGATTTGGTGGCTCCAGACCAGATGGTGCTGGAGGGAGACCTTTCTTCGGCCAAGGAGGCAGGCGTGGTGATGGAGAAGAAGAAACTGATGCTGCCCAACAAATTGGT 416  
2-1507    -------------------------------------------------------------------AGATTCGACGGACCTCGATTTGGTGGCTCCAGACCAGATGGTGCTGGAGGGAGACCTTTCTTCGGCCAAGGAGGCAGGCGTGGTGATGGAGAAGAAGAAACTGATGCTGCCCAACAAATTGGT 414  
2-1511    -------------------------------------------------------------------AGATTCGACGGACCTCGATTTGGTGGCTCCAGACCAGATGGTGCTGGAGGGAGACCTTTCTTCGGCCAAGGAGGCAGGCGTGGTGATGGAGAAGAAGAAACTGATGCTGCCCAACAAATTGGT 414  
2-1514    -------------------------------------------------------------------AGATTCGACGGACCTCGATTTGGTGGCTCCAGACCAGATGGTGCTGGAGGGAGACCTTTCTTCGGCCAAGGAGGAAGACGTGGTGATGGAAAAGAAGAAACTGATGCTGCCCAACAAATTGGT 416  
2-1516    -------------------------------------------------------------------AGATTCGACGGACCTCGATTTGGTGGCTCCAGACCAGATGGTGCTGGAGGGAGACCTTTCTTCGGCCAAGGAGGCAGGCGTGGTGATGGAGAAGAAGAAACTGATGCTGCCCAACAAATTGGT 416  
2-1528    -------------------------------------------------------------------AGATTCGACGGACCTCGATTTGGTGGCTCCAGACCAGATGGTGCTGGAGGGAGACCTTTCTTCGGCCAAGGAGGCAGGCGTGGTGATGGAGAAGAAGAAACTGATGCTGCCCAACAAATTGGT 416  
2-1532    -------------------------------------------------------------------AGATTCGACGGACCTCGATTTGGTGGCTCCAGACCAGATGGTGCTGGAGGGAGACCTTTCTTCGGCCAAGGAGGCAGGCGTGGTGATGGAGAAGAAGAAACTGATGCTGCCCAACAAGTTGGT 416  
2-1541    -------------------------------------------------------------------AGATTCGACGGACCTCGATTTGGTGGCTCCAGACCAGATGGTGCTGGAGGGAGACCTTTCTTCGGCCAAGGAGGCAGGCGTGGTGATGGAGAAGAAGAAACTGATGCTGCCCAACAAATTGGT 414  
2-1542    -------------------------------------------------------------------AGATTCGACGGACCTCGATTTGGTGGCTCCAGACCAGATGGTGCTGGAGGGAGACCTTTCTTCGGCCAAGGAGGCAGGCGTGGTGATGGAGAAGAAGAAACTGATGCTGCCCAACAAGTTGGT 416  
2-2448    -------------------------------------------------------------------AGATTCGACGGACCTCGATTTGGTGGCTCCAGACCAGATGGTGCTGGAGGGAGACCTTTCTTCGGCCAAGGAGGCAGGCGTGGTGATGGAGAAGAAGAAACTGATGCTGCCCAACAAATTGGT 416  
2-2405    -------------------------------------------------------------------AGATTCGACGGACCTCGATTTGGTGGCTCCAGACCAGATGGTGCTGGAGGGAGACCTTTCTTCGGCCAAGGAGGCAGGCGTGGTGATGGAGAAGAAGAAACTGATGCTGCCCAACAAATTGGT 416  
2-2406    -------------------------------------------------------------------AGATTCGACGGACCTCGATTTGGTGGCTCCAGACCAGATGGTGCTGGAGGGAGACCTTTCTTCGGCCAAGGAGGCAGGCGTGGTGATGGAGAAGAAGAAACTGATGCTGCCCAACAAATTGGT 416  
2-2407    -------------------------------------------------------------------AGATTCGACGGACCTCGATTTGGTGGCTCCAGACCAGATGGTGCTGGAGGGAGACCTTTCTTCGGCCAAGGAGGCAGGCGTGGTGATGGAGAAGAAGAAACTGATGCTGCCCAACAAATTGGT 416  
2-2408    -------------------------------------------------------------------AGATTCGACGGACCTCGATTTGGTGGCTCCAGACCAGATGGTGCTGGAGGGAGACCTTTCTTCGGCCAAGGAGGCAGGCGTGGTGATGGAGAAGAAGAAACTGATGCTGCCCAACAAATTGGT 416  
2-2410    -------------------------------------------------------------------AGATTCGACGGACCTCGATTTGGTGGCTCCAGACCAGATGGTGCTGGAGGGAGACCTTTCTTCGGCCAAGGAGGCAGGCGTGGTGATGGAGAAGAAGAAACTGATGCTGCCCAACAAATTGGT 416  
2-2412    -------------------------------------------------------------------AGATTCGACGGACCTCGATTTGGTGGCTCCAGACCAGATGGTGCTGGAGGGAGACCTTTCTTCGGCCAAGGAGGCAGGCGTGGTGATGGAGAAGAAGAAACTGATGCTGCCCAACAAATTGGT 416  
2-2413    -------------------------------------------------------------------AGATTCGACGGACCTCGATTTGGTGGCTCCAGACCAGATGGTGCTGGAGGGAGACCTTTCTTCGGCCAAGGAGGCAGGCGTGGTGATGGAGAAGAAGAAACTGATGCTGCCCAACAAATTGGT 416  
2-2419    -------------------------------------------------------------------AGATTCGACGGACCTCGATTTGGTGGCTCCAGACCAGATGGTGCTGGAGGGAGACCTTTCTTCGGCCAAGGAGGCAGGCGTGGTGATGGAGAAGAAGAAACTGATGCTGCCCAACAAATTGGT 416  
2-2420    -------------------------------------------------------------------AGATTCGACGGACCTCGATTTGGTGGCTCCAGACCAGATGGTGCTGGAGGGAGACCTTTCTTCGGCCAAGGAGGCAGGCGTGGTGATGGAGAAGAAGAAACTGATGCTGCCCAACAAATTGGT 416  
2-2422    -------------------------------------------------------------------AGATTCGACGGACCTCGATTTGGTGGCTCCAGACCAGATGGTGCTGGAGGGAGACCTTTCTTCGGCCAAGGAGGCAGGCGTGGTGATGGAGAAGAAGAAACTGATGCTGCCCAACAAATTGGT 416  
2-2424    -------------------------------------------------------------------AGATTCGACGGACCTCGATTTGGTGGCTCCAGACCAGATGGTGCTGGAGGGAGACCTTTCTTCGGCCAAGGAGGCAGGCGTGGTGATGGAGAAGAAGAAACTGATGCTGCCCAACAAATTGGT 416  
2-2427    -------------------------------------------------------------------AGATTCGACGGACCTCGATTTGGTGGCTCCAGACCAGATGGTGCTGGAGGGAGACCTTTCTTCGGCCAAGGAGGCAGGCGTGGTGATGGAGAAGAAGAAACTGATGCTGCCCAACAAATTGGT 416  
2-2428    -------------------------------------------------------------------AGATTCGACGGACCTCGATTTGGTGGCTCCAGACCAGATGGTGCTGGAGGGAGACCTTTCTTCGGCCAAGGAGGCAGGCGTGGTGATGGAGAAGAAGAAACTGATGCTGCCCAACAAATTGGT 416  
2-2429    -------------------------------------------------------------------AGATTCGACGGACCTCGATTTGGTGGCTCCAGACCAGATGGTGCTGGAGGGAGACCTTTCTTCGGCCAAGGAGGCAGGCGTGGTGATGGAGAAGAAGAAACTGATGCTGCCCAACAAATTGGT 416  
2-2431    -------------------------------------------------------------------AGATTCGACGGACCTCGATTTGGTGGCTCCAGACCAGATGGTGCTGGAGGGAGACCTTTCTTCGGCCAAGGAGGCAGGCGTGGTGATGGAGAAGAAGAAACTGATGCTGCCCAACAAATTGGT 416  
2-2436    -------------------------------------------------------------------AGATTCGACGGACCTCGATTTGGTGGCTCCAGACCAGATGGTGCTGGAGGGAGACCTTTCTTCGGCCAAGGAGGCAGGCGTGGTGATGGAGAAGAAGAAACTGATGCTGCCCAACAAATTGGT 416  
2-2441    -------------------------------------------------------------------AGATTCGACGGACCTCGATTTGGTGGCTCCAGACCAGATGGTGCTGGAGGGAGACCTTTCTTCGGCCAAGGAGGCAGGCGTGGTGATGGAGAAGAAGAAACTGATGCTGCCCAACAAATTGGT 415  
2-2446    -------------------------------------------------------------------AGATTCGACGGACCTCGATTTGGTGGCTCCAGACCAGATGGTGCTGGAGGGAGACCTTTCTTCGGCCAAGGAGGCAGGCGTGGTGATGGAGAAGAAGAAACTGATGCTGCCCAACAAATTGGT 416  
2-2416    -------------------------------------------------------------------AGATTCGACGGACCTCGATTTGGTGGCTCCAGACCAGATGGTGCTGGAGGGAGACCTTTCTTCGGCCAAGGAGGCAGGCGTGGTGATGGAGAAGAATAAACTGATGCTGCCCAACAAATTGGT 415  
2-2411    ---------------------------------------------------------------------------------------------------------------------------------------------------------------------------------------------- 161  
2-2415    ---------------------------------------------------------------------------------------------------------------------------------------------------------------------------------------------- 161  
2-2440    ---------------------------------------------------------------------------------------------------------------------------------------------------------------------------------------------- 161  
2-2423    ---------------------------------------------------------------------------------------------------------------------------------------------------------------------------------------------- 17   
2-2418    -------------------------------------------------------------------AGATTCGACGGACCTCGATTTGGTGGCTCCAGACCAGATGGTGCTGGAGGGAGACCTTTCTTCGGCCAAGGGGAAGACGTGGTGATGGAAAAGAAGAAACTGATGCTGCCCAACAAATTGGTG 413  
2-2421    -------------------------------------------------------------------AGATTCGACGGACCTCGATTTGGTGGCTCCAGACCAGATGGTGCTGAAGGAAGACCTTTCTTCGGCCAAGGAGGCAGGCGTGGTGATGGAGAAGAAGAAACTGATGCTGCCCAACAAATTGGT 416  
2-2438    -------------------------------------------------------------------AGATTCGACGGACCTCGATTTGGTGGCTCCAGACCAGATGGTGCTGGAGGGAGACCTTTCTTCGGCCAAGGAGGCAGGCGTGGTGATGGAGAAGAAGAAACTGATGCTGCCCAACAAATTGGT 416  
2-2409    TGGACCTGGATTTGGTGCCCCGGAGATGGATGGACGGAGACAAAATGGCGGTCCGATGGGTGGAAGGAGATTCGACGGACCTGGATTTGGTGGCTCCAGACCAGATGGTGCTGGAGGAAGACCTTTCTTCGGCCAAGGAGGCAGGCGTGGTGATGGAGAAGAAGAAACTGATGCTGCCCAACAAATTGGT 487  
2-2444    TGGACCTGGATTTGGTGCCCCGGAGATGGATGGACGGAGACAAAATGGCGGTCCGATGGGTGGAAGGAGATTCGACGGACCTGGATTTGGTGGCTCCAGACCAGTTGGTGCTGGAGGAAGACCTTTCTTCGGCCAAGGAGGCAGGCGTGGTGATGAAGAAGAAGAAACTGATGCTGCCCAACAAATTGGT 488  
2-2414    -------------------------------------------------------------------AGATTCGACGGACCTCGATTTGGTGGCTCCAGACCAGATGGTGCTGGAGGGAGACCTTTCTTCGGCCAAGGAGGCAGGCGTGGTGATGGAGAAGAAGAAACTGATGCTGCCCAACAAGTTGGT 416  
2-2403    -------------------------------------------------------------------AGATTCGACGGACCTCGATTTGGTGGCTCCAGACCAGATGGTGCTGGAGGGAGACCTTTCTTCGGCCAAGGAGGCAGGCGTGGTGATGGAGAAGAAGAAACTGATGCTGCCCAACAAATTGGT 416  
2-2404    -------------------------------------------------------------------AGATTCGACGGACCTCGATTTGGTGGCTCCAGACCAGATGGTGCTGGAGGGAGACCTTTCTTCGGCCAAGGAGGAAGACGTGGTGATGGAAAAGAAGAAACTGATGCTGCCCAACAAATTGGT 413  
2-2425    -------------------------------------------------------------------AGATTCGACGGACCTCGATTTGGTGGCTCCAGACCAGATGGTGCTGGAGGGAGACCTTTCTTCGGCCAAGGAGGAAGACGTGGTGATGGAAAAGAAGAAACTGATGCTGCCCAACAAATTGGT 413  
2-2426    -------------------------------------------------------------------AGATTCGACGGACCTCGATTTGGTGGCTCCAGACCAGATGGTGCTGGAGGGAGACCTTTCTTCGGCCAAGGAGGCAGGTGTGGTGATGGAGAAGAAGAAACTGATGCTGCCCAACAAATTGGT 416  
2-2430    -------------------------------------------------------------------AGATTCGACGGACCTCGATTTGGTGGCTCCAGACCAGATGGTGCTGGAGGGAGACCTTTCTTCGGCCAAGGAGGCAGGCGTGGTGATGGAGAAGAAGAAACTGATGCTGCCCAACAAATTGGT 416  
2-2432    -------------------------------------------------------------------AGATTCGACGGACCTCGATTTGGTGGCTCCAGACCAGATGGTGCTGGAGGGAGACCTTTCTTCGGCCAAGGAGGCAGGCGTGGTGATGGAGAAGAAGAAACTGATGCTGCCCAACAAATTGGT 413  
2-2437    -------------------------------------------------------------------AGATTCGACGGACCTCGATTTGGTGGCTCCAGACCAGATGGTGCTGGAGGGAGACCTTTCTTCGGCCAAGGAGGAAGACGTGGTGATGGAAAAGAAGAAACTGATGCTGCCCAACAAATTGGT 413  
2-2439    -------------------------------------------------------------------AGATTCGACGGACCTCGATTTGGTGGCTCCAGACCAGATGGTGCTGGAGGGAGACCTTTCTTCGGCCAAGGAGGCAGGCGTGGTGATGGAGAAGAAGAAACTGATGCTGCCCAACAAATTGGT 416  
2-2442    -------------------------------------------------------------------AGATTCGACGGACCTCGATTTGGTGGCTCCAGACCAGATGGTGCTGGAGGGAGACCTTTCTTCGGCCAAGGAGGCAGGCGTGGTGATGGAGAAGAAGAAACTGATGCTGCCCAACAAATTGGT 416  
2-2443    -------------------------------------------------------------------AGATTCGACGGACCTCGATTTGGTGGCTCCAGACCAGATGGTGCTGGAGGGAGACCTTTCTTCGGCCAAGGAGGCAGGCGTGGTGATGGAGAAGAAGAAACTGATGCTGCCCAACAAATTGGT 416  
2-2445    -------------------------------------------------------------------AGATTCGACGGACCTCGATTTGGTGGCTCCAGACCAGATGGTGCTGGAGGGAGACCTTTCTTCGGCCAAGGAGGCAGGCGTGGTGATGGAGAAGAAGAAACTGATGCTGCCCAACAAATTGGT 416  
2-2435    -------------------------------------------------------------------AGATTCGACGGACCTCGATTTGGTGGCTCCAGACCAGATGGTGCTGGAGGAAGACCTTTCTCCGGCCAAGGAGGCAGGCGTGGTGATGGAGAAGAAGAAACTGATGCTGCCCAACAAATTGGT 416  
2-2447    -------------------------------------------------------------------AGATTCGACGGACCTCGATTTGGTGGCTCCAGACCAGATGGTGCTGGAGGAAGACCTTTCTTCGGCCAAGGAGGCAGGCGTGGTGATGGAGAAGAAGAAACTGATGCTGCCCAACAAATTGGT 416  
2-2401    -------------------------------------------------------------------AGATTCGACGGACCTCGATTTGGTGGCTCCAGACCAGATGGTGCTGGAGGAAGACCTTTCTTCGGCCAAGGAGGCAGGCGTGGTGATGGAGAAGAAGAAACTGATGCTGCCCAACAAATTGGT 416  
9-1504    -------------------------------------------------------------------AGATTCGACGGACCTCGATTTGGTGGCTCCAGACCAGATGGTGCTGGAGGGAGACCTTTCTTCGGCCAAGGAGGCAGGCGTGGTGATGGAGAAGAAGAAACTGATGCTGCCCAACAAATTGGT 416  
9-1505    -------------------------------------------------------------------AGATTCGACGGACCTCGATTTGGTGGCTCCAGACCAGATGGTGCTGGAGGGAGACCTTTCTTCGGCCAAGGAGGCAGGCGTGGTGATGGAGAAGAAGAAACTGATGCTGCCCAACAAATTGGT 416  
9-1506    -------------------------------------------------------------------AGATTCGACGGACCTCGATTTGGTGGCTCCAGACCAGATGGTGCTGGAGGGAGACCTTTCTTCGGCCAAGGAGGCAGGCGTGGTGATGGAGAAGAAGAAACTGATGCTGCCCAACAAATTGGT 416  
9-1507    -------------------------------------------------------------------AGATTCGGCGGACCTCGATTTGGTGGCTCCAGACCAGATGGTGCTGGAGGGAGACCTTTCTTCGGCCAAGGAGGCAGGCGTGGTGATGGAGAAGAAGAAACTGATGCTGCCCAACAAATTGGT 416  
9-1509    -------------------------------------------------------------------AGATTCGACGGACCTCGATTTGGTGGCTCCAGACCAGATGGTGCTGGAGGGAGACCTTTCTTCGGCCAAGGAGGCAGGCGTGGTGATGGAGAAGGAGAAACTGATGCTGCCCAACAAATTGGT 416  
9-1512    -------------------------------------------------------------------AGATTCGACGGACCTCGATTTGGTGGCTCCAGACCAGATGGTGCTGGAGGGAGACCTTTCTTCGGCCAAGGAGGCAGGCGTGGTGATGGAGAAGAAGAAACTGATGCTGCCCAACAAATTGGT 416  
9-1514    -------------------------------------------------------------------AGATTCGACGGACCTCGATTTGGTGGCTCCAGACCAGATGGTGCTGGAGGGAGACCTTTCTTCGGCCAAGGAGGCAGGCGTGGTGATGGAGAAGAAGAAACTGATGCTGCCCAACAAATTGGT 416  
9-1515    -------------------------------------------------------------------AGATTCGACGGACCTCGATTTGGTGGCTCCAGACCAGATGGTGCTGGAGGGAGACCTTTCTTCGGCCAAGGAGGCAGGCGTGGTGATGGAGAAGAAGAAACTGATGCTGCCCAACAAATTGGT 416  
9-1516    -------------------------------------------------------------------AGATTCGACGGACCTCGATTTGGTGGCTCCAGACCAGATGGTGCTGGAGGGAGACCTTTCTTCGGCCAAGGAGGCAGGCGTGGTGATGGAGAAGAAGAAACTGATGCTGCCCAACAAATTGGT 416  
9-1518    -------------------------------------------------------------------AGATTCGACGGACCTCGATTTGGTGGCTCCAGACCAGATGGTGCTGGAGGGAGACCTTTCTTCGGCCAAGGAGGCAGGCGTGGTGATGGAGAAGAAGAAACTGATGCTGCCCAACAAATTGGT 416  
9-1519    -------------------------------------------------------------------AGATTCGACGGACCTCGATTTGGTGGCTCCAGACCAGATGGTGCTGGAGGGAGACCTTTCTTCGGCCAAGGAGGCAGGCGTGGTGATGGAGAAGAAGAAACTGATGCTGCCCAACAAATTGGT 416  
9-1520    -------------------------------------------------------------------AGATTCGACGGACCTCGATTTGGTGGCTCCAGACCAGATGGTGCTGGAGGGAGACCTTTCTTCGGCCAAGGAGGCAGGCGTGGTGATGGAGAAGAAGAAACTGATGCTGCCCAACAAATTGGT 416  
9-1521    -------------------------------------------------------------------AGATTCGACGGACCTCGATTTGGTGGCTCCAGACCAGATGGTGCTGGAGGGAGACCTTTCTTCGGCCAAGGAGGCAGGCGTGGTGATGGAGAAGAAGAAACTGATGCTGCCCAACAAATTGGT 416  
9-1523    -------------------------------------------------------------------AGATTCGACGGACCTCGATTTGGTGGCTCCAGACCAGATGGTGCTGGAGGGAGACCTTTCTTCGGCCAAGGAGGCAGGCGTGGTGATGGAGAAGAAGAAACTGATGCTGCCCAACAAATTGGT 416  
9-1524    -------------------------------------------------------------------AGATTCGACGGACCTCGATTTGGTGGCTCCAGACCAGATGGTGCTGGAGGGAGACCTTTCTTCGGCCAAGGAGGCAGGCGTGGTGATGGAGAAGAAGAAACTGATGCTGCCCAACAAATTGGT 414  
9-1526    -------------------------------------------------------------------AGATTCGACGGACCTCGATTTGGTGGCTCCAGACCAGATGGTGCTGGAGGGAGACCTTTCTTCGGCCAAGGAGGCAGGCGTGGTGATGGAGAAGAAGAAACTGATGCTGCCCAACAAATTGGT 416  
9-1527    -------------------------------------------------------------------AGATTCGACGGACCTCGATTTGGTGGCTCCAGACCAGATGGTGCTGGAGGGAGACCTTTCTTCGGCCAAGGAGGCAGGCGTGGTGATGGAGAAGAAGAAACTGATGCTGCCCAACAAATTGGT 416  
9-1530    -------------------------------------------------------------------AGATTCGACGGACCTCGATTTGGTGGCTCCAGACCAGATGGTGCTGGAGGGAGACCTTTCTTCGGCCAAGGAGGCAGGCGTGGTGATGGAGAAGAAGAAACTGATGCTGCCCAACAAATTGGT 416  
9-1531    -------------------------------------------------------------------AGATTCGACGGACCTCGATTTGGTGGCTCCAGACCAGATGGTGCTGGAGGGAGACCTTTCTTCGGCCAAGGAGGCAGGCGTGGTGATGGAGAAGAAGAAACTGATGCTGCCCAACAAATTGGT 416  
9-1533    -------------------------------------------------------------------AGATTCGACGGACCTCGATTTGGTGGCTCCAGACCAGATGGTGCTGGAGGGAGACCTTTCTTCGGCCAAGGAGGCAGGCGTGGTGATGGAGAAGAAGAAACTGATGCTGCCCAACAAATTGGT 416  
9-1537    -------------------------------------------------------------------AGATTCGACGGACCTCGATTTGGTGGCTCCAGACCAGATGGTGCTGGAGGGAGACCTTTCTTCGGCCAAGGAGGCAGGCGTGGTGATGGAGAAGAAGAAACTGATGCTGCCCGACAAATTGGT 416  
9-1538    -------------------------------------------------------------------AGATTCGACGGACCTCGATTTGGTGGCTCCAGACCAGATGGTGCTGGAGGGAGACCTTTCTTCGGCCAAGGAGGCAGGCGTGGTGATGGAGAAGAAGAAACTGATGCTGCCCAACAAATTGGT 416  
9-1539    -------------------------------------------------------------------AGATTCGACGGACCTCGATTTGGTGGCTCCAGACCAGATGGTGCTGGAGGGAGACCTTTCTTCGGCCAAGGAGGCAGGCGTGGTGATGGAGAAGAAGAAACTGATGCTGCCCAACAAATTGGT 416  
9-1540    -------------------------------------------------------------------AGATTCGACGGACCTCGATTTGGTGGCTCCAGACCAGATGGTGCTGGAGGGAGACCTTTCTTCGGCCAAGGAGGCAGGCGTGGTGATGGAGAAGAAGAAACTGATGCTGCCCAACAAATTGGT 416  
9-1543    -------------------------------------------------------------------AGATTCGACGGACCTCGATTTGGTGGCTCCAGACCAGATGGTGCTGGAGGGAGACCTTTCTTCGGCCAAGGAGGCAGGCGTGGTGATGGAGAAGAAGAAACTGATGCTGCCCAACAAATTGGT 416  
9-1544    -------------------------------------------------------------------AGATTCGACGGACCTCGATTTGGTGGCTCCAGACCAGATGGTGCTGGAGGGAGACCTTTCTTCGGCCAAGGAGGCAGGCGTGGTGATGGAGAAGAAGAAACTGATGCTGCCCAACAAATTGGT 416  
9-1545    -------------------------------------------------------------------AGATTCGACGGACCTCGATTTGGTGGCTCCAGACCAGATGGTGCTGGAGGGAGACCTTTCTTCGGCCAAGGAGGCAGGCGTGGTGATGGAGAAGAAGAAACTGATGCTGCCCAACAAATTGGT 416  
9-1546    -------------------------------------------------------------------AGATTCGACGGACCTCGATTTGGTGGCTCCAGACCAGATGGTGCTGGAGGGAGACCTTTCTTCGGCCAAGGAGGCAGGCGTGGTGATGGAGAAGAAGAAACTGATGCTGCCCAACAAATTGGT 416  
9-1547    -------------------------------------------------------------------AGATTCGACGGACCTCGATTTGGTGGCTCCAGACCAGATGGTGCTGGAGGGAGACCTTTCTTCGGCCAAGGAGGCAGGCGTGGTGATGGAGAAGAAGAGACTGATGCTGCCCAACAAATTGGT 416  
9-1548    -------------------------------------------------------------------AGATTCGACGGACCTCGATTTGGTGGCTCCAGACCAGATGGTGCTGGAGGGAGACCTTTCTTCGGCCAAGGAGGCAGGCGTGGTGATGGAGAAGAAGAAACTGATGCTGCCCAACAAATTGGT 416  
9-1513    TGGACCTGGATTTGGTGCCCCGGAGATGGATGGACGGAGACAAAATGGCGGTCCGATGGGTGGAAGGAGATTCGACGGACCTGGATTTGGTGGCTCCAGACCAGATGGTGCTGGAGGAAGACCTTTCTTCGGCCAAGGAGGCAGGCGTGGTGATGGAGAAGAAGAAACTGATGCTGCCCAACAAATTGGT 488  
9-1541    TGGACCTGGATTTGGTGCCCCGGAGATGGATGGACGGAGACAAAATGGCGGTCCGATGGGTGGAAGGAGATTCGACGGACCTGGATTTGGTGGCTCCAGACCAGATGGTGCTGGAGGAAGACCTTTCTTCGGCCAAGGAGGCAGGCGTGGTGATGGAGAAGAAGAAACTGACGCTGCCCAACAAATTGGT 488  
9-1542    TGGACCTGGATTTGGTGCCCCGGAGATGGATGGACGGAGACAAAATGGCGGTCCGATGGGTGGAAGGAGATTCGACGGACCTGGATTTGGTGGCTCCAGACCAGATGGTGCTGGAGGAAGACCTTTCTTCGGCCAAGGAGGCAGGCGTGGTGATGGAGAAGAAGAAACTGATGCTGCCCAACAAATTGGT 488  
9-1525    TGGACCTGGATTTGGTGCCCCGCAAATGGGTGGACCTAGGCAAAATGGCGGTCCGATGGGTGGTAGGAGATTCGACGGACCTGGATTTGGTGGCTCCAGACCAGATGGTGCTGGAGGAAGACCTTTCTTCGGCGAAGGAGGTAGGCGTGGTGATGGAGAAGAAGAAACTGATGCTGCCCGACAAATTGGG 566  
9-1501    -------------------------------------------------------------------AGATTCGACGGACCTCGATTTGGTGGCTCCAGACCAGATGGTGCTGGAGGGAGACCTTTCTTCGGCCAAGGAGGCAGGCGTGGTGATGGAGAAGAAGAAACTGATGCTGCCCAACAAATTGGT 415  
9-1502    -------------------------------------------------------------------AGATTCGACGGACCTCGATTTGGTGGCTCCAGACCAGATGGTGCTGGAGGGAGACCTTTCTTCGGCCAAGGAGGCAGGCGTGGTGATGGAGAAGAAGAAACTGATGCTGCCCAACAAATTGGT 416  
9-1508    -------------------------------------------------------------------AGATTCGACGGACCTCGATTTGGTGGCTCCAGACCAGATGGTGCTGGAGGGAGACCTTTCTTCGGCCAAGGAGGCAGGCGTGGTGATGGAGAAGAAGAAACTGATGCTGCCCAACAAGTTGGT 416  
9-1511    -------------------------------------------------------------------AGATTCGACGGACCTCGATTTGGTGGCTCCAGACCAGATGGTGCTGGAGGGAGACCTTTCTTCGGCCAAGGAGGCAGGCGTGGTGATGGAGAAGAAGAAACTGATGCTGCCCAACAAGTTGGT 416  
9-1517    -------------------------------------------------------------------AGATTCGACGGACCTCGATTTGGTGGCTCCAGACCAGATGGTGCTGGAGGGAGACCTTTCTTCGGCCAAGGAGGCAGGCGTGGTGATGGAGAAGAAGAAACTGATGCTGCCCAACAAATTGGT 416  
9-1528    -------------------------------------------------------------------AGATTCGACGGACCTCGATTTGGTGGCTCCAGACCAGATGGTGCTGGAGGGGGACCTTTCTTCGGCCAAGGAGGCAGGCGTGGTGATGGAGAAGAAGAAACTGATGCTGCCCAACAAGTTGGT 416  
9-1529    -------------------------------------------------------------------AGATTCGACGGACCTCGATTTGGTGGCTCCAGACCAGATGGTGCTGGAGGGAGACCTTTCTTCGGCCAAGGAGGCAGGCGTGGTGATGGAGAAGAAGAAACTGATGCTGCCCAACAAATTGGT 416  
9-1534    -------------------------------------------------------------------AGATTCGACGGACCTCGATTTGGTGGCTCCAGACCAGATGGTGCTGGAGGGAGACCTTTCTTCGGCCAAGGAGGCAGGCGTGGTGATGGAGAAGAAGAAACTGATGCTGCCCAACAAGTTGGT 416  
9-1535    -------------------------------------------------------------------AGATTCGACGGACCTCGATTTGGTGGCTCCAGACCAGATGGTGCTGGAGGGAGACCTTTCTTCGGCCAAGGAGGCAGGCGTGGTGATGGAGAAGAAGAAACTGATGCTGCCCAACAAGTTGGT 416  
9-2448    -------------------------------------------------------------------AGATTCGACGGACCTCGATTTGGTGGCTCCAGACCAGATGGTGCTGGAGGGAGACCTTTCTTCGGCCAAGGAGGCAGGCGTGGTGATGGAGAAGAAGAAACTGATGCTGCCCAACAAATTGGT 416  
9-2401    -------------------------------------------------------------------AGATTCGACGGACCTCGATTTGGTGGCTCCAGACCAGATGGTGCTGGAGGGAGACCTTTCTTCGGCCAAGGAGGCAGGCGTGGTGATGGAGAAGAAGAAACTGATGCTGCCCAACAAATTGGT 416  
9-2402    -------------------------------------------------------------------AGATTCGACGGACCTCGATTTGGTGGCTCCAGACCAGATGGTGCTGGAGGGAGACCTTTCTTCGGCCAAGGAGGCAGGCGTGGTGATGGAGAAGAAGAAACTGATGCTGCCCAACAAATTGGT 416  
9-2404    -------------------------------------------------------------------AGATTCGACGGACCTCGATTTGGTGGCTCCAGACCAGATGGTGCTGGAGGGAGACCTTTCTTCGGCCAAGGAGGCAGGCGTGGTGATGGAGAAGAAGAAACTGATGCTGCCCAACAAATTGGT 416  
9-2406    -------------------------------------------------------------------AGATTCGACGGACCTCGATTTGGTGGCTCCAGACCAGATGGTGCTGGAGGGAGACCTTTCTTCGGCCAAGGAGGCAGGCGTGGTGATGGAGAAGAAGAAACTGATGCTGCCCAACAAATTGGT 416  
9-2408    -------------------------------------------------------------------AGATTCGACGGACCTCGATTTGGTGGCTCCAGACCAGATGGTGCTGGAGGGAGACCTTTCTTCGGCCAAGGAGGCAGGCGTGGTGATGGAGAAGAAGAAACTGATGCTGCCCAACAAATTGGT 416  
9-2409    -------------------------------------------------------------------AGATTCGACGGACCTCGATTTGGTGGCTCCAGACCAGATGGTGCTGGAGGGAGACCTTTCTTCGGCCAAGGAGGCAGGCGTGGTGATGGAGAAGAAGAAACTGATGCTGCCCAACAAATTGGT 416  
9-2411    -------------------------------------------------------------------AGATTCGACGGACCTCGATTTGGTGGCTCCAGACCAGATGGTGCTGGAGGGAGACCTTTCTTCGGCCAAGGAGGCAGGCGTGGTGATGGAGAAGAAGAAACTGATGCTGCCCAACAAATTGGT 416  
9-2413    -------------------------------------------------------------------AGATTCGACGGACCTCGATTTGGTGGCTCCAGACCAGATGGTGCTGGAGGGAGACCTTTCTTCGGCCAAGGAGGCAGGCGTGGTGATGGAGAAGAAGAAACTGATGCTGCCCAACAAATTGGT 416  
9-2414    -------------------------------------------------------------------AGATTCGACGGACCTCGATTTGGTGGCTCCAGACCAGATGGTGCTGGAGGGAGACCTTTCTTCGGCCAAGGAGGCAGGCGTGGTGATGGAGAAGAAGAAACTGATGCTGCCCAACAAATTGGT 416  
9-2417    -------------------------------------------------------------------AGATTCGACGGACCTCGATTTGGTGGCTCCAGACCAGATGGTGCTGGAGGGAGACCTTTCTTCGGCCAAGGAGGCAGGCGTGGTGATGGAGAAGAAGAAACTGATGCTGCCCAACAAATTGGT 416  
9-2418    -------------------------------------------------------------------AGATTCGACGGACCTCGATTTGGTGGCTCCAGACCAGATGGTGCTGGAGGGAGACCTTTCTTCGGCCAAGGAGGCAGGCGTGGTGATGGAGAAGAAGAAACTGATGCTGCCCAACAAATTGGT 416  
9-2419    -------------------------------------------------------------------AGATTCGACGGACCTCGATTTGGTGGCTCCAGACCAGATGGTGCTGGAGGGAGACCTTTCTTCGGCCAAGGAGGCAGGCGTGGTGATGGAGAAGAAGAAACTGATGCTGCCCAACAAATTGGT 416  
9-2420    -------------------------------------------------------------------AGATTCGACGGACCTCGATTTGGTGGCTCCAGACCAGATGGTGCTGGAGGGAGACCTTTCTTCGGCCAAGGAGGCAGGCGTGGTGATGGAGAAGAAGAAACTGATGCTGCCCAACAAATTGGT 416  
9-2421    -------------------------------------------------------------------AGATTCGACGGACCTCGATTTGGTGGCTCCAGACCAGATGGTGCTGGAGGGAGACCTTTCTTCGGCCAAGGAGGCAGGCGTGGTGATGGAGAAGAAGAAACTGATGCTGCCCAACAAATTGGT 416  
9-2424    -------------------------------------------------------------------AGATTCGACGGACCTCGATTTGGTGGCTCCAGACCAGATGGTGCTGGAGGGAGACCTTTCTTCGGCCAAGGAGGCAGGCGTGGTGATGGAGAAGAAGAAACTGATGCTGCCCAACAAATTGGT 416  
9-2427    -------------------------------------------------------------------AGATTCGACGGACCTCGATTTGGTGGCTCCAGACCAGATGGTGCTGGAGGGAGACCTTTCTTCGGCCAAGGAGGCAGGCGTGGTGATGGAGAAGAAGAAACTGATGCTGCCCAACAAATTGGT 416  
9-2429    -------------------------------------------------------------------AGATTCGACGGACCTCGATTTGGTGGCTCCAGACCAGATGGTGCTGGAGGGAGACCTTTCTTCGGCCAAGGAGGCAGGCGTGGTGATGGAGAAGAAGAAACTGATGCTGCCCAACAAATTGGT 416  
9-2430    -------------------------------------------------------------------AGATTCGACGGACCTCGATTTGGTGGCTCCAGACCAGATGGTGCTGGAGGGAGACCTTTCTTCGGCCAAGGAGGCAGGCGTGGTGATGGAGAAGAAGAAACTGATGCTGCCCAACAAATTGGT 416  
9-2433    -------------------------------------------------------------------AGATTCGACGGACCTCGATTTGGTGGCTCCAGACCAGATGGTGCTGGAGGGAGACCTTTCTTCGGCCAAGGAGGCAGGCGTGGTGATGGAGAAGAAGAAACTGATGCTGCCCAACAAATTGGT 416  
9-2435    -------------------------------------------------------------------AGATTCGACGGACCTCGATTTGGTGGCTCCAGGCCAGATGGTGCTGGAGGGAGACCTTTCTTCGGCCAAGGAGGCAGGCGTGGTGATGGAGAAGAAGAAACTGATGCTGCCCAACAAATTGGT 416  
9-2438    -------------------------------------------------------------------AGATTCGACGGACCTCGATTTGGTGGCTCCAGACCAGATGGTGCTGGAGGGAGACCTTTCTTCGGCCAAGGAGGCAGGCGTGGTGATGGAGAAGAAGAAACTGATGCTGCCCAACAAATTGGT 416  
9-2439    -------------------------------------------------------------------AGATTCGACGGACCTCGATTTGGTGGCTCCATACCAGATGGTGCTGGAGGGAGACCTTTCTTCGGCCAAGGAGGCAGGCGTGGTGATGGAGAAGAAGAAACTGATGCTGCCCAACAAATTGGT 416  
9-2440    -------------------------------------------------------------------AGATTCGACGGACCTCGATTTGGTGGCTCCAGACCAGATGGTGCTGGAGGGAGACCTTTCTTCGGCCAAGGAGGCAGGCGTGGTGATGGAGAAGAAGAAACTGATGCTGCCCAACAAATTGGT 416  
9-2441    -------------------------------------------------------------------AGATTCGACGGACCTCGATTTGGTGGCTCCAGACCAGATGGTGCTGGAGGGAGACCTTTCTTCGGCCAAGGAGGCAGGCGTGGTGATGGAGAAGAAGAAACTGATGCTGCCCAACAAATTGGT 416  
9-2442    -------------------------------------------------------------------AGATTCGACGGACCTCGATTTGGTGGCTCCAGACCAGATGGTGCTGGAGGGAGACCTTTCTTCGGCCAAGGAGGCAGGCGTGGTGATGGAGAAGAAGAAACTGATGCTGCCCAACAAATTGGT 416  
9-2443    -------------------------------------------------------------------AGATTCGACGGACCTCGATTTGGTGGCTCCAGACCAGATGGTGCTGGAGGGAGACCTTTCTTCGGCCAAGGAGGCAGGCGTGGTGATGGAGAAGAAGAAACTGATGCTGCCCAACAAATTGGT 416  
9-2445    -------------------------------------------------------------------AGATTCGACGGACCTCGATTTGGTGGCTCCAGACCAGATGGTGCTGGAGGGAGACCTTTCTTCGGCCAAGGAGGCAGGCGTGGTGATGGAGAAGAAGGAACTGATGCTGCCCAACAAATTGGT 416  
9-2446    -------------------------------------------------------------------AGATTCGACGGACCTCGATTTGGTGGCTCCAGACCAGATGGTGCTGGAGGGAGACCTTTCTTCGGCCAAGGAGGCAGGCGTGGTGATGGAGAAGAAGAAACTGATGCTGCCCAACAAATTGGT 416  
9-2416    TGGACCTGGATTTGGTGCCCCGGAGATGGATGGACGGAGACAAAATGGCGGTCCGATGGGTGGAAGGAGATTCGACGGACCTGGATTTGGTGGCTCCAGACCAGGTGGTGCTGGAGGAAGACCTTTCTTCGGCCAAGGAGGCAGGCGTGGTGATGGAGAAGAAGAAACTGATGCTGCCCAACAAATTGGT 488  
9-2423    TGGACCTGGATTTGGTGCCCCGGAGATGGATGGACGGAGACAAAATGGCGGTCCGATGGGTGGAAGGAGATTCGACGGACCTGGATTTGGTGGCTCCAGACCAGATGGTGCTGGAGGAAGACCTTTCTTCGGCCAAGGAGGCAGGCGTGGTGATGGAGAAGAAGAAACTGATGCTGCCCAACAAATTGGT 488  
9-2403    -------------------------------------------------------------------AGATTCGACGGACCTCGATTTGGTGGCTCCAGACCAGATGGTGCTGGAGGGAGACCTTTCTTCGGCCAAGGAGGCAGGCGTGGTGATGGAGAAGAAGAAACTGATGCTGCCCAACAAATTGGT 416  
9-2415    -------------------------------------------------------------------AGATTCGACGGACCTCGATTTGGTGGCTCCAGACCAGATGGTGCTGGAGGGAGACCTTTCTTCGGCCAAGGAGGCAGGCGTGGTGATGGAGAAGAAGAAACTGATGCTGCCCAACAAGTTGGT 416  
9-2422    -------------------------------------------------------------------AGATTCGACGGACCTCGATTTGGTGGCTCCAGACCAGATGGTGCTGGAGGGAGACCTTTCTTCGGCCAAGGAGGCAGGCGTGGTGATGGAGAAGAAGAAACTGATGCTGCCCAACAAATTGGT 414  
9-2428    -------------------------------------------------------------------AGATTCGACGGACCTCGATTTGGTGGCTCCAGACCAGATGGTGCTGGAGGGAGACCTTTCTTCGGCCAAGGAGGCAGGCGTGGTGATGGAGAAGAAGAAACTGATGCTGCCCAACAAATTGGT 416  
9-2431    -------------------------------------------------------------------AGATTCGACGGACCTCGATTTGGTGGCTCCAGACCAGATGGTGCTGGAGGGAGACCTTTCTTCGGCCAAGGAGGCAGGCGTGGTGATGGAGAAGAAGAAACTGATGCTGCCCAACAAGTTGGT 416  
9-2432    -------------------------------------------------------------------AGATTCGACGGACCTCGATTTGGTGGCTCCAGACCAGATGGTGCTGGAGGGAGACCTTTCTTCGGCCAAGGAGGCAGGCGTGGTGATGGAGAAGAAGAAACTGATGCTGCCCAACAAATTGGT 416  
9-2434    -------------------------------------------------------------------AGATTCGACGGACCTCGATTTGGTGGCTCCAGACCAGATGGTGCTGGAGGGAGACCTTTCTTCGGCCAAGGAGGCAGGCGTGGTGATGGAGAAGAAGAAACTGATGCTGCCCAACAAATTGGT 416  
9-2437    -------------------------------------------------------------------AGATTCGACGGACCTCGATTTGGTGGCTCCAGACCAGATGGTGCTGGAGGGAGACCTTTCTTCGGCCAAGGAGGCAGGCGTGGTGATGGAGAAGAAGAAACTGATGCTGCCCAACAAATTGGT 416  
9-2444    -------------------------------------------------------------------AGATTCGACGGACCTCGATTTGGTGGCTCCAGACCAGATGGTGCTGGAGGGAGACCTTTCTTCGGCCAAGGAGGCAGGCGTGGTGATGGAGAAGAAGAAACTGATGCTGCCCAACAAGTTGGT 416  
4-1504    TGGACCTGGATTTGGTGCCCCGGAGATGGATGGACGGAGACAAAATGGCGGTCCGATGGGTGGAAGGAGATTCGACGGACCTGGATTTGGTGGCTCCAGACCAGATGGTGCTGGAGGAAGACCTTTCTTCGGCCAAGGAGGCAGGCGTGGTGATGGAGAAGAAGAAACTGATGCTGCCCAACAAATTGGT 488  
4-1522    -------------------------------------------------------------------AGATTCGACGGACCTGGATTTGGTGGCTCCAGACCAGATGGTGCTGGAGGAAGACCTTTCTTCGGCCAAGGAGGCAGGCGTGGTGATGGAGAAGAAGAAACTGATGCTGCCCAACAAATTGGT 416  
4-1507    -------------------------------------------------------------------AGATTCGACGGACCTCGATTTGGTGGCTCCAGACCAGATGGTGCTGGAGGGAGACCTTTCTTCGGCCAAGGAGGCAGGCGTGGTGATGGAGAAGAAGAAACTGATGCTGCCCAACAAATTGGT 416  
4-1519    -------------------------------------------------------------------AGATTCGACGGACCTCGATTTGGTGGCTCCAGACCAGATGGTGCTGGAGGGAGACCTTTCTTCGGCCAAGGAGGCAGGCGTGGTGATGGAGAAGAAGAAACTGATGCTGCCCAACAAATTGGT 416  
4-1529    -------------------------------------------------------------------AGATTCGACGGACCTCGATTTGGTGGCTCCAGACCAGATGGTGCTGGAGGGAGACCTTTCTTCGGCCAAGGAGGCAGGCGTGGTGATGGAGAAGAAGAAACTGATGCTGCCCAACAAATTGGT 416  
4-1549    -------------------------------------------------------------------AGATTCGACGGACCTCGATTTGGTGGCTCCAGACCAGATGGTGCTGGAGGGAGACCTTTCTTCGGCCAAGGAGGCAGGCGTGGTGATGGAGAAGAAGAAACTGATGCTGCCCAACAAATTGGT 416  
4-1510    -------------------------------------------------------------------AGATTCGACGGACCTCGATTTGGTGGCTCCAGACCAGATGGTGCTGGAGGGAGACCTTTCTTCGGCCAAGGAGGCAGGCGTGGTGATGGAGAAGAAGAAACTGATGCTGCCCAACAAATTGGT 418  
4-1539    -------------------------------------------------------------------AGATTCGACGGACCTCGATTTGGTGGCTCCAGACCAGATGGTGCTGGAGGGAGACCTTTCTTCGGCCAAGGAGGCAGGCGTGGTGATGGAGAAGAAGAAACTGATGCTGCCCAACAAATTGGT 416  
4-2424    -------------------------------------------------------------------AGATTCGACGGACCTCGATTTGGTGGCTCCAGACCAGATGGTGCTGGAGGGAGACCTTTCTTCGGCCAAGGAGGCAGGCGTGGTGATGGAGACGAAGAAACTGATGCTGCCCAACAAATTGGT 388  
4-2401    -------------------------------------------------------------------AGATTCGACGGACCTCGATTTGGTGGCTCCAGACCAGATGGTGCTGGAGGGAGACCTTTCTTCGGCCAAGGAGGAAGACGTGGTGATGGAGAAGAAGAAACTGATGCTGCCCAACAAATTGGT 414  
4-2405    -------------------------------------------------------------------AGATTCGACAGACCTCGATTTGGTGGCCCCAGACCAGATGGTGCTGGAGGGAGACCTTTCTTCGGCCAAGGAGGCAGGCGTGGTGATGGAGAAGAAGAAACTGATGCTGCCCAACAAATTGGT 414  
4-2407    -------------------------------------------------------------------AGATTCGACGGACCTCGATTTGGTGGCTCCAGACCAGATGGTGCTGGAGGGAGACCTTTCTTCGGCCAAGGAGGAAGACGTGGTGATGGAGAAGAAGAAACTGATGCTGCCCAACAAATTGGT 414  
4-2408    -------------------------------------------------------------------AGATTCGACGGACCTCGATTTGGTGGCTCCAGACCAGATGGTGCTGGAGGGAGACCTTTCTTCGGCCAAGGAGGAAGACGTGGTGATGGAGAAGAAGAAACTGATGCTGCCCAACAAATTGGT 414  
4-2411    -------------------------------------------------------------------AGATTCGACGGACCTCGATTTGGTGGCTCCAGACCAGATGGTGCTGGAGGGAGACCTTTCTTCGGCCAAGGAGGCAGGCGTGGTGATGGAGAAGAAGAAACTGATGCTGCCCAACAAATTGGT 413  
4-2417    -------------------------------------------------------------------AGATTCGACGGACCTCGATTTGGTGGCTCCAGACCAGATGGTGCTGGAGGGAGACCTTTCTTCGGCCAAGGAGGAAGACGTGGTGATGGAGAAGAAGAAACTGATGCTGCCCAACAAATTGGT 414  
4-2418    -------------------------------------------------------------------AGATTCGACGGACCTCGATTTGGTGGCTCCAGACCAGATGGTGCTGGAGGGAGACCTTTCTTCGGCCAAGGAGGAAGACGTGGTGATGGAGAAGAAGAAACTGATGCTGCCCAACAAATTGGT 414  
4-2419    -------------------------------------------------------------------AGATTCGACGGACCTCGATTTGGTGGCTCCAGACCAGATGGTGCTGGAGGGAGACCTTTCTTCGGCCAAGGAGGAAGACGTGGTGATGGAGAAGAAGAAACTGATGCTGCCCAACAAATTGGT 412  
4-2421    -------------------------------------------------------------------AGATTCGACGGACCTCGATTTGGTGGCTCCAGACCAGATGGTGCTGGAGGGAGACCTTTCTTCGGCCAAGGAGGAAGACGTGGTGATGGAGAAGAAGAAACTGATGCTGCCCAACGAATTGGT 414  
4-2422    -------------------------------------------------------------------AGATTCGACGGACCTCGATTTGGTGGCTCCAGACCAGATGGTGCTGGAGGGAGACCTTTCTTCGGCCAAGGAGGAAGACGTGGTGATGGAGAAGAAGAAACTGATGCTGCCCAACAAATTGGT 414  
4-2426    -------------------------------------------------------------------AGATTCGACGGACCTCGATTTGGTGGCTCCAGACCAGATGGTGCTGGAGGGAGACCTTTCTTCGGCCAAGGAGGAAGACGTGGTGATGGAGAAGAAGAAACTGATGCTGCCCAACAAATTGGT 414  
4-2428    -------------------------------------------------------------------AGATTCGACGGACCTCGATTTGGTGGCTCCAGACCAGATGGTGCTGGAGGGAGACCTTTCTTCGGCCAAGGAGGAAGACGTGGTGATGGAGAAGAAGAAACTGATGCTGCCCAACAAATTGGT 414  
4-2429    -------------------------------------------------------------------AGATTCGACGGACCTCGATTTGGTGGCTCCAGACCAGATGGTGCTGGAGGGAGACCTTTCTTCGGCCAAGGAGGAAGACGTGGTGATGGAGAAGAAGAAACTGATGCTGCCCAACAAATTGGT 414  
4-2435    -------------------------------------------------------------------AGATTCGACGGACCTCGATTTGGTGGCTCCAGACCAGATGGTGCTGGAGGGAGACCTTTCTTTGGCCAAGGAGGAAGACGTGGTGATGGAGAAGAAGAAACTGATGCTGCCCAACAAATTGGT 414  
4-2437    -------------------------------------------------------------------AGATTCGACGGACCTCGATTTGGTGGCTCCAGACCAGATGGTGCTGGAGGGAGACCTTTCTTCGGCCAAGGAGGCAGGCGTGGTGATGGAGAAGAAGAAACTGATGCTGCCCAACAAGTTGGT 416  
4-2444    -------------------------------------------------------------------AGATTCGACGGACCTCGATTTGGTGGCTCCAGACCAGATGGTGCTGGAGGGAGACCTTTCTTCGGCCAAGGAGGAAGACGTGGTGATGGAGAAGAAGAAACTGATGCTGCCCAACAAATTGGT 414  
4-2445    -------------------------------------------------------------------AGATTCGACGGACCTCGATTTGGTGGCTCCAGACCAGATGGTGCTGGAGGGAGACCTTTCTTCGGCCAAGGAGGAAGACGTGGTGATGGAGAAGAAGAAACTGATGCTGCCCAACAAATTGGT 414  
4-2447    -------------------------------------------------------------------AGATTCGACGGACCTCGATTTGGTGGCTCCAGACCAGATGGTGCTGGAGGGAGACCTTTCTTCGGCCAAGGAGGAAGACGTGGTGATGGAGAAGAAGAAACTGATGCTGCCCAACAAATTGGT 414  
4-2448    -------------------------------------------------------------------AGATTCGACGGACCTCGATTTGGTGGCTCCAGACCAGATGGTGCTGGAGGGAGACCTTTCTTCGGCCAAGGAGGCAGGCGTGGTGATGGAGAAGAAGACACTGATGCTGCCCAACAAATTGGT 406  
4-2404    TGGACCTGGATTTGGTGCCCCGGAGATGGATGGACGGAGACAAAATGGCGGTCCGATGGGTGGAAGGAGATTCGACGGACCTGGATTTGGTGGCTCCAGACCAGATGGTGCTGGAGGAAGACCTTTCTTCGGCCAAGGAGGCAGGCGTGGTGATGGAGAAGAAGAAACTGATGCTGCCCAACAAATTGGT 487  
4-2413    TGGACCTGGATTTGGTGCCCCGGAGATGGATGGACGGAGACAAAATGGCGGTCCGATGGGTGGAAGGAGATTCGACGGACCTGGATTTGGTGGCTCCAGACCAGTTGGTGCTGGAGGAAGACCTTTCTTCGGCCAAGGAGGCAGGCGTGGTGATGGAGAAGAAGAAACTGATGCTGCCCAACAAATTGGT 488  
4-2450    TGGACCTGGATTTGGTGCCCCGGAGATGGATGGACGGAGACAAAATGGCGGTCCGATGGGTGGAAGGAGATTCGACGGACCTGGATTTGGTGGCTCCAGACCAGTTGGTGCTGGAGGAAGACCTTTCTTCGGCCAAGGAGGCAGGCGTGGTGATGGAGAAGAAGAAACTGATGCTGCCCAACAAATTGGT 488  
4-2420    TGGACCTGGATTTGGTGCCCCGGAGATGGATGGACGGAGACAAAATGGCGGTCCGATGGGTGGTAGGAGATTCGACGGACCTGGATTTGGTGGCTCCAGACCAGATGGTGCTGGAGGAAGACCTTTCTTCGGCCAAGGAGGCAGGCGTGGTGATGGAGAAGAAGAAACTGACGCTGCCCAACAAATTGGT 489  
4-2438    TGGACCTGGATTTGGTGCCCCGGAGATGGATGGACGGAGACAAAATGGCGGTCCGATGGGTGGAAGGAGATTCGACGGACCTGGATTTGGTGGCTCCAGACCAGATGGTGCTGGAGGAAGACCTTTCTTCGGCCAAGGAGGCAGGCGTGGTGATGGAGAAGAAGAAACTGATGCTGCCCAACAAATTGGT 489  
4-2430    TGGACCTGGATTTGGTGCCCCGGAGATGGATGGACGGAGACAAAATGGCGGTCCGATGGGTGGTAGGAGATTCGACGGACCTGGATTTGGTGGCTCCAGACCAGATGGTGCTGGAGGAAGACCTTTCTTCGGCCAAGGAGGCAGGCGTGGTGATGGAGAAGAAGAAACTGACGCTGCCCAACAAATTGGT 489  
4-2439    -------------------------------------------------------------------AGATTCGACGGACCTCGATTTGGTGGCTCCAGACCAGATGGTGCTGGAGGGAGACCTTTCTTCGGCCAAGGAGGAAGACGTGGTGATGGAGAAGAAGAAACTGATGCTGCCCAACAAATTGGT 414  
4-2433    -------------------------------------------------------------------AGATTCGACGGACCTCGATTTGGTGGCTCCAGACCAGATGGTGCTGGAGGAAGACCTTTCTTCGGCCAAGGAGGAAGACGTGGTGATGGAGAAGAAGAAACTGATGCTGCCCAACAAATTGGT 414  
4-2441    -------------------------------------------------------------------AGATTCGACGGACCTCGATTTGGTGGCTCCAGACCAGATGGTGCTGGAGGAAGACCTTTCTTCGGCCAAGGAGGAAGACGTGGTGATGGAGAAGAAGAAACTGATGCTGCCCAACAAATTGGT 414  
4-2406    -------------------------------------------------------------------AGATTCGACGGACCTGGATTTGGTGGCTCCAGACCAGATGGTGCTGGAGGAAGACCTTTCTTCGGCGAAGGAGGTAGGCGTGGTGATGGAGAAGAAGAAACTGATGCTGCCCGACAAATTGAT 489  
4-2425    -------------------------------------------------------------------AGATTCGACGGACCTGGATTTGGTGGCTCCAGACCAGATGGTGCTGGAGGAAGACCTTTCTTCGGCCAAGGAGGCAGGCGTGGTGATGGAGAAGAAGAAACTGATGCTGCCCAACAAATTGGT 413  
4-2432    -------------------------------------------------------------------AGATTCGACGGACCTGGATTTGGTGGCTCCAGACCAGATGGTGCTGGAGGAAGACCTTTCTTCGGCCAAGGAGGCAGGCGTGGTGATGGAGAAGAAGAAACTGATGCTGCCCAACAAATTGGT 413  
4-2440    -------------------------------------------------------------------AGATTCGACGGACCTGGATTTGGTGGCTCCAGACCAGATGGTGCTGGAGGAAGACCTTTCTTCGGCCAAGGAGGCAGGCGTGGTGATGGAGAAGAAGAAACTGATGCTGCCCAACAAATTGGT 413  
4-2402    ---------------------------------------------------------------------------------------------------------------------------------------------------------------------------------------------- 114  
4-2442    ---------------------------------------------------------------------------------------------------------------------------------------------------------------------------------------------- 17   
4-2443    -------------------------------------------------------------------AGATTCGACGGACCTGGATTTGGTGGCTCCAGACCAGATGGTGCTGGAGGAAGACCTTTCTTCGGCCAAGGAGGCAGGCGTGGTGATGGAGAAGAAGAAACTGATGCTGCCCAACAAATTGGT 413  
4-2416    ---------------------------------------------------------------------------------------------------------------------------------------------------------------------------------------------- 296  
4-2446    -------------------------------------------------------------------AGATTCGACGGACCTCGATTTGGTGGCTCCAGACCAGATGGTGCTGGAGGGAGACCTTTCTTCGGCCAAGGAGGCAGGCGTGGTGATGGAGAAGAAGAAACTGATGCTGCCCAACAAATTGGT 416  
4-2403    -------------------------------------------------------------------AGATTCGACGGACCTCGATTTGGTGGCTCCAGACCAGATGGTGCTGGAGGGAGACCTTTCTTCGGCCAAGGAGGCAGGCGTGGTGATGGAGAAGAAGAAACTGATGCTGCCCAACAAATTGGT 416  
4-2423    -------------------------------------------------------------------AGATTCGACGGACCTCGATTTGGTGGCTCCAGACCAGATGGTGCTGGAGGGAGACCTTTCTTCGGCCAAGGAGGCAGGCGTGGTGATGGAGAAGAAGAAACTGATGCTGCCCAACAAATTGGT 416  
4-2409    -------------------------------------------------------------------AGATTCGACGGACCTCGATTTGGTGGCTCCAGACCAGATGGTGCTGGAGGGAGACCTTTCTTCGGCCAAGGAGGCAGGCGTGGTGATGGAGAAGAAGAAACTGATGCTGCCCAACAAATTGGT 416  
4-2434    -------------------------------------------------------------------AGATTCGACGGACCTCGATTTGGTGGCTCCAGACCAGATGGTGCTGGAGGGAGACCTTTCTTCGGCCAAGGAGGCAGGCGTGGTGATGGAGAAGAAGAAACTGATGCTGCCCAACAAATTGGT 416  
5-1502    TGGACCTGGATTTGGTACCCCGCAGATGGATGGACGGAGACAAAATGGCGGTCCGATGAGTGGTAGGAGATTCGACGGACCTCGATTTGGTGGCTCCAGACCAGATGGTGCTGGAGGGAGACCTTTCTTCGGCCAAGGAGGAAGACGTGGTGATGGAGAGGAAGAAACTGATGCTGCCCAACAAATTGGT 489  
5-1503    TGGACCTGGATTTGGTACCCTGCAGATGGATGGACGGAGACAAAATGGCGGTCCGATGGGTGGTAGGAGATTCGACGGACCTCGATTTGGTGGCTCCAGACCAGATGGTGCTGGAGGGAGACCTTTCTTCGGCCAAGGAGGAAGACGTGGTGATGGAGAAGAAGAAACTGATGCTGCCCAACAAATTGGT 489  
5-1504    TGGACCTGGATTTGGTACCCCGCAGATGGATGGACGGAGACAAAATGGCGGTCCGATGGGTGGTAGGAGATTCGACGGACCTCGATTTGGTGGCTCCAGACCAGATGGTGCTGGAGGGAGACCTTTCTTCGGCCAAGGAGGAAGACGTGGTGATGGAGAAGAAGAAACTGATGCTGCCCAACAAATTGGT 489  
5-1505    TGGACCTGGATTTGGTACCCCGCAGATGGATGGACGGAGACAAAATGGCGGTCCGATGGGTGGTAGGAGATTCGACGGACCTCGATTTGGTGGCTCCAGACCAGATGGTGCTGGAGGGAGACCTTTCTTCGGCCAAGGAGGAAGACGTGGTGATGGAGAAGAAGAAACTGATGCTGCCCAACAAATTGGT 489  
5-1507    TGGACCTGGATTTGGTACCCCGCAGATGGATGGACGGAGACAAAATGGCGGTCCGATGGGTGGTAGGAGATTCGACGGACCTCGATTTGGTGGCTCCAGACCAGATGGTGCTGGAGGGAGACCTTTCTTCGGCCAAGGAGGAAGACGTGGTGATGGAGAAGAAGAAACTGATGCTGCCCAACAAATTGGT 490  
5-1509    TGGACCTGGATTTGGTACCCCGCAGATGGATGGACGGAGACAAAATGGCGGTCCGATGGGTGGTAGGAGATTCGACGGACCTCGATTTGGTGGCTCCAGACCAGATGGTGCTGGAGGGAGACCTTTCTTCGGCCAAGGAGGAAGACGTGGTGATGGAGAAGAAGAAACTGATGCTGCCCAACAAATTGGT 490  
5-1511    TGGACCTGGATTTGGTACCCCGCAGATGGATGGACGGAGACAAAATGGCGGTCCGATGGGTGGTAGGAGATTCGACGGACCTCGATTTGGTGGCTCCAGACCAGATGGTGCTGGAGGGAGACCTTTCTTCGGCCAAGGAGGAAGACGTGGTGATGGAGAAGAAGAAACTGATGCTGCCCAACAAATTGGT 490  
5-1513    TGGACCTGGATTTGGTACCCCGCAGATGGATGGACGGAGACAAAATGGCGGTCCGATGGGTGGTAGGAGATTCGACGGACCTCGATTTGGTGGCTCCAGACCAGATGGTGCTGGAGGGAGACCTTTCTTCGGCCAAGGAGGAAGACGTGGTGATGGAGAAGAAGAAACTGATGCTGCCCAACAAATTGGT 491  
5-1514    TGGACCTGGATTTGGTACCCCGCAGATGGATGGACGGAGACAAAATGGCGGTCCGATGGGTGGTAGGAGATTCGACGGACCTCGATTTGGTGGCTCCAGACCAGATGGTGCTGGAGGGAGACCTTTCTTCGGCCAAGGAGGAAGACGTGGTGATGGAGAAGAAGAAACTGATGCTGCCCAACAAATTGGT 490  
5-1508    TGGACCTGGATTTGGTACCCCGCAGATGGATGGACGGAGACAAAATGGCGGTCCGATGGGTGGTAGGAGATTCGACGGACCTCGATTTGGTGGCTCCAGACCAGATGGTGCTGGAGGGAGACCTTTCTTCGGCCAAGGAGGAAGACGTGGTGATGGAGAAGAAGAAACTGATGCTGCCCAACAAATTGGT 489  
5-1510    TGGACCTGGATTTGGTACCCCGCAGATGGATGGACGGAGACAAAATGGCGGTCCGATGGGTGGTAGGAGATTCGACGGACCTCGATTTGGTGGCTCCAGACCAGATGGTGCTGGAGGGAGACCTTTCTTCGGCCAAGGAGGAAGACGTGGTGATGGAGAAGAAGAAACTGATGCTGCCCAACAAATTGGT 489  
5-2401    TGGACCTGGATTTGGTGCCCCGGAGATGGATGGACGGAGACAAAATGGCGGTCCGATGGGTGGAAGGAGATTCGACGGACCTGGATTTGGTGGCTCCAGACCAGATGGTGCTGGAGGAAGACCTTTCTTCGGCCAAGGAGGCAGGCGTGGTGATGGAGAAGAAGAAACTGATGCTGCCCAACAAATTGGT 488  
5-2402    TGGACCTGGATTTGGTGCCCCGGAGATGGATGGACGGAGACAAAATGGCGGTCCGATGGGTGGAAGGAGATTCGACGGACCTGGATTTGGTGGCTCCAGACCAGATGGTGCTGGAGGAAGACCTTTCTTCGGCCAAAGAGGCAGGCGTGGTGATGGAGAAGAAGAAACTGATGCTGCCCAACAAATTGGT 488  
5-2404    TGGACCTGGATTTGGTGCCCCGGAGATGGATGGACGGAGACAAAATGGCGGTCCGATGGGTGGAAGGAGATTCGACGGACCTGGATTTGGTGGCTCCAGACCAGATGGTGCTGGAGGAAGACCTTTCTTCGGCCAAGGAGGCAGGCGTGGTGATGGAGAAGAAGAAACTGATGCTGCCCAACAAATTGGT 489  
5-2407    TGGACCTGGATTTGGTGCCCCGGAGATGGATGGACGGAGACAAAATGGCGGTCCGATGGGTGGAAGGAGATTCGACGGACCTGGATTTGGTGGCTCCAGACCAGTTGGTGCTGGAGGAAGACCTTTCTTCGGCCAAGGAGGCAGGCGTGGCGATGGAGAAGAAGAAACTGATGCTGCCCAACAAATTGGT 489  
5-2409    TGGGCCTGGATTTGGTGCCCCGGAGATGGATGGACGGAGACAAAATGGCGGTCCGATGGGTGGAAGGAGATTCGACGTTCCTGGATTTGGTGGCTCCAGACCAGATGGTGCTGGAGGAAGACCTTTCTTCGGCCAAGGAGGCAGGCGTGGTGATGGAGAAGAAGAAACTGATGCTGCCCAACAAATTGGT 488  
5-2410    -------------------------------------------------------------------AGATTCGACGGACCTCGATTTGGTGGCTCCAGACCAGATGGTGCTGGAGGGAGACCTTTCTTCGGCCAAGGAGGCAGGCGTGGTGATGGAGAAGAAGAAACTGATGCTGCCCAACAAATTGGT 419  
5-2411    -------------------------------------------------------------------AGATTCGACGGACCTCGATTTGGTGGCTCCAGACCAGATGGTGCTGGAGGGAGACCTTTCTTCGGCCAAGGAGGCAGGCGTGGTGATGGAGAAGAAGAAACTGATGCTGCCCAACAAATTGGT 414  
5-2413    -------------------------------------------------------------------AGATTCGACGGACCTCGATTTGGTGGCTCCAGACCAGATGGTGCTGGAGGGAGACCTTTCTTCGGCCAAGGAGGCAGGCGTGGTGATGGAGAAGAAGAAACTGATGCTGCCCAACAAATTGGT 414  
5-2414    -------------------------------------------------------------------AGATTCGACGGACCTCGATTTGGTGGCTCCAGACCAGATGGTGCTGGAGGGAGACCTTTCTTCGGCCAAGGAGGCAGGCGTGGTGATGGAGAAGAAGAAACTGATGCTGCCCAACAAATTGGT 414  
5-2415    -------------------------------------------------------------------AGATTCGACGGACCTCGATTTGGTGGCTCCAGACCAGATGGTGCTGGAGGGAGACCTTTCTTCGGCCAAGGAGGCAGGCGTGGTGATGGAGAAGAAGAAACTGATGCTGCCCAACAAATTGGT 414  
5-2403    -------------------------------------------------------------------AGATTCGACGGACCTCGATTTGGTGGCTCCAGACCAGATGGTGCTGGAGGAAGACCTTTCTTCGGCCAAGGAGGAAGACGTGGTGATGGAGAAGAAGAAACTGATGCTGCCCAACAAATTGGT 404  
5-2406    -------------------------------------------------------------------AGATTCGACGGACCTCGATTTGGTGGCTCCAGACCAGATGGTGCCGGAGGAAGACCTTTCTTCGGCCAAGGAGGCAGGCGTGGTGATGGAGAAGAAGAAACTGATGCTGCCCAACAAATTGGT 413  
5-2412    GATGGAGAAGAAGAAACTGATGCTGCCCAACAAATTGGTGATGGTCTAG--------------------------------------------------------------------------------------------------------------------------------------------- 423  
5-2408    TGGGCCTGGATTTGGTGCCCCGGAGATGGATGGACGGAGACAAAATGGCGGTCCGATGGGTGGAAGGAGATTCGACGTTCCTGGATTTGGTGGCTCCAGACCAGATGGTGCTGGAGGAAGACCTTTCTTCGGCCAAGGAGGCAGGCGTGGTGATGGAGAAGAAGAAACTGATGCTGCCCAACAAATTGGT 486  


                  580       590       600       610       620       630       640       650       660       670       680       690       700       710       720       730       740       750       760                  
          ....|....|....|....|....|....|....|....|....|....|....|....|....|....|....|....|....|....|....|....|....|....|....|....|....|....|....|....|....|....|....|....|....|....|....|....|....|....|
1-1515    GATGGTCTAGGAGGGCGCGGTCAGTTTGATGGT------------------------------------------------------------------------------------------------------------------CATGGACGTGGACATCATGGTCACCGTCAAGGTCCTCCTCAGG 492  
1-1523    GATGGTCTAGGAGGGCGCGGTCAGTTTGATGGT------------------------------------------------------------------------------------------------------------------CATGGACGTGGACATCATGGTCACCGTCAAGGTCCTCCTCAGG 492  
1-1504    GATGGTCTAGGAGGGCGCGGTCAGTTTGATGGT------------------------------------------------------------------------------------------------------------------CATGGACGTGGACATCATGGTCACCGTCAAGGTCCTCCTCAGG 492  
1-1533    GATGGTCTAGGAGGGCGCGGTCAGTTTGATGGT------------------------------------------------------------------------------------------------------------------CATGGACGTGGACATCATGGTCACCGTCAAGGTCCTCCTCAGG 467  
1-1547    GATGGTCTAGGAGGGCGCGGTCAGTTTGATGGT------------------------------------------------------------------------------------------------------------------CATTGACGTGGACATCATGGTCACCGTCAAGGTCCTCCTCAGG 492  
1-1549    GATGGTCTAGGAGGGCGCGGTCAGTTTGATGGT------------------------------------------------------------------------------------------------------------------CATTGACGTGGACATCATGGTCACCGTCAAGGTCCTCCTCAGG 494  
1-1505    GATGGTCTAGGAGGGCGCGGTCAGTTTGATGGT------------------------------------------------------------------------------------------------------------------CATTGACGTGGACATCATGGTCACCGTCAAGGTCCTCCTCAGG 492  
1-1512    GATGGTCTAGGAGGGCGCGGTCAGTTTGATGGT------------------------------------------------------------------------------------------------------------------CATTGACGTGGACATCATGGTCACCGTCAAGGTCCTCCTCAGG 492  
1-1514    GATGGTCTAGGAGGGCGCGGTCAGTTTGATGGT------------------------------------------------------------------------------------------------------------------CATTGACGTGGACATCATGGTCACCGTCAAGGTCCTCCTCAGG 492  
1-1528    GATGGTCTAGGAGGGCGCGGTCAGTTTGATGGT------------------------------------------------------------------------------------------------------------------CATTGACGTGGACATCATGGTCACCGTCAAGGTCCTCCTCAGG 492  
1-1532    GATGGTCTAGGAGGGCGCGGTCAGTTTGATGGT------------------------------------------------------------------------------------------------------------------CATTGACGTGGACATCATGGTCACCGTCAAGGTCCTCCTCAGG 492  
1-1539    GATGGTCTAGGAGGGCGCGGTCAGTTTGATGGT------------------------------------------------------------------------------------------------------------------CATTGACGTGGACATCATGGTCACCGTCAAGGTCCTCCTCAGG 492  
1-1536    GATGGTCTAGGAGGGCGCGGTCAGTTTGATGGT------------------------------------------------------------------------------------------------------------------CATTGACGTGGACATCATGGTCACCGTCAAGGTCCTCCTCAGG 491  
1-1535    GATGGTCTAGGAGGGCGCGGTCAGTTTGATGGT------------------------------------------------------------------------------------------------------------------CATTGACGTGGACATCATGGTCACCGTCAAGGTCCTCCTCAGG 492  
1-1534    GATGGTCTAGGAGGGCGCGGTCAGTTTGATGGT------------------------------------------------------------------------------------------------------------------CATTGACGTGGACATCATGGTCACCGTCAAGGTCCTCCTCAGG 492  
1-2402    GATGGTCTAGGAGGGCGCGGTCAGTTTGATGGT------------------------------------------------------------------------------------------------------------------CATGGACGTGGACATCATGGTCACCGTCAAGGTCCTCCTCAGG 492  
1-2404    GATGGTCTAGGAGGGCGCGGTCAGTTCGATGGT------------------------------------------------------------------------------------------------------------------CATGGACGTGGACATCATGGTCACCGTCAAGGTCCTCCTCATG 490  
1-2405    GATGGTCTAGGAGGGCGCGGTCAGTTTGATGGT------------------------------------------------------------------------------------------------------------------CATGGACGTGGACATCATGGTCACCGTCAAGGTCCTCCTCAGG 492  
1-2406    GATGGTCTAGGAGGGCGCGGTCAGTTTGATGGT------------------------------------------------------------------------------------------------------------------CATGGACGTGGACATCATGGTCACCGTCAAGGTCCTCCTCAGG 492  
1-2407    GATGGTCTAGGAGGGCGCGGTCAGTTCGATGGT------------------------------------------------------------------------------------------------------------------CATGGACGTGGACATCATGGTCACCGTCAAGGTCCTCCTCATG 490  
1-2412    GATGGTCTAGGAGGGCGCGGTCAGTTTGATGGT------------------------------------------------------------------------------------------------------------------CATGGACGTGGACATCATGGTCACCGTCAAGGTCCTCCTCAGG 492  
1-2413    GATGGTCTAGGAGGGCGCGGTCAGTTTGATGGT------------------------------------------------------------------------------------------------------------------CATGGACGTGGACATCATGGTCACCGTCAAGGTCCTCCTCAGG 492  
1-2416    GATGGTCTAGGAGGGCGCGGTCAGTTCGATGGT------------------------------------------------------------------------------------------------------------------CATGGACGTGGACATCATGGTCACCGTCAAGGTCCTCCTCATG 490  
1-2417    GATGGTCTAGGAGGGCGCGGTCAGTTCGATGGT------------------------------------------------------------------------------------------------------------------CATGGACGTGGACATCATGGTCACCGTCAAGGTCCTCCTCATG 490  
1-2418    GATGGTCTAGGAGGGCGCGGTCAGTTTGATGGT------------------------------------------------------------------------------------------------------------------CATGGACGTGGACATCATGGTCACCGTCAAGGTCCTCCTCAGG 492  
1-2420    GATGGTCTAGGAGGGCGCGGTCAGTTCGATGGT------------------------------------------------------------------------------------------------------------------CATGGACGTGGACATCATGGTCACCGTCAAGGTCCTCCTCATG 490  
1-2421    GATGGTCTAGGAGGGCGCGGTCAGTTCGATGGT------------------------------------------------------------------------------------------------------------------CATGGACGTGGACATCATGGTCACCGTCAAGGTCCTCCTCATG 490  
1-2422    GATGGTCTAGGAGGGCGCGGTCAGTTTGATGGT------------------------------------------------------------------------------------------------------------------CATGGACGTGGACATCATGGTCACCGTCAAGGTCCTCCTCAGG 492  
1-2425    GATGGTCTAGGAGGGCGCGGTCAGTTTGATGGT------------------------------------------------------------------------------------------------------------------CATGGACGTGGACATCATGGTCACCGTCAAGGTCCTCCTCAGG 492  
1-2426    GATGGTCTAGGAGGGCGCGGTCAGTTTGATGGT------------------------------------------------------------------------------------------------------------------CATGGACGTGGACATCATGGTCACCGTCAAGGTCCTCCTCAGG 492  
1-2427    GATGGTCTAGGAGGGCGCGGTCAGTTTGATGGT------------------------------------------------------------------------------------------------------------------CATGGACGTGGACATCATGGTCACCGTCAAGGTCCTCCTCAGG 492  
1-2428    GATGGTCTAGGAGGGCGCGGTCAGTTTGATGGT------------------------------------------------------------------------------------------------------------------CATGGACGTGGACATCATGGTCACCGTCAAGGTCCTCCTCAGG 492  
1-2430    GATGGTCTAGGAGGGCGCGGTCAGTTTGATGGT------------------------------------------------------------------------------------------------------------------CATGGACGTGGACATCATGGTCACCGTCAAGGTCCTCCTCAGG 492  
1-2431    GATGGTCTAGGAGGGCGCGGTCAGTTCGATGGT------------------------------------------------------------------------------------------------------------------CATGGACGTGGACATCATGGTCACCGTCAAGGTCCTCCTCATG 490  
1-2432    GATGGTCTAGGAGGGCGCGGTCAGTTTGATGGT------------------------------------------------------------------------------------------------------------------CATGGACGTGGACATCATGGTCACCGTCAAGGTCCTCCTCAGG 492  
1-2433    GATGGTCTAGGAGGGCGCGGTCAGTTTGATGGT------------------------------------------------------------------------------------------------------------------CATGGACGTGGACATCATGGTCACCGTCAAGGTCCTCCTCAGG 492  
1-2434    GATGGTCTAGGAGGGCGCGGTCAGTTCGATGGT------------------------------------------------------------------------------------------------------------------CATGGACGTGGACATCATGGTCACCGTCAAGGTCCTCCTCGTG 490  
1-2435    GATGGTCTAGGAGGGCGCGGTCAGTTCGATGGT------------------------------------------------------------------------------------------------------------------CATGGACGTGGACATCATGGTCACCGTCAAGGTCCTCCTCATG 490  
1-2436    GATGGTCTAGGAGGGCGCGGTCAGTTTGATGGT------------------------------------------------------------------------------------------------------------------CATGGACGTGGACATCATGGTCACCGTCAAGGTCCTCCTCAGG 490  
1-2437    GATGGTCTAGGAGGGCGCGGTCAGTTTGATGGT------------------------------------------------------------------------------------------------------------------CATGGACGTGGACATCATGGTCACCGTCAAGGTCCTCCTCAGG 492  
1-2439    GATGGTCTAGGAGGGCGCGGTCAGTTCGATGGT------------------------------------------------------------------------------------------------------------------CATGGACGTGGACATCATGGTCACCGTCAAGGTCCTCCTCATG 490  
1-2440    GATGGTCTAGGAGGGCGCGGTCAGTTCGATGGT------------------------------------------------------------------------------------------------------------------CATGGACGTGGACATCATGGTCACCGTCAAGGTCCTCCTCATG 490  
1-2441    GATGGTCTAGGAGGGCGCGGTCAGTTTGATGGT------------------------------------------------------------------------------------------------------------------CATGGACGTGGACATCATGGTCACCGTCAAGGTCCTCCTCAGG 492  
1-2442    GATGGTCTAGGAGGGCGCGGTCAGTTCGATGGT------------------------------------------------------------------------------------------------------------------CATGGACGTGGACATCATGGTCACCGTCAAGGTCCTCCTCATG 490  
1-2414    GATGGTCTAGGAGGGCCCGGTCAGTTTGATGGTCCTGGACGTCGTCATCATGGT------------------------------------------------------------------------------------------------------------------CACCGTCAAGGTCATCCTCAGG 562  
1-2424    GATGGTCTAGGAGGGCGCGGTCAGTTCAATGGT------------------------------------------------------------------------------------------------------------------CATGGACGTGGACATCATGGTCACCGTCAAGGTCCTCCTCATG 402  
1-2429    GATGGTCTAGGAGGGCGCGGTCAGTTCAATGGT------------------------------------------------------------------------------------------------------------------CATGGACGTGGACATCATGGTCACCGTCAAGGTCCTCCTCATG 401  
2-1501    GATGGTCTAGGAGGGTCCGATCGGTTTGATGGTCCTAGACGTGGTCATCATGGT------------------------------------------------------------------------------------------------------------------CACCGTCAAGGTCCTCCTCAGG 565  
2-1502    GATGGTCTAGGAGGGTCCCATCGGTTTGATGGTCCTAGACGTGGTCATCATGGT------------------------------------------------------------------------------------------------------------------CACCGTCAAGGTCCTCCTCAGG 565  
2-1505    GATGGTCTAGGAGGGTCCGATCGGTTTGATGGTCCTAGACGTGGTCATCATGGT------------------------------------------------------------------------------------------------------------------CACCGTCAAGGTCCTCCTCAGG 565  
2-1506    GATGGTCTAGGAGGGTCCGATCGGTTTGATGGTCCTAGACGTGGTCATCATGGT------------------------------------------------------------------------------------------------------------------CACCGTCAAGGTCCTCCTCAGG 565  
2-1507    GATGGTCTAGGAGGGTCCGATCGGTTTGATGGTCCTAGACGTGGTCATCATGGT------------------------------------------------------------------------------------------------------------------CACCGTCAAGGTCCTCCTCAGG 566  
2-1508    GATGGTCTAGGAGGGTCCGATCGGTTTGATGGTCCTAGACGTGGTCATCATGGT------------------------------------------------------------------------------------------------------------------CACCGTCAAGGTCCTCCTCAGG 565  
2-1509    GATGGTCTAGGAGGGTCCGATCGGTTTGATGGTCCTAGACGTGGTCATCATGGT------------------------------------------------------------------------------------------------------------------CACCGTCAAGGTCCTCCTCAGG 565  
2-1510    GATGGTCTAGGAGGGTCCGATCGGTTTGATGGTCCTAGACGTGGTCATCATGGT------------------------------------------------------------------------------------------------------------------CACCGTCAAGGTCCTCCTCAGG 557  
2-1514    GATGGTCTAGGAGGGTCCGATCGGTTTGATGGTCCTAGACGTGGTCATCATGGT------------------------------------------------------------------------------------------------------------------CACCGTCAAGGTCCTCCTCAGG 565  
2-1511    GATGGTCTAGGAGGGTCCGACCGGTTTGATGGTCCTAGACGTGGTCATCATGGT------------------------------------------------------------------------------------------------------------------CACCGTCAAGGTCCTCCTCAGG 565  
2-2401    GATGGTCTAGGAGGGCGCGGTCAGTTTGATGGTCCTAGACGTGGTCATCATGGT------------------------------------------------------------------------------------------------------------------CACCGTCAAGGTCCTCCTCAGG 489  
2-2404    GATGGTCTAGGAGGGCGCGGTCAGTTTGATGGTCCTAGACGTGGTCATCATGGT------------------------------------------------------------------------------------------------------------------CACCGTCAAGGTCCTCCTCAGG 489  
2-2406    GATGGTCTAGGAGGGCGCGGTCAGTTTGATGGT------------------------------------------------------------------------------------------------------------------CATGGACGTGGTCATCATGGTCACCGTCAAGGTCCTCCTCAGG 489  
2-2407    GATGGTCTAGGAGGGCGCGGTCAGTTTGATGGTCCTAGACGTGGTCATCATGGT------------------------------------------------------------------------------------------------------------------CACCGTCAAGGTCCTCCTCAGG 489  
2-2408    GATGGTCTAGGAGGGCGCGGTCAGTTTGATGGTCCTAGACGTGGTCATCATGGT------------------------------------------------------------------------------------------------------------------CACCGTCAAGGTCCTCCTCAGG 489  
2-2409    GATGGTCTAGGAGGGCGCGGTCAGTTTGATGGTCCTAGACGTGGTCATCATGGT------------------------------------------------------------------------------------------------------------------CACCGTCAAGGTCCTCCTCAGG 489  
2-2411    GATGGTCTAGGAGGGCGCGGTCAGTTTGATGGTCCTAGACGTGGTCATCATGGT------------------------------------------------------------------------------------------------------------------CACCGTCAAGGTCCTCCTCAGG 490  
2-2405    GATGGTCTAGGAGGGCGCGGTCAGTTTGATGGT------------------------------------------------------------------------------------------------------------------CATGGACGTCGTCATCATGGTCACCGTCAAGGTCCACCTCAGG 490  
2-2413    GATGGTCTAGGAGGGCGCGGTCAGTTTGATGGT------------------------------------------------------------------------------------------------------------------CATGGACGTCGTCATCATGGTCACCGTCAAGGTCCACCTCAGG 490  
2-2403    GATGGTCTAGGAGGGCCCGGTCAGTTTGATGGTCCTGGACGTCGTCATCATGGT---------------------------------------------------------------------------------------------------------------------------------------- 470  
2-2415    GATGGTCTAGGAGGGCCCGGTCAGTTTGATGGTCCTGGACGTCGTCATCATGGT------------------------------------------------------------------------------------------------------------------CACCGTCAAGGTCATCCTCAGG 564  
2-2414    GATGGTCCAGGACGGCCCGGTCAGTCTGATGGTCCTGGACGTCGTCATCATGGT------------------------------------------------------------------------------------------------------------------CACCGTCAAGGTCATCCTCAGG 563  
3-15-1006 GATGGTCTAGGAGGGTCCGATCGGTTTGATGGTCCTAGACGTGGTCATCATGGT------------------------------------------------------------------------------------------------------------------CACCGTCAAGGTCCTCCTCAGG 566  
3-15-4003 GATGGTCTAGGAGGGTCCGATCGGTTTGATGGTCCTAGACGTGGTCATCATGGT------------------------------------------------------------------------------------------------------------------CACCGTCAAGGTCCTCCTCAGG 565  
3-15-4004 GATGGTCTAGGAGGGTCCGATCGGTTTGATGATCCTAGACGTGGTCATCATGGT------------------------------------------------------------------------------------------------------------------CACCGTCAAGGTCCTCCTCAGG 565  
3-15-4005 GATGGTCTAGGAGGGTCCGATCGGTTTGATGGTCCTAGACGTGGTCATCATGGT------------------------------------------------------------------------------------------------------------------CACCGTCAAGGTCCTCCTCAGG 566  
3-15-4007 GATGGTCTAGGAGGGTCCGATCGGTTTGATGGTCCTAGACGTGGTCATCATGGT------------------------------------------------------------------------------------------------------------------CACCGTCAAGGTCCTCCTCAGG 564  
3-15-4011 GATGGTCTAGGAGGGTCCGATCGGTTTGATGGTCCTAGACGTGGTCATCATGGT------------------------------------------------------------------------------------------------------------------CACCGTCAAGGTCCTCCTCAGG 565  
3-15-4013 GATGGTCTAGGAGGGTCCGATCGGTTTGATGGTCCTAGACGTGGTCATCATGGT------------------------------------------------------------------------------------------------------------------CACCGTCAAGGTCCTCCTCAGG 566  
3-15-4015 GATGGTCTAGGAGGGTCCGATCGGTTTGATGGTCCTAGACGTGGTCATCATGGT------------------------------------------------------------------------------------------------------------------CACCGTCAAGGTCCTCCTCAGG 565  
3-15-4018 GATGGTCTAGGAGGGTCCGATCGGTTTGATGGTCCTAGACGTGGTCATCATGGT------------------------------------------------------------------------------------------------------------------CACCGTCAAGGTCCTCCTCAGG 565  
3-15-4019 GATGGTCTAGGAGGGTCCGATCGGTTTGATGGTCCTAGACGTGGTCATCATGGT------------------------------------------------------------------------------------------------------------------CACCGTCAGGGTCCTCCTCAGG 565  
3-15-4022 GATGGTCTAGGAGGGTCCGATCCGTTTGATGGTCCTAGACGTGGTCATCATGGT------------------------------------------------------------------------------------------------------------------CACCGTCAAGGTCCTCCTCAGG 566  
3-15-4024 GATGGTCTAGGAGGGTCCGATCGGTTTGATGGTCCTAGACGTGGTCATCATGGT------------------------------------------------------------------------------------------------------------------CACCGTCAAGGTCCTCCTCAGG 565  
3-15-4017 GATGGTCTAGGAGGGTCCGATCGGTTTGATGGTCCTAGACGTGGTCATCATGGT------------------------------------------------------------------------------------------------------------------CACCGTCAAGGTCCTCCTCAGG 566  
3-15-1003 ATGGTCTAG-GAGGGTCCGATCGGTTTGATGGTCCTAGACGTGGTCATCATGGT------------------------------------------------------------------------------------------------------------------CACCGTCAAGGTCCTCCTCAGG 564  
3-15-4021 ----------GAGGGCCCGGTCAGTTTGATGGTCCTGGACGTCGTCATCATGGT---------------------------------------------------------------------------------------------------------------------------------------- 466  
3-15-1002 GATGGTCTAGGAGGGCCCGGTCAGTTTGATGGTCCTGGACGTCGTCATCATGGT---------------------------------------------------------------------------------------------------------------------------------------- 466  
3-15-1004 ----------AAGGGCCCCGTCAGTTTGATGGTCCTGGACGTCGTCATCATGGT---------------------------------------------------------------------------------------------------------------------------------------- 466  
3-15-4008 GATGGTCTAGGAGGGTCCGATCGGTTTGATGGTCCTAGACGTGGTCATCATGGT------------------------------------------------------------------------------------------------------------------CACCGTCAAGGTCCTCCTCAGG 563  
3-24-4003 GATGGTCTAGGAGGGCGCGGTCAGTTTGATGGT------------------------------------------------------------------------------------------------------------------CATGGACGTGGACATCATGGTCACCGTCAAGGTCCTCCTCAGG 490  
3-24-4004 GATGGTCTAGAAGGGCGCGGTCAGTTTGATGGT------------------------------------------------------------------------------------------------------------------CATGGACGTGGACATCATGGTCACCGTCAAGGTCCTCCTCAGG 490  
3-24-1006 GATGGTCTAGGAGGGCGCGGTCAGTTTGATGGT------------------------------------------------------------------------------------------------------------------CATGGACGTGGACATCATGGTCACCGTCAAGGTCCTCCTCAGG 490  
3-24-4006 GATGGTCTAGGAGGGCGCGGTCAGTTTGATGGT------------------------------------------------------------------------------------------------------------------CATGGACGTGGACATCATGGTCACCGTCAAGGTCCTCCTCAGG 490  
3-24-4015 GATGGTCTAGGAGGGCGCGGGCAGTTTGATGGT------------------------------------------------------------------------------------------------------------------CATGGACGTGGACATCATGGTCACCGTCAAGGTCCTCCTCAGG 490  
3-24-4021 GATGGTCTAGGAGGGCGCGGTCAGTTTGATGGT------------------------------------------------------------------------------------------------------------------CATGGACGTGGACATCATGGTCACCGTCAAGGTCCTCCTCAGG 490  
3-24-4024 GATGGTCTAGGAGGGCGCGGTCAGTTTGATGGT------------------------------------------------------------------------------------------------------------------CATGGACGTGGACATCATGGTCACCGTCAAGGTCCTCCTCAGG 489  
3-24-4023 GATGGTCTAGGAGGGCGCGGTCAGTTTGATGGT------------------------------------------------------------------------------------------------------------------CATGGACGTGGACATCATGGTCACCGTCAAGGTCCTCCTCAGG 490  
3-24-4001 GATGGTCTAGAAGGGTCCGATCGGTTTGATGGTCCTAGACGTGGTCATCATGGT------------------------------------------------------------------------------------------------------------------CACCGTCAAGGTCCTCCTCAGG 489
[truncated: 831,323 more chars]
