# Supplementary material for: Distinctive expression patterns of 185/333 genes in the purple sea urchin, Strongylocentrotus purpuratus: an unexpectedly diverse family of transcripts in response to LPS, β-1,3-glucan, and dsRNA
Source: BMC Mol Biol. 2007 Mar 1;8:16. doi: 10.1186/1471-2199-8-16 (PMC1831783; doi:10.1186/1471-2199-8-16)
Supplement: Additional File 2 — Alignment of amino acid sequences deduced from the 185/333 cDNAs. Alignment of the translated cDNA sequences showing the 185/333 proteins and their diversity, gaps, elements, repeats, glycosylation sites, motifs and stop codons. [file 1471-2199-8-16-S2.rtf]

Additional file 2.  Alignment of amino acid sequences deduced from the 185/333 cDNAs.  The deduced amino acid sequences were derived from the nucleotide sequences shown in Additional file 1.  Gaps define 25 elements that are numbered at the top.  Five types of repeats previously defined by Nair et al. [29] are highlighted (type 1; type 2; type 3; type 4; type 5).  An RGD motif, located in element 7, is outlined with a dashed box.  Acidic stretches are indicated with red brackets along the bottom.  Defined along the top are the Glycine-rich region (orange line), the Histidine-rich region (purple line), Histidine patches (downward facing arrow [↓] located at the N-terminal side of the patch), conserved O-linked glycosylation motifs (circles at the top), and conserved motifs for N-linked glycosylation (triangles).  Element 25 is subdivided into a, b and c based on the location of the stop codon (*and outlined with a shaded box). 

                   10        20        30        40        50        60        70        80        90       100       110       120       130       140       150       160       170       180       190                           
          ....|....|....|....|....|....|....|....|....|....|....|....|....|....|....|....|....|....|....|....|....|....|....|....|....|....|....|....|....|....|....|....|....|....|....|....|....|....|
1-1515    MEVKVTLIVAIVAALAISAHAQRDFNERRGKENDTERGQGGFGGRPGGMQMGGPRQDGGPMGGRRFDGPESGAPQMEGRRQNGGPMGGR--------------------------------------------------RFDGPRFGGSRPDGAGGRPFFGQGGRRGDGEEETDAAQQIGDGLGGRGQFD 140 
1-1523    MEVKVTLIVAIVAALAISAHAQRDFNERRGKENDTERGQGGFGGRPGGMQMGGPRQDGGPMGGRRFDGPESGAPQMEGRRQNGGPMGGR--------------------------------------------------RFDGPRFGGSRPDGAGGRPFFGQGGRRGDGEEETDAAQQIGDGLGGRGQFD 140 
1-1504    MEVKVTLIVAIVAALAISAHAQRDFNERRGKENDTERGQGGFGGRPGGMQMGGPRQDGGPMGGRRFDGPESGAPQMEGRRQNGGPMGGR--------------------------------------------------RFDGPRFGGSRPDGAGGRPFFGQGGRRGDGEEETDAAQQIGDGLGGRGQFD 140 
1-1533    MEVKVTLIVAIVAALAISAHAQRDFNERRGKENDTERGQGGFGGRPGGMQMGGPRQDGGPMGGRRFDGPESGAPQMEGRRQNGGPMGGR--------------------------------------------------RFDGPRFGGSRPDGAGGRPFFGQGGRRGDGEEETDAAQQIGDGLGGRGQFD 140 
1-1547    MEVKVTLIVAIVAALAISAHAQRDFNERRGKENDTERGQGGFGGRPGGMQMGGPRQDGGPMGGGRFDGPESGAPQMEGRRQNGGPMGGR--------------------------------------------------RFDGPRFGGSRPDGAGGRPFFGQGGRRGDGEEETDAAQQIGDGLGGRGQFD 140 
1-1549    MEVKVTLIVAIVAALAISAHAQRDFNERRGKENDTERGQGGFGGRPGGMQMGGPRQDGGPMGGGRFDGPESGAPQMEGRRQNGGPMGGR--------------------------------------------------RFDGPRFGGSRPDGAGGRPFFGQGGRRGDGEEETDAAQQIGDGLGGRGQFD 140 
1-1505    MEVKVTLIVAIVAALAISAHAQRDFNERRGKENDTERGQGGFGGRPGGMQMGGPRQDGGPMGGGRFDGPESGAPQMEGRRQNGGPMGGR--------------------------------------------------RFDGPRFGGSRPDGAGGRPFFGQGGRRGDGEEETDAAQQIGDGLGGRGQFD 140 
1-1512    MEVKVTLIVAIVAALAISAHAQRDFNERRGKENDTERGQGGFGGRPGGMQMGGPRQDGGPMGGGRFDGPESGAPQMEGRRQNGGPMGGR--------------------------------------------------RFDGPRFGGSRPDGAGGRPFFGQGGRRGDGEEETDAAQQIGDGLGGRGQFD 140 
1-1514    MEVKVTLIVAIVAALAISAHAQRDFNERRGKENDTERGQGGFGGRPGGMQMGGPRQDGGPMGGGRFDGPESGAPQMEGRRQNGGPMGGR--------------------------------------------------RFDGPRFGGSRPDGAGGRPFFGQGGRRGDGEEETDAAQQIGDGLGGRGQFD 140 
1-1528    MEVKVTLIVAIVAALAISAHAQRDFNERRGKENDTERGQGGFGGRPGGMQMGGPRQDGGPMGGGRFDGPESGAPQMEGRRQNGGPMGGR--------------------------------------------------RFDGPRFGGSRPDGAGGRPFFGQGGRRGDGEEETDAAQQIGDGLGGRGQFD 140 
1-1532    MEVKVTLIVAIVAALAISAHAQRDFNERRGKENDTERGQGGFGGRPGGMQMGGPRQDGGPMGGGRFDGPESGAPQMEGRRQNGGPMGGR--------------------------------------------------RFDGPRFGGSRPDGAGGRPFFGQGGRRGDGEEETDAAQQIGDGLGGRGQFD 140 
1-1539    MEVKVTLIVAIVAALAISAHAQRDFNERRGKENDTERGQGGFGGRPGGMQMGGPRQDGGPMGGGRFDGPESGAPQMEGRRQNGGPMGGR--------------------------------------------------RFDGPRFGGSRPDGAGGRPFFGQGGRRGDGEEETDAAQQIGDGLGGRGQFD 140 
1-1536    MEVKVTLIVAIVAALAISAHAQRDFNERRGKENDTERGQGGFGGRPGGMQMGGPRQDGGPMGGGRFDGPESGAPQMEGRRQNGGPMGGR--------------------------------------------------RFDGPRFGGSRPDGAGGRPFFGQGGRRGDGEEETDAAQQIGDGLGGRGQFD 140 
1-1535    MEVKVTLIVAIVAALAISAHAQRDFNERRGKENDTERGQGGFGGRPGGMQMGGPRQDGGPMGGGRFDGPESGAPQMEGRRQNGGPMGGR--------------------------------------------------RFDGPRFGGSRPDGAGGRPFFGQGGRRGDGEEETDAAQQIGDGLGGRGQFD 140 
1-1534    MEVKVTLIVAIVAALAISAHAQRDFNERRGKENDTERGQGGFGGRPGGMQMGGPRQDGGPMGGGRFDGPESGAPQMEGRRQNGGPMGGR--------------------------------------------------RFDGPRFGGSRPDGAGGRPFFGQGGRRGDGEEETDAAQQIGDGLGGRGQFD 140 
1-2402    MEVKVTLIVAIVAALAISAHAQRDFNERRGKENDTERGQGGFGGRPGGIQMGGPRQDGGPMGGRRFDGPESGAPQMEGRRQNGGPMGGR--------------------------------------------------RFDGPRFGGSRPDGAGGRPFFGQGGRRGDGEEETDAAQQIGDGLGGRGQFD 140 
1-2404    MEVKVTLIVAIVAALAISAHAQRDFNERRGKENDTERGQGRFGGRPGGMQMGGPRQDGGPMGGRRFDGPESGAPQMEGRRQNGGPMGGR--------------------------------------------------RFDGPRFGGSRPDGAGGRPFFGQGGRRGDGEEETDAAQQIGDGLGGRGQFD 140 
1-2405    MEVKVTLIVAIVAALAISAHAQRDFNERRGKENDTERGQGGFGGRPGGMQMGGPRQDGGPMGGRRFDGPESGAPQMEGRRQNGGPMGGR--------------------------------------------------RFDGPRFGGSRPDGAGGRPFFGQGGRRGDGEEETDAAQQIGDGLGGRGQFD 140 
1-2406    MEVKVTLIVAIVAALAISAHAQRDFNERRGKENDTERGQGGFGGRPGGMQMGGPRQDGGPMGGRRFDGPESGAPQMEGRRQNGGPMGGR--------------------------------------------------RFDGPRFGGSRPDGAGGRPFFGQGGRRGDGEEETDAAQQIGDGLGGRGQFD 140 
1-2407    MEVKVTLIVAIVAALAISAHAQRDFNERRGKENDTERGQGRFGGRPGGMQMGGPRQDGGPMGGRRFDGPESGAPQMEGRRQNGGPMGGR--------------------------------------------------RFDGPRFGGSRPDGAGGRPFFGQGGRRGDGEEETDAAQQIGDGLGGRGQFD 140 
1-2412    MEVKVTLIVAIVAALAISAHAQRDFNERRGKENDTERGQGGFGGRPGGMQMGGPRQDGGPMGGRRFDGPESGAPQMEGRRQNGGPMGGR--------------------------------------------------RFDGPRFGGSRPDGAGGRPFFGQGGRRGDGEEETDAAQQIGDGLGGRGQFD 140 
1-2413    MEVKVTLIVAIVAALAISAHAQRDFNERRGKENDTERGQGGFGGRPGGMQMGGPRQDGGPMGGRRFDGPESGAPQMEGRRQNGGPMGGR--------------------------------------------------RFDGPRFGGSRPDGAGGRPFFGQGGRRGDGEEETDAAQQIGDGLGGRGQFD 140 
1-2416    MEVKVTLIVAIVAALAISAHAQRDFNERRGKENDTERGQGRFGGRPGGMQMGGPRQDGGPMGGRRFDGPESGAPQMEGRRQNGGPMGGR--------------------------------------------------RFDGPRFGGSRPDGAGGRPFFGQGGRRGDGEEETDAAQQIGDGLGGRGQFD 140 
1-2417    MEVKVTLIVAIVAALAISAHAQRDFNERRGKENDTERGQGRFGGRPGGMQMGGPRQDGGPMGGRRFDGPESGAPQMEGRRQNGGPMGGR--------------------------------------------------RFDGPRFGGSRPDGAGGRPFFGQGGRRGDGEEETDAAQQIGDGLGGRGQFD 140 
1-2418    MEVKVTLIVAIVAALAISAHAQRDFNERRGKENDTERGQGGFGGRPGGMQMGGPRQDGGPMGGRRFDGPESGAPQMEGRRQNGGPMGGR--------------------------------------------------RFDGPRFGGSRPDGAGGRPFFGQGGRRGDGEEETDAAQQIGDGLGGRGQFD 140 
1-2420    MEVKVTLIVAIVAALAISAHAQRDFNERRGKENDTERGQGRFGGRPGGMQMGGPRQDGGPMGGRRFDGPESGAPQMEGRRQNGGPMGGR--------------------------------------------------RFDGPRFGGSRPDGAGGRPFFGQGGRRGDGEEETDAAQQIGDGLGGRGQFD 140 
1-2421    MEVKVTLIVAIVAALAISAHAQRDFNERRGKENDTERGQGRFGGRPGGMQMGGPRQDGGPMGGRRFDGPESGAPQMEGRRQNGGPMGGR--------------------------------------------------RFDGPRFGGSRPDGAGGRPFFGQGGRRGDGEEETDAAQQIGDGLGGRGQFD 140 
1-2422    MEVKVTLIVAIVAALAISAHAQRDFNERRGKENDTERGQGGFGGRPGGMQMGGPRQDGGPMGGRRFDGPESGAPQMEGRRQNGGPMGGR--------------------------------------------------RFDGPRFGGSRPDGAGGRPFFGQGGRRGDGEEETDAAQQIGDGLGGRGQFD 140 
1-2425    MEVKVTLIVAIVAALAISAHAQRDFNERRGKENDTERGQGGFGGRPGGMQMGGPRQDGGPMGGRRFDGPESGAPQMEGRRQNGGPMGGR--------------------------------------------------RFDGPRFGGSRPDGAGGRPFFGQGGRRGDGEEETDAAQQIGDGLGGRGQFD 140 
1-2426    MEVKVTLIVAIVAALAISAHAQRDFNERRGKENDTERGQGGFGGRPGGMQMGGPRQDGGPMGGRRFDGPESGAPQMEGRRQNGGPMGGR--------------------------------------------------RFDGPRFGGSRPDGAGGRPFFGQGGRRGDGEEETDAAQQIGDGLGGRGQFD 140 
1-2427    MEVKVTLIVAIVAALAISAHAQRDFNERRGKENDTERGQGGFGGRPGGMQMGGPRQDGGPMGGRRFDGPESGAPQMEGRRQNGGPMGGR--------------------------------------------------RFDGPRFGGSRPDGAGGRPFFGQGGRRGDGEEETDAAQQIGDGLGGRGQFD 140 
1-2428    MEVKVTLIVAIVAALAISAHAQRDFNERRGKENDTERGQGGFGGRPGGMQMGGPRQDGGPMGGRRFDGPESGAPQMEGRRQNGGPMGGR--------------------------------------------------RFDGPRFGGSRPDGAGGRPFFGQGGRRGDGEEETDAAQQIGDGLGGRGQFD 140 
1-2430    MEVKVTLIVAIVAALAISAHAQRDFNERRGKENDTERGQGGFGGRPGGMQMGGPRQDGGPMGGRRFDGPESGAPQMEGRRQNGGPMGGR--------------------------------------------------RFDGPRFGGSRPDGAGGRPFFGQGGRRGDGEEETDAAQQIGDGLGGRGQFD 140 
1-2431    MEVKVTLIVAIVAALAISAHAQRDFNERRGKENDTERGQGRFGGRPGGMQMGGPRQDGGPMGGRRFDGPESGAPQMEGRRQNGGPMGGR--------------------------------------------------RFDGPRFGGSRPDGAGGRPFFGQGGRRGDGEEETDAAQQIGDGLGGRGQFD 140 
1-2432    MEVKVTLIVAIVAALAISAHAQRDFNERRGKENDTERGQGGFGGRPGGMQMGGPRQDGGPMGGRRFDGPESGAPQMEGRRQNGGPMGGR--------------------------------------------------RFDGPRFGGSRPDGAGGRPFFGQGGRRGDGEEETDAAQQIGDGLGGRGQFD 140 
1-2433    MEVKVTLIVAIVAALAISAHAQRDFNERRGKENDTERGQGGFGGRPGGMQMGGPRQDGGPMGGRRFDGPESGAPQMEGRRQNGGPMGGR--------------------------------------------------RFDGPRFGGSRPDGAGGRPFFGQGGRRGDGEEETDAAQQIGDGLGGRGQFD 140 
1-2434    MEVKVTLIVAIVAALAISAHAQRDFNERRGKENDTERGQGRFGGRPGGMQMGGPRQDGGPMGGRRFDGPESGAPQMEGRRQNGGPMGGR--------------------------------------------------RFDGPRFGGSRPDGAGGRPFFGQGGRRGDGEEETDAAQQIGDGLGGRGQFD 140 
1-2435    MEVKVTLIVAIVAALAISAHAQRDFNERRGKENDTERGQGRFGGRPGGMQMGGPRQDGGPMGGRRFDGPESGAPQMEGRRQNGGPMGGR--------------------------------------------------RFDGPRFGGSRPDGAGGRPFFGQGGRRGDGEEETDAAQQIGDGLGGRGQFD 140 
1-2436    MEVKVTLIVAIVAALAISAHAQRDLNERRGKENDTERGQGGFGGRPGGMQMGGPRQDGGPMGGRRFDGPESGAPQMEGRRQNGGPMGGR--------------------------------------------------RFDGPRFGGSRPDGAGGRPFFGQGGRRGDGEEETDAAQQIGDGLGGRGQFD 140 
1-2437    MEVKVTLIVAIVAALAISAHAQRDFNERRGKENDTERGQGGFGGRPGGMQMGGPRQDGGPMGGRRFDGPESGAPQMEGRRQNGGPMGGR--------------------------------------------------RFDGPRFGGSRPDGAGGRPFFGQGGRRGDGEEETDAAQQIGDGLGGRGQFD 140 
1-2439    MEVKVTLIVAIVAALAISAHAQRDFNERRGKENDTERGQGRFGGRPGGMQMGGPRQDGGPMGGRRFDGPESGAPQMEGRRQNGGPMGGR--------------------------------------------------RFDGPRFGGSRPDGAGGRPFFGQGGRRGDGEEETDAAQQIGDGLGGRGQFD 140 
1-2440    MEVKVTLIVAIVAALAISAHAQRDFNERRGKENDTERGQGRFGGRPGGMQMGGPRQDGGPMGGRRFDGPESGAPQMEGRRQNGGPMGGR--------------------------------------------------RFDGPRFGGSRPDGAGGRPFFGQGGRRGDGEEETDAAQQIGDGLGGRGQFD 140 
1-2441    MEVKVTLIVAIVAALAISAHAQRDFNERRGKENDTERGQGGFGGRPGGMQMGGPRQDGGPMGGRRFDGPESGAPQMEGRRQNGGPMGGR--------------------------------------------------RFDGPRFGGSRPDGAGGRPFFGQGGRRGDGEEETDAAQQIGDGLGGRGQFD 140 
1-2442    MEVKVTLIVAIVAALAISAHAQRDFNERRGKENDTERGQGRFGGRPGGMQMGGPRQDGGPMGGRRFDGPESGAPQMEGRRQNGGPMGGR--------------------------------------------------RFDGPRFGGSRPDGAGGRPFFGQGGRRGDGEEETDAAQQIGDGLGGRGQFD 140 
1-2414    M-VKVTLIIAIVAALAISAHAQRDYNELRGNKNGRERGQGRFGGRPGGMQMGGSRQDGGPMGGRRFDGPDSGAPQMDGRRQDGGPMGGR-------------------------RFDGPGFGAPEMDGRRQNGGPMGGRRFDGPGFGGSRPDGAGGRPFFGQGGRRGDGEEETDAAQQIGDGLGGPGQFD 164 
1-2424    MEVKVTLIVAIVAALAISAHAQRDFNERRGKENDTERGQGRFGGRPGGMQTKWRSDGW*                                                                                                                                     58  
1-2429    MEVKVTLIVAIVAALAISAHAQRDFNERRGKENDTERGQGRFGGRPGGMQTKWRSDGW*                                                                                                                                     58  
2-1501    MEVKVTLIVAIVAALAISAHTQRDYNERRGNENGRERGQGRFGGRPGGMQMGGPRTDGGPMGGR-------------------------RFDGHGFGAPPMGGPRQDGGPMGGRRFDGPGFGTPQMDGRRQNGGPMGGRRFDGPRFGGSDQMVTGGRPFFGQGGRRGDGEEETDAAQQIGDGLGGSDRFD 165 
2-1502    MEVKVTLIVAIVAALAISAHTQRDYNERRGNENGRERGQGRFGGRPGGMQMGGPRQDGGPMGGR-------------------------RFDGHGFGAPPMGGPRQDGGLMGGRRLDGPGFGTPQMDGRRQNGGPMGGRRFDGPRFGGSRPDGAGGRPFFGQGGRRGDGEEETDAAQQIGDGLGGSHRFD 165 
2-1505    MEVKVTLIVAIVAALAISAHTQRDYNERRGNENGRERGQGRFGGRPGGMQMGGPRQDGGPMGGR-------------------------RFDGHGFGAPPMGGPRQDGGPMGGRRFDGPGFGTPQMDGRRQNGGPMGGRRFDGPRFGGSRPDGAGGRPFFGQGGRRGDGEEETDAAQQIGDGLGGSDRFD 165 
2-1506    MEVKVTLIVAIVAALAISAHTQRDYNERRGNENGRERGQGRFGGRPGGMQMGGPRQDGGPMGGR-------------------------RFDGHGFGAPPMGGPRQDGGPMGGRRFDGPGFGTPQMDGRRQNGGPMGGRRFDGPRFGGSRPDGAGGRPFFGQGGRRGDGEEETDAAQQIGDGLGGSDRFD 165 
2-1507    MEVKVTLIVAIVAALAISAHTQRDYNERRGNENGRERGQGRFGGRPGGMQMGGPRQDGGPMGGR-------------------------RFDGHGFGAPPMGGPRQDGGPMGGRRFDGPGFGTPQMDGRRQNGGPMGGRRFDGPRFGGSRPDGAGGRPFFGQGGRRGDGEEETDAAQQIGDGLGGSDRFD 165 
2-1508    MEVKVTLIVAIVAALAISAHTQRDYNERRGNENGRERGQGRFGGRPGGMQMGGPRQDGGPMGGR-------------------------RFDGHGFGAPPMGGPRQDGGPMGGRRFDGPGFGTPQMDGRRQNGGPMGGRRFDGPRFGGSRPDGAGGRPFFGQGGRRGDGEEETDAAQQIGDGLGGSDRFD 165 
2-1509    MEVKVTLIVAIVAALAISAHTQRDYNERRGNENGRERGQGRFGGRPGGMQMGGPRQDGGPMGGR-------------------------RFDGHGFGAPPMGGPRQDGGPMGGRRFDGPGFGTPQMDGRRQNGGPMGGRRFDGPRFGGSRPDGAGGRPFFGQGGRRGDGEEETDAAQQIGDGLGGSDRFD 165 
2-1510    MEVKVTLIVAIVAALAISAHTQRDYNERRGNENGRERGQGRFGGRPGGMQMGGPRQDGGPMGGR-------------------------RFDGHGFGAPPMGGPRQDGGPMGGRRFDGPGFGTPQMDGRRQNGGPMGGRRFDGPRFGGSRPDGAGGRPFFGQGGRRGDGEEETDAAQQIGDGLGGSDRFD 165 
2-1514    MEVKVTLIVAIVAALAISAHTQRDYNERRGNENGRERGQGRFGGRPGGMQMGGPRQDGGPMGGR-------------------------RFDGHGFGAPPMGGPRQDGGPMGGRRFDGPGFGTPQMDGRRQNGGPMGGRRFDGPRFGGSRPDGAGGRPFFGQGGRRGDGEEETDAAQQIGDGLGGSDRFD 165 
2-1511    MGVKVTLIVAIVAALAISAHTQRDYNERRGNENGRERGQGRFGGRPGGMQMGGPRQDGGPMGGR-------------------------RFDGHGFGAPPMGGPRQDGGPMGGRRFDGPGFGTPQMDGRRQNGGPMGGRRFDGPRFGGSRPDGAGGRPFFGQGGRRGDGEEETDAAQQIGDGLGGSDRFD 165 
2-2401    M-VKVTLIVAIVAALAISAHAQRDFNERRGKENDTERGQGGFGGRPGGMQMGGPRQDGGPMGGRRFDGPESGAPQMEGRRQNGGPMGGR--------------------------------------------------GFDGPGFGGSRPDGAGGRPFFGQGGRRGDGEEETDAAQQIGDGLGGRGQFD 139 
2-2404    M-VKVTLIVAIVAALAISAHAQRDFNERRGKENDTERGQGGFGGRPGGMQMGGPRQDGGPMGGRRFDGPESGAPQMEGRRQNGGPMGGR--------------------------------------------------RFDGPGFGGSRPDGAGGRPFFGQGGRRGDGEEETDAAQQIGDGLGGRGQFD 139 
2-2406    M-VKVTLIVAIVAALAISAHAERDFNERRGKENGRERGQGGFGGRPGGMQTGSPRQDGGPMGGMRFDGPESGAPQMDGRRQNGGPMGGR--------------------------------------------------RFDGPRFGGSRPDGAGGRPFFGQGGRRGDGKEETDAAQQIGDGLGGRGQFD 139 
2-2407    M-VKVTLIVAFVAALAISAHAQRDFNERRGKENDTERGQGGFGGRPGGMQMGGPRQDGGPMGGRRFDGPESGAPQMEGRRQNGGPMGGR--------------------------------------------------RFDGPGFGGSRPDGAGGRPFFGQGGRRGDGEEETDAAQQIGDGLGGRGQFD 139 
2-2408    M-VKVTLIVAIVAALAISAHAQRDFNERRGKENDTERGQGGFGGRPGGMQMGGPRQDGGPMGGRRFDGPESGAPQMEGRRQNGGPMGGR--------------------------------------------------RFDGPGFGGSRPDGAGGRPFFGQGGRRGDGEEETDAAQQIGDGLGGRGQFD 139 
2-2409    M-VKVTLIVAIVAALAISAHAQRDFNERRGKENDTERGQGGFGGRPGGMQMGGPRQDGGPMGGRRFDGPESGAPQMEGRRQNGGPMGGR--------------------------------------------------RFDGPGFGGSRPDGAGGRPFFGQGGRRGDGEEETDAAQQIGDGLGGRGQFD 139 
2-2411    M-VKVTLIVAIVAALAISAHAQRDFNERRGKENDTERGQGGFGGRPGGMQMGGPRQDGGPMGGRRFDGPESGAPQMEGRRQNGGPMGGR--------------------------------------------------RFDGPGFGGSRPDGAGGRPFFGQGGRRGDGEEETDAAQQIGDGLGGRGQFD 139 
2-2405    MEVKATLIVAIVAALAISAHARRDFNERRGNENGRERGQGRFGARPGGMQMGGSRQDGGPVVGR--------------------------------------------------RFDGPGFGAPHMDGRRQNGGPMGGRRFDGPGFGGSRPDGAGGRPFFGQGGRRGDGEEETDAAQQIGDGLGGRGQFD 140 
2-2413    MEVKATLIVAIVAALAISAHARRDFNERRGNENGRERGQGRFGARPGGMQMGGSRQDGGPVVGR--------------------------------------------------RFDGPGFGAPHMDGRRQNGGPMGGRRFDGPGFGGSRPDGAGGRPFFGQGGRRGDGEEETDAAQQIGDGLGGRGQFD 140 
2-2403    MEVKVTLIVAIVAALAISAHARRDFNERRGKENGRERGQGGFGGRPGGMQTGSPRQDGGPMGGMRFDGPESGAPQMDGRRQNGGPMGGR--------------------------------------------------RFDGPRFGGSRPDGAGGRPFFGQGGRRGDGEEETDAAQQIGDGLGGPGQFD 140 
2-2415    M-VKVTLIVAIVAALAISAHAQRDYNELRGNKNGRERGQGRFGGRPGGMQMGGSRQDGGPMGGRRFDGPDSGAPQMDGRRQDGGPMGGR-------------------------RFDGPGFGAPEMDGRRQNGGPMGGRRFDGPGFGGSRPVGAGGRPFFGQGGRRGDEEEETDAAQQIGDGLGGPGQFD 164 
2-2414    M-VKVTLIVAIVAALAISAHAQRDYNELRGNKNGREXGQGRFGGRPGGMQMGGSRQDGGPMGGRRFDGPDSGAPQMDGRRQDGGPMGGR-------------------------RFDGPGFGAPEMDGRRQNGGPMGGRRFDGPGFGGSRPDGAGGRPFFGQGGRRGDGEEETDAAQQIGDGPGRPGQSD 164 
3-15-1006 MEVKVTLIVAIVAALAISAHTQRDYNERRGNENGRERGQGRFGGRPGGMQMGGPRQDGGPMGGR-------------------------RFDGHGFGAPPMGGPRQDGGPMGGRRFDGPGFGTPQMDGRRQNGGPMGGRRFDGPRFGGSRPDGAGGRPFFGQGGRRGDGEEETDAAQQIGDGLGGSDRFD 165 
3-15-4003 MEVKVTLIVAIVAALAISAHTQRDYNERRGNENGRERGQGRFGGRPGGMQMGGPRQDGGPMGGR-------------------------RFDGHGFGAPPMGGPRQDGGPMGGRRFDGPGFGTPQMDGRRQNGGPMGGRRFDGPRFGGSRPDGAGGRPFFGQGGRRGDGEEETDAAQQIGDGLGGSDRFD 165 
3-15-4004 MEVKVTLIVAIVAALAISAHTQRDYNERRGNENGRERGQGRFGGRPGGMQMGGPRQDGGPMGGR-------------------------RFDGHGFGAPPMGGPRQDGGPMGGRRFDGPGFGTPQMDGRRQNGGPMGGRRFDGPRFGGSRPDGAGGRPFFGQGGRRGDGEEDTDAAQQIGDGLGGSDRFD 165 
3-15-4005 MEVKVTLIVAIVAALAISAHTQRDYNERRGNENGRERGQGRFGGRPGGMQMGGPRQDGGPMGGR-------------------------RFDGHGFGAPPMGGPRQDGGPMGGRRFDGPGFGTPQMDGRRQNGGPMGGRRFDGLRFGGSRPDGAGGRPFFGQGGRRGDGEEETDAAQQIGDGLGGSDRFD 165 
3-15-4007 MEVKVTLIVAIVAALAISAHTQRDYNERRGNENGRERGQGRFGGRPGGMQMGGPRQDGGPMGGR-------------------------RFDGHGFGAPPMGGPRQDGGPMGGRRFDGPGFGTPQMDGRRQNGGPMGGRRFDGPRFGGSRPDGAGGRPFFGQGGRRGDGEEETDAAQQIGDGLGGSDRFD 165 
3-15-4011 MEVKVTLIVAIVAALAISAHTQRDYNERRGNENGRERGQGRFGGRPGGMQMGGPRQDGGPMGGR-------------------------RFDGHGFGAPPMGGPRQDGGPMGGRRFDGPGFGTPQMDGRRQNGGPMGGRRFDGPRFGGSRPDGAGGRPFFGQGGRRGDGEEETDAAQQIGDGLGGSDRFD 165 
3-15-4013 MEVKVTLIVAIVAALAISAHTQRDYNERRGNENGRERGQGRFGGRPGGMQMGGPRQDGGPMGGR-------------------------RFDGHGFGAPPMGGPRQDGGPMGGRRFDGPGFGTPQMDGRRQNGGPMGGRRFDGLRFGGSRPDGAGGRPFFGQGGRRGDGEEETDAAQQIGDGLGGSDRFD 165 
3-15-4015 MEVKVTLIVAIVAALAISAHTQRDYNERRGNENGRERGQGRFGGRPGGMQMGGPRQDGGPMGGR-------------------------RFDGHGFGAPPMGGPRQDGGPMGGRRFDGPGFGTPQMDGRRQNGGPMGGRRFDGPRFGGSRPDGAGGRPFFGQGGRRGDGEEETDAAQQIGDGLGGSDRFD 165 
3-15-4018 MEVKVTLIVAIVAALAISAHTQRDYNERRGNENGRERGQGRFGGRPGGMQMGGPRQDGGPMGGR-------------------------RFDGHGFGAPPMGGPRQDGGPMGGRRFDGPGFGTPQMDGRRQNGGPMGGRRFDGPRFGGSRPDGAGGRPFFGQGGRRGDGEEETDAAQQIGDGLGGSDRFD 165 
3-15-4019 MEVKVTLIVAIVAALAISAHTQRDYNERRGNENGRERGQGRFGGRPGGMQMGGPRQDGGPMGGR-------------------------RFDGHGFGAPPMGGPRQDGGPMGGRRFDGPGFGTPQMDGRRQNGGPMGGRRFDGPRFGGSRPDGAGGRPFFGQGGRRGDGEEETDAAQQIGDGLGGSDRFD 165 
3-15-4022 MEVKVTLIVAIVAALAISAHTQRDYNERRGNENGRERGQGRFGGRPGGMQMGGPRQDGGPMGGR-------------------------RFDGHGFGAPPMGGPRQDGGPMGGRRFDGPGFGTPQMDGRRQNGGPMGGRRFDGPRFGGSRPDGAGGRPFFGQGGRRGDGEEETDAAQQIGDGLGGSDPFD 165 
3-15-4024 MEVKVTLIVAIVAALAISAHTQRDYNERRGNENGRERGQGRFGGRPGGMQMGGPRQDGGPMGGR-------------------------RFDGHGFGAPPMGGPRQDGGPMGGRRFDGPGFGTPQMDGRRQNGGPMGGRRFDGPRFGGSRPDGAGGRPFFGQGGRRGDGEEETDAAQQIGDGLGGSDRFD 165 
3-15-4017 MEVKVTLIVAIVAALAISAHTQRDYNERRGNENGRERGQGRFGGRPGGMQMGGPRQDGGPMGGR-------------------------RFDGHGFGAPPMGGPRQDGGPMGGRRFDGPGFGTPQMDGRRQNGGPMGGRRFDGPRFGGSRPDGAGGRPFFGQGGRRGDGEEETDAAQQIGDGLGGSDRFD 165 
3-15-1003 MEVKVTLIVAIVAALAISAHTQRDYNERRGNENGRERGQGRFGGRPGGMQMGGPRQDGGPMGGR-------------------------RFDGHGFGAPPMGGPRQDGGPMGGRRFDGPGFGTPQMDGRRQMAVRWVVGDSTDLDLVAPDQMVLEGDLSSAKEEDVVMEKKKLMLPNKLVMV*        157 
3-15-4021 M-VKVTLIVAIVAALAISAHAERDFNELRGKENGRERGQGGFGGRPGGMQTGSPRQDGGPMGGMRFDGPESGAPQMDGRRQMAVRWVVGDSTDLDLVAPDQMVLEEDLSSAKEAGVVMEKKKLMLPNKLVMV*                                                          131 
3-15-1002 M-VKVTLIVAIVAALAISAHAERDFNERRGKENGRERGQGGFGGRPGGMQTGSPRQDGGPMGGMRFDGPESGAPQMDGRRQMAVRWVVGDSTDLDLVAPDQMVLEEDLSSAKEAGVVMEKK*                                                                     120 
3-15-1004 M-VKVTLIVAIVAALAISAHAERDFNERRGKENGRERGQGGFGGRPGGMQTGSPRQDGGPMGGMRFDGPESGAPQMDGRRQMAVRWVVGDSTDLDLVAPDQMVLEEDLSSAKEAGVVMEKKKLILPNKLVMV*                                                          131 
3-15-4008 MEVKVTLIVAIVAALAISAHTQRDYNERRGNENGRERTRSLWRKAWWNADGWTKARWRSDGW*                                                                                                                                62  
3-24-4003 MEVKATLIVAILAVLAISAHAQRDFNERRGKENDTERGQGGFGGRPGGMQMGGPRQDGGPMGGRRFDGPESGAPQMEGRRQNGGPMGGR--------------------------------------------------RFDGPRFGGSRPDGAGGRPFFGQGGRRGDGEEETDAAQQIGDGLGGRGQFD 140 
3-24-4004 MEVKVTLIVAIVAALAISAHAQRDFNERRGKENDTERGQGGFGGRPGGMQMGGPRQDGGPMGGRRFDGPESGAPQMEGRRQNGGPMGGR--------------------------------------------------RFDGPRFGGSRPDGARGRPFFGQGGRRGDGEEETDAAQQIGDGLEGRGQFD 140 
3-24-1006 MEVKATLIVAILAVLAISAHAQRDFNERRGKENDTERGQGGFGGRPGGMQMGGPRQDGGPMGGRRFDGPESGAPQMEGRRQNGGPMGGR--------------------------------------------------RFDGPRFGGSRPDGAGGRPFFGQGGRRGDGEEETDAAQQIGDGLGGRGQFD 140 
3-24-4006 MEVKVTLIVAIVAALAISAHAQRDFNERRGKENDTERGQGGFGGRPGGMQMGGPRQDGGPMGGRRFDGPESGAPQMEGRRQNGGPMGGR--------------------------------------------------RFDGPRFGGSRPDGAGGRPFFGQGGRRGDGEEETDAAQQIGDGLGGRGQFD 140 
3-24-4015 MEVKATLIVAILAVLAISAHAQRDFNERRGKENDTERGQGGFGGRPGGMQMGGPRQDGGPMGGRRFDGPESGAPQMEGRRQNGGPMGGR--------------------------------------------------RFDGPRFGGSRPDGAGGRPFFGQGGRRGDGEEETDAAQQIGDGLGGRGQFD 140 
3-24-4021 MEVKVTLIVAIVAALAISAHAQRDFNERRGKENDTERGQGGFGGRPGGMQMGGPRQDGGPMGGRRFDGPESGAPQMEGRRQNGGPMGGR--------------------------------------------------RFDGPRFGGSRPDGAGGRPFFGQGGRRGDGEEETDAAQQIGDGLGGRGQFD 140 
3-24-4024 MEVKVTLIVAIVAALAISAHAQRDFNERRGKENDTERGQGGFGGRPGGMQMGGPRQDGGPMGGRRFDGPESGAPQMEGRRQNGGPMGGR--------------------------------------------------RFDGPRFGGSRPDGAGGRPFFGQGGRRGDGEEETDAAQQIGDGLGGRGQFD 140 
3-24-4023 MEVKATLIVAILAVLAISAHAQRDFNERRGKENDTERGQGGFGGRPGGMQMGGPRQDGGPMGGRRFDGPESGAPQMEGRRQNGGPMGGR--------------------------------------------------RFDGPRFGGSRPDGAGGRPFFGQGGRRGDGEEETDAAQQIGDGLGGRGQFD 140 
3-24-4001 M-VKVTLIVAIVAALAISAHARRDFNERRGKENGTERGQGGFGGRPGGMQTGSPRQDGGPMGGRRFDGPESGAPQMDGRRQNGGPMGGR--------------------------------------------------RFDGPRFGGSRPDGAGGRPFFGQGGRRGDGEEETDAAQQIGDGLEGSDRFD 139 
3-24-4019 M-VKVTLIVAIVAALAISAHARRDFNERRGKENGTERGQGGFGGRPGGMQTGSPRQDGGPMGGRRFDGPESGAPQMDGRRQNGGPMGGR--------------------------------------------------RFDGPRFGGSRPDGAGGRPFFGQGGRRGDGEEETDAAQQIGDGLEGSDRFD 139 
3-24-1003 M-VKVTLIVAIVAALAISAHARRDFNERRGKENGRERGQGGFGGRPGGMQTGSPRQDGGPMGGMRFDGPESGAPQMDGRRQNGGPMGGR--------------------------------------------------RFDGPRFGGSRPDGAGGRPFFGQGGRRSDGEEETDAAQQIGDGLGEPGQFD 139 
3-24-4017 M-VKVTLIVAIVAALAISAHAQRDYNELRGNKNGRERGQGRFGGRPGGMQMGGLRQDGGPMGGRRFDGPDSGAPQMDGRRQDGGPMGGR-------------------------RFDGNGFGAPEMDGRRQNGGPMGGRRFDGPGSGGSRPDGAGGRPFFGQGGRRGDGEEETDAAQQIGDGLGGPGQFD 164 
3-24-4016 M-VKVTLIVAIVAALAISAHAQRDYNELRGNKNGRERGQGRFGRRPGGMQMGGLRQDGGPMGGRRFDGPDSGAPQMDGRRQDGGPMGGR-------------------------RFDGPGFGAPEMDGRRQNGGPMGGRRFDGPGFGGSRPDGAGGRPFFGQGGRRGDGEEETDAAQQIGDGLGGPGQFD 164 
3-24-4011 MEVKVTLIVAIVAALAISAHTQRDYNERRGNENGRERGQGRFGGRPGGMQMGGPRQDGGPMGGR-------------------------RFDGHGFGAPPMGGPRQDGGPMGGRRFDGPGFGTPQMDGRRQNGGPMGGRRFDGPRFGGSRPDGAGGRPFFGQGGRPGDGEEETDAAQQIGDGLGGSDRFD 165 
3-24-4005 MEVKVTLIVAIVAALAISAHTQRDYNERRGNENGRERGQGRFGGRPGGMQMGGPRQDGGPMGGR-------------------------RFDGHGFGAPPMGGPRQDGGPMGGRRFDGPGFGTPQMDGRRQNGGPMGGRRFDGPRFGGSRPDGAGGRPFFGQGGRRGDGEEETDAAQQIGDGLGGSDRFD 165 
3-24-4018 MEVKVTLIVAIVAALAISAHTQRDYNERRGNENGRERGQGRFGGRPGGMQMGGPRQDGGPMGGR-------------------------RFDGHGFGAPPMGGPRQDGGPMGGRRFDGPGFGTPQMDGRRQNGGPMGGRRFDGPRFGGSRPDGAGGRPFFGQGGRRGDGEEETDAAQQIGDGLGGSDRFD 165 
3-24-4022 MEVKVTLIVAIVAALAISAHTQRDYNERRGNENGRERGQGRFGGRPGGMQMGGPRQDGGPMGGR-------------------------RFDGHGFGAPPMGGPRQDGGPMGGRRFDGPGFGTPQMMDGDKMAVRWVVGDSTDLDLVAPDQMVLEGDLSSAKEEDVVMEKKKLMLPNKLVMV*        157 
6-2415    M-VKVTLIVAIVAALAISAHARRDFNERRGKENGRERGQGGFGGRPGGMQTGSPRQDGGPMDGMRFDGPESGAPQMEGRRQNGGPMGGR--------------------------------------------------RFDGPRFGGSRPDGAGGRPFFGQGGRRGDGEEETDAAQQIGDGLGGPGQFD 139 
6-2426    M-VKVTLIVAIVAALAISAHARRDFNERRGKENGRERGQGGFGGRPGGMQTGSPRQDGGPMDGMRFDGPESGAPQMEGRRQNGGPMGGR--------------------------------------------------RFDGPRFGGSRPDGAGGRPFFGQGGRRGDGEEETDAAQQIGDGLGGPGQFD 139 
6-2446    M-VKVTLIVAIVAALAISAHARRDFNERRGKENGRERGQGGFGGRPGGMQTGSPRQDGGPMDGMRFDGPESGAPQMEGRRQNGGPMGGR--------------------------------------------------RFDGPRFGGSRPDGAGGRPFFGQGGRRGDGEEETDAAQQIGDGLGGPGQFD 139 
6-2401    MEVKVTLIVAIVAALAISAHAQRDFNERRGKENDTERGQGGFGGRPGGMQMGGPRQDGGQMGGRRFDGPESGAPQMEGRRQNGGPMGGR--------------------------------------------------RFDGPRFGGSRPDGAGGRPFFGQGGRRGDGEEETDAAQQIGDGLGGRGQFD 140 
6-2402    MEVKVTLIVAIVAALAISAHAQRDFNERRGKENDTERGQGGFGGRPGGMQMGGPRQDGGPMGGRRFDGPESGAPQMEGRRQNGGPMGGR--------------------------------------------------RFDGPRFGGSRPDGAGGRPFFGQGGRRGDGEEETDAAQQIGDGLGGRGQFD 140 
6-2404    MEVKVTLIVAIVAALAISAHAQRDFNERRGKENDTERGQGGFGGRPGGMQMGGPRQDGGPMGGRRFDGPESGAPQMEGRRQNGGPMGGR--------------------------------------------------RFDGPRFGGSRPDGAGGRPFFGQGGRRGDGEEETDAAQQIGDGLGGRGQFD 140 
6-2407    MEVKVTLIVAIVAALAISAHAQRDFNERRGKENDTERGQGGFGGRPGGMQMGGPRQDGGQMGGRRFDGPESGAPQMEGRRQNGGPMGGR--------------------------------------------------RFDGPRFGGSRPDGAGGRPFFGQGGRRGDGEEETDAAQQIGDGLGGRGQFD 140 
6-2408    MEVKVTLIVAIVAALAISAHAQRDFNERRGKENDTERGQGGFGGRPGGMQMGGPRQDGGPMGGRRFDGPESGAPQMEGRRQNGGPMGGR--------------------------------------------------RFDGPRFGGSRPDGAGGRPFFGQGGRRGDGEEETDAAQQIGDGLGGRGQFD 140 
6-2409    MEVKVTLIVAIVAALAISAHAQRDFNERRGKENDTERGQGGFGGRPGGMQMGGPRQDGGQMGGRRFDGPESGAPQMEGRRQNGGPMGGR--------------------------------------------------RFDGPRFGGSRPDGAGGRPFFGQGGRRGDGEEVTDAAQQIGDGLGGRGQFD 140 
6-2410    MEVKVTLIVAIVAALAISAHAQRDFNERRGKENDTERGQGGFGGRPGGMQMGGPRQDGGPMGGRRFDGPESGAPQMEGRRQNGGPMGGR--------------------------------------------------RFDGPRFGGSRPDGAGGRPFFGQGGRRGDGEEETDAAQQIGDGLGGRGQFD 140 
6-2411    MEVKVTLIVAIVAALAISAHAQRDFNERRGKENDTERGQGGFGGRPGGMQMGGPRQDGGQMGGRRFDGPESGAPQMEGRRQNGGPMGGR--------------------------------------------------RFDGPRFGGSRPDGAGGRPFFGQGGRRGDGEEETDAAQQIGDGLGGRGQFD 140 
6-2412    MEVKVTLIVAIVAALAISAHAQRDFNERRGKENDTERGQGGFGGRPGGMQMGGPRQDGGPMGGRRFDGPESGAPQMEGRRQNGGPMGGR--------------------------------------------------RFDGPRFGGSRPDGAGGRPFFGQGGRRGDGEEETDAAQQIGDGLGGRGQFD 140 
6-2413    MEVKVTLIVAIVAALAISAHAQRDFNERRGKENDTERGQGGFGGRPGGMQMGGPRQDGGQMGGRRFDGPESGAPQMEGRRQNGGPMGGR--------------------------------------------------RFDGPRFGGSRPDGAGGRPFFGQGGRRGDGEEETDAAQQIGDGLGGRGQFD 140 
6-2414    MEVKVTLIVAIVAALAISAHAQRDFNERRGKENDTERGQGGFGGRPGGMQMGGPRQDGGPMGGRRFDGPESGAPQMEGRRQNGGPMGGR--------------------------------------------------RFDGPRFGGSRPDGAGGRPFFGQGGRRGDGEEETDAAQQVGDGLGGRGQFD 140 
6-2416    MEVKVTLIVAIVAALAISAHAQRDFNERRGKENDTERGQGGFGGRPGGMQMGGPRQDGGQMGGRRFDGPESGAPQMEGRRQNGGPMGGR--------------------------------------------------RFDGPRFGGSRPDGAGGRPFFGQGGRRGDGEEETDAAQQIGDGLGGRGQFD 140 
6-2417    MEVKVTLIVAIVAALAISAHAQRDFNERRGKENDTERGQGGFGGRPGGMQMGGPRQDGGPMGGRRFDGPESGAPQMEGRRQNGGPMGGR--------------------------------------------------RFDGPRFGGSRPDGAGGRPFFGQGGRRGDGEEETDAAQQIGDGLGGRGQFD 140 
6-2420    MEVKVTLIVAIVAALAISAHAQRDFNERRGKENDTERGQGGFGGRPGGMQMGGPRQDGGQMGGRRFDGPESGAPQMEGRRQNGGPMGGR--------------------------------------------------RFDGPRFGGSRPDGAGGRPFFGQGGRRGDGEEETDAAQQIGDGLGGRGQFD 140 
6-2421    MEVKVTLIVAIVAALAISAHAQRDFNERRGKENDTERGQGGFGGRPGGMQMGGPRQDGGQMGGRRFDGPESGAPQMEGRRQNGGPMGGR--------------------------------------------------RFDGPRFGGSRPDGAGGRPFFGQGGRRGDGEEETDAAQQIGDGLGGRGQFD 140 
6-2422    MEVKVTLIVAIVAALAISAHAQRDFNERRGKENDTERGQGGFGGRPGGMQMGGPRQDGGPMGGRRFDGPESGAPQMEGRRQNGGPMGGR--------------------------------------------------RFDGPRFGGSRPDGAGGRPFFGQGGRRGDGEEETDAAQQIGDGLGGRGQFD 140 
6-2424    MEVKVTLIVAIVAALAISAHAQRDFNERRGKENDTERGQGGFGGRPGGMQMGGPRQDGGQMGGRRFDGPESGAPQMEGRRQNGGPMGGR--------------------------------------------------RFDGPRFGGSRPDGAGGRPFFGQGGRRGDGEEETDAAQQIGDGLGGRGQFD 140 
6-2425    MEVKVTLIVAIVAALAISAHAQRDFNERRGKENDTERGQGGFGGRPGGMQMGGPRQDGGQMGGRRFDGPESGAPQMEGRRQNGGPMGGR--------------------------------------------------RFDGPRFGGSRPDGAGGRPFFGQGGRRGDGEEETDAAQQIGDGLGGRGQFD 140 
6-2427    MEVKVTLIVAIVAALAISAHAQRDFNERRGKENDTERGQGGFGGRPGGMQMGGPRQDGGQMGGRRFDGPESGAPQMEGRRQNGGPMGGR--------------------------------------------------RFDGPRFGGSRPDGAGGRPFFGQGGRRGDGEEETDAAQQIGDGLGGRGQFD 140 
6-2428    MEVKVTLIVAIVAALAISAHAQRDFNERRGKENDTERGQGGFGGRPGGMQMGGPRQDGGPMGGRRFDGPESGAPQMEGRRQNGGPMGGR--------------------------------------------------RFDGPRFGGSRPDGAGGRPFFGQGGRRGDGEEETDAAQQIGDGLGGRGQFD 140 
6-2430    MEVKVTLIVAIVAALAISAHAQRDFNERRGKENDTERGQGGFGGRPGGMQMGGPRQDGGPMGGRRFDGPESGAPQMEGRRQNGGPMGGR--------------------------------------------------RFDGPRFGGSRPDGAGGRPFFGQGGRRGDGEEETDAAQQIGDGLGGRGQFD 140 
6-2431    MEVKVTLIVAIVAALAISAHAQRDFNERRGKENDTERGQGGFGGRPGGMQMGGPRQDGGQMGGRRFDGPESGAPQMEGRRQNGGPMGGR--------------------------------------------------RFDGPRFGGSRPDGAGGRPFFGQGGRRGDGEEETDAAQQIGDGLGGRGQFD 140 
6-2432    MEVKVTLIVAIVAALAISAHAQRDFNERRGKENDTERGQGGFGGRPGGMQMGGPRQDGGQMGGRRFDGPESGAPQMEGRRQNGGPMGGR--------------------------------------------------RFDGPRFGGSRPDGAGGRPFFGQGGRRGDGEEETDAAQQIGDGLGGRGQFD 140 
6-2433    MEVKVTLIVAIVAALAISAHAQRDFNERRGKENDTERGQGGFGGRPGGMQMGGPRQDGGQMGGRRFDGPESGAPQMEGRRQNGGPMGGR--------------------------------------------------RFDGPRFGGSRPDGAGGRPFFGQGGRRGDGEEETDAAQQIGDGLGGRGQFD 140 
6-2434    MEVKVTLIVAIVAALAISAHAQRDFNERRGKENDTERGQGGFGGRPGGMQMGGPRQDGGQMGGRRFDGPESGAPQMEGRRQNGGPMGGR--------------------------------------------------RFDGPRFGGSRPDGAGGRPFFGQGGRRGDGEEETDAAQQIGDGLGGRGQFD 140 
6-2439    MEVKVTLIVAIVAALAISAHAQRDFNERRGKENDTERGQGGFGGRPGGMQMGGPRQDGGQMGGRRFDGPESGAPQMEGRRQNGGPMGGR--------------------------------------------------RFDGPRFGGSRPDGAGGRPFFGQGGRRGDGEEETDAAQQIGDGLGGRGQFD 140 
6-2440    MEVKVTLIVAIVAALAISAHAQRDFNERRGKENDTERGQGGFGGRPGGMQMGGPRQDGGPMGGRRFDGPESGAPQMEGRRQNGGPMGGR--------------------------------------------------RFDGPRFGGSRPDGAGGRPFFGQGGRRGDGEEETDAAQQIGDGLGGRGQFD 140 
6-2441    MEVKVTLIVAIVAALAISAHAQRDFNERRGKENDTERGQGGFGGRPGGMQMGGPRQDGGPMGGRRFDGPESGAPQMEGRRQNGGPMGGR--------------------------------------------------RFDGPRFGGSRPDGAGGRPFFGQGGRRGDGEEETDAAQQIGDGLGGRGQFD 140 
6-2435    MEVKVTLIVAIVAALAISAHAQRDFNERRGKENDTERGQGGFGGRPGGMQMGGPRQDGGQMGGRRFDGPESGAPQMEGRRQNGGPMGGR--------------------------------------------------RFDGPRFGGSRPDGAGGRPFFGQGGRRGDGEEETDAAQQIGDGLGGRGQFD 140 
6-2447    MEVKVTLIVAIVAALAISAHAQRDFNERRGKENDTERGQGGFGGRPGGMQMGGPRQDGGQMGGRRFDGPESGAPQMEGRRQNGGPMGGR--------------------------------------------------RFDGPRFGGSRPDGAGGRPFFGQGGRRGDGEEETDAAQQIGDGLGGRGQFD 140 
6-2448    MEVKVTLIVAIVAALAISAHAQRDFNERRGKENDTERGQGGFGGRPGGMQMGGPRQDGGPMGGRRFDGPESGAPQMEGRRQNGGPMGGR--------------------------------------------------RFDGPRFGGSRPDGAGGRPFFGQGGRRGDGEEETDAAQQIGDGLGGRGQFD 140 
6-2436    MEVKVTLIVAIVAALAISAHAQRDFNERRGKENDTERGQGGFGGRPGGMQMGGLRQDGGPMGGRRFDGPESGAPQMEGRRQNGGPMGGR--------------------------------------------------RFDGPRFGGSRPDGAGGRPFFGQGGRRGDGEEETDAAQQIGDGLGGRGQFD 140 
6-2450    MEVKVTLIVAIVAALAISAHAQRDFNERRGKENDTERGQGGFGGRPGGMQMGGPRQDGGQMGGRRFDGPESGAPQMEGRRQNGGPMGGR--------------------------------------------------RFDGPRFGGSRPDGAGGRPFFGQGGRRGDGEEETDAAQQIGDGLGGRGQFD 140 
6-2429    MEVKVTLIVAIVAALAISAHAQRDFNERRGKENDTERGQGGFGGRPGGMQMGGPRQDGGPMGGR--------------------------------------------------RFDGPGFGAPEMDGRRQNGGPMGGRRFDGPGFGGSRPDGAGGRPFFGQGGRRGDGEEETDAAQQIGDGPGGPGQFD 140 
6-2438    MEVKATLIVAIVAALAISAHARRDFNERRGNENGRERGQGRFGGRPGGMQMGGSRQDGGPMGGR--------------------------------------------------RFDGPGFGAPHMDGRRQNGGPMGGRRFDGPRFGGSRPDGAGGRPFFGQGGRRGDGEEETDAAQQIGDGLGGRGQFD 140 
6-2423    MEVKATLIVAIVAALAISAHARRDFNERRGNENGRERGQGRFGGRPGGMQMGGSRQDGGPMGGR--------------------------------------------------RFDGPGFGAPHMDGRRQNGGPMGGRRFDGPRFGGSRPDGAGGRPFFGQGGRRGDGEEETDAAQQIGDGLGGRGQFD 140 
6-2403    MEVKVTLIVAIVAALAISAHAQRDFNERRGKENDTERGQGGFGGRPGGMQMGGPRQDGGPMGGRRFDGPESGAPQMEGRRQNGGPMGGR--------------------------------------------------RFDGPRFGGSRPDGAGGRPFFGQGGRRGDGEEETDAAQQIGDGLGGRGQFD 140 
6-2444    MEVKVTLIVAIVAALAISAHAQRDFNERRGKENDTERGQGGFGGRPGGMQMGGPRQDGGPMGGGRFDGPESGAPQMEGRRQNGGPMGGR--------------------------------------------------RFDGPRFGGSRPDGAGGRPFFGQGGRRGDGEEETDAAQQIGDGLGGRGQFD 140 
6-2449    MEV*                                                                                                                                                                                           3   
2-1503    MEVKATLIVAIVAALAISAHAQRDFNERRGKENDTERGQGGFGGRPGGMQMGGPRQDGGPMGGGRFDGPESGAPQMEGRRQNGGPMGGR--------------------------------------------------RFDGPRFGGSRPDGAGGRPFFGQGGRRGDGEEETDAAQQIGDGLGGRGQFD 140 
2-1509    MEVKVTLIVAIVAALAISAHAQRDFNERRGKENDTERGQGGFGGRPGGMQMGGPRQDGGPMGGGRFDGPESGAPQMEGRRQNGGPMGGR--------------------------------------------------RFDGPRFGGSRPDGAGGRPFFGQGGRRGDGEEETDAAQQIGDGLGGRGQFD 140 
2-1513    MEVKVTLIVAIVAALAISAHAQRDFNERRGKENDTERGQGGFGGRPGGMQMGGPRQDGGPMGGGRFDGPESGAPQMEGRRQNGGPMGGR--------------------------------------------------RFDGPRFGGSRPDGAGGRPFFGQGGRRGDGEEETDAAQQIGDGLGGRGQFD 140 
2-1523    MEVKVTLIVAIVAALAISAHAQRDFNERRGKENDTERGQGGFGGRPGGMQMGGPRQDGGPMGGGRFDGPESGAPQMEGRRQNGGPMGGR--------------------------------------------------RFDGPRFGGSRPDGAGGRPFFGQGGRRGDGEEETDAAQQIGDGLGGRGQFD 140 
2-1524    MEVKVTLIVAIVAALAISAHAQRDFNERRGKENDTERGQGGFGGRPGGMQMGGPRQDGGPMGGGRFDGPESGAPQMEGRRQNGGPMGGR--------------------------------------------------RFDGPRFGGSRPDGAGGRPFFGQGGRRGDGEEETDAAQQIGDGLGGRGQFD 140 
2-1531    MEVKVTLIVAIVAALAISAHAQRDFNERRGKENDTERGQGGFGGRPGGMQMGGPRQDGGPMGGGRFDGPESGAPQMEGRRQNGGPMGGR--------------------------------------------------RFDGPRFGGSRPDGAGGRPFFGQGGRRGDGEEETDAAQQIGDGLGGRGQFD 140 
2-1533    MEVKVTLIVAIVAALAISAHAQRDFNERRGKENDTERGQGGFGGRPGGMQMGGPRQDGGPMGGGRFDGPESGAPQMEGRRQNGGPMGGR--------------------------------------------------RFDGPRFGGSRPDGAGGRPFFGQGGRRGDGEEETDAAQQIGDGLGGRGQFD 140 
2-1536    MEVKVTLIVAIVAALAISAHAQRDFNERRGKENDTERGQGGFGGRPGGMQMGGPRQDGGPMGGGRFDGPESGAPQMEGRRQNGGPMGGR--------------------------------------------------RFDGPRFGGSRPDGAGGRPFFGQGGRRGDGEEETDAAQQIGDGLGGRGQFD 140 
2-1502    MEVKVTLIVAIVAALAISAHAQRDFNERRGKENDTERGQGGFGGRPGGMQMGGPRQDGGPMGGRRFDGPESGAPQMEGRRQNGGPMGGR--------------------------------------------------RFDGPRFGGSRPDGAGGRPFFGQGGRRGDGEEETDAAQQIGDGLGGRGQFD 140 
2-1518    MEVKVTLIVAIVAALAISAHAQRDFNERRGKENDTERGQGGFGGRPGGMQMGGPRQDGGQMGGRRFDGPESGAPQMEGRRQNGGPMGGR--------------------------------------------------RFDGPRFGGSRPDGAGGRPFFGQGGRRGDGEEETDAAQQIGDGLGGRGQFD 140 
2-1519    MEVKVTLIVAIVAALAISAHAQRDFNERRGKENDTERGQGGFGGRPGGMQMGGPRQDGGQMGGRRFDGPEPGAPQMEGRRQNGGPMGGR--------------------------------------------------RFDGPRFGGSRPDGAGGRPFFGQGGRRGDGEEETDAAQQIGDGLGGRGQFD 140 
2-1511    MEVKVTLIVAIVAALAISAHAQRDFNERRGKENDTERGQGGFGGRPGGMQMGGPRQDGGQMGGRRFDGPESGAPQMEGRRQNGGPMGGR--------------------------------------------------RFDGPRFGGSRPDGAGGRPFFGQGGRRGDGEEETDAAQQIGDGLGGRGQFD 140 
2-1546    MEVKVTLIVAIVAALAISAHAQRDFNERRGKENDTERGQGGFGGRPGGMQMGGPRQDGGQMGGRRFDGPESGAPQMEGRRQNGGPMGGR--------------------------------------------------RFDGPRFGGSRPDGAGGRPFFGQGGRRGDGEEETDAAQQIGDGLGGRGQFD 140 
2-1548    MEVKVTLIVAIVAALAISAHAQRDFNERRGKENDTERGQGGFGGRPGGMQMGGPRQDGGQMGGRRFDGPESGAPQMEGRRQNGGPMGGR--------------------------------------------------RFDGPRFGGSRPDGAGGRPFFGQGGRRGDGEEETDAAQQIGDGLGGRGQFD 140 
2-1540    MEVKVTLIVAIVAALAISAHAQRDFNERRGKENDTERGQGGFGGRPGGMQMGGPRQDGGPMGGGRFDGPESGAPQMEGRRQNGGPMGGR--------------------------------------------------RFDGPRFGGSRPDGAGGRPSSAKEAGVVMEKKKLMLPNKLVMV         132 
2-2423    MEVKVTLIVAIVAALAISAHAQRDFNERRGKENDTERGQGGFGGRPGGMQMGGPRQDGGPMGGGRFDGPESGAPQMEGRRQNGGPMGGR--------------------------------------------------RFDGPRFGGSRPDGAGGRPFFGQGGRRGDGEEETDAAQQIGDGLGGRGQFD 140 
2-2436    MEVKVTLIVAIVAALAISAHAQRDFNERRGKENDTERGQGGFGGRPGGMQMGGPRQDGGPMGGGRFDGPKSGAPQMEGRRQNGGPMGGR--------------------------------------------------RFDGPRFGGSRPDGAGGRPFFGRGGRRGDGEEETDAAQQIGDGLGGRGQFD 140 
2-2405    MEVKVTLIVAIVAALAISAHAQRDFNERRGKENDTERGQGGFGGRPGGMQMGGPRQDGGPMGGR--------------------------------------------------RFDGPGFGAPEMDGRRQNGGPMGGRRFDGPGFGGSRPDGAGGRPFFGQGGRRGDGEEETDAAQQIGDGLGGRGQFD 140 
2-2403    MEVKVTLIVAIVAALAISAHAQRDFNERRGKENDTERGQGGFGGRPGGMQMGGPRQDGGQMGGRRFDGPESGAPQMEGRRQNGGPMGGR--------------------------------------------------RFDGPRFGGSRPDGAGGRPFFGQGGRRGDGEEETDAAQQIGDGLGGRGQFD 140 
2-2448    MEVKVTLIVAIVAALAISAHAQRDFNERRGKENDTERGQGGFGGRPGGMQMGGPRQDGGPMGGRRFDGPESGAPQMEGRRQNGGPMGGR--------------------------------------------------RFDGPRFGGSRPDGAGGRPFFGQGGRRGDGEEETDAAQQIGDGLGGRGQFD 140 
2-2404    MEVKVTLIVAIVAALAISAHAQRDFNERRGKENDTERGQGGFGGRPGGMQMGGPRQDGGPMGGRRFDGPESGAPQMEGRRQNGGPMGGR--------------------------------------------------RFDGPRFGGSRPDGAGGRPFFGQGGRRGDGEEETDAAQQIGDGLGGRGQFD 140 
2-2406    MEVKVTLIVAIVAALAISAHAQRDFNERRGKENDTERGQGGFGGRPGGMQMGGPRQDGGPMGGRRFDGPESGAPQMEGRRQNGGPMGGR--------------------------------------------------RFDGPRFGGSRPDGAGGRPFFGQGGRRGDGEEETDAAQQIGDGLGGRGQFD 140 
2-2409    MEVKVTLIVAIVAALAISAHAQRDFNERRGKENDTERGQGGFGGRPGGMQMGGPRQDGGPMGGRRFDGPESGAPQMEGRRQNGGPMGGR--------------------------------------------------RFDGPRFGGSRPDGAGGRPFFGQGGRRGDGEEETDAAQQIGDGLGGRGQFD 140 
2-2410    MEVKVTLIVAIVAALAISAHAQRDFNERRGKENDTERGQGGFGGRPGGMQMGGPRQDGGPMGGRRFDGPESGAPQMEGRRQNGGPMGGR--------------------------------------------------RFDGPRFGGSRPDGAGGRPFFGQGGRRGDGEEETDAAQQIGDGLGGRGQFD 140 
2-2411    MEVKVTLIVAIVAALAISAHAQRDFNERRGKENDTERGQGGFGGRPGGMQMGGPRQDGGPMGGRRFDGPESGAPQMEGRRQNGGPMGGR--------------------------------------------------RFDGPRFGGSRPDGAGGRPFFGQGGRRGDGEEETDAAQQIGDGLGGRGQFD 140 
2-2412    MEVKVTLIVAIVAALAISAHAQRDFNERRGKENDTERGQGGFGGRPGGMQMGGPRQDGGPMGGRRFDGPESGAPQMEGRRQNGGPMGGR--------------------------------------------------RFDGPRFGGSRPDGAGGRPFFGQGGRRGDGEEETDAAQQIGDGLGGRGQFD 140 
2-2413    MEVKVTLIVAIVAALAISAHAQRDSNERRGKENDTERGQGGFGGRPGGMQMGGPRQDGGPMGGRRFDGPESGAPQMEGRRQNGGPMGGR--------------------------------------------------RFDGPRFGGSRPDGAGGRPFFGQGGRRGDGEEETDAAQQIGDGLGGRGQFD 140 
2-2415    MEVKVTLIVAIVAALAISAHAQRDFNERRGKENDTERGQGGFGGRPGGMQMGGPRQDGGQMGGRRFDGPESGAPQMEGRRQNGGPMGGR--------------------------------------------------RFDGPRFGGSRPDGAGGRPFFGQGGRRGDGEEETDAAQQIGDGLGGRGQFD 140 
2-2416    MEVKVTLIVAIVAALAISAHAQRDFNERRGKENDTERGQGGFGGRPGGMQMGGPRQDGGQMGGRRFDGPESGAPQMEGRRQNGGPMGGR--------------------------------------------------RFDGPRFGGSRPDGAGGRPFFGQGGRRGDGEEETDAAQQIGDGLGGRGQFD 140 
2-2417    MEVKVTLIVAIVAALAISAHAQRDFNERRGKENDTERGQGGFGGRPGGMQMGGPRQDGGPMGGRRFDGPESGAPQMEGRRQNGGPMGGR--------------------------------------------------RFDGPRFGGSRPDGAGGRPFFGQGGRRGDGEEETDAAQQIGDGLGGRGQFD 140 
2-2418    MEVKVTLIVAIVAALAISAHAQRDFNERRGKENDTERGQGGFGGRPGGMQMGGPRQDGGPMGGRRFDGPESGAPQMEGRRQNGGPMGGR--------------------------------------------------RFDGPRFGGSRPDGAGGRPFFGQGGRRGDGEEETDAAQQIGDGLGGRGQFD 140 
2-2419    MEVKVTLIVAIVAALAISAHAQRDFNERRGKENDTERGQGGFGGRPGGMQMGGPRQDGGPMGGRRFDGPESGAPQMDGRRQNGGPMGGR--------------------------------------------------RFDGPRFGGSRPDGAGGRPFFGQGGRRGDGKEETDAAQQIGDGLGGRGQFD 140 
2-2420    MEVKVTLIVAIVAALAISAHAQRDFNERRGKENDTERGQGGFGGRPGGMQMGGPRQDGGPMGGRRFDGPESGAPQMEGRRQNGGPMGGR--------------------------------------------------RFDGPRFGGSRPDGAGGRPFFGQGGRRGDGEEETDAAQQIGDGLGGRGQFD 140 
2-2421    MEVKVTLIVAIVAALAISAHAQRDFNERRGKENDTERGQGGFGGRPGGMQMGGPRQDGGPMGGRRFDGPESGAPQMEGRRQNGGPMGGR--------------------------------------------------RFDGPRFGGSRPDGAGGRPFFGQGGRRGDGEEETDAAQQIGDGLGGRGQFD 140 
2-2422    MEVKVTLIVAIVAALAISAHAQRDFNERRGKENDTERGQGGFGGRPGGMQMGGPRQDGGPMGGRRFDGPESGAPQMEGRRQNGGPMGGR--------------------------------------------------RFDGPRFGGSRPDGAGGRPFFGQGGRRGDGEEETDAAQQIGDGLGGRGQFD 140 
2-2424    MEVKVTLIVAIVAALTISAHAQRDFNERRGKENDTERGQGGFGGRPGGMQMGGPRQDGGPMGGRRFDGPESGAPQMEGRRQNGGPMGGR--------------------------------------------------RFDGPRFGGSRPDGAGGRPFFGQGGRRGDGEEETDAAQQIGDGLGGRGQFD 140 
2-2425    MEVKVTLIVAIVAALAISAHAQRDFNERRGKENDTERGQGGFGGRPGGMQMGGPRQDGGPMGGRRFDGPESGAPQMEGRRQNGGPMGGR--------------------------------------------------RFDGPRFGGSRPDGAGGRPFFGQGGRRGDGEEETDAAQQIGDGLGGRGQFD 140 
2-2426    MEVKVTLIVAIVAALAISAHAQRDFNERRGKENDTERGQGGFGGRPGGMQMGGPRQDGGPMGGRRFDGPESGAPQMEGRRQNGGPMGGR--------------------------------------------------RFDGPRFGGSRPDGAGGRPFFGQGGRRGDGEEETDAAQQIGDGLGGRGQFD 140 
2-2427    MEVKVTLIVAIVAALAISAHAQRDFNERRGKENDTERGQGGFGGRPGGMQMGGPRQDGGPMGGRRFDGPESGAPQMEGRRQNGGPMGGR--------------------------------------------------RFDGPRFGGSRPDGAGGRPFFGQGGRRGDGEEETDAAQQIGDGLGGRDQFD 140 
2-2430    MEVKVTLIVAIVAALAISAHAQRDFNERRGKENDTERGQGGFGGRPGGMQMGGPRQDGGPMGGRRFDGPESGAPQMEGRRQNGGPMGGR--------------------------------------------------RFDGPRFGGSRPDGAGGRPFFGQGGRRGDGEEETDAAQQIGDGLGGRGQFD 140 
2-2431    MEVKVTLIVAIVAALAISAHAQRDFNERRGKENDTERGQGGFGGRPGGMQMGGPRQDGGPMGGRRFDGPESGAPQMEGRRQNGGPMGGR--------------------------------------------------RFDGPRFGGSRPDGAGGRPFFGQGGRRGDGEEETDAAQQIGDGLGGRGQFD 140 
2-2432    MEVKVTLIVAIVAALAISAHAQRDFNERRGKENDTERGQGGFGGRPGGMQMGGPRQDGGPMGGRRFDGPESGAPQMEGRRQNGGPMGGR--------------------------------------------------RFDGPRFGGSRPDGAGGRPFFGQGGRRGDGEEETDAAQQIGDGLGGRGQFD 140 
2-2434    MEVKVTLIVAIVAALAISAHAQRDFNERRGKENDTERGQGGFGGRPGGMQMGGPRQDGGPMGGRRFDGPESGAPQMEGRRQNGGPMGGR--------------------------------------------------RFDGPRFGGSRPDGAGGRPFFGQGGRRGDGEEETDAAQQIGDGLGGRGQFD 140 
2-2437    MEVKVTLIVAIVAALAISAHAQRDFNERRGKENDTERGQGGFGGRPGGMQMGGPRQDGGPMGGRRFDGPESGAPQMEGRRQNGGPMGGR--------------------------------------------------RFDGPRFGGSRPDGAGGRPFFGQGGRRGDGEEETDAAQQIGDGLGGRGQFD 140 
2-2438    MEVKVTLIVAIVAALAISAHAQRDFNERRGKENDTERGQGGFGGRPGGMQMGGPRQDGGQMGGRRFDGPESGAPQMEGRRQNGGPMGGR--------------------------------------------------RFDGPRFGGSRPDGAGGRPFFGQGGRRGDGEEETDAAQQIGDGLGGRGQFD 140 
2-2439    MEVKVTLIVAIVAALAISAHAQRDFNERRGKENDTERGQGGFGGRPGGVQMGGPRQDGGPMGGRRFDGPESGAPQMEGRRQNGGPMGGR--------------------------------------------------RFDGPRFGGSRPDGAGGRPFFGQGGRRGDGEEETDAAQQIGDGLGGRGQFD 140 
2-2440    MEVKVTLIVAIVAALAISAHAQRDFNERRGKENDTERGQGGFGGRPGGMQMGGPRQDGGPMGGRRFDGPESGAPQMEGRRQNGGPMGGR--------------------------------------------------RFDGPRFGGSRPDGAGGRPFFGQGGRRGDGEEETDAAQQIGDGLGGRGQFD 140 
2-2442    MEVKVTLIVAIVAALAISAHAQRDFNERRGKENDTERGQGGFGGRPGGMQMGGPRQDGGPMGGRRFDGPESGAPQMEGRRQNGGPMGGR--------------------------------------------------RFDGPRFGGSRPDGAGGRPFFGQGGRRGEGEEETDAAQQIGDGLGGRGQFD 140 
2-2445    MEVKVTLIVAIVAALAISAHAQRDFNERRGKENDTERGQGGFGGRPGGMQMGGPRQDGGQMGGRRFDGPESGAPQMEGRRQNGGPMGGR--------------------------------------------------RFDGPRFGGSRPDGAGGRPFFGQGGRRGDGEEETDAAQQIGDGLGGRGQFD 140 
2-2446    MEVKVTLIVAIVAALAISAHAQRDFNERRGKENDTERGQGGFGGRPGGMQMGGPRQDGGQMGGRRFDGPESGAPQMEGRRQNGGPMGGR--------------------------------------------------RFDGPRFGGSRPDGAGGRPFFGQGGRRGDGEEETDAAQQIGDGLGGRGQFD 140 
7-1501    MEVKVTLIVAIVAALAISAHAQRDFNERRGKENDTERGQGGFGGRPGGMQMGGPRQDGGPMGGGRFDGPESGAPQMEGRRQNGGPMGGR--------------------------------------------------RFDGPRFGGSRPDGAGGRPFFGQGGRRGDGEEETDAAQQIGDGLGGRGQFD 140 
7-1502    MEVKVTLIVAIVAALAISAHAQRDFNERRGKESDTERGQGGFGGRPGGMQMGGPRQDGGPMGGGRFDGPESGAPQMEGRRQNGGPMGGR--------------------------------------------------RFDGPRFGGSRPDGAGGRPFFGQGGRRGDGEEETDAAQQIGDGLGGRGQFD 140 
7-1503    MEVKVTLIVAIVAALAISAHAQRDFNERRGKENDTERGQGGFGGRPGGMQMGGPRQDGGPMGGGRFDGPESGAPQMEGRRQNGGPMGGR--------------------------------------------------RFDGPRFGGSRPDGAGGRPFFGQGGRRGDGEEETDAAQQIGDGLGGRGQFD 140 
7-1504    MEVKVTLIVAIVAALAISAHAQRDFNERRGKENDTERGQGGFGGRPGGMQMGGPRQDGGPMGGGRFDGPESGAPQMEGRRQNGGPMGGR--------------------------------------------------RFDGPRFGGSRPDGAGGRPFFGQGGRRGDGEEETDAAQQIGDGLGGRGQFD 140 
7-1505    MEVKVTLIVAIVAALAISAHAQRDFNERRGKENDTERGQGGFGGRPGGMQMGGPRQDGGPMGGGRFDGPESGAPQMEGRRQNGGPMGGR--------------------------------------------------RFDGPRFGGSRPDGAGGRPFFGQGGRRGDGEEETDAAQQIGDGLGGRGQFD 140 
7-1506    MEVKVTLIVAIVAALAISAHAQRDFNERRGKENDTERGQGGFGGRPGGMQMGGPRQDGGPMGGGRFDGPESGAPQMEGRRQNGGPMGGR--------------------------------------------------RFDGPRFGGSRPDGAGGRPFFGQGGRRGDGEEETDAAQQIGDGLGGRGQFD 140 
7-1508    MEVKVTLIVAIVAALAISAHAQRDFNERRGKENDTERGQGGFGGRPGGMQMGGPRQDGGPMGGGRFDGPESGAPQMEGRRQNGGPMGGR--------------------------------------------------RFDGPRFGGSRPDGAGGRPFFGQGGRRGDGEEETDAAQQIGDGLGGRGQFD 140 
7-1509    MEVKVTLIVAIVAALAISAHAQRDFNERRGKENDTERGQGGFGGRPGGMQMGGPRQDGGPMGGGRFDGPESGAPQMEGRRQNGGPMGGR--------------------------------------------------RFDGPRFGGSRPDGAGGRPFFGQGGRRGDGEEETDAAQQIGDGLGGRGQFD 140 
7-1510    MEVKVTLIVAIVAALAISAHAQRDFNERRGKENDTERGQGGFGGRPGGMQMGGPRQDGGPMGGGRFDGPESGAPQMEGRRQNGGPMGGR--------------------------------------------------RFDGPRFGGSRPDGAGGRPFFGQGGRRGDGEEETDAAQQIGDGLGGRGQFD 140 
7-1511    MEVKVTLIVAIVAALAISAHAQRDFNERRGKENDTERGQGGFGGRPGGMQMGGPRQDGGPMGGGRFDGPESGAPQMEGRRQNGGPMGGR--------------------------------------------------RFDGPRFGGSRPDGAGGRPFFGQGGRRGDGEEETDAAQQIGDGLGGRGQFD 140 
7-1512    MEVKVTLIVAIVAALAISAHAQRDFNERRGKENDTERGQGGFGGRPGGMQMGGPRQDGGPMGGGRFDGPESGAPQMEGRRQNGGPMGGR--------------------------------------------------RFDGPRFGGSRPDGAGGRPFFGQGGRRGDGEEETDAAQQIGDGLGGRGQFD 140 
7-1513    MEVKVTLIVAIVAALAISAHAQRDFNERRGKENDTERGQGGFGGRPGGMQMGGPRQDGGPMGGGRFDGPESGAPQMEGRRQNGGPMGGR--------------------------------------------------RFDGPRFGGSRPDGAGGRPFFGQGGRRGDGEEETDAAQQIGDGLGGRGQFD 140 
7-1515    MEVKVTLIVAIVAALAISAHAQRDFNERRGKENDTERGQGGFGGRPGGMQMGGPRQDGGPMGGGRFDGPESGAPQMEGRRQNGGPMGGR--------------------------------------------------RFDGPRFGGSRPDGAGGRPFFGQGGRRGDGEEETDAAQQIGDGLGGRGQFD 140 
7-1516    MEVKVTLIVAIVAALAISAHAQRDFNERRGMENDTERGQGGFGGRPGGMQMGGPRQDGGPMGGGRFDGPESGAPQMEGRRQNGGPMGGR--------------------------------------------------RFDGPRFGGSRPDGAGGRPFFGQGGRRGDGEEETDAAQQIGDGLGGRGQFD 140 
7-1517    MEVKVTLIVAIVAALAISAHAQRDFNERRGKENDTERGQGGFGGRPGGMQMGGPRQDGGPMGGGRFDGPESGAPQMEGRRQNGGPMGGR--------------------------------------------------RFDGPRFGGSRPDGAGGRPFFGQGGRRGDGEEETDAAQQIGDGLGGRGQFD 140 
7-1519    MEVKVTLIVAIVAALAISAHAQRDFNERRGKENDTERGQGGFGGRPGGMQMGGPRQDGGPMGGGRFDGPESGAPQMEGRRQNGGPMGGR--------------------------------------------------RFDGPRFGGSRPDGAGGRPFFGQGGRRGDGEEETDAAQQIGDGLGGRGQFD 140 
7-1520    MEVKVTLIVAIVAALAISAHAQRDFNERRGKENDTERGQGGFGGRPGGMQMGGPRQDGGPMGGGRFDGPESGAPQMEGRRQNGGPMGGR--------------------------------------------------RFDGPRFGGSRPDGAGGRPFFGQGGRRGDGEEETDAAQQIGDGLGGRGQFD 140 
7-1521    MEVKVTLIVAIVAALAISAHAQRDFNERRGKENDTERGQGGFGGRPGGMQMGGPRQDGGPMGGGRFDGPESGAPQMEGRRQNGGPMGGR--------------------------------------------------RFDGPRFGGSRPDGAGGRPFFGQGGRRGDGEEETDAAQQIGDGLGGRGQFD 140 
7-1523    MEVKVTLIVAIVAALAISAHAQRDFNERRGKENDTERGQGGFGGRPGGMQMGGPRQDGGPMGGGRFDGPESGAPQMEGRRQNGGPMGGR--------------------------------------------------RFDGPRFGGSRPDGAGGRPFFGQGGRRGDGEEETDAAQQIGDGLGGRGQFD 140 
7-1524    MEVKVTLIVAIVAALAISAHAQRDFNERRGKENDTERGQGGFGGRPGGMQMGGPRQDGGPMGGGRFDGPESGAPQMEGRRQNGGPMGGR--------------------------------------------------RFDGPRFGGSRPDGAGGRPFFGQGGRRGDGEEETDAAQQIGDGLGGRGQFD 140 
7-1525    MEVKVTLIVAIVAALAISAHAQRDFNERRGKENDTERGQGGFGGRPGGMQMGGPRQDGGPMGGGRFDGPESGAPQMEGRRQNGGPMGGR--------------------------------------------------RFDGPRFGGSRPDGAGGRPFFGQGGRRGDGEEETDAAQQIGDGLGGRGQFD 140 
7-1526    MEVKVTLIVAIVAALAISAHAQRDFNERRGKENDTERGQGGFGGRPGGMQMGGPRQDGGPMGGGRFDGPESGAPQMEGRRQNGGPMGGR--------------------------------------------------RFDGPRFGGSRPDGAGGRPFFGQGGRRGDGEEETDAAQQIGDGLGGRGQFD 140 
7-1527    MEVKVTLIVAIVAALAISAHAQRDFNERRGKENDTERGQGGFGGRPGGMQMGGPRQDGGPMGGGRFDGPESGAPQMEGRRQNGGPMGGR--------------------------------------------------RFDGPRFGGSRPDGAGGRPFFGQGGRRGDGEEETDAAQQIGDGLGGRGQFD 140 
7-1528    MEVKVTLIVAIVAALAISAHAQRDFNERRGKENDTERGQGGFGGRPGGMQMGGPRQDGGPMGGGRFDGPESGAPQMEGRRQNGGPMGGR--------------------------------------------------RFDGPRFGGSRPDGAGGRPFFGQGGRRGDGEEETDAAQQIGDGLGGRGQFD 140 
7-1529    MEVKVTLIVAIVAALAISAHAQRDFNERRGKENDTERGQGGFGGRPGGMQMGGPRQDGGPMGGGRFDGPESGAPQMEGRRQNGGPMGGR--------------------------------------------------RFDGPRFGGSRPDGAGGRPFFGQGGRRGDGEEETDAAQQIGDGLGGRGQFD 140 
7-1530    MEVKVTLIVAIVAALAISAHAQRDFNERRGKENDTERGQGGFGGRPGGMQMGGPRQDGGPMGGGRFDGPESGAPQMEGRRQNGGPMGGR--------------------------------------------------RFDGPRFGGSRPDGAGGRPFFGQGGRRGDGEEETDAAQQIGDGLGGRGQFD 140 
7-1533    MEVKVTLIVAIVAALAISAHAQRDFNERRGKENDTERGQGGFGGRPGGMQMGGPRQDGGPMGGGRSDGPESGAPQMEGRRQNGGPMGGR--------------------------------------------------RFDGPRFGGSRPDGAGGRPFFGQGGRRGDGEEETDAAQQIGDGLGGRGQFD 140 
7-1534    MEVKVTLIVAIVAALAISAHAQRDFNERRGKENDTERGQGGFGGRPGGMQMGGPRQDGGPMGGGRFDGPESGAPQMEGRRQNGGPMGGR--------------------------------------------------RFDGPRFGGSRPDGAGGRPFFGQGGRRGDGEEETDAAQQIGDGLGGRGQFD 140 
7-1536    MEVKVTLIVAIVAALAISAHAQRDFNERRGKENDTERGQGGFGGRPGGMQMGGPRQDGGPMGGGRFDGPESGAPQMEGRRQNGGPMGGR--------------------------------------------------RFDGPRFGGSRPDGAGGRPFFGQGGRRGDGEEETDAAQQIGDGLGGRGQFD 140 
7-1537    MEVKVTLIVAIVAALAISAHAQRDFNERRGKENDTERGQGGFGGRPGGMQMGGPRQDGGPMGGGRFDGPESGAPQMEGRRQNGGPMGGR--------------------------------------------------RFDGPRFGGSRPDGAGGRPFFGQGGRRGDGEEETDAAQQIGDGLGGRGQFD 140 
7-1538    MEVKVTLIVAIVAALAISAHAQRDFNERRGKENDTERGQGGFGGRPGGMQMGGPRQDGGPMGGGRFDGPESGAPQMEGRRQNGGPMGGR--------------------------------------------------RFDGPRFGGSRPDGAGGRPFFGQGGRRGDGEEETDAAQQIGDGLGGRGQFD 140 
7-1542    MEVKVTLIVAIVAALAISAHAQRDFNERRGKENDTERGQGGFGGRPGGMQMGGPRQDGGPMGGGRFDGPESGAPQMEGRRQNGGPMGGR--------------------------------------------------RFDGPRFGGSRPDGAGGRPFFGQGGRRGDGEEETDAAQQIGDGLGGRGQFD 140 
7-1544    MEVKVTLIVAIVAALAISAHAQRDFNERRGKENDTERGQGGFGGRPGGMQMGGPRQDGGPMGGGRFDGPESGAPQMEGRRQNGGPMGGR--------------------------------------------------RFDGPRFGGSRPDGAGGRPFFGQGGRRGDGEEETDAAQQIGDGLGGRGQFD 140 
7-1545    MEVKVTLIVAIVAALAISAHAQRDFNERRGKENDTERGQGGFGGRPGGMQMGGPRQDGGPMGGGRFDGPESGAPQMEGRRQNGGPMGGR--------------------------------------------------RFDGPRFGGSRPDGAGGRPFFGQGGRRGDGEEETDAAQQIGDGLGGRGQFD 140 
7-1546    MEVKVTLIVAIVAALAISAHAQRDFNERRGKENDTERGQGGFGGRPGGMQMGGPRQDGGPMGGGRFDGPESGAPQMEGRRQNGGPMGGR--------------------------------------------------RFDGPRFGGSRPDGAGGRPFFGQGGRRGDGEEETDAAQQIGDGLGGRGQFD 140 
7-1548    MEVKVTLIVAIVAALAISAHAQRDFNERRGKENDTERGQGGFGGRPGGMQMGGPRQDGGPMGGGRFDGPESGAPQMEGRRQNGGPMGGR--------------------------------------------------RFDGPRFGGSRPDGAGGRPFFGQGGRRGDGEEETDAAQQIGGGLGGRGQFD 140 
7-1549    MEVKVTLIVAIVAALAISAHAQRDFNERRGKENDTERGQGGFGGRPGGMQMGGPRQDGGPMGGGRFDGPESGAPQMEGRRQNGGPMGGR--------------------------------------------------RFDGPRFGGSRPDGAGGRPFFGRGGRRGDGEEETDAAQQIGDGLGGRGQFD 140 
7-1539    MEVKVTLIVAIVAALAISAHAQRDFNERRGKENDTERGQGGFGGRPGGMQMGGPRQDGGPMGGGRFDGPESGAPQMEGRRQNGGPMGGR--------------------------------------------------RFDGPRFGGSRPDGAGGRPFFGQGGRRGDGEEETDAAQQIGDGLGGRGQFD 140 
7-1550    MEVKVTLIVAIVAALAISAHAQRDFNERRGKENDTERGQGGFGGRPGGMQMGGPRQDGGPMGGGRFDGPESGAPQMEGRRQNGGPMGGR--------------------------------------------------RFDGPRFGGSRPDGAGGRPFFGQGGRRGDGEEETDAAQQIGDGLGGRGQFD 140 
7-1518    MEVKVTLIVAIVAALAISAHAQRDFNERRGKENDTERGQGGFGGRPGGMQMGGPRQDGGQMGGRRFDGPESGAPQMEGRRQNGGPMGGR--------------------------------------------------RFDGPRFGGSRPDGAGGRPFFGQGGRRGDGEEETDAAQQIGDGLGGRGQFD 140 
7-1540    MEVKVTLIVAIVAALAISAHAQRDFNERRGKENDTERGQGGFGGRPGGMQMGGPRQDGGPMGGGRFDGPESGAPQMEGR*                                                                                                               79  
7-1514    MEVKVTLIVAIVAALAISAHAQRDFNERRGKENDTERGQGGFGGRPGGMQMGGPRQDGGQMGGRRFDGPESGAPQMEGRRQNGGPMGGR--------------------------------------------------RFDGPRFGGSRPDGAGGRPFFGQGGRRGDGEEETDAAQQIGDGLGGRGQFD 140 
7-1507    MEVKVTLIVAIVAALAISAHAQRDFNERRGKENDTERGQGGFGGRPGGMQMGGLRQDGGPMGGRRFDGPESGAPQMEGRRQNGGPMGGR--------------------------------------------------RFDGPRFGGSRPDGAGGRPFFGQGGRRGDGEEETDAAQQIGDGLGGRGQFD 140 
7-1522    MEVKVTLIVAIVAALAISAHAQRDFNERRGKENDTERGQGGFGGRPGGMQMGGPRQDGGQMGGRRFDGPESGAPQMEGRRQNGGPMGGR--------------------------------------------------RFDGPRFGGSRPDGAGGRPFFGQGGRRGDGEEETDAAQQIGDGLGGRGQFD 140 
7-1532    MEVKVTLIVAIVAALAISAHAQRDFNERRGKENDTERGQGGFGGRPGGMQMGGPRQDGGQMGGRRFDGPESGAPQMEGRRQNGGPMGGR--------------------------------------------------RFDGPRFGGSRPDGAGGRPFFGQGGRRGDGEEETDAAQQIGDGLGGRGQFD 140 
7-1543    MEVKVTLIVAIVAALAISAHAQRDFNERRGKENDTERGQGGFGGRPGGMQMGGPRQDGGQMGGRRFDGPESGAPQMEGRRQNGGPMGGR--------------------------------------------------RFDGPRFGGSRPDGAGGRPFFGQGGRRGDGEEETDAAQQIGDGLGGRGQFD 140 
7-1547    MEVKVTLIVAIVAALAISAHAQRDFNERRGKENDTERGQGGFGGRPGGMQMGGLRQDGGPMGGRRFDGPESGAPQMEGRRQNGGPMGGR--------------------------------------------------RFDGPRFGGSRPDGAGGRPFFGQGGRRGDGEEETDAAQQIGDGLGGRGQFD 140 
7-2401    MEVKVTLIVAIVAALAISAHAQRDFNERRGKENDTERGQGGFGGRPGGMQMGGPRQDGGPMGGGRFDGPESGAPQMEGRRQNGGPMGGR--------------------------------------------------RFDGPRFGGSRPDGAGGRPFFGQGGRRGDGEEETDAAQQIGDGLGGRGQFD 140 
7-2402    MEVKVTLIVAIVAALAISAHAQRDFNERRGKENDTERGQGGFGGRPGGMQMGGPRQDGGPMGGGRFDGPESGAPQMEGRRQNGGPMGGR--------------------------------------------------RFDGPRFGGSRPDGAGGRPFFGQGGRRGDGEEETDAAQQIGDGLGGRGQFD 140 
7-2403    MEVKVTLIVAIVAALAISAHAQRDFNERRGKENDTERGQGGFGGRPGGMQMGGPRQDGGPMGGGRFDGPESGAPQMEGRRQNGGPMGGR--------------------------------------------------RFDGPRFGGSRPDGAGGRPFFGQGGRRGDGEEETDAAQQIGDGLGGRGQFD 140 
7-2404    MEVKVTLIVAIVAALAISAHAQRDFNERRGKENDTERGQGGFGGRPGGMQMGGPRQDGGPMGGGRFDGPESGAPQMEGRRQNGGPMGGR--------------------------------------------------RFDGPRFGGSRPDGAGGRPFFGQGGRRGDGEEETDAAQQIGDGLGGRGQFD 140 
7-2405    MEVKVTLIVAIVAALAISAHAQRDFNERRGKENDTERGQGGFGGRPGGMQMGGPRQDGGPMGGGRFDGPESGAPQMEGRRQNGGPMGGR--------------------------------------------------RFDGPRFGGSRPDGAGGRPFFGQGGRRGDGEEETDAAQQIGDGLGGRGQFD 140 
7-2406    MEVKVTLIVAIVAALAISAHAQRDFNERRGKENDTERGQGGFGGRPGGMQMGGPRQDGGPMGGGRFDGPESGAPQMEGRRQNGGPMGGR--------------------------------------------------RFDGPRFGGSRPDGAGGRPFFGQGGRRGDGEEETDAAQQIGDGLGGRGQFD 140 
7-2407    MEVKVTLIVAIVAALAISAHAQRDFNERRGKENDTERGQGGFGGRPGGMQMGGPRQDGGPMGGGRFDGPESGAPQMEGRRQNGGPMGGR--------------------------------------------------RFDGPRFGGSRPDGAGGRPFFGQGGRRGDGEEETDAAQQIGDGLGGRGQFD 140 
7-2408    MEVKVTLIVAIVAALAISAHAQRDFNERRGKENDTERGQGGFGGRPGGMQMGGPRQDGGPMGGGRFDGPESGAPQMEGRRQNGGPMGGR--------------------------------------------------RFDGPRFGGSRPDGAGGRPFFGQGGRRGDGEEETDAAQQIGDGLGGRGQFD 140 
7-2409    MEVKVTLIVAIVAALAISAHAQRDFNERRGKENDTERGQGGFGGRPGGMQMGGPRQDGGPMGGGRFDGPESGAPQMEGRRQNGGPMGGR--------------------------------------------------RFDGPRFGGSRPDGAGGRPFFGQGGRRGDGEEETDAAQQIGDGLGGRGQFD 140 
7-2410    MEVKVTLIVAIVAALAISAHAQRDFNERRGKENDTERGQGGFGGRPGGMQMGGPRQDGGPMGGGRFDGPESGAPQMEGRRQNGGPMGGR--------------------------------------------------RFDGPRFGGSRPDGAGGRPFFGQGGRRGDGEEETDAAQQIGDGLGGRGQFD 140 
7-2411    MEVKVTLIVAIVAALAISAHAQRDFNERRGKENDTERGQGGFGGRPGGMQMGGPRQDGGPMGGGRFDGPESGAPQMEGRRQNGGPMGGR--------------------------------------------------RFDGPRFGGSRPDGAGGRPFFGQGGRRGDGEEETDAAQQIGDGLGGRGQFD 140 
7-2412    MEVKVTLIVAIVAALAISAHAQRDFNERRGKENDTERGQGGFGGRPGGMQMGGPRQDGGPMGGGRFDGPESGAPQMEGRRQNGGPMGGR--------------------------------------------------RFDGPRFGGSRPDGAGGRPFFGQGGRRGDGEEETDAAQQIGDGLGGRGQFD 140 
7-2413    MEVKVTLIVAIVAALAISAHAQRDFNERRGKENDTERGQGGFGGRPGGMQMGGPRQDGGPMGGGRFDGPESGAPQMEGRRQNGGPMGGR--------------------------------------------------RFDGPRFGGSRPDGAGGRPFFGQGGRRGDGEEETDAAQQIGDGLGGRGQFD 140 
7-2414    MEVKVTLIVAIVAALAISAHAQRDFNERRGKENDTERGQGGFGGRPGGMQMGGPRQDGGPMGGGRFDGPESGAPQMEGRRQNGGPMGGR--------------------------------------------------RFDGPRFGGSRPDGAGGRPFFGQGGRRGDGEEETDAAQQIGDGLGGRGQFD 140 
7-2416    MEVKVTLIVAIVAALAISAHAQRDFNERRGKENDTERGQGGFGGRPGGMQMGGPRQDGGPMGGGRFDGPESGAPQMEGRRQNGGPMGGR--------------------------------------------------RFDGPRFGGSRPDGAGGRPFFGQGGRRGDGEEETDAAQQIGDGLGGRGQFD 140 
7-2417    MEVKVTLIVAIVAALAISAHAQRDFNERRGKENDTERGQGGFGGRPGGMQMGGPRQDGGPMGGGRFDGPESGAPQMEGRRQNGGPMGGR--------------------------------------------------RFDGPRFGGSRPDGAGGRPFFGQGGRRGDGEEETDAAQQIGDGLGGRGQFD 140 
7-2418    MEVKVTLIVAIVAALAISAHAQRDFNERRGKENDTERGQGGFGGRPGGMQMGGPRQDGGPMGGGRFDGPESGAPQMEGRRQNGGPMGGR--------------------------------------------------RFDGPRFGGSRPDGAGGRPFFGQGGRRGDGEEETDAAQQIGDGLGGRGQFD 140 
7-2420    MEVKVTLIVAIVAALAISAHAQRDFNERRGKENDTERGQGGFGGRPGGMQMGGPRQDGGPMGGGRFDGPESGAPQMEGRRQNGGPMGGR--------------------------------------------------RFDGPRFGGSRPDGAGGRPFFGQGGRRGDGEEETDAAQQIGDGLGGRGQFD 140 
7-2421    MEVKVTLIVAIVAALAISAHAQRDFNERRGKENDTERGQGGFGGRPGGMQMGGPRQDGGPMGGGRFDGPESGAPQMEGRRQNGGPMGGR--------------------------------------------------RFDGPRFGGSRPDGAGGRPFFGQGGRRGDGEEETDAAQQIGDGLGGRGQFD 140 
7-2423    MEVKVTLIVAIVAALAISAHAQRDFNERRGKENDTERGQGGFGGRPGGMQMGGPRQDGGPMGGGRFDGPESGAPQMEGRRQNGGPMGGR--------------------------------------------------RFDGPRFGGSRPDGAGGRPFFGQGGRRGDGEEETDAAQQIGDGLGGRGQFD 140 
7-2424    MEVKVTLIVAIVAALAISAHAQRDFNERRGKENDTERGQGGFGGRPGGMQMGGPRQDGGPMGGGRFDGPESGAPQMEGRRQNGGPMGGR--------------------------------------------------RFDGPRFGGSRPDGAGGRPFFGQGGRRGDGEEETDAAQQIGDGLGGRGQFD 140 
7-2425    MEVKVTLIVAIVAALAISAHAQRDFNERRGKENDTERGQGGFGGRPGGMQMGGPRQDGGPMGGGRFDGPESGAPQMEGRRQNGGPMGGR--------------------------------------------------RFDGPRFGSSRPDGAGGRPFFGQGGRRGDGEEETDAAQQIGDGLGGRGQFD 140 
7-2426    MEVKVTLIVAIVAALAISAHAQRDFNERRGKENDTERGQGGFGGRPGGMQMGGPRQDGGPMGGGRFDGPESGAPQMEGRRQNGGPMGGR--------------------------------------------------RFDGPRFGGSRPDGAGGRPFFGQGGRRGDGEEETDAAQQIGDGLGGRGQFD 140 
7-2430    MEVIVTLIVAIVAALAISAHAQRDFNERRGKENDTERGQGGFGGRPGGMQMGGPRQDGGPMGGGRFDGPESGAPQMEGRRQNGGPMGGR--------------------------------------------------RFDGPRFGGSRPDGAGGRPFFGQGGRRGDGEEETDAAQQIGDGLGGRGQFD 140 
7-2431    MEVKVTLIVAIVAALAISAHAQRDFNERRGKENDTERGQGGFGGRPGGMQMGGPRQDGGPMGGGRFDGPESGAPQMEGRRQNGGPMGGR--------------------------------------------------RFDGPRFGGSRPDGAGGRPFFGQGGRRGDGEEETDAAQQIGDGLGGRGQFD 140 
7-2432    MEVKVTLIVAIVAALAISAHAQRDFNERRGKENDTERGQGGFGGRPGGMQMGGPRQDGGPMGGGRFDGPESGAPQMEGRRQNGGPMGGR--------------------------------------------------RFDGPRFGGSRPDGAGGRPFFGQGGRRGDGEEETDAAQQIGDGLGGRGQFD 140 
7-2436    MEVKVTLIVAIVAALAISAHAQRDFNERRGKENDTERGQGGFGGRPGGMQMGGPRQDGGPMGGGRFDGPKSGAPQMEGRRQNGGPMGGR--------------------------------------------------RFDGPRFGGSRPDGAGGRPFFGRGGRRGDGEEETDAAQQIGDGLGGRGQFD 140 
7-2437    MEVKVTLIVAIVAALAISAHAQRDFNERRGKENDTERGQGGFGGRPGGMQMGGPRQDGGPMGGGRFDGPESGAPQMEGRRQNGGPMGGR--------------------------------------------------RFDGPRFGGSRPDGAGGRPFFGQGGRRGDGEEETDAAQQIGDGLGGRGQFD 140 
7-2439    MEVKVTLIVAIVAALAISAHAQRDFNERRGKENDTERGQGGFGGRPGGMQMGGPRQDGGPMGGGRFDGPESGAPQMEGRRQNGGPMGGW--------------------------------------------------RFDGPRFGGSRPDGAGGRPFFGQGGRRGDGEEETDAAQQIGDGLGGRGQFD 140 
7-2442    MEVKVTLIVAIVAALAISAHAQRDFNERRGKENDTERGQGGFGGRPGGMQMGGPRQDGGPMGGGRFDGPESGAPQMEGRRQNGGPMGGR--------------------------------------------------RFDGPRFGGSRPDGAGGRPFFGQGGRRGDGEEETDAAQQIGDGLGGRGQFD 140 
7-2443    MEVKVTLIVAIVAALAISAHAQRDFNERRGKENDTERGQGGFGGRPGGMQMGGPRQDGGPMGGGRFDGPESGAPQMEGRRQNGGPMGGR--------------------------------------------------RFDGPRFGGSRPDGAGGRPFFGRGGRRGDGEEETDAAQQIGDGLGGRGQFD 140 
7-2444    MEVKVTLIVAIVAALAISAHAQRDFNERRGKENDTERGQGGFGGRPGGMQMGGPRQDGGPMGGGRFDGPESGAPQMEGRRQNGGPMGGR--------------------------------------------------RFDGPRFGGSRPDGAGGRPFFGQGGRRGDGEEETDAAQQIGDGLGGCGQFD 140 
7-2445    MEVKVTLIVAIVAALAISAHAQRDFNERRGKENDTERGQGGFGGRPGGMQMGGPRQDGGPMGGGRFDGPESGAPQMEGRRQNGGPMGGR--------------------------------------------------RFDGPRFGGSRPDGAGGRPFFGQGGRRGDGEEETDAAQQIGDGLGGRGQFD 140 
7-2446    MEVKVTLIVAIVAALAISAHAQRDFNERRGKENDTERGQGGFGGRPGGMQMGGPRQDGGPMGGGRFDGPESGAPQMEGRRQNGGPMGGR--------------------------------------------------RFDGPRFGGSRPDGAGGRPFFGQGGRRGDGEEETDAAQQIGDGLGGRGQFD 140 
7-2447    MEVKVTLIVAIVAALAISAHAQRDFNERRGKENDTERGQGGFGGRPGGMQMGGPRQDGGPMGGGRFDGPESGAPQMEGRRQNGGPMGGR--------------------------------------------------RFDGPRFGGSRPDGAGGRPFFGQGGRRGDGEEETDAAQQIGDGLGGRGQFD 140 
7-2448    MEVKVTLIVAIVAALAISAHAQRDFNERRGKENDTERGQGGFGGRPGGMQMGGPRQDGGPMGGGRFDGPESGAPQMEGRRQNGGPMGGR--------------------------------------------------RFDGPRFGGSRPDGAGGRPFFGQGGRRGDGEEETDAAQQIGDGLGGRGQFD 140 
7-2450    MEVKVTLIVAIVAALAISAHAQRDFNERRGKENDTERGQGGFGGRPGGMQMGGPRQDGGPMGGGRFDGPESGAPQMEGRRQNGGPMGGR--------------------------------------------------RFDGPRFGGSRPDGAGGRPFFGQGGRRGDGEEETDAAQQIGDGLGGRGQFD 140 
7-2415    MEVKVTLIVAIVAALAISAHAQRDFNERRGKENDTERGQGGFGGRPGGMQMGGLRQDGGPMGGRRFDGPESGAPQMEGRRQNGGPMGGR--------------------------------------------------RFDGPRFGGSRPDGAGGRPFFGQGGRRGDGEEETDAAQQIGDGLGGRGQFD 140 
7-2427    MEVKVTLIVAIVAALAISAHAQRDFNERRGKENDTERGQGGFGGRPGGMQMGGPRQDGGPMGGRRFDGPESGAPQMEGRRQNGGPMGGR--------------------------------------------------RFDGPRFGGSRPDGAGGRPFFGQGGRRGDGEEETDAAQQIGDGLGGRGQFD 140 
7-2428    MEVKVTLIVAIVAALAISAHAQRDFNERRGKENDTERGQGGFGGRPGGMQMGGPRQDGGPMGGRRFDGPESGAPQMEGRRQNGGPMGGR--------------------------------------------------RFDGPRFGGSRPDGAGGRPFFGQGGRRGDGEEETDAAQQIGDGLGGRGQFD 140 
7-2435    MEVKVTLIVAIVAALAISAHAQRDFNERRGKENDTERGQGGFGGRPGGMQMGGPRQDGGQMGGRRFDGPESGAPQMEGRRQNGGPMGGR--------------------------------------------------RFDGPRFGGSRPDGAGGRPFFGQGGRRGDGEEETDAAQQIGDGLGGRGQFD 140 
7-2440    MEVKVTLIVAIVAALAISAHAQRDFNERRGKENDTERGQGGFGGRPGGMQMGGPRQDGGPMGGRRFDGPESGAPQMEGRRQNGGPMGGR--------------------------------------------------RFDGPQFGGSRPDGAGGRPFFGQGGRRGDGEEETDAAQQIGDGLGGRGQFD 140 
7-2441    MEVKVTLIVAIVAALAISAHAQRDFNERRGKENDTERGQGGFGGRPGGMQMGGPRQDGGQMGGRRFDGPESGAPQMEGRRQNGGPMGGR--------------------------------------------------RFDGPRFGGSRPDGAGGRPFFGQGGRRGDGEEETDAAQQIGDGLGGRGQFD 140 
7-2449    MEVKVTLIVAIVAALAISAHAQRDFNERRGKENDTERGQGGFGGRPGGMQMGGPRQDGGQMGGRRFDGPESGAPQMEGRRQNGGPMGGR--------------------------------------------------RFDGPRFGGSRPDGAGGRPFFGQGGRRGDGEEETDAAQQIGDGLGGRGQFD 140 
8-1501    MEVKVTLIVAIVAALAISAHAQRDFNERRGKENDTERGQGGFGGRPGGMQMGGPRQDGGPMGGGRFDGPESGAPQMEGRRQNGGPMGGR--------------------------------------------------RFDGPRFGGSRPDGAGGRPFFGQGGRRGDGEEETDAAQQIGDGLGGRGQFD 140 
8-1502    MEVKVTLIVAIVAALAISAHAQRDFNERRGKENDTERGQGGFGGRPGGMQMGGPRQDGGPMGGGRFDGPESGAPQMEGRRQNGGPMGGR--------------------------------------------------RFDGPRFGGSRPDGAGGRPFFGQGGRRGDGEEETDAAQQIGDGLGGRGQFD 140 
8-1503    MEVKVTLIVAIVAALAISAHAQRDFNERRGKENDTERGQGGFGGRPGGMQMGGPRQDGGPMGGGRFDGPESGAPQMEGRRQNGGPMGGR--------------------------------------------------RFDGPRFGGSRPDGAGGRPFFGQGGRRGDGEEETDAAQQIGDGLGGRGQFD 140 
8-1504    MEVKVTLIVAIVAALAISAHAQRDFNERRGKENDTERGQGGFGGRPGGMQMGGPRQDGGPMGGGRFDGPESGAPQMEGRRQNGGPMGGR--------------------------------------------------RFDGPRFGGSRPDGAGGRPFFGQGGRRGDGEEETDAAQQIGDGLGGRGPFD 140 
8-1505    MEVKVTLIVAIVAALAISAHAQRDFNERRGKENDTERGQGGFGGRPGGMQMGGPRQDGGPMGGGRFDGPESGAPQMEGRRQNGGPMGGR--------------------------------------------------RFDGPRFGGSRPDGAGGRPFFGQGGRRGDGEEETDAAQQIGDGLGGRGQFD 140 
8-1506    MEVKVTLIVAIVAALAISAHAQRDFNERRGKENDTERGQGGFGGRPGGMQMGGPRQDGGPMGGGRFDGPESGAPQMEGRRQNGGPMGGR--------------------------------------------------RFDGPRFGGSRPDGAGGRPFFGQGGRRGDGEEETDAAQQIGDGLGGRGQFD 140 
8-1507    MEVKVTLIVAIVAALAISAHAQRDFNERRGKENDTERGQGGFGGRPGGVQMGGPRQDGGPMGGGRFDGPESGAPQMEGRRQNGGPMGGR--------------------------------------------------RFDGPRFGGSRPDGAGGRPFFGQGGRRGDGEEETDAAQQIGDGLGGRGQFD 140 
8-1508    MEVKVTLIVAIVAALAISAHAQRDFNERRGKENDTERGQGGFGGRPGGMQTGGPRQDGGPMGGGRFDGPESGAPQMEGRRQNGGPMGGR--------------------------------------------------RFDGPRFGGSRPDGAGGRPFFGQGGRRGDGEEETDAAQQIGDGLGGRGQFD 140 
8-1509    MEVKVTLIVAIVAALAISAHAQRDFNERRGKENDTERGQGGFGGRPGGMQMGGPRQDGGPMGGGRFDGPESGAPQMEGRRQNGGPMGGR--------------------------------------------------RFDGPRFGGSRPDGAGGRPFFGQGGRRGDGEEETDAAQQIGDGLGGRGQFD 140 
8-1510    MEVKVTLIVAIVAALAISAHAQRDFNERRGKENDTERGQGGFGGRPGGMQMGGPRQDGGPMGGGRFDGPESGAPQMEGRRQNGGPMGGR--------------------------------------------------RFDGPRFGGSRPDGAGGRPFFGQGGRRGDGEEETDAAQQIGDGLGGRGQFD 140 
8-1511    MEVKVTLIVAIVAALAISAHAQRDFNERRGKENDTERGQGGFGGRPGGMQMGGPRQDGGPMGGGRFDGPESGAPQMEGRRQNGGPMGGR--------------------------------------------------RFDGPRFGGSRPDGAGGRPFFGQGGRRGDGEEETDAAQQIGDGLGGRGQFD 140 
8-1512    MEVKVTLIVAIVAALAISAHAQRDFNERRGKENDTERGQGGFGGRPGGMQMGGPRQDGGPMGGGRFDGPESGAPQMEGRRQNGGPMGGR--------------------------------------------------RFDGPRFGGSRPDGAGGRPFFGQGGRRGDGEEETDAAQQIGDGLGGRGQFD 140 
8-1513    MEVKVTLIVAIVAALAISAHAQRDFNERRGKENDTERGQGGFGGRPGGMQMGGPRQDGGPMGGGRFDGPESGAPQMEGRRQNGGPMGGR--------------------------------------------------RFDGPRFGGSRPDGAGGRPFFGQGGRRGDGEEETDAAQQIGDGLGGRGQFD 140 
8-1514    MEVKVTLIVAIVAALAISAHAQRDFNERRGKENDTERGQGGFGGRPGGMQMGGPRQDGGPMGGGRFDGPESGAPQMEGRRQNGGPMGGR--------------------------------------------------RFDGPRFGGSRPDGAGGRPFFGQGGRRGDGEEETDAAQQIGDGLRGRGQFD 140 
8-1515    MEVKVTLIVAIVAALAISAHAQRDFNERRGKENDTERGQGGFGGRPGGMQMGGPRQDGGPMGGGRFDGPESGAPQMEGRRQNGGPMGGR--------------------------------------------------RFDGPRFGGSRPDGAGGRPFFGQGGRRGDGEEETDAAQQIGDGLGGRGQFD 140 
8-1516    MEVKVTLIVAIVAALAISAHAQRDFNERREKENDTERGQGGFGGRPGGMQMGGPRQDGGPMGGGRFDGPESGAPQMEGRRQNGGPMGGR--------------------------------------------------RFDGPRFGGSRPDGAGGRPFFGQGGRRGDGEEETDAAQQIGDGLGGRGQFD 140 
8-1517    MEVKVTLIVAIVAALAISAHAQRDFNERRGKENDTERGQGGFGGRPGGMQMGGPRQDGGPMGGGRFDGPESGAPQMEGRRQNGGPMGGR--------------------------------------------------RFDGPRFGGSRPDGAGGRPFFGQGGRRGDGEEETDAAQQIGDGLGGRGQFD 140 
8-1518    MEVKVTLIVAIVAALAISAHAQRDFNERRGKENDTERGQGGFGGRPGGMQMGGPRQDGGPMGGGRFDGPESGAPQMEGRRQNGGPMGGR--------------------------------------------------RFDGPRFGGSRPDGAGGRPFFGQGGRRGDGEEGTDAAQQIGDGLGGRGQFD 140 
8-1519    MEVKVTLIVAIVAALAISAHAQRDFNERRGKENDTERGQGGFGGRPGGMQMGGPRQDGGPMGGGRFDGPESGAPQMEGRRQNGGPMGGR--------------------------------------------------RFDGPRFGGSRPDGAGGRPFFGQGGRRGDGEEETDAAQQIGDGLGGRGQFD 140 
8-1520    MEVKVTLIVAIVAALAISAHAQRDFNERRGKENDTERGQGGFGGRPGGMQMGGPRQDGGPMGGGRFDGPESGAPQMEGRRQNGGPMGGR--------------------------------------------------RFDGPRFGGSRPDGAGGRPFFGQGGRRGDGEEETDAAQQIGDGLGGRGQFD 140 
8-1521    MEVKVTLIVAIVAALAISAHAQRDFNERRGKENDTERGQGGFGGRPGGMQMGGPRQDGGPMGGGRFDGPESGAPQMEGRRQNGGPMGGR--------------------------------------------------RFDGPRFGGSRPDGAGGRPFFGQGGRRGDGEEETDAAQQIGDGLGGRGQFD 140 
8-1522    MEVKVTLIVAIVAALAISAHAQRDFNERRGKENDTERGQGGFGGRPGGMQMGGPRQDGGPMGGGRFDGPESGAPQMEGRRQNGGPMGGR--------------------------------------------------RFDGPRFGGSRPDGAGGRPFFGQGGRRGDGEEETDAAQQIGDGLGGRGQFD 140 
8-1524    MEVKVTLIVAIVAALAISAHAQRDFNERRGKENDTERGQGGFGGRPGGMQMGGPRQDGGPMGGGRFDGPESGAPQMEGRRQNGGPMGGR--------------------------------------------------RFDGPRFGGSRPDGAGGRPFFGQGGRRGDGEEETDAAQQIGDGLGGRGQFD 140 
8-1525    MEVKVTLIVAIVAALAISAHAQRDFNERRGKENDTERGQGGFGGRPGGMQMGGPRQDGGPMGGGRFDGPESGAPQMEGRRQNGGPMGGR--------------------------------------------------RFDGPRFGGSRPDGAGGRPFFGQGGRRGDGEEETDAAQQIGDGLGGRGQFD 140 
8-1526    MEVKVTLIVAIVAALAISAHAQRDFNERRGKENDTERGQGGFGGRPGGMQMGGPRQDGGPMGGGRFDGPESGAPQMEGRRRNGGPMGGR--------------------------------------------------RFDGPRFGGSRPDGAGGRPFFGQGGRRGDGEEETDAAQQIGDGLGGRGQFD 140 
8-1527    MEVKVTLIVAIVAALAISAHAQRDFNERRGKENDTERGQGGFGGRPGGMQMGGPRQDGGPMGGGRFDGPESGAPQMEGRRQNGGPMGGR--------------------------------------------------RFDGPRFGGSRPDGAGGRPFFGQGGRRGDGEEETDAAQQIGDGLGGRGQFD 140 
8-1528    MEVKVTLIVAIVAALAISAHAQRDFNERRGKENDTERGQGGFGGRPGGMQMGGPRQDGGPMGGGRFDGPESGAPQMEGRRQNGGPMGGR--------------------------------------------------RFDGPRFGGSRPDGAGGRPFFGQGGRRGDGEEETDAAQQIGDGLGGRGQFD 140 
8-1529    MEVKVTLIVAIVAALAISAHAQRDFNERRGKENDTERGQGGFGGRPGGMQMGGPRQDGGPMGGGRFDGPESGAPQMEGRRQNGGPMGGR--------------------------------------------------RFDGPRFGGSRPDGAGGRPFFGQGGRRGDGEEETDAAQQIGDGLGGRGQFD 140 
8-1530    MEVKVTLIVAIVAALAISAHAQRDFNERRGKENDTERGQGGFGGRPGGMQMGGPRQDGGPMGGGRFDGPESGAPQMEGRRQNGGPMGGR--------------------------------------------------RFDGPRFGGSRPDGAGGRPFFGQGGRRGDGEEETDAAQQIGDGLGGRGQFD 140 
8-1531    MEVKVTLIVAIVAALAISAHAQRGFNERRGKENDTERGQGGFGGRPGGMQMGGPRQDGGPMGGGRFDGPESGAPQMEGRRQNGGPMGGR--------------------------------------------------RFDGPRFGGSRPDGAGGRPFFGQGGRRGDGEEETDAAQQIGDGLGGRGQFD 140 
8-1532    MEVKVTLIVAIVAALAISAHAQRDFNERRGKENDTERGQGGFGGRPGGMQMGGPRQDGGPMGGGRFDGPESGAPQMEGRRQNGGPMGGR--------------------------------------------------RFDGPRFGGSRPDGAGGRPFFGQGGRRGDGEEETDAAQQIGDGLGGRGQFD 140 
8-1533    MEVKVTLIVAIVAALAISAHAQRDFNERRGKENDTERGRGGFGGRPGGMQMGGPRQDGGPMGGGRFDGPESGAPQMEGRRQNGGPMGGR--------------------------------------------------RFDGPRFGGSRPDGAGGRPFFGQGGRRGDGEEETDAAQQIGDGLGGRGQFD 140 
8-1534    MEVKVTLIVAIVAALAISAHAQRDFNERRGKENDTERGQGGFGGRPGGMQMGGPRQDGGPMGGGRFDGPESGAPQMEGRRQNGGPMGGR--------------------------------------------------RFDGPRFGGSRPDGAGGRPFFGQGGRRGDGEEETDAAQQIGDGLGGRGQFD 140 
8-1535    MEVKVTLIVAIVAALAISAHAQRDFNERRGKENDTERGQGGFGGRPGGMQMGGPRQDGGPMGGGRFDGPESGAPQMEGRRQNGGPMGGR--------------------------------------------------RFDGPRFGGSRPDGAGGRPFFGQGGRRGDGEEETDAAQQIGDGLGGRGQFD 140 
8-1536    MEVKVTLIVAIVAALAISAHAQRDFNERRGKENDTERGQGGFGGRPGGMQMGGPRQDGGPMGGGRFDGPESGAPQMEGRRQNGGPMGGR--------------------------------------------------RFDGPRFGGSRPDGAGGRPFFGQGGRRGDGEEETDAAQQIGDSLGGRGQFD 140 
8-1537    MEVKVTLIVAIVAALAISAHAQRDFNERRGKENDTERGQGGFGGRPGGMQMGGPRQDGGPMGGGRFDGPESGAPQMEGRRQNGGPMGGR--------------------------------------------------RFDGPRFGGSRPDGAGGRPFFGQGGRRGDGEEETDAAQQIGDGLGGRGQFD 140 
8-1538    MEVKVTLIVAIVAALAISAHAQRDFNERRGKENDTERGQGGFGGRPGGMQMGGPRQDGGPMGGGRFDGPESGAPQMEGRRQNGGPMGGR--------------------------------------------------RFDGPRFGGSRPDGAGGRPFFGQGGRRGDGEEETDAAQQIGDGLGGRGQFD 140 
8-1539    MEVKVTLIVAIVAALAISAHAQRDFNERRGKENDTERGQGGFGGRPGGMQMGGPRQDGGPMGGGRFDGPESGAPQMEGRRQNGGPMGGR--------------------------------------------------RFDGPRFGGSGPDGAGGRPFFGQGGRRGDGEEETDAAQQIGDGLGGRGQFD 140 
8-1540    MEVKVTLIVAIVAALAISAHAQRDFNERRGKENDTERGQGGFGGRPGGMQMGGPRQDGGPMGGGRFDGPESGAPQMEGRRQNGGPMGGR--------------------------------------------------RFDGPRFGGSRPDGAGGRPFFGQGGRRGDGEEETDAAQQIGDGLGGRGQFD 140 
8-1542    MEVKVTLIVAIVAALAISAHAQRDFNERRGKENDTERGQGGFGGRPGGMQMGGPRQDGGPMGGGRFDGPESGAPQMEGRRQNGGPMGGR--------------------------------------------------RFDGPRFGGSRPDGAGGRPFFGQGGRRGDGEEETDAVQQIGDGLGGRGQFD 140 
8-1546    MEVKVTLIVAIVAALAISAHAQRDFNERRGKENDTERGQGGFGGRPGGMQMGGPRQDGGPMGGGRFDGPESGAPQMEGRRQNGGPMGGR--------------------------------------------------RFDGPRFGGSRPDGAGGRPFFGQGGRRGDGEEETDAAQQIGDGLGGRGQFD 140 
8-1548    MEVKVTLIVAIVAALAISAHAQRDFNERRGKENDTERGQGGFGGRPGGMQMGGPRQDGGPMGGGRFDGPESGAPQMEGRRQNGGPMGGR--------------------------------------------------RFDGPRFGGSRPDGAGGRPFFGQGGRRGDGEEETDAAQQIGDGLGGRGQFD 140 
8-1549    MEVKVTLIVAIVAALAISAHAQRDFNERRGKENDTERGQGGFGGRPGGMQMGGPRQDGGPMGGGRFDGPESGAPQMEGRRQNGGPMGGR--------------------------------------------------RFDGPRFGGSRPDGAGGRPFFGQGGRRGDGEEETDAAQQIGDGLGGRGQFD 140 
8-1550    MEVKVTLIVAIVAALAISAHAQRDFNERRGKENDTERGQGGFGGRPGGMQMGGPRQDGGPMGGGRFDGPESGAPQMEGRRQNGGPMGGR--------------------------------------------------RFDGPRFGGSRPDGAGGRPFFGQGGRRGDGEEETDAAQQIGDGLGGRGQFD 140 
8-1541    MEVKVTLIVAIVAALAISAHAQRDFNERRGKENDTERGQGGFGGRPGGMQMGGPRQDGGPMGGRRFDGPESGAPQMEGRRQNGGPMGGR--------------------------------------------------RFDGPRFGGSRPDGAGGRPFFGQGGRRGDGEEETDAAQQIGDGLGGRGQFD 140 
8-1543    MEVKVTLIVAIVAALAISAHAQRDFNERRGKENDTERGQGGFGGRPGGMQMGGPRQDGGPMGGRRFDGPESGAPQMEGRRQNGGPMGGR--------------------------------------------------RFDGPRFGGSRPDGAGGRPFFGQGGRRGDGEEETDAAQQIGDGLGGRGQFD 140 
8-2401    MEVKVTLIVAIVAALAISAHAQRDFNERRGKENDTERGQGGFGGRPGGMQMGGPRQDGGPMGGGRFDGPESGAPQMEGRRQNGGPMGGR--------------------------------------------------RFDGPRFGGSRPDGAGGRPFFGQGGRRGDGEEETDAAQQIGDGLGGRGQFD 140 
8-2450    MEVKVTLIVAIVAALAISAHAQRDFNERRGKENDTERGQGGFGGRPGGMQMGGPRQDGGPMGGGRFDGPESGAPQMEGRRQNGGPMGGR--------------------------------------------------RFDGPRFGGSRPDGAGGRPFFGQGGRRGDGEEETDAAQQIGDGLGGRGQFD 140 
8-2406    MEVKVTLIVAIVAALAISAHAQRDFNERRGKENDTERGQGGFGGRPGGMQMGGPRQDGGPMGGGRFDGPESGAPQMEGRRQNGGPMGGR--------------------------------------------------RFDGPRFGGSRPDGAGGRPFFGQGGRRGDGEEETDAAQQIGDGLGGRGQFD 140 
8-2407    MEVKVTLIVAIVAALAISAHAQRDFNERRGKENDTERGQGGFGGRPGGMQMGGPRQDGGPMGGGRFDGPESGAPQMEGRRQNGGPMGGR--------------------------------------------------RFDGPRFGGSRPDGAGGRPFFGQGGRRGDGEEETDAAQQIGDGLGGRGQFD 140 
8-2409    MEVKVTLIVAIVAALAISAHAQRDFNERRGKENDTERGQGGFGGRPGGMQMGGPRQDGGPMGGGRFDGPESGAPQMEGRRQNGGPMGGR--------------------------------------------------RFDGPRFGGSRPDGAGGRPFFGQGGRRGDGEEETDAAQQIGDGLGGRGQFD 140 
8-2410    MEVKVTLIVAIVAALAISAHAQRDFNERRGKENDTERGQGGFGGRPGGMQMGGPRQDGGPMGGGRFDGPESGAPQMEGRRQNGGPMGGR--------------------------------------------------RFDGPRFGGSRPDGAGGRPFFGQGGRRGDGEEETDAAQQIGDGLGGRGQFD 140 
8-2411    MEVKVTLIVAIVAALAISAHAQRDFNERRGKENDTERGQGGFGGRPGGMQMGGPRQDGGPMGGGRFDGPESGAPQMEGRRQNGGPMGGR--------------------------------------------------RFDGPRFGGSRPDGAGGRPFFGQGGRRGDGEEETDAAQQIGDGLGGRGQFD 140 
8-2414    MEVKVTLIVAIVAALAISAHAQRDFNERRGKENDTERGQGGFGGRPGGMQMGGPRQDGGPMGGGRFDGPESGAPQMEGRRQNGGPMGGR--------------------------------------------------RFDGPRFGGSRPDGAGGRPFFGQGGRRGDGEEETDAAQQIGDGLGGRGQFD 140 
8-2417    MEVKVTLIVAIVAALAISAHAQRDFNERRGKENDTERGQGGFGGRPGGMQMGGPRQDGGPMGGGRFDGPESGAPQMEGRRQNGGPMGGR--------------------------------------------------RFDGPRFGGSRPDGAGGRPFFGQGGRRGDGEEETDAAQQIGDGLGGRGQFD 140 
8-2420    MEVKVTLIVAIVAALAISAHAQRDFNERRGKENDTERGQGGFGGRPGGMQMGGPRQDGGPMGGGRFDGPESGAPQMEGRRQNGGPMGGR--------------------------------------------------RFDGPRFGGSRPDGAGGRPFFGQGGRRGDGEEETDAAQQIGDGLGGRGQFD 140 
8-2421    MEVKVTLIVAIVAALAISAHAQRDFNERRGKENDTERGQGGFGGRPGGMQMGGPRQDGGPMGGGRFDGPESGAPQMEGRRQNGGPMGGR--------------------------------------------------RFDGPRFGGSRPDGAGGRPFFGQGGRRGDGEEETDAAQQIGDGLGGRGQFD 140 
8-2422    MEVKVTLIVAIVAALAISAHAQRDFNERRGKENDTERGQGGFGGRPGGMQMGGPRQDGGPMGGGRFDGPESGAPQMEGRRQNGGPMGGR--------------------------------------------------RFDGPRFGGSRPDGAGGRPFFGQGGRRGDGEEETDAAQQIGDGLGGRGQFD 140 
8-2424    MEVKVTLIVAIVAALAISAHAQRDFNERRGKENDTERGQGGFGGRPGGMQMGGPRQDGGPMGGGRFDGPESGAPQMEGRRQNGGPMGGR--------------------------------------------------RFDGPRFGGSRPDGAGGRPFFGQGGRRGDGEEETDAAQQIGDGLGGRGQFD 140 
8-2425    MEVKVTLIVAIVAALAISAHAQRDFNERRGKENDTERGQGGFGGRPGGMQMGGPRQDGGPMGGGRFDGPESGAPQMEGRRQNGGPMGGR--------------------------------------------------RFDGPRFGGSRPDGAGGRPFFGQGGRRGDGEEETDAAQQIGDGLGGRGQFD 140 
8-2427    MEVKVTLIVAIVAALAISAHAQRDFNERRGKENDTERGQGGFGGRPGGMQMGGPRQDGGPMGGGRFDGPESGAPQMEGRRQNGGPMGGR--------------------------------------------------RFDGPRFGGSRPDGAGGRPFFGQGGRRGDGEEETDAAQQIGDGLGGRGQFD 140 
8-2433    MEVKVTLIVAIVAALAISAHAQRDFNERRGKENDTERGQGGFGGRPGGMQMGGPRQDGGPMGGGRFDGPESGAPQMEGRRQNGGPMGGR--------------------------------------------------RFDGPRFGGSRPDGAGGRPFFGQGGRRGGGEEETDAAQQIGDGLGGRGQFD 140 
8-2434    MEVKVTLIVAIVAALAISAHAQRDFNERRGKENDTERGQGGFGGRPGGMQMGGPRQDGGPMGGGRFDGPESGAPQMEGRRQNGGPMGGR--------------------------------------------------RFDGPRFGGSRPDGAGGRPFFGQGGRRGDGEEETDAAQQIGDGLGGRGQFD 140 
8-2435    MEVKVTLIVAIVAALAISAHAQRDFNERRGKENGTERGQGGFGGRPGGMQMGGPRQDGGPMGGGRFDGPESGAPQMEGRRQNGGPMGGR--------------------------------------------------RFDGPRFGGSRPDGAGGRPFFGQGGRRGDGEEETDAAQQIGDGLGGRGQFD 140 
8-2436    MEVKVTLIVAIVAALAISAHAQRDFNERRGKENDTERGQGGFGGRPGGMQMGGPRQDGGPMGGGRFDGPESGAPQMEGRRQNGGPMGGR--------------------------------------------------RFDGPRFGGSRPDGAGGRPFFGQGGRRGDGEEETDAAQQIGDGLGGRGQFD 140 
8-2437    MEVKVTLIVAIVAALAISAHAQRDFNERRGKENDTERGQGGFGGRPGGMQMGGPRQDGGPMGGGRFDGPESGAPQMEGRRQNGGPMGGR--------------------------------------------------RFDGPRFGGSRPDGAGGRPFFGQGGRRGDGEEETDAAQQIGDGLGGRGQFD 140 
8-2438    MEVKVTLIVAIVAALAISAHAQRDFNERRGKENDTERGQGGFGGRPGGMQMGGPRQDGGPMGGGRFDGPESGAPQMEGRRQNGGPMGGR--------------------------------------------------RFDGPRFGGSRPDGAGGRPFFGQGGRRGDGEEETDAAQQIGDGLGGRGQFD 140 
8-2439    MEVRVTLIVAIVAALAISAHAQRDFNERRGKENDTERGQGGFGGRPGGMQMGGPRQDGGPMGGGRFDGPESGAPQMEGRRQNGGPMGGR--------------------------------------------------RFDGPRFGGSRPDGAGGRPFFGQGGRRGDGEEETDAAQQIGDGLGGRGQFD 140 
8-2440    MEVKVTLIVAIVAALAISAHAQRDFNERRGKENDTERGQGGFGGRPGGMQMGGPRQDGGPMGGGRFDGPESGAPQMEGRRQNGGPMGGR--------------------------------------------------RFDGPRFGGSRPDGAGGRPFFGQGGRRGDGEEETDAAQQIGDGLGGRGQFD 140 
8-2442    TEVKVTLIVAIVAALAISAHAQRDFNERRGKENDTERGQGGFGGRPGGMQMGGPRQDGGPMGGGRFDGPESGAPQMEGRRQNGGPMGGR--------------------------------------------------RFDGPRFGGSRPDGAGGRPFFGQGGRRGDGEEETDAAQQIGDGLGGRGQFD 140 
8-2444    MEVKVTLIVAIVAALAISAHAQRDFNERRGKENDTERGQGGFGGRPGGMQMGGPRQDGGPMGGGRFDGPESGAPQMEGRRQNGSPMGGR--------------------------------------------------RFDGPRFGGSRPDGAGGRPFFGQGGRRGDGEEETDAAQQIGDGLGGRGQFD 140 
8-2445    MEVKVTLIVAIVAALAISAHAQRDFSERRGKENDTERGQGGFGGRPGGMQMGGPRQDGGPMGGGRFDGPESGAPQMEGRRQNGGPMGGR--------------------------------------------------RFDGPRFGGSRPDGAGGRPFFGQGGRRGDGEEETDAAQQIGDGLGGRGQFD 140 
8-2448    MEVKVTLIVAIVAALAISAHAQRDFNERRGRENDTERGQGGFGGRPGGMQMGGPRQDGGPMGGGRFDGPESGAPQMEGRRQNGGPMGGR--------------------------------------------------RFDGPRFGGSRPDGAGGRPFFGQGGRRGDGEEETDAAQQIGDGLGGRGQFD 140 
8-2449    MEVKVTLIVAIVAALAISAHAQRDFNERRGKENDTERGQGGFGGRPGGMQMGGPRQDGGPMGGGRFDGPESGAPQMEGRRQNGGPMGGR--------------------------------------------------RFDGPRFGGSRPDGAGGRPFFGQGGRRGDGEEETDAAQQIGDGLGGRGQFD 140 
8-2432    M-VKVTLIVAIVAALAISAHAQRDYNELRGNKNGRERGQGRFGGRPGGMQMGGSRQDGGPMGGRRFDGPDSGAPQMDGRRQDGGPMGGR-------------------------RFDGPGFGAPEMDGRRQNGGPMGGRRFDGPGFGGSRPDGAGGRPFFGQGGRRGDGEEETDAAQQMGDGLGGPGQFD 164 
8-2405    M-VKVTLIVAIVAALAISAHAQRDYNELRGNKNGRERGQGRFGGRPGGMQMGGSRQDGGPVGGRRFDGPDSGAPQMDGRRQDGGPMGGR-------------------------RFDGPGFGAPEMDGRRQNGGPMGGRRFDGPGFGGSRPDGAGGRPFFGQGGRRGDGEEETDAAQQIGDGLGGPGQFD 164 
8-2443    MEVKVTLNVAIVAALAISAHAQRDFNERRGKENDTERGQGGFGGRPGGMQMCGPRQDGGPMGGRRFDGPESGAPQMEGRRQNGGPMGGR--------------------------------------------------RFDGPRFGGSRPDGAGGRPFFGQGGRRGDGEEETDAAQQIGDGLGGRGQFD 140 
8-2413    MEVKVTLIVAIVAALAISAHAQRDFNERRGKENDTERGQGGFGGRPGGMQMGGPRQDGGQMGGRRFDGPESGAPQMEGRRQNGGPMGGR--------------------------------------------------RFDGPRFGGSRPDGAGGRPFFGQGGRRGDGEEETDAAQQIGDGLGGRGQFD 140 
8-2418    MEVKVTLIVAIVAALAISAHAQRDFNERRGKENDTERGQGGFGGRPGGMQMGGLRQDGGPMGGRRFDGPESGAPQMEGRRQNGGPMGGR--------------------------------------------------RFDGPRFGGSRPDGAGGRPFFGQGGRRGDGEEETDAAQQIGDGLGGRGQFD 140 
8-2419    MEVKVTLIVAIVAALAISAHAQRDFNERRGKENDTERGQGGFGGRPGGMQMGGPRQDGGPMGGRRFDGPESGAPQMEGRRQNGGPMGGR--------------------------------------------------RFDGPRFGGSRPDGAGGRPFFGQGGRRGDGEEETDAAQQIGDGLGGRGQFD 140 
8-2423    MEVKVTLIVAIVAALAISAHAQRDFNERRGKENDTERGQGGFGGRPGGMQMGGPRQDGGPMGGRRFDGPESGAPQMEGRRQNGGPMGGR--------------------------------------------------RFDGPRFGGSRPDGAGGRPFFGQGGRRGDGEEETDAAQQIGDGLGGRGQFD 140 
8-2430    MEVKATLIVAIVAALAISAHAQRDFNERRGKENDTERGQGGFGGRPGGMQMGGPRQDGGQMGGRRFDGPESGAPQMEGRRQNGGPMGGR--------------------------------------------------RFDGPRFGGSRPDGAGGRPFFGQGGRRGDGEEETDAAQQIGDGLGGRGQFD 140 
8-2446    MEVKVTLIVAIVAALAISAHAQRDFNERRGKENDTERGQGGFGGRPGGMQMGGPRQDGGPMGGRRFDGPESGAPQMEGRRQNGGPMGGR--------------------------------------------------RFDGPRFGGSRPDGAGGRPFFGQGGRRGDGEEETDAAQQIGDGLGGRGQFD 140 
8-2447    MEVKVTLIVAIVAALAISAHAQRDFNERRGKENDTERGQGGFGGRPGGMQMGGPRQDGGPMGGRRFDGPESGAPQMEGRRQNGGPMGGR--------------------------------------------------RFDGPRFGGSRPDGAGGRPFFGQGGRCGDGEEETDAAQQIGDGLGGRGQFD 140 
8-2441    M-VKVTLIVAIVAALAISAHARRDFNERRGKENGRERGQGGFGGRPGGMQTGSPRQDGGPMGGMRFDGPESGAPQMDGRRQNGGPMGGR--------------------------------------------------RFDGPRFGGSRPDGTGGRPFFGQGGRRGDGEEETDAAQQIGDGLGGPGQFD 139 
8-2404    MEVKVTLIVAIVAALAISTHAQRDFNERRGKENDTERGQGGFGGRPGGMQMGGPRQDGGPMGGRRFDGPESGAPQMDGRRQNGGPMGGR--------------------------------------------------RFDGPRFGGSRPDGAGGRPFFGQGGRRGDGEEETDAAQQIGDGLGGRGQFD 140 
8-2415    MEVKVTLIVAIVAALAISAHAQRDFNERRGKENDTERGQGGFGGRPGGMQMGGPRQDGGPMGGRRFDGPESGAPQMEGRRQNGGPMGGR--------------------------------------------------RFDGPRFGGSRPDGAGGRPFFGQGGRRGDGEEETDAAQQIGDGLGGRGQFD 140 
2-1517    MEVKVTLIVAIVAALAISAHAQRDFNERRGKENDTERGQGGFGGRPGGMQMGGPRQDGGPMGGGRFDGPESGAPQMEGRRQNGGPMGGR--------------------------------------------------RFDGPRFGGSRPDGAGGRPFFGQGGRRGDGEEETDAAQQIGDGLGGRGQFD 140 
2-1501    MEVKVTLIVAIVAALAISAHAQRDFNERRGKENDTERGQGGFGGRPGGMQMGGPRQDGGPMGGGRFDGPESGAPQMEGRRQNGGPMGGR--------------------------------------------------RFDGPRFGGSRPDGAGGRPFFGQGGRRGDGEEETDAAQQIGDGLGGRGQFD 140 
2-1503    MEVKVTLIVAIVAALAISAHAQRDFNERRGKENDTERGQGGFGGRPGGMQMGGPRQDGGPMGGGRFDGPESGAPQMEGRRQNGGPMGGR--------------------------------------------------RFDGPRFGGSRPDGAGGRPFFGQGGRRGDGEEETDAAQQIGDGLGGRGQFD 140 
2-1505    MEVKVTLIVAIVAALAISAHAQRDFNERRGKENDTERGQGGFGGRPGGMQMGGPRQDGGPMGGGRFDGPESGAPQMEGRRQNGGPMGGR--------------------------------------------------RFDGPRFGGSRPDGAGGRPFFGQGGRRGDGEEETDAAQQIGDGLGGRGQFD 140 
2-1508    MEVKVTLIVAIVAALAISAHAQRDFNERRGKENDTERGQGGFGGRPGGMQMGGPRQDGGPMGGGRFDGPESGAPQMEGRRQNGGPMGGR--------------------------------------------------RFDGPRFGGSRPDGAGGRPFFGQGGRRGDGEEETDAAQQIGDGLGGRGQFD 140 
2-1509    MEVKVTLIVAIVAALAISAHAQRDFNERRGKENDTERGQGGFGGGPGGMQMGGPRQDGGPMGGGRFDGPESGAPQMEGRRQNGGPMGGR--------------------------------------------------RFDGPRFGGSRPDGAGGRPFFGQGGRRGDGEEETDAAQQIGDGLGGRGQFD 140 
2-1510    MEVKVTLIVAIVAALAISTHAQRDFNERRGKENDTERGQGGFGGRPGGMQMGGPRQDGGPMGGGRFDGPESGAPQMEGRRQNGGPMGGR--------------------------------------------------RFDGPRFGGSRPDGAGGRPFFGQGGRRGDGEEETDAAQQIGDGLGGRGQFD 140 
2-1519    MEVKVTLIVAIVAALAISAHAQRDFNERRGKENDTERGQGGFGGRPGGMQMGGPRQDGGPMGGGRFDGPESGAPQMEGRRQNGGPMGGR--------------------------------------------------RFDGPRFGGSRPDGAGGRPFFGQGGRRGDGEEETDAAQQIGDGLGGRGQFD 140 
2-1521    MEVKVTLIVAIVAALAISAHAQRDFNERRGKENDTERGQGGFGGRPGGMQMGGPRQDGGPMGGGRFDGPESGAPQMEGRRQNGGPMGGR--------------------------------------------------RFDGPRFGGSRPDGAGGRPFFGQGGRRGDGEEETDAAQQIGDGLGGRGQFD 140 
2-1522    MEVKVTLIVAIVAALAISAHAQRDFNERRGKENDTERGQGGFGGRPGGMQMGGPRQDGGPMGGGRFDGPESGAPQMEGRRQNGGPMGGR--------------------------------------------------RFDGPRFGGSRPDGAGGRPFFGQGGRRGDGEEETDAAQQIGDGLGGRGQFD 140 
2-1523    MEVKVTLIVAIVAALAISAHAQRDFNERRGKENDTERGQGGFGGRPGGMQMGGPRQDGGPMGGGRFDGPESGAPQMEGRRQNGGPMGGR--------------------------------------------------RFDGPRFGGSRPDGAGGRPFFGQGGRRGDGEEETDAAQQIGDGLGGRGQFD 140 
2-1527    MEVKVTLIVAIVAALAISAHAQRDFNERRGKENDTERGQGGFGGRPGGMQMGGPRQDGGPMGGGRFDGPESGAPQMEGRRQNGGPMGGR--------------------------------------------------RFDGPRFGGSRPDGAGGRPFFGQGGRRGDGEEETDAAQQIGDGLGGRGQFD 140 
2-1533    MEVKVTLIVAIVAALAISAHAQRDFNERRGKENDTERGQGGFGGRPGGMQMGGPRQDGGPMGGGRFDGPESGAPQMEGRRQNGGPMGGR--------------------------------------------------RFDGPRFGGSRPDGAGGRPFFGQGGRRGDGEEETDAAQQIGDGLGGRGQFD 140 
2-1535    MEVKVTLIVAIVAALAISAHAQRDFNERRGKENDTERGQGGFGGRPGGMQMGGPRQDGGPMGGGRFDGPESGAPQMEGRRQNGGPMGGR--------------------------------------------------RFDGPRFGGSRPDGAGGRPFFGQGGRRGDGEEETDAAQQIGDGLGGRGQFD 140 
2-1536    MEVKVTLIVAIVAALAISAHAQRDFNERRGKENDTERGQGGFGGRPGGMQMGGPRQDGGPMGGGRFDGPESGAPQMEGRRQNGGPMGGR--------------------------------------------------RFDGPRFGGSRPDGAGGRPFFGQGGRRGDGEEETDAAQQIGDGLGGRGQFD 140 
2-1537    MEVKVTLIVAIVAALAISAHAQRDFNERRGKENDTERGQGGFGGRPGGMQMGGPRQDGGPIGGGRFDGPESGAPQMEGRRQNGGPMGGR--------------------------------------------------RFDGPRFGGSRPDGAGGRPFFGQGGRRGDGEEETDAAQQIGDGLGGRGQFD 140 
2-1538    MEVKVTLIVAIVAALAISAHAQRDFNERRGKENDTERGQGGFGGRPGGMQMGGPRQDGGPMGGGRFDGPESGAPQMEGRRQNGGPMGGR--------------------------------------------------RFDGPRFGGSRPDGAGGRPFFGQGGRRGDGEEETDAAQQIGDGLGGRGQFD 140 
2-1539    MEVKVTLIVAIVAALAISAHAQRDFNERRGKENDTERGQGGFGGRPGGMQMGGPRQDGGPMGGGRFDGPESGAPQMEGRRQNGGPMGGR--------------------------------------------------RFDGPRFGGSRPDGAGGRPFFGQGGRRGDGEEETDAAQQIGDGLGGRGQFD 140 
2-1543    MEVKVTLIVAIVAALAISAHAQRDFNERRGKESDTERGQGGFGGRPGGMQMGGPRQDGGPMGGGRFDGPESGAPQMEGRRQNGGPMGGR--------------------------------------------------RFDGPRFGGSRPDGAGGRPFFGQGGRRGDGEEETDAAQQIGDGLGGRGQFD 140 
2-1546    MEVKVTLIVAIVAALAISAHAQRDFNERRGKENDTERGQGGFGGRPGGMQMGGPRQDGGPMGGGRFDGPESGAPQMEGRRQNGGPMGGR--------------------------------------------------RFDGPRFGGSRPDGAGGRPFFGQGGRRGDGEEETDAAQQIGDGLGGRGQFD 140 
2-1547    MEVKVTLIVAIVAALAISAHAQRDFNERRGKENDTERGQGGFGGRPGGMQMGGPRQDGGPMGGGRFDGPESGAPQMEGRRQNGGPMGGR--------------------------------------------------RFDGPRFGGSRPDGAGGRPFFGQGGRRGDGEEETDAAQQIGDGLGGRGQFD 140 
2-1529    MEVKVTLIVAIVAALAISAHTQRDYNERRGNENGRERGQGRFGGRPGGMQMGGPRQDGGPMGGR-------------------------RFDGHGFGAPPMGGPRQDGGPMGGRRFDGPGFGTPQMDGRRQNGGPMGGRRFDGPRFGGSRPDGAGGRPFFGQGGRRGDGEEETDAAQQIGDGLGGSDRFD 165 
2-1540    M-VKVTLIVAIVAALAISAHAQRDYNELRGNKNGRERGQGRFGGRPGGMQMGGSRQDGGPMGGRRFDGPDSGAPQMDGRRQDGGPMGGR-------------------------RFDGPGFGAPEMDGRRQNGGPMGGRRFDRPGFGGSRPDGAGGRPFFGQGGRCGDGEEETDAAQQIGDGPGGPGQFD 164 
2-1506    MEVKVTLIVAIVAALAISAHAQRDFNERRGKENDTERGQGGFGGRPGGMQMGGPRQDGGPMGGRRFDGPESGAPQMEGRRQNGGPMGGR--------------------------------------------------RFDGPRFGGSRPDGAGGRPFFGQGGRRGDGEEETDAAQQIGDGLGGRGQFD 140 
2-1507    MEVKVTLIVAIVAALAISAHAQRDFNERRGKENDTERGQGGFGGRPGGMQMGGPRQDGGQMGGRRFDGPESGAPQMEGRRQNGGPMGGR--------------------------------------------------RFDGPRFGGSRPDGAGGRPFFGQGGRRGDGEEETDAAQQIGDGLGGRGQFD 140 
2-1511    MEVKVTLIVAIVAALAISAHAQRDFNERRGKENDTERGQGGFGGRPGGMQMGGPRQDGGQMGGRRFDGPESGAPQMEGRRQNGGPMGGR--------------------------------------------------RFDGPRFGGSRPDGAGGRPFFGQGGRRGDGEEETDAAQQIGDGLGGRGQFD 140 
2-1514    MEVKVTLIVAIVAALAISAHAQRDFNERRGKENDTERGQGGFGGRPGGMQMGGPRQDGGPMGGRRFDGPESGAPQMDGRRQNGGPMGGR--------------------------------------------------RFDGPRFGGSRPDGAGGRPFFGQGGRRGDGKEETDAAQQIGDGLGGRGQFD 140 
2-1516    MEVKVTLIVAIVAALAISAHAQRDFNERRGKENDTERGQGGFGGRPGGMQMGGPRQDGGPMGGRRFDGPESGAPQMEGRRQNGGPMGGR--------------------------------------------------RFDGPRFGGSRPDGAGGRPFFGQGGRRGDGEEETDAAQQIGDGLGGRGQFD 140 
2-1528    MEVKVTLIVAIVAALAISAHAQRDFNERRGKENDTERGQGGFGGRPGGMQMGGPRQDGGPMGGRRFDGPESGAPQMEGRRQNGGPMGGR--------------------------------------------------RFDGPRFGGSRPDGAGGRPFFGQGGRRGDGEEETDAAQQIGDGLGGRGQFD 140 
2-1532    MEVKVTLIVAIVAALAISAHAQRDFNERRGKENDTERGQGGFGGRPGGMQMGGPRQDGGPMGGRRFDGPESGAPQMEGRRQNGGPMGGR--------------------------------------------------RFDGPRFGGSRPDGAGGRPFFGQGGRRGDGEEETDAAQQVGDGLGGRGQFD 140 
2-1541    MEVKVTLIVAIVAALAISAHAQRDFNERRGKENDTERGQGGFGGRPGGMQMGGPRQDGGQMGGRRFDGPESGAPQMEGRRQNGGPMGGR--------------------------------------------------RFDGPRFGGSRPDGAGGRPFFGQGGRRGDGEEETDAAQQIGDGLGGRGQFD 140 
2-1542    MEVKVTLIVAIVAALAISAHAQRDFNERRGKENDTERGQGGFGGRPGGMQMGGPRQDGGPMGGRRFDGPESGAPQMEGRRQNGGPMGGR--------------------------------------------------RFDGPRFGGSRPDGAGGRPFFGQGGRRGDGEEETDAAQQVGDGLGGRGQFD 140 
2-2448    MEVKVTLIVAIVAALAISAHAQRDFNERRGKENDTERGQGGFGGRPGGMQMGGPRQDGGPMGGGRFDGPESGAPQMEGRRQNGGPMGGR--------------------------------------------------RFDGPRFGGSRPDGAGGRPFFGQGGRRGDGEEETDAAQQIGDGLGGRGQFD 140 
2-2405    MEVKVTLIVAIVAALAISAHAQRDFNERRGKENDTERGQGGFGGRPGGMQMGGPRQDGGPMGGGRFDGPESGAPQMEGRRQNGGPMGGR--------------------------------------------------RFDGPRFGGSRPDGAGGRPFFGQGGRRGDGEEETDAAQQIGDGLGGRGQFD 140 
2-2406    MEVKVTLIVAIVAALAISAHAQRDFNERRGKENDTERGQGGFGGRPGGMQMGGPRQDGGPMGGGRFDGPESGAPQMEGRRQNGGPMGGR--------------------------------------------------RFDGPRFGGSRPDGAGGRPFFGQGGRRGDGEEETDAAQQIGDGLGGRGQFD 140 
2-2407    MEVKVTLIVAIVAALAISAHAQRDFNERRGKENDTERGQGGFGGRPGGMQMGGPRQDGGPMGGGRFDGPESGAPQMEGRRQNGGPMGGR--------------------------------------------------RFDGPRFGGSRPDGAGGRPFFGQGGRRGDGEEETDAAQQIGDGLGGRGQFD 140 
2-2408    MEVKVTLIVAIVAALAISAHAQRDFNERRGKENDTERGQGGFGGRPGGMQMGGPRQDGGPMGGGRFDGPESGAPQMEGRRQNGGPMGGR--------------------------------------------------RFDGPRFGGSRPDGAGGRPFFGQGGRRGDGEEETDAAQQIGDGLGGRGQFD 140 
2-2410    MEVKVTLIVAIVAALAISAHAQRDFNERRGKENDTERGQGGFGGRPGGMQMGGPRQDGGPMGGGRFDGPESGAPQMEGRRQNGGPMGGR--------------------------------------------------RFDGPRFGGSRPDGAGGRPFFGQGGRRGDGEEETDAAQQIGDGLGGRGQFD 140 
2-2412    MEVKVTLIVAIVAALAISAHAQRDFNERRGKENDTERGQGGFGGRPGGMQMGGPRQDGGPMGGGRFDGPESGAPQMEGRRQNGGPMGGR--------------------------------------------------RFDGPRFGGSRPDGAGGRPFFGQGGRRGDGEEETDAAQQIGDGLGGRGQFD 140 
2-2413    MEVKVTLIVAIVAALAISAHAQRDFNERRGKENDTERGQGGFGGRPGGMQMGGPRQDGGPMGGGRFDGPESGAPQMEGRRQNGGPMGGR--------------------------------------------------RFDGPRFGGSRPDGAGGRPFFGQGGRRGDGEEETDAAQQIGDGLGGRGQFD 140 
2-2419    MEVKVTLIVAIVAALAISAHTQRDFNERRGKENDTERGQGGFGGRPGGMQMGGPRQDGGPMGGGRFDGPESGAPQMEGRRQNGGPMGGR--------------------------------------------------RFDGPRFGGSRPDGAGGRPFFGQGGRRGDGEEETDAAQQIGDGLGGRGQFD 140 
2-2420    MEVKVTLIVAIVAALAISAHAQRDFNERRGKENDTERGQGGFGGRPGGMQMGGPRQDGGPMGGGRFDGPESGAPQMEGRRQNGGPMGGR--------------------------------------------------RFDGPRFGGSRPDGAGGRPFFGQGGRRGDGEEETDAAQQIGDGLGGRGQFD 140 
2-2422    MEVKVTLIVAIVAALAISAHAQRDFNERRGKENDTERGQGGFGGRPGGMQMGGPRQDGGPMGGGRFDGPESGAPQMEGRRQNGGPMGGR--------------------------------------------------RFDGPRFGGSRPDGAGGRPFFGQGGRRGDGEEETDAAQQIGDGLGGRGQFD 140 
2-2424    MEVKVTLIVAIVAALAISAHAQRDFNERRGKENDTERGQGGFGGRPGGMQMGGPRQDGGPMGGGRFDGPESGAPQMEGRRQNGGPMGGR--------------------------------------------------RFDGPRFGGSRPDGAGGRPFFGQGGRRGDGEEETDAAQQIGDGLGGRGQFD 140 
2-2427    MEVKVTLIVAIVAALAISAHAQRDFNERRGKENDTERGQGGFGGRPGGMQMGGPRQDGGPMGGGRFDGPESGAPQMEGRRQNGGPMGGR--------------------------------------------------RFDGPRFGGSRPDGAGGRPFFGQGGRRGDGEEETDAAQQIGDGLGGRGQFD 140 
2-2428    MEVKVTLIVAIVAALAISAHAQRDFNERRGKENDTERGQGGFGGRPGGMQMGGPRQDGGPMGGGRFDGPESGAPQMEGRRQNGGPMGGR--------------------------------------------------RFDGPRFGGSRPDGAGGRPFFGQGGRRGDGEEETDAAQQIGDGLGGRGQFD 140 
2-2429    MEVKVTLIVAIVAALAISAHAQRDFNERRGKENDTERGQGGFGGRPGGMQMGGPRQDGGPMGGGRFDGPESGAPQMEGRRQNGGPMGGR--------------------------------------------------RFDGPRFGGSRPDGAGGRPFFGQGGRRGDGEEETDAAQQIGDGLGGRGQFD 140 
2-2431    MEVKVTLIVAIVAALAISAHAQRDFNERRGKENDTERGQGGFGGRPGGMQMGGPRQDGGPMGGGRFDGPESGAPQMEGRRQNGGPMGGR--------------------------------------------------RFDGPRFGGSRPDGAGGRPFFGQGGRRGDGEEETDAAQQIGDGLGGRGQFD 140 
2-2436    MEVKVTLIVAIVAALAISAHAQRDFNERRGKENDTERGQGGFGGRPGGMQMGGPRQDGGPMGGGRFDGPESGAPQMEGRRQNGGPMGGR--------------------------------------------------RFDGPRFGGSRPDGAGGRPFFGQGGRRGDGEEETDAAQQIGDGLGGRGQFD 140 
2-2441    MEVKVTLIVAIVAALAISAHAQRDFNERRGKENDTERGQGGFGGRPGGMQMGGPRQDGGPMGGGRFDGPESGAPQMEGRRQNGGPMGGR--------------------------------------------------RFDGPRFGGSRPDGAGGRPFFGQGGRRGDGEEETDAAQQIGDGLGGRGQFD 140 
2-2446    MEVKVTLIVAIVAALAISAHAQRDFNERRGKENDTERGQGGFGGRPGGMQMGGPRQDGGPMGGGRFDGPESGAPQMEGRRQNGGPMGGR--------------------------------------------------RFDGPRFGGSRPDGAGGRPFFGQGGRRGDGEEETDAAQQIGDGLGGRGQFD 140 
2-2416    MEVKVTLIVAIVAALAISAHAQRDFNERRGKENDTERGQGGFGGRPGGMQMGGPRQDGGPMGGGRFDGPESGAPQMEGRRQNGGPMGGR--------------------------------------------------RFDGPRFGGSRPDGAGGRPFFGQGGRRGDGEE                    121 
2-2411    M-VKVTLIVAIVAALAISAHAQRDYNELRGNKNGRERGQGRRGVE*                                                                                                                                                 45  
2-2415    M-VKVTLIVAIVAALAISAHAQRDYNELRGNKNGRERGQGRRGVEF*                                                                                                                                                45  
2-2440    M-VKVTLIVAIVAALAISAHAQRDYNELRGNKNGRERGQGRRGVEF*                                                                                                                                                45  
2-2423    ---------------------------------------------------------------------------------------------------------------------------------------------------------------------------------------------- 1   
2-2418    M-VKVTLIVAIVAALAISAHAERDFNERRGKENGRERGQGGFGGRPGGMQTGSPRQDGGPMGGMRFDGPESGAPQMDGRRQNGGPMGGRRFDGPRFGGSRPDGAGGRPFFGQGEDVVMEKKKLMLPNKLVMV*                                                          131 
2-2421    MEVKVTLIVAIVAALAISAHARRDFNERRGKENGRERGQGGFGGRPGGMQTGSPRQDGGPMGGMRFDGPESGAPQMDGRRQNGGPMGGR--------------------------------------------------RFDGPRFGGSRPDGAEGRPFFGQGGRRGDGEEETDAAQQIGDGPGGPGQFD 140 
2-2438    MEVKVTLIVAIVAALAISAHAQRDFNERRGKENDTERGQGGFGGRPGGMQMGGPRQDGGPMGGGRFDGPESGAPQMEGRRQNGGPMGGR--------------------------------------------------RFDGPRFGGSRPDGAGGRPFFGQGGRRGDGEEETDAAQQIGDGLGGPGQFD 140 
2-2414    MEVKVTLIVAIVAALAISAHAQRDFNERRGKENDTERGQGGFGGRPGGMQMGGPRQDGGPMGGRRFDGPESGAPQMEGRRQNGGPMGGR--------------------------------------------------RFDGPRFGGSRPDGAGGRPFFGQGGRRGDGEEETDAAQQVGDGLGGRGQFD 140 
2-2403    MEVKVTLIVAIVAALAISAHAQRDFNERRGKENDTERGQGGFGGRPGGMQMGGPRQDGGPMGGRRFDGPESGAPQMEGRRQNGGPMGGR--------------------------------------------------RFDGPRFGGSRPDGAGGRPFFGQGGRRGDGEEETDAAQQIGDGLGGRGQFD 140 
2-2404    M-VKVTLIVAIVAALAISAHAERDFNERRGKENGRERGQGGFGGRPGGMQTGSPRQDGGPMGGMRFDGPESGAPQMDGRRQNGGPMGGR--------------------------------------------------RFDGPRFGGSRPDGAGGRPFFGQGGRRGDGKEETDAAQQIGDGLGGRGQFD 139 
2-2425    M-VKVTLIVAIVAALAISAHAERDFNERRGKENGRERGQGGFGGRPGGMQTGSPRQDGGPMGGMRFDGPESGAPQMDGRRQNGGPMGGR--------------------------------------------------RFDGPRFGGSRPDGAGGRPFFGQGGRRGDGKEETDAAQQIGDGLGGRGQFD 139 
2-2426    MEVKVTLIVAIVAALAISAHAQRDFNERRGKENDTERGQGGFGGRPGGMQMGGPRQDGGPMGGRRFDGPESGAPQMEGRRQNGGPMGGR--------------------------------------------------RFDGPRFGGSRPDGAGGRPFFGQGGRCGDGEEETDAAQQIGDGLGGRGQFD 140 
2-2430    MEVKVTLIVAIVAALAISAHAQRDFNERRGKENDTERGQGGFGGRPGGMQMGGPRQDGGPMGGRRFDGPESGAPQMEGRRQNGGPMGGR--------------------------------------------------RFDGPRFGGSRPDGAGGRPFFGQGGRRGDGEEETDAAQQIGDGLGGRGQFD 140 
2-2432    M-VKVTLIVAIVAALAISAHAQRDFNERRGKENDTERGQGGFGGRPGGMQMGGPRQDGGPMGGRRFDGPESGAPQMEGRRQNGGPMGGR--------------------------------------------------RFDGPRFGGSRPDGAGGRPFFGQGGRRGDGEEETDAAQQIGDGLGGRGQFD 139 
2-2437    M-VKVTLIVAIVAALAISAHAQRDFNERRGKENGRERGQGGFGGRPGGMQTGSPRQDGGPMGGMRFDGPESGAPQMDGRRQNGGPMGGR--------------------------------------------------RFDGPRFGGSRPDGAGGRPFFGQGGRRGDGKEETDAAQQIGDGLGGRGQFD 139 
2-2439    MEVKVTLIVAIVAALAISAHAQRDFNERRGKENDTERGQGGFGGRPGGMQMGGPRQDGGPMGGRRFDGPESGAPQMEGRRQNGGPMGGR--------------------------------------------------RFDGPRFGGSRPDGAGGRPFFGQGGRRGDGEEETDAAQQIGDGLGGRGQFD 140 
2-2442    MEVKVTLIVAIVAALAISAHAQRDFNERRGKENDTERGQGGFGGRPGGMQMGGPRQDGGPMGGRRFDGPESGAPQMEGRRQNGGPMGGR--------------------------------------------------RFDGPRFGGSRPDGAGGRPFFGQGGRRGDGEEETDAAQQIGDGLGGRGQFD 140 
2-2443    MEVKVTLIVAIVAALAISAHAQRDFNERRGKENDTERGQGGFGGRPGGMQMGGPRQDGGPMGGRRFDGPESGAPQMEGRRQNGGPMGGR--------------------------------------------------RFDGPRFGGSRPDGAGGRPFFGQGGRRGDGEEETDAAQQIGDGLGGRGQFD 140 
2-2445    MEVKVTLIVAIVAALAISAHAQRDFNERRGKENDTERGQGGFGGRPGGMQMGGPRQDGGPMGGRRFDGPESGAPQMEGRRQNGGPMGGR--------------------------------------------------RFDGPRFGGSRPDGAGGRPFFGQGGRRGDGEEETDAAQQIGDGLGGRGQFD 140 
2-2435    MEVKVTLIVAIVAALAISAHARRDFNERRGKENGRERGQGGFGGRPGGMQTGSPRQDGGPMGGMRFDGPESGAPQMDGRRQNGGPMGGR--------------------------------------------------RFDGPRFGGSRPDGAGGRPFSGQGGRRGDGEEETDAAQQIGDGLGGPGQFD 140 
2-2447    MEVKVTLIVAIVAALAISAHARRDFNERRGKENGRERGQGGFGGRPGGMQTGSPRQDGGPMGGMRFDGPESGAPQMDGRRQNGGPMGGR--------------------------------------------------RFDGPRFGGSRPDGAGGRPFFGQGGRRGDGEEETDAAQQIGDGLGGPGQFD 140 
2-2401    MEVKVTLIVAIVAALAISAHARRDFNERRGKENGRERGQGGFGGRPGGMQTGSPRQDGGPMGGMRFDGPESGAPQMDGRRQNGGPMGGR--------------------------------------------------RFDGPRFGGSRPDGAGGRPFFGQGGRRGDGEEETDAAQQIGDGLGGPGQFD 140 
2-2409    M-VKVTLIVAIVAALAISAHAQRDYNELRGNKNGRERGQGRFGGRPGGMQMGGSRQDGGPMGGRRFDGPDSGAPQMDGRRQDGGPMGGR-------------------------RFDGPGFGAPEMDGRRQNGGPMGGRRFDGPGFGGSRPDGAGGRPFFGQGGRRGDGEEETDAAQQIGDGPGGPGQFD 164 
2-2444    M-VKVTLIVAIVAALAISAHAQRDYNELRGNKNGRERGQGRFGGRPGGMQMGGSRQDGGPMGGRRFDGPDSGAPQMDGRRQDGGPMGGR-------------------------RFDGPGFGAPEMDGRRQNGGPMGGRRFDGPGFGGSRPVGAGGRPFFGQGGRRGDEEEETDAAQQIGDGLGGPGQFD 164 
9-1504    MEVKVTLIVAIVAALAISAHAQRDFNERRGKENDTERGQGGFGGRPGGMQMGGPRQDGGPMGGGRFDGPESGAPQMEGRRQNGGPMGGR--------------------------------------------------RFDGPRFGGSRPDGAGGRPFFGQGGRRGDGEEETDAAQQIGDGLGGRGQFD 140 
9-1505    MEVKVTLIVAIVAALAISAHAQRDFNERRGKENDTERGQGGFGGRPGGMQMGGPRQDGGPMGGGRFDGPESGAPQMEGRRQNGGPMGGR--------------------------------------------------RFDGPRFGGSRPDGAGGRPFFGQGGRRGDGEEETDAAQQIGDGLGGRGQFD 140 
9-1506    MEVKVTLIVAIVAALAISAHAQRDFNERRGKENDTERGQGGFGGRPGGMQMGGPRQDGGPMGGGRFDGPESGAPQMEGRRQNGGPMGGR--------------------------------------------------RFDGPRFGGSRPDGAGGRPFFGQGGRRGDGEEETDAAQQIGDGLGGRGQFD 140 
9-1507    MEVKVTLIVAIVAALAISAHAQRDFNERRGKENDTERGQGGFGGRPGGMQMGGPRQDGGPMGGGRFDGPESGAPQMEGRRQNGGPMGGR--------------------------------------------------RFGGPRFGGSRPDGAGGRPFFGQGGRRGDGEEETDAAQQIGDGLGGRGQFD 140 
9-1509    MEVKVTLIVAIVAALAISAHAQRDFNERRGKENDTERGQGGFGGRPGGMQMGGPRQDGGPMGGGRFDGPESGAPQMEGRRQNGGPMGGR--------------------------------------------------RFDGPRFGGSRPDGAGGRPFFGQGGRRGDGEGETDAAQQIGDGLGGRGQFD 140 
9-1512    MEVKVTLIVAIVAALAISAHAQRDFNERRGKENDTERGQGGFGGRPGGMQMGGPRQDGGPMGGGRFDGPESGAPQMEGRRQNGGPMGGR--------------------------------------------------RFDGPRFGGSRPDGAGGRPFFGQGGRRGDGEEETDAAQQIGDGLGGRGQFD 140 
9-1514    MEVKVTLIVAIVAALAISARAQRDFNERRGKENDTERGQGGFGGRPGGMQMGGPRQDGGPMGGGRFDGPESGAPQMEGRRQNGGPMGGR--------------------------------------------------RFDGPRFGGSRPDGAGGRPFFGQGGRRGDGEEETDAAQQIGDGLGGRGQFD 140 
9-1515    MEVKVTLIVAIVAALAISAHAQRDFNERRGKENDTERGQGGFGGRPGGMQMGGPRQDGGPMGGGRFDGPESGAPQMEGRRQNGGPMGGR--------------------------------------------------RFDGPRFGGSRPDGAGGRPFFGQGGRRGDGEEETDAAQQIGDGLGGRGQFD 140 
9-1516    MEVKVTLIVAIVAALAISAHAQRDFNERRGKENDTERGQGGFGGRPGGMQMGGPRQDGGPMGGGRFDGPESGAPQMEGRRQNGGPMGGR--------------------------------------------------RFDGPRFGGSRPDGAGGRPFFGQGGRRGDGEEETDAAQQIGDGLGGRGQFD 140 
9-1518    MEVKVTLIVAIVAALAISAHAQRDFNERRGKENDTERGQGGFGGRPGGMQMGGPRQDGGPMGGGRFDGPESGAPQMEGRRQNGGPMGGR--------------------------------------------------RFDGPRFGGSRPDGAGGRPFFGQGGRRGDGEEETDAAQQIGDGLGGRGQFD 140 
9-1519    MEVKVTLIVAIVAALAISAHAQRDFNERRGKENDTERGQGGFGGRPGGMQMGGPRQDGGPMGGGRFDGPESGAPQMEGRRQNGGPMGGR--------------------------------------------------RFDGPRFGGSRPDGAGGRPFFGQGGRRGDGEEETDAAQQIGDGLGGRGQFD 140 
9-1520    MEVKVTLIVAIVAALAISAHAQRDFNERRGKENDTERGQGGFGGRPGGMQMGGPRQDGGPMGGGRFDGPESGAPQMEGRRQNGGPMGGR--------------------------------------------------RFDGPRFGGSRPDGAGGRPFFGQGGRRGDGEEETDAAQQIGDGLGGRGQFD 140 
9-1521    MEVKVTLIVAIVAALAISAHAQRDFNERRGKENDTERGQGGFGGRPGGMQMGGPRQDGGPMGGGRFDGPESGAPQMEGRRQNGGPMGGR--------------------------------------------------RFDGPRFGGSRPDGAGGRPFFGQGGRRGDGEEETDAAQQIGDGLGGRGQFD 140 
9-1523    MEVKVTLIVAIVAALAISAHAQRDFNERRGKENDTERGQGGFGGRPGGMQMGGPRQDGGPMGGGRFDGPESGAPQMEGRRQNGGPMGGR--------------------------------------------------RFDGPRFGGSRPDGAGGRPFFGQGGRRGDGEEETDAAQQIGDGLGGRGQFD 140 
9-1524    MEVKVTLIVAIVAALAISAHAQRDFNERRGKENDTERGQGGFGGRPGGMQMGGPRQDGGPMGGGRFDGPESGAPQMEGRRQNGGPMGGR--------------------------------------------------RFDGPRFGGSRPDGAGGRPFFGQGGRRGDGEEETDAAQQIGDGLGGRGQFD 140 
9-1526    MEVKVTLIVAIVAALAISAHAQRDFNERRGKENDTERGQGGFGGRPGGMQMGGPRQDGGPMGGGRFDGPESGAPQMEGRRQNGGPMGGR--------------------------------------------------RFDGPRFGGSRPDGAGGRPFFGQGGRRGDGEEETDAAQQIGDGLGGRGQFD 140 
9-1527    MEVKVTLIVAIVAALAISAHAQRDFNERRGKENDTERGQGGFGGRPGGMQMGGPRQDGGPMGGGRFDGPESGAPQMEGRRQNGGPMGGR--------------------------------------------------RFDGPRFGGSRPDGAGGRPFFGQGGRRGDGEEETDAAQQIGDGLGGRGQFD 140 
9-1530    MEVKVTLIVAIVAALAISAHAQRDFNERRGKENDTERGQGGFGGRPGGMQMGGPRQDGGPMGGGRFDGPESGAPQMEGRRQNGGPMGGR--------------------------------------------------RFDGPRFGGSRPDGAGGRPFFGQGGRRGDGEEETDAAQQIGDGLGGRGQFD 140 
9-1531    MEVKVTLIVAIVAALAISAHAQRDFNERRGKENDTERGQGGFGGRPGGMQMGGPRQDGGPMGGGRFDGPESGAPQMEGRRQNGGPMGGR--------------------------------------------------RFDGPRFGGSRPDGAGGRPFFGQGGRRGDGEEETDAAQQIGDGLGGRGQFD 140 
9-1533    MEVKVTLIVAIVAALAISAHAQRDFNERRGKENDTERGQGGFGGRPGGMQMGGPRQDGGPMGGGRFDGPESGAPQMEGRRQNGGPMGGR--------------------------------------------------RFDGPRFGGSRPDGAGGRPFFGQGGRRGDGEEETDAAQQIGDGLGGRGQFD 140 
9-1537    MEVKVTLIVAIVAALAISAHAQRDFNERRGKENDTERGQGGFGGRPGGMQMGGPRQDGGPMGGGRFDGPESGAPQMEGRRQNGGPMGGR--------------------------------------------------RFDGPRFGGSRPDGAGGRPFFGQGGRRGDGEEETDAARQIGDGLGGRGQFD 140 
9-1538    MEVKVTLIVAIVAALAISAHAQRDFNERRGKENDTERGQGGFGGRPGGMQMGGPRQDGGPMGGGRFDGPESGAPQMEGRRQNGGPMGGR--------------------------------------------------RFDGPRFGGSRPDGAGGRPFFGQGGRRGDGEEETDAAQQIGDGLGGRGQFD 140 
9-1539    MEVKVTLIVAIVAALAISAHAQRDFNERRGKENDTERGQGGFGGRPGGMQMGGPRQDGGPMGGGRFDGPESGAPQMEGRRQNGGPMGGR--------------------------------------------------RFDGPRFGGSRPDGAGGRPFFGQGGRRGDGEEETDAAQQIGDGLGGCGQFD 140 
9-1540    MEVKVTLIVAIVAALAISAHAQRDFNERRGKENDTERGQGGFGGRPGGMQMGGPRQDGGPMGGGRFDGPESGAPQMEGRRQNGGPMGGR--------------------------------------------------RFDGPRFGGSRPDGAGGRPFFGQGGRRGDGEEETDAAQQIGDGLGGRGQFD 140 
9-1543    MEVKVTLIVAIVAALAISAHAQRDFNERRGKENDTERGQGGFGGRPGGMQMGGPRQDGGPMGGGRFDGPESGAPQMEGRRQNGGPMGGR--------------------------------------------------RFDGPRFGGSRPDGAGGRPFFGQGGRRGDGEEETDAAQQIGDGLGGRGQFD 140 
9-1544    MEVKVTLIVAIVAALAISAHAQRDFNERRGKENDTERGQGGFGGRPGGMQMGGPRQDGGPMGGGRFDGPESGAPQMEGRRQNGGPMGGR--------------------------------------------------RFDGPRFGGSRPDGAGGRPFFGQGGRRGDGEEETDAAQQIGDGLGGRGQFD 140 
9-1545    MEVKVTLIVAIVAALAISAHAQRDFNERRGKENDTERGQGGFGGRPGGMQMGGPRQDGGPMGGGRFDGPESGAPQMEGRRQNGGPMGGR--------------------------------------------------RFDGPRFGGSRPDGAGGRPFFGQGGRRGDGEEETDAAQQIGDGLGGRGQFD 140 
9-1546    MEVKVTLIVAIVAALAISAHAQRDFNERRGKENDTERGQGGFGGRPGGMQMGGPRQDGGPMGGGRFDGPESGAPQMEGRRQNGGPMGGR--------------------------------------------------RFDGPRFGGSRPDGAGGRPFFGQGGRRGDGEEETDAAQQIGDGLGGRGQFD 140 
9-1547    MEVKVTLIVAIVAALAISAHAQRDFNERRGKENDTERGQGGFGGRPGGMQMGGPRQDGGPMGGGRFDGPESGAPQMEGRRQNGGPMGGR--------------------------------------------------RFDGPRFGGSRPDGAGGRPFFGQGGRRGDGEEETDAAQQIGDGLGGRGQFD 140 
9-1548    MEVKVTLIVAIVAALAISAHAQRDFNERRGKENDTERGQGGFGGRPGGMQMGGPRQDGGPMGGGRFDGPESGAPQMEGRRQNGGPMGGR--------------------------------------------------RFDGPRFGGSRPDGAGGRPFFGQGGRRGDGEEETDAAQQIGDGLGGRGQFD 140 
9-1513    M-VKVTLIVAIVAALAISAHAQRDYNELRGNKNGRERGQGRFGGRPGGMQMGGSRQDGGPMGGRRFDGPDSGAPQMDGRRQDGGPMGGR-------------------------RFDGPGFGAPEMDGRRQNGGPMGGRRFDGPGFGGSRPDGAGGRPFFGQGGRRGDGEEETDAAQQIGDGPGGPGQFD 164 
9-1541    M-VKVTLIVAIVAALAISAHAQRDYNELRGNKNGRERGQGRFGGRPGGMQMGGSRQDGGPMGGRRFDGPDSGAPQMDGRRQDGGPMGGR-------------------------RFDGPGFGAPEMDGRRQNGGPMGGRRFDGPGFGGSRPDGAGGRPFFGQGGRRGDGEEETDAAQQIGDGPGGPGQFD 164 
9-1542    M-VKVTLIVAIVAALAISAHAQRDYNELRGNKNGRERGQGRFGGRPGGMQMGGSRQDGGPMGGRRFDGPDSGAPQMDGRRQDGGPMGGR-------------------------RFDGPGFGAPEMDGRRQNGGPMGGRRFDGPGFGGSRPDGAGGRPFFGQGGRRGDGEEETDAAQQIGDGPGGPGQFD 164 
9-1525    MEVKVTLIVAIVAALAISAHAQSDFNERRGKENGRERGQDRFGGRPDGMQMGGPRQDGGPMGGRRFDGPRFGAPQMGGPRQNGGPMGGRRFDGPGFGAPPMGGPRQDGGPMGGRRFDGPGFGAPQMGGPRQNGGPMGGRRFDGPGFGGSRPDGAGGRPFFGEGGRRGDGEEETDAARQIGPGRFDGPGHG 190 
9-1501    MEVKVTLIVAIVAALAISAHAQRDFNERRGKENDTERGQGGFGGRPGGMQMGGPRQDGGPMGGRRFDGPESGAPQMEGRRQNGGPMGGR--------------------------------------------------RFDGPRFGGSRPDGAGGRPFFGQGGRRGDGEEETDAAQQIGDGLGGRGQFD 140 
9-1502    MEVKVTLIVAIVAALAISAHAQRDFNERRGKENDTERGQGGFGGRPGGMQMGGPRQDGGPMGGRRFDGPESGAPQMEGRRQNGGPMGGR--------------------------------------------------RFDGPRFGGSRPDGAGGRPFFGQGGRRGDGEEETDAAQQIGDGLGGRGQFD 140 
9-1508    MEVKVSLIVAIVAALAISAHAQRDFNERRGKENDTERGQGGFGGRPGGMQMGGPRQDGGPMGGRRFDGPESGAPQMEGRRQNGGPMGGR--------------------------------------------------RFDGPRFGGSRPDGAGGRPFFGQGGRRGDGEEETDAAQQVGDGLGGRGQFD 140 
9-1511    MEVKVTLIVAIVAALAISAHVQRDFNERRGKENDTERGQGGFGGRPGGMQMGGPRQDGGPMGGRRFDGPESGAPQMEGRRQNGGPMGGR--------------------------------------------------RFDGPRFGGSRPDGAGGRPFFGQGGRRGDGEEETDAAQQVGDGLGGRGQFD 140 
9-1517    MEVKVTLIVAIVAALAISAHAQRDFNERRGKENDTERGQGGFGGRPGGMQMGGPRQDGGPMGGRRFDGPESGAPQMEGRRQNGGPMGGR--------------------------------------------------RFDGPRFGGSRPDGAGGRPFFGQGGRRGDGEEETDAAQQIGDGLGGRGQFD 140 
9-1528    MEVKVTLIVAIVAALAISAHAQRDFNERRGKENDTERGQGGFGGRPGGMQMGGPRQDGGPMGGRRFDGPESGAPQMEGRRQNGGPMGGR--------------------------------------------------RFDGPRFGGSRPDGAGGGPFFGQGGRRGDGEEETDAAQQVGDGLGGRGQFD 140 
9-1529    MEVKVTLIVAIVAALAISAHAQRDFNERRGKENDTERGQGGFGGRPGGMQMGGPRQDGGPMGGRRFDGPESGAPQMEGRRQNGGPMGGR--------------------------------------------------RFDGPRFGGSRPDGAGGRPFFGQGGRRGDGEEETDAAQQIGDGLGGRGQFD 140 
9-1534    MEVKVTLIVAIVAALAISAHAQRDFNERRGKENDTERGQGGFGGRPGGMQMGGPRQDGGPMGGRRFDGPESGAPQMEGRRQNSGPMGGR--------------------------------------------------RFDGPRFGGSRPDGAGGRPFFGQGGRRGDGEEETDAAQQVGDGLGGRGQFD 140 
9-1535    MEVKVTLIVAIVAALAISAHAQRDFNERRGKENDTERGQGGFGGRPGGMQMGGPRQDGGPMGGRRFDGPESGAPQMEGRRQNGGPMGGR--------------------------------------------------RFDGPRFGGSRPDGAGGRPFFGQGGRRGDGEEETDAAQQVGDGLGGRGQFD 140 
9-2401    MEVKVTLIVAIVAALAISAHAQRDFNERRGKENDTERGQGGFGGRPGGMQMGGPRQDGGPMGGGRFDGPESGAPQMEGRRQNGGPMGGR--------------------------------------------------RFDGPRFGGSRPDGAGGRPFFGQGGRRGDGEEETDAAQQIGDGLGGRGQFD 140 
9-2402    MEVKVTLIVAIVAALAISAHAQRDFNERRGKENDTERGQGGFGGRPGGMQMGGPRQDGGPMGGGRFDGPESGAPQMEGRRQNGGPMGGR--------------------------------------------------RFDGPRFGGSRPDGAGGRPFFGQGGRRGDGEEETDAAQQIGDGLGGRGQFD 140 
9-2404    MEVKVTLIVAIVAALAISAHAQRDFNERRGKENDTERGQGGFGGRPGGMQMGGPRQDGGPMGGGRFDGPESGAPQMEGRRQNGGPMGGR--------------------------------------------------RFDGPRFGGSRPDGAGGRPFFGQGGRRGDGEEETDAAQQIGDGLGGRGQFD 140 
9-2406    TEVKVTLIVAIVAALAISAHAQRDFNERRGKENDTERGQGGFGGRPGGMQMGGPRQDGGPMGGGRFDGPESGAPQMEGRRQNGGPMGGR--------------------------------------------------RFDGPRFGGSRPDGAGGRPFFGQGGRRGDGEEETDAAQQIGDGLGGRGQFD 140 
9-2408    MEVKVTLIVAIVAALAISAHAQRDFNERRGKENDTERGQGGFGGRPGGMQMGGPRQDGGPMGGGRFDGPESGAPQMEGRRQNGGPMGGR--------------------------------------------------RFDGPRFGGSRPDGAGGRPFFGQGGRRGDGEEETDAAQQIGDGLGGRGQFD 140 
9-2409    MEVKVTLIVAIVAALAISAHAQRDFNERRGKENDTERGQGGFGGRPGGMQMGGPRQDGGPMGGGRFDGPESGAPQMEGRRQNGGPMGGR--------------------------------------------------RFDGPRFGGSRPDGAGGRPFFGQGGRRGDGEEETDAAQQIGDGLGGRGQFD 140 
9-2411    MEVKVTLIVAIVAALAISAHAQRDFNERRGKENDTERGQGGFGGRPGGMQMGGPRQDGGPMGGGRFDGPESGAPQMEGRRQNGGPMGGR--------------------------------------------------RFDGPRFGGSRPDGAGGRPFFGQGGRRGDGEEETDAAQQIGDGLGGRGQFD 140 
9-2413    MEVKVTLIVAIVAALAISAHAQRDFNERREKENDTERGQGGFGGRPGGMQMGGPRQDGGPMGGGRFDGPESGAPQMEGRRQNGGPMGGR--------------------------------------------------RFDGPRFGGSRPDGAGGRPFFGQGGRRGDGEEETDAAQQIGDGLGGRGQFD 140 
9-2414    MEVKVTLIVAIVAALAISAHAQRDFNERRGKENDTERGQGGFGGRPGGMQMGGPRQDGGPMGGGRFDGPESGAPQMEGRRQNGGPMGGR--------------------------------------------------RFDGPRFGGSRPDGAGGRPFFGQGGRRGDGEEETDAAQQIGDGLGGRGQFD 140 
9-2417    MEVKVTLIVAIVATLAISAHAHRDFNERRGKENDTERGQGGFGGRPGGMQMGGPRQDGGPMGGGRFDGPESGAPQMEGRRQNGGPMGGR--------------------------------------------------RFDGPRFGGSRPDGAGGRPFFGQGGRRGDGEEETDAAQQIGDGLGGRGQFD 140 
9-2418    MEVKVTLIVAIVAALAISAHAQRDFNERRGKENDTERGQGGFGGRPGGMQMGGPRQDGGPMGGGRFDGPESGAPQMEGRRQNGGPMGGR--------------------------------------------------RFDGPRFGGSRPDGAGGRPFFGQGGRRGDGEEETDAAQQIGDGLGGRGQFD 140 
9-2419    MEVKVTLIVAIVAALAISAHAQRDFNERRGKENDTERGQGGFGGRPGGMQMGGPRQDGGPMGGGRFDGPESGAPQMEGRRQNGGPMGGR--------------------------------------------------RFDGPRFGGSRPDGAGGRPFFGQGGRRGDGEEETDAAQQIGDGLGGRGQFD 140 
9-2420    MEVKVTLIVAIVAALAISAHAQRDFNERRGKENDTERGQGGFGGRPGGMQMGGPRQDGGPMGGGRFDGPESGAPQMEGRRQNGGPMGGR--------------------------------------------------RFDGPRFGGSRPDGAGGRPFFGQGGRRGDGEEETDAAQQIGDGLGGRGQFD 140 
9-2421    MEVKVTLIVAIVAALAISAHAQRDFNERRGKENDTERGQGGFGGRPGGMQMGGPRQDGGPMGGGRFDGPESGAPQMEGRRQNGGPMGGR--------------------------------------------------RFDGPRFGGSRPDGAGGRPFFGQGGRRGDGEEETDAAQQIGDGLGGRGQFD 140 
9-2424    MEVKVTLIVAIVAALAISAHAQRDFNERRGKENDTERGQGGFGGRPGGMQMGGPRQDGGPMGGGRFDGPESGAPQMEGRRQNGGPMGGR--------------------------------------------------RFDGPRFGGSRPDGAGGRPFFGQGGRRGDGEEETDAAQQIGDGLGGRGQFD 140 
9-2427    MEVKVTLIVAIVAALAISAHAQRDFNERRGKENDTERGQGGFGGRPGGMQMGGPRQDGGPMGGGRFDGPESGAPQMEGRRQNGGPMGGR--------------------------------------------------RFDGPRFGGSRPDGAGGRPFFGQGGRRGDGEEETDAAQQIGDGLGGRGQFD 140 
9-2429    MEVKVTLIVAIVAALAISAHAQRDFNERRGKENDTERGQGGFGGRPGGMQMGGPRQDGGPMGGGRFDGPESGAPQMEGRRQNGGPMGGR--------------------------------------------------RFDGPRFGGSRPDGAGGRPFFGQGGRRGDGEEETDAAQQIGDGLGGRGQFD 140 
9-2430    MEVKVTLIVAIVAALAISAHAQRDFNERRGKENDTERGQGGFGGRPGGMQMGGPRQDGGPMGGGRFDGPESGAPQMEGRRQNGGPMGGR--------------------------------------------------RFDGPRFGGSRPDGAGGRPFFGQGGRRGDGEEETDAAQQIGDGLGGRGQFD 140 
9-2433    MEVKVTLIVAIVAALAISAHAQRDFNERRGKENDTERGQGGFGGRPGGMQMGGPRQDGGPMGGGRFDGPESGAPQMEGRRQNGGPMGGR--------------------------------------------------RFDGPRFGGSRPDGAGGRPFFGQGGRRGDGEEETDAAQQIGDGLGGRGQFD 140 
9-2435    MEVKVTLIVAIVAALAISAHAQRDFNERRGKENDTERGQGGFGGRPGGMQMGGPRQDGGPMGGGRFDGPESGAPQMEGRRQNGGPMGGR--------------------------------------------------RFDGPRFGGSRPDGAGGRPFFGQGGRRGDGEEETDAAQQIGDGLGGRGQFD 140 
9-2438    MEVKVTLIVAIVAALAISAHAQRDFNERRGKENDTERGQGGFGGRPGGMQMGGPRQDGGPMGGGRFDGPESGAPQMEGRRQNGGPMGGR--------------------------------------------------RFDGPRFGGSRPDGAGGRPFFGQGGRRGDGEEETDAAQQIGDGLGGRGQFD 140 
9-2439    MEVKVTLIVAIVAALAISAHAQRDFNERRGKENDTERGQGGFGGRPGGMQMGGPRQDGGPMGGGRFDGPESGAPQMEGRRQNGGPMGGR--------------------------------------------------RFDGPRFGGSIPDGAGGRPFFGQGGRRGDGEEETDAAQQIGDGLGGRGQFD 140 
9-2440    MEVKVTLIVAIVAALAISAHAQRDFNERRGKENDTERGQGGFGGRPGGMQMGGPRQDGGPMGGGRFDGPESGAPQMEGRRQNGGPMGGR--------------------------------------------------RFDGPRFGGSRPDGAGGRPFFGQGGRRGDGEEETDAAQQIGDGLGGRGQFD 140 
9-2441    MEVKVTLIVAIVAALAISAHAQRDFNERRGKENDTERGQGGFGGRPGGMQMGGPRQDGGPMGGGRFDGPESGAPQMEGRRQNGGPMGGR--------------------------------------------------RFDGPRFGGSRPDGAGGRPFFGQGGRRGDGEEETDAAQQIGDGLGGRGQFD 140 
9-2442    MEVKVTLIVAIVAALAISAHAQRDFNERRGKENDTERGQGGFGGRPGGMQMGGPRQDGGPMGGGRFDGPESGAPQMEGRRQNGGPMGGR--------------------------------------------------RFDGPRFGGSRPDGAGGRPFFGQGGRRGDGEEETDAAQQIGDGLGGRGQFD 140 
9-2443    MEVKVTLIVAIVAALAISAHAQRDFNERRGKENDTERGQGGFGGRPGGMQMGGPRQDGGPMGGGRFDGPESGAPQMEGRRQNGGPMGGR--------------------------------------------------RFDGPRFGGSRPDGAGGRPFFGQGGRRGDGEEETDAAQQIGDGLGGRGQFD 140 
9-2445    MEVKVTLIVAIVAALAISAHAQRDFNERRGKENDTERGQGGFGGRPGGMQMGGPRQDGGPMGGGRFDGPESGAPQMEGRRQNGGPMGGR--------------------------------------------------RFDGPRFGGSRPDGAGGRPFFGQGGRRGDGEEGTDAAQQIGDGLGGRGQFD 140 
9-2446    MEVKVTLIVAIVAALAISAHAQRDFNERRGKENDTERGQGGFGGRPGGMQMGGPRQDGGPMGGGRFDGPESGAPQMEGRRQNGGPMGGR--------------------------------------------------RFDGPRFGGSRPDGAGGRPFFGQGGRRGDGEEETDAAQQIGDGLGGRGQFD 140 
9-2448    MEVKVTLIVAIVAALAISAHAQRDFNERRGKENDTERGQGGFGGRPGGMQMGGPRQDGGPMGGGRFDGPESGAPQMEGRRQNGGPMGGR--------------------------------------------------RFDGPRFGGSRPDGAGGRPFFGQGGRRGDGEEETDAAQQIGDGLGGRGQFD 140 
9-2416    M-VKVTLIVAIVAALAISAHAQRDYNELRGNKNGRERGQGRFGGRPGGMQMGGSRQDGGPMGGRRFDGPDSGAPQMDGRRQDGGPMGGR-------------------------RFDGPGFGAPEMDGRRQNGGPMGGRRFDGPGFGGSRPGGAGGRPFFGQGGRRGDGEEETDAAQQIGDGPGGPGQFD 164 
9-2423    M-VKVTLIVAIVAALAISAHAQRDYNELRGNKNGRERGQGRFGGRPGGMQMGGSRQDGGPMGGRRFDGPDSGAPQMDGRRQDGGPMGGR-------------------------RFDGPGFGAPEMDGRRQNGGPMGGRRFDGPGFGGSRPDGAGGRPFFGQGGRRGDGEEETDAAQQIGDGPGGPGQFD 164 
9-2403    MEVKVTLIVAIVAALAISAHAQRDFNERRGKENDTERGQGGFGGRPGGMQMGGPRQDGGPMGGRRFDGPESGAPQMEGRRQNGGPMGGR--------------------------------------------------RFDGPRFGGSRPDGAGGRPFFGQGGRRGDGEEETDAAQQIGDGLGGRGQFD 140 
9-2415    MEVKVTPIVAIVAALAISAHAQRDFNERRGKENDTERGQGGFGGRPGGMQMGGPRQDGGPMGGRRFDGPESGAPQMEGRRQNGGPMGGR--------------------------------------------------RFDGPRFGGSRPDGAGGRPFFGQGGRRGDGEEETDAAQQVGDGLGGRGQFD 140 
9-2422    MEVKVTLIVAIVAALAISAHAQRDFNERRGKENDTERGQGGFGGRPGGMQMGGPRQDGGQMGGRRFDGPESGAPQMEGRRQNGGPMGGR--------------------------------------------------RFDGPRFGGSRPDGAGGRPFFGQGGRRGDGEEETDAAQQIGDGLGGRGQFD 140 
9-2428    MEVKVTLIVAIVAALAISAHAQRDFNERRGKENDTERGQGGFGGRPGGMQMGGPRQDGGPMGGRRFDGPESGAPQMEGRRQNGGPMGGR--------------------------------------------------RFDGPRFGGSRPDGAGGRPFFGQGGRRGDGEEETDAAQQIGDGLGGRGQFD 140 
9-2431    MEVKVTLIVAIVAALAISAHAQRDFNERRGKENDTERGQGGFGGRPGGMQMGGPRQDGGPMGGRRFDGPESGAPQMEGRRQNGGPMGGR--------------------------------------------------RFDGPRFGGSRPDGAGGRPFFGQGGRRGDGEEETDAAQQVGDGLGGRGQFD 140 
9-2432    MEVKVTLIVAIVAALAISAHAQRDFNERRGKENDTERGQGGFGGRPGGMQMGGPRQDGGPMGGRRFDGPESGAPQMEGRRQNGGPMGGR--------------------------------------------------RFDGPRFGGSRPDGAGGRPFFGQGGRRGDGEEETDAAQQIGDGLGGRGQFD 140 
9-2434    MEVKVTLIVAIVAALAISAHAQRDFNERRGKENDTERGQGGFGGRPGGMQMGGPRQDGGPMGGGRFDGPESGAPQMEGRRQNGGPMGGR--------------------------------------------------RFDGPRFGGSRPDGAGGRPFFGQGGRRGDGEEETDAAQQIGDGLGGRGQFD 140 
9-2437    MEVKVTLIVAIVAALAISAHAQRDFNERRGKENDTERGQGGFGGRPGGMQMGGPRQDGGPMGGRRFDGPESGAPQMEGRRQNGGPMGGR--------------------------------------------------RFDGPRFGGSRPDGAGGRPFFGQGGRRGDGEEETDAAQQIGDGLGGRGQFD 140 
9-2444    MEVKVTLIVAIVAALAISAHAQRDFNERRGKENDTERGQGGFGGRPGGMQMGGPRQDGGPMGGRRFDGPESGAPQMEGRRQNGGPMGGR--------------------------------------------------RFDGPRFGGSRPDGAGGRPFFGQGGRRGDGEEETDAAQQVGDGLGGRGQFD 140 
4-1504    M-VKVTLIVAIVAALAISAHAQRDYNELRGNKNGRERGQGRFGGRPGGMQMGGSRQDGGPMGGRRFDGPDSGAPQMDGRRQDGGPMGGR-------------------------RFDGPGFGAPEMDGRRQNGGPMGGRRFDGPGFGGSRPDGAGGRPFFGQGGRRGDGEEETDAAQQIGDGPGGPGQFD 164 
4-1522    MEVKVTLIVAIVAALAISAHAQRDFNERRGKENDTERGQGGFGGRPGGMQMGGPRQDGGPMGGGRFDGPESGAPQMEGRRQNGGPMGGR--------------------------------------------------RFDGPGFGGSRPDGAGGRPFFGQGGRRGDGEEETDAAQQIGDGLGGPGQFD 140 
4-1507    MEVKVTLIVAIVAALAISAHAQRDFNERRGKENDTERGQGGFGGRPGGMQIGGPRQDGGPMGGRRFDGPESGAPQMEGRRQNGGPMGGR--------------------------------------------------RFDGPRFGGSRPDGAGGRPFFGQGGRRGDGEEETDAAQQIGDGLGGRGQFD 140 
4-1519    MEVKVTLIVAIVAALAISAHAQRDFNERRGKENDTERGQGGFGGRPGGMQMGGPRQDGGPMGGGRFDGPESGAPQMEGRRQNGGPMGGR--------------------------------------------------RFDGPRFGGSRPDGAGGRPFFGQGGRRGDGEEETDAAQQIGDGLGGRGQFD 140 
4-1529    MEVKVTLIVAIVAALAISAHAQRDFNERRGKENDTERGQGGFGGRPGGMQMGGPRQDGGPMGGGRFDGPESGAPQMEGRRQNGGPMGGR--------------------------------------------------RFDGPRFGGSRPDGAGGRPFFGQGGRRGDGEEETDAAQQIGDGLGGRGQFD 140 
4-1549    MEVKVTLIVAIVAALAISAHAQRDFNERRGKENDTERGQGGFGGRPGGMQMGGPRQDGGPMGGGRFDGPESGAPQMEGRRQNGGPMGGR--------------------------------------------------RFDGPRFGGSRPDGAGGRPFFGQGGRRGDGEEETDAAQQIGDGLGGRGQFD 140 
4-1510    MEVKVTLIVAIVAALAISAHAQRDFNERRGKENDTERGQGGFGGRPGGMQMGGPRQDGGPMGGGRFDGPESGAPQMEGRRQNGGPMGGR--------------------------------------------------RFDGPRFGGSRPDGAGGRPFFGQGGRRGDGEEETDAAQQIGDGLGGRGQFD 140 
4-1539    MEVKVTLIVAIVAALAISAHAQRDFNERRGKENDTERGQGGFGGRPGGMQMGGPRQDGGPMGGGRFDGPESGAPQMEGRRQNGGPMGGR--------------------------------------------------RFDGPRFGGSRPDGAGGRPFFGQGGRRGDGEEETDAAQQIGDGLGGRGQFD 140 
4-2424    M-VKVTLIVAIVAALAISAHARRDFNERRGKENGRERGQGGFGGRPGGMQTGSPRQDDGPMGGRRFDGPESGAPQMDGRRQNGGPMGGR--------------------------------------------------RFDGPRFGGSRPDGAGGRPFFGQGGRRGDGDEETDAAQQIGDGLGGSGQFD 139 
4-2401    MEVKVTLIVAIVAALAISAHAQRDFNERRGKENDTERGQGGFGGRPGGMQMGGPRQDGGPMGGRRFDGPESGAPQMEGRRQNGGPMGGR--------------------------------------------------RFDGPRFGGSRPDGAGGRPFFGQGGRRGDGEEETDAAQQIGDGLGGRDQFD 140 
4-2405    MEVKVTLIVAIVAALAISAHAQRDFNERRGKENDTERGQGGFGGRPGGMQMGSPRQDGGQMGGRRFDGPESGAPQMEGRRQNGGPMGGR--------------------------------------------------RFDRPRFGGPRPDGAGGRPFFGQGGRRGDGEEETDAAQQIGDGLGGRGQFD 140 
4-2407    MEVKVTLIVAIVAALAISAHAQRDFNERRGKENDTERGQGGFGGRPGGMQMGGPRQDGGPMGGRRFDGPESGAPQMEGRRQNGGPMGGR--------------------------------------------------RFDGPRFGGSRPDGAGGRPFFGQGGRRGDGEEETDAAQQIGDGLGGRDQFD 140 
4-2408    MEVKVTLIVAIVAALAISAHAQRDFNERQGKENDTERGQGGFGGRPGGMQMGGPRQDGGPMGGRRFDGPESGAPQMEGRRQNGGPMGGR--------------------------------------------------RFDGPRFGGSRPDGAGGRPFFGQGGRRGDGEEETDAAQQIGDGLGGRDQFD 140 
4-2411    MEVKVTLIVAIVAALAISAHAQRDFNERRGKENDTERGQGGFGGRPGGMQMGSPRQDGGQMGGRRFDGPESGAPQMEGRRQNGGPMGGR--------------------------------------------------RFDGPRFGGSRPDGAGGRPFFGQGGRRGDGEEETDAAQQIGDGLGGRGQFD 140 
4-2417    MEVKVTLIVAIVAALAISAHAQRDFNERRGKENDAERGQGGFGGRPGGMQMGGPRQDGGPMGGRRFDGPESGAPQMEGRRQNGGPMGGR--------------------------------------------------RFDGPRFGGSRPDGAGGRPFFGQGGRRGDGEEETDAAQQIGDGLGGRDQFD 140 
4-2418    MEVKVTLIVAIVAALAISAHAQRDFNERRGKENDTERGQGGFGGRPGGMQMGGPRQDGGPMGGRRFDGPESGAPQMEGRRQNGGPMGGR--------------------------------------------------RFDGPRFGGSRPDGAGGRPFFGQGGRRGDGEEETDAAQQIGDGLGGRDQFD 140 
4-2419    MEVKVTLIVAIVAALAISAHAQRDFNERRGKENDTERGQGGFGGRPGGMQMGGPRQDGGPMGGRRFDGPESGAPQMEGRRQNGGPMGGR--------------------------------------------------RFDGPRFGGSRPDGAGGRPFFGQGGRRGDGEEETDAAQQIGDGLGGRDQFD 140 
4-2421    MEVKVTLIVAIVAALAISAHAQRDFNERRGKENDTERGQGGFGGRPGGMQMGGPRQDGGPMGGRRFDGPESGAPQMEGRRQNGGPMGGR--------------------------------------------------RFDGPRFGGSRPDGAGGRPFFGQGGRRGDGEEETDAAQRIGDGLGGRDQFD 140 
4-2422    MEVKVTLIVAIVAALAISAHAQRDFNERRGKENDTERGQGGFGGRPGGMQMGGPRQDGGPMGGRRFDGPESGAPQMEGRRQNGGPMGGR--------------------------------------------------RFDGPRFGGSRPDGAGGRPFFGQGGRRGDGEEETDAAQQIGDGLGGRDQFD 140 
4-2426    MEVKVTLIVAIVAALAISAHAQRDFNERRGKENDTERGQGGFGGRPGGMQMGGPRQDGGPMGGRRFDGPESGAPQMEGRRQNGGPMGGR--------------------------------------------------RFDGPRFGGSRPDGAGGRPFFGQGGRRGDGEEETDAAQQIGDGLGGRDQFD 140 
4-2428    MEVKVTLIVAIVAALAISAHAQRDFNERRGKENDTERGQGGFGGRPGGMQMGGPRQDGGPMGGRRFDGPESGAPQMEGRRQNGGPMGGR--------------------------------------------------RFDGPRFGGSRPDGAGGRPFFGQGGRRGDGEEETDAAQQIGDGLGGRDQFD 140 
4-2429    MEVKVTLIVAIVAALAISAHAQRDFNERRGKENDTERGQGGFGGRPGGMQMGGPRQDGGPMGGRRFDGPESGAPQMEGRRQNGGPMGGR--------------------------------------------------RFDGPRFGGSRPDGAGGRPFFGQGGRRGDGEEETDAAQQIGDGLGGRDQFD 140 
4-2435    MEVKATLIVAIVAALAISAHARRDFNERRGNENGRERGQGRFGGRPGGMQMGGSRQDGGPMGGRRFDGPGFGAPHMDGRRQNGGPMGGR--------------------------------------------------RFDGPRFGGSRPDGAGGRPFFGQGGRRGDGEEETDAAQQIGDGLGGRDQFD 140 
4-2437    MEVKVTLIVAIVAALAISAHAQRDFNERRGKENDTERGQGGFGGRPGGMQMGGPRQDGGPMGGRRFDGPESGAPQMEGRRQNGGPMGGR--------------------------------------------------RFDGPRFGGSRPDGAGGRPFFGQGGRRGDGEEETDAAQQVGDGLGGRGQFD 140 
4-2444    MEVKVTLIVAIVAALAISAHAQRDFNERRGKENDTERGQGGFGGRPGGMQMGGPRQDGGPMGGRRFDGPESGAPQMEGRRQNGGPMGGR--------------------------------------------------RFDGPRFGGSRPDGAGGRPFFGQGGRRGDGEEETDAAQQIGDGLGGRDQFD 140 
4-2445    MEVKVTLIVAIVAALAISAHAQRDFNERRGKENDTERGQGGFGGRPGGMQMGGPRQDGGPMGGRRFDGPESGAPQMEGRRQNGVPMGGR--------------------------------------------------RFDGPRFGGSRPDGAGGRPFFGQGGRRGDGEEETDAAQQIGDGLGGRDQFD 140 
4-2447    MEVKVTLIVAIVAALAISAHAQRDFNERRGKENDTERGQGGFGGRPGGMQMGGPRQDGGPMGGRRFDGPESGAPQMEGRRQNGGPMGGR--------------------------------------------------RFDGPRFGGSRPDGAGGRPFFGQGGRRGDGEEETDAAQQIGDGLGGRDQFD 140 
4-2448    MEVKVTLIVAIVAALAISAHAQRDFNERRGKENDTERGQGGFGGRPGGMQMGGPRQDGGPMGGRRFDGPESGAPQMEGRRQNGGPMGGR--------------------------------------------------RFDGPRFGGSRPDGAGGRPFFGQGGRRGDGEEDTDAAQQIGDGLGGRGQFD 140 
4-2404    M-VKVTLIVAIVAALAISAHAQRDYNELRGNKNGRERGQGRFEGRQGGMQMVGSRQDGGPMGGRRFDGPDSGAPQMDGRRQDGGPMGGR-------------------------RFDGPGFGAPEMDGRRQNGGPMGGRRFDGPGFGGSRPDGAGGRPFFGQGGRRGDGEEETDAAQQIGDGLGGPGQFD 164 
4-2413    M-VKVTLIVAIVAALAISAHAQRDYNELRGNKNGRERGQGRFGGRPGGMQMGGSRQDGGPMGGRRFDGPDSGAPQMDGRRQDGGPMGGR-------------------------RFDGPGFGAPEMDGRRQNGGPMGGRRFDGPGFGGSRPVGAGGRPFFGQGGRRGDGEEETDAAQQIGDGLGGPGQFD 164 
4-2450    M-VKVTLIVAIVAALAISAHAQRDYNELRGNKNGRERGQGRFGGRPGGMQMGGSRQDGGPMGGRRFDGPDSGAPQMDGRRQDGGPMGGR-------------------------RFDGPGFGAPEMDGRRQNGGPMGGRRFDGPGFGGSRPVGAGGRPFFGQGGRRGDGEEETDAAQQIGDGLGGPGQFD 164 
4-2420    MEVKVTLIFAIVAALAISAHARRDYNERRGNENGRERGQGRFGGRPGGMQMGGPRQDGGPMGGRRFDGPDSGSPQMDGRRQDGGPMGGR-------------------------RFDGPGFGAPEMDGRRQNGGPMGGRRFDGPGFGGSRPDGAGGRPFFGQGGRRGDGEEETDAAQQIGDGLGGPGQFD 165 
4-2438    MEVKVTLIVAIVAALAISAHAQRDYNELRGNKNGRERGQGRFGGRPGGMQMGGSRQDGGPMGGRRFDGPDSGAPQMDGRRQDGGPMGGR-------------------------RFDGPGFGAPEMDGRRQNGGPMGGRRFDGPGFGGSRPDGAGGRPFFGQGGRRGDGEEETDAAQQIGDGLGGPGQFD 165 
4-2430    MEVKVTLIVAIVAALAISAHAQRDFNERRGKENDTERGQGGFGGRPGGMQMGGPRQDGGPMGGRRFDGPDSGSPQMDGRRQDGGPMGGR-------------------------RFDGPGFGAPEMDGRRQNGGPMGGRRFDGPGFGGSRPDGAGGRPFFGQGGRRGDGEEETDAAQQIGDGLGGPGQFD 165 
4-2439    MEVKVTLIVAIVAALAISAHAQRDFNERRGKENDTERGQGGFGGRPGGMQMGGPRQDGGPMGGRRFDGPESGAPQMEGRRQNGGPMGGR--------------------------------------------------RFDGPRFGGSRPDGAGGRPFFGQGGRRGDGEEETDAAQQIGDGLGGRDQFD 140 
4-2433    MEVKATLIVAIVAALAISAHARRDFNERRGNENGRERGQGRFGGRPGGMQMGGSRQDGGPMGGRRFDGPGFGAPHMDGRRQNGGPMGGR--------------------------------------------------RFDGPRFGGSRPDGAGGRPFFGQGGRRGDGEEETDAAQQIGDGLGGRGQFD 140 
4-2441    MEVKATLIVAIVAALAISAHARRDFNERRGNENGRERGQGRFGGRPGGMQMGGSRQDGGPMGGRRFDGPGFGAPHMDGRRQNGGPMGGR--------------------------------------------------RFDGPRFGGSRPDGAGGRPFFGQGGRRGDGEEETDAAQQIGDGLGGRGQFD 140 
4-2406    MEVKATLIVAILAVLAISAHARRDFNELRGKENGRERGQGRFGGRPDGMQMGGPRQDGGPMGGRRFDGPGFGAPQMGGPRQNGGPMGGRRFDGPRFGAPPMGGPRQDGGPMGGR-------------------------RFDGPGFGGSRPDGAGGRPFFGEGGRRGDGEEETDAARQIDDGLGGPGRFD 165 
4-2425    M-VKVTLIVAIVAALAISAHARRDFNERRGKENGRERGQGGFGGRPGGMQTGSPRQDGGPMGGMRFDGPESGAPQMDGRRQNGGPMGGR--------------------------------------------------RFDGPGFGGSRPDGAGGRPFFGQGGRRGDGEEETDAAQQIGDGLGGPGQFD 139 
4-2432    M-VKVTLIVAIVAALAISAHARRDFNERRGKENGRERGQGGFGGRPGGMQTGSPRQDGGPMGGMRFDGPESGAPQMDGRRQNGGPMGGR--------------------------------------------------RFDGPGFGGSRPDGAGGRPFFGQGGRRGDGEEETDAAQQIGDGLGGPGQFD 139 
4-2440    M-VKVTLIVAIVAALAISAHARRDFNERRGKENGRERGQGGFGGRPGGMQTGSPRQDGGPMGGMRFDGPESGAPQMDGRRQNGGPMGGR--------------------------------------------------RFDGPGFGGSRPDGAGGRPFFGQGGRRGDGEEETDAAQQIGDGLGGPGQFD 139 
4-2402    MEVKVTLIVAIVAALAISAHAQRDFNERRK---------------------------------------------------------------------------------------------------------------------------------------------------------------- 30  
4-2442    ---------------------------------------------------------------------------------------------------------------------------------------------------------------------------------------------- 1   
4-2443    M-MKVTLIVAIVAALAISAHARRDFNERRGKENGRERGQGGFGGRPGGMQTGSPRQDGGPMG*                                                                                                                                61  
4-2416    M-VKVTLIVAIVAALAISAHARRDFNERRGKENGRERGQGGFGGRPGGMQTGSPRQDDGPMGGR-------------------------RFDGPESGAPPSSQQDRRWRSGQTNV*                                                                           89  
4-2446    MEVKVTLIVAIVAALAISAHAQRDFNERRGKENDTERGQGGFGGRPGGMQMGGPRQDGGPMGGGRFDGPESGAPQMEGRRQNGGPMGGR--------------------------------------------------RFDGPRFGGSRPDGAGGRPFFGQGGRRGDGEEETDAAQQIGDGLGGRGQFD 140 
4-2403    MEVKVTLIVAIVAALAISAHAQRDFNERRGKENDTERGQGGFGGRPGGMQMGGPRQDGGPMGGGRFDGPESGAPQMEGRRQNGGPMGGR--------------------------------------------------RFDGPRFGGSRPDGAGGRPFFGQGGRRGDGEEETDAAQQIGDGLGGRGQSD 140 
4-2423    MEVKVTLIVAIVAALAISAHAQRDFNERRGKENDTERGQGGFGGRPGGMQMGGPRQDGGPMGGGRFDGPESGAPQMEGRRQNGGPMGGR--------------------------------------------------RFDGPRFGGSRPDGAGGRPFFGQGGRRGDGEEETDAAQQIGDGLGGRGQFD 140 
4-2409    MEVKVTLIVAIVAALAISAHAQRDFNERRGKENDTERGQGGFGGRPGGMQMGGPRQDGGPTGGGRFDGPESGAPQMEGRRQNGGPMGGR--------------------------------------------------RFDGPRFGGSRPDGAGGRPFFGQGGRRGDGEEETDAAQQIGDGLGGRGQFD 140 
4-2434    MEVEVTLIVAIVAALAISAHAQRDFNERRGKENDTERGQGGFGGRPGGMQMGGPRQDGGPMGGGRFDGPESGAPQMEGRRQNGGPMGGR--------------------------------------------------RFDGPRFGGSRPDGAGGRPFFGQGGRRGDGEEETDAAQQIGDGLGGRGQFD 140 
5-1502    MEVKVTLIVAIVAALAISAHTQRDYNERRGNENGRERGQGRFGGRPGGMQMGGPRQDGGPMGGR-------------------------RFDGHGFGAPPMGGPRQDGGPMGGRRFDGPGFGTPQMDGRRQNGGPMSGRRFDGPRFGGSRPDGAGGRPFFGQGGRRGDGEEETDAAQQIGDGLGGSDRFD 165 
5-1503    MEVKVTLIVAIVAALAISAHTQRDYNERRGNENGRERGQGRFGGRPGGMQMGGPRQDGGPMGGR-------------------------RFDGHGFGAPPMGGPRQDGGPMGGRRFDGPGFGTLQMDGRRQNGGPMGGRRFDGPRFGGSRPDGAGGRPFFGQGGRRGDGEEETDAAQQIGDGLGGSDWFD 165 
5-1504    MEVKVTLIVAIVAALAISAHTQRDYNERRGNENGRGRGQGRFGGRPGGMQMGGPRQDGGPMGGR-------------------------RFDGHGFGAPPMGGPRQDGGPMGGRRFDGPGFGTPQMDGRRQNGGPMGGRRFDGPRFGGSRPDGAGGRPFFGQGGRRGDGEEETDAAQQIGDGLGGSDRFD 165 
5-1505    MEVKVTLIVAIVAALAISAHTHRDYNERRGNENGRERGQGRFGGRPGGMQMGGPRQDGGPMGGR-------------------------RFDGHGFGAPPMGGPRQDGGPMGGRRFDGPGFGTPQMDGRRQNGGPMGGRRFDGPRFGGSRPDGAGGRPFFGQGGRRGDGEEETDAAQQIGDGLGGSDRFD 165 
5-1507    MEVKVTLIVAIVAALAISAHTQRDYNERRGNENGRERGQGRFGGRPGGMQMGGPRQDGGPMGGR-------------------------RFDGHGFGAPPMGGPRQDGGPMGGRRFDGPGFGTPQMDGRRQNGGPMGGRRFDGPRFGGSRPDGAGGRPFFGQGGRRGDGEEETDAAQQIGDGLGGSDRFD 165 
5-1509    MEVKVTLIVAIVAALAISAHTQRDYNERRGNENGRERGQGRFGGRPGGMQMGGPRQDGGPMGGR-------------------------RFDGHGFGAPPMGGPRQDGGPMGGRRFDGPGFGTPQMDGRRQNGGPMGGRRFDGPRFGGSRPDGAGGRPFFGQGGRRGDGEEETDAAQQIGDGLGGSDRFD 165 
5-1511    MEVKVTLIVAIVAALAISAHTQRDYNERRGNENGRERGQGRFGGRPGGMQMGGPRQDGGPMGGR-------------------------RFDGHGFGAPPMGGPRQDGGPMGGRRFDGPGFGTPQMDGRRQNGGPMGGRRFDGPRFGGSRPDGAGGRPFFGQGGRRGDGEEETDAAQQIGDGLGGSDRFD 165 
5-1513    MEVKVTLIVAIVAALAISAHTQRDYNERRGNENGRERGQGRFGGRPGGMQMGGPRQDGGPMGGR-------------------------RFDGHGFGAPPMGGPRQDGGPMGGRRFDGPGFGTPQMDGRRQNGGPMGGRRFDGPRFGGSRPDGAGGRPFFGQGGRRGDGEEETDAAQQIGDGLGGSDRFD 165 
5-1514    MEVKVTLIVAIVAALAISAHTQRDYNERRGNENGRERGQGRFGGRPGGMQMGGPRQDGGPMGGR-------------------------RFDGHGFGAPPMGGPRQDGGPMGGRRFDGPGFGTPQMDGRRQNGGPMGGRRFDGPRFGGSRPDGAGGRPFFGQGGRRGDGEEETDAAQQIGDGLGGSDRFD 165 
5-1508    MEVEVTLIVAIVAALAISAHTQRDYNERRGNENGRERGQGRFGGRPGGMQMGGPRQDGGPMGGR-------------------------RFDGHGFGAPPMGGPRQDGGPMGGRRFDGPGFGTPQMDGRRQNGGPMGGRRFDGPRFGGSRPDGAGGRPFFGQGGRRGDGEEETDAAQQIGDGLGGSDRFD 165 
5-1510    MEVKVTLIVAIVAALAISAHTQRDYNERRGNENGRERGQGRFGGRPGGMQMGGPRQDGGPMGGR-------------------------RFDGHGFGAPPMGGPRQDGGPMGGRRFDGPGFGTPQMDGRRQNGGPMGGRRFDGPRFGGSRPDGAGGRPFFGQGGRRGDGEEETDAAQQIGDGLGGSDRFD 165 
5-2401    M-VKVTLIVAIVAALAISAHAQRDYNELRGNKNGRERGQGRFGGRPGGMQMGGSRQDGGPMGGRRFDGPDSGAPQMDGRRQDGGPMGGR-------------------------RFDGPGFGAPEMDGRRQNGGPMGGRRFDGPGFGGSRPDGAGGRPFFGQGGRRGDGEEETDAAQQIGDGLGGPGQFD 164 
5-2402    M-VKVTLIVAIVAALAISAHAQRDYNELRGNKNGRERGQGRFGGRPGGMQMGGSRQDGGPMGGRRFDGPDSGAPQMDGRRQDGGPMGGR-------------------------RFDGPGFGAPEMDGRRQNGGPMGGRRFDGPGFGGSRPDGAGGRPFFGQRGRRGDGEEETDAAQQIGDGLGGPGQFD 164 
5-2404    M-VKVTLIVAIVAALAISAHAQRDYNELRGNKNGRERGQGRFGGRPGGMQMGGSRQDGGPMGGRRFDGPDSGAPQMDGRRQDGGPMGGR-------------------------RFDGPGFGAPEMDGRRQNGGPMGGRRFDGPGFGGSRPDGAGGRPFFGQGGRRGDGEEETDAAQQIGDGPGGPGQFD 164 
5-2407    M-VKVTLIVAIVAALAISAHAQRDYNELRGNKNGRERGQGRFGGRPGGMQMGGSRQDGGPMGGRRFDGPDSGAPQMDGRRQDGGPMGGR-------------------------RFDGPGFGAPEMDGRRQNGGPMGGRRFDGPGFGGSRPVGAGGRPFFGQGGRRGDGEEETDAAQQIGDGLGGPGQFD 164 
5-2409    M-VKVTLIVAIVAALAISAHAQRDYNELRGNKNGRERGQGRFGGRPGGMQMGGSRQDGGPMGGRRFDGPDSGAPQMDGRRQDGGPMGGR-------------------------RFDGPGFGAPEMDGRRQNGGPMGGRRFDVPGFGGSRPDGAGGRPFFGQGGRRGDGEEETDAAQQIGDGLGGPGQFD 164 
5-2410    MEVKVTLIVAIVAALAISAHAQRDFNDRRGMENDTERGQGGFGGRPGGMQMGGPRQDGGPMGGRRFDGPESGAPQMEGRRQNGGPMGGR--------------------------------------------------RFDGPRFGGSRPDGAGGRPFFGQGGRRGDGEEETDAAQQIGDGLGGRGQFD 140 
5-2411    MEVKVTLIVAIVAALAISAHAQRDFNERRGKENDTERGQGGFGGRPGGMQMGGPRQDGGPMGGRRFDGPESGAPQMEGRRQNGGPMGGR--------------------------------------------------RFDGPRFGGSRPDGAGGRPFFGQGGRRGDGEEETDAAQQIGDGLGGRGQFD 140 
5-2413    MEVKVTLIVAIVAALAISAHAQRDFNERRGKENDTERGQGGFGGRPGGMQMGGPRQDGGPMGGRRFDGPESGAPQMEGRRQNGGPMGGR--------------------------------------------------RFDGPRFGGSRPDGAGGRPFFGQGGRRGDGEEETDAAQQIGDGLGGRGQFD 140 
5-2414    MEVKVTLIVAIVAALAISAHAQRDFNERRGKENDTERGQGGFGGRPGGMQMGGPRQDGGPMGGRRFDGPESGAPQMEGRRQNGGPMGGR--------------------------------------------------RFDGPRFGGSRPDGAGGRPFFGQGGRRGDGEEETDAAQQIGDGLGGRGQFD 140 
5-2415    MEVKVTLIVAIVAALAISAHAQRDFNERRGKENDTERGQGGFGGRPGGMQMGGPRQDGGPMGGRRFDGPESGAPQMEGRRQNGGPMGGR--------------------------------------------------RFDGPRFGGSRPDGAGGRPFFGQGGRRGDGEEETDAAQQIGDGLGGRGQFD 140 
5-2403    MEVKATLIVAIVAALAISAHARRDFNERRGNENGRERGQGRFGGRPGGMQMGGSRQDGGPMGGRRFDGPGFGAPHMDGRRQNGGPMGGR--------------------------------------------------RFDGPRFGGSRPDGAGGRPFFGQGGRRGDGEEETDAAQQIGDGLGGRGQFD 140 
5-2406    M-VKVTLIVAIVAALAISAHARRDFNERRGKENGRERGQGGFGGRPGGMQTGSPRQDGGPMGGMRFDGPESGAPQMDGRRQNGGPMGGR--------------------------------------------------RFDGPRFGGSRPDGAGGRPFFGQGGRRGDGEEETDAAQQIGDGLGGPGQFD 139 
5-2412    MEVKVTLIVAIVAALAISAHAERDFNERRGKENGRERGQGGFGGRPGGMQTGSPRQDGGPIGGMRFDGPESGAPQMDGRRQMAVRWVVGDSTDLDLVAPDQMVLEEDLSSAKEAGVVMEKKKLMLPNKLVMV*                                                          132 
5-2408    M-VKVTLIVAIVAALAISAHAQRDYNELRGNKNGRERTRSLWRKAGWNADGWIEARWWTNGWKKVRWT*                                                                                                                          67  


                  200       210       220       230       240       250       260       270       280       290       300       310       320       330       340       350       360       370       380                  
          ....|....|....|....|....|....|....|....|....|....|....|....|....|....|....|....|....|....|....|....|....|....|....|....|....|....|....|....|....|....|....|....|....|....|....|....|....|....|
1-1515    G--------------------------------------HGRGHHGHRQGPPQDRPEEQPFGQRNESSDEDGRPHPRHHGR-----HH-----QH-HH--------------------------------------------RNHTEGHQGHNETGDHPHRHHNKTGDGDQDRPMFEMRPFRFNPFGRKP 237 
1-1523    G--------------------------------------HGRGHHGHRQGPPQDRPEEQPFGQRNESSDEDGRPHPRHHGR-----HH-----QH-HH--------------------------------------------RNHTEGHQGHNETGDHSHRHHNKTGDGDQDRPMFEMRPFRFNPFGRKP 237 
1-1504    G--------------------------------------HGRGHHGHRQGPPQDRPEEQPFGQRNESSDEDGRPHPRHHGR-----HH-----QH-HH--------------------------------------------RNHTEGHQGHNETGDHPHRHHNKTGDGDQDRPMFEMRPFRFNPFGRKP 237 
1-1533    G--------------------------------------HGRGHHGHRQGPPQDRPEDQPFGQRNESSDEDGRPHPRHHGR-----HH-----QH-HH--------------------------------------------RNHTEGHQGHNETGDHPHRHHNKTGDGDQDRPMFEMRPFRFNPFGRKP 237 
1-1547    G--------------------------------------H                                                                                                                                                       142 
1-1549    G--------------------------------------H                                                                                                                                                       142 
1-1505    G--------------------------------------H                                                                                                                                                       142 
1-1512    G--------------------------------------H                                                                                                                                                       142 
1-1514    G--------------------------------------H                                                                                                                                                       142 
1-1528    G--------------------------------------H                                                                                                                                                       142 
1-1532    G--------------------------------------H                                                                                                                                                       142 
1-1539    G--------------------------------------H                                                                                                                                                       142 
1-1536    G--------------------------------------H                                                                                                                                                       142 
1-1535    G--------------------------------------H                                                                                                                                                       142 
1-1534    G--------------------------------------H                                                                                                                                                       142 
1-2402    G--------------------------------------HGRGHHGHRQGPPQDRPEEQPFGQRNESSDEDGRPHPRHHGR-----HH-----QH-HH--------------------------------------------RNHTEGHQGHNETGDHPHRHHNKTGDGDQDRPMFEMRPFRFNPFGRKP 237 
1-2404    G--------------------------------------HGRGHHGHRQGPPHDRPEEQPFGQRNESSDEDGRPHPRHHGR-----HH-----QH-HH--------------------------------------------RNHTEGHQGHNETGDHPHRHHNKTGDGDQDRPMFEMRPFRFNPLGRKP 237 
1-2405    G--------------------------------------HGRGHHGHRQGPPQDRPEEQPFGQRNESSDEDGRPHPRHHGR-----HH-----QH-HH--------------------------------------------RNHTEGHQGHNETGDHPHRHHNKTGDGDQDRPMFEMRPFRFNPFGRKP 237 
1-2406    G--------------------------------------HGRGHHGHRQGPPQDRPEEQPFGQRNESSDEDGRPHPRHHGR-----HH-----QH-HH--------------------------------------------RNHTEGHQGHNETGDHPHRHHNKTGDGDQDRPMFEMRPFRFNPFGRKP 237 
1-2407    G--------------------------------------HGRGHHGHRQGPPHDRPEEQPFGQRNESSDEDGRPHPRHHGR-----HH-----QH-HH--------------------------------------------RNHTEGHQGHNETGDHPHRHHNKTGDGDQDRPMFEMRPFRFNPFGRKP 237 
1-2412    G--------------------------------------HGRGHHGHRQGPPQDRPEEQPFGQRNESSDEDGRPHPRHHGR-----HH-----QH-HH--------------------------------------------RNHTEGHQGHNETGDHPHRHHNKTGDGDQDRPMFEMRPFRFNPFGRKP 237 
1-2413    G--------------------------------------HGRGHHGHRQGPPQDRPEEQPFGQRNESSDEDGRPHPRHHGR-----HH-----QH-HH--------------------------------------------RNHTEGHQGHNETGDHPHRHHNKTGDGDQDRPMFEMRPFRFNPFGRKP 237 
1-2416    G--------------------------------------HGRGHHGHRQGPPHDRPEEQPFGQRNESSDEDGRPHPRHHGR-----HH-----QH-HH--------------------------------------------RNHTEGHQGHNETGDHPHRHHNKTGDGDQDRPMFEMRPFRFNPFGRKP 237 
1-2417    G--------------------------------------HGRGHHGHRQGPPHDRPEEQPFGQRNESSDEDGRPHPRHHGR-----HH-----QH-HH--------------------------------------------RNHTEGHQGHNETGDHPHRHHNKTGDGDQDRPMFEMRPFRFNPFGRKP 237 
1-2418    G--------------------------------------HGRGHHGHRQGPPQDRPEEQPFGQRNESSDEDGRPHPRHHGR-----HH-----QH-HH--------------------------------------------RNHTEGHQGHNETGDHPHRHHNKTGDGDQDRPMFEMRPFRFNPFGRKP 237 
1-2420    G--------------------------------------HGRGHHGHRQGPPHDRPEEQPFGQRNESSDEDGRPHPRHHGR-----HH-----QH-HH--------------------------------------------RNHTEGHQGHNETGDHPHRHHNKTGDGDQDRPMFEMRPFRFNPFGRKP 237 
1-2421    G--------------------------------------HGRGHHGHRQGPPHDRPEEQPFGQRNESSDEDGRPHPRHHGR-----HH-----QH-HH--------------------------------------------RNHTEGHQGHNETGDHPHRHHNKTGDGDQDRPMFEMRPFRFNPFGRKP 237 
1-2422    G--------------------------------------HGRGHHGHRQGPPQDRPEEQPFGQRNESSDEDGRPHPRHHGR-----HH-----QH-HH--------------------------------------------RNHTEGHQGHNETGDHPHRHHNKTGDGDQDRPMFEMRPFRFNPFGRKP 237 
1-2425    G--------------------------------------HGRGHHGHRQGPPQDRPEEQPFGQRNESSDEDGRPHPRHHGR-----HH-----QH-HH--------------------------------------------RNHTEGHQGHNETGDHPHRHHNKTGDGDQDRPMFEMRPFRFNPFGRKP 237 
1-2426    G--------------------------------------HGRGHHGHRQGPPQDRPEEQPFGQRNESSDEDGRPHPRHHGR-----HH-----QH-HH--------------------------------------------RNHTEGHQGHNETGDHPHRHHNKTGDGDQDRPMFEMRPFRFNPFGRKP 237 
1-2427    G--------------------------------------HGRGHHGHRQGPPQDRPEEQPFGQRNESSDEGGRPHPRHHGR-----HH-----QH-HH--------------------------------------------RNHTEGHQGHNETGDHPHRHHNKTGDGDQDRPMFEMRPFRFNPFGRKP 237 
1-2428    G--------------------------------------HGRGHHGHRQGPPQDRPEEQPFGQRNESSDEDGRPHPRHHGR-----HH-----QH-HH--------------------------------------------RNHTEGHQGHNETGDHPHRHHNKTGDGDQDRPMFEMRPFRFNPFGRKP 237 
1-2430    G--------------------------------------HGRGHHGHRQGPPQDRPEEQPFGQRNESSDEDGRPHPRHHGR-----HH-----QH-HH--------------------------------------------RNHTEGHQGHNETGDHPHRHHNKTGDGDQDRPMFEMRPFRFNPFGRKP 237 
1-2431    G--------------------------------------HGRGHHGHRQGPPHDRPEEQPFGQRNESSDEDGRPHPRHHGR-----HH-----QH-HH--------------------------------------------RNHTEGHQGHNETGDHPHRHHNKTGDGDQDRPMFEMRPFRFNPFGRKP 237 
1-2432    G--------------------------------------HGRGHHGHRQGPPQDRPEEQPFGQRNESSDEDGRPHPRHHGR-----HH-----QH-HH--------------------------------------------RNHTEGHQGHNETGDHPHRHHNKTGDGDQDRPMFEMRPFRFNPFGRKP 237 
1-2433    G--------------------------------------HGRGHHGHRQGPPQDRPEEQPFGQRNESSDEDGRPHPRHHGR-----HH-----QH-HH--------------------------------------------RNHTEGHQGHNETGDHPHRHHNKTGDGDQDRPMFEMRPFRFNPFGRKP 237 
1-2434    G--------------------------------------HGRGHHGHRQGPPRDRPEEQPFGQRNESSDEDGRPHPRHHGR-----HH-----QH-HH--------------------------------------------RNHTEGHQGHNETGDHPHRHHNKTGDGDQDRPMFEMRPFRFSPFGRKP 237 
1-2435    G--------------------------------------HGRGHHGHRQGPPHDRPEEQPFGQRNESSDEDGRPHPRHHGR-----HH-----QH-HH--------------------------------------------RNHTEGHQGHNETGDHPHRHHNKTGDGDQDRPMFEMRPFRFNPFGRKP 237 
1-2436    G--------------------------------------HGRGHHGHRQGPPQDRPEEQPFGQRNESSDEDGRPHPRHHGR-----HH-----QH-HH--------------------------------------------RNHTEGHQGHNETGDHPHRHHNKTGDGDQDRPMFEMRPFRFNPFGRKP 237 
1-2437    G--------------------------------------HGRGHHGHRQGPPQDRPEEQPFGQRNESSDEDGRPHPRHHGR-----HH-----QH-HH--------------------------------------------RNHTEGHQGHNETGDHPHRHHNKTGDGDQDRPMFEMRPFRFNPFGRKP 237 
1-2439    G--------------------------------------HGRGHHGHRQGPPHDRPEEQPFGQRNESSDEDGRPHPRHHGR-----HH-----QH-HH--------------------------------------------RNHTEGHQGHNETGDHPHRHHNKTGDGDQDRPMFEMRPFRFNPFGRKP 237 
1-2440    G--------------------------------------HGRGHHGHRQGPPHDRPEEQPFGQRNESSDEDGRPHPRHHGR-----HH-----QH-HH--------------------------------------------RNHTEGHQGHNETGDHPHRHHNKTGDGDQDRPMFEMRPFRFNPFGRKP 237 
1-2441    G--------------------------------------HGRGHHGHRQGPPQDRPEEQPFGQRNESSDEDGRPHPRHHGR-----HH-----QH-HH--------------------------------------------RNHTEGHQGHNETGDHPHRHHNKTGDGDQDRPMFEMRPFRFNPFGRKP 237 
1-2442    G--------------------------------------HGRGHHGHRQGPPHDRPEEQPFGQRNESSDEDGRPHPRHHGR-----HH-----QH-HH--------------------------------------------RNHTEGHQGHNETGDHPHRHHNKTGDGDQDRPMFEMRPFRFNPFGRKP 237 
1-2414    GPGRRHHG--------------------------------------HRQGHPQDQAEEQPFGQRNESSEEDGRPHPHHRGH---HGHH----HR--HHNHTEGHQGHN-ETGDQDQDKLHDTRPFRYNHFGRKPFGDRPFGRRNHTEGHQGHNETGDHPHRHHNKTRDGDQDRPMFEMRPFRFNPFGRKP 306 
1-2424                                                                                                                                                                                                   58  
1-2429                                                                                                                                                                                                   58  
2-1501    GPRRGHHG--------------------------------------HRQGPPQDRPEEQPFGQRNYSSEEDGRPHPHHHRH---HGHHRHH---H-HHNQTEGHQGHN-ETGDQDQDKPIDTRPFRFNHFGRKPFGGRPFGRRDHTEGHQGHNETGDHPHRHHNKTGDGDQDRPMFESRPFRFNPFGRKP 309 
2-1502    GPRRGHHG--------------------------------------HRQGPPQDRPEEQPFGQRNYSSEEDGRPHPHHHGH---HGHHRHH---H-HHNQTEGHQGHN-ETGDQDQDKPIDTRPFRFNHFGRKPFGGRPFGRRNHTEGHQGHNETGDHPHRHHNKTGDGDQDRPMFESRPFRFNPFGRKP 309 
2-1505    GPRRGHHG--------------------------------------HRQGPPQDRPEEQPFGQRNYSSEEDGRPHPHHHGH---HGHHRHH---H-HHNQTEGHQGHN-ETGDQDQDKPIDTRPFRFNHFGRKPFGGRPFGRRNHTEGHQGHNETGDHPHRHHNKTGDGDQDRPMFESRPFRFNPFGRKP 309 
2-1506    GPRRGHHG--------------------------------------HRQGPPQDRPEEQPFGQRNYSSEEDGDLHPHHHRH---HGHHRHH---H-HHNQTEGHQGHN-ETGDQDQDKPIDTRPFRFNHFGRKPFGGRPFGRRNHTEGHQGHNETGDHPHRHHNKTGDGDQDRPMFESRPFRFNPFGRKP 309 
2-1507    GPRRGHHG--------------------------------------HRQGPPQDRPEEQPFGQRNYSSEEDGRPHPHHHRH---HGHHRHH---H-HHNQTEGHQGHN-ETGDQDQDKPIDTRPFRFNHFGRKPFGGRPFGRRNHTEGHQGHNETGDHPHRHHNKTGDGDQDRPMFESRPFRFNPFGRKP 309 
2-1508    GPRRGHHG--------------------------------------HRQGPPQDRPEEQPFGQRNYSSEEDGRPHPHHHGH---HGHHRHH---H-HHNQTEGHQGHN-ETGDQDQDKPIDTRPFRFNHFGRKPFGGRPFGRRNHTEGHQGHNETGDHPHRHHNKTGDGDQDRPMFESRPFRFNPFGRKP 309 
2-1509    GPRRGHHG--------------------------------------HRQGPPQDRPEEQPFGQRNYSSEEDGRPHPHHHRH---HGHHRHH---H-HHNQTEGHQGHN-ETGDQDQDKPIDTRPFRFNHFGRKPFGGRPFGRRNHTEGHQGHNETGDHPHRHHNKTGDGDQDRPMFESRPFRFNPFGRKP 309 
2-1510    GPRRGHHG--------------------------------------HRQGPPQDRPEEQPFGQRNYSSEEDGRLHPHHHRH---HGHHRHH---H-HHNQTEGHQGHN-ETGDQDQDKPIDTRPFRFNHFGRKPFGGRPFGRRNHTEGHRGHNETGDHPHRHHNKTGDGDQDRPMLESRPFRFNPFGRKP 309 
2-1514    GPRRGHHG--------------------------------------HRQGPPQDRPEEQPFGQRNYSSEEDGRPHPHHHRH---HGHHRHH---H-HHNQTEGHQGHN-ETGDQDQDKPIDTRPFRFNHFGRKPFGGRPFGRRNHTEGHQGHNETGDHPHRHHNKTGDGDQDRPMFESRPFRFNPFGRKP 309 
2-1511    GPRRGHHG--------------------------------------HRQGPPQDRPEEQPFGQRNYSSEEDGRPHPHHHRH---HGHHRHH---H-HHDQTEGHQGHN-ETGDQDQDKPIDTRPFRFNHFGRKPFGGRPFGRRNHTEGHQGHNETGDHPHRHHNKTGDGDQDRPMFESRPFRFNPFGRKP 309 
2-2401    GPRRGHHG--------------------------------------HRQGPPQDRPEEQPFGQRNESSDEDGRPHPRHHGR-----HH-----QH-HH--------------------------------------------RNHTEGHQGHNETGDHPHRHHNKTGDGDQDRPMFEMRPFRFNPFGRKP 236 
2-2404    GPRRGHHG--------------------------------------HRQGPPQDRPEEQPFGQRNESSDEDGRPHPRHHGR-----HH-----QH-HH--------------------------------------------RNHTEGHQGHNETGDHPHRHHNKTGDGDQDRPMFEMRPFRFNPFGRKP 236 
2-2406    G--------------------------------------HGRGHHGHRQGPPQDRPEEQPFGQRNESSDEDGRPHPRHHGR-----HH-----QH-HH--------------------------------------------RNHTEGHQGHNETGDHPHRHHNKTGDGDQDRPMFEMRPFRFNPFGRKP 236 
2-2407    GPRRGHHG--------------------------------------HRQGPPQDRPEEQPFGQRNESSDEDGRPHPRHHGR-----HH-----QH-HH--------------------------------------------RNHTEGHQGHNETGDHPHRHHNKTGDGDQDRPMFEMRPFRFNPFGRKP 236 
2-2408    GPRRGHHG--------------------------------------HRQGPPQDRPEEQPFGQRNESSDEDGRPHPRHHGR-----HH-----QH-HH--------------------------------------------RNHTEGHQGHNETGDHPHRHHNKTGDGDQDRPMFEMRPFRFNPFGRKP 236 
2-2409    GPRRGHHG--------------------------------------HRQGPPQDRPEEQPFGQRNESSDEDGRPHPRHHGR-----HH-----QH-HH--------------------------------------------RNHTEGHQGHNETGDHPHRHHNKTGDGDQDRPMFEMRPFRFNPFGRKP 236 
2-2411    GPRRGHHG--------------------------------------HRQGPPQDRPEEQPFGQRNESSDEDGRPHPRHHGR-----HH-----QH-HH--------------------------------------------RNHTEGHQGHNETGDHPHRHHNKTGDGDQDRPMFEMRPFRFNPFGRKP 236 
2-2405    G--------------------------------------HGRRHHGHRQGPPQDRPEEQPFGQRNERNEEDGRPHPHHHGH-----HHRHH-------NQTEGHQGHN-ETGDQDQDKPNDTRPFRFNHFG----------RRNHTEGHQGHNETGDHPHRHHNKTGDRDQDRPMFEMR----------- 258 
2-2413    G--------------------------------------HGRRHHGHRQGPPQDRPEEQPFGQRNERNEEDGRPHPHHHGH-----HHRHH-------NRTEGHQGHN-ETGDQDQDKPNDTRPFRFNHFG----------RRNHTEGHQGHNETGDHPHRHHNKTGDRDQDRPMFEMR----------- 258 
2-2403    GPGRRHHG---------------------------------------------------------------------------------------------------------------------------RKPFGDRPFGRRNHTEGHQGHNETGDHPHRHHNKTRDGDQDRPMFEMRPFRFNPFGRKP 207 
2-2415    GPGRRHHG--------------------------------------HRQGHPQDQAEEQPFGQRNESSEEDGRPHPHHHRH---HGHH----HR--HHNHTEGHQGHN-ETGDQDQDKLHDTRPFRYNHFGRKPFGDRPFGRRNHTEGHQGHNETGDHPHRHHNKTRDGDQDRPMFEMRPFRFNPFGRKP 306 
2-2414    GPGRRHHG--------------------------------------HRQGHPQDQAEEQPFGQRNESSEEDGLPHPHHHRH---HGHH----HR--HHNHTEGHQGHN-ETGDQDQDKLHDTRPFRYNHFGRKPFGDRPFGRRNHTEGHQGHNETGDHPHRHHNKTRDGDQDRPMFEMRPFRFNPFGRKP 306 
3-15-1006 GPRRGHHG--------------------------------------HRQGPPQDRPEEQPFGQRNYSSEEDGRPHPHHHRH---HGHHRHH---H-HHNQTEGHQGHN-ETGDQDQDKPIDTRPFRFNHFGRKPFGGRPFGRRNHTEGHQGHNETGDHPHRHHNKTGDGDQDRPMFESRPFRFNPFGRKP 309 
3-15-4003 GPRRGHHG--------------------------------------HRQGPPQDRPEEQPFGQRNYSSEEDGRPHPHHHRH---HGHHRHH---H-HHNQTEGHQGHN-ETGDQDQDKPIDTRPFRFNHFGRKPFGGRPFGRRNHTEGHQGHNETGDHPHRHHNKTGDGDQDRPMFESRPFRFNPFGRKP 309 
3-15-4004 DPRRGHHG--------------------------------------HRQGPPQDRPEEQPFGQRNYSSEEDGRPHPHHHRH---HGHHRHH---H-HHNQTEGHQGHN-ETGDQDQDKPIDTRPFRFNHFGRKPFGGRPFGRRNHTEGHQGHNETGDHPHRHHNKTGDGDQDRPMFESRPFRFNPFGRKP 309 
3-15-4005 GPRRGHHG--------------------------------------HRQGPPQDRPEEQPFGQRNYSSEEDGRPHPHHHRH---HGHHRHH---H-HHNQTEGHQGHN-ETGDQDQDKPIDTRPFRFNHFGRKPFGGRPFGRRNHTEGHQGHNETGDHPHRHHNKTGDGDQDRPMFESRPFRFNPFGRKP 309 
3-15-4007 GPRRGHHG--------------------------------------HRQGPPQDRPEEQPFGQRNYSSEEDGRPHPHHHRH---HGHHRHH---H-HHNQTEGHQGHN-ETGDQDQDKPIDTRPFRFNHFGRKPFGGRPFGRRNHTEGHQGHNETGDHPHRHHNKTGDGDQDRPMFESRPFRFNPFGRKP 309 
3-15-4011 GPRRGHHG--------------------------------------HRQGPPQDRPEEQPFGQRNYSSEEDGRPHPHHHRH---HGHHRHH---H-HHNQTEGHQGHN-ETGDQDQDKPIDTRPFRFNHFGRKPFGGRPFGRRNHTEGHQGHNETGDHPHRHHNKTGDGDQDRPMFESRPFRFNPFGRKP 309 
3-15-4013 GPRRGHHG--------------------------------------HRQGPPQDRPEEQPFGQRNYSSEEDGRPHPHHHRH---HGHHRHH---H-HHNQTEGHQGHN-ETGDQDQDKPIDTRPFRFNHFGRKPFGGRPFGRRNHTEGHQGHNETGDHPHRHHNKTGDGDQDRPMFESRPFRFNPFGRKP 309 
3-15-4015 GPRRGHHG--------------------------------------HRQGPPQDRPEEQPFGQRNYSSEEDGRPHPHHHRH---HGHHRHH---H-HHNQTEGHQGHN-ETGDQDQDKPIDTRPFRFNHFGRKPFGGRPFGRRNHTEGHQGHNETGDHPHRHHNKTGDGDQDRPMFESRPFRFNPFGRKP 309 
3-15-4018 GPRRGHHG--------------------------------------HRQGPPQDRPEEQPFGQRNYSSEEDGRPHPHHHRH---HGHHRHH---H-HHNQTEGHQGHN-ETGDQDQDKPIDTRPFRFNHFGRKPFGGRPFGRRNHTEGHQGHNETGDHPHRHHNKTGDGDQDRPMFESRPFRFNPFGRKP 309 
3-15-4019 GPRRGHHG--------------------------------------HRQGPPQDRPEEQPFGQRNYSSEEDGRPHPHHHRH---HGHHRHH---H-HHNQTEGHQGHN-ETGDQDQDKPIDTRPFRFNHFGRKPFGGRPFGRRNHTEGHQGHNETGDHPHRHHNKTGDGDQDRPMFESRPFRFNPFGRKP 309 
3-15-4022 GPRRGHHG--------------------------------------HRQGPPQDRPEEQPFGQRNYSSEEDGRPHPHHHRH---HGHHRHH---H-HHNQTEGHQGHN-ETGDQDQDKPIDTRPFRFNHFGRKPFGGRPFGRRNHTEGHQGHNETGDHPHRHHNKTGDGDQDRPMFESGPFRFNPFGRKP 309 
3-15-4024 GPRRGHHG--------------------------------------HRQGPPQDRPEEQPFGQRNYSSEEDGRPHPHHHRH---HGHHRHH---H-HHNQTEGHQGHN-ETGDQDQDKPIDTRPFRFNHFGRKPFGGRPFGRRNHTEGHQGHNETGDHPHRHHNKTGDGDQDRPMFESRPFRFNPFGRKP 309 
3-15-4017 GPRRGHHG--------------------------------------HRQGPPQDRPEEQPFGQRNYSSEEDGRPHPHHHRH---HGHHRHH---H-HHNQTEGHQGHN-ETGDQDQDKPIDTRPFRFNHFGRKPFGGRPFGRRNHTEGHQGHNETGDHPHRHHNKTGDGDQDRPMFESRPFRFNPFGRKP 309 
3-15-1003                                                                                                                                                                                                157 
3-15-4021                                                                                                                                                                                                131 
3-15-1002                                                                                                                                                                                                120 
3-15-1004                                                                                                                                                                                                131 
3-15-4008                                                                                                                                                                                                62  
3-24-4003 G--------------------------------------HGRGHHGHRQGPPQDRPEEQPFGQRNESSDEDGRPHPRHHGR-----HH-----QH-HH--------------------------------------------RNHTEGHQGHNETGDHPHRHHNKTGDGDQDRPMFEMRPFRFNPFGRKP 237 
3-24-4004 G--------------------------------------HGRGHHGHRQGPPQDRPEEQPFGQRNESSDEDGRPHPRHHGR-----HH-----QH-HH--------------------------------------------RNHTEGHQGHNETGDHPHRHHNKTGDGDQDRPMFEMRPFRFNPFGRKP 237 
3-24-1006 G--------------------------------------HGRGHHGHRQGPPQDRPEEQPFGQRNESSDEDGRPHPRHHGR-----HH-----QH-HH--------------------------------------------RNHTEGHQGHNETGDHPHRHHNKTGGGDQDRPMFEMRPFRFNPFGRKP 237 
3-24-4006 G--------------------------------------HGRGHHGHRQGPPQDRPEEQPFGQRNESSDEDGRPHPRHHGR-----HH-----QH-HH--------------------------------------------RNHTEGHQGHNETGDHPHRHHNKTGDGDQDRPMLEMRPFRFNPFGRKP 237 
3-24-4015 G--------------------------------------HGRGHHGHRQGPPQDRPEEQPFGQRNESSDEDGRPHPRHHGR-----HH-----QH-HH--------------------------------------------RNHTEGHQGHNETGDHPHRHHNKTGDGDQDRPMFEMRPFRFNPFGRKP 237 
3-24-4021 G--------------------------------------HGRGHHGHRQGPPQDRPEEQPFGQRNESSDEDGRPHPRHHGR-----HH-----QH-HH--------------------------------------------RNHTEGHQGHNETGDHPHRHHNKTGDGDQDRPMFEMRPFRFNPFGRKP 237 
3-24-4024 G--------------------------------------HGRGHHGHRQGPPQDRPEEQPFGQRNESSDEDGRPHPRHHGR-----HH-----QH-HH--------------------------------------------RNHTEGHQGHNETGDHPHRHHNKTGDGDQDRPMFEMRPFRFNPFGRKP 237 
3-24-4023 G--------------------------------------HGRGHHGHRQGPPQDRPEEQPFGQRNESSDEDGRPHPRHHGR-----HH-----QH-HH--------------------------------------------RNHTEGHQGHNETGDHPHRHHNKTGDGDQDRPMFEMRPFRFNPFGRKP 237 
3-24-4001 GPRRGHHG--------------------------------------HRQGPPQDRPEEQPFGQRNESSDEDGRPHPRHHGR-----HH-----QH-HH--------------------------------------------RNHTEGHQGHNETGDHPHRHHNKTGDGDQDRPMFEMRPFRFNPFGRKP 236 
3-24-4019 GPRRGHHG--------------------------------------HRQGPPQDRPEEQPFGQRNESSDEDGRPHPRHHGR-----HH-----QH-HH--------------------------------------------RNHTEGHQGHNETGDHPHRHHNKTGDGDQDRPMFEMRPFRFNPFGRKP 236 
3-24-1003 GPGRRHHD---------------------------------------------------------------------------------------------------------------------------RKPFGDRPFGRRNHTEGHQGHNETGDHPHRHHNKTRDGDQDRPMFEMRPFRFNPFGRKP 206 
3-24-4017 GPGRRHHG--------------------------------------HRQRHPQDRPEEQPFGQRNERNEEDGRPHPHHHRH---HGHH----HR--HHNHTEGHQGHN-EMGDQDQDKLHDTRPFRYNHFGRKPFGDRPFGRRNHTEGHQGHNETGDHPHRHHNKTRDGDQDRPMFEMRPFRFNPFGRKP 306 
3-24-4016 GPGRRHHG--------------------------------------HRQGHPQDQAEEQPFGQRNESSEEDGRPHPHHHRH---HGHP----HR--HHNHTEGHQGHN-ETGDQDQDKLNDTRPFRYNHFGRKPFGDRPFGRRNHTEGHQGHNETGDHPHRHHNKTRDGDQDRPMFEMRPFRFNPFGRKP 306 
3-24-4011 GPRRGHHG--------------------------------------HRQGPPQDRPEEQPFGQRNYRSEEDGRPYPYHHCH---HGHHRHH---H-HHNQTEGHQGHN-ETGDQDQDKPIDTRPFRFNHFGRKPFGGRPFGRRNHTEGHQGHNETGDHPHRHHNKTGDGDQDRPMFESRPFRFNPFGRKP 309 
3-24-4005 GPRRGHHG--------------------------------------HRQGPPQDRPEEQPFGQRNYSSEEDGRPHPHHHRH---HGHHRHH---H-HHNQTEGHQGHN-ETGDQDQDKPIDTRPFRFNHFGRKPFGGRPFGRRNHTEGHRGHNETGDHPHRHHNKTGDGDQDRPMFESRPFRFNPFGRKP 309 
3-24-4018 GPRRGHHG--------------------------------------HRQGPPQDRPEEQPFGQRNYSSEEDGRPHPHHHRH---HGHHRHH---H-HHNQTEGHQGHN-ETGDQDQDKPIDTRPFRFNHFGRKPFGGRPFGRRNHTEGHRGHNETGDHPHRHHNKTGDGDQDRPMFESRPFRFNPFGRKP 309 
3-24-4022                                                                                                                                                                                                157 
6-2415    GPGRRHHG---------------------------------------------------------------------------------------------------------------------------RKPFGDRPFGRRNHTEGHQGHNETGDHPHRHHNKTRDGDQDRPMFEMRPFRFNPFGRKP 206 
6-2426    GPGRRHHG---------------------------------------------------------------------------------------------------------------------------RKPFGDRPFGRRNHTEGHQGHNETGDHPHRHHNKTRDGDQDRPMFEMRPFRFNPFGRKP 206 
6-2446    GPGRRHHG---------------------------------------------------------------------------------------------------------------------------RKPFGDRPFGRRNHTEGHQGHNETGDHPHRHHNKTRDGDQDRPMFEMRPFRFNPFGRKP 206 
6-2401    G--------------------------------------HGRGHHGHRQGPPQDRPEEQPFGQRNESSEEDGRPHPRHHGR-----HH-----QH-HH--------------------------------------------RNHTEGHQGHNETGDHPHRHHNKTGDGDQDRPMFEMRPFRFNPFGRKP 237 
6-2402    G--------------------------------------HGRGHHGHRQGPPQDRPEEQPFGQRNESSDEDGRPHPRHHGR-----HH-----QH-HH--------------------------------------------RNHTEGHQGHNETGDHPHRHHNKTGDGDQDRPMFEMRPFRFNPFGRKP 237 
6-2404    G--------------------------------------HGRGHHGHRQGPPQDRPEEQPFGQRNESSDEDGRPHPRHHGR-----HH-----QH-HH--------------------------------------------RNHTEGHQGHNETGDHPHRHHNKTGDGDQDRPMFEMRPFRFNPFGRKP 237 
6-2407    G--------------------------------------HGRGHHGHRQGPPQDRPEEQPFGQRNESSEEDGRPHPRHHGR-----HH-----QH-HH--------------------------------------------RNHTEGHQGHNETGDHPHRHHNKTGDGDQDRPMFEMRPFRFNPFGRKP 237 
6-2408    G--------------------------------------HGRGHHGHRQGPPQDRPEEQPFGQRNESSDEDGRPHPRHHGR-----HH-----QH-HH--------------------------------------------RNHTEGHQGHNETGDHPHRHHNKTGDGDQDRPMFEMRPFRFNPFGRKP 237 
6-2409    G--------------------------------------HGRGHHGHRQGPPQDRPEEQPFGQRNESSEEDGRPHPRHHGR-----HH-----QH-HH--------------------------------------------RNHTEGHQGHNETGDHPHRHHNKTGDGDQDRPMFEMRPFRFNPFGRKP 237 
6-2410    G--------------------------------------HGRGHHGHRQGPPQDRPEEQPFGQRNESSDEDGRPHPRHHGR-----HH-----QH-HH--------------------------------------------RNHTEGHQGHNETGDHPHRHHNKTGDGDQDRPMFEMRPFRFNPFGRKP 237 
6-2411    G--------------------------------------HGRGHHGHRQGPPQDRPEEQPFGQRNESSEEDGRPHPRHHGR-----HH-----QH-HH--------------------------------------------RNHTEGHQGHNETGDHPHRHHNKTGDGDQDRPMFEMRPFRFNPFGRKP 237 
6-2412    G--------------------------------------HGRGHHGHRQGPPQDRPEEQPFGQRNESSDEDGRPHPRHHGR-----HH-----QH-HH--------------------------------------------RNHTEGHQGHNGTGDHPHRHHNKTGDGDQDRPMFEMRPFRFNPFGRKP 237 
6-2413    G--------------------------------------HGRGHHGHRQGPPQDRPEEQPFGQRNESSEEDGRPHPRHHGR-----HH-----QH-HH--------------------------------------------RNHTEGHQGHNETGDHPHRHHNKTGDGDQDRPMFEMRPFRFNPFGRKP 237 
6-2414    G--------------------------------------HGRGHHGHRQGPPQDRPEEQPFGQRNESSDEDGRPHPRHHGR-----HH-----QH-HH--------------------------------------------RNHTEGHQGHNETGDHPHRHHNKTGDGDQDRPMFEMRPFRFNPFGRKP 237 
6-2416    G--------------------------------------HGRGHHGHRQGPPQDRPEEQPFGQRNESSEEDGRPHPRHHGR-----HH-----QH-HH--------------------------------------------RNHTEGHQGHNETGDHPHRHHNKTGDGDQDRPMFEMRPFRFNPFGRKP 237 
6-2417    G--------------------------------------HGRGHHGHRQGPPQDRPEEQPFGQRNESSDEDGRPHPRHHGR-----HH-----QH-HH--------------------------------------------RNHTEGHQGHNETGDHPHRHHNKTGDGDQDRPMFEMRPFRFNPFGRKP 237 
6-2420    G--------------------------------------HGRGHHGHRQGPPQDRPEEQPFGQRNESSEEDGRPHPRHHGR-----HH-----QH-HH--------------------------------------------RNHTEGHQGHNETGDHPHRHHNKTGDGDQDRPMFEMRPFRFNPFGRKP 237 
6-2421    G--------------------------------------HGRGHHGHRQGPPQDRPEEQPFGQRNESSEEDGRPHPRHHGR-----HH-----QH-HH--------------------------------------------RNHTEGHQGHNETGDHPHRHHNKTGDGDQDRPMFEMRPFRFNPFGRKP 237 
6-2422    G--------------------------------------HGRGHHGHRQGPPQDRPEEQPFGQRNESSDEDGRPHPRHHGR-----HH-----QH-HH--------------------------------------------RNHTEGHQGHNETGDHPHRHHNKTGDGDQDRPMFEMRPFRFNPFGRKP 237 
6-2424    G--------------------------------------HGRGHHGHRQGPPQDRPEEQPFGQRNESSEEDGRPHPRHHGR-----HH-----QH-HH--------------------------------------------RNHTEGHQGHNETGDHPHRHHNKTGDGDQDRPMFEMRPFRFNPFGRKP 237 
6-2425    G--------------------------------------HGRGHHGHRQGPPQDRPEEQPFGQRNESSDEDGRPHPRHHGR-----HH-----QH-HH--------------------------------------------RNHTEGHQGHNETGDHPHRHHNKTGDGDQDRPMFEMRPFRFNPFGRKP 237 
6-2427    G--------------------------------------HGRGHHGHRQGPPQDRPEEQPFGQRNESSEEDGRPHPRHHGR-----HH-----QH-HH--------------------------------------------RNHTEGHQGHNETGDHPHRHHNKTGDGDQDRPMFEMRPFRFNPFGRKP 237 
6-2428    G--------------------------------------HGRGHHGHRQGPPQDRPEEQPFGQRNESSDEDGRPHPRHHGR-----HH-----QH-HH--------------------------------------------RNHTEGHQGHNETGDHPHRHHNKTGDGDQDRPMFEMRPFRFNPFGRKP 237 
6-2430    G--------------------------------------HGRGHHGHRQGPPQDRPEEQPFGQRNESSDEDGRPHPRHHGR-----HH-----QH-HH--------------------------------------------RNHTEGHQGHNETGDHPHRHHNKTGDGDQDRPMFEMRPFRFNPFGRKP 237 
6-2431    G--------------------------------------HGRGHHGHRQGPPQDRPEEQPFGQRNESSEEDGRPHPRHHGR-----HH-----QH-HH--------------------------------------------RNHTEGHQGHNETGDHPHRHHNKTGDGDQDRPMFEMRPFRFNPFGRKP 237 
6-2432    G--------------------------------------HGRGHHGHRQGPPQDRPEEQPFGQRNESSEEDGRPHPRHHGR-----HH-----QH-HH--------------------------------------------RNHTEGHQGHNETGDHPHRHHNKTGDGDQDRPMFEMRPFRFNPFGRKP 237 
6-2433    G--------------------------------------HGRGHHGHRQGPPQDRPEEQPFGQRNESSEEDGRPHPRHHGR-----HH-----QH-HH--------------------------------------------RNHTEGHQGHNETGDHPHRHHNKTGDGDQDRPMFEMRPFRFNPFGRKP 237 
6-2434    G--------------------------------------HGRGHHGHRQGPPQDRPEEQPFGQRNESSEEDGRPRPRHHGR-----HH-----QH-HH--------------------------------------------RNHTEGHQGHNETGDHPHRHHNKTGDGDQDRPMFEMRPFRFNPFGRKP 237 
6-2439    G--------------------------------------HGRGHHGHRQGPPQDRPEEQPFGQRNESSEEDGRPHPRHHGR-----HH-----QH-HH--------------------------------------------RNHTEGHQGHNETGDHPHRHHNKTGDGDQDRPMFEMRPFRFNPFGRKP 237 
6-2440    G--------------------------------------HGRGHHGHRQGPPQDRPEEQPFGQRNESSDEDGRPHPRHHGR-----HH-----QH-HH--------------------------------------------RNHTEGHQGHNETGDHPHRHHNKTGDGDQDRPMFEMRPFRFNPFGRKP 237 
6-2441    G--------------------------------------HGRGHHGHRQGPPQDRPEEQPFGQRNESSDEDGRPHPRHHGR-----HL-----QH-HH--------------------------------------------RNHTEGHQGHNETGDHPHRHHNKTGDGDQDRPMFEMRPFRFNPFGRKP 237 
6-2435    G--------------------------------------HGRGHHGHRQGPPQDRPEEQPFGQRNESSEEDGRPHPRHHGR-----HH-----QH-HH--------------------------------------------RNHTEGHQGHNETGDHPHRHHDKTGDGDQDRPMFEMRPFRFNPFGRKP 237 
6-2447    G--------------------------------------HGRGHHGHRQGPPQDRPEEQPFGQRNESSEEDGRPHPRHHGR-----HH-----QH-HH--------------------------------------------RNHTEGHQGHNETGDHPHRHHNKTGDGDQDRPMFEMRPFRFNPFGRKP 237 
6-2448    G--------------------------------------HGRGHHGHRQGPPQDRPEEQPFGQRNESSEEDGRPHPRHHGR-----HH-----QH-HH--------------------------------------------RNHTEGHQGHNETGDHPHRHHNKTGDGDQDRPMFEMRPFRFNPFGRKP 237 
6-2436    G--------------------------------------HGRGHHGHRQGPPQDRPEEQPFGQRNESSDEDGRPHPRHHGR-----HH-----RH-HH--------------------------------------------RNHTEGHQGHNETGDHPHRHHNKTGDGDQDRPMFEMRPFRFNPFGRKP 237 
6-2450    G--------------------------------------HGRGHHGHRQGPPQDRPEEQPFGQRNESSEEDGRPHPRHHGR-----HH-----RH-HH--------------------------------------------RNHTEGHQGHNETGDHPHRHHNKTGDGDQDRPMFEMRPFRFNPFGRKP 237 
6-2429    GPGRRHHG--------------------------------------HRQGHPQDQAEEQPFGQRNESSEEDGRPHPHHHRH---HGHH----HR--HHNHTEGHQGHN-ETGDQDQDKLHDTRPFRYNHFGRKPFGDRPFGRRNHTEGHQGHNETGDHPHRHHNKTRDGDQDRPMFEMRPFRFNPFGRKP 282 
6-2438    G--------------------------------------HGRRHHGHRQGPPQDRPEEQPFGQRNERNEEDGRPHPHHHGH---HGHQGHH-HR--HHNQTEGHQGHN-ETGDQDLDKPNDTRPFRFNHFG----------RRNHTEGHQGHNETGDHPHRHHNKTGDGDQDRPMFEMRPFWVNPFGRKP 275 
6-2423    G--------------------------------------HGRRHHGHRQGPPQDRPEEQPFGQRNERNEEDGRPHPHHHGH---HGHQGHH-HR--HHNQTEGHQGHN-ETGDQDQDKPNDTRPFRFNHFG----------RRNHTEGHQGHNETGDHPHRHHNKTGDGDQDRPMFEMRPFWVNPFGRKP 275 
6-2403    G--------------------------------------HGRGHHGHRQGPPQD                                                                                                                                         156 
6-2444    G--------------------------------------H                                                                                                                                                       142 
6-2449                                                                                                                                                                                                   3   
2-1503    G--------------------------------------H                                                                                                                                                       142 
2-1509    G--------------------------------------H                                                                                                                                                       142 
2-1513    G--------------------------------------H                                                                                                                                                       142 
2-1523    G--------------------------------------H                                                                                                                                                       142 
2-1524    G--------------------------------------H                                                                                                                                                       142 
2-1531    G--------------------------------------H                                                                                                                                                       142 
2-1533    G--------------------------------------H                                                                                                                                                       142 
2-1536    G--------------------------------------H                                                                                                                                                       142 
2-1502    G--------------------------------------HGRGHHGHRQGPPQDRPEEQPFGQRNESSDEDGRPHPRHHGR-----HH-----QH-HH--------------------------------------------RNHTEGHQGHNETGDHPHRHHNKTGDGDQDRPMFEMRPFRFNPFGRKP 237 
2-1518    G--------------------------------------HGRGHHGHRQGPPQDRPEEQPFGQRNESSEEDGRPHPRHHGR-----HH-----QH-HH--------------------------------------------RNHTEGHQGHNETGDHPHRHHNKTGDGDQDRPMFEMRPFRFNPFGRKP 237 
2-1519    G--------------------------------------HGRGHHGHRQGPPQDRPEEQPFGQRNESSEEDGRPHPRHHGR-----HH-----QH-HH--------------------------------------------RNHTEGHQGHNETGDHPHRHHNKTGDGDQDRPMFEMRPFRFNPFGRKP 237 
2-1511    G--------------------------------------HGRGHHGHRQGPPQDRPEEQPFGQRNESSEEDGRPHPRHHGR-----HH-----QH-HH--------------------------------------------RNHTEGRQGHNETGDHPHRHHNKTGDGDQDRPMFEMRPFRFNPFGRKP 237 
2-1546    G--------------------------------------HGRGHHGHRQGPPQDRPEEQPFGQRNESSEEDGRPHPRHHGR-----HH-----QH-HH--------------------------------------------RNHTEGHQGHNETGDHPHRHHNKTGDGDQDRPMFEMRPFRFNPFGRKP 237 
2-1548    G--------------------------------------YGRGHHGHRQGPPQDRPEEQPFGQRNESSEEDGRPHPRHHGR-----HH-----QH-HH--------------------------------------------RNHTEGHQGHNETGDHPHRHHNKTGDGDQDRPMFEMRPFRFNPFGRKP 237 
2-1540                                                                                                                                                                                                   132 
2-2423    G--------------------------------------H                                                                                                                                                       142 
2-2436    G--------------------------------------H                                                                                                                                                       142 
2-2405    G--------------------------------------HGRGHHGHRQGPPQDRPEEQPFGQRNESSDEDGRPHPRHHGR-----HH-----QH-HH--------------------------------------------RNHTEGHQGHNETGDHPHRHHNKTGDGDQDRPMFEMRPFRFNPFGRKP 237 
2-2403    G--------------------------------------HGRGHHGHRQGPPQDRPEEQPFGQRNESSDEDGRPHPRHHGR-----HH-----QH-HH--------------------------------------------RNHTEGHQGHNETGDHPHRHHNKTGDGDQDRPMFEMRPFRFNPFGRKP 237 
2-2448    G--------------------------------------HGRGHHGHRQGPPQDRPEEQPFGQRNESSDEDGRPHPRHHGR-----HH-----QH-HH--------------------------------------------RNHTEGHQGHNETGDHPHRHHNKTGDGDQDRPMFEMRPFRFNPFGRKP 237 
2-2404    G--------------------------------------HGRGHHGHRQGPPQDRPEEQPFGQRNESSDEDGRPHPRHHGR-----HH-----QH-HH--------------------------------------------RNHTEGHQGHNETGDHPHRHHNKTGDGDQDRPMFEMRPFRFNPFGRKP 237 
2-2406    G--------------------------------------HGRGHHGHRQGPPQDRPEEQPFGQRNESSDEDGRPHPRHHGR-----HH-----QH-HH--------------------------------------------RNHTEGHQGHNETGDHPHRHHNKTGDGDQDRPMFEMRPFRFNPFGRKP 237 
2-2409    G--------------------------------------HGRGHHGHRQGPPQDRPEEQPFGQRNESSDEDGRPHPRHHGR-----HH-----QH-HH--------------------------------------------RNHTEGHQGHNETGDHPHRHHNKTGDGDQDRPMFEMRPFRFNPFGRKP 237 
2-2410    G--------------------------------------HGRGHHGHRQGPPQDRPEEQPFGQRNESSDEDGRPHPRHHGR-----HH-----QH-HH--------------------------------------------RNHTEGHQGHNETGDHPHRHHNKTGDGDQDRPMFEMRPFRFNPFGRKP 237 
2-2411    G--------------------------------------HGRGHHGHRQGPPQDRPEEQPFGQRNESSDEDGRPHPRHHGR-----HH-----QH-HH--------------------------------------------RNHTEGHQGHNETGDHPHRHHNKTGDGDQDRPMFEMRPFRFNPFGRKP 237 
2-2412    G--------------------------------------HGRGHHGHRQGPPQDRPEEQPFGQRNESSDEDGRPHPRHHGR-----HH-----QH-HH--------------------------------------------RNHTEGHQGHNETGDHPHRHHNKTGDGDQDRPMFEMRPFRFNPFGRKP 237 
2-2413    G--------------------------------------HGRGHHGHRQGPPQDRPEEQPFGQRNESSDEDGRPHPRHHGR-----HH-----QH-HH--------------------------------------------RNHTEGHQGHNETGDHPHRHHNKTGDGDQDRPMFEMRPFRFNPFGRKP 237 
2-2415    G--------------------------------------HGRGHHGHRQGPPQDRPEEQPFGQRNESSDEDGRPHPRHHGR-----HH-----QH-HH--------------------------------------------RNHTEGHQGHNETGDHPHRHHNKTGDGDQDRPMFEMRPFRFNPFGRKP 237 
2-2416    G--------------------------------------HGRGHHGHRQGPPQDRPEEQPFGQRNESSDEDGRPHPRHHGR-----HH-----QH-HH--------------------------------------------RNHTEGHQGHNETGDHPHRHHNKTGDGDQDRPMFEMRPFRFNPFGRKP 237 
2-2417    G--------------------------------------HGRGHHGHRQGPPQDRPEEQPFGQRNESSDEDGRPHPRHHGR-----HH-----QH-HH--------------------------------------------RNHTEGHQGHNETGDHPHRHHNKTGDGDQDRPMFEMRPFRFNPFGRKP 237 
2-2418    G--------------------------------------HGRGHHGHRQGPPQDRPEEQPFGQRNESSDEDGRPHPRHHGR-----HH-----QH-HH--------------------------------------------RNHTEGHQGHNETGDHPHRHHNKTGDGDQDRPMFEMRPFRFNPFGRKP 237 
2-2419    G--------------------------------------HGRGHHGHRQGPPQDRPEEQPFGQRNESSDEDGRPHPRHHGR-----HH-----QH-HH--------------------------------------------RNHTEGHQGHNETGDHPHRHHNKTGDGDQDRPMFEMRPFRFNPFGRKP 237 
2-2420    G--------------------------------------HGRGHHGHRQGPPQDRPEEQPFGQRNESSDEDGRPHPRHHGR-----HH-----QH-HH--------------------------------------------RNHTEGHQGHNETGDHPHRHHNKTGDGDQDRPMFEMRPFRFNPFGRKP 237 
2-2421    G--------------------------------------HGRGHHGHRQGPPQDRPEEQPFGQRNESSEEDGRPHPRHHGR-----HH-----QH-HH--------------------------------------------RNHTEGHQGHNETGDHPHRHHNKTGDGDQDRPMFEMRPFRFNPFGRKP 237 
2-2422    G--------------------------------------HGRGHHGHRQGPPQDRPEEQPFGQRNESSDEDGRPHPRHHGR-----HH-----QH-HH--------------------------------------------RNHTEGHQGHNETGDHPHRHHNKTGDGDQDRPMFEMRPFRFNPFGRKP 237 
2-2424    G--------------------------------------HGRGHHGHRQGPPQDRPEEQPFGQRNESSDEDGRPHPRHHGR-----HH-----QH-HH--------------------------------------------RNHTEGHQGHNETGDHSHRHHNKTGDGDQDRPMFEMRPFRFNPFGRKP 237 
2-2425    G--------------------------------------HGRGHHGHRQGPPQDRPEEQPFGQRNESSDEDGRPHPRHHGR-----HH-----QH-HH--------------------------------------------RNHTEGHQGHNETGDHPHRHHNKTGDGDQDRPMFEMRPFRFNPFGRKP 237 
2-2426    G--------------------------------------HGRGHHGHRQGPPQDRPEEQPFGQRNESSDEDGRPHPRHHGR-----HH-----QH-HH--------------------------------------------RNHTEGHQGHNETGDHPHRHHNKTGDGDQDRPMFEMRPFRFNPFGRKP 237 
2-2427    G--------------------------------------HGRGHHGHRQGPPQDRPEEQPFGQRNESSDEDGRPHPRHHGR-----HH-----QH-HH--------------------------------------------RNHTEGHQGHNETGDHPHRHHNKTGDGDQDRPMFEMRPFRFNPFGRKP 237 
2-2430    G--------------------------------------HGRGHHGHRQGPPQDRPEEQPFGQRNESSDEDGRPHPRHHGR-----HH-----QH-HH--------------------------------------------RNHTEGHQGHNETGDHPHRHHNKTGDGDQDRPMFEMRPFRFNPFGRKP 237 
2-2431    G--------------------------------------HGRGHHGHRQGPPQDRPEEQPFGQRNESSDEDGRPHPRHHGR-----HH-----QH-HH--------------------------------------------RNHTEGHQGHNETGDHPHRHHNKTGDGDQDRPMFEMRPFRFNPFGRKP 237 
2-2432    G--------------------------------------HGRGHHGHRQGPPQDRPEEQPFGQRNESSDEDGRPHPRHHGR-----HH-----QH-HH--------------------------------------------RNHTEGHQGHNETGDHPHRHHNKTGDGDQDRPMFEMRPFRFNPFGRKP 237 
2-2434    G--------------------------------------HGRGHHGHRQGPPQDRPEEQPFGQRNESSDEDGRPHPRHHGR-----HH-----QH-HH--------------------------------------------RNHTEGHQGHNETGDHPHRHHNKTGDGDQDRPMFEMRPFRFNPFGRKP 237 
2-2437    G--------------------------------------HGRGHHGHRQGPPQDRPEEQPFGQRNESSDEDGRPHPRHHGR-----HH-----QH-HH--------------------------------------------RNHTEGHQGHNETGDHPHRHHNKTGDGDQDRPMFEMRPFRFNPFGRKP 237 
2-2438    G--------------------------------------HGRGHHGHRQGPPQDRPEEQPFGQRNESSDEDGRPHPRHHGR-----HH-----QH-HH--------------------------------------------RNHTEGHQGHNETGDHPHRHHNKTGDGDQDRPMFEMRPFRFNPFGRKP 237 
2-2439    G--------------------------------------HGRGHHGHRQGPPQDRPEEQPFGQRNESSDEDGRPHPRHHGR-----HH-----QH-HH--------------------------------------------RNHTEGHQGHNETGDHPHRHHNKTGDGDQDRPMFEMRPFRFNPFGRKP 237 
2-2440    G--------------------------------------HGRGHHGHRQGPPQDRPEEQPFGQRNESSDEDGRPHPRHHGR-----HH-----QH-HH--------------------------------------------RNHTEGHQGHNETGDHPHRHHNKTGDGDQDRPMFEMRPFRFNPFGRRP 237 
2-2442    G--------------------------------------HGRGHHGHRQGPPQDRPEEQPFGQRNESSDEDGRPHPRHHGR-----HH-----QH-HH--------------------------------------------RNHTEGHQGHNETGDHPHRHHNKTGDGDQDRPMLEMRPFRFNPFGRKP 237 
2-2445    G--------------------------------------HGRGHHGHRQGPPQDRPEEQPFGQRNESSEEDGRPHPRHHGR-----HH-----QH-HH--------------------------------------------RNHTEGHQGHNETGDHPHRHHNKTGDGDQDRPMFEMRPFRFNPFGRKP 237 
2-2446    G--------------------------------------HGRGHHGHRQGPPQDRPEEQPFGQRNESSDEDGRPHPRHHGR-----HH-----QH-HH--------------------------------------------RNHTEGHQGHNETGDHPHRHHNKTGDGDQDRPMFEMRPFRFNPFGRKP 237 
7-1501    G--------------------------------------H                                                                                                                                                       142 
7-1502    G--------------------------------------H                                                                                                                                                       142 
7-1503    G--------------------------------------H                                                                                                                                                       142 
7-1504    G--------------------------------------H                                                                                                                                                       142 
7-1505    G--------------------------------------H                                                                                                                                                       142 
7-1506    G--------------------------------------H                                                                                                                                                       142 
7-1508    G--------------------------------------H                                                                                                                                                       142 
7-1509    G--------------------------------------H                                                                                                                                                       142 
7-1510    G--------------------------------------H                                                                                                                                                       142 
7-1511    G--------------------------------------H                                                                                                                                                       142 
7-1512    G--------------------------------------H                                                                                                                                                       142 
7-1513    G--------------------------------------H                                                                                                                                                       142 
7-1515    G--------------------------------------H                                                                                                                                                       142 
7-1516    G--------------------------------------H                                                                                                                                                       142 
7-1517    G--------------------------------------H                                                                                                                                                       142 
7-1519    G--------------------------------------H                                                                                                                                                       142 
7-1520    G--------------------------------------H                                                                                                                                                       142 
7-1521    G--------------------------------------H                                                                                                                                                       142 
7-1523    G--------------------------------------H                                                                                                                                                       142 
7-1524    G--------------------------------------H                                                                                                                                                       142 
7-1525    G--------------------------------------H                                                                                                                                                       142 
7-1526    G--------------------------------------H                                                                                                                                                       142 
7-1527    G--------------------------------------H                                                                                                                                                       142 
7-1528    G--------------------------------------H                                                                                                                                                       142 
7-1529    G--------------------------------------H                                                                                                                                                       142 
7-1530    G--------------------------------------H                                                                                                                                                       142 
7-1533    G--------------------------------------H                                                                                                                                                       142 
7-1534    G--------------------------------------H                                                                                                                                                       142 
7-1536    G--------------------------------------H                                                                                                                                                       142 
7-1537    G--------------------------------------H                                                                                                                                                       142 
7-1538    G--------------------------------------H                                                                                                                                                       142 
7-1542    G--------------------------------------H                                                                                                                                                       142 
7-1544    G--------------------------------------H                                                                                                                                                       142 
7-1545    G--------------------------------------H                                                                                                                                                       142 
7-1546    G--------------------------------------H                                                                                                                                                       142 
7-1548    G--------------------------------------H                                                                                                                                                       142 
7-1549    G--------------------------------------H                                                                                                                                                       142 
7-1539    G--------------------------------------H                                                                                                                                                       142 
7-1550    G--------------------------------------H                                                                                                                                                       142 
7-1518    G--------------------------------------HGRGHHGHRQGPPQDRPEEQPFGQRNESSEEDGRPHPRHHGR-----HH-----QH-HH--------------------------------------------RNHTEGHQGHNETGDHPHRHHNKTGDGDQDRPMFEMRPFRFNPFGRKP 237 
7-1540                                                                                                                                                                                                   79  
7-1514    G--------------------------------------HGRGHHGHRQGPPQDRPEEQPFGQRNESSEEDGRPHPRHHGR-----HH-----QH-HH--------------------------------------------RNHTEGHQGHNETGDHPHRHHNKTGDGDQDRPMFEMRPFRFNPFGRKP 237 
7-1507    G--------------------------------------HGRGHHGHRQGPPQDRPEEQPFGQRNESSDEDGRPHPRHHGR-----HH-----RH-HH--------------------------------------------RNHTEGHQGHNETGDHPHRHHNKTGDGDQDRPMFEMRPFRFNPFGRKP 237 
7-1522    G--------------------------------------HGRGHHGHRQGPPQDRPEEQPFGQRNESSEEDGRPHPRHHGR-----HH-----QH-HH--------------------------------------------RNHTEGHQGHNETGDHPHRHHNKTGDGDQDRPMFEMRPFRFNPFGRKP 237 
7-1532    G--------------------------------------HGRGHHGHRQGPPQDRPEEQPFGQRNESSDEDGRPHPRHHGR-----HH-----QH-HH--------------------------------------------RNHTEGHQGHNETGDHPHRHHNKTGDGDQDRPMFEMRPFRFNPFGRKP 237 
7-1543    G--------------------------------------HGRGHHGHRQGPPQDRPEEQPFGQRNESSEEDGRPHPRHHGR-----HH-----QH-HH--------------------------------------------RNHTEGHQGHNETGDHPHRHHNKTGDGDQDRPMFEMRPFRFNPFGRKP 237 
7-1547    G--------------------------------------HGRGHHGHRQGPPQDRPEEQPFGQRNESSDEDGRPHPRHHGR-----HH-----RH-HH--------------------------------------------RNHTEGHQGHNETGDHPHRHHNKTGDGDQDRPMFEMRPFRFNPFGRKP 237 
7-2401    G--------------------------------------H                                                                                                                                                       142 
7-2402    G--------------------------------------H                                                                                                                                                       142 
7-2403    G--------------------------------------H                                                                                                                                                       142 
7-2404    G--------------------------------------H                                                                                                                                                       142 
7-2405    G--------------------------------------H                                                                                                                                                       142 
7-2406    G--------------------------------------H                                                                                                                                                       142 
7-2407    G--------------------------------------H                                                                                                                                                       142 
7-2408    G--------------------------------------H                                                                                                                                                       142 
7-2409    G--------------------------------------H                                                                                                                                                       142 
7-2410    G--------------------------------------H                                                                                                                                                       142 
7-2411    G--------------------------------------H                                                                                                                                                       142 
7-2412    G--------------------------------------H                                                                                                                                                       142 
7-2413    G--------------------------------------H                                                                                                                                                       142 
7-2414    G--------------------------------------H                                                                                                                                                       142 
7-2416    G--------------------------------------H                                                                                                                                                       142 
7-2417    G--------------------------------------H                                                                                                                                                       142 
7-2418    G--------------------------------------H                                                                                                                                                       142 
7-2420    G--------------------------------------H                                                                                                                                                       142 
7-2421    G--------------------------------------H                                                                                                                                                       142 
7-2423    G--------------------------------------H                                                                                                                                                       142 
7-2424    G--------------------------------------H                                                                                                                                                       142 
7-2425    G--------------------------------------H                                                                                                                                                       142 
7-2426    G--------------------------------------H                                                                                                                                                       142 
7-2430    G--------------------------------------H                                                                                                                                                       142 
7-2431    G--------------------------------------H                                                                                                                                                       142 
7-2432    G--------------------------------------H                                                                                                                                                       142 
7-2436    G--------------------------------------H                                                                                                                                                       142 
7-2437    G--------------------------------------H                                                                                                                                                       142 
7-2439    G--------------------------------------H                                                                                                                                                       142 
7-2442    G--------------------------------------H                                                                                                                                                       142 
7-2443    G--------------------------------------H                                                                                                                                                       142 
7-2444    G--------------------------------------H                                                                                                                                                       142 
7-2445    G--------------------------------------H                                                                                                                                                       142 
7-2446    G--------------------------------------H                                                                                                                                                       142 
7-2447    G--------------------------------------H                                                                                                                                                       142 
7-2448    G--------------------------------------H                                                                                                                                                       142 
7-2450    G--------------------------------------H                                                                                                                                                       142 
7-2415    G--------------------------------------HGRGHHGHRQGPPQDRPEEQPFGQRNESSDEDGRPHPRHHGR-----HH-----RH-HH--------------------------------------------RNHTEGHQGHNETGDHPHRHHNKTGDGDQDRPMFEMRPFRFNPFGRKP 237 
7-2427    G--------------------------------------HGRGHHGHRQGPPQDRPEEQPFGQRNESSDEDGRPHPRHHGR-----HH-----QH-HH--------------------------------------------RNHTEGHQGHNETGDHPHRHHNKTGDGDQDRPMFEMRPFRFNPFGRKP 237 
7-2428    G--------------------------------------HGRGHHGHRQGPPQDRPEEQPFGQRNESSDEDGRPHPRHHGR-----HH-----QH-HH--------------------------------------------RNHTEGHHGHNETGDHPHRHHNKTGDGDQDRPMFEMRPFRFNPFGRKP 237 
7-2435    G--------------------------------------HGRGHHGHRQGPPQDRPEEQPFGQRNESSEEDGRPHPRHHGR-----HH-----QH-HH--------------------------------------------RNHTEGHQGHNETGDHPHRHHNKTGDGDQDRPMFEMRPFRFNPFGRKP 237 
7-2440    G--------------------------------------HGRGHHGHRQGPPQDRPEEQPFGQRNESSDEDGRPHPRHHGR-----HH-----QH-HH--------------------------------------------RNHTEGHQGHNETGDHPHRHHNKTGDGDQDRPMFEMRPFRFNPFGRKP 237 
7-2441    G--------------------------------------HGRGHHGHRQGPPQDRPEEQPFGQRNESSEEDGRPHPRHHGR-----HH-----QH-HH--------------------------------------------RNHTEGHQGHNETGDHPHRHHNKTGDGDQDRPMFEMRPFRFNPFGRKP 237 
7-2449    G--------------------------------------HGRGHHGHRQGPPQDRPEEQPFGQRNESSEEDGRPHPRHHGR-----HH-----QH-HH--------------------------------------------RNHTEGHQGHNETGDHPHRHHNKTGDGDQDRPMFEMRPFRFNPFGRKP 237 
8-1501    G--------------------------------------H                                                                                                                                                       142 
8-1502    G--------------------------------------H                                                                                                                                                       142 
8-1503    G--------------------------------------H                                                                                                                                                       142 
8-1504    G--------------------------------------H                                                                                                                                                       142 
8-1505    G--------------------------------------H                                                                                                                                                       142 
8-1506    G--------------------------------------H                                                                                                                                                       142 
8-1507    G--------------------------------------H                                                                                                                                                       142 
8-1508    G--------------------------------------H                                                                                                                                                       142 
8-1509    G--------------------------------------H                                                                                                                                                       142 
8-1510    G--------------------------------------H                                                                                                                                                       142 
8-1511    G--------------------------------------H                                                                                                                                                       142 
8-1512    G--------------------------------------H                                                                                                                                                       142 
8-1513    G--------------------------------------H                                                                                                                                                       142 
8-1514    G--------------------------------------H                                                                                                                                                       142 
8-1515    G--------------------------------------H                                                                                                                                                       142 
8-1516    G--------------------------------------H                                                                                                                                                       142 
8-1517    G--------------------------------------H                                                                                                                                                       142 
8-1518    G--------------------------------------H                                                                                                                                                       142 
8-1519    G--------------------------------------H                                                                                                                                                       142 
8-1520    G--------------------------------------H                                                                                                                                                       142 
8-1521    G--------------------------------------H                                                                                                                                                       142 
8-1522    G--------------------------------------H                                                                                                                                                       142 
8-1524    G--------------------------------------H                                                                                                                                                       142 
8-1525    G--------------------------------------H                                                                                                                                                       142 
8-1526    G--------------------------------------H                                                                                                                                                       142 
8-1527    G--------------------------------------H                                                                                                                                                       142 
8-1528    G--------------------------------------H                                                                                                                                                       142 
8-1529    G--------------------------------------H                                                                                                                                                       142 
8-1530    G--------------------------------------H                                                                                                                                                       142 
8-1531    G--------------------------------------H                                                                                                                                                       142 
8-1532    G--------------------------------------H                                                                                                                                                       142 
8-1533    G--------------------------------------H                                                                                                                                                       142 
8-1534    G--------------------------------------H                                                                                                                                                       142 
8-1535    G--------------------------------------H                                                                                                                                                       142 
8-1536    G--------------------------------------H                                                                                                                                                       142 
8-1537    G--------------------------------------H                                                                                                                                                       142 
8-1538    G--------------------------------------H                                                                                                                                                       142 
8-1539    G--------------------------------------H                                                                                                                                                       142 
8-1540    G--------------------------------------H                                                                                                                                                       142 
8-1542    G--------------------------------------H                                                                                                                                                       142 
8-1546    G--------------------------------------H                                                                                                                                                       142 
8-1548    G--------------------------------------H                                                                                                                                                       142 
8-1549    G--------------------------------------H                                                                                                                                                       142 
8-1550    G--------------------------------------H                                                                                                                                                       142 
8-1541    G--------------------------------------HGRGHHGHRQGPPQDRPEEQPFGQRNESSDEDGRPHPRHHGR-----HH-----QH-HH--------------------------------------------RNHTEGHQGHNETGDHPHRHHNKTGDGDQDRPMFEMRPFRFNPFGRKP 237 
8-1543    G--------------------------------------HGRGHHGHRQGPPQDRPEEQPFGQRNESSDEDGRPHPRHHGR-----HH-----QH-HH--------------------------------------------RNHTEGHQGHNETGDHPHRHHNKTGDGDQDRPMFEMRPFRFNPFGRKP 237 
8-2401    G--------------------------------------H                                                                                                                                                       142 
8-2450    G--------------------------------------H                                                                                                                                                       142 
8-2406    G--------------------------------------H                                                                                                                                                       142 
8-2407    G--------------------------------------H                                                                                                                                                       142 
8-2409    G--------------------------------------H                                                                                                                                                       142 
8-2410    G--------------------------------------H                                                                                                                                                       142 
8-2411    G--------------------------------------H                                                                                                                                                       142 
8-2414    G--------------------------------------H                                                                                                                                                       142 
8-2417    G--------------------------------------H                                                                                                                                                       142 
8-2420    G--------------------------------------H                                                                                                                                                       142 
8-2421    G--------------------------------------H                                                                                                                                                       142 
8-2422    G--------------------------------------H                                                                                                                                                       142 
8-2424    G--------------------------------------H                                                                                                                                                       142 
8-2425    G--------------------------------------H                                                                                                                                                       142 
8-2427    G--------------------------------------H                                                                                                                                                       142 
8-2433    G--------------------------------------H                                                                                                                                                       142 
8-2434    G--------------------------------------H                                                                                                                                                       142 
8-2435    G--------------------------------------H                                                                                                                                                       142 
8-2436    G--------------------------------------H                                                                                                                                                       142 
8-2437    G--------------------------------------H                                                                                                                                                       142 
8-2438    G--------------------------------------H                                                                                                                                                       142 
8-2439    G--------------------------------------H                                                                                                                                                       142 
8-2440    G--------------------------------------H                                                                                                                                                       142 
8-2442    G--------------------------------------H                                                                                                                                                       142 
8-2444    G--------------------------------------H                                                                                                                                                       142 
8-2445    G--------------------------------------H                                                                                                                                                       142 
8-2448    G--------------------------------------H                                                                                                                                                       142 
8-2449    G--------------------------------------H                                                                                                                                                       142 
8-2432    GPGRRHHG--------------------------------------HRQGHPQDQAEEQPFGQRNESSEEDGRPHPHHHRH---HGHH----HR--HHNHTEGHQGHN-ETGDQDQDKLHDTRPFRYNHFGRKPFGDRPFGRRNHTEGHQGHNETGDHPHRHHNKTRDGDQDRPMFEMRPFRFNPFGRKP 306 
8-2405    GPGRRHHG--------------------------------------HRQGHPQDQAEEQPFGQRNESSEEDGRPHPHHHRH---HGHH----HR--HHNHTEGHQGHN-ETGDQDQDKLHDTRPFRYNHFGRKPFGDRPFGRRNHTEGHQGHNETGDHPHRHHNKTRDGDQDRPMFEMRPFRFNPFGRKP 306 
8-2443    G--------------------------------------HGRGHHGHRQGPPQDRPEEQPFGQRNESSDEDGRPHPRHHGR-----HH-----QH-HH--------------------------------------------RNHTEGHQGHNETGDHPHRHHNKTGDGDQDRPMFEMRPFRFNPFGRKP 237 
8-2413    G--------------------------------------HGRGHHGHRQGPPQDRPEEQPFGQRNESSDEDGRPHPRHHGR-----HH-----QH-HH--------------------------------------------RNHTEGHQGHNETGDHPHRHHNKTGDGDQDRPMFEMRPFRFNPFGRKP 237 
8-2418    G--------------------------------------HGRGHHGHRQGPPQDRPEEQPFGQRNESSDEDGRPHPRHHGR-----HH-----RH-HH--------------------------------------------RNHTEGHQGHNETGDHPHRHHNKTGDGDQDRPMFEMRPFRFNPFGRKP 237 
8-2419    G--------------------------------------HGRGHHGHRQGPPQDRPEEQPFGQRNESSDEDGRPHPRHHGR-----HH-----QH-HH--------------------------------------------RNHTEGHQGHNETGDHPHRHHNKTGDGDQDRPMFEMRPFRFNPFGRKP 237 
8-2423    G--------------------------------------HGRGHHGHRQGPPQDRPEEQPFGQRNESSDEDGRPHPRHHGR-----HH-----QH-HH--------------------------------------------RNHTEGHQGHNETGDHPHRHHNKTGDGDQDRPMFEMRPFRFNPFGRKP 237 
8-2430    G--------------------------------------HGRGHHGHRQGPPQDQAEEQPFGQRNESSDEDGRPHPRHHGR-----HH-----QH-HH--------------------------------------------RNHTEGHQGHNETGDHPHRHHNKTGDGDQDRPMFEMRPFRFNPFGRKP 237 
8-2446    G--------------------------------------HGRGHHGHRQGPPQDRPEEQPFGQRNESSDEDGRPHPRHHGR-----HH-----QH-HH--------------------------------------------RNHTEGHQGHNETGDHPHRHHNKTGDGDQDRPMFEMRPFRFNPFGRKP 237 
8-2447    G--------------------------------------HGRGHHGHRQGPPQDRPEEQPFGQRNESSDEDGRPHPRHHGR-----HH-----QH-HH--------------------------------------------RNHTEGHQGHNETGDHPHRHHNKTGDGDQDRPMFEMRPFRFNPFGRKP 237 
8-2441    GPGRRHHG---------------------------------------------------------------------------------------------------------------------------RKPFGDRPFGRRNHTEGHQGHNETGDHPHRHHNKTRDGDQDRPMFEMRPFRFNPFGRKP 206 
8-2404    GPRRGHHG--------------------------------------HRQGPPQDRPEEQPFGQRNESSDEDGRPHPRHHGR-----HH-----QH-HH--------------------------------------------RNHTEGHQGHNETGDYPHRHHNKTGDGDQDRPMFEMRPFRFNPFGRKP 237 
8-2415    G--------------------------------------HGRGHHGHRQGPPQTDQRNNRLVSATKAAMRMAVLTLATMAATTSIIIATTQKVTKVTMRQEITPTVITTRPEMEIRTDQCLR                                                                     224 
2-1517    G--------------------------------------H                                                                                                                                                       142 
2-1501    G--------------------------------------H                                                                                                                                                       142 
2-1503    G--------------------------------------H                                                                                                                                                       142 
2-1505    G--------------------------------------H                                                                                                                                                       142 
2-1508    G--------------------------------------H                                                                                                                                                       142 
2-1509    G--------------------------------------H                                                                                                                                                       142 
2-1510    G--------------------------------------H                                                                                                                                                       142 
2-1519    G--------------------------------------H                                                                                                                                                       142 
2-1521    G--------------------------------------H                                                                                                                                                       142 
2-1522    G--------------------------------------H                                                                                                                                                       142 
2-1523    G--------------------------------------H                                                                                                                                                       142 
2-1527    G--------------------------------------H                                                                                                                                                       142 
2-1533    G--------------------------------------H                                                                                                                                                       142 
2-1535    G--------------------------------------H                                                                                                                                                       142 
2-1536    G--------------------------------------H                                                                                                                                                       142 
2-1537    G--------------------------------------H                                                                                                                                                       142 
2-1538    G--------------------------------------H                                                                                                                                                       142 
2-1539    G--------------------------------------H                                                                                                                                                       142 
2-1543    G--------------------------------------H                                                                                                                                                       142 
2-1546    G--------------------------------------H                                                                                                                                                       142 
2-1547    G--------------------------------------H                                                                                                                                                       142 
2-1529    GPRRGHHG--------------------------------------HRQGPPQDRPEEQPFGQRNYSSEEDGRPHPHHHRH---HGHHRHH---H-HHNQTEGHQGHN-ETGDQDQDKPIDTRPFRFNHFGRKPFGGRPFGRRNHTEGHQGHNETGDHPHRHHNKTGDGDQDRPMFESRPFRFNPFGRKP 309 
2-1540    GPGRRHHG--------------------------------------HRQGHPQDQAEEQPFGQRNESSEEDGRPHPHHHRH---HGHH----HR--HHNHTEGHQGHN-ETGDQDQDKLHDTRPFRYNHFGRKPFGDRPFGRRNHTEGHRGHNETGDHPHRHHNKTRDGDQDRPMFEMRPFRFNPFGRKP 306 
2-1506    G--------------------------------------HGRGHHGHRQGPPQDRPEEQPFGQRNESSDEDGRPHPRHHGR-----HH-----QH-HH--------------------------------------------RNHTEGHQGHNETGDHPHRHHNKTGDGDQDRPMFEMRPFRFNPFGRKP 237 
2-1507    G--------------------------------------HGRGHHGHRQGPPQDRPEEQPFGQRNESSEEDGRPHPRHHGR-----HH-----QH-HH--------------------------------------------RNHTEGHQGHNEAGDHPHRHHNKTGDGDQDRPMFEMRPFRFNPFGRKP 237 
2-1511    G--------------------------------------HGRGHHGHRQGPPQDRPEEQPFGQRNESSEEDGRPHPRHHGR-----HH-----QH-HH--------------------------------------------RNHTEGHQGHNETGDHPHRHHNKTGDGDQDRPMFEMRPFRFNPFGRKP 237 
2-1514    G--------------------------------------HGRGHHGHRQGPPQDRPEEQPFGQRNESSDEDGRPHPRHHGR-----HH-----QH-HH--------------------------------------------RNHTEGHQGHNETGDHPHRHHNKTGDGDQDRPMFEMRPFRFNPFGRKP 237 
2-1516    G--------------------------------------HGRGHHGHRQGPPQDRPEEQPFGQRNESSDEDGRPHPRHHGR-----HH-----QH-HH--------------------------------------------RNHTEGHQGHNETGDHPHRHHNKTGDGDQDRPMFEMRPFRFNPFGRKP 237 
2-1528    G--------------------------------------HGRGHHGHRQGPPQDRPEEQPFGQRNESSEEDGRPHPRHHGR-----HH-----QH-HH--------------------------------------------RNHTEGHQGHNETGDHPHRHHNKTGDGDQDRPMFEMRPFRFNPFGRKP 237 
2-1532    G--------------------------------------HGRGHHGHRQGPPQDRPEEQPFGQRNESSDEDGRPHPRHHGR-----HH-----QH-HH--------------------------------------------RNHTEGHQGHNETGDHPHRHHNKTGDGDQDRPMFEMRPFRFNPLGRKP 237 
2-1541    G--------------------------------------HGRGHHGHRQGPPQDRPEEQPFGQRNESSEEDGRPHPRHHGR-----HH-----QH-HH--------------------------------------------RNHTEGHQGHNETGDHPHRHHNKTGDGDQDRPMFEMRPFRFNPFGRKP 237 
2-1542    G--------------------------------------HGRGHHGHRQGPPQDRPEEQPFGQRNESSDEDGRPHPRHHGR-----HH-----QH-HH--------------------------------------------RNHTEGHQGHNETGDHPHRHHNKTGDGDQDRPMFEMRPFRFNPFGRKP 237 
2-2448    G--------------------------------------H                                                                                                                                                       142 
2-2405    G--------------------------------------H                                                                                                                                                       142 
2-2406    G--------------------------------------H                                                                                                                                                       142 
2-2407    G--------------------------------------H                                                                                                                                                       142 
2-2408    G--------------------------------------H                                                                                                                                                       142 
2-2410    G--------------------------------------H                                                                                                                                                       142 
2-2412    G--------------------------------------H                                                                                                                                                       142 
2-2413    G--------------------------------------H                                                                                                                                                       142 
2-2419    G--------------------------------------H                                                                                                                                                       142 
2-2420    G--------------------------------------H                                                                                                                                                       142 
2-2422    G--------------------------------------H                                                                                                                                                       142 
2-2424    G--------------------------------------H                                                                                                                                                       142 
2-2427    G--------------------------------------H                                                                                                                                                       142 
2-2428    G--------------------------------------H                                                                                                                                                       142 
2-2429    G--------------------------------------H                                                                                                                                                       142 
2-2431    G--------------------------------------H                                                                                                                                                       142 
2-2436    G--------------------------------------H                                                                                                                                                       142 
2-2441    G--------------------------------------H                                                                                                                                                       142 
2-2446    G--------------------------------------H                                                                                                                                                       142 
2-2416                                                                                                                                                                                                   121 
2-2411                                                                                                                                                                                                   45  
2-2415                                                                                                                                                                                                   45  
2-2440                                                                                                                                                                                                   45  
2-2423    ---------------------------------------------------------------------------------------------------------------------------------------------------------------------------------------------- 1   
2-2418                                                                                                                                                                                                   131 
2-2421    GPGRRHHG--------------------------------------HRQGHPQDQAEEQPFGQRNESSEEDGRPHPHHHRH---HGHH----HR--HHNHTEGHQGHN-ETGDQDQDKLHDTRPFRYNHFGRKPFGDRPFGRRNHTEGHRGHNETGDHPHRHHNKTRDGDQDRPMFEMRPFRFNPFGRKP 282 
2-2438    GPGRRHHG--------------------------------------HRQGHPQDQAEEQPFGQRNESSEEDGRPHPHHHRH---HGHH----HR--HHNQTEGHQVHN-ETGDQDQDKLHDTRPFRYNHFGRKPFGDRPFGRRNHTEGHQGHNETGDHPHRHHNKTRDGDQDRPMFEMRPFRFNPFGRKP 282 
2-2414    G--------------------------------------HGRGHHGHRQGPPQDRPEEQPFGQRNESSDEDGRPHPRHHGR-----HH-----QH-HH--------------------------------------------RNHTEGHQGHNETGDHPHRHHNKTGDGDQDRPMFEMRPFRFNPFGRKP 237 
2-2403    G--------------------------------------HGRGHHGHRQGPPQDRPEEQPFGQRNESSDEDGRPHPRHHGR-----HH-----QH-HH--------------------------------------------RNHTEGHQGHNETGDHPHRHHNKTGDGDQDRPMFEMRPFRFNPFGRKP 237 
2-2404    G--------------------------------------HGRGHHGHRQGPPQDRPEEQPFGQRNESSDEDGRPHPRHHGR-----HH-----QH-HH--------------------------------------------RNHTEGHQGHNETGDHPHRHHNKTGDGDQDRPMFEMRPFRFNPFGRKP 236 
2-2425    G--------------------------------------HGRGHHGHRQGPPQDRPEEQPFGQRNESSDEDGRPHPRHHGR-----HH-----QH-HH--------------------------------------------RNHTEGHQGHNETGDHPHRHHNKTGDGDQDRPMFEMRPFRFNPFGRKP 236 
2-2426    G--------------------------------------HGRGHHGHRQGPPQDRPEEQPFGQRNESSDEDGRPHPRHHGR-----HH-----QH-HH--------------------------------------------RNHTEGHQGHNETGDHPHRHHNKTGDGDQDRPMFEMRPFRFNPFGRKP 237 
2-2430    G--------------------------------------HGRGHHGHRQGPPQDRPEEQPFGQRNESSDEDGRPHPRHHGR-----HH-----QH-HH--------------------------------------------RNHTEGHQGHNETGDHPHRHHNKTGDGDQDRPMFEMRPFRFNPFGRKP 237 
2-2432    G--------------------------------------HGRGHHGHRQGPPQDRPEEQPFGQRNESSDEDGRPHPRHHGR-----HH-----QH-HH--------------------------------------------RNHTEGHQGHNETGDHSHRHHNKTGDGDQDRPMFEMRPFRFNPFGRKP 236 
2-2437    G--------------------------------------HGRGHHGHRQGPPQDRPEEQPFGQRNESSDEDGRPHPRHHGR-----HH-----QH-HH--------------------------------------------RNHTEGHQGHNETGDHPHRHHNKTGDGDQDRPMFEMRPFRFNPFGRKP 236 
2-2439    G--------------------------------------HGRGHHGHRQGPPQDRPEEQPFGQRNESSDEDGRPHPRHHGR-----HH-----QH-HH--------------------------------------------RNHTEGHQGHNETGDHPHRHHNKTGDGDQDRPMFEMRPFRFNPFGRKP 237 
2-2442    G--------------------------------------HGRGHHGHRQGPPQDRPEEQPFGQRNESSDEDGRPHPRHHGR-----HH-----QH-HH--------------------------------------------RNHTEGHQGHNETGDHSHRHHNKTGDGDQDRPMFEMRPFRFNPFGRKP 237 
2-2443    G--------------------------------------HGRGHHGHRQGPPQDRPEEQPFGQRNESSDEDGRPHPRHHGR-----HH-----QH-HH--------------------------------------------RNHTEGHQGHNETGDHPHRHHNKTGDGDQDRPMFEMRPFRFNPFGRKP 237 
2-2445    G--------------------------------------HGRGHHGHRQGPPQDRPEEQPFGQRNESSDEDGRPHPRHHGR-----HH-----QH-HH--------------------------------------------RNHTEGHQGHNETGDHPHRHHNKTGDGDQDRPMFEMRPFRFNPFGRKP 237 
2-2435    GPGRRHHG---------------------------------------------------------------------------------------------------------------------------RKPFGDRPFGRRNHTEGHQGHNETGDHPHRHHNKTRDGDQDRPMFEMRPFRFNPFGRKP 207 
2-2447    GPGRRHHG---------------------------------------------------------------------------------------------------------------------------RKPFGDRPFGRRNHTEGHQGHNETGDHPHRHHNKTRDGDQDRPMFEMRPFRFNPFGRKP 207 
2-2401    GPGRRHHG---------------------------------------------------------------------------------------------------------------------------RKPFGDRPFGRRNHTEGHQGHNETGDHPHRHHNKTRDGDQDRPMFEMRPFRFNPFGRKP 207 
2-2409    GPGRRHHG--------------------------------------HRQGHPQDQAEEQPFGQRNESSEEDGRPHPHHHRH---HGHH----HR--HHNHTEGHQGHN-ETGDQDQDKLHDTRPFRYNHFGRKPFGDRPFGRRNHTEGHRGHNETGDHPHRHHNKTRDGDQDRPMFEMRPFRFNPFGRKP 306 
2-2444    GPGRRHHG--------------------------------------HRQGHPQDQAEEQPFGQRNESSEEDGRPHPHHHRH---HGHH----HR--HHNHTEGHQGHN-ETGDQDQDKLHDTRPFRYNHFGRKPFGDRPFGRRNHTEGHQGHNETGDHPHRHHNKTRDGDQDRPMFEMRPFRFNPFGRKP 306 
9-1504    G--------------------------------------H                                                                                                                                                       142 
9-1505    G--------------------------------------H                                                                                                                                                       142 
9-1506    G--------------------------------------H                                                                                                                                                       142 
9-1507    G--------------------------------------H                                                                                                                                                       142 
9-1509    G--------------------------------------H                                                                                                                                                       142 
9-1512    G--------------------------------------H                                                                                                                                                       142 
9-1514    G--------------------------------------H                                                                                                                                                       142 
9-1515    G--------------------------------------H                                                                                                                                                       142 
9-1516    G--------------------------------------H                                                                                                                                                       142 
9-1518    G--------------------------------------H                                                                                                                                                       142 
9-1519    G--------------------------------------H                                                                                                                                                       142 
9-1520    G--------------------------------------H                                                                                                                                                       142 
9-1521    G--------------------------------------H                                                                                                                                                       142 
9-1523    G--------------------------------------H                                                                                                                                                       142 
9-1524    G--------------------------------------H                                                                                                                                                       142 
9-1526    G--------------------------------------H                                                                                                                                                       142 
9-1527    G--------------------------------------H                                                                                                                                                       142 
9-1530    G--------------------------------------H                                                                                                                                                       142 
9-1531    G--------------------------------------H                                                                                                                                                       142 
9-1533    G--------------------------------------H                                                                                                                                                       142 
9-1537    G--------------------------------------H                                                                                                                                                       142 
9-1538    G--------------------------------------H                                                                                                                                                       142 
9-1539    G--------------------------------------H                                                                                                                                                       142 
9-1540    G--------------------------------------H                                                                                                                                                       142 
9-1543    G--------------------------------------H                                                                                                                                                       142 
9-1544    G--------------------------------------H                                                                                                                                                       142 
9-1545    G--------------------------------------H                                                                                                                                                       142 
9-1546    G--------------------------------------H                                                                                                                                                       142 
9-1547    G--------------------------------------H                                                                                                                                                       142 
9-1548    G--------------------------------------H                                                                                                                                                       142 
9-1513    GPGRRHHGH--------------------------------------RQGHPQDQAEEQPFGQRNESSEEDGRPHPHHHRH---HGHH----HR--HHNHTEGHQGHN-ETGDQDQDKLHDTRPFRYNHFGRKPFGDRPFGRRNHTEGHRGHNETGDHPHRHHNKTRDGDQDRPMFEMRPFRFNPFGRKP 306 
9-1541    GPGRRHHGH--------------------------------------RQGHPQDQAEEQPFGQRNESSEEDGRPHPHHHRH---HGHH----HR--HHNHTEGHQGHN-ETGDQDQDKLHDTRPFRYNHFGRKPFGDRPFGRRNHTEGHRGHNETGDHPHRHHNKTRDGDQDRPMFEMRPFRFNPFGRKP 306 
9-1542    GPGRRHHGH--------------------------------------RQGHPQDQAEEQPFGQRNESSEEDGRPHPHHHRH---HGHH----HR--HHNHTEGHQGHN-ETGDQDQDKLHDTRPFRYNHFGRKPFGDRPFGRRNHTEGHRGHNETGDHPHRHHNKTRDGDQDRPMFEMRPFRFNPFGRKP 306 
9-1525    HYGHHQGAG--------------------------------------RPFFGNPPPFNPEQEPRNDSSEEDGRHHRHHDRHHAHHGHHGHHEHHHQHHNHTEGHQDH----------------------------------------------------------------DRPMFEMRPFRFNPLGRKP 278 
9-1501    G--------------------------------------HGRGHHGHRQGPPQDRPEEQPFGQRNESSDEDGRPHPRHHGR-----HH-----QH-HR--------------------------------------------RNHTEGHQGHNETGDHPHRHHNKTGDGDQDRPMFEMRPFRFNPFGRKP 237 
9-1502    G--------------------------------------HGRGHHGHRQGPPQDRPEEQPFGQRNESSDEDGRPHPRHHGR-----HH-----QH-HH--------------------------------------------RNHTEGHQGHNETGDHSHRHHNKTGDGDQDRPMFEMRPFRFNPFGRKP 237 
9-1508    G--------------------------------------HGRGHHGHRQGPPQDRPEEQPFGQRNESSDEDGRPHPRHHGR-----HH-----QH-HH--------------------------------------------RNHTEGHQGHNETGDHPHRHHNKTGDGDQDRPMFEMRPFRFNPFGRKP 237 
9-1511    G--------------------------------------HGRGHHGHRQGPPQDRPEEQPFGQRNESSDEDGRPHPRHHGR-----HH-----QH-HH--------------------------------------------RNHTEGHQGHNETGDHPHRHHNKTGDGDQDRPMFEMRPFRFNPFGRKP 237 
9-1517    G--------------------------------------HGRGHHGHRQGPPQDRPEEQPFGQRNESSDEDGRPHPRHHGR-----HH-----QH-HH--------------------------------------------RNHTEGHQGHNETGDHSHRHHNKTGDGDQDRPMFEMRPFRFNPFGRKP 237 
9-1528    G--------------------------------------HGRGHHGHRQGPPQDRPEEQPFGQRNESSDEDGRPHPRHHGR-----HH-----QH-HH--------------------------------------------RNHTEGHQGHNETGDHPHRHHNKTGDGDQDRPMFEMRPFRFNPFGRKP 237 
9-1529    G--------------------------------------HGRGHHGHRQGPPQDRPEEQPFGQRNESSDEDGRPHPRHHGR-----HH-----QH-HH--------------------------------------------RNHTEGHQGHNETGDHPHRHHNKTGDGDQDRPMFEMRPFRFNPFGRKP 237 
9-1534    G--------------------------------------HGRGHHGHRQGPPQDRPEEQPFGQRNESSDEDGRPHPRHHGR-----HH-----QH-HH--------------------------------------------RNHTEGHQGHNETGDHPHRHHNKTGDGDQDRPMFEMRPFRFNPFGRKP 237 
9-1535    G--------------------------------------HGRGHHGHRQGPPQDRPEEQPFGQRNESSDEDGRPHPRHHGR-----HH-----QH-HH--------------------------------------------RNHTEGHQGHNETGDHPHRHHNKTGDGDQDRPMFEMRPFRFNPFGRKP 237 
9-2401    G--------------------------------------H                                                                                                                                                       142 
9-2402    G--------------------------------------H                                                                                                                                                       142 
9-2404    G--------------------------------------H                                                                                                                                                       142 
9-2406    G--------------------------------------H                                                                                                                                                       142 
9-2408    G--------------------------------------H                                                                                                                                                       142 
9-2409    G--------------------------------------H                                                                                                                                                       142 
9-2411    G--------------------------------------H                                                                                                                                                       142 
9-2413    G--------------------------------------H                                                                                                                                                       142 
9-2414    G--------------------------------------H                                                                                                                                                       142 
9-2417    G--------------------------------------H                                                                                                                                                       142 
9-2418    G--------------------------------------H                                                                                                                                                       142 
9-2419    G--------------------------------------H                                                                                                                                                       142 
9-2420    G--------------------------------------H                                                                                                                                                       142 
9-2421    G--------------------------------------H                                                                                                                                                       142 
9-2424    G--------------------------------------H                                                                                                                                                       142 
9-2427    G--------------------------------------H                                                                                                                                                       142 
9-2429    G--------------------------------------H                                                                                                                                                       142 
9-2430    G--------------------------------------H                                                                                                                                                       142 
9-2433    G--------------------------------------H                                                                                                                                                       142 
9-2435    G--------------------------------------H                                                                                                                                                       142 
9-2438    G--------------------------------------H                                                                                                                                                       142 
9-2439    G--------------------------------------H                                                                                                                                                       142 
9-2440    G--------------------------------------H                                                                                                                                                       142 
9-2441    G--------------------------------------H                                                                                                                                                       142 
9-2442    G--------------------------------------H                                                                                                                                                       142 
9-2443    G--------------------------------------H                                                                                                                                                       142 
9-2445    G--------------------------------------H                                                                                                                                                       142 
9-2446    G--------------------------------------H                                                                                                                                                       142 
9-2448    G--------------------------------------H                                                                                                                                                       142 
9-2416    GPGRRHHG--------------------------------------HRQGHPQDQAEEQPFGQRNESSEEDGRPHPHHHRH---HGHH----HR--HHNHTEGHQGHN-EAGDQDQDKLHDTRPFRYNHFGRKPFGDRPFGRRNHTEGHRGHNETGDHPHRHHNKTRDGDQDRPMFEMRPFRFNPFGRKP 306 
9-2423    GPGRRHHG--------------------------------------HRQGHPQDQAEEQPFGQRNESSEEDGRPHPHHHRH---HGHH----HR--HHNHTEGHQGHN-ETGDQDQDKLHDTRPFRYNHFGRKPFGDRPFGRRNHTEGHRGHNETGDHPHRHHNKTRDGDQDRPMFEMRPFRFNPFGRKP 306 
9-2403    G--------------------------------------HGRGHHGHRQGPPQDRPEEQPFGQRNESSDEDGRPHPRHHGR-----HH-----QH-HH--------------------------------------------RNHTEGHQGHNETGDHPHRHHNKTGDGDQDRPMFEMRPFRFNPFGRKP 237 
9-2415    G--------------------------------------HGRGHHGHRQGPPQDRPEEQPFGQRNESSDEDGRPHPRHHGR-----HH-----QH-HH--------------------------------------------RNHTEGHQGHNETGDHPHRHHNKTGDGDQDRPMFEMRPFRFNPFGRKP 237 
9-2422    G--------------------------------------HGRGHHGHRQGPPQDRPEEQPFGQRNESSEEDGRPHPRHHGR-----HH-----QH-HH--------------------------------------------RNHTEGHQGHNETGDHPHRHHNKTGDGDQDRPMFEMRPFRFNPFGRKP 237 
9-2428    G--------------------------------------HGRGHHGHRQGPPQDRPEEQPFGQRNESSDEDGRPHPRHHGR-----HH-----QH-HH--------------------------------------------RNHTEGHQGHNETGDHPHRHHNKTGDGDQDRPMFEMRPFRFNPFGRKP 237 
9-2431    G--------------------------------------HGRGHHGHRQGPPQDRPEEQPFGQRNESSDEDGRPHPRHHGR-----HH-----QH-HH--------------------------------------------RNHTEGHQGHNETGDHPHRHHNKTGDGDQDRPMFEMRPFRFNPFGRKP 237 
9-2432    G--------------------------------------HGRGHHGHRQGPPQDRPEEQPFGQRNESSDEDGRPHPRHHGR-----HH-----QH-HH--------------------------------------------RNHTEGHQGHNETGDHPHRHHNKTGDGDQDRPMFEMRPFRFNPFGRKP 237 
9-2434    G--------------------------------------HWRGHHGHRQGPPQDRPEEQPFGQRNESSDEDGRPHPRHHGR-----HH-----QH-HH--------------------------------------------RNHTEGHQGHNETGDHPHRHHNKTGDGDQDRPMFEMRPFRFNPFGRKP 237 
9-2437    G--------------------------------------HGRGHHGHRQGPPQDRPEEQPFGQRNESSDEDGRPHPRHHGR-----HH-----QH-HH--------------------------------------------RNHTEGHQGHNETGDHPHRHHNKTGDGDQDRPMFEMRPFRFNPFGRKP 237 
9-2444    G--------------------------------------HGRGHHGHRQGPPQDRPEEQPFGQRNESSDEDGRPHPRHHGR-----HH-----QH-HH--------------------------------------------RNHTEGHQGHNETGDHPHRHHNKTGDGDQDRPMFEMRPFRFNPFGRKP 237 
4-1504    GPGRRHHG--------------------------------------HRQGHPQDQAEEQPFGQRNESSEEDGRPHPHHHRH---HGHH----RR--HHNHTEGHQGHN-ETGDQDQDKLHDTRPFRYNHFGRKPFGDRPFGRRNHTEGHRGHNETGDHPHRHHNKTRDGDQDRPMFEMRPFRFNPFGRKP 306 
4-1522    GPGRRHHG--------------------------------------HRQGHPQDQAEEQPFSQRNESSEEDGRPHPHHHRH---HGHH----HR--HHNHTEGHQGHN-ETGDQDQDKLHDTRPFRYNHFGRKPFGDRPFGRRNHTEGHQGHNETGDHPHRHHNKTRDGDQDRPMFEMRPFRFNPFGRKP 282 
4-1507    G--------------------------------------HGRGHHGHRQGPPQDRPEEQPFGQRNESSEEDGRPHPRHHGR-----HH-----QH-HH--------------------------------------------RNHTEGHQGHNETGDHPHRHHNKTGDGDQDRPMFEMRPFRFNPFGRKP 237 
4-1519    G--------------------------------------H                                                                                                                                                       142 
4-1529    G--------------------------------------H                                                                                                                                                       142 
4-1549    G--------------------------------------H                                                                                                                                                       142 
4-1510    G--------------------------------------H                                                                                                                                                       142 
4-1539    G--------------------------------------H                                                                                                                                                       142 
4-2424    GPRRGHHG--------------------------------------HRQGPPQDRPEEQPFGQRNESSDEDGRPHPRHHGR-----HH-----QH-HH--------------------------------------------RNHAEGHQGHNETGDHPHRHHNKTGDGDQDRPMFEMRPFRFNPFGRKP 236 
4-2401    G--------------------------------------HGRGHHGHRQGPPQDRPEEQPFGQRNESSDEDGRPHPRHHGR-----HH-----QH-HH--------------------------------------------RNHTEGHQGHNETGDHPHRHHNKTGDGDQDRPMFEMRPFRFNPFGRKP 237 
4-2405    G--------------------------------------HGRGHHGHRQGPPQDRPEEQPFGQRNESSDEDGRPHPRHHGR-----HH-----QH-HH--------------------------------------------RNHTEGHQGHNETGDHPHRHHNKTGDGDQDRPMFEMRPFRFNPFGRKP 237 
4-2407    G--------------------------------------HGRGHHGHRQGPPQDRPEEQPFGQRNESSDEDGRPHPRHHGR-----HH-----QH-HH--------------------------------------------RNHTEGHQGHNETGDHPHRHHNKTGDGDQDRPMFEMRPFRFNPFGRKP 237 
4-2408    G--------------------------------------HGRGHHGHRQGPPQDRPEEQPFGQRNESSDEDGRPHPRHHGR-----HH-----QH-HH--------------------------------------------RNHTEGHQGHNETGDHPHRHHNKTGDGDQDRPMFEMRPFRFNPFGRKP 237 
4-2411    G--------------------------------------HGRGHHGHRQGPPQDRPEEQPFGQRNESSDEDGRPHPRHHGR-----HH-----QH-HH--------------------------------------------RNHTEGHQGHNETGDHPHRHHNKTGDGDQDRPMFEMRPFRFNPFGRKP 237 
4-2417    G--------------------------------------HGRGHHGHRQGPPQDRPEEQPFGQRNESSDEDGRPHPRHHGR-----HH-----QH-HH--------------------------------------------RNHTEGHQGHNETGDHPHRHHNKTGDGDQDRPMFEMRPFRFNPFGRKP 237 
4-2418    G--------------------------------------HGRGHHGHRQGPPQDRPEEQPFGQRNESSDEDGRPHPRHHGR-----HH-----QH-HH--------------------------------------------RNHTEGHQGHNETGDHPHRHHNKTGDGDQDRPMFEMKPFRFNPFGRKP 237 
4-2419    G--------------------------------------HGRGHHGHRQGPPQDRPEEQPFGQRNESSDEDGRPHPRHHGR-----HH-----QH-HH--------------------------------------------RNHTEGHQGHNETGDHPHRHHNKTGDGDQDRPMFEMRPFRFNPFGRKP 237 
4-2421    G--------------------------------------HGRGHHGHRQGPPQDRPEEQPFGQRNESSDEDGRPHPRHHGR-----HH-----QH-HH--------------------------------------------RNHTEGHQGHNETGDHPHRHHNKTGDGDQDRPMFEMRPFRFNPFGRKP 237 
4-2422    G--------------------------------------HGRGHHGHRQGPPQDRPEEQPFGQRNESSDEDGRPHPRHHGR-----HH-----QH-HH--------------------------------------------RNHTEGHQGHNETGDHPHRHHNKTGDGDQDRPMFEMRPFRFNPFGRKP 237 
4-2426    G--------------------------------------HGRGHHGHRQGPPQDRPEEQPFGQRNESSDEDGRPHPRHHGR-----HH-----QH-HH--------------------------------------------RNHTEGHQGHNETGDHPHRHHNKTGDGDQDRPMFEMRPFRFNPFGRKP 237 
4-2428    G--------------------------------------HGRGHHGHRQGPPQDRPEEQPFGQRNESSDEDGRPHPRHHGR-----HH-----QH-HH--------------------------------------------RNHTEGHQGHNEAGDHPHRHHNKTGDGDQDRPMFEMRPFRFNPFGRKP 237 
4-2429    G--------------------------------------HGRGHHGHRQGPPQDRPEEQPFGQRNESSDEDGRPHPRHHGR-----HH-----QH-HH--------------------------------------------RNHTEGHQGHNETGDHPHRHHNKTGDGDQDRPMFEMRPFRFNPFGRKP 237 
4-2435    G--------------------------------------HGRGHHGHRQGPPQDRPEEQPFGQRNESSDEDGRPHPRHHGR-----HH-----QH-HH--------------------------------------------RNHTEGHQGHNETGDHPHRHHNKTGDGDQDRPMFEMRPFRFNPFGRKP 237 
4-2437    G--------------------------------------HGRGHHGHRQGPPQDRPEEQPFGQRNESSDEDGRPHPRHHGR-----HH-----QH-HH--------------------------------------------RNHTEGHQGHNETGDHPHRHHNKTGDGDQDRPVFEMRPFRFNPFGRKP 237 
4-2444    G--------------------------------------HGRGHHGHRQGPPQDRPEEQPFGQRNESSDEDGRPHPRHHGR-----HH-----QH-HH--------------------------------------------RNHTEGHQGHNETGDHPHRHHNKTGDGDQDRPMFEMRPFRFNPFGRKP 237 
4-2445    G--------------------------------------HGRGHHGHRQGPPQDRPEEQPFGQRNESSDEDGRPHPRHHGR-----HH-----QH-HH--------------------------------------------RNHTEGHQGHNETGDHPHRHHNKTGDGDQDRPMFEMRPFRFNPFGRKP 237 
4-2447    G--------------------------------------HGRGHHGHRQGPPQDRPEEQPFGQRNESSDEDGRPHPRHHGR-----HH-----QH-HH--------------------------------------------RNHTEGHQGHNETGDHPHRHHNKTGDGDQDRPMFEMRPFWFNPFGRKP 237 
4-2448    G--------------------------------------HGRGHHGHRQGPPQDRPEEQPFGQRNESSDEDGRPHPRHHGR-----HH-----QH-HH--------------------------------------------RNHTEGHQGHNETGDHPHRHHNKTGDGDQDRPMFEMRPFRFNPFGRKP 237 
4-2404    GPGRRHHG--------------------------------------HRQGHPQDQAEEQPFSQRNESSEEDGRPHPHHHRH---HGHH----HR--HHNHTEGHQGHN-ETGDQDQDKLHDTRPFRYNHFGRKPFGDRPFGRRNHTEGHQGHNETGDHPHRHHNKTRDGDQDRPMFEMRPFRFNPFGRKP 306 
4-2413    GPGRRHHG--------------------------------------HRQGHPQDQAEEQPFGQRNESSEEDGRPHPHHHRH---HGHH----HR--HHNHTEGHQVHN-ETGDQDQDKLHDTRPFRYNHFGRKPFGDRPFGRRNHTEVHQGHNETGDHPHRHHNKTRDGDQDRPMFEMRPFRFNPFGRKP 306 
4-2450    GPGRRHHG--------------------------------------HRQGHPQDQAEEQPFGQRNESSEEDGRPHPHHHRH---HGHH----HR--HHNHTEGHQVHN-ETGDQDQDKLHDTRPFRYNHFGRKPFGDRPFGRRNHTEVHQGHNETGDHPHRHHNKTRDGDQDRPMFEMRPFRFNPFGRKP 306 
4-2420    GPGRRHHG--------------------------------------HRQGHPQDQAEEQPFGQRNESSEEDGRPHPHHHRH---HGHH----HP--HHNHTEGHQGHN-ETGDQDQDKLHGTRPFRYNHFGRKPFGDRPFGRRNHTEGHQGHNETGDHPHRHHNKTRDGDQDRPMFEMRPFRFNPFGRKP 307 
4-2438    GPGRRHHG--------------------------------------HRQGHPQDQAEEQPFSQRNESSEEDGRPHPHHHRH---HGHH----HR--HHNHTEGHQGHN-ETGDQDQDKLHDTRPFRYNHFGRKPFGDRPFGRRNHTEGHQGHNETGDHPHRHHNKTRDGDQDRPMFEMRPFRFNPFGRKP 307 
4-2430    GPGRRHHG--------------------------------------HRQGHPQDQAEEQPFGQRNESSEEDGRPHPHHHRH---HGHH----HP--HHNHTEGHQGHN-ETGDQDQDKLHGTRPFRYNHFGRKPFGGRPFDRR----------------------------------------------- 260 
4-2439    G--------------------------------------HGRGHHGHRQGPPQDRPEEQPFGQRNESSDEDGRPHPHHHRH---HGHH----HP--HHNHTEGHQGHN-ETGDQDQDKLHGTRPFRYNHFGRKPFGDRPFGRRNHTEGHQGHNETGDHPHRHHNKTRDGDQDRPMFEMRPFRFNPFGRKP 282 
4-2433    G--------------------------------------HGRRHHGHRQGPPQDRPEEQPFGQRNERNEEDGRPHPHHHGH---HGHQGHH-HR--HHNQTEGHQGHN-ETGDQDQDKPNDTRPFRFNHFG----------RRNHTEGHQGHNETGDHPHRHHNKTGDGDQDRPMFEMR----------- 264 
4-2441    G--------------------------------------HGRRHHGHRQGPPQDRPEEQPFGQRNERNEEDGRPHPHHHGH---HGHQGHH-HR--HHNQTEGHQGHN-ETGDQDQDKPNDTRPFRFNHFG----------RRNHTEGHQGHNETGDHPHRHHNKTGDGDQDRPMFEMR----------- 264 
4-2406    GPGHGHYGHHQGAGRPFFGNPPPFNPEQEP---------------------------------RNDSSEEDGRHHRHHDRHHAHHGHHGHHEHHHHH-NHTEGHQ----------------------------------------------------------------DHDRPMFEMRPFRFNPLGRKP 257 
4-2425    GPGRRHHG---------------------------------------------------------------------------------------------------------------------------RKPFGDRPFGRRNHTEGHQGHNETGDHPHRHHNKTRDGDQDRPMFEMRPFRFNPFGRKP 206 
4-2432    GPGRRHHG---------------------------------------------------------------------------------------------------------------------------RKPFGDRPFGRRNHTEGHQGHNETGDHPHRHHNKTRDGDQDRPMFEMRPFRFNPFGRKP 206 
4-2440    GPGRRHHG---------------------------------------------------------------------------------------------------------------------------RKPFGDRPFGRRNHTEGHQGHNETGDHPHRHHNKTRDGDQDRPMFEMRPFRFNPFGRKP 206 
4-2402    ---------------------------------------------------------------------------------------------------------------------------------------------------------------------------------------------- 30  
4-2442    ---------------------------------------------------------------------------------------------------------------------------------------------------------------------------------------------- 1   
4-2443                                                                                                                                                                                                   61  
4-2416                                                                                                                                                                                                   89  
4-2446    G--------------------------------------H                                                                                                                                                       142 
4-2403    G--------------------------------------H                                                                                                                                                       142 
4-2423    G--------------------------------------H                                                                                                                                                       142 
4-2409    G--------------------------------------H                                                                                                                                                       142 
4-2434    G--------------------------------------H                                                                                                                                                       142 
5-1502    GPRRGHHG--------------------------------------HRQGPPQDRPEEQPFGQRNYSSEEDGRPHPHHHRH---HGHHRHH---H-HHNQTEGHQGHN-ETGDQDQDKPIDTRPFRFNHFGRKPFGGRPFGRRNHTEGHQGHNETGDHPHRHHNKTGDGDQDRPMFESRPFRFNPFGRKP 309 
5-1503    GPRRGHHG--------------------------------------HRQGPPQDRPEEQPFGQRNYSSEEDGRPHPHHHRH---HGHHRHH---H-HHNQTEGHQGHN-ETGDQDQDKPIDTRPFRFNHFGRKPFGGRPFGRRNHTEGHQGHNETGDHPHRHHNKTGDGDQDRPMFESRPFRFNPFGRKP 309 
5-1504    GPRRGHHG--------------------------------------HRQGPPQDRPEEQPFGQRNYSSEEDGRPHPHHHRH---HGHHRHH---H-HHNQTEGHQGHN-ETGDQDQDKPIDTRPFRFNHFGRKPFGGRPFGRRNHTEGHQGHNETGDHPHRHHNKTGDGDQDRPMFESRPFRFNPFGRKP 309 
5-1505    GPRRGHHG--------------------------------------HRQGPPQDRPEEQPFGQRNYSSEEDGRPHPHHHRH---HGHHRHH---H-HHNQTEGHQGHN-ETGDQDQDKPIDTRPFRFNHFGRKPFGGRPFGRRNHTEGHQGHNETGDHPHRHHNKTGDGDQDRPMFESRPFRFNPFGRKP 309 
5-1507    GPRRGHHG--------------------------------------HRQGPPQDRPEEQPFGQRNYSSEEDGRPHPHHHRH---HGHHRHH---H-HHNQTEGHQGHN-ETGDQDQDKPIDTRPFRFNHFGRKPLGGRPFGRRNHTEGHQGHNETGDYPHRHHNKTGDGDQDRPMFESRPFRFNPFGRKP 309 
5-1509    GPRRGHHG--------------------------------------HRQGPPQDRPEEQPFGQRNYSSEEDGRPHPHHHRH---HGHHRHH---H-HHNQTEGHQGHN-ETGDQDQDKPIDTRPFRFNHFGRKPFGGRPFGRRNHTEGHQGHNETGDHPHRHHNKTGDGDQDRPMFESRPFRFNPFGRKP 309 
5-1511    GPRRGHHG--------------------------------------HRQGPPQDRPEEQPFGQRNYSSEEDGRPHPHHHRH---HGHHRHH---H-HHNQTEGHQGHN-ETGDQDQDKPIDTRPFRFNHFGRKPCGGRPFGRRNHTEGHQGHNETGDHPHRHHNKTGDGDQDRPMFESRPFRFNPFGRKP 309 
5-1513    GPRRGHHG--------------------------------------HRQGPPQDRPEEQPFGQRNYSSEEDGRPHPHHHRH---HGHHRHH---H-HHNQTEGHQGHN-ETGDQDQDKPIDTRPFRFNHFGRKPFGGRPFGRRNHTEGHQGHNETGDHPHRHHNKTGDGDQDRPMFESRPFRFNPFGRKP 309 
5-1514    GPRRGHHG--------------------------------------HRQGPPQDRPEEQPFGQRNYGGEEDGRPHPHPHRH---HGHHRHH---H-HHNQTEGHQGHN-ETGDQDQDKPIDTRPFRFNHFGRKPFGGRPFGRRNHTEGHQGHNETGDHPHRHHNKTGDGDQDRPMFESRPFRFNPFGRKP 309 
5-1508    GPRRGHHG--------------------------------------HRQGPPQDRPEEQPFGQRNYSSEEDGRPHPHHHRH---HGHHRHH---H-HHNQTEGHQGHN-ETGDQDQDKPIDTRPFRFNHFGRKPFGGRPFGRRNHTEGHQGHNETGDHPHRHHNKTGDGDQDRPMFESRPFRFNPFGRKP 309 
5-1510    GPRRGHHG--------------------------------------HRQGPPQDRPEEQPFGQRNYSSEEDGRPHPHHHRH---HGHHRHH---H-HHNQTEGHQGHN-ETGDQDQDKPIDTRPFRFNHFGRKPFGGRPFGRRNHTEGHQGHNETGDHPHRHHNTTGDEIRTDQCSSRGPSGSTPSVESL 309 
5-2401    GPGRRHHG--------------------------------------HRQGHPQDQAEEQPFGQRNESSEEDGRPHPHHHRH---HGHH-----HR-HHNHTEVHQGHN-ETGDQDQDKLHDTRPFRYNHFGRKPFGDRPFGRRNHTEGHQGHNETGDHPHRHHNKTRDGDQDRPMFEMRPFRFNPFGRKP 306 
5-2402    GPGRRHHG--------------------------------------HRQGHPQDQAEEQPFGQRNESSEEDGRPSPHHHRH---HGHH-----HR-HHNHTEVHQGHN-ETGDQDQDKLHDTRPFRYNHFGRKPFGDRPFGRRNHTEGHQGHNETGDHPHRHHNKTRDGDQDRPMFEMRPFRFNPFGRKP 306 
5-2404    GPGRRHHG--------------------------------------HRQGHPQDQAEEQPFGQRNESSEEDGRPHPHHHRH---HGHH-----HR-HHNHTEGHQGHN-ETGDQDQDKLHDTRPFRYNHFGRKPFGDRPFGRRNHTEGHQGHNETGDNPHRHHNKTRDGDQDRPMFEMRPFRFNPFGRKP 306 
5-2407    GPGRRHHG--------------------------------------HRQGHPQDQAEEQPFGQRNESSEEDGRPHPHHHRH---HGHH-----HR-HHNHTEGHQVHN-ETGDQDQDKLHDTRPFRYNHFGRKPFGDRPFGRRNHTEVHQGHNETGDHPHRHHNKTRDGDQDRPMFEMRPFRFNPFGRKP 306 
5-2409    GPGRRHHG--------------------------------------HRQGHPQDQAEEQLFGQRNESSEEDGRPHPHHHRH---HGHH-----HR-HHNHTEGHQGHN-ETGDQDQDKLHDTRPFRYNHFGRKPFGDRPFGRRNHTEGHQGHNETGDHPHRHHNKTRDGDQDRPMFEMRPFRFNPFGRKP 306 
5-2410    G--------------------------------------HGRGHHGHRQGPPQDRPEEQPFGQRNESSDEDGRPHPRHHGR-----HH-----QH-HH--------------------------------------------RNHTEGHQGHNETGDHPHRHHNKTGDGDQGRPMFEMRPFRFNPFGRKP 237 
5-2411    G--------------------------------------HGRGHHGHRQGPPQDRPEEQPFGQRNESSDEDGRPHPRHHGR-----HH-----QH-HH--------------------------------------------RNHTEGHQGHNETGDHPHRHHNKTGDGDQDRPMFEMRPFRFNPFGRKP 237 
5-2413    G--------------------------------------HGRGHHGHRQGPPQDRPEEQPFGQRNESSDEDGRPHPRHHGR-----HH-----QH-HH--------------------------------------------RNHTEGHQGHNETGDHPHRHHNKTGDGDQDRPMFEMRPFRFNPFGRKP 237 
5-2414    G--------------------------------------HGRGHHGHRQGPPQDRPEEQPFGQRNESSDEDGRPHPRHHGR-----HH-----QH-HH--------------------------------------------RNHTEGHQGHNETGDHPHRHHNKTGDGGQDRPMFEMRPFRFNPFGRKP 237 
5-2415    G--------------------------------------HGRGHHGHRQGPPQDRPEEQPFGQRNESSDEDGRPHPRHHGR-----HH-----QH-HH--------------------------------------------RNHTEGHQGHNETGDHPHRHHNKTGDGDQDRPMFEMRPFRFNPFGRKP 237 
5-2403    G--------------------------------------HGRRHHGHRQGPPQDRPEEQPFGQRNERNEEDGRPHPHHHGH---HGHQGHHH--R-HHNQTEGHQGHN-ETGDQDQDKPNDTRPFRFNHFG----------RRNHTEGHQGHNETGDHPHRHHNKTGDGDQDRPMFEMR----------- 264 
5-2406    GPGRRHHG---------------------------------------------------------------------------------------------------------------------------RKPFGDRPFGRRNHTEGHQGHNETGDHPHRHHNKTRDGDQDRPMFEMRPFRFNPFGRKP 206 
5-2412                                                                                                                                                                                                   132 
5-2408                                                                                                                                                                                                   67  


                  390       400       410       420       430       440       450       460       470       480       490       500       510       520       530       540       550       560       570                  
          ....|....|....|....|....|....|....|....|....|....|....|....|....|....|....|....|....|....|....|....|....|....|....|....|....|....|....|....|....|....|....|....|....|....|....|....|....|....|
1-1515    FGDRPFGRR-------------------------------------------------------------------------------------------------------------------------------------------NGTEEGSPRRDGQRRPYGNRGRWGENESEEKEHP--TMESVT 286 
1-1523    FGDRPFGRR-------------------------------------------------------------------------------------------------------------------------------------------NGTEEGSPRRDGQRRPYGNRGRWGENESEEKEHP--TMESVT 286 
1-1504    FGDRPFGRR-------------------------------------------------------------------------------------------------------------------------------------------NGTEEGSPRRDGQRRPYGNRGRWGENESEEKEHP--TMESVT 286 
1-1533    FGDRPFGRR-------------------------------------------------------------------------------------------------------------------------------------------NGTEEGSPRRDGQRRPYGNRGRWGENESEEKEHP--TMESVT 286 
1-1547                                                                                                                                                                                                   142 
1-1549                                                                                                                                                                                                   142 
1-1505                                                                                                                                                                                                   142 
1-1512                                                                                                                                                                                                   142 
1-1514                                                                                                                                                                                                   142 
1-1528                                                                                                                                                                                                   142 
1-1532                                                                                                                                                                                                   142 
1-1539                                                                                                                                                                                                   142 
1-1536                                                                                                                                                                                                   142 
1-1535                                                                                                                                                                                                   142 
1-1534                                                                                                                                                                                                   142 
1-2402    FGDRPFGRR-------------------------------------------------------------------------------------------------------------------------------------------NGTEEGSPRRDGQRRPYGNRGRWGENESEEKEHP--TMESVT 286 
1-2404    FGDRPFGRR-------------------------------------------------------------------------------------------------------------------------------------------NGTEEGSPRRDGQRRPYGNRGRWGENESEEKEHP--TTESVT 286 
1-2405    FGDRPFGRR-------------------------------------------------------------------------------------------------------------------------------------------NGTEEGSPRRDGQRRPYGNRGRWGENESEEKEHP--TMESVT 286 
1-2406    FGDRPFGRR-------------------------------------------------------------------------------------------------------------------------------------------NGTEEGSPRRDGQRRPYGNRGRWGENESEEKEHP--TMESVT 286 
1-2407    FGDRPFGRR-------------------------------------------------------------------------------------------------------------------------------------------NGTEEGSPRRDGQRRPYGNRGRWGENESEEKEHP--TAESVT 286 
1-2412    FGDRPFGRR-------------------------------------------------------------------------------------------------------------------------------------------NGTEEGSPRRDGQRRPCGNRGRWGENESEEKEHP--TTESVT 286 
1-2413    FGDRPFGRR-------------------------------------------------------------------------------------------------------------------------------------------NGTEEGSPRRDGQRRPYGNRGRWGENESEEKEHP--TMESVT 286 
1-2416    FGDRPFGRR-------------------------------------------------------------------------------------------------------------------------------------------NGTEEGSPRRDGQRRPYGNRGRWGENESEEKEHP--TTESVT 286 
1-2417    FGDRPFGRR-------------------------------------------------------------------------------------------------------------------------------------------NGTEEGSPRRDGQRRPYGNRGRWGENESEEKEHP--TTESVT 286 
1-2418    FGDRPFGRR-------------------------------------------------------------------------------------------------------------------------------------------NGTEEGSPRRDGQRRPYGNRGRWGENESEEKEHP--TMESVT 286 
1-2420    FGDRPFGRR-------------------------------------------------------------------------------------------------------------------------------------------NGTEEGSPRRDGQRRPYGNRGRWGENESEEKEHP--TTESVT 286 
1-2421    FGDRPFGRR-------------------------------------------------------------------------------------------------------------------------------------------NGTEEGSPRRDGQRRPYGNRGRWGENESEEKEHP--TTESVT 286 
1-2422    FGDRPFGRR-------------------------------------------------------------------------------------------------------------------------------------------NGTEEGSPRRDGQRRPYGNRGRWGENESEEKEHP--TMESVT 286 
1-2425    FGDRPFGRR-------------------------------------------------------------------------------------------------------------------------------------------NGTEEGSPRRDGQRRPYGNRGRWGENESEEKEHP--TMESVT 286 
1-2426    FGDRPFGRR-------------------------------------------------------------------------------------------------------------------------------------------NGTEEGSPRRDGQRRPYGNRGRWGENESEEKEHP--TMESVT 286 
1-2427    FGDRPFGRR-------------------------------------------------------------------------------------------------------------------------------------------NGTEEGSPRRDGQRRPYGNRGRWGENESEEKEHP--TMESVT 286 
1-2428    FGDRPFGRR-------------------------------------------------------------------------------------------------------------------------------------------NGTEEGSPRRDGQRRPYGNRGRWGENESEEKEHP--TMESVT 286 
1-2430    FGDRPFGRR-------------------------------------------------------------------------------------------------------------------------------------------NGTEEGSPRRDGQRRPYGNRGRWGENESEEKEHP--TMESVT 286 
1-2431    FGDRPFGRR-------------------------------------------------------------------------------------------------------------------------------------------NGTEEGSPRRDGQRRPYGNRGRWGENESEEKEHP--TTESVT 286 
1-2432    FGDRPFGRR-------------------------------------------------------------------------------------------------------------------------------------------NGTEEGSPRRDGQRRPYGNRGRWGENESEEKEHP--TTESVT 286 
1-2433    FGDRPFGRR-------------------------------------------------------------------------------------------------------------------------------------------NGTEEGSPRRDGQRRPYGNRGRWGENESEEKEHP--TMESVT 286 
1-2434    FGDRPFGRR-------------------------------------------------------------------------------------------------------------------------------------------NGTEEGSPRRDGQRRPYGNRGRWGENESEEKEHP--TTESVT 286 
1-2435    FGDRPFGRR-------------------------------------------------------------------------------------------------------------------------------------------NGTEEGSPRRDGQRRPYGNRGRWGENESEEKEHP--TAESVT 286 
1-2436    FGDRPFGRR-------------------------------------------------------------------------------------------------------------------------------------------NGTEEGSPRRDGQRRPYGNRGRWGENESEEKEHP--TMESVT 286 
1-2437    FGDRPFGRR-------------------------------------------------------------------------------------------------------------------------------------------NGTEEGSPRRDGQRRPYGNRGRWGENESEEKEHP--TMESVT 286 
1-2439    FGDRPFGRR-------------------------------------------------------------------------------------------------------------------------------------------NGTEEGSPRRDGQRRPYGNRGRWGENESEEKEHP--TTESVT 286 
1-2440    FGDRPFGRR-------------------------------------------------------------------------------------------------------------------------------------------NGTEEGSPRRDGQRRPYGNRGRWGENESEEKEHP--TTESVT 286 
1-2441    FGDRPFGRR-------------------------------------------------------------------------------------------------------------------------------------------NGTEEGSPRRDGQRRPYGNRGRWGENESEEKEHP--TMESVT 286 
1-2442    FGDRPFGRR-------------------------------------------------------------------------------------------------------------------------------------------NGTEEGSPRRDGQRRPYGNRGRWGENESEEKEHP--TTESVT 286 
1-2414    FGGRPFDRR-------------------------------------------------------------------------------------------------------------------------------------------NGTEEGSPRRDGHRRPYGNRGRWGENESEEKEHP--TTESVT 355 
1-2424                                                                                                                                                                                                   58  
1-2429                                                                                                                                                                                                   58  
2-1501    FGDRLFGRR-------------------------------------------------------------------------------------------------------------------------------------------NVTEEGSPRRDGHRRPYGNRGRWGENESEEKEHP--TTESVT 358 
2-1502    FGDRLFGRR-------------------------------------------------------------------------------------------------------------------------------------------NGTEEGSPRRDGHRRPYGNRGRWGENESEEKEHP--TTESVT 358 
2-1505    FGDRLFGRR-------------------------------------------------------------------------------------------------------------------------------------------NGTEEGSPRRDGHRRPYGNRGRWGENESEEKEHP--TTESVT 358 
2-1506    FGDRLFGRR-------------------------------------------------------------------------------------------------------------------------------------------NGTEEGSPRRDGHRRPYGNRGRWGENESEEKEHP--TTESVT 358 
2-1507    FGDRLFGRR-------------------------------------------------------------------------------------------------------------------------------------------NGTEEGSPRRDGHRRPYGNRGRWGENESEEKEHP--TTESVT 358 
2-1508    FGDRLFGRR-------------------------------------------------------------------------------------------------------------------------------------------NGTEEGSPRRDGHRRPYGNRGRWGENESEEKEQP--TTESVT 358 
2-1509    FGDRLFGRR-------------------------------------------------------------------------------------------------------------------------------------------NGTEEGSPRRDGHRRPYGNRGRWGENESEEKEHP--TTESVT 358 
2-1510    FGDRLFGRR-------------------------------------------------------------------------------------------------------------------------------------------NGTEEGSPRRDGHRRPYGNRGRWGENESVEKEHP--TTESVT 358 
2-1514    FGDRLFGRR-------------------------------------------------------------------------------------------------------------------------------------------NGTEEGSPRRDGHRRPYGNRGRWGENESEEKEHP--TTESVT 358 
2-1511    FGDRLFGRR-------------------------------------------------------------------------------------------------------------------------------------------NGTEEGSPRRDGHRRPYGNRGRWGENESEEKEHP--TTESVT 358 
2-2401    FGDRPFGRR-------------------------------------------------------------------------------------------------------------------------------------------NGTEEGSPRRDGQRRPYGNRGRWGENESEEKEHP--TTESVT 285 
2-2404    FGDRPFGRR-------------------------------------------------------------------------------------------------------------------------------------------NGTEEGSPRRDGQRRPYGNRGRWGENESEEKEHP--TTESVT 285 
2-2406    FGDRPFGRR-------------------------------------------------------------------------------------------------------------------------------------------NGTEEGSPRRDGQRRPYGNRGRWGENESEEKEYP--TTESVT 285 
2-2407    FGDRPFGRR-------------------------------------------------------------------------------------------------------------------------------------------NGTEEGSPRRDGQRRPYGNRGRWGENESEEKEHP--TTESVT 285 
2-2408    FGDRPFGRR-------------------------------------------------------------------------------------------------------------------------------------------NGTEEGSPRRDGQRRPYGNRGRWGENESEEKEHP--TTESVT 285 
2-2409    FGDRPFGRR-------------------------------------------------------------------------------------------------------------------------------------------NGTEEGSPRRDGQRRPYGNRGRWGENESEEKEHP--TTESVT 285 
2-2411    FGDRPFGRR-------------------------------------------------------------------------------------------------------------------------------------------NGTEEGSPRRDGQRRPYGNRGRWGENESEEKEHP--TTESVT 285 
2-2405    --------------------------------------------------------------------------------------------------------------------------------PFWVNPFGRKPFGDRPFGRRNGTEEGSPRRDGHRHPYGNRGRWGENESEEKEHP--TTESIT 318 
2-2413    --------------------------------------------------------------------------------------------------------------------------------PFWVNPFG                                                       266 
2-2403    FGDRPFGRR-------------------------------------------------------------------------------------------------------------------------------------------NGTEEGSPRRDGQRRPYGNRGRWGENESEEKEHP--TMESVT 256 
2-2415    FGGRPFDRR-------------------------------------------------------------------------------------------------------------------------------------------NGTEEGSPRRDGHRRPYGNRGRWGENESEEKEHP--TTESVT 355 
2-2414    FGGRPFDRR-------------------------------------------------------------------------------------------------------------------------------------------NGTEEGSPRRDGHRRPYGNRGRWSENESEEKEHP--TTESVT 355 
3-15-1006 FGDRLFGRR-------------------------------------------------------------------------------------------------------------------------------------------NGTEEGSPRRDGHRRPYGNRGRWGENESEEKEHP--TTESVT 358 
3-15-4003 FGDRLFGRR-------------------------------------------------------------------------------------------------------------------------------------------NGTEEGSPRRDGHRRPYGNRGRWGENESEEKEHP--TTESVT 358 
3-15-4004 FGDRLFGRR-------------------------------------------------------------------------------------------------------------------------------------------NGTEEGSPRRDGHRRPYGNRGRWGENESEEKEHP--TTESVT 358 
3-15-4005 FGDRLFGRR-------------------------------------------------------------------------------------------------------------------------------------------NGTEEGSPRRDGHRRPYGNRGRWGENESEEKEHP--TTESVT 358 
3-15-4007 FGDRLFGRR-------------------------------------------------------------------------------------------------------------------------------------------NGTEEGSPRRDGHRRPYGNRGRWGENESEEKEHP--TTESVT 358 
3-15-4011 FGDRLFGRR-------------------------------------------------------------------------------------------------------------------------------------------NGTEEGSPRRDGHRRPYGNRGRWGENESEEKEHP--TTESVT 358 
3-15-4013 FGDRLFGRR-------------------------------------------------------------------------------------------------------------------------------------------NGTEEGSPRRDGHRRPYGNRGRWGENESEEKEHP--TTESVT 358 
3-15-4015 FGDRLFGRR-------------------------------------------------------------------------------------------------------------------------------------------NGTEEGSPRRDGHRRPYGNRGRWGENESEEKEHP--TTESVT 358 
3-15-4018 FGGRLFGRR-------------------------------------------------------------------------------------------------------------------------------------------NGTEEGSPRRDGHRRPYGNRGRWGENESEEKEHP--TTESVT 358 
3-15-4019 FGDRLFGRR-------------------------------------------------------------------------------------------------------------------------------------------NGTEEGSPRRDGHRRPYGNRGRWGENESEEKEHP--TTESVT 358 
3-15-4022 FGDRLFGRR-------------------------------------------------------------------------------------------------------------------------------------------NGTEEGSPRRDGHRRPYGNRGRWGENESEEKEHP--TTESVT 358 
3-15-4024 FGDRLFGRR-------------------------------------------------------------------------------------------------------------------------------------------NGTEEGSPRRDGHRRPYGNRGRWGENESEEKEHP--TTESVT 358 
3-15-4017 FGDRLFGRR-------------------------------------------------------------------------------------------------------------------------------------------NGTEEGSPRRDGHRRPYGNRGRWVRMKVRRRSIQRRKA     356 
3-15-1003                                                                                                                                                                                                157 
3-15-4021                                                                                                                                                                                                131 
3-15-1002                                                                                                                                                                                                120 
3-15-1004                                                                                                                                                                                                131 
3-15-4008                                                                                                                                                                                                62  
3-24-4003 FGDRPFGRR-------------------------------------------------------------------------------------------------------------------------------------------NGTEDGSPRRDGQRRPYGNRGRWGENESEEKEHP--TTESVT 286 
3-24-4004 FGDRPFGRR-------------------------------------------------------------------------------------------------------------------------------------------NGTEEGSPRRDGHRRPYGNRGRWGENESEEKERP--TMESVT 286 
3-24-1006 FGDRPFGRR-------------------------------------------------------------------------------------------------------------------------------------------NGTEDGSPRRDGQRRPYGNRGRWGENESEEKEHP--TTESVT 286 
3-24-4006 FGDRPFGRR-------------------------------------------------------------------------------------------------------------------------------------------NGTEEGSPRRDGHRRPYGNRGRWGENESEEKEHP--TMESVT 286 
3-24-4015 FGDRPFGRR-------------------------------------------------------------------------------------------------------------------------------------------NGTEDGSPRRDGQRRPYGNRGRWGENESEEKEHP--TTESVT 286 
3-24-4021 FGDRPFGRR-------------------------------------------------------------------------------------------------------------------------------------------NGTEEGSPRRDGHRRPYGNRGRWGENESEEKEHP--TMESVT 286 
3-24-4024 FGDRPFGRR-------------------------------------------------------------------------------------------------------------------------------------------NGTEEGSPRRDGHRRPYGNRGRWGENESEEKEHP--TMESVT 286 
3-24-4023 FGDRPFGRR-------------------------------------------------------------------------------------------------------------------------------------------NGTEDGSPRRDGQRRPYGNRGRWGENESEEKEHP--TTESVT 286 
3-24-4001 FGGRPFGRR-------------------------------------------------------------------------------------------------------------------------------------------NGTEEGSPRRDGQRRPYGNRGRWGENESEEKEHP--TTESVT 285 
3-24-4019 FGGRPFGRR-------------------------------------------------------------------------------------------------------------------------------------------NGTEEGSPRRDGQRRPYGNRGRWGKNESEEKEHP--TTESVT 285 
3-24-1003 FGDRPFGRR-------------------------------------------------------------------------------------------------------------------------------------------NGTEEGSPRRDGQRRPHGNRGRWGENESEEKEHP--TTESVT 255 
3-24-4017 FGGRPFDRR-------------------------------------------------------------------------------------------------------------------------------------------NGTEEGSPRRDGHRRPYGNRGRWGENESEEKEHP--TTESVT 355 
3-24-4016 FGGLPSTDATEPKKDLPGVMAIVGPMVTEDDGVRMKVRRRSIQRRKA                                                                                                                                                353 
3-24-4011 FGDRLFGRR-------------------------------------------------------------------------------------------------------------------------------------------NGTEEGSPRRDGHRRPYGNRGRRGENESEEKEHP--TTESVT 358 
3-24-4005 FGDRLFGRR-------------------------------------------------------------------------------------------------------------------------------------------NGTEEGSPRRDGHRRPYGNRGRWGENESEEKEHP--TTESVT 358 
3-24-4018 FGDRLFGRR-------------------------------------------------------------------------------------------------------------------------------------------NGTEEGSPRRDGHRRPYGNRGRWGENESEEKEHP--TTESVT 358 
3-24-4022                                                                                                                                                                                                157 
6-2415    FGDRPFGRR-------------------------------------------------------------------------------------------------------------------------------------------NGTEEGSPRRDGQRRPHGNRGRWGENESEEKEHP--TTESIT 255 
6-2426    LGDRPFGRR-------------------------------------------------------------------------------------------------------------------------------------------NGTEEGSPRRDGQRRPHGNRGRWGENESEEKEHP--TTESIT 255 
6-2446    FGDRPFGRR-------------------------------------------------------------------------------------------------------------------------------------------NGTEEGSPRRDGQRRPHGNRGRWGENESEEKERP--TTESIT 255 
6-2401    FGDRPFGRR-------------------------------------------------------------------------------------------------------------------------------------------NGTEEGSPRRDGQRRPYGNRGRWGENESEEKEHP--TTESVT 286 
6-2402    FGDRPFGRR-------------------------------------------------------------------------------------------------------------------------------------------NGTEEGSPRRDGQRRPYGNRGRWGENKSEEKEHP--TMESVT 286 
6-2404    FGDRPFGRR-------------------------------------------------------------------------------------------------------------------------------------------NGTEEGSPRRDGQRRPYGNRGRWGENESEEKEHP--TMESVT 286 
6-2407    FGDRPFGRR-------------------------------------------------------------------------------------------------------------------------------------------NGTEEGSPRRDGQRRPYGNRGRWGENESEEKEHP--TTESVT 286 
6-2408    FGDRPFGRR-------------------------------------------------------------------------------------------------------------------------------------------NGTEEGSPRRDGQRRPYGNRGRWGENESEEKEHP--TMESVT 286 
6-2409    FGDRPFGRR-------------------------------------------------------------------------------------------------------------------------------------------NGTEEGSPRRDGQRRPYGNRGRWGENESEEKEHP--TTESVT 286 
6-2410    FGDRPFGRR-------------------------------------------------------------------------------------------------------------------------------------------NGTEEGSPRRDGQRRPYGNRGRWGENESEEKEHP--TMESVT 286 
6-2411    FGDRPFGRR-------------------------------------------------------------------------------------------------------------------------------------------NGTEEGSPRRDGQRRPYGNRGRWGENESEEKEHP--TTESVT 286 
6-2412    FGDRPFGRR-------------------------------------------------------------------------------------------------------------------------------------------NGTEEGSPRRDGQRRPYGNRGRWGENESEEKEHP--TMESVT 286 
6-2413    FGDRPFGRR-------------------------------------------------------------------------------------------------------------------------------------------NGTEEGSPRRDGQRRPYGNRGRWGENESEEKKHP--TTESVT 286 
6-2414    FGDRPFGRR-------------------------------------------------------------------------------------------------------------------------------------------NGTEEGSPRRDGQRRPYGNRGRWGENESEEKEHP--TMESVT 286 
6-2416    FGDRPFGRR-------------------------------------------------------------------------------------------------------------------------------------------NGTEEGSPRRDGQRRPYGNRGRWGENESEEKEHP--TTESVT 286 
6-2417    FGDRPFGRR-------------------------------------------------------------------------------------------------------------------------------------------NGTEEGSPRRDGQRRPYGNRGRWGENESEEKEHP--TMESVT 286 
6-2420    FGDRPFGRR-------------------------------------------------------------------------------------------------------------------------------------------NGTEEGSPRRDGQRRPYGNRGRWGENESEEKEHP--TTESVT 286 
6-2421    FGDRPFGRR-------------------------------------------------------------------------------------------------------------------------------------------NGTEEGSPRRDGQRRPYGNRGRWGENESEEKEHP--TTESVT 286 
6-2422    FGDRPFGRR-------------------------------------------------------------------------------------------------------------------------------------------NGTEEGSPRRDGQRRPYGNRGRWGENESEEKEHP--TMESVT 286 
6-2424    FGDRPFGRR-------------------------------------------------------------------------------------------------------------------------------------------NGTEEGSPRRDGQRRPYGNRGRWGENESEEKEHP--TTESVT 286 
6-2425    FGDRPFGRR-------------------------------------------------------------------------------------------------------------------------------------------NGTEEGSPRRDGQRRPYGNRGRWGENESEEKEHP--TMESVT 286 
6-2427    FGDRPFGRP-------------------------------------------------------------------------------------------------------------------------------------------NGTEEGSPRRDGQRRPYGNRGRWGENESEEKEHP--TTESVT 286 
6-2428    FGDRPFGRR-------------------------------------------------------------------------------------------------------------------------------------------NGTEEGSPRRDGQRRPYGNRGRWGENESEEKEHP--TMESVT 286 
6-2430    FGDRPFGRR-------------------------------------------------------------------------------------------------------------------------------------------NGTEEGSPRRDGQRRPYGNRGRWGENESEEKEHP--TMESVT 286 
6-2431    FGDRPFGRR-------------------------------------------------------------------------------------------------------------------------------------------NGTEEGSPRRDGQRRPYGNRGRWGENESEEKEHP--TTESVT 286 
6-2432    FGDRPFGRR-------------------------------------------------------------------------------------------------------------------------------------------NGTEEGSPRRDGQRRPYGNRGRWGENESEEKEHP--TTESVT 286 
6-2433    FGDRPFGRR-------------------------------------------------------------------------------------------------------------------------------------------NGTEEGSPRRDGQRRPYGNRGRWGENESEEKEHP--TTESVT 286 
6-2434    FGDRPFGRR-------------------------------------------------------------------------------------------------------------------------------------------NGTEEGSPRRDGQRRPYGNRGRWGENESEEKEHP--TTESVT 286 
6-2439    FGDRPFGRR-------------------------------------------------------------------------------------------------------------------------------------------NGTEEGSPRRDGQRRPYGNRGRWGENESEEKEHP--TTESVT 286 
6-2440    FGDRPFGRR-------------------------------------------------------------------------------------------------------------------------------------------NGTEEGSPRRDGQRRPYGNRGRWGENESEEKEHP--TMESVT 286 
6-2441    FGDRPFGRR-------------------------------------------------------------------------------------------------------------------------------------------NGTEEGSPRRDGQRRPYGNRGRWGENESEEKEHP--TMESVT 286 
6-2435    FGDRPFGRR-------------------------------------------------------------------------------------------------------------------------------------------NGTEEGSPRRDGQRRPYGNRGRWGENESEEKEHP--TTESVT 286 
6-2447    FGDRPFGRR-------------------------------------------------------------------------------------------------------------------------------------------NGTEEGSPRRDGQRRPYGNRGRWGENESEEKEHP--TTESVT 286 
6-2448    FGDRPFGRR-------------------------------------------------------------------------------------------------------------------------------------------NGTEEGSPRRDGQRRPYGNRGRWGENESEEKEHP--TTESVT 286 
6-2436    FGDRPFGRR-------------------------------------------------------------------------------------------------------------------------------------------NGTEEGSPRRDGQRRPYGNRGRWGENESEEKEHP--TMESVT 286 
6-2450    FGDRPFGRR-------------------------------------------------------------------------------------------------------------------------------------------NGTEEGSPRRDGQRRPYGNRGRWGENESEEKEHP--TTESVT 286 
6-2429    FGGRPFDRR-------------------------------------------------------------------------------------------------------------------------------------------NGTEEGSPRRDGHRRPYGNRGRWGENESEEKEHP--TTESVT 331 
6-2438    FGDRPFGRR-------------------------------------------------------------------------------------------------------------------------------------------NGTEEGSPRRDGHRHPYGNRGRWGENESEEKEHP--TTESVT 324 
6-2423    FGDRPFGRR-------------------------------------------------------------------------------------------------------------------------------------------NGTEEGSPRRDGHRHPYGNRGRWGENESEEKEHP--TTESVT 324 
6-2403                                                                                                                                                                                                   156 
6-2444                                                                                                                                                                                                   142 
6-2449                                                                                                                                                                                                   3   
2-1503                                                                                                                                                                                                   142 
2-1509                                                                                                                                                                                                   142 
2-1513                                                                                                                                                                                                   142 
2-1523                                                                                                                                                                                                   142 
2-1524                                                                                                                                                                                                   142 
2-1531                                                                                                                                                                                                   142 
2-1533                                                                                                                                                                                                   142 
2-1536                                                                                                                                                                                                   142 
2-1502    FGDRPLGRR-------------------------------------------------------------------------------------------------------------------------------------------NGTEEGSPRRDGQRRPYGNRGRWGENESEEKEHP--TMESVT 286 
2-1518    FGDRPFGRR-------------------------------------------------------------------------------------------------------------------------------------------NGTEEGSPRRDGQHRPYGNRGRWGENESEEKEHP--TTESVT 286 
2-1519    FGDRPFGRR-------------------------------------------------------------------------------------------------------------------------------------------NGTEEGSPRRDGQRRPYGNRGRWGENESEEKEHP--TTESVT 286 
2-1511    FGDRPFGRR-------------------------------------------------------------------------------------------------------------------------------------------NGTEEGSPRRDGQRRPYGNRGRWGENESEEKEHP--TTESVT 286 
2-1546    FGDRPFGRR-------------------------------------------------------------------------------------------------------------------------------------------NGTEEGSPRRDGQRRPYGNRGRWGENESEEKEHP--TTESVT 286 
2-1548    FGDRPFGRP-------------------------------------------------------------------------------------------------------------------------------------------NGTEEGSPRRDGQRRPYGNRGRWGENESEEKEHP--TTEGVT 286 
2-1540                                                                                                                                                                                                   132 
2-2423                                                                                                                                                                                                   142 
2-2436                                                                                                                                                                                                   142 
2-2405    FGDRPFGRR-------------------------------------------------------------------------------------------------------------------------------------------NGTEEGSPRRDGQRRPYGNRGRWGENESEEKEDP--TMESVT 286 
2-2403    FGDRPFGRR-------------------------------------------------------------------------------------------------------------------------------------------NGTEEGSPRRDGQRRPYGNRGRWGENESEEKEHP--TMESVT 286 
2-2448    FGDRPFGRR-------------------------------------------------------------------------------------------------------------------------------------------NGTEEGSPRRDGQRRPYGNRGRWGENESEEKEHP--TMESVT 286 
2-2404    FGDRPFGRR-------------------------------------------------------------------------------------------------------------------------------------------NGTEEGSPRRDGQRRPYGNRGRWGENESEEKEHP--TMESVT 286 
2-2406    FGDRPFGRR-------------------------------------------------------------------------------------------------------------------------------------------NGTEEGSPRRDGQRRPYGNRGRWGENESEEKEHP--TMESVT 286 
2-2409    FRDRPFGRR-------------------------------------------------------------------------------------------------------------------------------------------NGTEEGSPRRDGQRRPYGNRGRWGENESEEKEHP--TMESVT 286 
2-2410    FGDRPFGRR-------------------------------------------------------------------------------------------------------------------------------------------NGTEEGSPRRDGQRRPYGNRGRWGENESEEKEHP--TMESVT 286 
2-2411    FGDRPFGRR-------------------------------------------------------------------------------------------------------------------------------------------NGTEEGSPRRDGQRRPYGNRGRWGENESEEKEHP--TMESVT 286 
2-2412    FGDRPFGRR-------------------------------------------------------------------------------------------------------------------------------------------NGTEEGSPRRDGQRRPYGNRGRWGENESEEKEHP--TMESVT 286 
2-2413    FGDRPFGRR-------------------------------------------------------------------------------------------------------------------------------------------NGTEEGSPRRDGQRRPYGNRGRWGENESEEKEHP--TMESVT 286 
2-2415    FGDRPFGRR-------------------------------------------------------------------------------------------------------------------------------------------NGTEEGSPRRDGQRRPYGNRGRWGENESEEKEHP--TMESVT 286 
2-2416    FGDRPFGRR-------------------------------------------------------------------------------------------------------------------------------------------NGTEEGSPRRDGQRRPYGNRGRWGENESEEKEHP--TMESVT 286 
2-2417    FGDRPFGRR-------------------------------------------------------------------------------------------------------------------------------------------NGTEEGSPRRDGQRRPYGNRGRWGENESEEKEHP--TMESVT 286 
2-2418    FGDRPFGRR-------------------------------------------------------------------------------------------------------------------------------------------NGTEEGSPRRDGQRRPYGNRGRWGENESEEKEHP--TMESVT 286 
2-2419    FGDRPFGRR-------------------------------------------------------------------------------------------------------------------------------------------NGTEEGSPRRDGQRRPYGNRGRWGENESEEKEYP--TTESVT 286 
2-2420    FGDRPFGRR-------------------------------------------------------------------------------------------------------------------------------------------NGTEEGSPRRDGQRRPYGNRGRWGENESEEKEHP--TMESVT 286 
2-2421    FGDRPFGRP-------------------------------------------------------------------------------------------------------------------------------------------NGTEEGSPRRDGQRRPYGNRGRWGENESEEKEHP--TTESVT 286 
2-2422    FGDRPFGRR-------------------------------------------------------------------------------------------------------------------------------------------NGTEEGSPRRDGQRRPYGNRGRWGENESEEKEHP--TMESVT 286 
2-2424    FGDRPFGRR-------------------------------------------------------------------------------------------------------------------------------------------NGTEEGSPRRDGQRRPYGNRGRWGENESEEKEHP--TMESVT 286 
2-2425    FGDRPFGRR-------------------------------------------------------------------------------------------------------------------------------------------NGTEEGSPRRDGQRRPYGNRGRWGENESEEKEHP--TMESVT 286 
2-2426    FGDRPFGRR-------------------------------------------------------------------------------------------------------------------------------------------NGTEEGSPRRDGQRRPYGNRGRWGENESEEKEHP--TMESVT 286 
2-2427    FGDRPFGRR-------------------------------------------------------------------------------------------------------------------------------------------NGTEEGSPRRDGQRRPYGNRGRWGENESEEKEHP--TMESVT 286 
2-2430    FGDRPFGRR-------------------------------------------------------------------------------------------------------------------------------------------NGTEEGSPRRDGQRRPYGNRGRWGENESEEKEHP--TMESVT 286 
2-2431    FGDRPFGRR-------------------------------------------------------------------------------------------------------------------------------------------NGTEEGSPRRDGQRRPYGNRGRWGENESEEKEHP--TMESVT 286 
2-2432    FGDRPFGRR-------------------------------------------------------------------------------------------------------------------------------------------SGTEEGSPRRDGQRRPYGNRGRWGENESEEKEHP--TMESVT 286 
2-2434    FGDRPFGRR-------------------------------------------------------------------------------------------------------------------------------------------NGTEEGSPRRDGQRRPYGNRGRWGENESEEKEHP--TMESVT 286 
2-2437    FGDRPFGRR-------------------------------------------------------------------------------------------------------------------------------------------NGTEEGSPRRDGQRRPYGNRGRWGENESEEKEHP--TMESVT 286 
2-2438    FGDRPFGRR-------------------------------------------------------------------------------------------------------------------------------------------NGTEEGSPRRDGQRRPYGNRGRWGENESEEKEHP--TMESVT 286 
2-2439    FGDRPFGRR-------------------------------------------------------------------------------------------------------------------------------------------SGTEEGSPRRDGQRRPYGNRGRWGENESEEKEHP--TMESVT 286 
2-2440    FGDRPFGRR-------------------------------------------------------------------------------------------------------------------------------------------NGTEEGSPRRDGQRRPYGNRGRWGENESEEKEHP--TMESVT 286 
2-2442    FGDRPFGRR-------------------------------------------------------------------------------------------------------------------------------------------NGTEEGSPRRDGQRRPYGNRGRWGENESEEKEHP--TMESVT 286 
2-2445    FGDRPFGRP-------------------------------------------------------------------------------------------------------------------------------------------NGTEEGSPRRDGQRRPYGNRGRWGENESEEKEHP--TTESVT 286 
2-2446    FGDRPFGRR-------------------------------------------------------------------------------------------------------------------------------------------NGTEEGSPRRDGQRRPYGNRGRWGENESEEKEHP--TMESVT 286 
7-1501                                                                                                                                                                                                   142 
7-1502                                                                                                                                                                                                   142 
7-1503                                                                                                                                                                                                   142 
7-1504                                                                                                                                                                                                   142 
7-1505                                                                                                                                                                                                   142 
7-1506                                                                                                                                                                                                   142 
7-1508                                                                                                                                                                                                   142 
7-1509                                                                                                                                                                                                   142 
7-1510                                                                                                                                                                                                   142 
7-1511                                                                                                                                                                                                   142 
7-1512                                                                                                                                                                                                   142 
7-1513                                                                                                                                                                                                   142 
7-1515                                                                                                                                                                                                   142 
7-1516                                                                                                                                                                                                   142 
7-1517                                                                                                                                                                                                   142 
7-1519                                                                                                                                                                                                   142 
7-1520                                                                                                                                                                                                   142 
7-1521                                                                                                                                                                                                   142 
7-1523                                                                                                                                                                                                   142 
7-1524                                                                                                                                                                                                   142 
7-1525                                                                                                                                                                                                   142 
7-1526                                                                                                                                                                                                   142 
7-1527                                                                                                                                                                                                   142 
7-1528                                                                                                                                                                                                   142 
7-1529                                                                                                                                                                                                   142 
7-1530                                                                                                                                                                                                   142 
7-1533                                                                                                                                                                                                   142 
7-1534                                                                                                                                                                                                   142 
7-1536                                                                                                                                                                                                   142 
7-1537                                                                                                                                                                                                   142 
7-1538                                                                                                                                                                                                   142 
7-1542                                                                                                                                                                                                   142 
7-1544                                                                                                                                                                                                   142 
7-1545                                                                                                                                                                                                   142 
7-1546                                                                                                                                                                                                   142 
7-1548                                                                                                                                                                                                   142 
7-1549                                                                                                                                                                                                   142 
7-1539                                                                                                                                                                                                   142 
7-1550                                                                                                                                                                                                   142 
7-1518    FGDRPFGRR-------------------------------------------------------------------------------------------------------------------------------------------NGTEEGPPRRDGQRRPYGNRGRWGENESEEKEHP--TTESVT 286 
7-1540                                                                                                                                                                                                   79  
7-1514    FGDRPFGRR-------------------------------------------------------------------------------------------------------------------------------------------NGTEEGSPRRDGQRRPYGNRGRWGENESEEKEHP--TTESVT 286 
7-1507    FGDRPFGRR-------------------------------------------------------------------------------------------------------------------------------------------NGTEEGSPRRDGQRRPYGNRGRWGENESEEKEHP--TMESVT 286 
7-1522    FGDRPFGRR-------------------------------------------------------------------------------------------------------------------------------------------NGTEEGSPRRDGQRRPYGNRGRWGENESEEKEHP--TTESVT 286 
7-1532    FGDRPFGRR-------------------------------------------------------------------------------------------------------------------------------------------NGTEEGSPRRDGQRRPYGNRGRWGENESEEKEHP--TMESVT 286 
7-1543    FGDRPFGRR-------------------------------------------------------------------------------------------------------------------------------------------NGTEEGSPRRDGQRRPYGNRGRWGENESEEKEHP--TTESVT 286 
7-1547    FGDRPFGRR-------------------------------------------------------------------------------------------------------------------------------------------NGTEEGSPRRDGQRRPYGNRGRWGENESEEKEHP--TMESVT 286 
7-2401                                                                                                                                                                                                   142 
7-2402                                                                                                                                                                                                   142 
7-2403                                                                                                                                                                                                   142 
7-2404                                                                                                                                                                                                   142 
7-2405                                                                                                                                                                                                   142 
7-2406                                                                                                                                                                                                   142 
7-2407                                                                                                                                                                                                   142 
7-2408                                                                                                                                                                                                   142 
7-2409                                                                                                                                                                                                   142 
7-2410                                                                                                                                                                                                   142 
7-2411                                                                                                                                                                                                   142 
7-2412                                                                                                                                                                                                   142 
7-2413                                                                                                                                                                                                   142 
7-2414                                                                                                                                                                                                   142 
7-2416                                                                                                                                                                                                   142 
7-2417                                                                                                                                                                                                   142 
7-2418                                                                                                                                                                                                   142 
7-2420                                                                                                                                                                                                   142 
7-2421                                                                                                                                                                                                   142 
7-2423                                                                                                                                                                                                   142 
7-2424                                                                                                                                                                                                   142 
7-2425                                                                                                                                                                                                   142 
7-2426                                                                                                                                                                                                   142 
7-2430                                                                                                                                                                                                   142 
7-2431                                                                                                                                                                                                   142 
7-2432                                                                                                                                                                                                   142 
7-2436                                                                                                                                                                                                   142 
7-2437                                                                                                                                                                                                   142 
7-2439                                                                                                                                                                                                   142 
7-2442                                                                                                                                                                                                   142 
7-2443                                                                                                                                                                                                   142 
7-2444                                                                                                                                                                                                   142 
7-2445                                                                                                                                                                                                   142 
7-2446                                                                                                                                                                                                   142 
7-2447                                                                                                                                                                                                   142 
7-2448                                                                                                                                                                                                   142 
7-2450                                                                                                                                                                                                   142 
7-2415    FGDRPFGRR-------------------------------------------------------------------------------------------------------------------------------------------NGTEEGSPRRDGQRRPYGNRGRWGENESEEKEHP--TMESVT 286 
7-2427    FGDRPFGRR-------------------------------------------------------------------------------------------------------------------------------------------NGTEEGSPRRDGQRRPYGNRGRWGENESEEKEHP--TMESVT 286 
7-2428    FGDRPFGRR-------------------------------------------------------------------------------------------------------------------------------------------NGTEEGSPRRDGQRRPYGNRGRWGENESEEKEHP--TTESVT 286 
7-2435    FGDRPFGRR-------------------------------------------------------------------------------------------------------------------------------------------NGTEEGSPRRDGQRRPYGNRGRWGENESEEKEHP--TTESVT 286 
7-2440    FGDRPFGRR-------------------------------------------------------------------------------------------------------------------------------------------NGTEEGSPRRDGQRRPYGNRGRWGENESEEKEHP--TMESVT 286 
7-2441    FGDRPFGRR-------------------------------------------------------------------------------------------------------------------------------------------NGTEEGSPRRDGQRRPYGNRGRWGENESEEKEHP--TTESVT 286 
7-2449    FGDRPFGRR-------------------------------------------------------------------------------------------------------------------------------------------NGTEEGSPRRDGQRRPYGNRGRWGENESEEKEHP--TTESVT 286 
8-1501                                                                                                                                                                                                   142 
8-1502                                                                                                                                                                                                   142 
8-1503                                                                                                                                                                                                   142 
8-1504                                                                                                                                                                                                   142 
8-1505                                                                                                                                                                                                   142 
8-1506                                                                                                                                                                                                   142 
8-1507                                                                                                                                                                                                   142 
8-1508                                                                                                                                                                                                   142 
8-1509                                                                                                                                                                                                   142 
8-1510                                                                                                                                                                                                   142 
8-1511                                                                                                                                                                                                   142 
8-1512                                                                                                                                                                                                   142 
8-1513                                                                                                                                                                                                   142 
8-1514                                                                                                                                                                                                   142 
8-1515                                                                                                                                                                                                   142 
8-1516                                                                                                                                                                                                   142 
8-1517                                                                                                                                                                                                   142 
8-1518                                                                                                                                                                                                   142 
8-1519                                                                                                                                                                                                   142 
8-1520                                                                                                                                                                                                   142 
8-1521                                                                                                                                                                                                   142 
8-1522                                                                                                                                                                                                   142 
8-1524                                                                                                                                                                                                   142 
8-1525                                                                                                                                                                                                   142 
8-1526                                                                                                                                                                                                   142 
8-1527                                                                                                                                                                                                   142 
8-1528                                                                                                                                                                                                   142 
8-1529                                                                                                                                                                                                   142 
8-1530                                                                                                                                                                                                   142 
8-1531                                                                                                                                                                                                   142 
8-1532                                                                                                                                                                                                   142 
8-1533                                                                                                                                                                                                   142 
8-1534                                                                                                                                                                                                   142 
8-1535                                                                                                                                                                                                   142 
8-1536                                                                                                                                                                                                   142 
8-1537                                                                                                                                                                                                   142 
8-1538                                                                                                                                                                                                   142 
8-1539                                                                                                                                                                                                   142 
8-1540                                                                                                                                                                                                   142 
8-1542                                                                                                                                                                                                   142 
8-1546                                                                                                                                                                                                   142 
8-1548                                                                                                                                                                                                   142 
8-1549                                                                                                                                                                                                   142 
8-1550                                                                                                                                                                                                   142 
8-1541    FGDRPFGRR-------------------------------------------------------------------------------------------------------------------------------------------NGTEEGSPRRDGQRRPYGNRGRWGENESEEKEHP--TMESVT 286 
8-1543    FGDRPFGRR-------------------------------------------------------------------------------------------------------------------------------------------NGTEEGSPRRDGQRRPYGNRGRWGENESEEKEHP--TMESVT 286 
8-2401                                                                                                                                                                                                   142 
8-2450                                                                                                                                                                                                   142 
8-2406                                                                                                                                                                                                   142 
8-2407                                                                                                                                                                                                   142 
8-2409                                                                                                                                                                                                   142 
8-2410                                                                                                                                                                                                   142 
8-2411                                                                                                                                                                                                   142 
8-2414                                                                                                                                                                                                   142 
8-2417                                                                                                                                                                                                   142 
8-2420                                                                                                                                                                                                   142 
8-2421                                                                                                                                                                                                   142 
8-2422                                                                                                                                                                                                   142 
8-2424                                                                                                                                                                                                   142 
8-2425                                                                                                                                                                                                   142 
8-2427                                                                                                                                                                                                   142 
8-2433                                                                                                                                                                                                   142 
8-2434                                                                                                                                                                                                   142 
8-2435                                                                                                                                                                                                   142 
8-2436                                                                                                                                                                                                   142 
8-2437                                                                                                                                                                                                   142 
8-2438                                                                                                                                                                                                   142 
8-2439                                                                                                                                                                                                   142 
8-2440                                                                                                                                                                                                   142 
8-2442                                                                                                                                                                                                   142 
8-2444                                                                                                                                                                                                   142 
8-2445                                                                                                                                                                                                   142 
8-2448                                                                                                                                                                                                   142 
8-2449                                                                                                                                                                                                   142 
8-2432    FGGRPFDRR-------------------------------------------------------------------------------------------------------------------------------------------NGTEEGSPRRDGHRRPYGNRGRWGENESEEKEHP--TTESVT 355 
8-2405    FGGRPFDRR-------------------------------------------------------------------------------------------------------------------------------------------NGTEEGSPRRDGHRRPYGNRGRWGENESEEKEHP--TTESVT 355 
8-2443    FGDRPFGRR-------------------------------------------------------------------------------------------------------------------------------------------NGTEEGSPRRDGQRRPYGNRGRWGENESEEKEHP--TMESVT 286 
8-2413    FGDRPFGRR-------------------------------------------------------------------------------------------------------------------------------------------NGTEEGSPRRDGQRRPYGNRGRWGENESEEKEHP--TMESVT 286 
8-2418    FGDRPFGRR-------------------------------------------------------------------------------------------------------------------------------------------NGTEEGSPRRDGQRRPYGNRGRWGENESEEKEHP--TMESVT 286 
8-2419    FGDRPFGRR-------------------------------------------------------------------------------------------------------------------------------------------NGTEEGSPRRDGQRRPYGNRGRWGENESEEKEHP--TMESVT 286 
8-2423    FGDRPFGRR-------------------------------------------------------------------------------------------------------------------------------------------NGTEEGSPRRDGQRRPYGNRGRWGENESEEKEHP--TMESVT 286 
8-2430    FGDRPFGRR-------------------------------------------------------------------------------------------------------------------------------------------NGTEEGSPRRDGQRRPYGNRGRWGENESEEKEHP--TTESVT 286 
8-2446    FGDRPFGRR-------------------------------------------------------------------------------------------------------------------------------------------NGTEEGSPRRDGQRRPYGNRGRWGENESEEKEHP--TMESVT 286 
8-2447    FGDRPFGRR-------------------------------------------------------------------------------------------------------------------------------------------NGTEEGSPRRDGQRRPYGNRGRWGENESEEKEHP--TMESVT 286 
8-2441    FGDRPFGRR-------------------------------------------------------------------------------------------------------------------------------------------NGTEEGSPRRDGQRRPHGNRGRWGENESEEKEHP--TTESVT 255 
8-2404    FGDRPFGRR-------------------------------------------------------------------------------------------------------------------------------------------NGTEEGSPRRDGQRRPYGNRGRWGENESEEKKHP--TTESVT 286 
8-2415                                                                                                                                                                                                   224 
2-1517                                                                                                                                                                                                   142 
2-1501                                                                                                                                                                                                   142 
2-1503                                                                                                                                                                                                   142 
2-1505                                                                                                                                                                                                   142 
2-1508                                                                                                                                                                                                   142 
2-1509                                                                                                                                                                                                   142 
2-1510                                                                                                                                                                                                   142 
2-1519                                                                                                                                                                                                   142 
2-1521                                                                                                                                                                                                   142 
2-1522                                                                                                                                                                                                   142 
2-1523                                                                                                                                                                                                   142 
2-1527                                                                                                                                                                                                   142 
2-1533                                                                                                                                                                                                   142 
2-1535                                                                                                                                                                                                   142 
2-1536                                                                                                                                                                                                   142 
2-1537                                                                                                                                                                                                   142 
2-1538                                                                                                                                                                                                   142 
2-1539                                                                                                                                                                                                   142 
2-1543                                                                                                                                                                                                   142 
2-1546                                                                                                                                                                                                   142 
2-1547                                                                                                                                                                                                   142 
2-1529    FGDRLFGRR-------------------------------------------------------------------------------------------------------------------------------------------NGTEEGSPRRDGHRRPYGNRGRWGENESEEKEHP--TTESVT 358 
2-1540    FGGRPFDRR-------------------------------------------------------------------------------------------------------------------------------------------NGTEEGSPRRDGHRRPYGNRGRWGENESEEKEHP--TTESVT 355 
2-1506    FGDRPFGRR-------------------------------------------------------------------------------------------------------------------------------------------NGTEEGSPRRDGQRRPYGNRGRWGENESEEKEHP--TMESVT 286 
2-1507    FGDRPFGRR-------------------------------------------------------------------------------------------------------------------------------------------NGTEEGSPRRDGQHRPYGNRGRWGENESEEKEHP--TTESVT 286 
2-1511    FGDRPFGRR-------------------------------------------------------------------------------------------------------------------------------------------NGTEEGSPRRDGQHRPYGNRGRWGENESEEKEHP--TAESVT 286 
2-1514    FGDRPFGRR-------------------------------------------------------------------------------------------------------------------------------------------NGTEEGSPRRDGQRRPYGNRGRWGENESEEKEYP--TTESVT 286 
2-1516    FGDRPFGRR-------------------------------------------------------------------------------------------------------------------------------------------NGTEEGSPRRDGQRRPYGNRGRWGENESEEKEHP--TMESVT 286 
2-1528    FGDRPFGRP-------------------------------------------------------------------------------------------------------------------------------------------NGTEEGSPRRDGQRRPYGNRGRWGENESEEKEHP--TTESVT 286 
2-1532    FGDRPFGRR-------------------------------------------------------------------------------------------------------------------------------------------NGTEEGSPRRDGQRRPYGNRGRWGENESEEKEHP--TMESVT 286 
2-1541    FGDRPFGRR-------------------------------------------------------------------------------------------------------------------------------------------NGTEEGSPRRDGQHRPYGNRGRWGENESEEKEHP--TTESVT 286 
2-1542    FGDRPFGRR-------------------------------------------------------------------------------------------------------------------------------------------NGTEEGSPRRDGQRRPYGNRGRWGENESEEKEHP--TMESVT 286 
2-2448                                                                                                                                                                                                   142 
2-2405                                                                                                                                                                                                   142 
2-2406                                                                                                                                                                                                   142 
2-2407                                                                                                                                                                                                   142 
2-2408                                                                                                                                                                                                   142 
2-2410                                                                                                                                                                                                   142 
2-2412                                                                                                                                                                                                   142 
2-2413                                                                                                                                                                                                   142 
2-2419                                                                                                                                                                                                   142 
2-2420                                                                                                                                                                                                   142 
2-2422                                                                                                                                                                                                   142 
2-2424                                                                                                                                                                                                   142 
2-2427                                                                                                                                                                                                   142 
2-2428                                                                                                                                                                                                   142 
2-2429                                                                                                                                                                                                   142 
2-2431                                                                                                                                                                                                   142 
2-2436                                                                                                                                                                                                   142 
2-2441                                                                                                                                                                                                   142 
2-2446                                                                                                                                                                                                   142 
2-2416                                                                                                                                                                                                   121 
2-2411                                                                                                                                                                                                   45  
2-2415                                                                                                                                                                                                   45  
2-2440                                                                                                                                                                                                   45  
2-2423    ---------------------------------------------------------------------------------------------------------------------------------------------------------------------------------------------- 1   
2-2418                                                                                                                                                                                                   131 
2-2421    FGGRPFDRR-------------------------------------------------------------------------------------------------------------------------------------------NGTEEGSPRRDGHRRPYGNRGRWGENESEEKEHP--TTESVT 331 
2-2438    FGGRPFDRR-------------------------------------------------------------------------------------------------------------------------------------------NGTEEGSPRRDGQRRPYGNRGRWGENESEEKEHP--TMESVT 331 
2-2414    FGDRPFGRR-------------------------------------------------------------------------------------------------------------------------------------------NGTEEGSPRRDGQRRPYGNRGRWGENESEEKEHP--TMESVT 286 
2-2403    FGDRPFGRR-------------------------------------------------------------------------------------------------------------------------------------------NGTEEGSPRRDGQRRPYGNRGRWGENESEEKEHP--TMESVT 286 
2-2404    FGDRPFGRR-------------------------------------------------------------------------------------------------------------------------------------------NGTEEGSPRRDGQRRPYGNRGRWGENESEEKEYP--TTESVT 285 
2-2425    FGDRPFGRR-------------------------------------------------------------------------------------------------------------------------------------------NGTEEGSPRRDGQRRPYGNRGRWGENESEEKEYP--TTESVT 285 
2-2426    FGDRPFGRR-------------------------------------------------------------------------------------------------------------------------------------------NGTEEGSPRRDGQRRPYGNRGRWGENESEEKEHP--TMESVT 286 
2-2430    FGDRPFGRR-------------------------------------------------------------------------------------------------------------------------------------------NGTEEGSPRRDGQRRPYGNRGRWGENESEEKEHP--TTESVT 286 
2-2432    FGDRPFGRR-------------------------------------------------------------------------------------------------------------------------------------------NGTEEGSPRRDGQRRPYGNRGRWGENESEEKEHP--TMESVT 285 
2-2437    FGDRPFGRR-------------------------------------------------------------------------------------------------------------------------------------------NGTEEGSPRRDGQRRPYGNRGRWGENESEEKEHP--TMESVT 285 
2-2439    FGDRPFGRR-------------------------------------------------------------------------------------------------------------------------------------------NGTEEGSPRRDGQRRPYGNRGRWGENESEEKEHP--TMESVT 286 
2-2442    FGDRPFGRR-------------------------------------------------------------------------------------------------------------------------------------------NGTEEGSPRRDGQRRPYGNRGRWGENESEEKEHP--TMESVT 286 
2-2443    FGDRPFGRR-------------------------------------------------------------------------------------------------------------------------------------------NGTEEGSPRRDGQRRPYGNRGRWGENESEEKEHP--TMESVT 286 
2-2445    FGDRPFGRR-------------------------------------------------------------------------------------------------------------------------------------------NGTEEGSPRRDGQRRPYGNRGRWGENESEEKEHP--TMESVT 286 
2-2435    FGDRPFGRR-------------------------------------------------------------------------------------------------------------------------------------------NGTEEGSPRRDGQRRPYGNRGRWGENESEEKEHP--TMESVT 256 
2-2447    FGDRPFGRR-------------------------------------------------------------------------------------------------------------------------------------------NGTEEGSPRRDGQRRPYGNRGRWGENESEEKEHP--TMESVT 256 
2-2401    FGDRPFGRR-------------------------------------------------------------------------------------------------------------------------------------------NGTEEGSPRRDGQRRPYGNRGRWGENESEEKEHP--TMESVT 256 
2-2409    FGGRPFDRR-------------------------------------------------------------------------------------------------------------------------------------------NGTEEGSPRRDGHRRPYGNRGRWGENESEEKEHP--TTESVT 355 
2-2444    FGGRPFDRR-------------------------------------------------------------------------------------------------------------------------------------------NGTEEGSPRRDGHRRPYGNRGRWGENESEEKEHP--TTESVT 355 
9-1504                                                                                                                                                                                                   142 
9-1505                                                                                                                                                                                                   142 
9-1506                                                                                                                                                                                                   142 
9-1507                                                                                                                                                                                                   142 
9-1509                                                                                                                                                                                                   142 
9-1512                                                                                                                                                                                                   142 
9-1514                                                                                                                                                                                                   142 
9-1515                                                                                                                                                                                                   142 
9-1516                                                                                                                                                                                                   142 
9-1518                                                                                                                                                                                                   142 
9-1519                                                                                                                                                                                                   142 
9-1520                                                                                                                                                                                                   142 
9-1521                                                                                                                                                                                                   142 
9-1523                                                                                                                                                                                                   142 
9-1524                                                                                                                                                                                                   142 
9-1526                                                                                                                                                                                                   142 
9-1527                                                                                                                                                                                                   142 
9-1530                                                                                                                                                                                                   142 
9-1531                                                                                                                                                                                                   142 
9-1533                                                                                                                                                                                                   142 
9-1537                                                                                                                                                                                                   142 
9-1538                                                                                                                                                                                                   142 
9-1539                                                                                                                                                                                                   142 
9-1540                                                                                                                                                                                                   142 
9-1543                                                                                                                                                                                                   142 
9-1544                                                                                                                                                                                                   142 
9-1545                                                                                                                                                                                                   142 
9-1546                                                                                                                                                                                                   142 
9-1547                                                                                                                                                                                                   142 
9-1548                                                                                                                                                                                                   142 
9-1513    FGGRPFDRR-------------------------------------------------------------------------------------------------------------------------------------------NGTEEGSPRRDGHRRPYGTRGRWGENESEEKEHP--TTESVT 355 
9-1541    FGGRPFDRR-------------------------------------------------------------------------------------------------------------------------------------------NGTEEGSPRRDGHRRPYGNRGRWGENESEEKEHP--TTESVT 355 
9-1542    FGGRPFDRR-------------------------------------------------------------------------------------------------------------------------------------------NGTEEGSPRRDGHRRPYGNRGRWGENESEEKEHP--TTESVT 355 
9-1525    FGDHPFGRRNHTEGHQGHNETGDHPHRHHSKNVDGDQDTGHHGHHGHHEHHHHQHDHREGHQDHDRPMFEMRPFRFNPLGRKPFGDHPFGRRNHTEGHQGHNETGDHPHRHHSKTGDGDQDRPMFETRPFWVNPFGRRPFGDRPFDRRNGTEEGSPRRDGHPHPHGNRGRWGENESEEKEHP--TTESVT 466 
9-1501    FGDRPFGRR-------------------------------------------------------------------------------------------------------------------------------------------NGTEEGSPRRDGQRRPYGNRGRWGENESEEKEHP--TMESVT 286 
9-1502    FGDRPFGRR-------------------------------------------------------------------------------------------------------------------------------------------NGTEEGSPRRDGQRRPYGNRGRWGESESEEKEHP--TMESVT 286 
9-1508    FGDRPFGRR-------------------------------------------------------------------------------------------------------------------------------------------NGTEEGSPRRDGQRRPYGNRGRWGENESEEKEHP--TMESVT 286 
9-1511    FGDRPFGRR-------------------------------------------------------------------------------------------------------------------------------------------NGTEEGSPRRDGQRRPYGNRGRWGENESEEKEHP--TMESVT 286 
9-1517    FGDRPFGRR-------------------------------------------------------------------------------------------------------------------------------------------NGTEEGSPRRDGQRRPYGNRGRWGENESEEKEHP--TMESVT 286 
9-1528    FGDRPFGRR-------------------------------------------------------------------------------------------------------------------------------------------NGTEEGSPRRDGQRRPYGNRGRWGENESEEKEHP--TMESVT 286 
9-1529    FGDRPFGRR-------------------------------------------------------------------------------------------------------------------------------------------NGTEEGSPRRDGQRRPYGNRGRWGENESEEKEHP--TMESVT 286 
9-1534    FGDRPFGRR-------------------------------------------------------------------------------------------------------------------------------------------NGTEEGSPRRDGQRRPYGNRGRWGENESEEKEHP--TMESVT 286 
9-1535    FGDRPFGRR-------------------------------------------------------------------------------------------------------------------------------------------NGTEEGSPRRDGQRRPYGNRGRWGENESEEKEHP--TMESVT 286 
9-2401                                                                                                                                                                                                   142 
9-2402                                                                                                                                                                                                   142 
9-2404                                                                                                                                                                                                   142 
9-2406                                                                                                                                                                                                   142 
9-2408                                                                                                                                                                                                   142 
9-2409                                                                                                                                                                                                   142 
9-2411                                                                                                                                                                                                   142 
9-2413                                                                                                                                                                                                   142 
9-2414                                                                                                                                                                                                   142 
9-2417                                                                                                                                                                                                   142 
9-2418                                                                                                                                                                                                   142 
9-2419                                                                                                                                                                                                   142 
9-2420                                                                                                                                                                                                   142 
9-2421                                                                                                                                                                                                   142 
9-2424                                                                                                                                                                                                   142 
9-2427                                                                                                                                                                                                   142 
9-2429                                                                                                                                                                                                   142 
9-2430                                                                                                                                                                                                   142 
9-2433                                                                                                                                                                                                   142 
9-2435                                                                                                                                                                                                   142 
9-2438                                                                                                                                                                                                   142 
9-2439                                                                                                                                                                                                   142 
9-2440                                                                                                                                                                                                   142 
9-2441                                                                                                                                                                                                   142 
9-2442                                                                                                                                                                                                   142 
9-2443                                                                                                                                                                                                   142 
9-2445                                                                                                                                                                                                   142 
9-2446                                                                                                                                                                                                   142 
9-2448                                                                                                                                                                                                   142 
9-2416    FGGRPFDRR-------------------------------------------------------------------------------------------------------------------------------------------NGTEEGSPRRDGHRRPYGNRGRWGENESEEKEHP--TTESVT 355 
9-2423    FGGRPFDRR-------------------------------------------------------------------------------------------------------------------------------------------NGTEEGSPRRDGHRRPYGNRGRWGENESEEKEHP--TTESVT 355 
9-2403    FGDRPFGRR-------------------------------------------------------------------------------------------------------------------------------------------NGTEEGSPRRDGQRRPYGNRGRWGENESEEKEHP--TMESVT 286 
9-2415    FGDRPFGRR-------------------------------------------------------------------------------------------------------------------------------------------NGTEEGSPRRDGQRRPYGNRGRWGENESEEKEHP--TMESVT 286 
9-2422    FGDRPFGRR-------------------------------------------------------------------------------------------------------------------------------------------NGTEEGSPRRDGQHRPYGNRGRWGENESEEKEHP--TTESVT 286 
9-2428    FGDRPFGRR-------------------------------------------------------------------------------------------------------------------------------------------NGTEEGSPRRDGQRRPYGNRGRWGENESEEKEHP--TMESVT 286 
9-2431    FGDRPFGRR-------------------------------------------------------------------------------------------------------------------------------------------NGTEEGSPRRDGQRRPYGNRGRWGENESEEKEHP--TMESVT 286 
9-2432    FGDRPFGRR-------------------------------------------------------------------------------------------------------------------------------------------NGTEEGSPRRDGQRRPYGNRGRWGENESEEKEHP--TMESVT 286 
9-2434    FGDRPFGRR-------------------------------------------------------------------------------------------------------------------------------------------NGTEEGSPRRDGQRRPYGNRGRWGENESEEKEHP--TMESVT 286 
9-2437    LGDRPFGRR-------------------------------------------------------------------------------------------------------------------------------------------NGTEEGSPRRDGQRRPYGNRGRWGENESEEKEHP--TMESVT 286 
9-2444    FGDRPFGRR-------------------------------------------------------------------------------------------------------------------------------------------NGTEEGSPRRDGQRRPYGNRGRWGENESEEKEHP--TMESVT 286 
4-1504    FGGRPFDRR-------------------------------------------------------------------------------------------------------------------------------------------NGTEEGSPRRDGHRRPYGNRGRWGENESEEKEHP--TTESVT 355 
4-1522    FGGRPFDRR-------------------------------------------------------------------------------------------------------------------------------------------NGTEEGSPRRDGHRRPYGNRGRWGENESEEKEHP--TTESRN 331 
4-1507    FGDRPFGRP-------------------------------------------------------------------------------------------------------------------------------------------NGTEEGSPRRDGQRRPYGNRGRWGENESEEKEHP--TTESVT 286 
4-1519                                                                                                                                                                                                   142 
4-1529                                                                                                                                                                                                   142 
4-1549                                                                                                                                                                                                   142 
4-1510                                                                                                                                                                                                   142 
4-1539                                                                                                                                                                                                   142 
4-2424    FGDRPFGRR-------------------------------------------------------------------------------------------------------------------------------------------NGTEEGSPRRDGQRRPYGNRGRWGENESEEKEHP--TTESVT 285 
4-2401    FGDRPFGRR-------------------------------------------------------------------------------------------------------------------------------------------NGTEEGSPRRDGQRRPYGNRGRWGENESEEKEHP--MTESVT 286 
4-2405    FGDRPFGRR-------------------------------------------------------------------------------------------------------------------------------------------NGTEEGSPRRDGQRRPYGNRGRWGENESEEKEHP--TTESVT 286 
4-2407    FGDRPFGRR-------------------------------------------------------------------------------------------------------------------------------------------NGTEEGSPRRDGQRRPYGNRGRWGENESEEKEHP--TTESVT 286 
4-2408    FGDRPFGRR-------------------------------------------------------------------------------------------------------------------------------------------NGTEEGSPRRDGQRRPYGNRGRWGENESEEKEHP--TTESVT 286 
4-2411    FGDRPFGRR-------------------------------------------------------------------------------------------------------------------------------------------NGTEEGSPRRDGQRRPYGNRGRWGENESEEKEHP--TTESVT 286 
4-2417    FGDRPFGRR-------------------------------------------------------------------------------------------------------------------------------------------NGTEEGSPRRDGQRRPYGNRGRWGEDESEEKEHP--TTESVT 286 
4-2418    FGDRPFGRR-------------------------------------------------------------------------------------------------------------------------------------------NGTEEGSPRRDGQRRPYGNRGRWGENESEEKEHP--TTESVT 286 
4-2419    FGDRPFGRR-------------------------------------------------------------------------------------------------------------------------------------------NGTEEGSPRRDGQRRPYGNRGRWGENESEEKEHP--TTESVT 286 
4-2421    FGDCPFGRR-------------------------------------------------------------------------------------------------------------------------------------------NGTEEGSPRRDGQRRPYGNRGRWGENESEEKEHP--TTESVT 286 
4-2422    FGDRPFGRR-------------------------------------------------------------------------------------------------------------------------------------------NGTEEGSPRRDGQRRPYGNRGRWGENESEEKEHP--TTESVT 286 
4-2426    FGDRPFGRR-------------------------------------------------------------------------------------------------------------------------------------------NGTEEGSPRRDGQRRPYGNRGRWGENESEEKEHP--TTESVT 286 
4-2428    FGDRPFGRR-------------------------------------------------------------------------------------------------------------------------------------------NGTEEGSPRRDGQRRPYGNRGRWGENESEEKEHP--TTESVT 286 
4-2429    FGDRPFGRR-------------------------------------------------------------------------------------------------------------------------------------------NGTEEGSPRRDGQRRPYGNRGRWGENESEEKEHP--TTESVT 286 
4-2435    FGDRPFGRR-------------------------------------------------------------------------------------------------------------------------------------------NGTEEGSPRRDGQRRPYGNRGRWGENESEEKEHP--TTESVT 286 
4-2437    FGDRPFGRR-------------------------------------------------------------------------------------------------------------------------------------------NGTEEGSPRRDGQRRPYGNRGRWGENESEEKEHP--TMESVT 286 
4-2444    FGDRPFGRR-------------------------------------------------------------------------------------------------------------------------------------------NGTEEGSPRRDGQRRPYGNRGRWGENESEEKEHP--TTEGVT 286 
4-2445    FGDRPFGRR-------------------------------------------------------------------------------------------------------------------------------------------NGTEEGSPRRDGQRRPYGNRGRWGENESEEKEHP--TTESVT 286 
4-2447    FGDRPFGRR-------------------------------------------------------------------------------------------------------------------------------------------NGTEEGSPRRDGQRRPYGNRGRWGENESEEKEHP--TTESVT 286 
4-2448    FGDRPFGRR-------------------------------------------------------------------------------------------------------------------------------------------NGTEEGSPRRDGQRRPYGNRGRWGENESEEKEHP--TMESVT 286 
4-2404    FGGRPFDRR-------------------------------------------------------------------------------------------------------------------------------------------NGTEEGSPRRDGHRRPYGNRGRWGENESEEKEHP--TTESVT 355 
4-2413    FGGRPFDRR-------------------------------------------------------------------------------------------------------------------------------------------NGTEEGSPRRDGHRRPYGNRGRRSENESEEKEHP--TTESVT 355 
4-2450    FGGRPFDRR-------------------------------------------------------------------------------------------------------------------------------------------NGTEEGSPRRDGHRRPYGNRGRWSENGSEEKEHP--TTESVT 355 
4-2420    FGDRPFDRR-------------------------------------------------------------------------------------------------------------------------------------------NGTEEGSPRRDGHRHPYGNRGRWGENESEEKEHP--TTESVA 356 
4-2438    FGGRPFDRR-------------------------------------------------------------------------------------------------------------------------------------------NGTEEGSPRRDGHRRPYGNRGRWGENESEEKEHP--TTESVT 356 
4-2430    ----------------------------------------------------------------------------------------------------------------------------------------------------NGTEEGSPRRDGHRRPYGNRGRWGENESEEKEHP--TTESVT 300 
4-2439    FGDRPFDRR-------------------------------------------------------------------------------------------------------------------------------------------NGTEEGSPRRDGHRHPYGNRGRWGENESEEKEHP--TTESVA 331 
4-2433    --------------------------------------------------------------------------------------------------------------------------------PFWVNPFGRKPFGDRPFGRRNGTEEGSPRRDGHRHPYGNRGRWGENESEEKEHP--TTESVT 324 
4-2441    --------------------------------------------------------------------------------------------------------------------------------PFWVNPFGRKPFGDRPFGRRNGTEEGSPRRDGHRHPYGNRGRWGENESEEKEHP--TTESVT 324 
4-2406    FGDHPFGRRNHTEGHQGHNETGDHPHRHHSKTVDGDQDTGHHGHHGHHEHHHHQHDHREGHQDHDRPMFGMRPFRFNPFGRKPFGDHPFGRRNHTEGHQGHNETGDHPHRHHSKTGDGDQDRPMFETRPFWVNPFGRKPFGDRPFDRRNGTEEGSPRRDGHPHPHGNRGRWGENESEEKEHP--TTESVT 445 
4-2425    FGDRPFGRR-------------------------------------------------------------------------------------------------------------------------------------------NGTEEGSPRRDGHRRPYGNRGRWGENESEEKEHP--TTESVT 255 
4-2432    FGDRPFGRR-------------------------------------------------------------------------------------------------------------------------------------------NGTEEGSPRRDGHRRPYGNRGRWGENESEEKEHP--TTESVT 255 
4-2440    LGDRPFGRR-------------------------------------------------------------------------------------------------------------------------------------------NGTEEGSPRRDGHRRPYGNRGRWGENESEEKEHP--TTESVT 255 
4-2402    -------------------------------------------------------------------------------------------------------------------------------------------------------------------------------------------VVT 33  
4-2442    ---------------------------------------------------------------------------------------------------------------------------------------------------------------------------------------------- 1   
4-2443                                                                                                                                                                                                   61  
4-2416                                                                                                                                                                                                   89  
4-2446                                                                                                                                                                                                   142 
4-2403                                                                                                                                                                                                   142 
4-2423                                                                                                                                                                                                   142 
4-2409                                                                                                                                                                                                   142 
4-2434                                                                                                                                                                                                   142 
5-1502    FGDRLFGRR-------------------------------------------------------------------------------------------------------------------------------------------NGTEEGSPRRDGHRRPYGNRGRWGENESEEKEHP--TTESVT 358 
5-1503    FGDRLFGRR-------------------------------------------------------------------------------------------------------------------------------------------NGTEEGSPRRDGHRRPYGNRGRWGENESEEKEHP--TTESVT 358 
5-1504    FGDRLFGRR-------------------------------------------------------------------------------------------------------------------------------------------NGTEEGSPRRDGHRRPYGNRGRWGENESEEKEHP--TTESVT 358 
5-1505    FGDRLFGRR-------------------------------------------------------------------------------------------------------------------------------------------NGTEEGSPRRDGHRRPYGNRGRWGENESEEKEHP--TTESVT 358 
5-1507    FGDRLFGRR-------------------------------------------------------------------------------------------------------------------------------------------NGTEEGSPRRDGHRRPYGNRGRWGENESEEKEHP--TTESVT 358 
5-1509    FGDRLFGRR-------------------------------------------------------------------------------------------------------------------------------------------NGTEEGSPRRDGHRRPYGNRGRWGENESEEKEHP--TTESVT 358 
5-1511    FGDRLFGRR-------------------------------------------------------------------------------------------------------------------------------------------NGTEEGSPRRDGHRRPYGNRGRWGENESEEKEHP--TTESVT 358 
5-1513    FGDRLFGRR-------------------------------------------------------------------------------------------------------------------------------------------NGTEEGSPRRDGHRRPYGNRGRWGENESEEKEHP--TTESVT 358 
5-1514    FGDRLFGRR-------------------------------------------------------------------------------------------------------------------------------------------NGTEEGSPRRDGHRRPYGNRGRWGENESEEKEHP--TTESVT 358 
5-1508    FGDRLFGRR-------------------------------------------------------------------------------------------------------------------------------------------NGTEEGSPRRDGHRRPYGNRGRWGENESEEKEHP--TTESVT 358 
5-1510    SETVSSADATEPRKDLPGVMATVGSMVTEDVGVRMKVRRRSIQRRKA                                                                                                                                                356 
5-2401    FGGRPFGRR-------------------------------------------------------------------------------------------------------------------------------------------NGTEEGSPRRDGHRRPYGNRGRWGENESEEKEHP--TTESVT 355 
5-2402    FGGRPFGRR-------------------------------------------------------------------------------------------------------------------------------------------NGTEEGSPRRDGHRRPYGNRGRWGENESEEKEHP--ATKRKR 355 
5-2404    FGGRPFDRR-------------------------------------------------------------------------------------------------------------------------------------------NGTEEGSPRRDGHRRPYGNRGRWGENESEEKEHP--TTESVT 355 
5-2407    FGGRPFDRR-------------------------------------------------------------------------------------------------------------------------------------------NGTEEGSPRRDGHRRPYGNRGRWSENESEEKEHP--TTESVT 355 
5-2409    FGGRPFDRR-------------------------------------------------------------------------------------------------------------------------------------------NGTEEGSPRRDGHRRPYGNRGRWGENESEEKEHP--TTESVT 355 
5-2410    FGDRPFGRR-------------------------------------------------------------------------------------------------------------------------------------------NGTEEGSPRRDGQRRPYGNRGRWDENESEEKEHP--TTESVT 286 
5-2411    FGDRPFGRR-------------------------------------------------------------------------------------------------------------------------------------------NGTEEGSPRRDGQRRPYGNRGRWGENESEEKEHP--TTESVT 286 
5-2413    FGDRPFGRR-------------------------------------------------------------------------------------------------------------------------------------------NGTEEGSPRRDGQRRPHGNRGRWGENESEEKEHP--TTESVT 286 
5-2414    FGDRPFGRR-------------------------------------------------------------------------------------------------------------------------------------------NGTEEGSPRRDGQRRPYGNRGRWGENESEEKEHP--TTESVT 286 
5-2415    FGDRPFGRR-------------------------------------------------------------------------------------------------------------------------------------------NGTEEGSPRRDGQRRPYGNRGRWGENESEEKEHP--TTESVT 286 
5-2403    --------------------------------------------------------------------------------------------------------------------------------PFWVNPFGRKPFGDRPFGRRNGTEEGSPRRDGHRHPYGNRGRWGENESEEKEHP--TTESVT 324 
5-2406    FGDRPFGRR-------------------------------------------------------------------------------------------------------------------------------------------NGTEEGSPRRDGQRRPHGNRGRWGENESEEKEHP--TTESVT 255 
5-2412                                                                                                                                                                                                   132 
5-2408                                                                                                                                                                                                   67  


                  580       590       
          ....|....|....|....|....|
1-1515    TSSPP                     291 
1-1523    TSSPP                     291 
1-1504    TSSPP                     291 
1-1533    TSSPP                     291 
1-1547                              142 
1-1549                              142 
1-1505                              142 
1-1512                              142 
1-1514                              142 
1-1528                              142 
1-1532                              142 
1-1539                              142 
1-1536                              142 
1-1535                              142 
1-1534                              142 
1-2402    TSSPP                     291 
1-2404    TSSPP                     291 
1-2405    TSSPP                     291 
1-2406    TSSPP                     291 
1-2407    TSSPP                     291 
1-2412    TSSPPEVVEIA-----INDVAEV   304 
1-2413    TSSPP                     291 
1-2416    TSSPP                     291 
1-2417    TSSPP                     291 
1-2418    TSSPP                     291 
1-2420    TSSPP                     291 
1-2421    TSSPP                     291 
1-2422    TSSPP                     291 
1-2425    TSSPP                     291 
1-2426    TSSPP                     291 
1-2427    TSSPP                     291 
1-2428    TSSPP                     291 
1-2430    TSSPP                     291 
1-2431    TSSPP                     291 
1-2432    TSSPP                     291 
1-2433    TSSPP                     291 
1-2434    TSSPP                     291 
1-2435    TSSPP                     291 
1-2436    TSSPP                     291 
1-2437    TSSPP                     291 
1-2439    TSSPP                     291 
1-2440    TSSPP                     291 
1-2441    TSSPP                     291 
1-2442    TSSPP                     291 
1-2414    TSSPPEVV--AINEEDINVVAEV   376 
1-2424                              58  
1-2429                              58  
2-1501    TSSPPEVVEIAFNEEDVNVVAEV   381 
2-1502    TSSPPEVVEIAFNEEDVNVVAEV   381 
2-1505    TSSPPAVVEIAFNEEDAKVVAEV   381 
2-1506    TSPPPEVVEIAFNEEDVNVVAEV   381 
2-1507    TSSPPEVVEIAFNEEDVNVVAEV   381 
2-1508    TSSPPEVVEIAFNEEDVNVVAEV   381 
2-1509    TSSPPEVVEIAFNEEDVNVVAEV   381 
2-1510    TSSPPEVVEIAFNEEDVNVVAEV   381 
2-1514    TSSPPEVVEIAFNEEDVNVVAEV   381 
2-1511    TSSPPEVVEIAFNEEDVNVVAEV   381 
2-2401    TSSPP                     290 
2-2404    TSSPP                     290 
2-2406    TSSSP                     290 
2-2407    TSSPP                     290 
2-2408    TSSPP                     290 
2-2409    TSSPP                     290 
2-2411    TSSPP                     290 
2-2405    TSSPPEVVEIAVNEEDVNVVAEV   341 
2-2413                              266 
2-2403    TSSPPEVVEIAIN--D---VAEV   274 
2-2415    TYSPP                     360 
2-2414    TSSPPEVV--AINEEDINVVAEV   376 
3-15-1006 TSSPPEVVEIAFNEEDVNVVAEV   381 
3-15-4003 TSSPPEVVEIAFNEEDVNVVAEV   381 
3-15-4004 TSSPPEVVEIAFNEEDVNVVAEV   381 
3-15-4005 TSSPPEVVEIAFNEEDVNVVAEV   381 
3-15-4007 TSSPPEVVEIAFNEEDVNVVAEV   381 
3-15-4011 TSSPPEVVEIAFNEEDVNVVAEV   381 
3-15-4013 TSSPPEVVEIAFNEEDVNVVAEV   381 
3-15-4015 TSSPPEVVEIAFNEEDVNVVAEV   381 
3-15-4018 TSSPPEVVEIAFNEEDVNVVAEV   381 
3-15-4019 TSSPPEVVEIAFNEEDVNVVAEV   381 
3-15-4022 TSSPPEVVEIAFNEEDVNVVAEV   381 
3-15-4024 TSSPPEVVEIAFNEEDVNVVAEV   381 
3-15-4017                           356 
3-15-1003                           157 
3-15-4021                           131 
3-15-1002                           120 
3-15-1004                           131 
3-15-4008                           62  
3-24-4003 TSSPP                     291 
3-24-4004 TSSPP                     291 
3-24-1006 TSSPP                     291 
3-24-4006 TSSPP                     291 
3-24-4015 TSSPP                     291 
3-24-4021 TSSPP                     291 
3-24-4024 TSSPP                     291 
3-24-4023 TSSPP                     291 
3-24-4001 TSSPP                     290 
3-24-4019 TSSPP                     290 
3-24-1003 TSSPPEVVEIAIN--D---VAEV   273 
3-24-4017 TSSPPEVV--AINEEDINVVAEV   376 
3-24-4016                           353 
3-24-4011 TSSPPEVVEIAFNEEDVNVVAEV   381 
3-24-4005 TSSPPEVVEIAFNEEDVNVVAEV   381 
3-24-4018 TSSPPEVVEIAFNEEDVNVVAEV   381 
3-24-4022                           157 
6-2415    TSSPPEVVEIAIN--D---VAEV   273 
6-2426    TSSPPEVVEIAIN--D---VAEV   273 
6-2446    TSSPPEVVEIAIN--D---VAEV   273 
6-2401    TSSPP                     291 
6-2402    TSSPP                     291 
6-2404    TSSPP                     291 
6-2407    TSSPP                     291 
6-2408    TSSPP                     291 
6-2409    TSSPP                     291 
6-2410    TSSPP                     291 
6-2411    TSSPP                     291 
6-2412    TSSPP                     291 
6-2413    TSSPP                     291 
6-2414    TSSPP                     291 
6-2416    TSSPP                     291 
6-2417    TSSPP                     291 
6-2420    TPSPP                     291 
6-2421    TSSPP                     291 
6-2422    TSSPP                     291 
6-2424    TSSPP                     291 
6-2425    TSSPP                     291 
6-2427    TSSPP                     291 
6-2428    TSSPP                     291 
6-2430    TSSPP                     291 
6-2431    TSSPP                     291 
6-2432    TSSPP                     291 
6-2433    TSSPP                     291 
6-2434    TSSPP                     291 
6-2439    TSSPP                     291 
6-2440    TSSPP                     291 
6-2441    TSSPP                     291 
6-2435    TSSPP                     291 
6-2447    TSSPP                     291 
6-2448    TSSPP                     291 
6-2436    TSSPP                     291 
6-2450    TSSPP                     291 
6-2429    TSSPPEVV--AINEEDINVVAEV   352 
6-2438    TSSPPEVVEIAVNEEDVNVVAEV   347 
6-2423    TSSPPEVVEIAVNEEDVNVVAEV   347 
6-2403                              156 
6-2444                              142 
6-2449                              3   
2-1503                              142 
2-1509                              142 
2-1513                              142 
2-1523                              142 
2-1524                              142 
2-1531                              142 
2-1533                              142 
2-1536                              142 
2-1502    TSSPP                     291 
2-1518    TSSPP                     291 
2-1519    TSSPP                     291 
2-1511    TSSPP                     291 
2-1546    TSSPP                     291 
2-1548    TSSPP                     291 
2-1540                              132 
2-2423                              142 
2-2436                              142 
2-2405    TSSPP                     291 
2-2403    TSSPP                     291 
2-2448    TSSPP                     291 
2-2404    TSSPP                     291 
2-2406    TSSPP                     291 
2-2409    TSSPP                     291 
2-2410    TSSPP                     291 
2-2411    TSSPP                     291 
2-2412    TSSPP                     291 
2-2413    TSSPP                     291 
2-2415    TSSPP                     291 
2-2416    TSSPP                     291 
2-2417    TSSPP                     291 
2-2418    TSSPP                     291 
2-2419    TSSPP                     291 
2-2420    TSSPP                     291 
2-2421    TSSPP                     291 
2-2422    TSSPP                     291 
2-2424    TSSPP                     291 
2-2425    TSSPP                     291 
2-2426    TSSPP                     291 
2-2427    TSSPP                     291 
2-2430    TSSPP                     291 
2-2431    TSSPP                     291 
2-2432    TSSPP                     291 
2-2434    TSSPP                     291 
2-2437    TSSPP                     291 
2-2438    TSSPP                     291 
2-2439    TSSPP                     291 
2-2440    TSSPP                     291 
2-2442    TSSPP                     291 
2-2445    TSSPP                     291 
2-2446    TSSPP                     291 
7-1501                              142 
7-1502                              142 
7-1503                              142 
7-1504                              142 
7-1505                              142 
7-1506                              142 
7-1508                              142 
7-1509                              142 
7-1510                              142 
7-1511                              142 
7-1512                              142 
7-1513                              142 
7-1515                              142 
7-1516                              142 
7-1517                              142 
7-1519                              142 
7-1520                              142 
7-1521                              142 
7-1523                              142 
7-1524                              142 
7-1525                              142 
7-1526                              142 
7-1527                              142 
7-1528                              142 
7-1529                              142 
7-1530                              142 
7-1533                              142 
7-1534                              142 
7-1536                              142 
7-1537                              142 
7-1538                              142 
7-1542                              142 
7-1544                              142 
7-1545                              142 
7-1546                              142 
7-1548                              142 
7-1549                              142 
7-1539                              142 
7-1550                              142 
7-1518    TSSPP                     291 
7-1540                              79  
7-1514    TSSPP                     291 
7-1507    TSSPP                     291 
7-1522    TSSPP                     291 
7-1532    TSSPP                     291 
7-1543    TSSPP                     291 
7-1547    TSSPP                     291 
7-2401                              142 
7-2402                              142 
7-2403                              142 
7-2404                              142 
7-2405                              142 
7-2406                              142 
7-2407                              142 
7-2408                              142 
7-2409                              142 
7-2410                              142 
7-2411                              142 
7-2412                              142 
7-2413                              142 
7-2414                              142 
7-2416                              142 
7-2417                              142 
7-2418                              142 
7-2420                              142 
7-2421                              142 
7-2423                              142 
7-2424                              142 
7-2425                              142 
7-2426                              142 
7-2430                              142 
7-2431                              142 
7-2432                              142 
7-2436                              142 
7-2437                              142 
7-2439                              142 
7-2442                              142 
7-2443                              142 
7-2444                              142 
7-2445                              142 
7-2446                              142 
7-2447                              142 
7-2448                              142 
7-2450                              142 
7-2415    TSSPP                     291 
7-2427    TSSPP                     291 
7-2428    TSSPP                     291 
7-2435    TSSPP                     291 
7-2440    TSSPP                     291 
7-2441    TSSPP                     291 
7-2449    TSSPP                     291 
8-1501                              142 
8-1502                              142 
8-1503                              142 
8-1504                              142 
8-1505                              142 
8-1506                              142 
8-1507                              142 
8-1508                              142 
8-1509                              142 
8-1510                              142 
8-1511                              142 
8-1512                              142 
8-1513                              142 
8-1514                              142 
8-1515                              142 
8-1516                              142 
8-1517                              142 
8-1518                              142 
8-1519                              142 
8-1520                              142 
8-1521                              142 
8-1522                              142 
8-1524                              142 
8-1525                              142 
8-1526                              142 
8-1527                              142 
8-1528                              142 
8-1529                              142 
8-1530                              142 
8-1531                              142 
8-1532                              142 
8-1533                              142 
8-1534                              142 
8-1535                              142 
8-1536                              142 
8-1537                              142 
8-1538                              142 
8-1539                              142 
8-1540                              142 
8-1542                              142 
8-1546                              142 
8-1548                              142 
8-1549                              142 
8-1550                              142 
8-1541    TSSPP                     291 
8-1543    TSSPP                     291 
8-2401                              142 
8-2450                              142 
8-2406                              142 
8-2407                              142 
8-2409                              142 
8-2410                              142 
8-2411                              142 
8-2414                              142 
8-2417                              142 
8-2420                              142 
8-2421                              142 
8-2422                              142 
8-2424                              142 
8-2425                              142 
8-2427                              142 
8-2433                              142 
8-2434                              142 
8-2435                              142 
8-2436                              142 
8-2437                              142 
8-2438                              142 
8-2439                              142 
8-2440                              142 
8-2442                              142 
8-2444                              142 
8-2445                              142 
8-2448                              142 
8-2449                              142 
8-2432    TSSPPEVV--AINEEDINVVAEV   376 
8-2405    TSSPPEVV--AINEEDINVVAEV   376 
8-2443    TSSPP                     291 
8-2413    TSSPP                     291 
8-2418    TSSPP                     291 
8-2419    TSSPP                     291 
8-2423    TSSPP                     291 
8-2430    TSSPP                     291 
8-2446    TSSPP                     291 
8-2447    TSSPP                     291 
8-2441    TSSPPEVVEIAIN--D---VAEV   273 
8-2404    TSSPP                     291 
8-2415                              224 
2-1517                              142 
2-1501                              142 
2-1503                              142 
2-1505                              142 
2-1508                              142 
2-1509                              142 
2-1510                              142 
2-1519                              142 
2-1521                              142 
2-1522                              142 
2-1523                              142 
2-1527                              142 
2-1533                              142 
2-1535                              142 
2-1536                              142 
2-1537                              142 
2-1538                              142 
2-1539                              142 
2-1543                              142 
2-1546                              142 
2-1547                              142 
2-1529    TSSPPEVVEIAFNEEDVNVVAEV   381 
2-1540    TSSPPEVV--AINEEDINVVAEV   376 
2-1506    TSSPP                     291 
2-1507    TSSPP                     291 
2-1511    TSSPP                     291 
2-1514    TSSPP                     291 
2-1516    TSSPP                     291 
2-1528    TSSPP                     291 
2-1532    TSSPP                     291 
2-1541    TSSPP                     291 
2-1542    TSSPP                     291 
2-2448                              142 
2-2405                              142 
2-2406                              142 
2-2407                              142 
2-2408                              142 
2-2410                              142 
2-2412                              142 
2-2413                              142 
2-2419                              142 
2-2420                              142 
2-2422                              142 
2-2424                              142 
2-2427                              142 
2-2428                              142 
2-2429                              142 
2-2431                              142 
2-2436                              142 
2-2441                              142 
2-2446                              142 
2-2416                              121 
2-2411                              45  
2-2415                              45  
2-2440                              45  
2-2423    -----                     1   
2-2418                              131 
2-2421    TSSPPEVV--AINEEDINVVAEV   352 
2-2438    TSSPP                     336 
2-2414    TSSPP                     291 
2-2403    TSSPP                     291 
2-2404    TSSPP                     290 
2-2425    TSSPP                     290 
2-2426    TSSPP                     291 
2-2430    TSSPP                     291 
2-2432    TSSPP                     290 
2-2437    TSSPP                     290 
2-2439    TSSPP                     291 
2-2442    TSSPP                     291 
2-2443    TSSPP                     291 
2-2445    TSSPP                     291 
2-2435    TSSPPEVVEIAIN--D---VAEV   274 
2-2447    TSSPPEVVEIAIN--D---VAEV   274 
2-2401    TSSPPEVVEIAIN--D---VAEV   274 
2-2409    TSSPPEVV--AINEEDINVVAEV   376 
2-2444    TSSPPEVV--AINEEDINVVAEV   376 
9-1504                              142 
9-1505                              142 
9-1506                              142 
9-1507                              142 
9-1509                              142 
9-1512                              142 
9-1514                              142 
9-1515                              142 
9-1516                              142 
9-1518                              142 
9-1519                              142 
9-1520                              142 
9-1521                              142 
9-1523                              142 
9-1524                              142 
9-1526                              142 
9-1527                              142 
9-1530                              142 
9-1531                              142 
9-1533                              142 
9-1537                              142 
9-1538                              142 
9-1539                              142 
9-1540                              142 
9-1543                              142 
9-1544                              142 
9-1545                              142 
9-1546                              142 
9-1547                              142 
9-1548                              142 
9-1513    TSSPPEVV--AINEEDINVVAEV   376 
9-1541    TSSPPEVV--AINEEDINVVAEV   376 
9-1542    TSSPPEVV--AINEEDINVVAEV   376 
9-1525    TFSPLKVIEIAINEVDTNVVAEV   489 
9-1501    TSSPP                     291 
9-1502    TSSPP                     291 
9-1508    TSSPP                     291 
9-1511    TSSPP                     291 
9-1517    TSSPP                     291 
9-1528    TSSPP                     291 
9-1529    TSSPP                     291 
9-1534    TSSPP                     291 
9-1535    TSSPP                     291 
9-2401                              142 
9-2402                              142 
9-2404                              142 
9-2406                              142 
9-2408                              142 
9-2409                              142 
9-2411                              142 
9-2413                              142 
9-2414                              142 
9-2417                              142 
9-2418                              142 
9-2419                              142 
9-2420                              142 
9-2421                              142 
9-2424                              142 
9-2427                              142 
9-2429                              142 
9-2430                              142 
9-2433                              142 
9-2435                              142 
9-2438                              142 
9-2439                              142 
9-2440                              142 
9-2441                              142 
9-2442                              142 
9-2443                              142 
9-2445                              142 
9-2446                              142 
9-2448                              142 
9-2416    TSSPPEVV--AINEEDINVVAEV   376 
9-2423    TSSPPEVV--AINEEDINVVAEV   376 
9-2403    TSSPP                     291 
9-2415    TSSPP                     291 
9-2422    TSSPP                     291 
9-2428    TSSPP                     291 
9-2431    TSSPP                     291 
9-2432    TSSPP                     291 
9-2434    TSSPP                     291 
9-2437    TSSPP                     291 
9-2444    TSSPP                     291 
4-1504    TSSPPEVV--AINEEDVNVVAEV   376 
4-1522    X----------------------   332
[truncated: 2,995 more chars]
